# Supplementary material for: Prevalence and Etiological Characteristics of Norovirus Infection in China: A Systematic Review and Meta-Analysis
Source: Viruses. 2023 Jun 7;15(6):1336. doi: 10.3390/v15061336 (PMC10302178; doi:10.3390/v15061336)
Supplement: Supplementary file 1 [file viruses-15-01336-s001.zip › Appendix S2.pdf]

Supplementary Appendix S2

Supplement to: Prevalence and etiological characteristics of norovirus infection in China: a systematic review and meta-analysis

Table of Contents

Appendix Table S1: Literature search syntax of norovirus........3

Appendix Table S2: Criteria applied for title and abstract screening........4

Appendix Table S3: Criteria applied for full text review of articles........5

Appendix Table S4: Data extracted for each article included in the review........6

Appendix Table S5: Reference number of norovirus infection genotypes in China........8

Appendix Table S6: Attack rate with 95% CI of norovirus outbreaks for each year in North China and South China of figure 3A........12

Appendix Table S7: Attack rate with 95% CI of norovirus outbreaks for each month in North China and South China of figure 3B........13

Appendix Table S8: Attack rate with 95% CI of norovirus outbreaks in seven ecological regions of appendix figure S2........14

Appendix Table S9: Attack rate with 95% CI of norovirus outbreaks for each month in different age groups of figure 3C........15

Appendix Table S10: Attack rate with 95% CI of norovirus outbreaks for each month in in settings of figure 3D........17

Appendix Table S11: Attack rate with 95% CI of norovirus outbreaks for each genotype in North China and South China of figure 4A........21

Appendix Table S12: Proportions with 95% CI of clinical presentations of norovirus infection in different age groups of appendix figure S4A........22

Appendix Table S13: Proportions with 95% CI of clinical presentations of norovirus infection in different GI genotypes of appendix figure S4B........24

Appendix Table S14: Proportions with 95% CI of clinical presentations of norovirus infection in different GII genotypes of appendix figure S4C........26

Appendix Table S15: Reference number for analyzed risk factors of attack rate with Beta-Binomial Model........29

Appendix Table S16: Comparative analysis of the attack rate by the Beta-Binomial Model........30

Appendix Figure S1: Temporal distribution of recombinant norovirus genotypes.........31

Appendix Figure S2: Distribution of norovirus outbreaks in seven ecoclimatic regions, China. (A) number and attack rate, (B) Genotype. 32

Appendix Figure S3: The number of norovirus outbreaks of transmission routes in different settings in China (A), and the proportion of norovirus outbreaks of transmission routes in different settings in China (B). 33

Appendix Figure S4: Clinical manifestations in patients infected with norovirus by age (A), GI genogroup (B) and GII genogroup (C)..34

Appendix Table S1: Literature search syntax of norovirus

Literature database	Website	Query
PubMed	https://pubmed.ncbi.nlm.nih.gov/, accessed on 1 April 2021.	((Norovirus [MeSH Terms]) OR (Norwalk virus [MeSH Terms])) AND (China), Filters: from 2009/1/1—2021/3/31
China National Knowledge Infrastructure (CNKI)	http://www.cnki.net/, accessed on 1 April 2021.	(主题=诺如病毒) OR (主题=诺瓦克病毒) OR (主题=诺瓦克样病毒) AND 发表时间: 2009-2021
Wanfang Database (Wanfang)	http://www.wanfangdata.com.cn/, accessed on 1 April 2021.	(主题:(诺如病毒) or 主题:(诺瓦克病毒) or 主题:(诺瓦克样病毒)) and Date: 2009-2021
Chongqing VIP Chinese Science and Technology Journal Database (CQVIP)	http://www.cqvip.com.cn/, accessed on 1 April 2021.	((题名或关键词=诺如病毒 OR 题名或关键词=诺瓦克病毒) OR 题名或关键词=诺瓦克样病毒) AND (years:[2009 TO 2021]))

Appendix Table S2: Criteria applied for title and abstract screening

Criteria	Guidance	Outcome
#1: Norovirus	Does the Title/Abstract relate to norovirus?	If Yes, remain and evaluate #2.<br>If No, exclude.
#2: Year of study	Does the Title/Abstract refer the year of study which is 2009 or later?	If Yes, remain and evaluate #3.<br>If No, exclude.
#3: Human infection	Does the Title/Abstract refer the norovirus which are in human infection?	If Yes, remain and evaluate #4.<br>If No, exclude.
#4: Study site	Does the Title/Abstract refer the study site which is the mainland of China?	If Yes, remain and evaluate #5.<br>If No, exclude.
#5: Not review	Does the Title/Abstract refer the article which is not a review? (Not reviewing the published articles, with presenting new primary data)	If No, remain for full text review.<br>If Yes, exclude.

Appendix Table S3: Criteria applied for full text review of articles

Criteria	Guidance	Outcome
#1: Re-screening	Does the article meet the screening criteria before?<br>1-norovirus<br>2-in human infection<br>3-in the mainland of China<br>4-not review<br>5-not drug or vaccine trials<br>6-study period was between 2009 and 2021	If Yes, remain and evaluate #2.<br>If No, exclude.
#2: Study design	Does the study is designed as an etiological surveillance or outbreak investigation?	If Yes, remain and evaluate #3.<br>If No, exclude.
#3: Repeat study	Does the article refer the data which is duplicate with other articles? (The repeated etiological surveillance: study time, author, research unit and project. The repeated outbreak events: outbreak time, location, and the number of cases.)	If Yes, exclude.<br>If No, remain for data extracting.

Appendix Table S4: Data extracted for each article included in the review

Variable ID	Criteria	Guidance
1	Reference ID	Unique identifier assigned to an article.
2	Article title	Article title that included in the review.
3	Authors	Authors of the included article.
4	Publication year	Publication year of the article identified for data extraction.
5	Study duration	Record the start and end year for the period over which the reported study was conducted.
6	Study design	Classify each record as: etiological surveillance and outbreak investigation.
7	Geographic location	Province (geographic location information recorded at province-level), Prefecture (geographic location information recorded at prefecture-level), County (geographic location information recorded at county-level).
8	Area	Classify each record as: I) North II) South, III) unspecified area category.
9	Ecological region	Classify the geographical location as: I) Northeast, II) North China, III) Inner Mongolia-Xinjiang, IV) Qinghai-Tibet, V) Southwest, VI) Central China, and VII) South China.
10	Month	The month of norovirus detection in etiological surveillance and month in which the norovirus outbreak occurred.
11	Season	According to the average monthly temperature of the sentinel city, the top six months were classified as "warm months", while the rest were "cold months".
12	Age group	Classify each record as: I) 0–4 years old, II) 5–17 years old, III) 18–59 years old, IV) ≥60 years old, and V) unspecified age category.
13	Route of transmission	Foodborne: e.g., consumption of contaminated shellfish or foods contaminated during production or preparation by an infected food handler, waterborne: e.g., drinking or recreational water, and human-to-human: direct contact with infected vomitus or other aerosolized virus particles.
14	Setting	Childcare center, primary school, middle school, university, community, nursing home, hospital, restaurant, workplace and others: e.g., household, transportation, welfare, military setting, prison, multiple setting types and multiple school types.
15	Genotype	Record the genotypes and the positive number: (GI.1, GI.2, GI.3, GI.4, GI.5, GI.6, GI.7, GI.8, GI.9, GI.Pd, GI.5[P4], GI.5[P2], GI.6[P11], GI.3[P13], GI.6[Pb], GI.3[Pd], GII.1,GII.P1,GII.2, GII.P2,GII.3, GII.P3, GII.4, GII.P4, GII.5, GII.P5, GII.6, GII.P6, GII.7, GII.P7, GII.8, GII.P8, GII.10, GII.11, GII.12, GII.P12, GII.13, GII.P13, GII.14, GII.15, GII.16, GII.P16, GII.17, GII.P17, GII.21, GII.P21, GII.22, GII.P22, GII.P31, GII.b, GII.e, GII.g, GII.Pe, GII.Pg, GIV.1, GII.3[P4], GII.4Sydney_2012[P4], GII.4_2006b[P4], GII.4New_Orleans[P4], GII.4New_Orleans[P4_Sydney,2012], GII.7[P6], GII.6[P7], GII.9[P7], GII.14[P7], GII.2[P12], GII.3[P12], GII.4[P12], GII.4Sydney_2012[P12], GII.17[P12], GII.1[P16], GII.2[P16], GII.3[P16], GII.13[P16], GII.4[P16], GII.4Sydney_2012[P16], GII.13[P21], GII.3[P21], GII.5[P22], GII.3[P25], GII.4[P31], GII.4Sydney_2012[P31], GII.42006b[Pa], GII.2[Pe], GII.3[Pe], GII.9[Pe], GII.4[Pe], GⅡ.4Sydney_2012[Pe], GII.17[Pe], GII.1[Pg], GII.12[Pg]), GIV.1[P15]).
16	Outbreak duration	Record the start and end date for the period over which the outbreak was occured.

17	Primary case	Record the number of primary cases.
18	Person at risk	Record the number of persons at risk.
19	Primary attack rate	Record the number of primary cases / the number of persons at risk.
20	Clinical presentation	Record the clinical presentations (including vomiting, diarrhea, fever, abdominal pain, nausea, headache, dizziness, abdominal distension) and the positive number.

Appendix Table S5: Reference number of norovirus infection genotypes in China

Genotype	Reference ID
GI.1	121, 122, 123, 131, 165, 238, 273, 627, 987, 1002, 1055, 1057, 1071, 1110, 1120, 1122, 1128, 1132
GI.2	56, 97, 110, 121, 122, 130, 131, 273, 312, 513, 615, 666, 887, 912, 1002, 1009, 1055, 1058, 1113, 1119
GI.3	46, 122, 130, 131, 165, 215, 271, 331, 331, 465, 471, 606, 635, 645, 777, 912, 1006, 1006, 1040, 1055, 1057, 1058, 1071, 1072, 1102, 1121, 1122, 1128, 1132
GI.4	93, 97, 110, 114, 121, 131, 273, 635, 777, 791, 1006, 1008, 1009, 1040, 1057, 1058, 1120, 1121, 1122, 1128, 1130
GI.5	97, 114, 121, 777, 782, 1002, 1014, 1015, 1040, 1079, 1121, 1122, 1130
GI.6	93, 97, 130, 131, 268, 349, 465, 534, 641, 777, 788, 862, 887, 911, 912, 924, 1003, 1062, 1071, 1119, 1130
GI.7	56, 130, 268, 648, 731, 777, 1002, 1003, 1040, 1121
GI.8	130, 648, 777, 838, 1055, 1057
GI.9	606, 635, 838, 887, 1055
GI.Pd	779
GII.1	102, 156, 394, 637, 646, 728, 1027, 1108, 1120, 1130
GII.P1	1002
GII.2	5, 52, 56, 71, 92, 97, 98, 114, 130, 155, 156, 177, 228, 260, 268, 347, 349, 355, 382, 393, 465, 474, 577, 587, 637, 664, 691, 714, 731, 732, 746, 749, 777, 786, 791, 843, 857, 858, 911, 924, 988, 991, 997, 1003, 1014, 1017, 1026, 1027, 1036, 1038, 1040, 1047, 1049, 1057, 1075, 1093, 1108, 1115, 1120, 1121, 1128, 1132
GII.P2	71, 1027
GII.3	5, 56, 71, 97, 98, 114, 121, 127, 130, 155, 156, 165, 215, 228, 268, 289, 299, 333, 349, 353, 354, 355, 357, 360, 382, 389, 394, 465, 523, 530, 543, 577, 622, 632, 637, 644, 662, 698, 714, 731, 747, 777, 788, 790, 808, 843, 858, 864, 887, 911, 984, 988, 991, 997, 1003, 1010, 1011, 1014, 1018, 1027, 1036, 1038, 1040, 1047, 1048, 1049, 1053, 1057, 1059, 1062, 1073, 1084, 1089, 1093, 1105, 1107, 1108, 1112, 1115, 1120, 1121, 1122, 1127, 1128, 1132
GII.P3	219, 1002, 1072
GII.4	1, 5, 18, 19, 25, 26, 41, 43, 56, 58, 59, 71, 78, 88, 93, 97, 98, 102, 110, 114, 121, 122, 130, 131, 134, 155, 156, 160, 165, 213, 215, 228, 238, 264, 268, 275, 287, 289, 299, 311, 312, 333, 340, 353, 354, 355, 357, 360, 375, 382, 389, 394, 430, 464, 465, 466, 471, 509, 523, 530, 543, 577, 591, 592, 594, 611, 612, 615, 622, 632, 635, 637, 638, 644, 648, 662, 692, 698, 714, 724, 728, 741, 747, 752, 764, 777, 788, 790, 791, 833, 837, 843, 854, 858, 864, 866, 887, 903, 960, 984, 988, 991, 996, 997, 1001, 1003, 1005, 1010, 1011, 1014, 1017, 1026, 1027, 1028, 1031, 1032, 1033, 1036, 1038, 1040, 1045, 1047, 1048, 1049, 1053, 1057, 1059, 1061, 1062, 1063, 1064, 1072, 1073, 1078, 1079, 1081, 1084, 1089, 1093, 1095, 1098, 1099, 1100, 1105, 1107, 1108, 1110, 1111, 1112, 1113, 1115, 1118, 1119, 1120, 1121, 1122, 1127, 1128, 1130, 1132
GII.P4	219, 632, 1011, 1027
GII.5	56, 102, 110, 471, 728, 866, 997, 1014, 1040, 1110, 1121, 1130
GII.P5	219
GII.6	5, 56, 71, 93, 97, 110, 114, 121, 122, 130, 202, 228, 238, 268, 287, 333, 353, 355, 382, 465, 471, 513, 543, 544, 547, 577, 587, 632, 648, 698, 714, 731, 843, 911, 988, 996, 997, 1002, 1003, 1011, 1014, 1017, 1027, 1028, 1030, 1036,

Genotype	Reference ID
	1038, 1040, 1045, 1047, 1049, 1057, 1064, 1071, 1076, 1079, 1093, 1105, 1108, 1110, 1111, 1112, 1113, 1115, 1118, 1120, 1121, 1122, 1132
GII.P6	632, 1027
GII.7	71, 110, 134, 160, 202, 232, 287, 352, 547, 731, 887, 996, 997, 1017, 1026, 1027, 1036, 1038, 1039, 1040, 1047, 1049, 1093, 1105, 1112, 1115, 1121, 1132
GII.P7	71, 355, 632, 1002, 1027, 1058
GII.8	56, 843, 848, 997, 1002, 1013, 1017, 1027, 1062
GII.P8	1027
GII.10	357
GII.11	102, 728
GII.12	61, 114, 130, 163, 296, 334, 389, 530, 827, 911, 996, 997, 1026, 1040, 1047, 1048, 1049, 1112, 1121, 1128, 1132
GII.P12	71, 156, 219, 355, 632, 714, 996, 1011, 1022, 1027
GII.13	71, 97, 110, 121, 123, 130, 228, 333, 353, 354, 465, 632, 731, 777, 858, 988, 997, 1003, 1010, 1011, 1014, 1047, 1057, 1072, 1108, 1110, 1120, 1122
GII.P13	215, 1002
GII.14	71, 131, 465, 543, 637, 777, 1006, 1017, 1049, 1093, 1113, 1128, 1132
GII.15	465, 632, 913, 1013, 1028
GII.16	130, 190, 887
GII.P16	71, 156, 355, 632, 714, 991, 1027
GII.17	30, 56, 60, 71, 122, 156, 202, 270, 276, 285, 312, 333, 349, 355, 356, 456, 465, 513, 540, 543, 560, 572, 577, 614, 646, 649, 666, 671, 699, 716, 731, 745, 757, 778, 788, 821, 843, 888, 912, 964, 986, 991, 998, 1003, 1007, 1008, 1009, 1013, 1017, 1037, 1043, 1046, 1053, 1072, 1077, 1080, 1105, 1112
GII.P17	5, 71, 156, 219, 355, 714, 1027, 1072
GII.21	56, 71, 97, 228, 577, 731, 858, 997, 1002, 1013
GII.P21	71, 572, 1072
GII.22	572, 632
GII.P22	632
GII.P31	991
GII.b	130, 238, 271, 1026, 1064, 1110
GII.e	130, 594
GII.g	130, 131
GII.Pe	5, 71, 156, 355, 632, 714, 779, 1002, 1027, 1058, 1072
GII.Pg	164, 1027, 1058
GIV.1	983
GI.5[P4]	270, 273, 582, 1009
GI.5[P2]	273

Genotype	Reference ID
GI.6[P11]	122, 303, 1009
GI.3[P13]	122, 303, 312
GI.6[Pb]	273, 731, 1002, 1003, 1063, 1072
GI.3[Pd]	273, 779
GII.3[P4]	122
GII.4Sydney_2012[P4]	56, 122, 1112
GII.4_2006b[P4]	1112
GII.4New_Orleans[P4]	1112
GII.4New_Orleans[P4Sydney_2012]	788
GII.7[P6]	1009, 1013
GII.6[P7]	63, 71, 122, 190, 269, 287, 312, 355, 375, 543, 590, 632, 731, 748, 788, 843, 850, 995, 1002, 1003, 1058, 1105, 1112
GII.9[P7]	572, 1013
GII.14[P7]	71, 543, 1013
GII.2[P12]	991
GII.3[P12]	71, 84, 122, 156, 190, 287, 296, 347, 355, 375, 543, 560, 606, 632, 699, 748, 788, 843, 850, 912, 991, 996, 1011, 1013, 1028, 1058, 1063, 1072, 1093, 1105, 1106, 1111, 1112, 1132
GII.4[P12]	560, 731, 1002
GII.4Sydney_2012[P12]	991
GII.17[P12]	560
GII.1[P16]	190, 991, 1009
GII.2[P16]	30, 84, 117, 122, 155, 156, 272, 312, 355, 404, 511, 560, 561, 606, 699, 715, 788, 843, 850, 857, 906, 991, 1000, 1008, 1009, 1013, 1016, 1020, 1028, 1043, 1106, 1111
GII.3[P16]	122, 155
GII.13[P16]	71, 155, 375, 572, 632, 748, 912, 1003, 1009, 1013, 1028, 1058
GII.4[P16]	606, 122, 1013
GII.4Sydney_2012[P16]	71, 122, 991
GII.13[P21]	122, 560, 843, 991, 1003, 1008, 1013, 1111
GII.3[P21]	1028, 1112
GII.5[P22]	632, 1013
GII.3[P25]	991
GII.4[P31]	122, 312, 850
GII.42006b[Pa]	1112

Genotype	Reference ID
GII.2[Pe]	156
GII.3[Pe]	156, 355
GII.9[Pe]	572
GII.4[Pe]	71, 155, 156, 190, 287, 296, 355, 484, 543, 561, 572, 606, 632, 699, 731, 748, 843, 912, 1002, 1003, 1011, 1013, 1043, 1058, 1063, 1072, 1105, 1106
GII.17[Pe]	71, 1013
GII.1[Pg]	164, 1013
GII.12[Pg]	296
GVI.1[P15]	991

Appendix Table S6: Attack rate with 95% CI of norovirus outbreaks for each year in North China and South China of figure 3A

Year of publication	Area	Attack rate by fixed effect % (95% CI)	Attack rate by Random effect % (95% CI)	I^2 (%)	Attack rate (%)
2009	North	--	--	--	--
	South	1·91 (1·41, 2·57)	1·91 (1·41, 2·57)	--	1·91
2010	North	--	--	--	--
	South	2·59 (2·37, 2·84)	5·19 (2·38, 10·94)	98·90	5·19
2011	North	--	--	--	--
	South	1·91 (1·82, 2·00)	6·64 (1·94, 20·38)	99·70	6·64
2012	North	1·36 (1·29, 1·43)	3·79 (0·42, 26·85)	99·90	3·79
	South	5·32 (5·07, 5·59)	7·69 (4·35, 13·24)	98·70	7·69
2013	North	--	--	--	--
	South	2·61 (2·52, 2·70)	5·71 (3·76, 8·58)	99·20	5·71
2014	North	4·99 (4·17, 5·97)	8·14 (3·02, 20·15)	95·90	8·14
	South	1·34 (1·30, 1·39)	3·44 (2·41, 4·88)	99·20	3·44
2015	North	6·89 (6·28, 7·55)	11·28 (6·83, 18·07)	94·90	11·28
	South	2·26 (2·18, 2·34)	5·72 (3·85, 8·42)	98·90	5·72
2016	North	12·17 (11·25, 13·15)	15·77 (10·23, 23·54)	95·60	15·77
	South	2·45 (2·37, 2·53)	4·38 (3·03, 6·28)	99·00	4·38
2017	North	3·20 (3·10, 3·29)	8·66 (5·41, 13·58)	99·10	8·66
	South	3·58 (3·47, 3·68)	4·93 (3·70, 6·53)	98·10	4·93
2018	North	3·54 (3·41, 3·67)	5·88 (4·13, 8·30)	97·80	5·88
	South	2·99 (2·92, 3·07)	4·85 (3·96, 5·92)	98·30	4·85
2019	North	8·76 (8·42, 9·11)	17·39 (14·46, 20·76)	95·40	17·39
	South	2·55 (2·45, 2·65)	4·86 (3·44, 6·83)	98·60	4·86
2020	North	3·68 (3·43, 3·94)	5·79 (3·90, 8·52)	97·50	5·79
	South	2·11 (2·03, 2·19)	4·28 (3·00, 6·08)	98·60	4·28
2021	North	1·99 (1·73, 2·28)	2·50 (1·31, 4·73)	96·00	2·50
	South	3·81 (3·55, 4·09)	4·56 (2·50, 8·18)	98·60	4·56

Appendix Table S7: Attack rate with 95% CI of norovirus outbreaks for each month in North China and South China of figure 3B

Month	Area	Attack rate by fixed effect % (95% CI)	Attack rate by Random effect % (95% CI)	I^2 (%)	Attack rate (%)
1	North	0·38 (0·34, 0·41)	6·54 (1·81, 21·01)	99·60	6·54
	South	2·48 (2·39, 2·57)	3·92 (2·87, 5·33)	98·40	3·92
2	North	11·07 (9·81, 12·47)	17·68 (9·34, 30·93)	94·50	17·68
	South	2·34 (2·29, 2·40)	4·68 (3·46, 6·31)	98·90	4·68
3	North	3·51 (3·38, 3·64)	9·81 (7·07, 13·46)	98·40	9·81
	South	2·10 (2·05, 2·17)	4·99 (3·74, 6·62)	99·20	4·99
4	North	3·50 (3·31, 3·69)	7·52 (4·83, 11·54)	97·40	7·52
	South	6·02 (5·81, 6·23)	7·28 (5·09, 10·32)	98·60	7·28
5	North	3·73 (3·50, 3·97)	13·57 (9·22, 19·52)	96·90	13·57
	South	3·24 (3·06, 3·43)	4·39 (2·67, 7·14)	97·80	4·39
6	North	7·42 (7·16, 7·70)	10·97 (7·12, 16·52)	97·70	10·97
	South	5·34 (4·85, 5·88)	6·40 (3·62, 11·08)	95·20	6·40
7	North	4·32 (3·70, 5·04)	5·83 (2·68, 12·20)	95·60	5·83
	South	1·80 (1·69, 1·91)	5·00 (1·85, 12·84)	99·50	5·00
8	North	4·14 (--)	4·14 (--)	--	4·14
	South	3·49 (3·20, 3·81)	4·32 (2·56, 7·19)	97·50	4·32
9	North	5·31 (4·79, 5·87)	5·66 (3·70, 8·55)	96·70	5·66
	South	1·96 (1·89, 2·03)	3·89 (2·54, 5·92)	99·00	3·89
10	North	7·37 (7·04, 7·71)	11·72 (5·08, 24·79)	99·60	11·72
	South	1·50 (1·43, 1·57)	4·37 (3·12, 6·09)	98·50	4·37
11	North	2·75 (2·60, 2·91)	8·88 (5·10, 15·03)	98·20	8·88
	South	2·47 (2·40, 2·54)	4·18 (3·19, 5·45)	98·70	4·18
12	North	5·00 (4·61, 5·43)	11·71 (6·27, 20·82)	98·50	11·71
	South	3·11 (3·01, 3·22)	6·12 (4·37, 8·52)	98·50	6·12

Appendix Table S8: Attack rate with 95% CI of norovirus outbreaks in seven ecological regions of appendix figure S2

Setting	Attack rate by fixed effect % (95% CI)	Attack rate by Random effect % (95% CI)	I^2 (%)	Attack rate (%)
Northeast China district	2·47 (2·35, 2·59)	7·35 (4·93, 10·81)	97·90	7·35
North China district	3·24 (3·18, 3·29)	8·96 (7·57, 10·59)	98·80	8·96
Inner Mongolia-Xinjiang district	4·54 (4·08, 5·04)	4·38 (2·75, 6·92)	96·30	4·38
Qinghai-Tibet district	--	--	--	--
Southwest China district	--	--	--	--
Central China district	3·34 (3·30, 3·39)	5·07 (4·45, 5·78)	98·40	5·07
South China district	1·57 (1·54, 1·60)	4·80 (3·76, 6·12)	99·30	4·80

Appendix Table S9: Attack rate with 95% CI of norovirus outbreaks for each month in different age groups of figure 3C

Month	Age group	Attack rate by fixed effect % (95% CI)	Attack rate by Random effect % (95% CI)	I^2 (%)	Attack rate (%)
1	0–4Y	5·38 (4·50, 6·42)	5·96 (3·13, 11·06)	90·60	5·96
	5–17Y	3·12 (2·95, 3·30)	3·29 (2·05, 5·26)	96·50	3·29
	18–60Y	1·94 (1·83, 2·05)	9·14 (4·00, 19·53)	99·70	9·14
	≥60Y	7·56 (5·72, 9·39)	13·14 (7·22, 19·05)	87·00	13·14
2	0–4Y	8·48 (7·67, 9·36)	8·19 (5·48, 12·07)	92·80	8·19
	5–17Y	4·60 (4·44, 4·77)	3·92 (3·04, 5·04)	96·60	3·92
	18–60Y	0·77 (0·72, 0·82)	5·50 (1·90, 14·91)	99·30	5·50
	≥60Y	27·85 (--)	27·85 (--)	--	27·85
3	0–4Y	5·02 (4·55, 5·53)	8·06 (4·49, 14·04)	98·00	8·06
	5–17Y	4·99 (4·80, 5·19)	6·55 (4·80, 8·88)	97·40	6·55
	18–60Y	2·74 (2·65, 2·84)	7·47 (4·10, 13·23)	99·40	7·47
	≥60Y	16·05 (--)	16·05 (--)	--	16·05
4	0–4Y	5·38 (4·50, 6·42)	5·96 (3·13, 11·06)	90·60	5·96
	5–17Y	3·12 (2·95, 3·30)	3·29 (2·05, 5·26)	96·50	3·29
	18–60Y	1·94 (1·83, 2·05)	9·14 (4·00, 19·53)	99·70	9·14
	≥60Y	--	--	--	--
5	0–4Y	7·70 (6·76, 8·77)	17·70 (9·44, 30·73)	95·60	17·70
	5–17Y	3·92 (3·71, 4·15)	9·50 (6·10, 14·49)	97·80	9·50
	18–60Y	1·47 (1·29, 1·68)	1·41 (0·23, 8·05)	98·80	1·41
	≥60Y	--	--	--	--
6	0–4Y	9·78 (7·90, 12·06)	11·77 (4·16, 29·10)	94·70	11·77
	5–17Y	4·38 (4·03, 4·76)	8·21 (4·64, 14·12)	95·90	8·21
	18–60Y	7·91 (7·60, 8·23)	9·27 (5·60, 14·97)	99·30	9·27
	≥60Y	--	--	--	--
7	0–4Y	7·03 (4·72, 10·36)	7·94 (1·76, 29·29)	95·50	7·94
	5–17Y	2·85 (2·33, 3·49)	4·19 (1·40, 11·87)	97·10	4·19
	18–60Y	4·34 (3·97, 4·75)	3·17 (0·54, 16·50)	99·70	3·17
	≥60Y	--	--	--	--
8	0–4Y	--	--	--	--
	5–17Y	8·68 (--)	8·68 (--)	--	8·68

Month	Age group	Attack rate by fixed effect % (95% CI)	Attack rate by Random effect % (95% CI)	I^2 (%)	Attack rate (%)
	18–60Y	3·83 (3·39, 4·31)	3·76 (2·02, 6·88)	92·10	3·76
	≥60Y	--	--	--	--
9	0–4Y	9·03 (7·77, 10·46)	9·86 (5·69, 16·53)	92·10	9·86
	5–17Y	3·25 (3·00, 3·52)	3·55 (2·29, 5·47)	98·00	3·55
	18–60Y	1·64 (1·57, 1·71)	2·16 (1·18, 3·91)	99·40	2·16
	≥60Y	8·97 (--)	8·97 (--)	--	8·97
10	0–4Y	6·13 (5·55, 6·76)	8·75 (5·44, 13·79)	94·40	8·75
	5–17Y	3·24 (3·02, 3·46)	3·62 (2·15, 6·03)	97·80	3·62
	18–60Y	0·73 (0·61, 0·86)	0·72 (0·50, 1·05)	89·40	0·72
	≥60Y	--	--	--	--
11	0–4Y	6·30 (5·68, 7·00)	8·86 (5·51, 13·95)	93·90	8·86
	5–17Y	3·98 (3·82, 4·14)	4·35 (3·15, 5·98)	97·00	4·35
	18–60Y	1·19 (1·13, 1·24)	1·35 (0·71, 2·53)	99·30	1·35
	≥60Y	2·27 (1·36, 3·17)	11·01 (0·00, 29·97)	91·20	11·01
12	0–4Y	10·17 (9·07, 11·38)	11·76 (5·57, 23·13)	96·40	11·76
	5–17Y	4·76 (4·54, 4·98)	6·65 (3·94, 10·99)	98·40	6·65
	18–60Y	0·95 (0·87, 1·03)	3·39 (1·41, 7·94)	98·50	3·39
	≥60Y	13·21 (--)	13·21 (--)	--	13·21

Appendix Table S10: Attack rate with 95% CI of norovirus outbreaks for each month in in settings of figure 3D

Month	setting	Attack rate by fixed effect % (95% CI)	Attack rate by Random effect % (95% CI)	I^2 (%)	Attack rate (%)
1	nursery	5·38 (4·50, 6·42)	5·96 (3·13, 11·06)	90·60	5·96
	primary school	3·42 (3·21, 3·66)	3·69 (1·98, 6·79)	97·60	3·69
	secondary school	3·77 (3·56, 3·99)	3·30 (2·16, 5·02)	97·70	3·30
	university	1·08 (0·99, 1·16)	3·57 (0·77, 15·00)	99·70	3·57
	restaurant	0·13 (0·11, 0·16)	0·23 (0·04, 1·30)	99·60	0·23
	community	1·33 (1·05, 1·69)	2·57 (0·75, 8·41)	96·90	2·57
	workplace	11·42 (10·46, 12·46)	13·30 (6·12, 26·52)	98·60	13·30
	hospital	17·5 (14·50, 20·51)	17·50 (14·50, 20·51)	43·00	17·50
	nursing home	3·16 (2·09, 4·23)	8·77 (1·52, 16·02)	91·60	8·77
	others	1·36 (--)	1·36 (--)	--	1·36
2	nursery	8·78 (7·97, 9·67)	9·57 (6·04, 14·83)	93·40	9·57
	primary school	4·15 (3·91, 4·40)	3·90 (2·93, 5·19)	95·40	3·90
	secondary school	4·57 (4·39, 4·76)	4·45 (2·65, 7·36)	98·70	4·45
	university	0·56 (0·51, 0·61)	0·51 (0·30, 0·86)	97·70	0·51
	restaurant	6·23 (4·96, 7·80)	12·58 (2·86, 41·30)	97·60	12·58
	community	0·95 (0·88, 1·02)	6·35 (2·51, 15·18)	99·20	6·35
	workplace	79·41 (--)	79·41 (--)	--	79·41
	hospital	9·64 (7·11, 12·17)	9·43 (5·53, 13·33)	56·70	9·43
	nursing home	26·50 (21·86, 31·14)	26·50 (21·86, 31·14)	0·00	26·50
	others	3·45 (3·27, 3·64)	6·24 (1·36, 24·26)	98·60	6·24
3	nursery	4·86 (4·45, 5·30)	6·57 (3·93, 10·77)	97·50	6·57
	primary school	4·97 (4·75, 5·19)	6·15 (3·95, 9·44)	98·10	6·15
	secondary school	5·48 (5·12, 5·86)	6·67 (4·76, 9·28)	95·00	6·67
	nurseries	1·41 (1·36, 1·46)	3·68 (1·58, 8·33)	99·70	3·68
	restaurant	10·84 (7·24, 15·91)	12·90 (0·70, 75·79)	97·50	12·90
	community	2·59 (2·22, 3·03)	2·47 (1·02, 5·87)	98·10	2·47
	workplace	13·29 (12·04, 14·66)	12·80 (8·75, 18·35)	92·10	12·80
	hospital	12·56 (9·04, 16·07)	12·56 (9·04, 16·08)	0·00	12·56
	nursing home	16·05 (--)	16·05 (--)	--	16·05

Month	setting	Attack rate by fixed effect % (95% CI)	Attack rate by Random effect % (95% CI)	I^2 (%)	Attack rate (%)
	others	5·65 (5·22, 6·11)	7·78 (4·92, 12·10)	93·90	7·78
4	nursery	4·85 (4·20, 5·60)	6·74 (3·79, 11·71)	92·30	6·74
	primary school	4·04 (3·79, 4·31)	7·00 (3·74, 12·72)	98·30	7·00
	secondary school	4·90 (4·68, 5·13)	4·54 (2·83, 7·20)	98·80	4·54
	university	2·09 (1·80, 2·42)	2·60 (1·16, 5·73)	98·30	2·60
	restaurant	12·79 (11·19, 14·59)	20·09 (12·32, 31·01)	94·40	20·09
	community	1·90 (--)	1·90 (--)	--	1·90
	workplace	15·61 (13·61, 17·84)	20·01 (8·36, 40·71)	96·80	20·01
	hospital	11·56 (--)	11·56 (--)	--	11·56
	nursing home	--	--	--	--
	others	9·96 (9·29, 10·67)	13·47 (7·51, 22·98)	98·00	13·47
5	nursery	7·13 (6·45, 7·87)	12·71 (7·66, 20·37)	94·50	12·71
	primary school	5·13 (4·84, 5·45)	11·10 (7·22, 16·69)	97·20	11·10
	secondary school	1·83 (1·61, 2·09)	2·06 (1·14, 3·70)	96·20	2·06
	university	0·94 (0·79, 1·12)	0·75 (0·08, 6·80)	99·30	0·75
	restaurant	--	--	--	--
	community	40·54 (--)	40·54 (--)	--	40·54
	workplace	4·83 (--)	4·83 (--)	--	4·83
	hospital	--	--	--	--
	nursing home	--	--	--	--
	others	2·77 (2·42, 3·17)	3·20 (1·19, 8·31)	98·70	3·20
6	nursery	13·07 (11·09, 15·33)	17·03 (7·54, 34·06)	94·00	17·03
	primary school	4·75 (4·32, 5·21)	9·14 (4·84, 16·60)	95·70	9·14
	secondary school	4·48 (3·98, 5·04)	5·84 (2·42, 13·40)	96·80	5·84
	university	7·91 (7·59, 8·24)	7·48 (3·32, 15·99)	99·80	7·48
	restaurant	5·39 (--)	5·39 (--)	--	5·39
	community	9·32 (--)	9·32 (--)	--	9·32
	workplace	12·46 (9·24, 16·60)	10·34 (5·02, 20·12)	80·30	10·34
	hospital	14·49 (--)	14·49 (--)	--	14·49
	nursing home	--	--	--	--
	others	12·28 (10·26, 14·65)	14·00 (7·85, 23·73)	94·70	14·00

Month	setting	Attack rate by fixed effect % (95% CI)	Attack rate by Random effect % (95% CI)	I^2 (%)	Attack rate (%)
7	nursery	2·79 (--)	2·79 (--)	--	2·79
	primary school	1·88 (--)	1·88 (--)	--	1·88
	secondary school	1·08 (--)	1·08 (--)	--	1·08
	university	0·89 (--)	0·89 (--)	--	0·89
	restaurant	12·11 (9·33, 15·59)	12·11 (9·33, 15·59)	0·00	12·11
	community	1·17 (1·07, 1·27)	2·08 (0·78, 5·43)	99·20	2·08
	workplace	--	--	--	--
	hospital	11·10 (10·14, 12·06)	17·84 (11·27, 24·40)	84·30	17·84
	nursing home	--	--	--	--
	others	--	--	--	--
8	nursery	--	--	--	--
	primary school	8·68 (--)	8·68 (--)	--	8·68
	secondary school	--	--	--	--
	university	5·07 (4·54, 5·65)	6·34 (3·47, 11·31)	98·00	6·34
	restaurant	2·23 (--)	2·23 (--)	--	2·23
	community	1·82 (--)	1·82 (--)	--	1·82
	workplace	7·14 (--)	7·14 (--)	--	7·14
	hospital	--	--	--	--
	nursing home	--	--	--	--
	others	2·80 (2·45, 3·19)	3·33 (1·48, 7·30)	98·60	3·33
9	nursery	7·48 (6·48, 8·61)	8·63 (4·96, 14·60)	93·20	8·63
	primary school	3·14 (2·79, 3·53)	3·18 (2·53, 4·00)	75·20	3·18
	secondary school	2·68 (2·46, 2·92)	3·11 (1·60, 5·95)	98·90	3·11
	university	0·90 (0·83, 0·96)	1·38 (0·67, 2·80)	99·00	1·38
	restaurant	26·07 (--)	26·07 (--)	--	26·07
	community	--	--	--	--
	workplace	3·00 (2·85, 3·17)	3·03 (2·11, 4·32)	98·20	3·03
	hospital	--	--	--	--
	nursing home	8·97 (--)	8·97 (--)	--	8·97
	others	6·56 (5·75, 7·46)	7·92 (4·24, 14·30)	97·60	7·92
10	nursery	6·69 (6·16, 7·27)	8·91 (5·94, 13·15)	94·20	8·91

Month	setting	Attack rate by fixed effect % (95% CI)	Attack rate by Random effect % (95% CI)	I^2 (%)	Attack rate (%)
	primary school	3·13 (2·89, 3·40)	2·68 (1·44, 4·96)	98·10	2·68
	secondary school	14·47 (13·81, 15·16)	6·66 (2·63, 15·89)	99·60	6·66
	university	0·73 (0·61, 0·86)	0·72 (0·50, 1·05)	89·40	0·72
	restaurant	--	--	--	--
	community	1·79 (1·62, 1·98)	7·28 (2·28, 20·90)	99·40	7·28
	workplace	--	--	--	--
	hospital	--	--	--	--
	nursing home	--	--	--	--
	others	0·69 (0·63, 0·75)	1·68 (0·70, 3·97)	98·90	1·68
11	nursery	7·84 (7·18, 8·54)	9·45 (6·06, 14·44)	95·10	9·45
	primary school	4·87 (4·65, 5·10)	5·23 (3·34, 8·11)	97·60	5·23
	secondary school	2·19 (2·01, 2·38)	2·17 (1·56, 3·00)	95·00	2·17
	university	1·19 (1·13, 1·25)	0·95 (0·51, 1·76)	99·50	0·95
	restaurant	9·15 (6·35, 13·02)	11·48 (4·78, 25·10)	90·20	11·48
	community	7·05 (6·34, 7·84)	9·27 (3·88, 20·56)	98·80	9·27
	workplace	6·77 (5·42, 8·43)	7·83 (4·68, 12·79)	82·00	7·83
	hospital	6·53 (4·45, 8·61)	7·67 (2·89, 12·45)	54·60	7·67
	nursing home	2·87 (2·21, 3·54)	4·50 (1·24, 7·76)	86·10	4·5
	others	4·10 (3·84, 4·37)	4·85 (2·24, 10·20)	98·60	4·85
12	nursery	9·61 (8·83, 10·45)	10·15 (6·26, 16·03)	96·00	10·15
	primary school	5·06 (4·80, 5·34)	7·23 (4·04, 12·60)	98·30	7·23
	secondary school	5·32 (5·00, 5·67)	5·03 (2·32, 10·57)	98·60	5·03
	university	1·39 (1·26, 1·53)	1·45 (1·12, 1·88)	88·60	1·45
	restaurant	15·05 (--)	15·05 (--)	--	15·05
	community	17·14 (--)	17·14 (--)	--	17·14
	workplace	0·25 (--)	0·25 (--)	--	0·25
	hospital	6·02 (4·77, 7·27)	11·53 (6·60, 16·46)	90·60	11·53
	nursing home	13·87 (10·39, 17·35)	13·87 (10·39, 17·35)	0·00	13·87
	others	4·50 (4·01, 5·05)	7·73 (3·02, 18·40)	97·90	7·73

Appendix Table S11: Attack rate with 95% CI of norovirus outbreaks for each genotype in North China and South China of figure 4A

Genotype	Area	Attack rate by fixed effect % (95% CI)	Attack rate by Random effect % (95% CI)	I^2 (%)	Attack rate (%)
GI.1	North	4·14 (--)	4·14 (--)	--	4·14
	South	4·17 (--)	4·17 (--)	--	4·17
GI.6	North	2·13 (1·63, 2·78)	2·57 (0·36, 16·21)	98·70	2·57
	South	12·07 (10·96, 13·26)	15·28 (5·76, 34·74)	99·50	15·28
GII.2	North	5·25 (4·53, 6·09)	15·94 (4·37, 44·04)	98·30	15·94
	South	2·73 (2·49, 2·99)	4·37 (1·85, 9·97)	98·50	4·37
GII.3	North	--	--	--	--
	South	7·24 (6·46, 8·11)	9·79 (2·33, 33·02)	99·50	9·79
GII.4	North	25·71 (--)	25·71 (--)	--	25·71
	South	0·94 (0·90, 0·98)	2·66 (1·65, 4·26)	99·00	2·66
GII.12	North	43·52 (--)	43·52 (--)	--	43·52
	South	17·85 (--)	17·85 (--)	--	17·85
GII.17	North	2·23 (2·11, 2·37)	6·03 (1·29, 23·94)	99·50	6·03
	South	3·30 (3·10, 3·51)	3·95 (2·18, 7·06)	98·50	3·95
Recombination	North	3·70 (3·18, 4·31)	3·71 (3·14, 4·39)	26·80	3·71
	South	1·31 (1·22, 1·40)	4·83 (2·33, 9·75)	98·40	4·83
Others	North	7·52 (6·74, 8·39)	8·94 (6·41, 12·34)	91·20	8·94
	South	0·83 (0·73, 0·95)	4·38 (0·88, 19·05)	99·00	4·38

Appendix Table S12: Proportions with 95% CI of clinical presentations of norovirus infection in different age groups of appendix figure S4A

Age groups	Presentation	Proportion by fixed effect % (95% CI)	Proportion by Random effect % (95% CI)	I^2 (%)	Proportion (%)
All	Vomiting	63·49 (62·89, 64·08)	78·01 (75·06, 80·84)	97·00	78·01
	Diarrhea	68·14 (67·56, 68·72)	59·94 (55·76, 64·06)	97·90	59·94
	Abdominal pain	44·47 (43·79, 45·14)	51·58 (48·31, 54·85)	96·40	51·58
	Nausea	53·62 (52·84, 54·40)	60·83 (56·59, 64·99)	95·90	60·83
	Abdominal distension	30·98 (29·87, 32·08)	38·31 (31·13, 45·49)	97·70	38·31
	Fever	26·52 (25·96, 27·09)	22·57 (20·28, 25·05)	92·00	22·57
	Headache	44·83 (43·31, 46·42)	23·33 (19·34, 28·14)	95·30	23·33
	Dizziness	31·98 (30·62, 33·36)	35·59 (28·40, 43·13)	95·20	35·59
0–4Y	Vomiting	87·08 (85·42, 88·67)	92·97 (89·09, 96·19)	88·00	92·97
	Diarrhea	60·89 (58·57, 63·19)	38·96 (28·32, 50·12)	97·80	38·96
	Abdominal pain	44·13 (41·13, 47·14)	50·41 (41·02, 59·79)	90·60	50·41
	Nausea	44·27 (39·44, 49·16)	50·29 (31·09, 69·45)	93·20	50·29
	Abdominal distension	14·20 (10·76, 17·65)	30·64 (6·98, 54·30)	96·00	30·64
	Fever	34·64 (32·54, 36·80)	29·28 (23·32, 36·06)	82·40	29·28
	Headache	22·01 (12·80, 37·86)	22·01 (12·80, 37·86)	21·60	22·01
	Dizziness	8·93 (1·69, 21·05)	8·93 (1·69, 21·05)	0·00	8·93
5–17Y	Vomiting	74·90 (74·12, 75·67)	82·28 (78·58, 85·72)	96·20	82·28
	Diarrhea	66·20 (65·34, 67·04)	52·68 (46·98, 58·35)	98·10	52·68
	Abdominal pain	42·90 (41·97, 43·83)	50·37 (45·78, 54·96)	97·10	50·37
	Nausea	58·78 (57·64, 59·91)	61·35 (55·46, 67·08)	94·20	61·35
	Abdominal distension	43·09 (40·83, 45·35)	42·77 (30·65, 54·90)	96·00	42·77
	Fever	28·72 (27·93, 29·53)	20·91 (18·06, 24·07)	92·60	20·91
	Headache	51·55 (48·99, 54·23)	26·26 (20·41, 33·78)	91·20	26·26
	Dizziness	41·41 (38·95, 43·90)	40·01 (28·86, 51·72)	94·50	40·01
18–60Y	Vomiting	45·52 (44·60, 46·43)	58·11 (52·99, 63·15)	97·10	58·11
	Diarrhea	70·97 (70·13, 71·81)	80·15 (75·41, 84·52)	98·20	80·15
	Abdominal	46·50 (45·47,	54·81 (49·74, 59·84)	96·30	54·81

Age groups	Presentation	Proportion by fixed effect % (95% CI)	Proportion by Random effect % (95% CI)	I^2 (%)	Proportion (%)
	pain	47·52)			
	Nausea	49·30 (48·21, 50·40)	61·75 (55·56, 67·76)	97·40	61·75
	Abdominal distension	29·01 (27·65, 30·38)	35·43 (25·75, 45·12)	98·20	35·43
	Fever	21·93 (21·10, 22·79)	22·34 (18·03, 27·34)	93·00	22·34
	Headache	39·98 (38·12, 41·93)	21·23 (15·71, 28·69)	97·20	21·23
	Dizziness	27·67 (26·05, 29·33)	34·24 (24·45, 44·76)	95·70	34·24
≥60Y	Vomiting	79·80 (72·37, 86·45)	78·87 (58·71, 94·15)	83·20	78·87
	Diarrhea	74·48 (66·66, 81·69)	74·05 (55·02, 89·75)	79·70	74·05
	Abdominal pain	38·17 (24·77, 52·42)	43·17 (13·68, 75·19)	81·70	43·17
	Nausea	71·36 (55·00, 85·25)	75·76 (39·26, 98·16)	77·30	75·76
	Abdominal distension	68·18 (--)	68·18 (--)	--	68·18
	Fever	27·20 (20·13, 35·65)	22·49 (8·35, 48·03)	79·80	22·49
	Headache	19·71 (8·33, 46·61)	15·35 (3·43, 68·65)	53·10	15·35
	Dizziness	17·25 (5·95, 32·85)	21·33 (0·00, 68·20)	85·50	21·33

Appendix Table S13: Proportions with 95% CI of clinical presentations of norovirus infection in different GI genotypes of appendix figure S4B

Genotype	Presentation	Proportion by fixed effect % (95%CI)	Proportion by Random effect % (95%CI)	I^2 (%)	Proportion (%)
All	Vomiting	78·96 (75·55, 82·19)	83·80 (73·48, 92·15)	83·60	83·80
	Diarrhea	92·17 (90·75, 93·59)	52·22 (33·21, 71·23)	98·70	52·22
	Abdominal pain	55·74 (51·32, 60·16)	51·86 (38·49, 65·23)	84·60	51·86
	Nausea	65·97 (61·35, 70·59)	64·12 (57·79, 70·46)	12·00	65·97
	Fever	26·41 (22·16, 30·87)	25·73 (20·86, 30·90)	39·90	26·41
	Headache	22·81 (17·59, 28·43)	22·81 (17·59, 28·43)	0·00	22·81
	Dizziness	31·76 (26·15, 37·37)	27·18 (13·51, 40·85)	73·10	27·18
GI.1	Vomiting	65·55 (--)	65·55 (--)	--	65·55
	Diarrhea	98·19 (96·67, 99·72)	85·08 (55·55, 100·00)	98·80	85·08
	Abdominal pain	64·11 (--)	64·11 (--)	--	64·11
	Nausea	70·33 (--)	70·33 (--)	--	70·33
	Fever	29·67 (--)	29·67 (--)	--	29·67
	Headache	24·88 (--)	24·88 (--)	--	24·88
	Dizziness	36·36 (--)	36·36 (--)	--	36·36
GI.3	Vomiting	92·61 (85·27, 97·81)	92·61 (85·27, 97·81)	0·00	92·61
	Diarrhea	26·98 (18·13, 35·83)	28·56 (0·00, 66·90)	94·60	28·56
	Abdominal pain	52·50 (42·17, 62·83)	57·79 (20·57, 95·00)	91·50	57·79
	Nausea	57·14 (--)	57·14 (--)	--	57·14
	Fever	26·79 (--)	26·79 (--)	--	26·79
	Headache	--	--	--	--
	Dizziness	--	--	--	--
GI.5	Vomiting	100·00 (--)	100·00 (--)	--	100·00
	Diarrhea	15·79 (--)	15·79 (--)	--	15·79
	Abdominal pain	26·32 (--)	26·32 (--)	--	26·32
	Nausea	--	--	--	--
	Fever	5·26 (--)	5·26 (--)	--	5·26
	Headache	--	--	--	--
	Dizziness	--	--	--	--
GI.6	Vomiting	80·79 (75·28, 85·8)	83·19 (70·89, 92·85)	62·50	83·19

Genotype	Presentation	Proportion by fixed effect % (95%CI)	Proportion by Random effect % (95%CI)	I^2 (%)	Proportion (%)
	Diarrhea	69·47 (63·68, 75·25)	62·10 (46·61, 77·58)	70·10	62·10
	Abdominal pain	53·85 (--)	53·85 (--)	--	53·85
	Nausea	56·41 (--)	56·41 (--)	--	56·41
	Fever	23·97 (12·81, 37·02)	20·27 (3·16, 45·13)	64·40	20·27
	Headache	17·95 (--)	17·95 (--)	--	17·95
	Dizziness	17·95 (--)	17·95 (--)	--	17·95
Recombination GI	Vomiting	80·44 (71·31, 88·34)	72·58 (39·98, 96·17)	80·00	72·58
	Diarrhea	47·28 (39·79, 54·77)	50·44 (1·43, 99·45)	97·70	50·44
	Abdominal pain	48·33 (38·99, 57·66)	50·77 (23·79, 77·75)	87·50	50·77
	Nausea	63·92 (54·23, 73·61)	63·92 (54·23, 73·61)	0·00	63·92
	Fever	26·98 (17·90, 37·09)	26·98 (17·90, 37·09)	0·00	26·98
	Headache	22·22 (--)	22·22 (--)	--	22·22
	Dizziness	22·22 (--)	22·22 (--)	--	22·22

Appendix Table S14: Proportions with 95% CI of clinical presentations of norovirus infection in different GII genotypes of appendix figure S4C

Genotype	Presentation	Proportion by fixed effect % (95% CI)	Proportion by Random effect % (95% CI)	I^2 (%)	Proportion (%)
All	Vomiting	65·13 (63·99, 66·27)	75·11 (69·22, 80·60)	96·90	75·11
	Diarrhea	64·11 (62·94, 65·28)	57·97 (49·74, 65·99)	97·70	57·97
	Abdominal pain	54·91 (53·47, 56·35)	53·81 (47·49, 60·08)	93·50	53·81
	Nausea	88·61 (87·92, 89·30)	60·06 (52·76, 67·35)	99·10	60·06
	Abdominal distension	32·31 (30·20, 34·43)	38·82 (27·90, 49·75)	94·00	38·82
	Fever	30·51 (29·40, 31·64)	24·37 (19·81, 29·60)	90·30	24·37
	Headache	38·80 (36·69, 40·93)	21·46 (9·65, 36·12)	98·10	21·46
	Dizziness	42·70 (38·87, 46·91)	14·78 (8·58, 25·44)	95·00	14·78
GII.2	Vomiting	89·45 (85·91, 92·59)	84·87 (67·74, 96·76)	87·50	84·87
	Diarrhea	41·12 (34·63, 47·75)	46·07 (22·12, 70·93)	90·50	46·07
	Abdominal pain	60·25 (55·17, 65·24)	57·30 (39·54, 74·21)	93·40	57·30
	Nausea	76·66 (72·42, 80·91)	67·00 (52·34, 81·66)	86·80	67·00
	Abdominal distension	--	--	--	--
	Fever	24·67 (20·60, 29·25)	26·96 (16·07, 41·58)	77·80	26·96
	Headache	6·01 (0·70, 14·47)	6·01 (0·70, 14·47)	0·00	6·01
	Dizziness	3·27 (0·85, 12·62)	3·21 (0·48, 21·59)	49·70	3·27
GII.3	Vomiting	96·35 (93·48, 98·53)	96·2 (90·68, 99·57)	57·60	96·20
	Diarrhea	22·02 (17·15, 27·27)	21·35 (11·15, 33·57)	77·80	21·35
	Abdominal pain	47·68 (41·67, 53·73)	57·04 (16·99, 92·42)	97·50	57·04
	Nausea	33·33 (--)	33·33 (--)	--	33·33
	Abdominal distension	--	--	--	--
	Fever	42·18 (36·48, 48·10)	47·55 (23·39, 72·92)	94·30	47·55
	Headache	--	--	--	--
	Dizziness	--	--	--	--
GII.4	Vomiting	57·18 (54·70, 59·64)	63·04 (50·79, 74·53)	97·00	63·04
	Diarrhea	87·66 (85·92, 89·32)	78·40 (65·94, 88·77)	96·00	78·40
	Abdominal pain	56·63 (53·76, 59·48)	51·47 (37·80, 65·04)	93·20	51·47

Genotype	Presentation	Proportion by fixed effect % (95% CI)	Proportion by Random effect % (95% CI)	I^2 (%)	Proportion (%)
	Nausea	93·34 (92·45, 94·22)	56·42 (43·62, 69·23)	99·40	56·42
	Abdominal distension	43·26 (39·44, 47·08)	46·12 (38·24, 54·01)	78·20	38·82
	Fever	27·48 (25·35, 29·71)	23·01 (14·42, 34·64)	90·40	23·01
	Headache	21·40 (17·16, 25·95)	19·82 (5·07, 40·50)	94·60	19·82
	Dizziness	25·24 (21·22, 30·01)	15·77 (8·02, 31·02)	89·40	15·77
GII.12	Vomiting	61·14 (58·68, 63·58)	77·79 (24·57, 100·00)	99·70	77·79
	Diarrhea	73·19 (70·94, 75·39)	49·61 (0·01, 99·95)	99·80	49·61
	Abdominal pain	55·07 (--)	55·07 (--)	--	55·07
	Nausea	--	--	--	--
	Abdominal distension	--	--	--	--
	Fever	42·93 (40·47, 45·43)	36·05 (23·08, 51·45)	97·30	36·05
	Headache	56·23 (--)	56·23 (--)	--	56·23
	Dizziness	--	--	--	--
GII.17	Vomiting	66·02 (63·92, 68·09)	73·63 (66·03, 80·60)	95·90	73·63
	Diarrhea	46·52 (44·33, 48·71)	55·12 (40·38, 69·44)	97·00	55·12
	Abdominal pain	52·24 (49·88, 54·60)	57·64 (47·06, 67·88)	95·70	57·64
	Nausea	84·08 (82·89, 85·27)	62·00 (49·00, 75·00)	99·30	62·00
	Abdominal distension	27·47 (24·93, 30·01)	29·41 (9·08, 49·75)	95·60	29·41
	Fever	22·58 (20·70, 24·58)	18·69 (12·85, 26·37)	88·80	18·69
	Headache	7·11 (4·59, 10·08)	13·99 (0·34, 39·59)	90·40	13·99
	Dizziness	16·78 (13·08, 21·52)	15·55 (5·42, 44·59)	92·10	15·55
Recombination GII	Vomiting	54·4 (51·18, 57·60)	77·01 (60·88, 90·09)	96·50	77·01
	Diarrhea	62·69 (59·52, 65·81)	54·42 (35·99, 72·28)	94·50	54·42
	Abdominal pain	60·12 (56·33, 63·86)	49·78 (33·90, 65·67)	91·70	49·78
	Nausea	82·49 (76·79, 88·18)	82·39 (75·79, 89·00)	8·20	82·49
	Abdominal distension	--	--	--	--
	Fever	27·93 (24·65, 31·46)	20·34 (11·36, 33·71)	88·30	20·34
	Headache	75·00 (--)	75·00 (--)	--	75·00

Genotype	Presentation	Proportion by fixed effect % (95% CI)	Proportion by Random effect % (95% CI)	I^2 (%)	Proportion (%)
	Dizziness	72·99 (64·37, 82·76)	22·46 (4·81, 100·00)	85·10	22·46
GII.other	Vomiting	79·02 (73·96, 83·70)	84·80 (65·58, 97·46)	94·40	84·80
	Diarrhea	42·35 (35·55, 49·29)	37·00 (20·20, 55·47)	85·30	37·00
	Abdominal pain	49·35 (42·45, 56·27)	50·37 (32·53, 68·16)	79·90	50·37
	Nausea	42·27 (36·13, 48·40)	46·14 (17·17, 75·10)	97·00	46·14
	Abdominal distension	--	--	--	--
	Fever	33·59 (28·13, 39·52)	33·45 (26·96, 40·63)	40·40	33·59
	Headache	5·00 (--)	5·00 (--)	--	5·00
	Dizziness	--	--	--	--

Appendix Table S15: Reference number for analyzed risk factors of attack rate with Beta-Binomial Model

<table>
<tr><th>Risk factors</th><th>Reference ID</th></tr>
<tr><td>Age groups</td><td rowspan="5">10, 11, 14, 17, 18, 21, 25, 26, 32, 41, 44, 50, 54, 55, 58, 59, 62, 63, 69, 73, 78, 79, 80, 96, 102, 103, 104, 108, 109, 125, 127, 129, 148, 161, 162, 171, 172, 174, 176, 177, 182, 183, 186, 192, 194, 195, 196, 197, 205, 206, 222, 224, 231, 232, 233, 241, 242, 245, 252, 253, 254, 257, 260, 261, 265, 284, 285, 291, 294, 303, 307, 308, 315, 316, 318, 320, 329, 340, 344, 346, 348, 349, 352, 359, 366, 374, 376, 380, 381, 388, 393, 396, 403, 404, 410, 411, 413, 415, 420, 421, 422, 433, 436, 441, 456, 458, 462, 472, 484, 495, 507, 510, 511, 512, 515, 521, 522, 524, 525, 532, 534, 548, 554, 555, 579, 589, 597, 600, 604, 616, 625, 631, 645, 652, 657, 659, 665, 668, 671, 678, 688, 689, 706, 708, 718, 719, 727, 729, 730, 732, 734, 735, 736, 737, 738, 741, 742, 743, 744, 745, 758, 767, 772, 774, 795, 796, 808, 819, 820, 822, 824, 845, 859, 860, 861, 862, 867, 868, 869, 875, 883, 885, 891, 903, 904, 906, 907, 908, 909, 918, 919, 929, 936, 946, 950, 952, 954, 956, 958, 964, 968, 987, 995, 1000, 1001, 1007, 1015, 1025, 1029, 1039, 1055, 1071, 1080, 1088, 1097, 1103</td></tr>
<tr><td>Routes of transmission</td></tr>
<tr><td>Settings</td></tr>
<tr><td>Seasons</td></tr>
<tr><td>Areas</td></tr>
</table>

Appendix Table S16: Comparative analysis of the attack rate by the Beta-Binomial Model

	N	Univariate analysis		Multivariable analysis	
		RR# (95%CI)	*p*	RR# (95%CI)	*p*
**Age groups(year)**					
Older adults (≥60)	2	1	-	1	-
Children (0–4)	64	0.32 (0.11, 0.95)	0.039*	0.13 (0.03, 0.63)	0.012*
Adolescents (5–17)	162	0.19 (0.07, 0.55)	0.002*	0.09 (0.02, 0.35)	<0.001*
Adults (18–60)	74	0.16 (0.06, 0.48)	0.001*	0.18 (0.05, 0.65)	0.009*
**Settings**					
Universities	30	1	-	1	-
Nurseries	67	3.33 (2.24, 4.96)	<0.001*	4.43 (1.57, 12.55)	0.005*
Primary schools	98	2.27 (1.55, 3.34)	<0.001*	4.01 (1.98, 8.12)	<0.001*
Secondary schools	49	1.62 (1.06, 2.48)	0.026*	3.15 (1.55, 6.40)	0.002*
Restaurants	7	4.81 (2.46, 9.42)	<0.001*	4.47 (2.28, 8.79)	<0.001*
Communities	6	1.83 (0.83, 4.04)	0.132	2.18 (0.96, 4.92)	0.061
Workplaces	21	3.01 (1.84, 4.92)	<0.001*	2.91 (1.81, 4.67)	<0.001*
Nursing homes	2	2.55 (0.75, 8.70)	0.134	1.57 (0.40, 6.21)	0.521
Hospitals	4	2.44 (0.99, 6.01)	0.053	2.37 (0.99, 5.66)	0.051
Others	18	2.25 (1.33, 3.80)	0.002*	3.93 (2.05, 7.51)	<0.001*
**Seasons**					
Cold	195	1	-	No significant and excluded	
Warm	107	1.15 (0.93, 1.42)	0.206		
**Areas**					
South	188	1	-	1	-
North	114	2.04 (1.67, 2.50)	<0.001*	1.97 (1.61, 2.41)	<0.001*
**Routes of transmission**					
Human-to-human	176	1	-	1	-
Foodborne	81	0.71 (0.56, 0.91)	0.006*	1.05 (0.78, 1.41)	0.745
Waterborne	45	0.73 (0.55, 0.99)	0.041*	0.99 (0.69, 1.42)	0.962

Relative Risk; * Significant at $p < 0.05$; The number of articles included in the Beta-Binomial Model is 216 (Appendix1 p29). the beta-Binomial Model was used to analyze the risk factors for the attack rate, including age group, setting, season, region and transmission route.

Appendix Figure S1: Temporal distribution of recombinant norovirus genotypes.

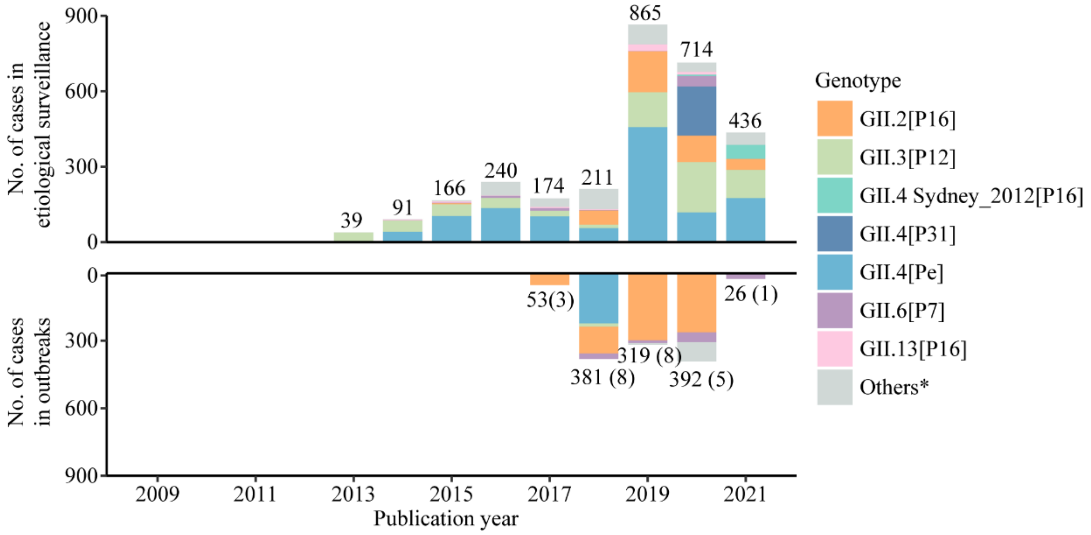

The abscissa is the year of study, the upper ordinate is the number of detected recombinant noroviruses in the surveillance study, and the lower ordinate is the number of cases caused by outbreaks of recombinant norovirus, and the number of outbreaks is in parentheses. *Others indicate GI.3[P13], GI.3[Pd], GI.5[P2], GI.5[P4], GI.6[P11], GI.6[Pb], GII.1[Pg], GII.2[P12],GII.2[Pe], GII.3[P4], GII.3[P16], GII.3[P21], GII.3[P25], GII.3[Pe], GII.4Sydney.2012[P4], GII.4New.Orleans[P4], GII.4New.Orleans[P4_Sydney.2012], GII.4[P12], GII.4[P16], GII.4_2006b[Pa], GII.5[P22], GII.9[P7], GII.9[Pe], GII.12[Pg], GII.13[P21], GII.14[P7], GII.17[P12], GII.17[Pe].

Appendix Figure S2: Distribution of norovirus outbreaks in seven ecoclimatic regions, China. (A) number and attack rate, (B) Genotype.

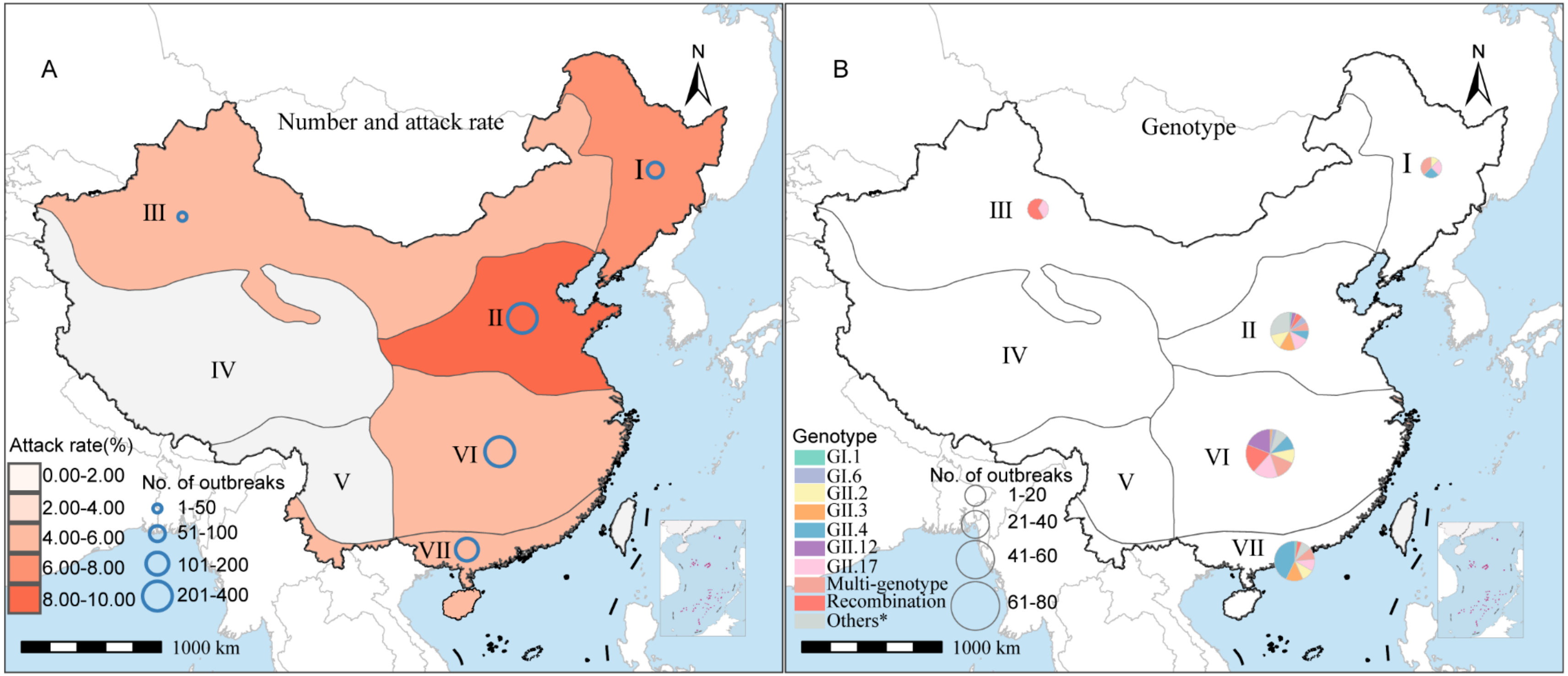

I=Northeast region (NE), II=North China region (N), III=Inner Mongolia-Xinjiang region (IMX), IV=Qinghai-Tibet region (QT), V=Southwest region (SW), VI=Central China region (C) and VII=South China region (S). Panel A represent the number and attack rate of norovirus outbreaks in different climatic regions and panel B represent the distribution of genotypes in the norovirus outbreak in different climatic regions. No norovirus outbreaks were reported in Qinghai-Tibet region and Southwest region. * includes GI.2, GI.3, GI.5, GII.6, GII.7, GII.8, GII.14, GII.21. The base layer of the map is publically available on the Resource and Environmental Sciences and Data Centre (https://www.resdc.cn/DOI/DOI.aspx?DOIID=122, accessed on 15 February 2023).

Appendix Figure S3: The number of norovirus outbreaks of transmission routes in different settings in China (A), and the proportion of norovirus outbreaks of transmission routes in different settings in China (B).

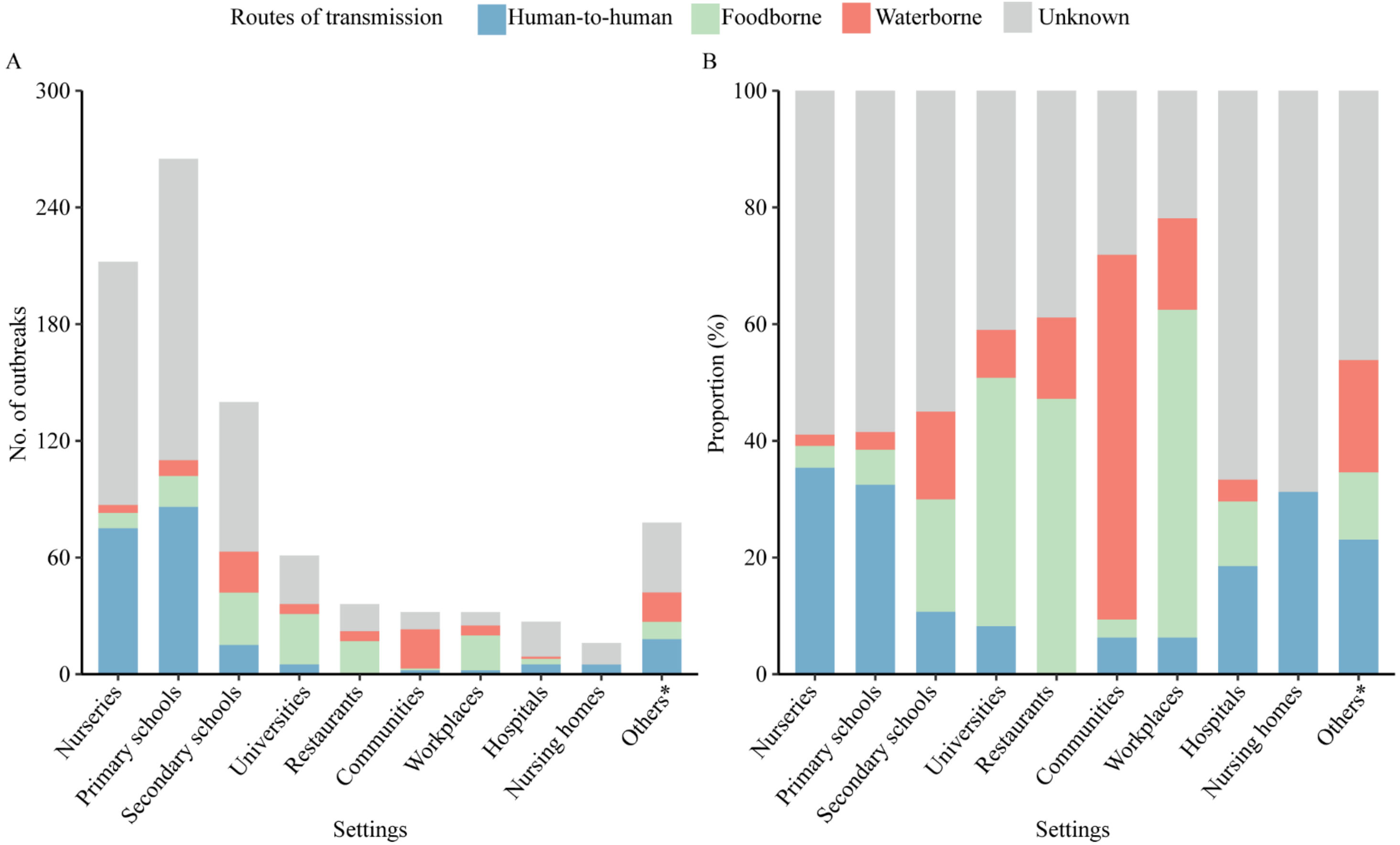

(A) The horizontal axis represents the norovirus outbreak settings, and the vertical axis represents the number of outbreaks in different transmission routes; (B) The horizontal axis represents the norovirus outbreak settings and the vertical axis represents the proportion of different transmission routes. * indicates households, transportations, welfares, military settings, prisons, multiple setting types and multiple school types.

Appendix Figure S4: Clinical manifestations in patients infected with norovirus by age (A), GI genogroup (B) and GII genogroup (C).

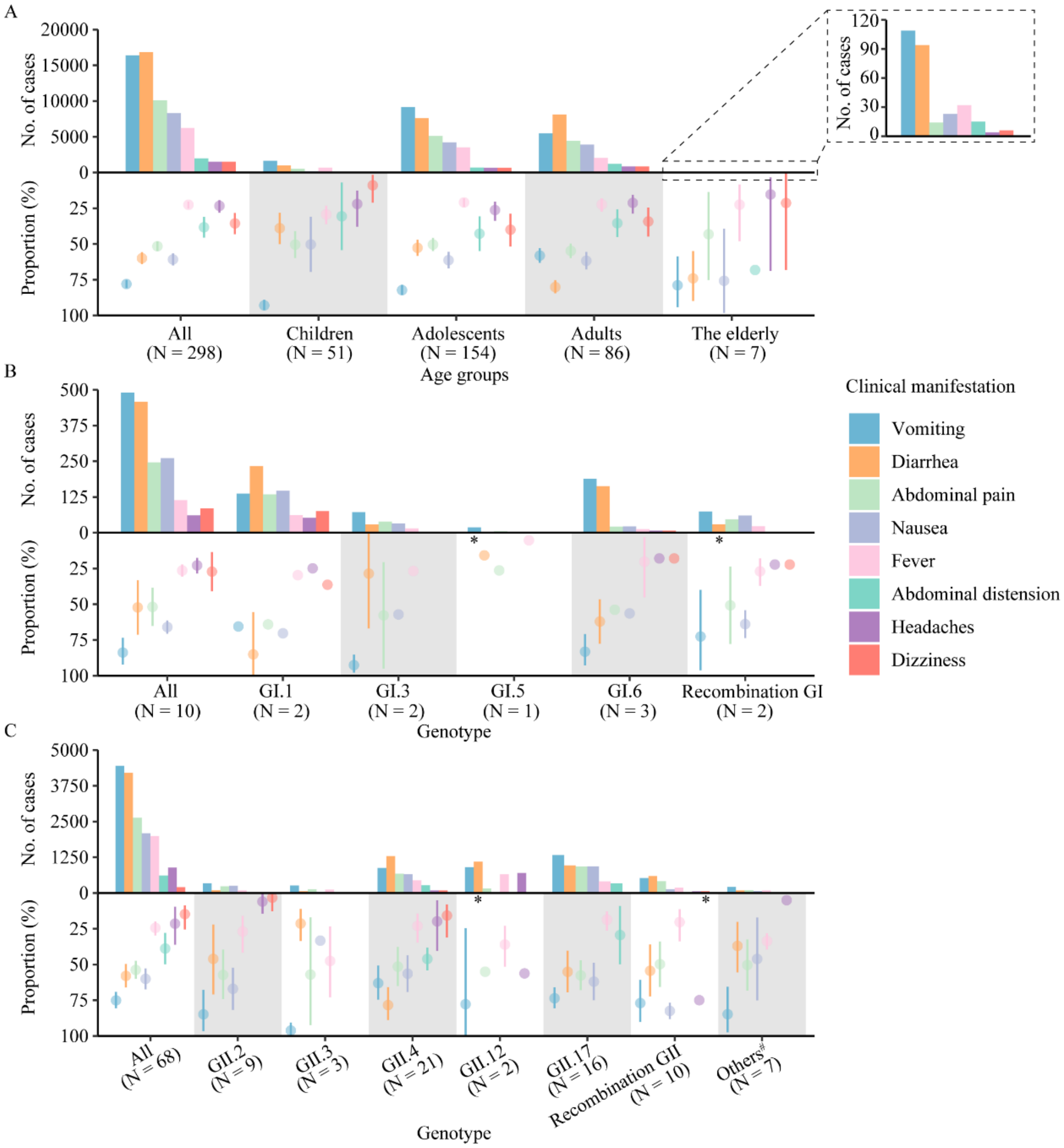

Number and mean proportion of patients with the clinical manifestation and 95% CI based on the meta-analysis. Numbers in parentheses indicate the number of articles included in this analysis. Clinical manifestations for patients infected with norovirus. * indicates either a quite high mean proportion of patients with the clinical manifestation or a wide 95% CI, which was presented for vomiting in GI.5 (100.00%) and for diarrhea in recombination GI [50.44% (95% CI: 1.43, 99.45)] on panel B. The number in parentheses indicates the number of articles included in this analysis. * indicates either a quite high mean proportion of patients with the clinical manifestation or a wide 95% CI, which was presented for diarrhea in GII.12 (49.61% [95% CI: 0.01, 99.95]) and for dizziness in Recombination GII (22.46% [95% CI: 4.81, 100.00]) on panel C. # includes GII.6, GII.7, and GII.8.
