# Supplementary material for: Prevalence and Etiological Characteristics of Norovirus Infection in China: A Systematic Review and Meta-Analysis
Source: Viruses. 2023 Jun 7;15(6):1336. doi: 10.3390/v15061336 (PMC10302178; doi:10.3390/v15061336)
Supplement: Supplementary file 1 [file viruses-15-01336-s001.zip › Appendix S3.pdf]

Supplementary Appendix S3

Supplement to: Prevalence and etiological characteristics of norovirus infection in China: a systematic review and meta-analysis

Appendix Figure S5: Forest plots for meta-analysis

(A) Results of meta-analysis of Table 1:

(a1) total positive rate in China, (a2) total attack rate in China, (a3) positive rate in North and South China, (a4) attack rate in North and South China.

(B) Results of meta-analysis of figure 3:

(b1) annual outbreak attack rate in North China, (b2) annual outbreak attack rate in South China, (b3) monthly outbreak attack rate in North China, (b4) monthly outbreak attack rate in South China, (b5) monthly outbreak attack rates in 0 to 4 years old, (b6) monthly outbreak attack rates in 5 to 17 years old, (b7) monthly outbreak attack rates in 18 to 60 years old, (b8) monthly outbreak attack rates in ≥60 years old, (b9) monthly outbreak attack rates in nurseries, (b10) monthly outbreak attack rates in primary schools, (b11) monthly outbreak attack rates in secondary schools, (b12) monthly outbreak attack rates in universities, (b13) monthly outbreak attack rates in restaurants, (b14) monthly outbreak attack rates in communities, (b15) monthly outbreak attack rates in workplaces, (b16) monthly outbreak attack rates in hospitals, (b17) monthly outbreak attack rates in nursing homes, (b18) monthly outbreak attack rates in other settings.

(C) Results of meta-analysis of appendix figure s2:

(c1) outbreak attack rates in ecological regions.

(D) Results of meta-analysis of figure 4:

(d1) attack rates of outbreaks by genotypes in North China, (d2) attack rates of outbreaks by genotypes in South China.

(E) Results of meta-analysis of figure 5:

(e1) vomiting in different age groups, (e2) diarrhea in different age groups, (e3) abdominal pain in different age groups, (e4) nausea in different age groups, (e5) abdominal distension in different age groups, (e6) fever in different age groups, (e7) headache in different age groups, (e8) dizziness in different age groups, (e9) vomiting in different GII genotypes, (e10) diarrhea in different GII genotypes, (e11) abdominal pain in different GII genotypes, (e12) nausea in different GII genotypes, (e13) abdominal distension in different GII genotypes, (e14) fever in different GII genotypes, (e15) headache in different GII genotypes, (e16) dizziness in different GII genotypes;

(F) Results of meta-analysis of appendix figure s4:

(f1) vomiting in different GI genotypes, (f2) diarrhea in different GI genotypes, (f3) abdominal pain in different GI genotypes, (f4) nausea in different GI genotypes, (f5) fever in different GI genotypes, (f6) headache in different GI genotypes, (f7) dizziness in different GI genotypes.

(A)

(a1)

Study Total	Events	Total	Positive rate	95%−CI
AnShuYi 2013[1]	10	155	0.06	[0.03; 0.12]
Xiao−Wei An 2021[2]	5	82	0.06	[0.02; 0.14]
BaiJing 2018[4]	67	353	0.19	[0.15; 0.23]
BaiXue 2020[5]	58	1240	0.05	[0.04; 0.06]
BaiYongFeng 2014[7]	34	654	0.05	[0.04; 0.07]
BaoLin 2019[9]	58	300	0.19	[0.15; 0.24]
BiWenJun 2020[12]	115	1041	0.11	[0.09; 0.13]
BiWenJun 2019[13]	76	768	0.10	[0.08; 0.12]
CaiMiaoSen 2016[15]	27	1319	0.02	[0.01; 0.03]
CaiMin 2019[16]	65	658	0.10	[0.08; 0.12]
KongFanMing 2019[19]	73	1100	0.07	[0.05; 0.08]
CaiTe 2015[22]	27	300	0.09	[0.06; 0.13]
CaiYing 2017[28]	113	2129	0.05	[0.04; 0.06]
CaoJian 2017[29]	106	465	0.23	[0.19; 0.27]
CaoRanRan 2015[31]	107	428	0.25	[0.21; 0.29]
CaoYiHui 2021[34−1]	43	308	0.14	[0.10; 0.18]
CaoYiHui 2021[34−2]	73	376	0.19	[0.16; 0.24]
CaoYiHui 2021[35−1]	96	709	0.14	[0.11; 0.16]
CaoYiHui 2021[35−2]	24	869	0.03	[0.02; 0.04]
CaoYiHui 2017[36−1]	42	443	0.09	[0.07; 0.13]
CaoYiHui 2017[36−2]	167	923	0.18	[0.16; 0.21]
CaoYiHui 2017[36−3]	119	1149	0.10	[0.09; 0.12]
CaoYiHui 2017[36−4]	193	1708	0.11	[0.10; 0.13]
CaoYongNing 2020[38]	737	2135	0.35	[0.33; 0.37]
ZengDeXing 2015[40]	25	150	0.17	[0.11; 0.24]
ZengHuaShu 2015[42]	19	120	0.16	[0.10; 0.24]
ZengJunRong 2013[43]	38	218	0.17	[0.13; 0.23]
ChangHaiLing 2016[48]	98	881	0.11	[0.09; 0.13]
ChangXueHong 2017[49]	88	1061	0.08	[0.07; 0.10]
ShenMeiYun 2016[51]	24	231	0.10	[0.07; 0.15]
ShenYueHua 2016[56]	149	797	0.19	[0.16; 0.22]
ChenAQun 2017[57]	428	2274	0.19	[0.17; 0.20]
ChenFengQin 2013[65]	24	156	0.15	[0.10; 0.22]
ChenFengQin 2012[66]	61	459	0.13	[0.10; 0.17]
ChenGaoShang 2020[67]	80	1363	0.06	[0.05; 0.07]
ChenGaoShang 2018[68]	84	1154	0.07	[0.06; 0.09]
ChenGuoPing 2018[70]	234	1415	0.17	[0.15; 0.19]
ChenHaiLi 2020[71]	162	912	0.18	[0.15; 0.20]
ChenHaoChuan 2019[72]	63	411	0.15	[0.12; 0.19]
ChenHuiFang 2014[75]	77	709	0.11	[0.09; 0.13]
ChenHuiZhong 2019[76]	136	1365	0.10	[0.08; 0.12]
ChenHuiZhong 2018[77]	98	1007	0.10	[0.08; 0.12]
ChenJiang 2019[81]	655	6753	0.10	[0.09; 0.10]
ChenJingHong 2015[82]	70	825	0.08	[0.07; 0.11]
ChenJing 2020[83]	25	372	0.07	[0.04; 0.10]
ChenJun 2020[85]	36	464	0.08	[0.05; 0.11]
ChenLiLi 2018[86]	2253	19930	0.11	[0.11; 0.12]
ChenLiPing 2020[87]	414	1757	0.24	[0.22; 0.26]
ChenLinLin 2018[88]	26	313	0.08	[0.05; 0.12]
ChenLingXia 2016[89]	58	246	0.24	[0.18; 0.29]
ChenLongHui 2015[90]	73	459	0.16	[0.13; 0.20]
ChenMin 2016[91]	49	374	0.13	[0.10; 0.17]

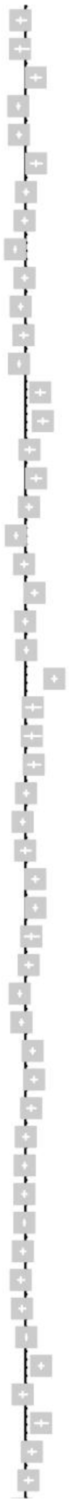

ChenMinMei 2014[93]	24	323	0.07	[0.05; 0.11]
ChenPing 2013[94]	147	811	0.18	[0.16; 0.21]
ChenWeiPing 2018[97]	138	1308	0.11	[0.09; 0.12]
ChenXiKai 2012[98]	60	485	0.12	[0.10; 0.16]
ChenXiaoDong 2020[99]	15	122	0.12	[0.07; 0.19]
ChenYan 2020[105]	94	6986	0.01	[0.01; 0.02]
ChenYan 2016[106]	13	1747	0.01	[0.00; 0.01]
ChenYanWei 2017[107]	224	1450	0.15	[0.14; 0.17]
ChenYu 2016[110]	100	936	0.11	[0.09; 0.13]
Yu−Feng Chen 2021[111]	109	4805	0.02	[0.02; 0.03]
ChenYun 2017[112]	115	408	0.28	[0.24; 0.33]
ChenZhenMing 2012[113]	22	426	0.05	[0.03; 0.08]
ChenZhiHao 2012[114]	81	366	0.22	[0.18; 0.27]
ChengCheng 2019[116]	9	524	0.02	[0.01; 0.03]
ChengSiSi 2018[118]	239	1250	0.19	[0.17; 0.21]
ChengYuan 2017[119]	107	627	0.17	[0.14; 0.20]
CuiDaWei 2016[121]	298	1109	0.27	[0.24; 0.30]
YanYuXiao 2020[122]	392	909	0.43	[0.40; 0.46]
CuiXiaoShuang 2016[126]	137	942	0.15	[0.12; 0.17]
DaiLei 2019[128]	2	504	0.00	[0.00; 0.01]
DengAiPing 2014[130]	1028	4644	0.22	[0.21; 0.23]
DengJianKai 2015[131]	45	290	0.16	[0.12; 0.20]
DengLi 2015[132]	113	1259	0.09	[0.07; 0.11]
Ying Deng 2012[134]	15	450	0.03	[0.02; 0.05]
DiGuangFu 2019[136]	66	10085	0.01	[0.01; 0.01]
DiQianQian 2016[137]	389	2489	0.16	[0.14; 0.17]
DingMing 2016[139]	1314	4214	0.31	[0.30; 0.33]
DingYouFa 2013[140]	17	70	0.24	[0.15; 0.36]
DongHeGui 2017[142]	174	1352	0.13	[0.11; 0.15]
DongHongYan 2015[143]	26	336	0.08	[0.05; 0.11]
DongHongYan 2017[144]	112	926	0.12	[0.10; 0.14]
DongJianHua 2019[145]	16	114	0.14	[0.08; 0.22]
DongZhaoJing 2020[147]	78	753	0.10	[0.08; 0.13]
DuZhenYuan 2014[149]	45	415	0.11	[0.08; 0.14]
DuYao 2013[150]	157	708	0.22	[0.19; 0.25]
DuanJingJing 2018[151]	74	1287	0.06	[0.05; 0.07]
EJingWen 2014[153]	155	869	0.18	[0.15; 0.21]
FanChaoMeng 2019[154]	169	832	0.20	[0.18; 0.23]
FangYuLian 2019[155]	241	758	0.32	[0.28; 0.35]
FangYuLian 2021[156]	809	3116	0.26	[0.24; 0.28]
FeiYi 2013[159]	58	619	0.09	[0.07; 0.12]
FuJianGuang 2011[165]	26	92	0.28	[0.19; 0.39]
FuYun 2015[166]	171	628	0.27	[0.24; 0.31]
FuYun 2019[167]	331	1991	0.17	[0.15; 0.18]
FuLi 2015[168]	271	1904	0.14	[0.13; 0.16]
FuYaLi 2017[169]	110	583	0.19	[0.16; 0.22]
FuYiFei 2013[170]	110	1327	0.08	[0.07; 0.10]
GaoGuiLing 2015[173]	39	617	0.06	[0.05; 0.09]
GaoHongMei 2020[175]	51	3003	0.02	[0.01; 0.02]
GaoLei 2021[178]	337	3801	0.09	[0.08; 0.10]
GaoLu 2019[179]	99	1536	0.06	[0.05; 0.08]
GaoLu 2019[180]	30	455	0.07	[0.04; 0.09]
GaoXiang 2021[184]	33	588	0.06	[0.04; 0.08]
GaoXin 2020[185]	8	160	0.05	[0.02; 0.10]
GeBin 2018[188]	37	180	0.21	[0.15; 0.27]

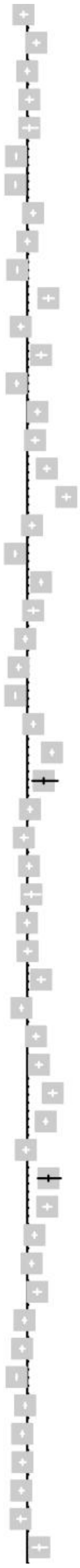

GeHaiXia 2018[189]	293	1735	0.17	[0.15; 0.19]
GengQian 2017[190]	177	1516	0.12	[0.10; 0.13]
GongChunHua 2018[191]	123	617	0.20	[0.17; 0.23]
GongMin 2018[193]	103	462	0.22	[0.19; 0.26]
GuanHongXia 2016[198]	86	915	0.09	[0.08; 0.11]
GuanHongXia 2014[199]	45	291	0.15	[0.12; 0.20]
GuoBaoFu 2016[201]	203	1226	0.17	[0.15; 0.19]
GuoJing 2019[203]	193	781	0.25	[0.22; 0.28]
GuoJuan 2021[204]	60	1036	0.06	[0.04; 0.07]
GuoLiMin 2018[207]	34	463	0.07	[0.05; 0.10]
GuoXinHui 2017[210]	54	18912	0.00	[0.00; 0.00]
HanDongJie 2016[211−1]	112	782	0.14	[0.12; 0.17]
HanDongJie 2016[211−2]	174	4226	0.04	[0.04; 0.05]
HanNing 2016[213]	50	287	0.17	[0.13; 0.22]
HanYiFei 2020[214]	137	822	0.17	[0.14; 0.19]
HaoShiXuan 2017[215]	42	832	0.05	[0.04; 0.07]
HeChuJie 2019[217]	241	914	0.26	[0.24; 0.29]
HeLanXiang 2018[218]	37	318	0.12	[0.08; 0.16]
HeLiYing 2017[219]	95	850	0.11	[0.09; 0.13]
HeMengTing 2020[220]	14	240	0.06	[0.03; 0.10]
HeQiuYu 2019[221]	402	3066	0.13	[0.12; 0.14]
HeFangQing 2017[223]	17	150	0.11	[0.07; 0.18]
HongChengJi 2016[225]	80	517	0.15	[0.12; 0.19]
HongWanSheng 2015[226]	46	236	0.19	[0.15; 0.25]
HongWanSheng 2015[227]	71	507	0.14	[0.11; 0.17]
HongYing 2019[228]	134	911	0.15	[0.12; 0.17]
HongZhanTong 2015[229]	192	1587	0.12	[0.11; 0.14]
HuHui 2018[234]	54	700	0.08	[0.06; 0.10]
HuJing 2019[235]	41	261	0.16	[0.12; 0.21]
HuJuMei 2014[236]	91	454	0.20	[0.16; 0.24]
HuTingTing 2013[237]	39	312	0.12	[0.09; 0.17]
HuTingTing 2013[238]	11	127	0.09	[0.04; 0.15]
HuZhuo 2017[240]	14	190	0.07	[0.04; 0.12]
HuaWeiYu 2017[243]	94	572	0.16	[0.13; 0.20]
HuangAiXia 2020[244]	38	449	0.08	[0.06; 0.11]
HuangEnMiao 2019[249]	465	32831	0.01	[0.01; 0.02]
HuangFengGuang 2018[250]	45	673	0.07	[0.05; 0.09]
HuangPengFei 2018[255]	59	258	0.23	[0.18; 0.28]
HuangTianRan 2020[258]	15	48	0.31	[0.19; 0.46]
HuangYong 2018[262]	732	5026	0.15	[0.14; 0.16]
JiYanLi 2020[266]	136	972	0.14	[0.12; 0.16]
JiYanLi 2017[267]	37	354	0.10	[0.07; 0.14]
JiLei 2018[268]	101	501	0.20	[0.17; 0.24]
JiLei 2019[273]	183	1259	0.15	[0.13; 0.17]
JiXinFeng 2012[275]	27	241	0.11	[0.08; 0.16]
Li−Li Jia 2016[277]	58	502	0.12	[0.09; 0.15]
JiaNing 2012[278]	36	214	0.17	[0.12; 0.23]
JiangLingLing 2018[279]	15	2610	0.01	[0.00; 0.01]
JiangXiao 2018[280]	14	300	0.05	[0.03; 0.08]
JiangZhenLong 2014[281]	66	350	0.19	[0.15; 0.23]
JiangHongBo 2019[282]	63	400	0.16	[0.12; 0.20]
JiangChunMei 2013[286]	17	271	0.06	[0.04; 0.10]
JiangCuiLian 2016[287]	67	322	0.21	[0.17; 0.26]
JiangCuiLian 2018[288]	31	300	0.10	[0.07; 0.14]

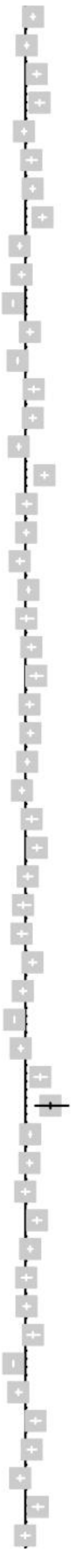

JiangCuiLian 2016[289]	27	178	0.15	[0.10; 0.21]
JiangHongJun 2020[290]	325	2230	0.15	[0.13; 0.16]
JiangWenJun 2019[292]	22	121	0.18	[0.12; 0.26]
JiangZhuoJing 2020[295]	93	645	0.14	[0.12; 0.17]
JiaoYang 2019[296]	237	1451	0.16	[0.14; 0.18]
JinDan 2017[297]	60	1309	0.05	[0.04; 0.06]
JinDi 2021[298]	31	442	0.07	[0.05; 0.10]
JinSaiYan 2012[299]	45	325	0.14	[0.10; 0.18]
JinSongGuo 2015[300]	80	831	0.10	[0.08; 0.12]
JinXiaoMin 2018[301]	43	514	0.08	[0.06; 0.11]
KangYanJu 2017[304]	21	88	0.24	[0.15; 0.34]
KongBoLi 2015[305]	212	819	0.26	[0.23; 0.29]
ZhangJinMing 2017[306]	91	636	0.14	[0.12; 0.17]
LanYingYing 2021[309]	129	1012	0.13	[0.11; 0.15]
LeiLei 2016[310]	274	7120	0.04	[0.03; 0.04]
LeiYue 2020[312]	121	1028	0.12	[0.10; 0.14]
LiJian 2014[313]	262	1001	0.26	[0.23; 0.29]
LiJingQuan 2013[314]	100	224	0.45	[0.38; 0.51]
LiBo 2014[317]	64	227	0.28	[0.22; 0.35]
LiHaiQing 2019[321]	38	752	0.05	[0.04; 0.07]
LiHui 2019[322]	32	363	0.09	[0.06; 0.12]
LiJiShan 2017[323]	133	714	0.19	[0.16; 0.22]
LiJiShan 2012[325]	14	104	0.13	[0.08; 0.22]
LiJiYao 2019[327]	64	520	0.12	[0.10; 0.15]
LiJie 2020[330]	153	2052	0.07	[0.06; 0.09]
LiJing 2015[331−1]	15	108	0.14	[0.08; 0.22]
LiJing 2015[331−2]	15	109	0.14	[0.08; 0.22]
LiJing 2021[332]	260	1957	0.13	[0.12; 0.15]
LiJing 2017[333]	149	687	0.22	[0.19; 0.25]
LiJunXia 2014[335]	76	718	0.11	[0.08; 0.13]
LiLiYan 2019[337]	52	152	0.34	[0.27; 0.42]
LiPing 2020[339]	6	433	0.01	[0.01; 0.03]
LiRongHua 2019[341]	27	285	0.09	[0.06; 0.13]
LiRuiFeng 2018[342]	155	202	0.77	[0.70; 0.82]
LiRuiQiang 2014[343]	75	282	0.27	[0.22; 0.32]
LiWei 2015[351]	5	20	0.25	[0.09; 0.49]
LiXiang 2015[353−1]	70	385	0.18	[0.14; 0.22]
LiXiang 2015[353−2]	69	385	0.18	[0.14; 0.22]
LiXiang 2013[354]	51	375	0.14	[0.10; 0.17]
LiXiang 2019[355]	177	1335	0.13	[0.11; 0.15]
LiXiang 2019[356]	38	619	0.06	[0.04; 0.08]
LiXiaoLe 2012[357]	64	856	0.07	[0.06; 0.09]
LiXiaoYu 2012[358]	24	2046	0.01	[0.01; 0.02]
LiYan 2015[360]	23	160	0.14	[0.09; 0.21]
LiYanFen 2014[362]	146	831	0.18	[0.15; 0.20]
LiYanHua 2021[363]	277	1513	0.18	[0.16; 0.20]
LiYanYan 2017[364]	62	423	0.15	[0.11; 0.18]
LiZhen 2009[367]	38	80	0.47	[0.36; 0.59]
LiangCuiQiong 2020[369]	179	1050	0.17	[0.15; 0.19]
LiangJunHe 2015[370]	282	908	0.31	[0.28; 0.34]
LiangLiRong 2021[371]	236	2144	0.11	[0.10; 0.12]
LiangLiang 2017[372]	23	201	0.11	[0.07; 0.17]
LiangLu 2014[373]	220	1607	0.14	[0.12; 0.15]
LiangQi 2017[375]	16	483	0.03	[0.02; 0.05]
LiangXiaoLian 2015[377]	75	546	0.14	[0.11; 0.17]

LiangYing 2021[378]	76	468	0.16 [0.13; 0.20]
LiaoYang 2011[382]	122	484	0.25 [0.21; 0.29]
LinDan 2016[383]	48	395	0.12 [0.09; 0.16]
LinLiQun 2020[385−1]	16	202	0.08 [0.05; 0.13]
LinLiQun 2020[385−2]	33	405	0.08 [0.06; 0.11]
LinLiJuan 2016[386]	51	326	0.16 [0.12; 0.20]
LinLin 2019[387]	150	1017	0.15 [0.13; 0.17]
LinQian 2012[389]	67	300	0.22 [0.18; 0.27]
LinSheng 2019[392]	2330	12083	0.19 [0.19; 0.20]
LinYiXiong 2016[394]	381	1150	0.33 [0.30; 0.36]
LiuBaiWei 2015[397−1]	134	946	0.14 [0.12; 0.17]
LiuBaiWei 2015[397−2]	269	1892	0.14 [0.13; 0.16]
LiuChen 2020[400]	138	1398	0.10 [0.08; 0.12]
LiuDaJing 2018[401]	10	983	0.01 [0.00; 0.02]
LiuDan 2021[402]	1938	9397	0.21 [0.20; 0.21]
LiuGuiDan 2017[405]	12	494	0.02 [0.01; 0.04]
LiuGuoRong 2019[407]	38	239	0.16 [0.12; 0.21]
LiuHaiBo 2020[408]	23	395	0.06 [0.04; 0.09]
LiuHanZhao 2018[409]	49	381	0.13 [0.10; 0.17]
LiuJiZhao 2020[412]	65	588	0.11 [0.09; 0.14]
LiuLiang 2015[414]	64	526	0.12 [0.09; 0.15]
LiuKaiQian 2013[416]	26	78	0.33 [0.23; 0.45]
LiuLu 2015[418]	63	543	0.12 [0.09; 0.15]
LiuMeiFang 2011[419]	9	90	0.10 [0.05; 0.18]
LiuTingTing 2019[425]	33	1029	0.03 [0.02; 0.04]
LiuWei 2021[426]	107	485	0.22 [0.18; 0.26]
LiuWenFu 2018[427]	42	2411	0.02 [0.01; 0.02]
LiuWuGao 2017[429]	215	6262	0.03 [0.03; 0.04]
LiuXiaoFeng 2015[431]	54	595	0.09 [0.07; 0.12]
LiuXiuLan 2019[432]	24	202	0.12 [0.08; 0.17]
LiuXueJie 2019[434]	69	412	0.17 [0.13; 0.21]
LiuYaWei 2013[435]	91	619	0.15 [0.12; 0.18]
LiuLiJiang 2016[439]	18	609	0.03 [0.02; 0.05]
LongHaoYu 2010[440]	29	252	0.12 [0.08; 0.16]
LongQiZhi 2019[443]	65	580	0.11 [0.09; 0.14]
LongQianJin 2020[444]	13	120	0.11 [0.06; 0.18]
LouQian 2012[445]	12	130	0.09 [0.05; 0.16]
LuHongPing 2020[446]	132	670	0.20 [0.17; 0.23]
LuLiBin 2020[447−1]	36	171	0.21 [0.15; 0.28]
LuLiBin 2020[447−2]	36	172	0.21 [0.15; 0.28]
LuLiBin 2017[448]	46	239	0.19 [0.14; 0.25]
LuXiangDui 2019[449]	39	2201	0.02 [0.01; 0.02]
LuZhanPeng 2014[450]	135	838	0.16 [0.14; 0.19]
LuDongLei 2020[452]	895	6664	0.13 [0.13; 0.14]
LuHanMing 2018[453]	70	419	0.17 [0.13; 0.21]
LuHuan 2021[455]	85	474	0.18 [0.15; 0.22]
LuQun 2016[457]	82	2376	0.03 [0.03; 0.04]
LuGongJin 2017[459]	59	699	0.08 [0.06; 0.11]
LuLiJun 2015[460]	174	999	0.17 [0.15; 0.20]
LuPeng 2015[461]	399	4568	0.09 [0.08; 0.10]
LuanMingChun 2017[464]	24	1253	0.02 [0.01; 0.03]
LuanMingChun 2018[465]	25	1900	0.01 [0.01; 0.02]
LuoHengLi 2018[468]	12	135	0.09 [0.05; 0.15]
LuoJianZhong 2016[469]	17	280	0.06 [0.04; 0.10]

LuoKaiWei 2018[470]	81	1122	0.07 [0.06; 0.09]
LuoLan 2013[471]	395	1066	0.37 [0.34; 0.40]
LuoLiang 2018[473]	51	321	0.16 [0.12; 0.20]
LuoXueMei 2016[476]	345	1822	0.19 [0.17; 0.21]
LuoCheng 2020[477]	5	121	0.04 [0.01; 0.09]
LuoLingFei 2017[478]	207	803	0.26 [0.23; 0.29]
LuoShanCai 2018[479]	78	1672	0.05 [0.04; 0.06]
MaHuaPing 2016[480]	20	95	0.21 [0.13; 0.31]
MaJuHong 2019[481]	4	163	0.02 [0.01; 0.06]
MaJuan 2020[482]	52	375	0.14 [0.11; 0.18]
MaLiangLiang 2018[483]	19	301	0.06 [0.04; 0.10]
MaShuBo 2015[485]	38	2836	0.01 [0.01; 0.02]
MaXin 2016[488]	67	1033	0.06 [0.05; 0.08]
MaXueLian 2021[489]	41	250	0.16 [0.12; 0.22]
MaYaPing 2018[490]	190	883	0.22 [0.19; 0.24]
MaYongJun 2016[491]	176	822	0.21 [0.19; 0.24]
MaoJianYing 2016[492]	98	2486	0.04 [0.03; 0.05]
MeiGuoYong 2019[494]	15	308	0.05 [0.03; 0.08]
MengLiXia 2020[496]	25	110	0.23 [0.15; 0.32]
MengQingHe 2018[497]	48	169	0.28 [0.22; 0.36]
MengQingHe 2016[498]	15	101	0.15 [0.09; 0.23]
MiaoShengHao 2018[500]	111	2473	0.04 [0.04; 0.05]
MiaoShengHao 2017[501]	58	1030	0.06 [0.04; 0.07]
MinPei 2011[502]	98	912	0.11 [0.09; 0.13]
MinPei 2012[503]	14	241	0.06 [0.03; 0.10]
MiaoGuoZhong 2013[506]	12	66	0.18 [0.10; 0.30]
NaYongDong 2016[508]	86	1210	0.07 [0.06; 0.09]
NongHao 2020[514]	238	1199	0.20 [0.18; 0.22]
PanHao 2017[517]	1172	6543	0.18 [0.17; 0.19]
PanLiFeng 2015[518]	1053	6392	0.16 [0.16; 0.17]
PanLiFeng 2012[519]	110	1327	0.08 [0.07; 0.10]
PanLiFeng 2015[520−1]	362	1701	0.21 [0.19; 0.23]
PanLiFeng 2015[520−2]	362	1702	0.21 [0.19; 0.23]
PangBeiBei 2016[523]	324	615	0.53 [0.49; 0.57]
PengPai 2018[527]	154	791	0.19 [0.17; 0.22]
QiHuiZhou 2014[530]	63	116	0.54 [0.45; 0.64]
QiXianQun 2021[531]	44	529	0.08 [0.06; 0.11]
QiJi 2019[533]	14	264	0.05 [0.03; 0.09]
QianYanHua 2011[538]	7	163	0.04 [0.02; 0.09]
QiaoHongYing 2021[541]	17	641	0.03 [0.02; 0.04]
QiaoKun 2014[542]	180	539	0.33 [0.29; 0.38]
QiaoYingQin 2016[543]	67	511	0.13 [0.10; 0.16]
QinMeng 2014[546]	7	47	0.15 [0.06; 0.28]
QiuCanLin 2015[550]	156	431	0.36 [0.32; 0.41]
QiuXiang 2019[552]	32	1495	0.02 [0.01; 0.03]
QiuZhengYong 2019[553]	273	5412	0.05 [0.04; 0.06]
RenShuMin 2018[558]	17	340	0.05 [0.03; 0.08]
RenYaPing 2020[559]	97	484	0.20 [0.17; 0.24]
RenYan 2020[560]	23	105	0.22 [0.14; 0.31]
RenYan 2018[561]	53	362	0.15 [0.11; 0.19]
RongJiangRui 2014[563]	18	335	0.05 [0.03; 0.08]
RongXiaoSu 2021[564]	47	347	0.14 [0.10; 0.18]
RuanYang 2019[565]	21	1580	0.01 [0.01; 0.02]
SangHao 2016[567]	344	858	0.40 [0.37; 0.43]
SangHao 2019[568]	70	880	0.08 [0.06; 0.10]

SangXiangLai 2016[569]	61	1545	0.04 [0.03; 0.05]
SangXiangLai 2018[570]	607	1580	0.38 [0.36; 0.41]
ShaBiReMu·TuoHeTaMu 2013[571]	73	379	0.19 [0.15; 0.24]
ShaBiReMu·TuoHeTaMu 2018[572]	150	895	0.17 [0.14; 0.19]
ShangLiHong 2016[573]	229	1064	0.22 [0.19; 0.24]
ShangXiaoChun 2020[574]	310	1350	0.23 [0.21; 0.25]
ShenHongWei 2019[575]	228	1972	0.12 [0.10; 0.13]
ShenTuPingPing 2018[576]	90	1339	0.07 [0.05; 0.08]
ShiAiPing 2021[578]	54	419	0.13 [0.10; 0.16]
ShiQianFeng 2014[580]	9	124	0.07 [0.03; 0.13]
ShiQianFeng 2015[581]	19	124	0.15 [0.09; 0.23]
ShiPing 2016[583]	52	338	0.15 [0.12; 0.20]
ShiXin 2020[584]	40	560	0.07 [0.05; 0.10]
ShiWenFeng 2019[585]	116	1105	0.10 [0.09; 0.12]
ShiYongLin 2014[586]	66	549	0.12 [0.09; 0.15]
ShuYouPing 2020[588]	80	776	0.10 [0.08; 0.13]
SuJing 2016[602]	55	987	0.06 [0.04; 0.07]
SuTong 2017[603]	372	2211	0.17 [0.15; 0.18]
SuWenJun 2013[605]	98	912	0.11 [0.09; 0.13]
SuWenZhe 2019[606]	220	854	0.26 [0.23; 0.29]
SunBo 2021[607]	60	680	0.09 [0.07; 0.11]
SunHuaMin 2019[609]	54	1244	0.04 [0.03; 0.06]
SunJianFei 2014[610]	20	120	0.17 [0.10; 0.25]
SunMingHua 2015[615]	24	170	0.14 [0.09; 0.20]
SunQingShuang 2017[617]	17	201	0.08 [0.05; 0.13]
SunYaNa 2020[619]	35	271	0.13 [0.09; 0.18]
SunYangMing 2016[620]	162	1541	0.11 [0.09; 0.12]
SunYangMing 2016[621]	92	863	0.11 [0.09; 0.13]
SunYu 2018[622]	36	201	0.18 [0.13; 0.24]
SunYueLin 2017[623]	26	419	0.06 [0.04; 0.09]
QinLin 2017[626]	15	75	0.20 [0.12; 0.31]
TanWeiWei 2015[628]	6	753	0.01 [0.00; 0.02]
TanWeiWei 2018[629]	34	363	0.09 [0.07; 0.13]
TangXiang 2014[632]	84	384	0.22 [0.18; 0.26]
TangZhen 2016[635]	1308	16658	0.08 [0.07; 0.08]
TianDeng 2018[637]	47	238	0.20 [0.15; 0.25]
TianGeng 2015[638]	133	519	0.26 [0.22; 0.30]
TianHong 2011[639]	31	226	0.14 [0.10; 0.19]
TianJiGui 2019[640]	11	228	0.05 [0.02; 0.08]
WangHui 2016[644]	136	732	0.19 [0.16; 0.22]
WangYang 2014[647]	237	2236	0.11 [0.09; 0.12]
WangAnNa 2016[648]	15	75	0.20 [0.12; 0.31]
WangChunRong 2015[650]	99	579	0.17 [0.14; 0.20]
WangDaHu 2012[651]	99	14281	0.01 [0.01; 0.01]
WangDongYue 2016[653]	232	2234	0.10 [0.09; 0.12]
WangGe 2016[654]	41	572	0.07 [0.05; 0.10]
WangHaiYan 2014[655]	75	300	0.25 [0.20; 0.30]
WangHuan 2017[658]	21	530	0.04 [0.02; 0.06]
WangJie 2016[661]	4	3190	0.00 [0.00; 0.00]
WangJuan 2012[662]	40	198	0.20 [0.15; 0.26]
WangLiLi 2021[667]	326	2123	0.15 [0.14; 0.17]
WangPing 2016[672]	34	64	0.53 [0.40; 0.66]
WangQiMei 2019[673]	179	4243	0.04 [0.04; 0.05]
WangSanTao 2018[674]	84	1393	0.06 [0.05; 0.07]
WangSheLiang 2011[675]	30	300	0.10 [0.07; 0.14]

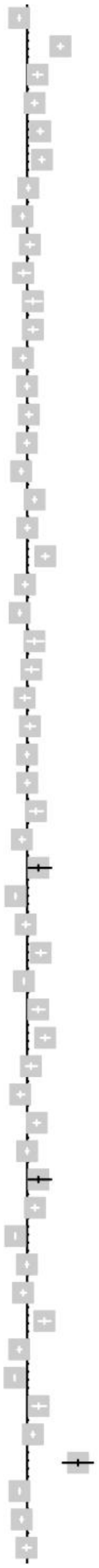

WangTongYu 2019[679]	356	2490	0.14 [0.13; 0.16]
WangWeiRu 2013[680]	13	96	0.14 [0.07; 0.22]
WangWenYing 2011[681]	204	650	0.31 [0.28; 0.35]
WangWenQing 2020[682]	1428	9301	0.15 [0.15; 0.16]
WangWenLei 2017[683]	125	2450	0.05 [0.04; 0.06]
WangWeiXiang 2021[684]	292	1358	0.22 [0.19; 0.24]
WangXi 2016[685]	140	650	0.22 [0.18; 0.25]
WangXi 2017[686]	67	340	0.20 [0.16; 0.24]
WangXi 2018[687]	51	846	0.06 [0.05; 0.08]
WangXiaoYi 2017[690]	13	120	0.11 [0.06; 0.18]
WangXiaoYi 2017[691]	13	120	0.11 [0.06; 0.18]
WangYaFang 2019[694]	86	3766	0.02 [0.02; 0.03]
WangYanBo 2017[695]	78	344	0.23 [0.18; 0.27]
WangYanHua 2021[696]	43	300	0.14 [0.11; 0.19]
WangYanYan 2019[697]	64	1123	0.06 [0.04; 0.07]
WangYongXia 2012[698]	27	295	0.09 [0.06; 0.13]
WangYu 2019[699]	169	938	0.18 [0.16; 0.21]
WangYuPing 2016[700]	23	848	0.03 [0.02; 0.04]
WangYuanYuan 2015[701]	48	459	0.10 [0.08; 0.14]
WangYue 2020[702]	65	718	0.09 [0.07; 0.11]
WangYueSheng 2015[703]	58	506	0.11 [0.09; 0.15]
WangZiYou 2020[704]	87	976	0.09 [0.07; 0.11]
WeiKongFu 2014[707]	120	385	0.31 [0.27; 0.36]
WeiXinHong 2020[709]	31	433	0.07 [0.05; 0.10]
WengXiaoQin 2017[712]	137	1000	0.14 [0.12; 0.16]
WuBingShan 2018[714]	141	892	0.16 [0.13; 0.18]
WuJing 2015[720]	7	42	0.17 [0.07; 0.31]
WuJing 2018[721]	6	153	0.04 [0.01; 0.08]
WuJing 2019[722]	13	310	0.04 [0.02; 0.07]
WuPei 2011[724]	146	1878	0.08 [0.07; 0.09]
WuShengHai 2019[726]	66	605	0.11 [0.09; 0.14]
WuWei 2012[728]	113	540	0.21 [0.18; 0.25]
WuXiaoFang 2017[731]	256	873	0.29 [0.26; 0.32]
WuYaTing 2019[740]	9	915	0.01 [0.00; 0.02]
XiaoLin 2014[747]	60	319	0.19 [0.15; 0.24]
XiaoYong 2016[748]	40	409	0.10 [0.07; 0.13]
XieChunYan 2014[750]	153	1095	0.14 [0.12; 0.16]
XieHongYi 2020[751]	48	204	0.24 [0.18; 0.30]
XieSiRou 2017[753]	62	600	0.10 [0.08; 0.13]
XieYaXian 2021[755]	110	1050	0.10 [0.09; 0.12]
XingYuFang 2019[760]	21	1079	0.02 [0.01; 0.03]
XiongFeiYu 2016[761]	176	2148	0.08 [0.07; 0.09]
XiongXiaoShun 2017[762]	5	7138	0.00 [0.00; 0.00]
XuJi 2021[763]	712	2021	0.35 [0.33; 0.37]
XuDan 2014[764]	125	748	0.17 [0.14; 0.20]
XuDan 2012[765]	24	440	0.05 [0.04; 0.08]
XuFenFen 2018[766]	170	2793	0.06 [0.05; 0.07]
XuLiXia 2016[768]	61	417	0.15 [0.11; 0.18]
XuQin 2014[769]	55	513	0.11 [0.08; 0.14]
XuRuiQuan 2021[770]	259	856	0.30 [0.27; 0.33]
XuZhongQing 2016[773]	148	1554	0.10 [0.08; 0.11]
XuHuaJing 2019[776]	14	108	0.13 [0.07; 0.21]
XuJinFeng 2017[777]	87	1605	0.05 [0.04; 0.07]
XuJun 2012[780]	7	218	0.03 [0.01; 0.07]

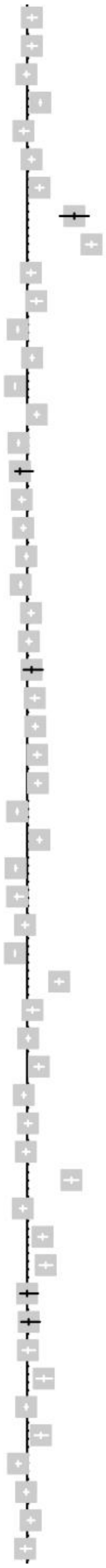

XuLiQin 2015[781−1]	45	311	0.14	[0.11; 0.19]
XuLiQin 2015[781−2]	45	311	0.14	[0.11; 0.19]
XuXiaoChan 2015[783]	64	642	0.10	[0.08; 0.13]
XueCaoYi 2017[785]	2186	10126	0.22	[0.21; 0.22]
YanXin 2018[787]	15	200	0.07	[0.04; 0.12]
YanYuXiao 2020[788]	112	785	0.14	[0.12; 0.17]
YanWei 2019[789]	121	578	0.21	[0.18; 0.24]
YanYan 2013[790]	35	70	0.50	[0.38; 0.62]
YanYan 2012[791]	274	426	0.64	[0.60; 0.69]
YanWei 2020[793]	48	351	0.14	[0.10; 0.18]
YanFang 2018[794]	48	264	0.18	[0.14; 0.23]
YangHui 2020[799]	54	2000	0.03	[0.02; 0.04]
YangHui 2016[800]	318	2161	0.15	[0.13; 0.16]
YangJinHong 2020[803]	73	17516	0.00	[0.00; 0.01]
YangLi 2018[805]	240	1292	0.19	[0.16; 0.21]
YangMei 2017[806]	52	1557	0.03	[0.03; 0.04]
YangMing 2011[807]	2	44	0.05	[0.01; 0.15]
YangRuSong 2016[809]	22	361	0.06	[0.04; 0.09]
YangTianChi 2016[812]	53	728	0.07	[0.06; 0.09]
YangXianDa 2019[814]	183	1855	0.10	[0.09; 0.11]
YangXiaoJin 2016[815]	102	1982	0.05	[0.04; 0.06]
YangYanNa 2018[816]	113	818	0.14	[0.12; 0.16]
YangYiLong 2015[817]	92	766	0.12	[0.10; 0.15]
YaoJianXiang 2016[823]	12	84	0.14	[0.08; 0.24]
YaoLiLi 2019[825]	70	414	0.17	[0.13; 0.21]
YaoLiLi 2020[826]	188	1083	0.17	[0.15; 0.20]
YaoXiuPing 2021[828]	116	614	0.19	[0.16; 0.22]
YeHongYan 2013[829]	176	900	0.20	[0.17; 0.22]
YeYanHua 2018[834]	28	1259	0.02	[0.01; 0.03]
YeYuHui 2015[835]	364	1744	0.21	[0.19; 0.23]
YiMing 2018[836]	5	550	0.01	[0.00; 0.02]
YiYing 2013[838]	2	94	0.02	[0.00; 0.07]
ZhaoWei 2011[841]	38	432	0.09	[0.06; 0.12]
YouXingYong 2020[842]	22	5426	0.00	[0.00; 0.01]
YuFangYuan 2020[843]	265	709	0.37	[0.34; 0.41]
YuMiao 2019[844]	30	200	0.15	[0.10; 0.21]
YuYingHui 2020[846]	171	1497	0.11	[0.10; 0.13]
YuGuangQing 2013[847]	82	403	0.20	[0.17; 0.25]
YuJinCun 2020[850]	79	1017	0.08	[0.06; 0.10]
YuanJianMing 2019[853]	48	428	0.11	[0.08; 0.15]
YuanLu 2018[855]	38	400	0.10	[0.07; 0.13]
YuanYongJuan 2019[856]	137	288	0.48	[0.42; 0.54]
ZhangDongYu 2020[865]	25	360	0.07	[0.05; 0.10]
ZhangHaiLong 2013[866]	114	486	0.23	[0.20; 0.27]
ZhangHuanZhu 2015[870]	67	257	0.26	[0.21; 0.32]
ZhangJianMin 2014[872−1]	8	76	0.11	[0.05; 0.20]
ZhangJianMin 2014[872−2]	9	76	0.12	[0.06; 0.21]
ZhangJianMin 2014[872−3]	17	152	0.11	[0.07; 0.17]
ZhangJin 2018[873]	42	172	0.24	[0.18; 0.32]
ZhangJing 2016[874]	184	1878	0.10	[0.08; 0.11]
ZhangLiShuang 2019[876]	47	213	0.22	[0.17; 0.28]
ZhangLi 2015[877]	9	303	0.03	[0.01; 0.06]
ZhangLingLing 2016[878]	82	842	0.10	[0.08; 0.12]
ZhangMin 2015[881]	72	532	0.14	[0.11; 0.17]
ZhangQian 2017[884]	15	169	0.09	[0.05; 0.14]

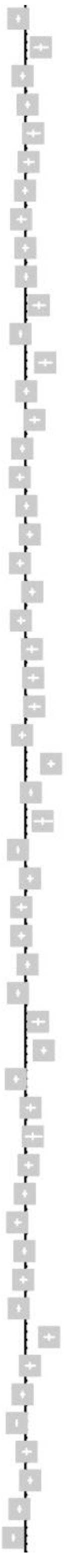

ZhangShuHong 2019[886]	58	1240	0.05	[0.04; 0.06]
ZhangShuang 2016[887]	56	240	0.23	[0.18; 0.29]
ZhangWangSheng 2020[889]	110	1341	0.08	[0.07; 0.10]
ZhangWeiWei 2021[890]	232	1870	0.12	[0.11; 0.14]
ZhangYaKong 2017[892]	36	212	0.17	[0.12; 0.23]
ZhangYaLin 2018[893]	40	303	0.13	[0.10; 0.18]
ZhangYan 2017[895]	136	1320	0.10	[0.09; 0.12]
ZhangYanLi 2013[896]	17	242	0.07	[0.04; 0.11]
ZhangYanLi 2019[897]	101	961	0.11	[0.09; 0.13]
ZhangYanFei 2018[899]	398	3592	0.11	[0.10; 0.12]
ZhangYongHong 2015[901]	62	286	0.22	[0.17; 0.27]
ZhangYong 2017[902]	309	4728	0.07	[0.06; 0.07]
ZhangZhiQiang 2019[905]	79	293	0.27	[0.22; 0.32]
ZhangShaSha 2017[910]	166	1476	0.11	[0.10; 0.13]
ZhaoDan 2017[912]	74	409	0.18	[0.14; 0.22]
ZhaoDan 2019[913]	113	1412	0.08	[0.07; 0.10]
ZhaoHuiLing 2017[914]	20	336	0.06	[0.04; 0.09]
ZhaoJiaYong 2016[915]	225	1964	0.11	[0.10; 0.13]
ZhaoLiJiang 2019[917]	160	1125	0.14	[0.12; 0.16]
ZhaoTianWang 2018[922]	27	440	0.06	[0.04; 0.09]
ZhaoWenNa 2019[925]	236	1451	0.16	[0.14; 0.18]
ZhaoXiangJu 2018[926]	24	352	0.07	[0.04; 0.10]
ZhaoXiangJu 2018[927]	47	295	0.16	[0.12; 0.21]
ZhaoXiaoLing 2013[928]	63	371	0.17	[0.13; 0.21]
ZhaoXueQin 2014[930]	55	307	0.18	[0.14; 0.23]
ZhaoYaLi 2020[931]	40	510	0.08	[0.06; 0.11]
ZhaoYunQing 2020[934]	471	1478	0.32	[0.29; 0.34]
ZhaoYun 2018[935]	871	5763	0.15	[0.14; 0.16]
ZhengLei 2013[938]	36	145	0.25	[0.18; 0.33]
ZhengShuFa 2016[939]	207	4680	0.04	[0.04; 0.05]
ZhengWenLong 2019[940]	180	1249	0.14	[0.13; 0.16]
ZhengXiaoYan 2018[941]	16	222	0.07	[0.04; 0.11]
ZhengYaPing 2016[942]	39	543	0.07	[0.05; 0.10]
ZhuGeXiaoLing 2011[943]	265	2144	0.12	[0.11; 0.14]
ZhengYuXun 2020[945]	77	1739	0.04	[0.04; 0.06]
ZhongQuanChang 2014[947]	59	280	0.21	[0.16; 0.26]
ZhongYanXu 2017[949]	577	2236	0.26	[0.24; 0.28]
ZhouAiMin 2020[951]	11	408	0.03	[0.01; 0.05]
ZhouHuiFang 2018[953]	65	439	0.15	[0.12; 0.18]
ZhouMingLi 2017[957]	22	132	0.17	[0.11; 0.24]
ZhouYinZhu 2018[963]	72	547	0.13	[0.10; 0.16]
ZhouYing 2017[965]	177	1754	0.10	[0.09; 0.12]
ZhuFang 2017[969]	11	290	0.04	[0.02; 0.07]
ZhuGuanQi 2020[970]	245	2514	0.10	[0.09; 0.11]
ZhuHuiLin 2020[972]	41	467	0.09	[0.06; 0.12]
ZhuJiHua 2020[973]	76	1520	0.05	[0.04; 0.06]
ZhuMin 2019[974]	160	530	0.30	[0.26; 0.34]
ZhuTingTing 2015[975]	49	341	0.14	[0.11; 0.19]
ZhuXiaoLu 2018[977]	129	1565	0.08	[0.07; 0.10]
ZhuXun 2019[978]	306	9047	0.03	[0.03; 0.04]
ZhuLin 2020[979]	42	364	0.12	[0.08; 0.15]
ZouHuiYing 2016[981]	310	2132	0.15	[0.13; 0.16]
ZouYongWen 2021[982]	76	1357	0.06	[0.04; 0.07]
Yuan-yun Ao 2014[983]	2	466	0.00	[0.00; 0.02]

Cao RR 2021[984]	242	1181	0.20	[0.18; 0.23]
Chang H 2017[985]	123	1360	0.09	[0.08; 0.11]
Chen C 2020[986]	134	1849	0.07	[0.06; 0.09]
Chen H 2015[988]	90	529	0.17	[0.14; 0.20]
Chen Y 2013[990]	102	811	0.13	[0.10; 0.15]
JialLiPing 2021[991]	215	1213	0.18	[0.16; 0.20]
Fu JG 2016[996]	164	413	0.40	[0.35; 0.45]
Z Gao 2015[997]	263	3832	0.07	[0.06; 0.08]
Gao Z 2015[998]	191	640	0.30	[0.26; 0.34]
Gong XH 2018[999]	1744	8797	0.20	[0.19; 0.21]
Han J 2015[1002]	193	809	0.24	[0.21; 0.27]
Han J 2018[1003]	204	1001	0.20	[0.18; 0.23]
T He 2017[1004]	156	4612	0.03	[0.03; 0.04]
Ji L 2020[1009]	100	551	0.18	[0.15; 0.22]
Jia L 2017[1010]	304	999	0.30	[0.28; 0.33]
Jia LP 2014[1011]	182	249	0.73	[0.67; 0.78]
Kuang X 2019[1013]	1077	7883	0.14	[0.13; 0.14]
Li HY 2019[1014]	450	1863	0.24	[0.22; 0.26]
Li J 2018[1016]	64	424	0.15	[0.12; 0.19]
Liu J 2018[1022]	28	35	0.80	[0.63; 0.92]
Liu P 2014[1023]	34	124	0.27	[0.20; 0.36]
Liu X 2015[1024]	328	362	0.91	[0.87; 0.93]
Lu L 2015[1026]	126	436	0.29	[0.25; 0.33]
Lu L 2019[1027]	220	1433	0.15	[0.14; 0.17]
Lu QB 2015[1028]	1120	2140	0.52	[0.50; 0.54]
Mai H 2016[1031]	9	124	0.07	[0.03; 0.13]
Mai H 2013[1032]	26	171	0.15	[0.10; 0.21]
Pan L 2016[1033]	539	2169	0.25	[0.23; 0.27]
Qiao N 2017[1036]	115	5633	0.02	[0.02; 0.02]
Ren Z 2013[1038]	130	500	0.26	[0.22; 0.30]
Sang S 2014[1040]	66	685	0.10	[0.08; 0.12]
H. Shen 2016[1042]	34	412	0.08	[0.06; 0.11]
Shen W 2020[1043]	139	1464	0.09	[0.08; 0.11]
Shen XX 2019[1044]	21	634	0.03	[0.02; 0.05]
Shen Z 2013[1045]	37	748	0.05	[0.04; 0.07]
C Sun 2021[1047−1]	75	733	0.10	[0.08; 0.13]
C Sun 2021[1047−2]	76	733	0.10	[0.08; 0.13]
Sun XM 2016[1048−1]	19	202	0.09	[0.06; 0.14]
Sun XM 2016[1048−2]	5	222	0.02	[0.01; 0.05]
Tan D 2015[1049]	101	354	0.29	[0.24; 0.34]
ZhangJianYing 2020[1051]	28	120	0.23	[0.16; 0.32]
Wang L P 2021[1052]	9707	77855	0.12	[0.12; 0.13]
Wang X 2016[1053]	209	1681	0.12	[0.11; 0.14]
Wang X 2019[1054]	510	3421	0.15	[0.14; 0.16]
ZhangXiaoYan 2021[1056]	42	116	0.36	[0.27; 0.46]
W Wu 2014[1057]	210	983	0.21	[0.19; 0.24]
Wu X 2015[1058]	211	796	0.27	[0.23; 0.30]
C Xue 2018[1061]	1363	5927	0.23	[0.22; 0.24]
Xue L 2019[1062]	43	217	0.20	[0.15; 0.26]
Xue L 2016[1063]	25	215	0.12	[0.08; 0.17]
Xue L 2013[1064]	9	89	0.10	[0.05; 0.18]
Xue Y 2015[1065]	903	2114	0.43	[0.41; 0.45]
Yang F 2018[1066]	22	412	0.05	[0.03; 0.08]
Yang S 2019[1067]	13	81	0.16	[0.09; 0.26]
Yu J 2017[1069]	771	3877	0.20	[0.19; 0.21]

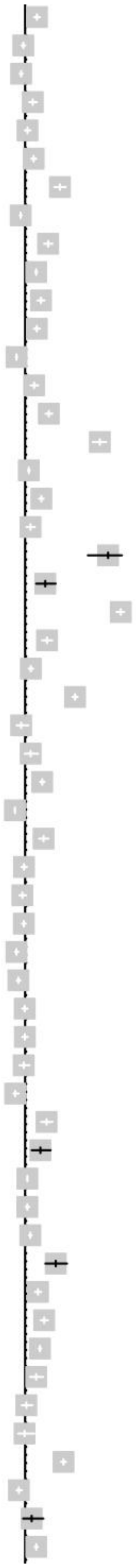

Zhang DM 2015[1070]	6	1128	0.01	[0.00; 0.01]
Zhang P 2016[1072]	196	746	0.26	[0.23; 0.30]
Zhang S 2011[1073]	60	201	0.30	[0.24; 0.37]
Zhang SX 2016[1074]	31	420	0.07	[0.05; 0.10]
Zhu XH 2016[1082]	88	381	0.23	[0.19; 0.28]
Zhu YN 2020[1083]	197	1220	0.16	[0.14; 0.18]
ZL Zhuang 2017[1084]	32	295	0.11	[0.08; 0.15]
Zou W 2015[1085]	383	1346	0.28	[0.26; 0.31]
CuiXiaoShuang 2013[1086]	96	566	0.17	[0.14; 0.20]
HouJun 2012[1087]	6	57	0.11	[0.04; 0.22]
PangBeiBei 2012[1089]	164	753	0.22	[0.19; 0.25]
WangGuoZheng 2014[1090]	49	1134	0.04	[0.03; 0.06]
XuShaoJian 2015[1092]	10	205	0.05	[0.02; 0.09]
YaoWeiLei 2013[1093]	531	2290	0.23	[0.21; 0.25]
Aixiaoman 2015[1094]	1	43	0.02	[0.00; 0.12]
ChenFengQin 2014[1095]	204	594	0.34	[0.31; 0.38]
LuXueDong 2012[1098]	38	528	0.07	[0.05; 0.10]
SunLiMei 2014[1099]	326	1932	0.17	[0.15; 0.19]
WangQiong 2013[1100]	38	307	0.12	[0.09; 0.17]
Xiao−Ling Zhu Ge 2011[1102]	191	920	0.21	[0.18; 0.24]
ZengHao 2019[1104]	493	96732	0.01	[0.00; 0.01]
ChenCen 2016[1105]	52	485	0.11	[0.08; 0.14]
DangWen 2019[1106]	219	1083	0.20	[0.18; 0.23]
HanNing 2016[1107]	55	324	0.17	[0.13; 0.22]
HeFei 2020[1108]	610	2185	0.28	[0.26; 0.30]
HeYaQing 2017[1109]	1613	8241	0.20	[0.19; 0.20]
HuTingTing 2013[1110]	32	360	0.09	[0.06; 0.12]
HuangDouDou 2015[1111]	100	716	0.14	[0.12; 0.17]
HuangYeWei 2018[1112]	552	2402	0.23	[0.21; 0.25]
JiXinFeng 2012[1113]	41	383	0.11	[0.08; 0.14]
LiWeiWei 2020[1114]	138	648	0.21	[0.18; 0.25]
LiuCheng 2017[1115]	277	1214	0.23	[0.20; 0.25]
LiuYun 2019[1116]	353	1384	0.26	[0.23; 0.28]
LuoCan 2011[1117]	28	241	0.12	[0.08; 0.16]
MaYan 2014[1118]	52	311	0.17	[0.13; 0.21]
QianMingMing 2015[1119]	85	648	0.13	[0.11; 0.16]
SaiLinTao 2014[1120]	122	1247	0.10	[0.08; 0.12]
SangShaoWei 2012[1121]	66	685	0.10	[0.08; 0.12]
ShenZhen 2014[1122]	139	846	0.16	[0.14; 0.19]
SuCheng 2020[1123]	137	1042	0.13	[0.11; 0.15]
TanZiMing 2018[1124]	49	683	0.07	[0.05; 0.09]
TangZhiJian 2013[1125]	27	384	0.07	[0.05; 0.10]
WangHaiYan 2017[1126]	70	671	0.10	[0.08; 0.13]
WangPeng 2015[1127]	32	295	0.11	[0.08; 0.15]
WangYanGe 2011[1128]	70	852	0.08	[0.06; 0.10]
WuJie 2017[1129]	48	181	0.27	[0.20; 0.34]
WuWei 2013[1130]	186	925	0.20	[0.18; 0.23]
XueChaoChao 2017[1131]	3	183	0.02	[0.00; 0.05]
ZhongXueFei 2014[1132]	119	560	0.21	[0.18; 0.25]
**Common effect model**		**991786**	**0.11**	**[0.11; 0.11]**
**Random effects model**			**0.12**	**[0.11; 0.12]**

Heterogeneity: $I^2 = 99\%$, $\tau^2 = 0.9290$, $p = 0$

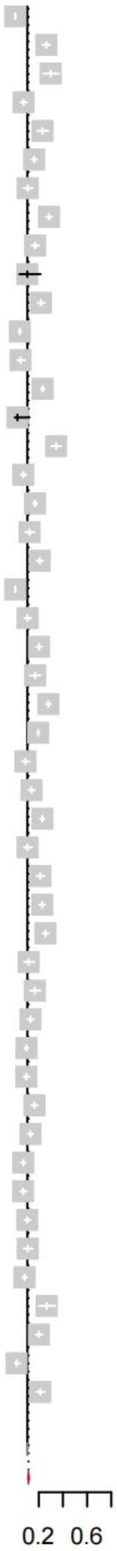

(a2)

Study Total	Events	Total	Attack rate	95%−CI
BaiAiLi 2020[3]	20	738	0.03	[0.02; 0.04]
BaiYao 2020[6]	22	169	0.13	[0.08; 0.19]
BaiYun 2018[8]	61	648	0.09	[0.07; 0.12]
BiHua 2014[10]	95	18930	0.01	[0.00; 0.01]
BiHua 2015[11]	55	1262	0.04	[0.03; 0.06]
CaiJian 2013[14−1]	53	2219	0.02	[0.02; 0.03]
CaiJian 2013[14−2]	257	16056	0.02	[0.01; 0.02]
Ming−Wei Cai 2021[17−1]	25	628	0.04	[0.03; 0.06]
Ming−Wei Cai 2021[17−2]	50	693	0.07	[0.05; 0.09]
Ming−Wei Cai 2021[17−3]	27	816	0.03	[0.02; 0.05]
Ming−Wei Cai 2021[17−4]	56	1202	0.05	[0.04; 0.06]
Cai MW 2018[18]	34	223	0.15	[0.11; 0.21]
Cai SJ 2018[20]	69	1781	0.04	[0.03; 0.05]
Cai SX 2017[21]	17	1276	0.01	[0.01; 0.02]
Cai W 2018[23−1]	21	25	0.84	[0.64; 0.95]
Cai W 2018[23−2]	38	117	0.32	[0.24; 0.42]
Cai W 2018[23−3]	217	2504	0.09	[0.08; 0.10]
Cai W 2017[24−1]	10	19	0.53	[0.29; 0.76]
Cai W 2017[24−2]	8	21	0.38	[0.18; 0.62]
Cai W 2017[24−3]	7	29	0.24	[0.10; 0.44]
Cai W 2017[24−4]	12	32	0.38	[0.21; 0.56]
Cai W 2017[24−5]	10	32	0.31	[0.16; 0.50]
Cai W 2017[24−6]	24	36	0.67	[0.49; 0.81]
Cai W 2017[24−7]	8	39	0.21	[0.09; 0.36]
Cai W 2017[24−8]	13	40	0.32	[0.19; 0.49]
Cai W 2017[24−9]	12	43	0.28	[0.15; 0.44]
Cai W 2017[24−10]	17	49	0.35	[0.22; 0.50]
Cai W 2017[24−11]	10	67	0.15	[0.07; 0.26]
Cai W 2017[24−12]	16	78	0.21	[0.12; 0.31]
CaiWenFeng 2013[25]	141	16600	0.01	[0.01; 0.01]
CaiWenFeng 2014[26]	27	816	0.03	[0.02; 0.05]
CaiXiuZhi 2018[27−1]	34	150	0.23	[0.16; 0.30]
CaiXiuZhi 2018[27−2]	34	400	0.08	[0.06; 0.12]
CaiXiuZhi 2018[27−3]	40	1045	0.04	[0.03; 0.05]
CaiXiuZhi 2018[27−4]	24	1279	0.02	[0.01; 0.03]
Cao RR 2020[30−1]	41	2853	0.01	[0.01; 0.02]
CaoShen 2019[32]	62	6966	0.01	[0.01; 0.01]
CaoXiaoPing 2018[33]	113	1037	0.11	[0.09; 0.13]
CenYongZhuang 2014[39]	32	106	0.30	[0.22; 0.40]
ZengFengMei 2020[41]	25	255	0.10	[0.06; 0.14]
ZengLei 2018[44]	28	388	0.07	[0.05; 0.10]
ChaRiSheng 2016[46]	22	453	0.05	[0.03; 0.07]
ChaRiSheng 2014[47−1]	36	170	0.21	[0.15; 0.28]
ChaRiSheng 2014[47−2]	29	498	0.06	[0.04; 0.08]
ChaRiSheng 2014[47−3]	43	594	0.07	[0.05; 0.10]
ChaRiSheng 2014[47−4]	18	1111	0.02	[0.01; 0.03]
ChaRiSheng 2014[47−5]	16	1168	0.01	[0.01; 0.02]
ChaRiSheng 2014[47−6]	31	1422	0.02	[0.01; 0.03]
ChaRiSheng 2014[47−7]	74	1749	0.04	[0.03; 0.05]
ChaRiSheng 2014[47−8]	47	1858	0.03	[0.02; 0.03]
ChaRiSheng 2014[47−9]	17	1910	0.01	[0.01; 0.01]
ChaRiSheng 2014[47−10]	168	2002	0.08	[0.07; 0.10]

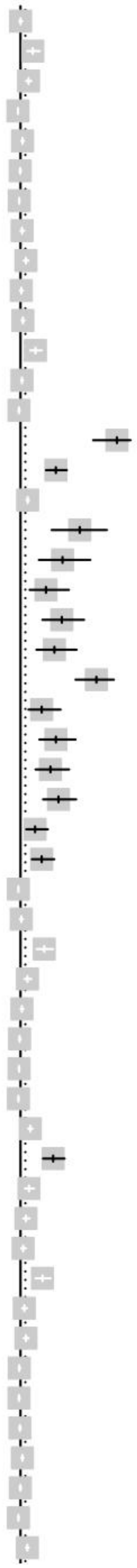

ChaRiSheng 2014[47−11]	61	2202	0.03 [0.02; 0.04]
ChaRiSheng 2014[47−12]	139	2786	0.05 [0.04; 0.06]
ChaRiSheng 2014[47−13]	39	3095	0.01 [0.01; 0.02]
ShenJiChuan 2011[50]	312	1736	0.18 [0.16; 0.20]
ShenYiPing 2018[52]	26	612	0.04 [0.03; 0.06]
ShenYiPing 2013[53]	65	1900	0.03 [0.03; 0.04]
ShenYuGang 2016[54]	43	480	0.09 [0.07; 0.12]
ShenYuGang 2016[55]	233	1702	0.14 [0.12; 0.15]
ChenAQun 2015[58]	64	19153	0.00 [0.00; 0.00]
ChenAQun 2016[59]	64	18924	0.00 [0.00; 0.00]
ChenBinBin 2017[60−1]	54	392	0.14 [0.11; 0.18]
ChenBinBin 2017[60−2]	161	2122	0.08 [0.06; 0.09]
ChenCaiRong 2020[62]	16	275	0.06 [0.03; 0.09]
ChenCan 2018[63]	24	61	0.39 [0.27; 0.53]
ChenChun 2014[64]	107	1242	0.09 [0.07; 0.10]
ChenGuoCui 2011[69]	27	34	0.79 [0.62; 0.91]
ChenHeJuan 2018[73]	14	49	0.29 [0.17; 0.43]
ChenJian 2016[78]	20	1551	0.01 [0.01; 0.02]
ChenJianMei 2017[79]	110	9736	0.01 [0.01; 0.01]
ChenJian 2017[80]	69	6323	0.01 [0.01; 0.01]
ChenJingFang 2018[84−1]	18	316	0.06 [0.03; 0.09]
ChenJingFang 2018[84−2]	14	360	0.04 [0.02; 0.06]
ChenMinHong 2017[92−1]	21	236	0.09 [0.06; 0.13]
ChenMinHong 2017[92−2]	186	657	0.28 [0.25; 0.32]
ChenMinHong 2017[92−3]	60	4286	0.01 [0.01; 0.02]
ChenQian 2014[95]	78	1844	0.04 [0.03; 0.05]
ChenQuan 2021[96]	84	821	0.10 [0.08; 0.13]
ChenXiaoFeng 2015[100]	7	648	0.01 [0.00; 0.02]
ChenXin 2020[101]	3	169	0.02 [0.00; 0.05]
ChenXingHong 2012[102]	478	7113	0.07 [0.06; 0.07]
ChenXingFu 2018[103]	43	1240	0.03 [0.03; 0.05]
ChenYan 2019[104]	59	536	0.11 [0.08; 0.14]
ChenYiXiong 2018[108]	21	1861	0.01 [0.01; 0.02]
ChenYiYi 2015[109]	282	38001	0.01 [0.01; 0.01]
ChenZhiQiong 2017[115]	255	1843	0.14 [0.12; 0.15]
ChuXiuJuan 2015[120]	17	147	0.12 [0.07; 0.18]
CuiLiangLiang 2017[123−1]	5	90	0.06 [0.02; 0.12]
CuiLiangLiang 2017[123−2]	14	4600	0.00 [0.00; 0.01]
CuiLiangLiang 2017[123−3]	55	10000	0.01 [0.00; 0.01]
CuiLiangLiang 2017[123−4]	1434	11000	0.13 [0.12; 0.14]
CuiLiangLiang 2017[123−5]	385	15000	0.03 [0.02; 0.03]
CuiLiangLiang 2017[123−6]	235	20000	0.01 [0.01; 0.01]
CuiXiaoMan 2015[124]	34	332	0.10 [0.07; 0.14]
CuiXiaoMan 2018[125]	17	829	0.02 [0.01; 0.03]
DaiBenNa 2020[127]	117	3119	0.04 [0.03; 0.04]
DaiYingXue 2018[129−1]	14	378	0.04 [0.02; 0.06]
DaiYingXue 2018[129−2]	12	547	0.02 [0.01; 0.04]
DaiYingXue 2018[129−3]	18	1008	0.02 [0.01; 0.03]
DaiYingXue 2018[129−4]	14	1053	0.01 [0.01; 0.02]
DengXingChao 2019[133]	439	42403	0.01 [0.01; 0.01]
DingJianQing 2012[138]	82	10177	0.01 [0.01; 0.01]
DongShengCao 2017[146]	24	200	0.12 [0.08; 0.17]
DuYueHe 2019[148]	29	1250	0.02 [0.02; 0.03]
DuanRong 2017[152−1]	13	77	0.17 [0.09; 0.27]
DuanRong 2017[152−2]	13	81	0.16 [0.09; 0.26]

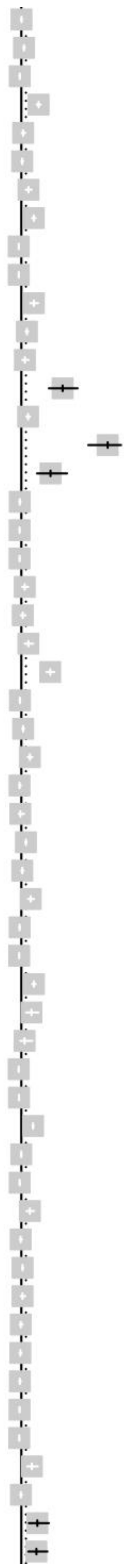

DuanRong 2017[152−3]	11	89	0.12 [0.06; 0.21]
DuanRong 2017[152−4]	9	93	0.10 [0.05; 0.18]
DuanRong 2017[152−5]	12	165	0.07 [0.04; 0.12]
DuanRong 2017[152−6]	11	179	0.06 [0.03; 0.11]
DuanRong 2017[152−7]	10	182	0.05 [0.03; 0.10]
DuanRong 2017[152−8]	9	199	0.05 [0.02; 0.08]
DuanRong 2017[152−9]	14	215	0.07 [0.04; 0.11]
DuanRong 2017[152−10]	36	227	0.16 [0.11; 0.21]
DuanRong 2017[152−11]	5	229	0.02 [0.01; 0.05]
DuanRong 2017[152−12]	8	238	0.03 [0.01; 0.07]
DuanRong 2017[152−13]	5	245	0.02 [0.01; 0.05]
DuanRong 2017[152−14]	8	249	0.03 [0.01; 0.06]
DuanRong 2017[152−15]	8	271	0.03 [0.01; 0.06]
DuanRong 2017[152−16]	7	441	0.02 [0.01; 0.03]
DuanRong 2017[152−17]	5	495	0.01 [0.00; 0.02]
DuanRong 2017[152−18]	9	538	0.02 [0.01; 0.03]
DuanRong 2017[152−19]	7	565	0.01 [0.00; 0.03]
DuanRong 2017[152−20]	5	590	0.01 [0.00; 0.02]
DuanRong 2017[152−21]	11	699	0.02 [0.01; 0.03]
DuanRong 2017[152−22]	7	701	0.01 [0.00; 0.02]
DuanRong 2017[152−23]	8	706	0.01 [0.00; 0.02]
DuanRong 2017[152−24]	7	733	0.01 [0.00; 0.02]
DuanRong 2017[152−25]	5	753	0.01 [0.00; 0.02]
DuanRong 2017[152−26]	24	802	0.03 [0.02; 0.04]
DuanRong 2017[152−27]	9	918	0.01 [0.00; 0.02]
DuanRong 2017[152−28]	14	927	0.02 [0.01; 0.03]
DuanRong 2017[152−29]	5	976	0.01 [0.00; 0.01]
DuanRong 2017[152−30]	5	976	0.01 [0.00; 0.01]
DuanRong 2017[152−31]	68	1036	0.07 [0.05; 0.08]
DuanRong 2017[152−32]	5	1145	0.00 [0.00; 0.01]
DuanRong 2017[152−33]	13	1216	0.01 [0.01; 0.02]
DuanRong 2017[152−34]	11	1420	0.01 [0.00; 0.01]
FangYunXia 2018[157]	27	357	0.08 [0.05; 0.11]
FengZhi 2020[161]	26	50	0.52 [0.37; 0.66]
FengZhi 2018[162]	111	2020	0.05 [0.05; 0.07]
FuJianGuang 2013[163]	276	1546	0.18 [0.16; 0.20]
FuXiaoFei 2012[171]	20	728	0.03 [0.02; 0.04]
GanXiangYang 2014[172]	74	2200	0.03 [0.03; 0.04]
GaoHaiMing 2014[174]	19	187	0.10 [0.06; 0.15]
GaoHuiJuan 2015[176]	18	51	0.35 [0.22; 0.50]
GaoJunYing 2017[177]	12	1501	0.01 [0.00; 0.01]
GaoPeng 2020[181]	28	217	0.13 [0.09; 0.18]
GaoRiHong 2019[182]	30	72	0.42 [0.30; 0.54]
GaoShuPing 2019[183]	51	1698	0.03 [0.02; 0.04]
GaoZhiPeng 2018[186]	130	397	0.33 [0.28; 0.38]
GaoZhiYong 2017[187−1]	12	32	0.38 [0.21; 0.56]
GaoZhiYong 2017[187−2]	10	46	0.22 [0.11; 0.36]
GaoZhiYong 2017[187−3]	15	57	0.26 [0.16; 0.40]
GaoZhiYong 2017[187−4]	16	63	0.25 [0.15; 0.38]
GaoZhiYong 2017[187−5]	7	78	0.09 [0.04; 0.18]
GaoZhiYong 2017[187−6]	25	953	0.03 [0.02; 0.04]
GaoZhiYong 2017[187−7]	21	980	0.02 [0.01; 0.03]
GaoZhiYong 2017[187−8]	22	1047	0.02 [0.01; 0.03]
GongLiQiang 2013[192]	139	2787	0.05 [0.04; 0.06]
GongShuiYing 2017[194]	93	990	0.09 [0.08; 0.11]

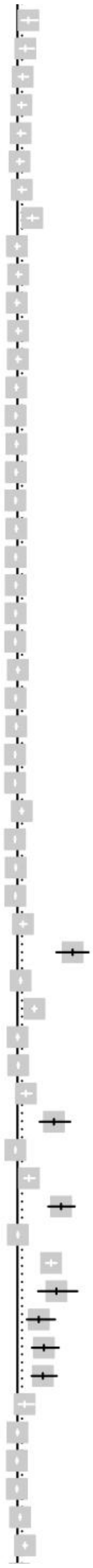

GuKaiChen 2020[195]	21	500	0.04 [0.03; 0.06]
GuShiPing 2009[196]	42	2199	0.02 [0.01; 0.03]
GuYiFu 2020[197]	45	1903	0.02 [0.02; 0.03]
GuiGuoPing 2018[200]	79	1204	0.07 [0.05; 0.08]
GuoJianXin 2015[202−1]	9	34	0.26 [0.13; 0.44]
GuoJianXin 2015[202−2]	20	43	0.47 [0.31; 0.62]
GuoJianXin 2015[202−3]	15	142	0.11 [0.06; 0.17]
GuoJianXin 2015[202−4]	22	172	0.13 [0.08; 0.19]
GuoJianXin 2015[202−5]	53	350	0.15 [0.12; 0.19]
GuoJianXin 2015[202−6]	55	611	0.09 [0.07; 0.12]
GuoLi 2019[205]	75	456	0.16 [0.13; 0.20]
GuoLi 2019[206−1]	7	12	0.58 [0.28; 0.85]
GuoLi 2019[206−2]	8	15	0.53 [0.27; 0.79]
GuoLi 2019[206−3]	9	22	0.41 [0.21; 0.64]
GuoLi 2019[206−4]	5	25	0.20 [0.07; 0.41]
GuoLi 2019[206−5]	6	26	0.23 [0.09; 0.44]
GuoLi 2019[206−6]	8	26	0.31 [0.14; 0.52]
GuoLi 2019[206−7]	8	27	0.30 [0.14; 0.50]
GuoLi 2019[206−8]	7	27	0.26 [0.11; 0.46]
GuoLi 2019[206−9]	8	28	0.29 [0.13; 0.49]
GuoLi 2019[206−10]	10	28	0.36 [0.19; 0.56]
GuoLi 2019[206−11]	11	28	0.39 [0.22; 0.59]
GuoLi 2019[206−12]	7	30	0.23 [0.10; 0.42]
GuoLi 2019[206−13]	22	30	0.73 [0.54; 0.88]
GuoLi 2019[206−14]	10	30	0.33 [0.17; 0.53]
GuoLi 2019[206−15]	9	31	0.29 [0.14; 0.48]
GuoLi 2019[206−16]	8	31	0.26 [0.12; 0.45]
GuoLi 2019[206−17]	10	31	0.32 [0.17; 0.51]
GuoLi 2019[206−18]	14	32	0.44 [0.26; 0.62]
GuoLi 2019[206−19]	9	33	0.27 [0.13; 0.46]
GuoLi 2019[206−20]	18	33	0.55 [0.36; 0.72]
GuoLi 2019[206−21]	8	34	0.24 [0.11; 0.41]
GuoLi 2019[206−22]	10	34	0.29 [0.15; 0.47]
GuoLi 2019[206−23]	14	34	0.41 [0.25; 0.59]
GuoLi 2019[206−24]	18	35	0.51 [0.34; 0.69]
GuoLi 2019[206−25]	16	35	0.46 [0.29; 0.63]
GuoLi 2019[206−26]	6	35	0.17 [0.07; 0.34]
GuoLi 2019[206−27]	17	36	0.47 [0.30; 0.65]
GuoLi 2019[206−28]	10	37	0.27 [0.14; 0.44]
GuoLi 2019[206−29]	14	38	0.37 [0.22; 0.54]
GuoLi 2019[206−30]	6	38	0.16 [0.06; 0.31]
GuoLi 2019[206−31]	12	39	0.31 [0.17; 0.48]
GuoLi 2019[206−32]	18	40	0.45 [0.29; 0.62]
GuoLi 2019[206−33]	16	40	0.40 [0.25; 0.57]
GuoLi 2019[206−34]	7	40	0.17 [0.07; 0.33]
GuoLi 2019[206−35]	7	41	0.17 [0.07; 0.32]
GuoLi 2019[206−36]	16	41	0.39 [0.24; 0.55]
GuoLi 2019[206−37]	10	41	0.24 [0.12; 0.40]
GuoLi 2019[206−38]	18	41	0.44 [0.28; 0.60]
GuoLi 2019[206−39]	9	41	0.22 [0.11; 0.38]
GuoLi 2019[206−40]	13	42	0.31 [0.18; 0.47]
GuoLi 2019[206−41]	5	42	0.12 [0.04; 0.26]
GuoLi 2019[206−42]	12	42	0.29 [0.16; 0.45]
GuoLi 2019[206−43]	16	43	0.37 [0.23; 0.53]
GuoLi 2019[206−44]	13	44	0.30 [0.17; 0.45]

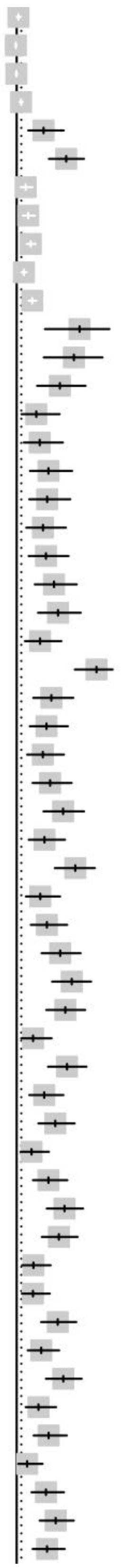

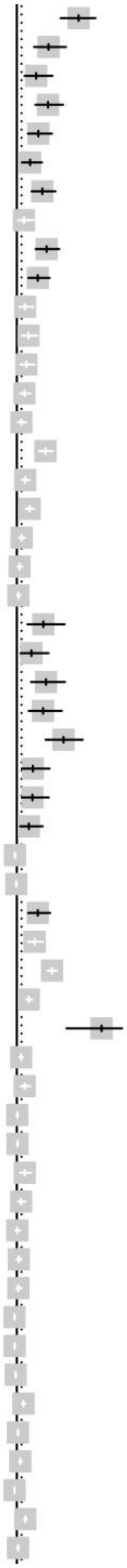

GuoLi 2019[206−45]	25	44	0.57 [0.41; 0.72]
GuoLi 2019[206−46]	14	46	0.30 [0.18; 0.46]
GuoLi 2019[206−47]	9	46	0.20 [0.09; 0.34]
GuoLi 2019[206−48]	18	60	0.30 [0.19; 0.43]
GuoLi 2019[206−49]	14	65	0.22 [0.12; 0.33]
GuoLi 2019[206−50]	10	70	0.14 [0.07; 0.25]
GuoLi 2019[206−51]	18	72	0.25 [0.16; 0.37]
GuoLi 2019[206−52]	7	79	0.09 [0.04; 0.17]
GuoLi 2019[206−53]	23	80	0.29 [0.19; 0.40]
GuoLi 2019[206−54]	17	81	0.21 [0.13; 0.31]
GuoLi 2019[206−55]	10	101	0.10 [0.05; 0.17]
GuoLi 2019[206−56]	13	101	0.13 [0.07; 0.21]
GuoLi 2019[206−57]	13	119	0.11 [0.06; 0.18]
GuoLi 2019[206−58]	16	171	0.09 [0.05; 0.15]
GuoLi 2019[206−59]	14	211	0.07 [0.04; 0.11]
GuoLi 2019[206−60]	61	220	0.28 [0.22; 0.34]
GuoLi 2019[206−61]	28	281	0.10 [0.07; 0.14]
GuoLi 2019[206−62]	47	339	0.14 [0.10; 0.18]
GuoLi 2019[206−63]	30	440	0.07 [0.05; 0.10]
GuoLi 2019[206−64]	25	498	0.05 [0.03; 0.07]
GuoLi 2019[206−65]	28	698	0.04 [0.03; 0.06]
GuoMinJian 2017[208−1]	8	31	0.26 [0.12; 0.45]
GuoMinJian 2017[208−2]	6	39	0.15 [0.06; 0.31]
GuoMinJian 2017[208−3]	11	39	0.28 [0.15; 0.45]
GuoMinJian 2017[208−4]	10	39	0.26 [0.13; 0.42]
GuoMinJian 2017[208−5]	17	39	0.44 [0.28; 0.60]
GuoMinJian 2017[208−6]	7	42	0.17 [0.07; 0.31]
GuoMinJian 2017[208−7]	7	43	0.16 [0.07; 0.31]
GuoMinJian 2017[208−8]	7	53	0.13 [0.05; 0.25]
GuoMinJian 2017[208−9]	16	1488	0.01 [0.01; 0.02]
GuoMinJian 2017[208−10]	48	2200	0.02 [0.02; 0.03]
GuoShuiLian 2014[209]	16	76	0.21 [0.13; 0.32]
HaoYongJian 2020[216]	25	137	0.18 [0.12; 0.26]
HeXuXin 2017[222]	156	466	0.33 [0.29; 0.38]
HeHanZhen 2014[224]	76	578	0.13 [0.11; 0.16]
HouWei 2018[230]	10	13	0.77 [0.46; 0.95]
HouYuYuan 2014[231]	63	984	0.06 [0.05; 0.08]
HuGuangYi 2017[232]	20	210	0.10 [0.06; 0.14]
HuHongAn 2015[233]	105	3453	0.03 [0.02; 0.04]
HuaWeiYu 2018[241]	63	1951	0.03 [0.02; 0.04]
HuaWeiYu 2018[242−1]	15	156	0.10 [0.05; 0.15]
HuaWeiYu 2018[242−2]	17	267	0.06 [0.04; 0.10]
HuaWeiYu 2018[242−3]	9	290	0.03 [0.01; 0.06]
HuaWeiYu 2018[242−4]	14	320	0.04 [0.02; 0.07]
HuaWeiYu 2018[242−5]	13	325	0.04 [0.02; 0.07]
HuaWeiYu 2018[242−6]	5	833	0.01 [0.00; 0.01]
HuaWeiYu 2018[242−7]	13	1806	0.01 [0.00; 0.01]
HuangBinBin 2020[245]	41	2530	0.02 [0.01; 0.02]
HuangChunLi 2015[246]	108	1283	0.08 [0.07; 0.10]
HuangGe 2013[251]	92	2527	0.04 [0.03; 0.04]
HuangGuo 2015[252]	69	1220	0.06 [0.04; 0.07]
HuangGuo 2015[253]	87	13856	0.01 [0.01; 0.01]
HuangLiQing 2020[254]	184	1823	0.10 [0.09; 0.12]
HuangShiTeng 2021[256]	61	1644	0.04 [0.03; 0.05]

HuangSiYue 2020[257]	90	19800	0.00	[0.00; 0.01]
HuangYanHong 2019[259−1]	20	29	0.69	[0.49; 0.85]
HuangYanHong 2019[259−2]	37	79	0.47	[0.36; 0.58]
HuangYanHong 2019[259−3]	27	115	0.23	[0.16; 0.32]
HuangYanHong 2019[259−4]	23	120	0.19	[0.13; 0.27]
HuangYanHong 2019[259−5]	24	120	0.20	[0.13; 0.28]
HuangYanHong 2019[259−6]	21	140	0.15	[0.10; 0.22]
HuangYanHong 2019[259−7]	31	146	0.21	[0.15; 0.29]
HuangYanHong 2019[259−8]	27	172	0.16	[0.11; 0.22]
HuangYanHong 2019[259−9]	30	176	0.17	[0.12; 0.23]
HuangYanHong 2019[259−10]	26	199	0.13	[0.09; 0.19]
HuangYanHong 2019[259−11]	32	456	0.07	[0.05; 0.10]
HuangYanHong 2019[259−12]	58	498	0.12	[0.09; 0.15]
HuangYanHong 2019[260]	84	760	0.11	[0.09; 0.14]
HuangYanHong 2019[261]	86	1190	0.07	[0.06; 0.09]
HuangZhongXue 2013[263]	22	92	0.24	[0.16; 0.34]
JiHong 2015[264]	78	3194	0.02	[0.02; 0.03]
JiJinHua 2018[265]	41	694	0.06	[0.04; 0.08]
JiLei 2020[270]	19	34	0.56	[0.38; 0.73]
Lei Ji 2011[271]	42	2210	0.02	[0.01; 0.03]
JiLei 2018[272−1]	7	55	0.13	[0.05; 0.24]
JiLei 2018[272−2]	16	92	0.17	[0.10; 0.27]
JiLei 2018[272−3]	18	191	0.09	[0.06; 0.14]
JiRuPing 2020[274]	119	2030	0.06	[0.05; 0.07]
JiaLiPing 2017[276]	12	30	0.40	[0.23; 0.59]
JiangXianChen 2014[283−1]	16	1485	0.01	[0.01; 0.02]
JiangXianChen 2014[283−2]	23	1877	0.01	[0.01; 0.02]
JiangYingCi 2015[284]	66	1904	0.03	[0.03; 0.04]
JiangChen 2019[285]	26	137	0.19	[0.13; 0.27]
JiangLie 2014[291]	15	590	0.03	[0.01; 0.04]
JiangXiHong 2019[293]	40	304	0.13	[0.10; 0.17]
JiangYiMei 2017[294]	15	3050	0.00	[0.00; 0.01]
JinXiaoFang 2015[302]	38	281	0.14	[0.10; 0.18]
KangQian 2020[303−1]	35	736	0.05	[0.03; 0.07]
KangQian 2020[303−2]	50	1622	0.03	[0.02; 0.04]
KuangHaoCheng 2016[307]	667	11300	0.06	[0.05; 0.06]
LaiShiMing 2014[308]	105	7113	0.01	[0.01; 0.02]
LeiYongLiang 2016[311]	46	984	0.05	[0.03; 0.06]
LiBing 2019[315]	67	2354	0.03	[0.02; 0.04]
LiBo 2013[316]	120	2303	0.05	[0.04; 0.06]
LiCaiYun 2012[318]	147	940	0.16	[0.13; 0.18]
LiChunLing 2020[319]	15	196	0.08	[0.04; 0.12]
LiDaiBo 2018[320]	18	56	0.32	[0.20; 0.46]
LiJiShan 2018[324−1]	6	22	0.27	[0.11; 0.50]
LiJiShan 2018[324−2]	7	40	0.17	[0.07; 0.33]
LiJiShan 2018[324−3]	7	78	0.09	[0.04; 0.18]
LiJiShan 2016[326−1]	16	35	0.46	[0.29; 0.63]
LiJiShan 2016[326−2]	18	41	0.44	[0.28; 0.60]
LiJiShan 2016[326−3]	8	46	0.17	[0.08; 0.31]
LiJianSen 2015[328]	70	268	0.26	[0.21; 0.32]
LiJie 2019[329]	106	2868	0.04	[0.03; 0.04]
LiJun 2012[334]	1252	2877	0.44	[0.42; 0.45]
LiMeng 2020[338−1]	7	74	0.09	[0.04; 0.19]
LiMeng 2020[338−2]	16	204	0.08	[0.05; 0.12]
LiMeng 2020[338−3]	7	260	0.03	[0.01; 0.05]

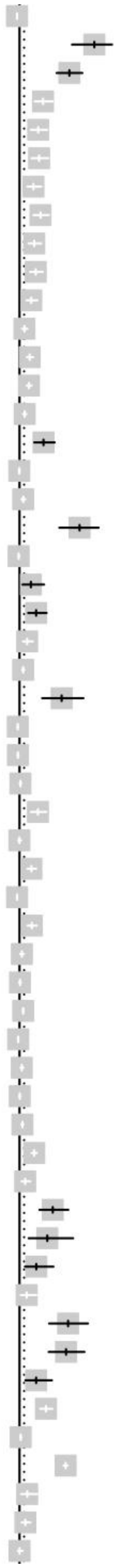

LiMeng 2020[338−4]	6	358	0.02	[0.01; 0.04]
LiMeng 2020[338−5]	6	369	0.02	[0.01; 0.04]
LiMeng 2020[338−6]	7	416	0.02	[0.01; 0.03]
LiMeng 2020[338−7]	11	476	0.02	[0.01; 0.04]
LiMeng 2020[338−8]	11	659	0.02	[0.01; 0.03]
LiMeng 2020[338−9]	12	749	0.02	[0.01; 0.03]
LiMeng 2020[338−10]	13	754	0.02	[0.01; 0.03]
LiMeng 2020[338−11]	19	1168	0.02	[0.01; 0.03]
LiMeng 2020[338−12]	9	1462	0.01	[0.00; 0.01]
LiMeng 2020[338−13]	17	1580	0.01	[0.01; 0.02]
LiMeng 2020[338−14]	85	1814	0.05	[0.04; 0.06]
LiMeng 2020[338−15]	6	1863	0.00	[0.00; 0.01]
LiMeng 2020[338−16]	17	2026	0.01	[0.00; 0.01]
LiMeng 2020[338−17]	5	2026	0.00	[0.00; 0.01]
LiQun 2010[340]	258	14269	0.02	[0.02; 0.02]
LiShiCong 2020[344]	181	3622	0.05	[0.04; 0.06]
LiShiCong 2019[345]	91	259	0.35	[0.29; 0.41]
LiShiCong 2018[346]	64	1487	0.04	[0.03; 0.05]
LiShiE 2018[347−1]	6	36	0.17	[0.06; 0.33]
LiShiE 2018[347−2]	15	38	0.39	[0.24; 0.57]
LiShiE 2018[347−3]	16	48	0.33	[0.20; 0.48]
LiShiE 2018[347−4]	22	66	0.33	[0.22; 0.46]
LiShiE 2018[347−5]	37	89	0.42	[0.31; 0.53]
LiShiE 2018[347−6]	14	94	0.15	[0.08; 0.24]
LiShiE 2018[347−7]	24	126	0.19	[0.13; 0.27]
LiShiE 2018[347−8]	34	173	0.20	[0.14; 0.26]
LiShiE 2018[347−9]	10	221	0.05	[0.02; 0.08]
LiShiE 2018[348]	92	1419	0.06	[0.05; 0.08]
LiShiE 2018[349]	77	8153	0.01	[0.01; 0.01]
LiShouJun 2016[350]	64	1023	0.06	[0.05; 0.08]
LiXiTai 2015[352]	51	1336	0.04	[0.03; 0.05]
LiXiuFang 2018[359]	20	378	0.05	[0.03; 0.08]
LiYan 2017[361]	93	2355	0.04	[0.03; 0.05]
LiYiLan 2014[365]	228	18930	0.01	[0.01; 0.01]
LiYueRong 2015[366]	18	952	0.02	[0.01; 0.03]
LiangPing 2020[374]	25	196	0.13	[0.08; 0.18]
LiangRiCheng 2017[376]	27	2518	0.01	[0.01; 0.02]
LiaoChan 2021[380]	19	675	0.03	[0.02; 0.04]
LiaoKeChang 2018[381]	41	161	0.25	[0.19; 0.33]
LinJian 2019[384]	77	2476	0.03	[0.02; 0.04]
LinQiFeng 2018[388]	96	1494	0.06	[0.05; 0.08]
LinQin 2015[390]	34	713	0.05	[0.03; 0.07]
LinQingShuang 2016[391]	29	603	0.05	[0.03; 0.07]
LinYanYan 2018[393]	76	3288	0.02	[0.02; 0.03]
LiuBaiWei 2017[395]	24	471	0.05	[0.03; 0.07]
LiuBaiWei 2017[396−1]	43	222	0.19	[0.14; 0.25]
LiuBaiWei 2017[396−2]	172	3546	0.05	[0.04; 0.06]
LiuBaiWei 2017[396−3]	58	4500	0.01	[0.01; 0.02]
LiuBo 2015[398]	104	1883	0.06	[0.05; 0.07]
LiuCaiXia 2019[399−1]	8	9	0.89	[0.52; 1.00]
LiuCaiXia 2019[399−2]	7	9	0.78	[0.40; 0.97]
LiuCaiXia 2019[399−3]	20	208	0.10	[0.06; 0.14]
LiuDan 2019[403]	90	951	0.09	[0.08; 0.12]
LiuDongSheng 2019[404]	80	3720	0.02	[0.02; 0.03]
LiuGuoHong 2014[406]	109	636	0.17	[0.14; 0.20]

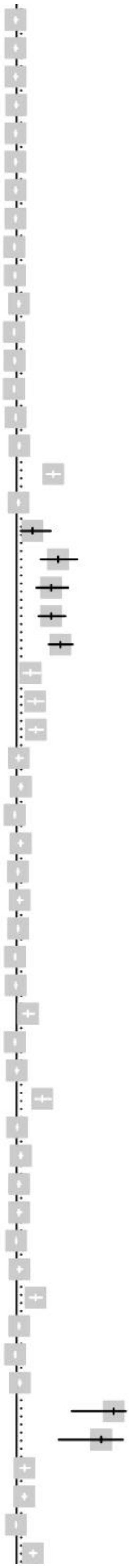

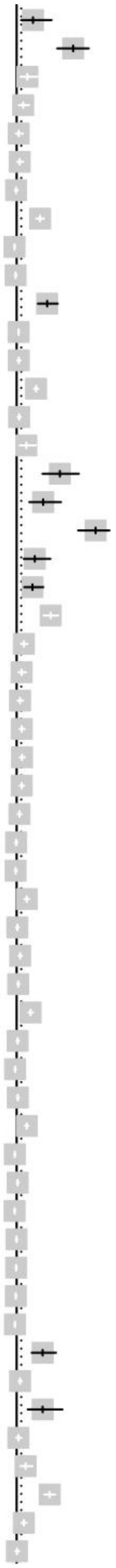

LiuHaoHui 2020[410−1]	6	35	0.17	[0.07; 0.34]
LiuHaoHui 2020[410−2]	28	53	0.53	[0.39; 0.67]
LiuHaoHui 2020[410−3]	10	82	0.12	[0.06; 0.21]
LiuHaoHui 2020[410−4]	14	166	0.08	[0.05; 0.14]
LiuHaoHui 2020[410−5]	9	202	0.04	[0.02; 0.08]
LiuHaoHui 2020[410−6]	16	300	0.05	[0.03; 0.09]
LiuHaoHui 2020[410−7]	12	561	0.02	[0.01; 0.04]
LiuHaoHui 2020[410−8]	145	618	0.23	[0.20; 0.27]
LiuHaoHui 2020[410−9]	17	2191	0.01	[0.00; 0.01]
LiuHongLian 2018[411]	19	1119	0.02	[0.01; 0.03]
LiuJingJing 2018[413]	33	111	0.30	[0.21; 0.39]
LiuKaiQian 2011[415]	634	14439	0.04	[0.04; 0.05]
LiuQingLian 2019[420]	37	817	0.05	[0.03; 0.06]
LiuShiKe 2012[421]	306	1539	0.20	[0.18; 0.22]
LiuShiKe 2016[422]	46	948	0.05	[0.04; 0.06]
LiuTian 2019[423]	10	89	0.11	[0.06; 0.20]
LiuTian 2017[424−1]	16	39	0.41	[0.26; 0.58]
LiuTian 2017[424−2]	11	42	0.26	[0.14; 0.42]
LiuTian 2017[424−3]	32	44	0.73	[0.57; 0.85]
LiuTian 2017[424−4]	9	48	0.19	[0.09; 0.33]
LiuTian 2017[424−5]	14	84	0.17	[0.09; 0.26]
LiuTian 2017[424−6]	70	213	0.33	[0.27; 0.40]
LiuTian 2017[424−7]	35	387	0.09	[0.06; 0.12]
LiuWenJun 2018[428−1]	18	255	0.07	[0.04; 0.11]
LiuWenJun 2018[428−2]	22	388	0.06	[0.04; 0.08]
LiuWenJun 2018[428−3]	38	528	0.07	[0.05; 0.10]
LiuWenJun 2018[428−4]	42	565	0.07	[0.05; 0.10]
LiuWenJun 2018[428−5]	48	672	0.07	[0.05; 0.09]
LiuWenJun 2018[428−6]	36	700	0.05	[0.04; 0.07]
LiuWenJun 2018[428−7]	18	826	0.02	[0.01; 0.03]
LiuWenJun 2018[428−8]	16	904	0.02	[0.01; 0.03]
LiuWenJun 2018[428−9]	105	907	0.12	[0.10; 0.14]
LiuWenJun 2018[428−10]	31	951	0.03	[0.02; 0.05]
LiuWenJun 2018[428−11]	55	955	0.06	[0.04; 0.07]
LiuWenJun 2018[428−12]	49	1213	0.04	[0.03; 0.05]
LiuWenJun 2018[428−13]	193	1285	0.15	[0.13; 0.17]
LiuWenJun 2018[428−14]	46	1314	0.04	[0.03; 0.05]
LiuWenJun 2018[428−15]	17	1328	0.01	[0.01; 0.02]
LiuWenJun 2018[428−16]	54	1467	0.04	[0.03; 0.05]
LiuWenJun 2018[428−17]	180	1554	0.12	[0.10; 0.13]
LiuWenJun 2018[428−18]	19	1776	0.01	[0.01; 0.02]
LiuWenJun 2018[428−19]	68	1926	0.04	[0.03; 0.04]
LiuWenJun 2018[428−20]	17	1932	0.01	[0.01; 0.01]
LiuWenJun 2018[428−21]	55	2174	0.03	[0.02; 0.03]
LiuWenJun 2018[428−22]	52	2332	0.02	[0.02; 0.03]
LiuWenJun 2018[428−23]	70	3608	0.02	[0.02; 0.02]
LiuWenJun 2018[428−24]	43	3707	0.01	[0.01; 0.02]
LiuXiaoXiao 2014[430]	18	70	0.26	[0.16; 0.38]
LiuXiuMei 2017[433]	65	1134	0.06	[0.04; 0.07]
LiuYi 2013[436]	9	35	0.26	[0.12; 0.43]
LiuYing 2019[437]	14	335	0.04	[0.02; 0.07]
LiuYuan 2016[438−1]	15	142	0.11	[0.06; 0.17]
LiuYuan 2016[438−2]	121	378	0.32	[0.27; 0.37]
LiuYuan 2016[438−3]	55	611	0.09	[0.07; 0.12]
LongJunBiao 2014[441]	13	444	0.03	[0.02; 0.05]

LongQiZhi 2016[442]	101	1707	0.06 [0.05; 0.07]
LuZhengXiang 2016[451]	83	4624	0.02 [0.01; 0.02]
LuHua 2016[454]	14	213	0.07 [0.04; 0.11]
LuJianYong 2015[456]	60	973	0.06 [0.05; 0.08]
LuCaiFang 2020[458]	19	499	0.04 [0.02; 0.06]
LuWeiWei 2016[462]	406	3808	0.11 [0.10; 0.12]
LuXiuZhi 2018[463]	47	3600	0.01 [0.01; 0.02]
LuoGuiHe 2013[467]	10	422	0.02 [0.01; 0.04]
LuoLe 2017[472]	39	1192	0.03 [0.02; 0.04]
LuoQiong 2019[474]	22	2256	0.01 [0.01; 0.01]
LuoTengXian 2018[475]	13	200	0.06 [0.04; 0.11]
MaMengMeng 2018[484]	223	30711	0.01 [0.01; 0.01]
MaTao 2015[486]	84	901	0.09 [0.08; 0.11]
MaTao 2018[487]	46	587	0.08 [0.06; 0.10]
MaoJianYing 2016[493]	51	559	0.09 [0.07; 0.12]
MengJian 2018[495]	37	465	0.08 [0.06; 0.11]
MengXiangJie 2012[499]	16	34	0.47 [0.30; 0.65]
MoGuiQiong 2016[504]	26	843	0.03 [0.02; 0.04]
MoYuJie 2018[505]	19	1117	0.02 [0.01; 0.03]
MiaoGuoZhong 2018[507]	25	350	0.07 [0.05; 0.10]
NiChaoRong 2019[510]	125	3622	0.03 [0.03; 0.04]
NiChaoRong 2019[511]	28	2158	0.01 [0.01; 0.02]
NiChunYan 2020[512]	55	491	0.11 [0.09; 0.14]
OuSheXiang 2019[515]	18	603	0.03 [0.02; 0.05]
PanYiFeng 2017[521]	82	7396	0.01 [0.01; 0.01]
PanYueFei 2020[522]	45	215	0.21 [0.16; 0.27]
PangZhiFeng 2017[524]	79	1696	0.05 [0.04; 0.06]
PangZhiFeng 2014[525]	20	548	0.04 [0.02; 0.06]
PangZhiMing 2015[526]	19	381	0.05 [0.03; 0.08]
PengXiaoXue 2015[528]	37	849	0.04 [0.03; 0.06]
PuPeiLong 2017[529]	98	1126	0.09 [0.07; 0.11]
QiYanQiu 2018[532]	27	570	0.05 [0.03; 0.07]
QiXiaoQi 2019[534]	185	2418	0.08 [0.07; 0.09]
QiYing 2018[535−1]	173	1621	0.11 [0.09; 0.12]
QiYing 2018[535−2]	71	1621	0.04 [0.03; 0.05]
QiYing 2019[535−3]	16	38	0.42 [0.26; 0.59]
QiYing 2019[535−4]	17	39	0.44 [0.28; 0.60]
QiYing 2019[535−5]	13	40	0.32 [0.19; 0.49]
QiYing 2019[535−6]	8	83	0.10 [0.04; 0.18]
QiYing 2019[535−7]	8	129	0.06 [0.03; 0.12]
QiYing 2019[535−8]	10	148	0.07 [0.03; 0.12]
QiYing 2019[535−9]	34	1015	0.03 [0.02; 0.05]
QiYing 2019[535−10]	72	1192	0.06 [0.05; 0.08]
QiYing 2019[535−11]	159	1448	0.11 [0.09; 0.13]
QianLiZhen 2020[537]	23	95	0.24 [0.16; 0.34]
QianZiYu 2012[539]	22	111	0.20 [0.13; 0.28]
QinDi 2016[544]	16	255	0.06 [0.04; 0.10]
QinLianYang 2021[545]	38	1697	0.02 [0.02; 0.03]
QinMeng 2015[547−1]	35	217	0.16 [0.11; 0.22]
QinMeng 2015[547−2]	25	228	0.11 [0.07; 0.16]
QinTianXiu 2013[548]	9	17	0.53 [0.28; 0.77]
QinYanMin 2010[549]	97	1031	0.09 [0.08; 0.11]
QiuHaiYan 2013[551]	76	5965	0.01 [0.01; 0.02]
RenFuLin 2013[554]	43	594	0.07 [0.05; 0.10]
RenFuLin 2013[555]	74	1751	0.04 [0.03; 0.05]

RenLiJun 2020[556]	9	253	0.04	[0.02; 0.07]
RenQiZhi 2018[557−1]	6	100	0.06	[0.02; 0.13]
RenQiZhi 2018[557−2]	12	150	0.08	[0.04; 0.14]
RenQiZhi 2018[557−3]	8	151	0.05	[0.02; 0.10]
RenQiZhi 2018[557−4]	15	200	0.07	[0.04; 0.12]
RenQiZhi 2018[557−5]	5	2000	0.00	[0.00; 0.01]
RenQiZhi 2018[557−6]	13	2500	0.01	[0.00; 0.01]
RenQiZhi 2018[557−7]	15	10000	0.00	[0.00; 0.00]
RenYuHua 2016[562]	110	1916	0.06	[0.05; 0.07]
RuiFang 2018[566−1]	18	180	0.10	[0.06; 0.15]
RuiFang 2018[566−2]	18	183	0.10	[0.06; 0.15]
RuiFang 2018[566−3]	11	263	0.04	[0.02; 0.07]
RuiFang 2018[566−4]	5	382	0.01	[0.00; 0.03]
RuiFang 2018[566−5]	18	473	0.04	[0.02; 0.06]
RuiFang 2018[566−6]	16	670	0.02	[0.01; 0.04]
RuiFang 2018[566−7]	54	908	0.06	[0.04; 0.08]
RuiFang 2018[566−8]	8	1059	0.01	[0.00; 0.01]
RuiFang 2018[566−9]	78	1184	0.07	[0.05; 0.08]
RuiFang 2018[566−10]	10	2084	0.00	[0.00; 0.01]
RuiFang 2018[566−11]	15	2905	0.01	[0.00; 0.01]
Wu−Yang Shi 2020[577−1]	35	441	0.08	[0.06; 0.11]
ShiChao 2013[579]	462	2024	0.23	[0.21; 0.25]
ShuaiHuiQun 2012[589]	14	569	0.02	[0.01; 0.04]
SongCanLei 2020[590]	44	812	0.05	[0.04; 0.07]
SongCanLei 2012[591]	18	385	0.05	[0.03; 0.07]
SongCanLei 2013[592−1]	9	25	0.36	[0.18; 0.57]
SongCanLei 2013[592−2]	8	161	0.05	[0.02; 0.10]
SongCanLei 2017[593]	36	888	0.04	[0.03; 0.06]
SongCanLei 2013[594]	26	162	0.16	[0.11; 0.23]
SongCanLei 2013[595]	11	71	0.15	[0.08; 0.26]
SongHuiRong 2017[597]	34	568	0.06	[0.04; 0.08]
SongJianQiang 2015[598]	259	10942	0.02	[0.02; 0.03]
SongJie 2014[599]	17	350	0.05	[0.03; 0.08]
SongYuFang 2019[600]	63	783	0.08	[0.06; 0.10]
SuTong 2020[604]	55	3432	0.02	[0.01; 0.02]
SunHaiBo 2012[608]	66	100177	0.00	[0.00; 0.00]
SunJing 2015[611]	92	8929	0.01	[0.01; 0.01]
SunLiMei 2012[612]	108	5854	0.02	[0.02; 0.02]
SunLiYan 2020[613]	11	51	0.22	[0.11; 0.35]
SunMingHua 2017[614]	36	496	0.07	[0.05; 0.10]
SunQin 2019[616]	32	128	0.25	[0.18; 0.33]
SunWenLong 2018[618]	8	142	0.06	[0.02; 0.11]
SunYunLan 2018[624]	63	2212	0.03	[0.02; 0.04]
SunZhou 2016[625]	230	45075	0.01	[0.00; 0.01]
TanDongMei 2012[627]	87	2086	0.04	[0.03; 0.05]
TangYuHuan 2017[630]	44	2494	0.02	[0.01; 0.02]
TangGuoJie 2019[631]	84	2250	0.04	[0.03; 0.05]
TangXuLi 2013[633]	23	116	0.20	[0.13; 0.28]
TangYuXin 2014[634]	97	994	0.10	[0.08; 0.12]
TaoLiYan 2020[636]	47	1857	0.03	[0.02; 0.03]
TianJing 2017[641]	39	392	0.10	[0.07; 0.13]
TianYaLin 2021[642]	38	546	0.07	[0.05; 0.09]
WangJinSheng 2019[645]	56	1800	0.03	[0.02; 0.04]
WangJinSheng 2016[646]	99	11594	0.01	[0.01; 0.01]

WangBing 2017[649]	58	20000	0.00 [0.00; 0.00]
WangDaHu 2020[652]	182	33479	0.01 [0.00; 0.01]
WangHu 2018[656]	72	28270	0.00 [0.00; 0.00]
WangHua 2014[657]	28	497	0.06 [0.04; 0.08]
WangJie 2015[659]	451	11467	0.04 [0.04; 0.04]
WangJun 2012[663]	17	400	0.04 [0.02; 0.07]
WangJun 2021[664]	20	4743	0.00 [0.00; 0.01]
WangKaiLiang 2019[665]	16	446	0.04 [0.02; 0.06]
Kun−Ming Wang 2017[666]	5	6	0.83 [0.36; 1.00]
WangMan 2017[668]	85	540	0.16 [0.13; 0.19]
WangMeiHuan 2020[669]	12	433	0.03 [0.01; 0.05]
WangMin 2018[670−1]	19	180	0.11 [0.06; 0.16]
WangMin 2018[670−2]	11	599	0.02 [0.01; 0.03]
WangMin 2018[670−3]	37	672	0.06 [0.04; 0.08]
WangMin 2018[670−4]	27	861	0.03 [0.02; 0.05]
WangMin 2018[670−5]	32	2843	0.01 [0.01; 0.02]
WangMin 2018[670−6]	47	2869	0.02 [0.01; 0.02]
WangMingLiang 2020[671]	107	550	0.19 [0.16; 0.23]
WangShuangYing 2016[676]	16	119	0.13 [0.08; 0.21]
WangTianYu 2018[677]	8	16	0.50 [0.25; 0.75]
WangTieJun 2020[678]	15	345	0.04 [0.02; 0.07]
WangXiaoDong 2017[688]	69	2708	0.03 [0.02; 0.03]
WangXiaoQin 2015[689]	11	142	0.08 [0.04; 0.13]
WeiYiYun 2015[705]	22	155	0.14 [0.09; 0.21]
WeiGuiYing 2019[706]	29	257	0.11 [0.08; 0.16]
WeiXia 2019[708]	22	1024	0.02 [0.01; 0.03]
WenYa 2018[710]	16	668	0.02 [0.01; 0.04]
WenYingMing 2019[711]	15	35	0.43 [0.26; 0.61]
WuGuoFu 2018[717]	17	506	0.03 [0.02; 0.05]
WuHongXing 2016[718]	63	726	0.09 [0.07; 0.11]
WuJingWen 2020[719]	34	1153	0.03 [0.02; 0.04]
WuMingXiong 2018[723]	10	1292	0.01 [0.00; 0.01]
WuQinDi 2018[725]	24	1578	0.02 [0.01; 0.02]
WuShuiXin 2012[727]	25	1376	0.02 [0.01; 0.03]
WuWenQian 2018[729]	36	2399	0.02 [0.01; 0.02]
WuXiaYan 2015[730]	92	2046	0.04 [0.04; 0.05]
WuXiaoMin 2021[732]	159	1378	0.12 [0.10; 0.13]
WuYang 2018[733]	85	265	0.32 [0.26; 0.38]
WuYang 2018[734]	26	2179	0.01 [0.01; 0.02]
WuYang 2018[735]	91	516	0.18 [0.14; 0.21]
WuYiLing 2017[736]	31	1311	0.02 [0.02; 0.03]
WuZhaoChun 2015[737]	17	130	0.13 [0.08; 0.20]
WuZhenYu 2012[738]	19	57	0.33 [0.21; 0.47]
WuZhiSheng 2014[741]	176	14612	0.01 [0.01; 0.01]
XiaGuangHui 2019[742]	106	2300	0.05 [0.04; 0.06]
XiaYingPin 2018[743]	177	1777	0.10 [0.09; 0.11]
XiaZhongFa 2012[744]	209	1616	0.13 [0.11; 0.15]
XiaoSongJian 2017[745]	51	416	0.12 [0.09; 0.16]
XiaoDaYong 2018[746]	47	154	0.31 [0.23; 0.38]
XieCaiWen 2017[749]	12	58	0.21 [0.11; 0.33]
XieHuaPing 2010[752]	55	1638	0.03 [0.03; 0.04]
XieYuanQi 2016[756]	34	207	0.16 [0.12; 0.22]
XieBin 2018[757]	95	5000	0.02 [0.02; 0.02]
XieBin 2020[758]	65	2036	0.03 [0.02; 0.04]
XingYan 2017[759]	119	3500	0.03 [0.03; 0.04]

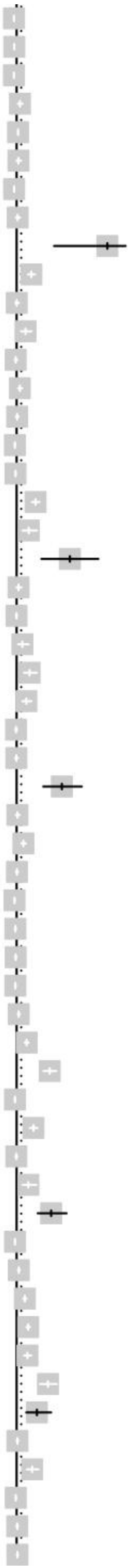

XuJianRong 2018[767]	92	2046	0.04 [0.04; 0.05]
XuShiMin 2013[771]	37	116	0.32 [0.24; 0.41]
XuYan 2018[772]	68	969	0.07 [0.05; 0.09]
XuFeng 2012[774]	22	79	0.28 [0.18; 0.39]
XuHao 2016[775]	28	2385	0.01 [0.01; 0.02]
XuJinFeng 2016[778−1]	10	933	0.01 [0.01; 0.02]
XuJinFeng 2016[778−2]	16	2500	0.01 [0.00; 0.01]
XuJun 2018[779]	75	2679	0.03 [0.02; 0.03]
XuanLingFeng 2014[784]	17	9523	0.00 [0.00; 0.00]
YanGeBin 2017[786−1]	14	35	0.40 [0.24; 0.58]
YanGeBin 2017[786−2]	25	62	0.40 [0.28; 0.54]
YanChaoYang 2017[792]	90	2168	0.04 [0.03; 0.05]
YangAiQing 2018[795]	40	355	0.11 [0.08; 0.15]
YangCaiBin 2018[796]	42	2159	0.02 [0.01; 0.03]
YangCheng 2017[797]	109	330	0.33 [0.28; 0.38]
YangGenMei 2015[798]	54	392	0.14 [0.11; 0.18]
YangJiXing 2019[801]	30	808	0.04 [0.03; 0.05]
YangJiChao 2016[802]	18	137	0.13 [0.08; 0.20]
YangJing 2018[804]	111	1718	0.06 [0.05; 0.08]
YangRongXing 2019[808]	15	334	0.04 [0.03; 0.07]
YangSenPing 2018[810]	30	305	0.10 [0.07; 0.14]
YangShiYong 2015[811]	28	144	0.19 [0.13; 0.27]
YangTongTong 2018[813]	36	450	0.08 [0.06; 0.11]
YangYiLong 2014[818]	121	18597	0.01 [0.01; 0.01]
YangYouQing 2016[819]	48	2363	0.02 [0.02; 0.03]
YangZhiWen 2013[820]	11	48	0.23 [0.12; 0.37]
YaoJianXiang 2016[822]	59	2992	0.02 [0.02; 0.03]
YaoJing 2017[824]	179	885	0.20 [0.18; 0.23]
Jin−Bo Ye 2018[830−1]	25	136	0.18 [0.12; 0.26]
Jin−Bo Ye 2018[830−2]	14	178	0.08 [0.04; 0.13]
Jin−Bo Ye 2018[830−3]	11	227	0.05 [0.02; 0.09]
Jin−Bo Ye 2018[830−4]	73	231	0.32 [0.26; 0.38]
Jin−Bo Ye 2018[830−5]	90	255	0.35 [0.29; 0.42]
Jin−Bo Ye 2018[830−6]	15	259	0.06 [0.03; 0.09]
Jin−Bo Ye 2018[830−7]	81	295	0.27 [0.22; 0.33]
Jin−Bo Ye 2018[830−8]	49	760	0.06 [0.05; 0.08]
Jin−Bo Ye 2018[830−9]	40	1125	0.04 [0.03; 0.05]
Jin−Bo Ye 2018[830−10]	55	1377	0.04 [0.03; 0.05]
Jin−Bo Ye 2018[830−11]	101	1784	0.06 [0.05; 0.07]
Jin−Bo Ye 2018[830−12]	77	2034	0.04 [0.03; 0.05]
Jin−Bo Ye 2018[830−13]	60	2572	0.02 [0.02; 0.03]
Jin−Bo Ye 2018[830−14]	132	2584	0.05 [0.04; 0.06]
YeShuJun 2019[831]	104	399	0.26 [0.22; 0.31]
YeXianMing 2017[832]	94	1156	0.08 [0.07; 0.10]
YeXiaoLing 2014[833]	27	264	0.10 [0.07; 0.15]
YinHongMei 2014[839]	37	511	0.07 [0.05; 0.10]
YingLiHong 2016[840]	48	3000	0.02 [0.01; 0.02]
YuXiaoYun 2018[845]	78	1007	0.08 [0.06; 0.10]
YuHong 2016[848]	80	1184	0.07 [0.05; 0.08]
YuZhuXian 2013[849]	13	357	0.04 [0.02; 0.06]
YuKuangMing 2015[851]	16	57	0.28 [0.17; 0.42]
YuanJun 2014[854]	652	115469	0.01 [0.01; 0.01]
YueYong 2017[859]	12	150	0.08 [0.04; 0.14]
ZhanYueWang 2019[860]	38	2311	0.02 [0.01; 0.02]
ZhangAiHua 2018[861]	19	214	0.09 [0.05; 0.14]

ZhangChong 2015[862]	13	2050	0.01	[0.00; 0.01]
ZhangDianXiang 2012[863]	5	40	0.12	[0.04; 0.27]
ZhangDongSheng 2013[864]	97	6614	0.01	[0.01; 0.02]
ZhangHaiYan 2019[867]	145	738	0.20	[0.17; 0.23]
ZhangHaiYan 2016[868]	78	508	0.15	[0.12; 0.19]
ZhangHengQiu 2014[869]	18	250	0.07	[0.04; 0.11]
ZhangHuiLing 2018[871]	40	100	0.40	[0.30; 0.50]
ZhangLi 2015[875−1]	33	474	0.07	[0.05; 0.10]
ZhangLi 2015[875−2]	72	656	0.11	[0.09; 0.14]
ZhangLing 2016[879]	132	735	0.18	[0.15; 0.21]
ZhangMeiMei 2013[880]	126	8154	0.02	[0.01; 0.02]
ZhangNing 2018[882]	31	340	0.09	[0.06; 0.13]
ZhangPan 2020[883]	16	244	0.07	[0.04; 0.10]
ZhangQin 2020[885]	57	4279	0.01	[0.01; 0.02]
ZhangShuang 2017[888]	28	45	0.62	[0.47; 0.76]
ZhangXiaoYi 2015[891]	21	57	0.37	[0.24; 0.51]
ZhangYan 2017[894−1]	8	37	0.22	[0.10; 0.38]
ZhangYan 2017[894−2]	12	2857	0.00	[0.00; 0.01]
ZhangYanMing 2019[898]	15	236	0.06	[0.04; 0.10]
ZhangZhen 2015[903]	76	414	0.18	[0.15; 0.22]
ZhangZhengDong 2017[904]	25	466	0.05	[0.04; 0.08]
ZhangZhiZhong 2020[906]	263	17752	0.01	[0.01; 0.02]
ZhangGuangMing 2018[907]	14	149	0.09	[0.05; 0.15]
ZhangGuoBao 2020[908]	13	105	0.12	[0.07; 0.20]
ZhangQingHe 2020[909]	109	538	0.20	[0.17; 0.24]
ZhaoJun 2014[916]	26	287	0.09	[0.06; 0.13]
ZhaoMeiLing 2019[918−1]	8	842	0.01	[0.00; 0.02]
ZhaoMeiLing 2019[918−2]	39	3305	0.01	[0.01; 0.02]
ZhaoMengJiao 2018[919]	87	263	0.33	[0.27; 0.39]
ZhaoQi 2021[920]	53	4760	0.01	[0.01; 0.01]
ZhaoTengLong 2015[921]	25	564	0.04	[0.03; 0.06]
ZhaoWeiQin 2020[923]	128	4927	0.03	[0.02; 0.03]
ZhaoWenNa 2021[924]	70	3000	0.02	[0.02; 0.03]
ZhaoXueCheng 2016[929]	149	1570	0.09	[0.08; 0.11]
ZhaoYiNan 2018[932]	67	3000	0.02	[0.02; 0.03]
ZhaoYuLi 2019[933]	119	1784	0.07	[0.06; 0.08]
ZhenGuoXin 2020[936]	23	204	0.11	[0.07; 0.16]
ZhenGuoXin 2020[937]	11	204	0.05	[0.03; 0.09]
HuangJiaCheng 2021[944]	48	5682	0.01	[0.01; 0.01]
ZhongJianMing 2014[946]	11	1208	0.01	[0.00; 0.02]
ZhongWenLong 2012[948]	10	69	0.14	[0.07; 0.25]
ZhouAiHua 2017[950]	80	650	0.12	[0.10; 0.15]
ZhouGuoYing 2016[952]	12	75	0.16	[0.09; 0.26]
ZhouJianHong 2013[954]	15	636	0.02	[0.01; 0.04]
ZhouLiHong 2016[956]	13	37	0.35	[0.20; 0.53]
ZhouXiaoHong 2015[958]	107	7113	0.02	[0.01; 0.02]
ZhouXiaoTao 2011[959]	96	1045	0.09	[0.08; 0.11]
ZhouXiaoHong 2010[960]	37	295	0.13	[0.09; 0.17]
ZhouXiaoHong 2017[961]	47	3464	0.01	[0.01; 0.02]
ZhouXiaoMin 2017[962]	47	3917	0.01	[0.01; 0.02]
ZhouYin 2016[964]	12	1061	0.01	[0.01; 0.02]
ZhouZhenQing 2016[966]	28	186	0.15	[0.10; 0.21]
ZhuChengMing 2017[967]	78	4454	0.02	[0.01; 0.02]
ZhuChunXiu 2019[968]	17	401	0.04	[0.02; 0.07]

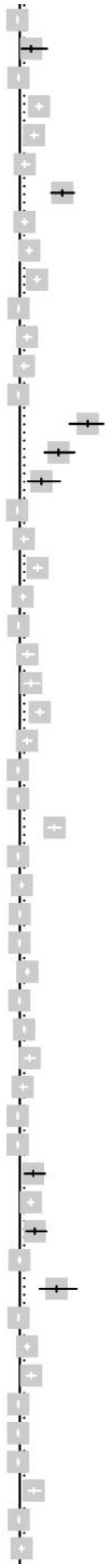

ZhuHaiYang 2016[971]	140	1319	0.11 [0.09; 0.12]
ZhuWeiWei 2018[976]	91	9702	0.01 [0.01; 0.01]
ZongJun 2017[980]	45	78	0.58 [0.46; 0.69]
Chen D 2019[987]	209	5043	0.04 [0.04; 0.05]
HuangKaiXiong 2021[989]	36	660	0.05 [0.04; 0.07]
LiXiaoJing 2021[992]	7	251	0.03 [0.01; 0.06]
ShenJingQiang 2020[993]	15	37	0.41 [0.25; 0.58]
Cui C 2017[994]	34	400	0.08 [0.06; 0.12]
Xiao GD 2019[995]	12	302	0.04 [0.02; 0.07]
Guo XH 2018[1000]	61	1606	0.04 [0.03; 0.05]
Guo Z 2014[1001]	87	7141	0.01 [0.01; 0.02]
Huang J 2013[1006−1]	5	22	0.23 [0.08; 0.45]
Huang J 2013[1006−2]	5	25	0.20 [0.07; 0.41]
Huang J 2013[1006−3]	6	33	0.18 [0.07; 0.35]
Huang J 2013[1006−4]	6	38	0.16 [0.06; 0.31]
Huang J 2013[1006−5]	14	103	0.14 [0.08; 0.22]
Huang XY 2017[1007]	753	22861	0.03 [0.03; 0.04]
Huang Z 2020[1008]	68	786	0.09 [0.07; 0.11]
S Kim 2019[1012]	15	48	0.31 [0.19; 0.46]
Li J 2018[1015]	19	1327	0.01 [0.01; 0.02]
Y Li 2021[1018]	143	346	0.41 [0.36; 0.47]
Y Li 2013[1019]	396	14387	0.03 [0.02; 0.03]
Liu D 2020[1021]	20	23	0.87 [0.66; 0.97]
Liu Y 2015[1025]	99	2049	0.05 [0.04; 0.06]
Lu Y 2020[1029]	157	785	0.20 [0.17; 0.23]
Luo LF 2015[1030]	32	45	0.71 [0.56; 0.84]
Qi L 2018[1034]	101	555	0.18 [0.15; 0.22]
WuLin 2021[1035]	17	194	0.09 [0.05; 0.14]
Ruan F 2013[1039]	63	22200	0.00 [0.00; 0.00]
Shang X 2017[1041]	924	23068	0.04 [0.04; 0.04]
Shi C 2016[1046]	57	1966	0.03 [0.02; 0.04]
Tsang TK 2018[1050]	159	643	0.25 [0.21; 0.28]
Wang X 2016[1055]	51	377	0.14 [0.10; 0.17]
Xu H 2013[1059−1]	207	1630	0.13 [0.11; 0.14]
Xu H 2013[1059−2]	65	1912	0.03 [0.03; 0.04]
Xue C 2014[1060]	278	1995	0.14 [0.12; 0.16]
Yang Z 2011[1068]	427	71534	0.01 [0.01; 0.01]
Zhang L 2018[1071]	631	15118	0.04 [0.04; 0.05]
Zhang TL 2017[1075]	20	327	0.06 [0.04; 0.09]
Zhang XF 2020[1076]	27	450	0.06 [0.04; 0.09]
Zhang XF 2015[1077]	276	2442	0.11 [0.10; 0.13]
Zheng QM 2015[1078]	37	277	0.13 [0.10; 0.18]
Zhou X 2019[1080]	75	1612	0.05 [0.04; 0.06]
Zhou X 2012[1081]	108	5769	0.02 [0.02; 0.02]
LiuQin 2011[1088]	257	2497	0.10 [0.09; 0.12]
XuHaoBin 2015[1091]	39	496	0.08 [0.06; 0.11]
LiYaPin 2019[1096]	162	1813	0.09 [0.08; 0.10]
LiYing 2018[1097]	53	1288	0.04 [0.03; 0.05]
XiangLunHui 2016[1101]	51	533	0.10 [0.07; 0.12]
ZouYong 2011[1103]	39	2672	0.01 [0.01; 0.02]
**Common effect model**		**1703852**	**0.03 [0.03; 0.03]**
**Random effects model**			**0.07 [0.06; 0.07]**

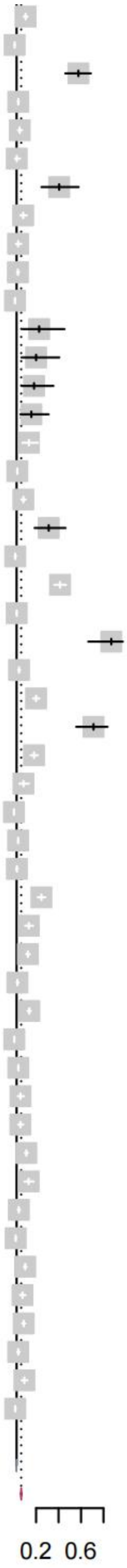

Heterogeneity: I^2 = 99%, τ^2 = 2.0111, p = 0

(a3)

Study	Events	Total	Positive rate	95%−CI
area = North				
AnShuYi 2013[1]	10	155	0.06	[0.03; 0.12]
Xiao−Wei An 2021[2]	5	82	0.06	[0.02; 0.14]
BaiJing 2018[4]	67	353	0.19	[0.15; 0.23]
BaiXue 2020[5]	58	1240	0.05	[0.04; 0.06]
KongFanMing 2019[19]	73	1100	0.07	[0.05; 0.08]
CaiTe 2015[22]	27	300	0.09	[0.06; 0.13]
CaoYongNing 2020[38]	737	2135	0.35	[0.33; 0.37]
ChangXueHong 2017[49]	88	1061	0.08	[0.07; 0.10]
ChenHuiZhong 2019[76]	136	1365	0.10	[0.08; 0.12]
ChenHuiZhong 2018[77]	98	1007	0.10	[0.08; 0.12]
ChenJing 2020[83]	25	372	0.07	[0.04; 0.10]
ChenLingXia 2016[89]	58	246	0.24	[0.18; 0.29]
ChenYanWei 2017[107]	224	1450	0.15	[0.14; 0.17]
Yu−Feng Chen 2021[111]	109	4805	0.02	[0.02; 0.03]
ChengSiSi 2018[118]	239	1250	0.19	[0.17; 0.21]
YanYuXiao 2020[122]	392	909	0.43	[0.40; 0.46]
CuiXiaoShuang 2016[126]	137	942	0.15	[0.12; 0.17]
DaiLei 2019[128]	2	504	0.00	[0.00; 0.01]
DengLi 2015[132]	113	1259	0.09	[0.07; 0.11]
Ying Deng 2012[134]	15	450	0.03	[0.02; 0.05]
DiQianQian 2016[137]	389	2489	0.16	[0.14; 0.17]
DongHeGui 2017[142]	174	1352	0.13	[0.11; 0.15]
DongJianHua 2019[145]	16	114	0.14	[0.08; 0.22]
DongZhaoJing 2020[147]	78	753	0.10	[0.08; 0.13]
DuZhenYuan 2014[149]	45	415	0.11	[0.08; 0.14]
DuanJingJing 2018[151]	74	1287	0.06	[0.05; 0.07]
EJingWen 2014[153]	155	869	0.18	[0.15; 0.21]
FanChaoMeng 2019[154]	169	832	0.20	[0.18; 0.23]
FangYuLian 2019[155]	241	758	0.32	[0.28; 0.35]
FangYuLian 2021[156]	809	3116	0.26	[0.24; 0.28]
GaoLu 2019[179]	99	1536	0.06	[0.05; 0.08]
GaoLu 2019[180]	30	455	0.07	[0.04; 0.09]
GaoXiang 2021[184]	33	588	0.06	[0.04; 0.08]
GaoXin 2020[185]	8	160	0.05	[0.02; 0.10]
GuoJing 2019[203]	193	781	0.25	[0.22; 0.28]
GuoLiMin 2018[207]	34	463	0.07	[0.05; 0.10]
GuoXinHui 2017[210]	54	18912	0.00	[0.00; 0.00]
HanYiFei 2020[214]	137	822	0.17	[0.14; 0.19]
HuaWeiYu 2017[243]	94	572	0.16	[0.13; 0.20]
HuangAiXia 2020[244]	38	449	0.08	[0.06; 0.11]
JiYanLi 2020[266]	136	972	0.14	[0.12; 0.16]
JiYanLi 2017[267]	37	354	0.10	[0.07; 0.14]
Li−Li Jia 2016[277]	58	502	0.12	[0.09; 0.15]
JiaNing 2012[278]	36	214	0.17	[0.12; 0.23]
JiangZhenLong 2014[281]	66	350	0.19	[0.15; 0.23]
JiangHongBo 2019[282]	63	400	0.16	[0.12; 0.20]
JiaoYang 2019[296]	237	1451	0.16	[0.14; 0.18]
JinSongGuo 2015[300]	80	831	0.10	[0.08; 0.12]
KangYanJu 2017[304]	21	88	0.24	[0.15; 0.34]
ZhangJinMing 2017[306]	91	636	0.14	[0.12; 0.17]
LeiYue 2020[312]	121	1028	0.12	[0.10; 0.14]
LiHaiQing 2019[321]	38	752	0.05	[0.04; 0.07]
LiHui 2019[322]	32	363	0.09	[0.06; 0.12]
LiJiShan 2017[323]	133	714	0.19	[0.16; 0.22]
LiJiShan 2012[325]	14	104	0.13	[0.08; 0.22]
LiJiYao 2019[327]	64	520	0.12	[0.10; 0.15]
LiJie 2020[330]	153	2052	0.07	[0.06; 0.09]

LiJunXia 2014[335]	76	718	0.11	[0.08; 0.13]
LiLiYan 2019[337]	52	152	0.34	[0.27; 0.42]
LiRongHua 2019[341]	27	285	0.09	[0.06; 0.13]
LiRuiFeng 2018[342]	155	202	0.77	[0.70; 0.82]
LiRuiQiang 2014[343]	75	282	0.27	[0.22; 0.32]
LiXiang 2015[353−1]	70	385	0.18	[0.14; 0.22]
LiXiang 2015[353−2]	69	385	0.18	[0.14; 0.22]
LiXiang 2013[354]	51	375	0.14	[0.10; 0.17]
LiXiang 2019[355]	177	1335	0.13	[0.11; 0.15]
LiXiang 2019[356]	38	619	0.06	[0.04; 0.08]
LiXiaoYu 2012[358]	24	2046	0.01	[0.01; 0.02]
LiYanHua 2021[363]	277	1513	0.18	[0.16; 0.20]
LiYanYan 2017[364]	62	423	0.15	[0.11; 0.18]
LiZhen 2009[367]	38	80	0.47	[0.36; 0.59]
LiangLiRong 2021[371]	236	2144	0.11	[0.10; 0.12]
LiangQi 2017[375]	16	483	0.03	[0.02; 0.05]
LiangYing 2021[378]	76	468	0.16	[0.13; 0.20]
LinLin 2019[387]	150	1017	0.15	[0.13; 0.17]
LiuBaiWei 2015[397−1]	134	946	0.14	[0.12; 0.17]
LiuBaiWei 2015[397−2]	269	1892	0.14	[0.13; 0.16]
LiuChen 2020[400]	138	1398	0.10	[0.08; 0.12]
LiuDaJing 2018[401]	10	983	0.01	[0.00; 0.02]
LiuGuoRong 2019[407]	38	239	0.16	[0.12; 0.21]
LiuHaiBo 2020[408]	23	395	0.06	[0.04; 0.09]
LiuJiZhao 2020[412]	65	588	0.11	[0.09; 0.14]
LiuLu 2015[418]	63	543	0.12	[0.09; 0.15]
LiuTingTing 2019[425]	33	1029	0.03	[0.02; 0.04]
LiuWei 2021[426]	107	485	0.22	[0.18; 0.26]
LiuXiaoFeng 2015[431]	54	595	0.09	[0.07; 0.12]
LuLiBin 2020[447−1]	36	171	0.21	[0.15; 0.28]
LuLiBin 2020[447−2]	36	172	0.21	[0.15; 0.28]
LuLiBin 2017[448]	46	239	0.19	[0.14; 0.25]
LuanMingChun 2017[464]	24	1253	0.02	[0.01; 0.03]
LuanMingChun 2018[465]	25	1900	0.01	[0.01; 0.02]
LuoJianZhong 2016[469]	17	280	0.06	[0.04; 0.10]
LuoCheng 2020[477]	5	121	0.04	[0.01; 0.09]
MaJuHong 2019[481]	4	163	0.02	[0.01; 0.06]
MaJuan 2020[482]	52	375	0.14	[0.11; 0.18]
MaShuBo 2015[485]	38	2836	0.01	[0.01; 0.02]
MaXin 2016[488]	67	1033	0.06	[0.05; 0.08]
MaXueLian 2021[489]	41	250	0.16	[0.12; 0.22]
MengLiXia 2020[496]	25	110	0.23	[0.15; 0.32]
MengQingHe 2018[497]	48	169	0.28	[0.22; 0.36]
MengQingHe 2016[498]	15	101	0.15	[0.09; 0.23]
MinPei 2011[502]	98	912	0.11	[0.09; 0.13]
MinPei 2012[503]	14	241	0.06	[0.03; 0.10]
NaYongDong 2016[508]	86	1210	0.07	[0.06; 0.09]
PengPai 2018[527]	154	791	0.19	[0.17; 0.22]
QiJi 2019[533]	14	264	0.05	[0.03; 0.09]
QiaoHongYing 2021[541]	17	641	0.03	[0.02; 0.04]
QinMeng 2014[546]	7	47	0.15	[0.06; 0.28]
QiuZhengYong 2019[553]	273	5412	0.05	[0.04; 0.06]
RenShuMin 2018[558]	17	340	0.05	[0.03; 0.08]
RongXiaoSu 2021[564]	47	347	0.14	[0.10; 0.18]
SangXiangLai 2016[569]	61	1545	0.04	[0.03; 0.05]
SangXiangLai 2018[570]	607	1580	0.38	[0.36; 0.41]
ShaBiReMu·TuoHeTaMu 2013[571]	73	379	0.19	[0.15; 0.24]
ShaBiReMu·TuoHeTaMu 2018[572]	150	895	0.17	[0.14; 0.19]
ShiXin 2020[584]	40	560	0.07	[0.05; 0.10]
ShiWenFeng 2019[585]	116	1105	0.10	[0.09; 0.12]

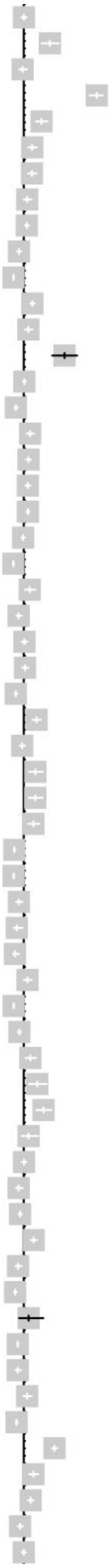

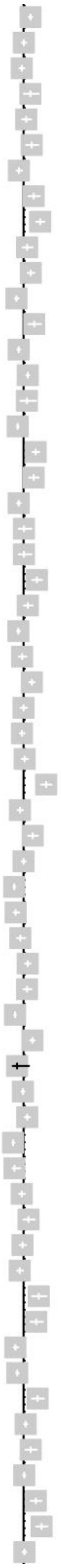

SuTong 2017[603]	372	2211	0.17	[0.15; 0.18]
SuWenJun 2013[605]	98	912	0.11	[0.09; 0.13]
SunBo 2021[607]	60	680	0.09	[0.07; 0.11]
SunJianFei 2014[610]	20	120	0.17	[0.10; 0.25]
SunYaNa 2020[619]	35	271	0.13	[0.09; 0.18]
SunYu 2018[622]	36	201	0.18	[0.13; 0.24]
SunYueLin 2017[623]	26	419	0.06	[0.04; 0.09]
TianDeng 2018[637]	47	238	0.20	[0.15; 0.25]
TianGeng 2015[638]	133	519	0.26	[0.22; 0.30]
TianHong 2011[639]	31	226	0.14	[0.10; 0.19]
WangChunRong 2015[650]	99	579	0.17	[0.14; 0.20]
WangHuan 2017[658]	21	530	0.04	[0.02; 0.06]
WangJuan 2012[662]	40	198	0.20	[0.15; 0.26]
WangSanTao 2018[674]	84	1393	0.06	[0.05; 0.07]
WangTongYu 2019[679]	356	2490	0.14	[0.13; 0.16]
WangWeiRu 2013[680]	13	96	0.14	[0.07; 0.22]
WangWenLei 2017[683]	125	2450	0.05	[0.04; 0.06]
WangXi 2016[685]	140	650	0.22	[0.18; 0.25]
WangXi 2017[686]	67	340	0.20	[0.16; 0.24]
WangXi 2018[687]	51	846	0.06	[0.05; 0.08]
WangXiaoYi 2017[690]	13	120	0.11	[0.06; 0.18]
WangXiaoYi 2017[691]	13	120	0.11	[0.06; 0.18]
WangYanBo 2017[695]	78	344	0.23	[0.18; 0.27]
WangYanHua 2021[696]	43	300	0.14	[0.11; 0.19]
WangYanYan 2019[697]	64	1123	0.06	[0.04; 0.07]
WangYongXia 2012[698]	27	295	0.09	[0.06; 0.13]
WangYu 2019[699]	169	938	0.18	[0.16; 0.21]
WangYuanYuan 2015[701]	48	459	0.10	[0.08; 0.14]
WangYue 2020[702]	65	718	0.09	[0.07; 0.11]
WangYueSheng 2015[703]	58	506	0.11	[0.09; 0.15]
WeiKongFu 2014[707]	120	385	0.31	[0.27; 0.36]
WeiXinHong 2020[709]	31	433	0.07	[0.05; 0.10]
XiaoLin 2014[747]	60	319	0.19	[0.15; 0.24]
XieYaXian 2021[755]	110	1050	0.10	[0.09; 0.12]
XingYuFang 2019[760]	21	1079	0.02	[0.01; 0.03]
XuJun 2012[780]	7	218	0.03	[0.01; 0.07]
YanXin 2018[787]	15	200	0.07	[0.04; 0.12]
YanYuXiao 2020[788]	112	785	0.14	[0.12; 0.17]
YanWei 2020[793]	48	351	0.14	[0.10; 0.18]
YangHui 2020[799]	54	2000	0.03	[0.02; 0.04]
YangLi 2018[805]	240	1292	0.19	[0.16; 0.21]
YangMing 2011[807]	2	44	0.05	[0.01; 0.15]
YangXianDa 2019[814]	183	1855	0.10	[0.09; 0.11]
YangYanNa 2018[816]	113	818	0.14	[0.12; 0.16]
YiMing 2018[836]	5	550	0.01	[0.00; 0.02]
YiYing 2013[838]	2	94	0.02	[0.00; 0.07]
ZhaoWei 2011[841]	38	432	0.09	[0.06; 0.12]
YuMiao 2019[844]	30	200	0.15	[0.10; 0.21]
YuanLu 2018[855]	38	400	0.10	[0.07; 0.13]
ZhangDongYu 2020[865]	25	360	0.07	[0.05; 0.10]
ZhangJin 2018[873]	42	172	0.24	[0.18; 0.32]
ZhangLiShuang 2019[876]	47	213	0.22	[0.17; 0.28]
ZhangLi 2015[877]	9	303	0.03	[0.01; 0.06]
ZhangShuHong 2019[886]	58	1240	0.05	[0.04; 0.06]
ZhangShuang 2016[887]	56	240	0.23	[0.18; 0.29]
ZhangWeiWei 2021[890]	232	1870	0.12	[0.11; 0.14]
ZhangYaKong 2017[892]	36	212	0.17	[0.12; 0.23]
ZhangYanFei 2018[899]	398	3592	0.11	[0.10; 0.12]
ZhangYongHong 2015[901]	62	286	0.22	[0.17; 0.27]
ZhangZhiQiang 2019[905]	79	293	0.27	[0.22; 0.32]
ZhangShaSha 2017[910]	166	1476	0.11	[0.10; 0.13]

ZhaoDan 2017[912]	74	409	0.18 [0.14; 0.22]
ZhaoDan 2019[913]	113	1412	0.08 [0.07; 0.10]
ZhaoHuiLing 2017[914]	20	336	0.06 [0.04; 0.09]
ZhaoJiaYong 2016[915]	225	1964	0.11 [0.10; 0.13]
ZhaoLiJiang 2019[917]	160	1125	0.14 [0.12; 0.16]
ZhaoWenNa 2019[925]	236	1451	0.16 [0.14; 0.18]
ZhaoXiangJu 2018[926]	24	352	0.07 [0.04; 0.10]
ZhaoXiangJu 2018[927]	47	295	0.16 [0.12; 0.21]
ZhaoYaLi 2020[931]	40	510	0.08 [0.06; 0.11]
ZhaoYunQing 2020[934]	471	1478	0.32 [0.29; 0.34]
ZhaoYun 2018[935]	871	5763	0.15 [0.14; 0.16]
ZhengWenLong 2019[940]	180	1249	0.14 [0.13; 0.16]
ZhouAiMin 2020[951]	11	408	0.03 [0.01; 0.05]
ZhouYing 2017[965]	177	1754	0.10 [0.09; 0.12]
ZhuGuanQi 2020[970]	245	2514	0.10 [0.09; 0.11]
ZhuJiHua 2020[973]	76	1520	0.05 [0.04; 0.06]
ZhuTingTing 2015[975]	49	341	0.14 [0.11; 0.19]
ZhuLin 2020[979]	42	364	0.12 [0.08; 0.15]
Yuan−yun Ao 2014[983]	2	466	0.00 [0.00; 0.02]
JialLiPing 2021[991]	215	1213	0.18 [0.16; 0.20]
Z Gao 2015[997]	263	3832	0.07 [0.06; 0.08]
Gao Z 2015[998]	191	640	0.30 [0.26; 0.34]
Jia L 2017[1010]	304	999	0.30 [0.28; 0.33]
Jia LP 2014[1011]	182	249	0.73 [0.67; 0.78]
Li HY 2019[1014]	450	1863	0.24 [0.22; 0.26]
Liu J 2018[1022]	28	35	0.80 [0.63; 0.92]
Liu P 2014[1023]	34	124	0.27 [0.20; 0.36]
Liu X 2015[1024]	328	362	0.91 [0.87; 0.93]
Mai H 2016[1031]	9	124	0.07 [0.03; 0.13]
Mai H 2013[1032]	26	171	0.15 [0.10; 0.21]
Qiao N 2017[1036]	115	5633	0.02 [0.02; 0.02]
Sang S 2014[1040]	66	685	0.10 [0.08; 0.12]
Shen XX 2019[1044]	21	634	0.03 [0.02; 0.05]
C Sun 2021[1047−1]	75	733	0.10 [0.08; 0.13]
C Sun 2021[1047−2]	76	733	0.10 [0.08; 0.13]
Sun XM 2016[1048−1]	19	202	0.09 [0.06; 0.14]
Yang S 2019[1067]	13	81	0.16 [0.09; 0.26]
Zhang S 2011[1073]	60	201	0.30 [0.24; 0.37]
CuiXiaoShuang 2013[1086]	96	566	0.17 [0.14; 0.20]
HouJun 2012[1087]	6	57	0.11 [0.04; 0.22]
Aixiaoman 2015[1094]	1	43	0.02 [0.00; 0.12]
DangWen 2019[1106]	219	1083	0.20 [0.18; 0.23]
HeFei 2020[1108]	610	2185	0.28 [0.26; 0.30]
HuangDouDou 2015[1111]	100	716	0.14 [0.12; 0.17]
LiWeiWei 2020[1114]	138	648	0.21 [0.18; 0.25]
LiuYun 2019[1116]	353	1384	0.26 [0.23; 0.28]
MaYan 2014[1118]	52	311	0.17 [0.13; 0.21]
SaiLinTao 2014[1120]	122	1247	0.10 [0.08; 0.12]
SangShaoWei 2012[1121]	66	685	0.10 [0.08; 0.12]
SuCheng 2020[1123]	137	1042	0.13 [0.11; 0.15]
TanZiMing 2018[1124]	49	683	0.07 [0.05; 0.09]
TangZhiJian 2013[1125]	27	384	0.07 [0.05; 0.10]
WangHaiYan 2017[1126]	70	671	0.10 [0.08; 0.13]
WuJie 2017[1129]	48	181	0.27 [0.20; 0.34]
**Common effect model**		**213651**	**0.11 [0.11; 0.11]**
**Random effects model**			**0.11 [0.10; 0.13]**

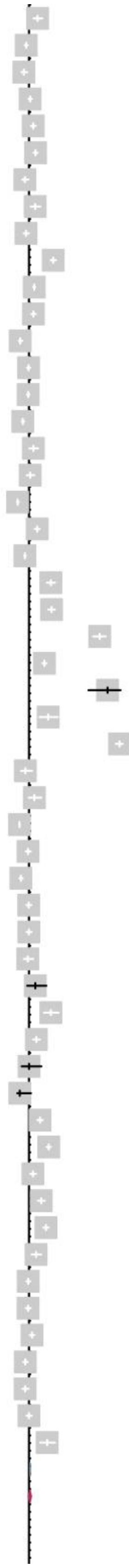

Heterogeneity: $I^2 = 98\%$, $\tau^2 = 0.9384$, $p = 0$

area = South

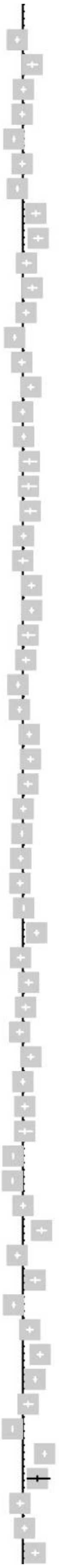

BaiYongFeng 2014[7]	34	654	0.05 [0.04; 0.07]
BaoLin 2019[9]	58	300	0.19 [0.15; 0.24]
BiWenJun 2020[12]	115	1041	0.11 [0.09; 0.13]
BiWenJun 2019[13]	76	768	0.10 [0.08; 0.12]
CaiMiaoSen 2016[15]	27	1319	0.02 [0.01; 0.03]
CaiMin 2019[16]	65	658	0.10 [0.08; 0.12]
CaiYing 2017[28]	113	2129	0.05 [0.04; 0.06]
CaoJian 2017[29]	106	465	0.23 [0.19; 0.27]
CaoRanRan 2015[31]	107	428	0.25 [0.21; 0.29]
CaoYiHui 2021[34−1]	43	308	0.14 [0.10; 0.18]
CaoYiHui 2021[34−2]	73	376	0.19 [0.16; 0.24]
CaoYiHui 2021[35−1]	96	709	0.14 [0.11; 0.16]
CaoYiHui 2021[35−2]	24	869	0.03 [0.02; 0.04]
CaoYiHui 2017[36−1]	42	443	0.09 [0.07; 0.13]
CaoYiHui 2017[36−2]	167	923	0.18 [0.16; 0.21]
CaoYiHui 2017[36−3]	119	1149	0.10 [0.09; 0.12]
CaoYiHui 2017[36−4]	193	1708	0.11 [0.10; 0.13]
ZengDeXing 2015[40]	25	150	0.17 [0.11; 0.24]
ZengHuaShu 2015[42]	19	120	0.16 [0.10; 0.24]
ZengJunRong 2013[43]	38	218	0.17 [0.13; 0.23]
ChangHaiLing 2016[48]	98	881	0.11 [0.09; 0.13]
ShenMeiYun 2016[51]	24	231	0.10 [0.07; 0.15]
ShenYueHua 2016[56]	149	797	0.19 [0.16; 0.22]
ChenAQun 2017[57]	428	2274	0.19 [0.17; 0.20]
ChenFengQin 2013[65]	24	156	0.15 [0.10; 0.22]
ChenFengQin 2012[66]	61	459	0.13 [0.10; 0.17]
ChenGaoShang 2020[67]	80	1363	0.06 [0.05; 0.07]
ChenGaoShang 2018[68]	84	1154	0.07 [0.06; 0.09]
ChenGuoPing 2018[70]	234	1415	0.17 [0.15; 0.19]
ChenHaiLi 2020[71]	162	912	0.18 [0.15; 0.20]
ChenHaoChuan 2019[72]	63	411	0.15 [0.12; 0.19]
ChenHuiFang 2014[75]	77	709	0.11 [0.09; 0.13]
ChenJiang 2019[81]	655	6753	0.10 [0.09; 0.10]
ChenJingHong 2015[82]	70	825	0.08 [0.07; 0.11]
ChenJun 2020[85]	36	464	0.08 [0.05; 0.11]
ChenLiLi 2018[86]	2253	19930	0.11 [0.11; 0.12]
ChenLiPing 2020[87]	414	1757	0.24 [0.22; 0.26]
ChenLinLin 2018[88]	26	313	0.08 [0.05; 0.12]
ChenLongHui 2015[90]	73	459	0.16 [0.13; 0.20]
ChenMin 2016[91]	49	374	0.13 [0.10; 0.17]
ChenMinMei 2014[93]	24	323	0.07 [0.05; 0.11]
ChenPing 2013[94]	147	811	0.18 [0.16; 0.21]
ChenWeiPing 2018[97]	138	1308	0.11 [0.09; 0.12]
ChenXiKai 2012[98]	60	485	0.12 [0.10; 0.16]
ChenXiaoDong 2020[99]	15	122	0.12 [0.07; 0.19]
ChenYan 2020[105]	94	6986	0.01 [0.01; 0.02]
ChenYan 2016[106]	13	1747	0.01 [0.00; 0.01]
ChenYu 2016[110]	100	936	0.11 [0.09; 0.13]
ChenYun 2017[112]	115	408	0.28 [0.24; 0.33]
ChenZhenMing 2012[113]	22	426	0.05 [0.03; 0.08]
ChenZhiHao 2012[114]	81	366	0.22 [0.18; 0.27]
ChengCheng 2019[116]	9	524	0.02 [0.01; 0.03]
ChengYuan 2017[119]	107	627	0.17 [0.14; 0.20]
CuiDaWei 2016[121]	298	1109	0.27 [0.24; 0.30]
DengAiPing 2014[130]	1028	4644	0.22 [0.21; 0.23]
DengJianKai 2015[131]	45	290	0.16 [0.12; 0.20]
DiGuangFu 2019[136]	66	10085	0.01 [0.01; 0.01]
DingMing 2016[139]	1314	4214	0.31 [0.30; 0.33]
DingYouFa 2013[140]	17	70	0.24 [0.15; 0.36]
DongHongYan 2015[143]	26	336	0.08 [0.05; 0.11]
DongHongYan 2017[144]	112	926	0.12 [0.10; 0.14]
DuYao 2013[150]	157	708	0.22 [0.19; 0.25]

FeiYi 2013[159]	58	619	0.09	[0.07; 0.12]
FuJianGuang 2011[165]	26	92	0.28	[0.19; 0.39]
FuYun 2015[166]	171	628	0.27	[0.24; 0.31]
FuYun 2019[167]	331	1991	0.17	[0.15; 0.18]
FuLi 2015[168]	271	1904	0.14	[0.13; 0.16]
FuYaLi 2017[169]	110	583	0.19	[0.16; 0.22]
FuYiFei 2013[170]	110	1327	0.08	[0.07; 0.10]
GaoGuiLing 2015[173]	39	617	0.06	[0.05; 0.09]
GaoHongMei 2020[175]	51	3003	0.02	[0.01; 0.02]
GaoLei 2021[178]	337	3801	0.09	[0.08; 0.10]
GeBin 2018[188]	37	180	0.21	[0.15; 0.27]
GeHaiXia 2018[189]	293	1735	0.17	[0.15; 0.19]
GengQian 2017[190]	177	1516	0.12	[0.10; 0.13]
GongChunHua 2018[191]	123	617	0.20	[0.17; 0.23]
GongMin 2018[193]	103	462	0.22	[0.19; 0.26]
GuanHongXia 2016[198]	86	915	0.09	[0.08; 0.11]
GuanHongXia 2014[199]	45	291	0.15	[0.12; 0.20]
GuoBaoFu 2016[201]	203	1226	0.17	[0.15; 0.19]
GuoJuan 2021[204]	60	1036	0.06	[0.04; 0.07]
HanDongJie 2016[211−1]	112	782	0.14	[0.12; 0.17]
HanDongJie 2016[211−2]	174	4226	0.04	[0.04; 0.05]
HanNing 2016[213]	50	287	0.17	[0.13; 0.22]
HaoShiXuan 2017[215]	42	832	0.05	[0.04; 0.07]
HeChuJie 2019[217]	241	914	0.26	[0.24; 0.29]
HeLanXiang 2018[218]	37	318	0.12	[0.08; 0.16]
HeLiYing 2017[219]	95	850	0.11	[0.09; 0.13]
HeMengTing 2020[220]	14	240	0.06	[0.03; 0.10]
HeQiuYu 2019[221]	402	3066	0.13	[0.12; 0.14]
HeFangQing 2017[223]	17	150	0.11	[0.07; 0.18]
HongChengJi 2016[225]	80	517	0.15	[0.12; 0.19]
HongWanSheng 2015[226]	46	236	0.19	[0.15; 0.25]
HongWanSheng 2015[227]	71	507	0.14	[0.11; 0.17]
HongYing 2019[228]	134	911	0.15	[0.12; 0.17]
HongZhanTong 2015[229]	192	1587	0.12	[0.11; 0.14]
HuHui 2018[234]	54	700	0.08	[0.06; 0.10]
HuJing 2019[235]	41	261	0.16	[0.12; 0.21]
HuJuMei 2014[236]	91	454	0.20	[0.16; 0.24]
HuTingTing 2013[237]	39	312	0.12	[0.09; 0.17]
HuTingTing 2013[238]	11	127	0.09	[0.04; 0.15]
HuZhuo 2017[240]	14	190	0.07	[0.04; 0.12]
HuangEnMiao 2019[249]	465	32831	0.01	[0.01; 0.02]
HuangFengGuang 2018[250]	45	673	0.07	[0.05; 0.09]
HuangPengFei 2018[255]	59	258	0.23	[0.18; 0.28]
HuangTianRan 2020[258]	15	48	0.31	[0.19; 0.46]
HuangYong 2018[262]	732	5026	0.15	[0.14; 0.16]
JiLei 2018[268]	101	501	0.20	[0.17; 0.24]
JiLei 2019[273]	183	1259	0.15	[0.13; 0.17]
JiXinFeng 2012[275]	27	241	0.11	[0.08; 0.16]
JiangLingLing 2018[279]	15	2610	0.01	[0.00; 0.01]
JiangXiao 2018[280]	14	300	0.05	[0.03; 0.08]
JiangChunMei 2013[286]	17	271	0.06	[0.04; 0.10]
JiangCuiLian 2016[287]	67	322	0.21	[0.17; 0.26]
JiangCuiLian 2018[288]	31	300	0.10	[0.07; 0.14]
JiangCuiLian 2016[289]	27	178	0.15	[0.10; 0.21]
JiangHongJun 2020[290]	325	2230	0.15	[0.13; 0.16]
JiangWenJun 2019[292]	22	121	0.18	[0.12; 0.26]
JiangZhuoJing 2020[295]	93	645	0.14	[0.12; 0.17]
JinDan 2017[297]	60	1309	0.05	[0.04; 0.06]
JinDi 2021[298]	31	442	0.07	[0.05; 0.10]
JinSaiYan 2012[299]	45	325	0.14	[0.10; 0.18]

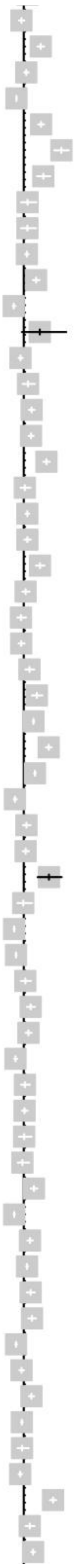

JinXiaoMin 2018[301]	43	514	0.08	[0.06; 0.11]
KongBoLi 2015[305]	212	819	0.26	[0.23; 0.29]
LanYingYing 2021[309]	129	1012	0.13	[0.11; 0.15]
LeiLei 2016[310]	274	7120	0.04	[0.03; 0.04]
LiJian 2014[313]	262	1001	0.26	[0.23; 0.29]
LiJingQuan 2013[314]	100	224	0.45	[0.38; 0.51]
LiBo 2014[317]	64	227	0.28	[0.22; 0.35]
LiJing 2015[331−1]	15	108	0.14	[0.08; 0.22]
LiJing 2015[331−2]	15	109	0.14	[0.08; 0.22]
LiJing 2021[332]	260	1957	0.13	[0.12; 0.15]
LiJing 2017[333]	149	687	0.22	[0.19; 0.25]
LiPing 2020[339]	6	433	0.01	[0.01; 0.03]
LiWei 2015[351]	5	20	0.25	[0.09; 0.49]
LiXiaoLe 2012[357]	64	856	0.07	[0.06; 0.09]
LiYan 2015[360]	23	160	0.14	[0.09; 0.21]
LiYanFen 2014[362]	146	831	0.18	[0.15; 0.20]
LiangCuiQiong 2020[369]	179	1050	0.17	[0.15; 0.19]
LiangJunHe 2015[370]	282	908	0.31	[0.28; 0.34]
LiangLiang 2017[372]	23	201	0.11	[0.07; 0.17]
LiangLu 2014[373]	220	1607	0.14	[0.12; 0.15]
LiangXiaoLian 2015[377]	75	546	0.14	[0.11; 0.17]
LiaoYang 2011[382]	122	484	0.25	[0.21; 0.29]
LinDan 2016[383]	48	395	0.12	[0.09; 0.16]
LinLiQun 2020[385−1]	16	202	0.08	[0.05; 0.13]
LinLiQun 2020[385−2]	33	405	0.08	[0.06; 0.11]
LinLiJuan 2016[386]	51	326	0.16	[0.12; 0.20]
LinQian 2012[389]	67	300	0.22	[0.18; 0.27]
LinSheng 2019[392]	2330	12083	0.19	[0.19; 0.20]
LinYiXiong 2016[394]	381	1150	0.33	[0.30; 0.36]
LiuDan 2021[402]	1938	9397	0.21	[0.20; 0.21]
LiuGuiDan 2017[405]	12	494	0.02	[0.01; 0.04]
LiuHanZhao 2018[409]	49	381	0.13	[0.10; 0.17]
LiuLiang 2015[414]	64	526	0.12	[0.09; 0.15]
LiuKaiQian 2013[416]	26	78	0.33	[0.23; 0.45]
LiuMeiFang 2011[419]	9	90	0.10	[0.05; 0.18]
LiuWenFu 2018[427]	42	2411	0.02	[0.01; 0.02]
LiuWuGao 2017[429]	215	6262	0.03	[0.03; 0.04]
LiuXiuLan 2019[432]	24	202	0.12	[0.08; 0.17]
LiuXueJie 2019[434]	69	412	0.17	[0.13; 0.21]
LiuYaWei 2013[435]	91	619	0.15	[0.12; 0.18]
LiuLiJiang 2016[439]	18	609	0.03	[0.02; 0.05]
LongHaoYu 2010[440]	29	252	0.12	[0.08; 0.16]
LongQiZhi 2019[443]	65	580	0.11	[0.09; 0.14]
LongQianJin 2020[444]	13	120	0.11	[0.06; 0.18]
LouQian 2012[445]	12	130	0.09	[0.05; 0.16]
LuHongPing 2020[446]	132	670	0.20	[0.17; 0.23]
LuXiangDui 2019[449]	39	2201	0.02	[0.01; 0.02]
LuZhanPeng 2014[450]	135	838	0.16	[0.14; 0.19]
LuDongLei 2020[452]	895	6664	0.13	[0.13; 0.14]
LuHanMing 2018[453]	70	419	0.17	[0.13; 0.21]
LuHuan 2021[455]	85	474	0.18	[0.15; 0.22]
LuQun 2016[457]	82	2376	0.03	[0.03; 0.04]
LuGongJin 2017[459]	59	699	0.08	[0.06; 0.11]
LuLiJun 2015[460]	174	999	0.17	[0.15; 0.20]
LuPeng 2015[461]	399	4568	0.09	[0.08; 0.10]
LuoHengLi 2018[468]	12	135	0.09	[0.05; 0.15]
LuoKaiWei 2018[470]	81	1122	0.07	[0.06; 0.09]
LuoLan 2013[471]	395	1066	0.37	[0.34; 0.40]
LuoLiang 2018[473]	51	321	0.16	[0.12; 0.20]
LuoXueMei 2016[476]	345	1822	0.19	[0.17; 0.21]

LuoLingFei 2017[478]	207	803	0.26	[0.23; 0.29]
LuoShanCai 2018[479]	78	1672	0.05	[0.04; 0.06]
MaHuaPing 2016[480]	20	95	0.21	[0.13; 0.31]
MaLiangLiang 2018[483]	19	301	0.06	[0.04; 0.10]
MaYaPing 2018[490]	190	883	0.22	[0.19; 0.24]
MaYongJun 2016[491]	176	822	0.21	[0.19; 0.24]
MaoJianYing 2016[492]	98	2486	0.04	[0.03; 0.05]
MeiGuoYong 2019[494]	15	308	0.05	[0.03; 0.08]
MiaoShengHao 2018[500]	111	2473	0.04	[0.04; 0.05]
MiaoShengHao 2017[501]	58	1030	0.06	[0.04; 0.07]
MiaoGuoZhong 2013[506]	12	66	0.18	[0.10; 0.30]
NongHao 2020[514]	238	1199	0.20	[0.18; 0.22]
PanHao 2017[517]	1172	6543	0.18	[0.17; 0.19]
PanLiFeng 2015[518]	1053	6392	0.16	[0.16; 0.17]
PanLiFeng 2012[519]	110	1327	0.08	[0.07; 0.10]
PanLiFeng 2015[520−1]	362	1701	0.21	[0.19; 0.23]
PanLiFeng 2015[520−2]	362	1702	0.21	[0.19; 0.23]
PangBeiBei 2016[523]	324	615	0.53	[0.49; 0.57]
QiHuiZhou 2014[530]	63	116	0.54	[0.45; 0.64]
QiXianQun 2021[531]	44	529	0.08	[0.06; 0.11]
QianYanHua 2011[538]	7	163	0.04	[0.02; 0.09]
QiaoKun 2014[542]	180	539	0.33	[0.29; 0.38]
QiaoYingQin 2016[543]	67	511	0.13	[0.10; 0.16]
QiuCanLin 2015[550]	156	431	0.36	[0.32; 0.41]
QiuXiang 2019[552]	32	1495	0.02	[0.01; 0.03]
RenYaPing 2020[559]	97	484	0.20	[0.17; 0.24]
RenYan 2020[560]	23	105	0.22	[0.14; 0.31]
RenYan 2018[561]	53	362	0.15	[0.11; 0.19]
RongJiangRui 2014[563]	18	335	0.05	[0.03; 0.08]
RuanYang 2019[565]	21	1580	0.01	[0.01; 0.02]
SangHao 2016[567]	344	858	0.40	[0.37; 0.43]
SangHao 2019[568]	70	880	0.08	[0.06; 0.10]
ShangLiHong 2016[573]	229	1064	0.22	[0.19; 0.24]
ShangXiaoChun 2020[574]	310	1350	0.23	[0.21; 0.25]
ShenHongWei 2019[575]	228	1972	0.12	[0.10; 0.13]
ShenTuPingPing 2018[576]	90	1339	0.07	[0.05; 0.08]
ShiAiPing 2021[578]	54	419	0.13	[0.10; 0.16]
ShiQianFeng 2014[580]	9	124	0.07	[0.03; 0.13]
ShiQianFeng 2015[581]	19	124	0.15	[0.09; 0.23]
ShiPing 2016[583]	52	338	0.15	[0.12; 0.20]
ShiYongLin 2014[586]	66	549	0.12	[0.09; 0.15]
ShuYouPing 2020[588]	80	776	0.10	[0.08; 0.13]
SuJing 2016[602]	55	987	0.06	[0.04; 0.07]
SuWenZhe 2019[606]	220	854	0.26	[0.23; 0.29]
SunHuaMin 2019[609]	54	1244	0.04	[0.03; 0.06]
SunMingHua 2015[615]	24	170	0.14	[0.09; 0.20]
SunQingShuang 2017[617]	17	201	0.08	[0.05; 0.13]
SunYangMing 2016[620]	162	1541	0.11	[0.09; 0.12]
SunYangMing 2016[621]	92	863	0.11	[0.09; 0.13]
QinLin 2017[626]	15	75	0.20	[0.12; 0.31]
TanWeiWei 2015[628]	6	753	0.01	[0.00; 0.02]
TanWeiWei 2018[629]	34	363	0.09	[0.07; 0.13]
TangXiang 2014[632]	84	384	0.22	[0.18; 0.26]
TangZhen 2016[635]	1308	16658	0.08	[0.07; 0.08]
TianJiGui 2019[640]	11	228	0.05	[0.02; 0.08]
WangHui 2016[644]	136	732	0.19	[0.16; 0.22]
WangYang 2014[647]	237	2236	0.11	[0.09; 0.12]
WangAnNa 2016[648]	15	75	0.20	[0.12; 0.31]
WangDaHu 2012[651]	99	14281	0.01	[0.01; 0.01]
WangDongYue 2016[653]	232	2234	0.10	[0.09; 0.12]
WangGe 2016[654]	41	572	0.07	[0.05; 0.10]

WangHaiYan 2014[655]	75	300	0.25	[0.20; 0.30]
WangJie 2016[661]	4	3190	0.00	[0.00; 0.00]
WangLiLi 2021[667]	326	2123	0.15	[0.14; 0.17]
WangPing 2016[672]	34	64	0.53	[0.40; 0.66]
WangQiMei 2019[673]	179	4243	0.04	[0.04; 0.05]
WangSheLiang 2011[675]	30	300	0.10	[0.07; 0.14]
WangWenYing 2011[681]	204	650	0.31	[0.28; 0.35]
WangWenQing 2020[682]	1428	9301	0.15	[0.15; 0.16]
WangWeiXiang 2021[684]	292	1358	0.22	[0.19; 0.24]
WangYaFang 2019[694]	86	3766	0.02	[0.02; 0.03]
WangYuPing 2016[700]	23	848	0.03	[0.02; 0.04]
WangZiYou 2020[704]	87	976	0.09	[0.07; 0.11]
WengXiaoQin 2017[712]	137	1000	0.14	[0.12; 0.16]
WuBingShan 2018[714]	141	892	0.16	[0.13; 0.18]
WuJing 2015[720]	7	42	0.17	[0.07; 0.31]
WuJing 2018[721]	6	153	0.04	[0.01; 0.08]
WuJing 2019[722]	13	310	0.04	[0.02; 0.07]
WuPei 2011[724]	146	1878	0.08	[0.07; 0.09]
WuShengHai 2019[726]	66	605	0.11	[0.09; 0.14]
WuWei 2012[728]	113	540	0.21	[0.18; 0.25]
WuXiaoFang 2017[731]	256	873	0.29	[0.26; 0.32]
WuYaTing 2019[740]	9	915	0.01	[0.00; 0.02]
XiaoYong 2016[748]	40	409	0.10	[0.07; 0.13]
XieChunYan 2014[750]	153	1095	0.14	[0.12; 0.16]
XieHongYi 2020[751]	48	204	0.24	[0.18; 0.30]
XieSiRou 2017[753]	62	600	0.10	[0.08; 0.13]
XiongFeiYu 2016[761]	176	2148	0.08	[0.07; 0.09]
XiongXiaoShun 2017[762]	5	7138	0.00	[0.00; 0.00]
XuJi 2021[763]	712	2021	0.35	[0.33; 0.37]
XuDan 2014[764]	125	748	0.17	[0.14; 0.20]
XuDan 2012[765]	24	440	0.05	[0.04; 0.08]
XuFenFen 2018[766]	170	2793	0.06	[0.05; 0.07]
XuLiXia 2016[768]	61	417	0.15	[0.11; 0.18]
XuQin 2014[769]	55	513	0.11	[0.08; 0.14]
XuRuiQuan 2021[770]	259	856	0.30	[0.27; 0.33]
XuZhongQing 2016[773]	148	1554	0.10	[0.08; 0.11]
XuHuaJing 2019[776]	14	108	0.13	[0.07; 0.21]
XuJinFeng 2017[777]	87	1605	0.05	[0.04; 0.07]
XuLiQin 2015[781−1]	45	311	0.14	[0.11; 0.19]
XuLiQin 2015[781−2]	45	311	0.14	[0.11; 0.19]
XuXiaoChan 2015[783]	64	642	0.10	[0.08; 0.13]
XueCaoYi 2017[785]	2186	10126	0.22	[0.21; 0.22]
YanWei 2019[789]	121	578	0.21	[0.18; 0.24]
YanYan 2013[790]	35	70	0.50	[0.38; 0.62]
YanYan 2012[791]	274	426	0.64	[0.60; 0.69]
YanFang 2018[794]	48	264	0.18	[0.14; 0.23]
YangHui 2016[800]	318	2161	0.15	[0.13; 0.16]
YangJinHong 2020[803]	73	17516	0.00	[0.00; 0.01]
YangMei 2017[806]	52	1557	0.03	[0.03; 0.04]
YangRuSong 2016[809]	22	361	0.06	[0.04; 0.09]
YangTianChi 2016[812]	53	728	0.07	[0.06; 0.09]
YangXiaoJin 2016[815]	102	1982	0.05	[0.04; 0.06]
YangYiLong 2015[817]	92	766	0.12	[0.10; 0.15]
YaoJianXiang 2016[823]	12	84	0.14	[0.08; 0.24]
YaoLiLi 2019[825]	70	414	0.17	[0.13; 0.21]
YaoLiLi 2020[826]	188	1083	0.17	[0.15; 0.20]
YaoXiuPing 2021[828]	116	614	0.19	[0.16; 0.22]
YeHongYan 2013[829]	176	900	0.20	[0.17; 0.22]
YeYanHua 2018[834]	28	1259	0.02	[0.01; 0.03]
YeYuHui 2015[835]	364	1744	0.21	[0.19; 0.23]

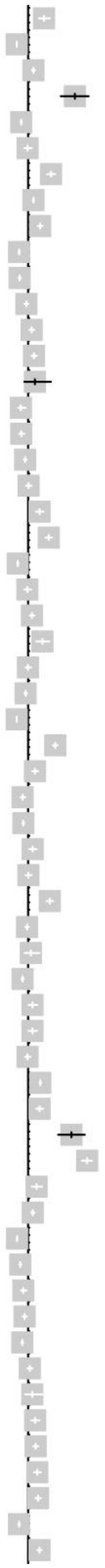

YouXingYong 2020[842]	22	5426	0.00	[0.00; 0.01]
YuFangYuan 2020[843]	265	709	0.37	[0.34; 0.41]
YuYingHui 2020[846]	171	1497	0.11	[0.10; 0.13]
YuGuangQing 2013[847]	82	403	0.20	[0.17; 0.25]
YuJinCun 2020[850]	79	1017	0.08	[0.06; 0.10]
YuanJianMing 2019[853]	48	428	0.11	[0.08; 0.15]
YuanYongJuan 2019[856]	137	288	0.48	[0.42; 0.54]
ZhangHaiLong 2013[866]	114	486	0.23	[0.20; 0.27]
ZhangHuanZhu 2015[870]	67	257	0.26	[0.21; 0.32]
ZhangJianMin 2014[872−1]	8	76	0.11	[0.05; 0.20]
ZhangJianMin 2014[872−2]	9	76	0.12	[0.06; 0.21]
ZhangJianMin 2014[872−3]	17	152	0.11	[0.07; 0.17]
ZhangJing 2016[874]	184	1878	0.10	[0.08; 0.11]
ZhangLingLing 2016[878]	82	842	0.10	[0.08; 0.12]
ZhangMin 2015[881]	72	532	0.14	[0.11; 0.17]
ZhangQian 2017[884]	15	169	0.09	[0.05; 0.14]
ZhangWangSheng 2020[889]	110	1341	0.08	[0.07; 0.10]
ZhangYaLin 2018[893]	40	303	0.13	[0.10; 0.18]
ZhangYan 2017[895]	136	1320	0.10	[0.09; 0.12]
ZhangYanLi 2013[896]	17	242	0.07	[0.04; 0.11]
ZhangYanLi 2019[897]	101	961	0.11	[0.09; 0.13]
ZhangYong 2017[902]	309	4728	0.07	[0.06; 0.07]
ZhaoTianWang 2018[922]	27	440	0.06	[0.04; 0.09]
ZhaoXiaoLing 2013[928]	63	371	0.17	[0.13; 0.21]
ZhaoXueQin 2014[930]	55	307	0.18	[0.14; 0.23]
ZhengLei 2013[938]	36	145	0.25	[0.18; 0.33]
ZhengShuFa 2016[939]	207	4680	0.04	[0.04; 0.05]
ZhengXiaoYan 2018[941]	16	222	0.07	[0.04; 0.11]
ZhengYaPing 2016[942]	39	543	0.07	[0.05; 0.10]
ZhuGeXiaoLing 2011[943]	265	2144	0.12	[0.11; 0.14]
ZhengYuXun 2020[945]	77	1739	0.04	[0.04; 0.06]
ZhongQuanChang 2014[947]	59	280	0.21	[0.16; 0.26]
ZhongYanXu 2017[949]	577	2236	0.26	[0.24; 0.28]
ZhouHuiFang 2018[953]	65	439	0.15	[0.12; 0.18]
ZhouMingLi 2017[957]	22	132	0.17	[0.11; 0.24]
ZhouYinZhu 2018[963]	72	547	0.13	[0.10; 0.16]
ZhuFang 2017[969]	11	290	0.04	[0.02; 0.07]
ZhuHuiLin 2020[972]	41	467	0.09	[0.06; 0.12]
ZhuMin 2019[974]	160	530	0.30	[0.26; 0.34]
ZhuXiaoLu 2018[977]	129	1565	0.08	[0.07; 0.10]
ZhuXun 2019[978]	306	9047	0.03	[0.03; 0.04]
ZouHuiYing 2016[981]	310	2132	0.15	[0.13; 0.16]
ZouYongWen 2021[982]	76	1357	0.06	[0.04; 0.07]
Cao RR 2021[984]	242	1181	0.20	[0.18; 0.23]
Chang H 2017[985]	123	1360	0.09	[0.08; 0.11]
Chen C 2020[986]	134	1849	0.07	[0.06; 0.09]
Chen H 2015[988]	90	529	0.17	[0.14; 0.20]
Chen Y 2013[990]	102	811	0.13	[0.10; 0.15]
Fu JG 2016[996]	164	413	0.40	[0.35; 0.45]
Gong XH 2018[999]	1744	8797	0.20	[0.19; 0.21]
Han J 2015[1002]	193	809	0.24	[0.21; 0.27]
Han J 2018[1003]	204	1001	0.20	[0.18; 0.23]
Ji L 2020[1009]	100	551	0.18	[0.15; 0.22]
Kuang X 2019[1013]	1077	7883	0.14	[0.13; 0.14]
Li J 2018[1016]	64	424	0.15	[0.12; 0.19]
Lu L 2015[1026]	126	436	0.29	[0.25; 0.33]
Lu L 2019[1027]	220	1433	0.15	[0.14; 0.17]
Lu QB 2015[1028]	1120	2140	0.52	[0.50; 0.54]
Pan L 2016[1033]	539	2169	0.25	[0.23; 0.27]
Ren Z 2013[1038]	130	500	0.26	[0.22; 0.30]

H. Shen 2016[1042]	34	412	0.08	[0.06; 0.11]
Shen W 2020[1043]	139	1464	0.09	[0.08; 0.11]
Shen Z 2013[1045]	37	748	0.05	[0.04; 0.07]
Sun XM 2016[1048−2]	5	222	0.02	[0.01; 0.05]
Tan D 2015[1049]	101	354	0.29	[0.24; 0.34]
ZhangJianYing 2020[1051]	28	120	0.23	[0.16; 0.32]
Wang X 2016[1053]	209	1681	0.12	[0.11; 0.14]
Wang X 2019[1054]	510	3421	0.15	[0.14; 0.16]
ZhangXiaoYan 2021[1056]	42	116	0.36	[0.27; 0.46]
W Wu 2014[1057]	210	983	0.21	[0.19; 0.24]
Wu X 2015[1058]	211	796	0.27	[0.23; 0.30]
C Xue 2018[1061]	1363	5927	0.23	[0.22; 0.24]
Xue L 2019[1062]	43	217	0.20	[0.15; 0.26]
Xue L 2016[1063]	25	215	0.12	[0.08; 0.17]
Xue L 2013[1064]	9	89	0.10	[0.05; 0.18]
Xue Y 2015[1065]	903	2114	0.43	[0.41; 0.45]
Yang F 2018[1066]	22	412	0.05	[0.03; 0.08]
Yu J 2017[1069]	771	3877	0.20	[0.19; 0.21]
Zhang DM 2015[1070]	6	1128	0.01	[0.00; 0.01]
Zhang P 2016[1072]	196	746	0.26	[0.23; 0.30]
Zhang SX 2016[1074]	31	420	0.07	[0.05; 0.10]
Zhu XH 2016[1082]	88	381	0.23	[0.19; 0.28]
Zhu YN 2020[1083]	197	1220	0.16	[0.14; 0.18]
ZL Zhuang 2017[1084]	32	295	0.11	[0.08; 0.15]
Zou W 2015[1085]	383	1346	0.28	[0.26; 0.31]
PangBeiBei 2012[1089]	164	753	0.22	[0.19; 0.25]
WangGuoZheng 2014[1090]	49	1134	0.04	[0.03; 0.06]
XuShaoJian 2015[1092]	10	205	0.05	[0.02; 0.09]
YaoWeiLei 2013[1093]	531	2290	0.23	[0.21; 0.25]
ChenFengQin 2014[1095]	204	594	0.34	[0.31; 0.38]
LuXueDong 2012[1098]	38	528	0.07	[0.05; 0.10]
SunLiMei 2014[1099]	326	1932	0.17	[0.15; 0.19]
WangQiong 2013[1100]	38	307	0.12	[0.09; 0.17]
Xiao−Ling Zhu Ge 2011[1102]	191	920	0.21	[0.18; 0.24]
ZengHao 2019[1104]	493	96732	0.01	[0.00; 0.01]
ChenCen 2016[1105]	52	485	0.11	[0.08; 0.14]
HanNing 2016[1107]	55	324	0.17	[0.13; 0.22]
HeYaQing 2017[1109]	1613	8241	0.20	[0.19; 0.20]
HuTingTing 2013[1110]	32	360	0.09	[0.06; 0.12]
HuangYeWei 2018[1112]	552	2402	0.23	[0.21; 0.25]
JiXinFeng 2012[1113]	41	383	0.11	[0.08; 0.14]
LiuCheng 2017[1115]	277	1214	0.23	[0.20; 0.25]
LuoCan 2011[1117]	28	241	0.12	[0.08; 0.16]
QianMingMing 2015[1119]	85	648	0.13	[0.11; 0.16]
ShenZhen 2014[1122]	139	846	0.16	[0.14; 0.19]
WangPeng 2015[1127]	32	295	0.11	[0.08; 0.15]
WangYanGe 2011[1128]	70	852	0.08	[0.06; 0.10]
WuWei 2013[1130]	186	925	0.20	[0.18; 0.23]
XueChaoChao 2017[1131]	3	183	0.02	[0.00; 0.05]
ZhongXueFei 2014[1132]	119	560	0.21	[0.18; 0.25]
**Common effect model**		**695668**	**0.10**	**[0.10; 0.10]**
**Random effects model**			**0.12**	**[0.11; 0.13]**
Heterogeneity: $I^2 = 99\%$, $\tau^2 = 0.9236$, $p = 0$				
**Common effect model**		**909319**	**0.10**	**[0.10; 0.11]**
**Random effects model**			**0.12**	**[0.11; 0.12]**

0.2 0.6

Heterogeneity: $I^2 = 99\%$, $\tau^2 = 0.9292$, $p = 0$
Test for subgroup differences (common effect): $\chi^2_1 = 234.11$, df = 1 ($p < 0.01$
Test for subgroup differences (random effects): $\chi^2_1 = 0.30$, df = 1 ($p = 0.59$

(a4)

Study	Events	Total	Attack rate	95%−CI
area = North				
BaiAiLi 2020[3]	20	738	0.03	[0.02; 0.04]
BaiYao 2020[6]	22	169	0.13	[0.08; 0.19]
BaiYun 2018[8]	61	648	0.09	[0.07; 0.12]
Cai W 2018[23−2]	38	117	0.32	[0.24; 0.42]
Cai W 2018[23−3]	217	2504	0.09	[0.08; 0.10]
Cai W 2017[24−4]	12	32	0.38	[0.21; 0.56]
Cai W 2017[24−5]	10	32	0.31	[0.16; 0.50]
Cai W 2017[24−6]	24	36	0.67	[0.49; 0.81]
Cai W 2017[24−7]	8	39	0.21	[0.09; 0.36]
Cai W 2017[24−8]	13	40	0.32	[0.19; 0.49]
Cai W 2017[24−9]	12	43	0.28	[0.15; 0.44]
Cai W 2017[24−10]	17	49	0.35	[0.22; 0.50]
Cai W 2017[24−11]	10	67	0.15	[0.07; 0.26]
Cai W 2017[24−12]	16	78	0.21	[0.12; 0.31]
CaiXiuZhi 2018[27−1]	34	150	0.23	[0.16; 0.30]
CaiXiuZhi 2018[27−2]	34	400	0.08	[0.06; 0.12]
CaiXiuZhi 2018[27−3]	40	1045	0.04	[0.03; 0.05]
CaiXiuZhi 2018[27−4]	24	1279	0.02	[0.01; 0.03]
ChenQian 2014[95]	78	1844	0.04	[0.03; 0.05]
CuiLiangLiang 2017[123−1]	5	90	0.06	[0.02; 0.12]
CuiLiangLiang 2017[123−2]	14	4600	0.00	[0.00; 0.01]
CuiLiangLiang 2017[123−3]	55	10000	0.01	[0.00; 0.01]
CuiLiangLiang 2017[123−4]	1434	11000	0.13	[0.12; 0.14]
CuiLiangLiang 2017[123−5]	385	15000	0.03	[0.02; 0.03]
CuiLiangLiang 2017[123−6]	235	20000	0.01	[0.01; 0.01]
CuiXiaoMan 2015[124]	34	332	0.10	[0.07; 0.14]
CuiXiaoMan 2018[125]	17	829	0.02	[0.01; 0.03]
DingJianQing 2012[138]	82	10177	0.01	[0.01; 0.01]
FangYunXia 2018[157]	27	357	0.08	[0.05; 0.11]
GaoPeng 2020[181]	28	217	0.13	[0.09; 0.18]
GaoRiHong 2019[182]	30	72	0.42	[0.30; 0.54]
GaoZhiPeng 2018[186]	130	397	0.33	[0.28; 0.38]
GaoZhiYong 2017[187−1]	12	32	0.38	[0.21; 0.56]
GaoZhiYong 2017[187−2]	10	46	0.22	[0.11; 0.36]
GaoZhiYong 2017[187−3]	15	57	0.26	[0.16; 0.40]
GaoZhiYong 2017[187−4]	16	63	0.25	[0.15; 0.38]
GaoZhiYong 2017[187−5]	7	78	0.09	[0.04; 0.18]
GaoZhiYong 2017[187−6]	25	953	0.03	[0.02; 0.04]
GaoZhiYong 2017[187−7]	21	980	0.02	[0.01; 0.03]
GaoZhiYong 2017[187−8]	22	1047	0.02	[0.01; 0.03]
GuKaiChen 2020[195]	21	500	0.04	[0.03; 0.06]
GuoJianXin 2015[202−1]	9	34	0.26	[0.13; 0.44]
GuoJianXin 2015[202−2]	20	43	0.47	[0.31; 0.62]
GuoJianXin 2015[202−3]	15	142	0.11	[0.06; 0.17]
GuoJianXin 2015[202−4]	22	172	0.13	[0.08; 0.19]
GuoJianXin 2015[202−5]	53	350	0.15	[0.12; 0.19]
GuoJianXin 2015[202−6]	55	611	0.09	[0.07; 0.12]
GuoLi 2019[205]	75	456	0.16	[0.13; 0.20]
GuoLi 2019[206−15]	9	31	0.29	[0.14; 0.48]
GuoLi 2019[206−16]	8	31	0.26	[0.12; 0.45]
GuoLi 2019[206−17]	10	31	0.32	[0.17; 0.51]
GuoLi 2019[206−18]	14	32	0.44	[0.26; 0.62]
GuoLi 2019[206−19]	9	33	0.27	[0.13; 0.46]
GuoLi 2019[206−20]	18	33	0.55	[0.36; 0.72]
GuoLi 2019[206−21]	8	34	0.24	[0.11; 0.41]
GuoLi 2019[206−22]	10	34	0.29	[0.15; 0.47]
GuoLi 2019[206−23]	14	34	0.41	[0.25; 0.59]
GuoLi 2019[206−24]	18	35	0.51	[0.34; 0.69]

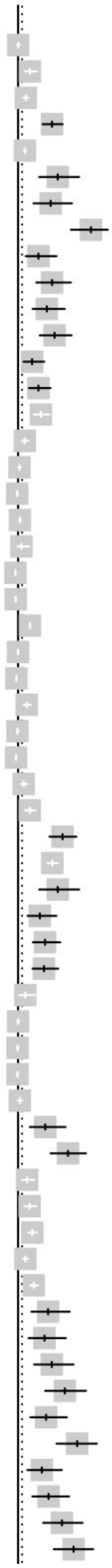

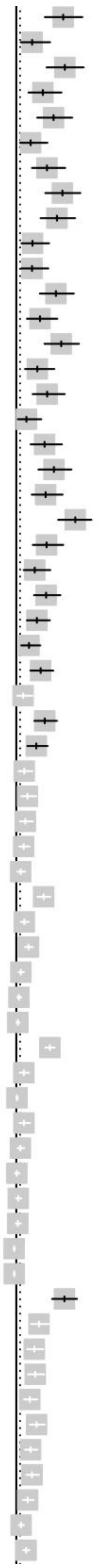

GuoLi 2019[206−25]	16	35	0.46	[0.29; 0.63]
GuoLi 2019[206−26]	6	35	0.17	[0.07; 0.34]
GuoLi 2019[206−27]	17	36	0.47	[0.30; 0.65]
GuoLi 2019[206−28]	10	37	0.27	[0.14; 0.44]
GuoLi 2019[206−29]	14	38	0.37	[0.22; 0.54]
GuoLi 2019[206−30]	6	38	0.16	[0.06; 0.31]
GuoLi 2019[206−31]	12	39	0.31	[0.17; 0.48]
GuoLi 2019[206−32]	18	40	0.45	[0.29; 0.62]
GuoLi 2019[206−33]	16	40	0.40	[0.25; 0.57]
GuoLi 2019[206−34]	7	40	0.17	[0.07; 0.33]
GuoLi 2019[206−35]	7	41	0.17	[0.07; 0.32]
GuoLi 2019[206−36]	16	41	0.39	[0.24; 0.55]
GuoLi 2019[206−37]	10	41	0.24	[0.12; 0.40]
GuoLi 2019[206−38]	18	41	0.44	[0.28; 0.60]
GuoLi 2019[206−39]	9	41	0.22	[0.11; 0.38]
GuoLi 2019[206−40]	13	42	0.31	[0.18; 0.47]
GuoLi 2019[206−41]	5	42	0.12	[0.04; 0.26]
GuoLi 2019[206−42]	12	42	0.29	[0.16; 0.45]
GuoLi 2019[206−43]	16	43	0.37	[0.23; 0.53]
GuoLi 2019[206−44]	13	44	0.30	[0.17; 0.45]
GuoLi 2019[206−45]	25	44	0.57	[0.41; 0.72]
GuoLi 2019[206−46]	14	46	0.30	[0.18; 0.46]
GuoLi 2019[206−47]	9	46	0.20	[0.09; 0.34]
GuoLi 2019[206−48]	18	60	0.30	[0.19; 0.43]
GuoLi 2019[206−49]	14	65	0.22	[0.12; 0.33]
GuoLi 2019[206−50]	10	70	0.14	[0.07; 0.25]
GuoLi 2019[206−51]	18	72	0.25	[0.16; 0.37]
GuoLi 2019[206−52]	7	79	0.09	[0.04; 0.17]
GuoLi 2019[206−53]	23	80	0.29	[0.19; 0.40]
GuoLi 2019[206−54]	17	81	0.21	[0.13; 0.31]
GuoLi 2019[206−55]	10	101	0.10	[0.05; 0.17]
GuoLi 2019[206−56]	13	101	0.13	[0.07; 0.21]
GuoLi 2019[206−57]	13	119	0.11	[0.06; 0.18]
GuoLi 2019[206−58]	16	171	0.09	[0.05; 0.15]
GuoLi 2019[206−59]	14	211	0.07	[0.04; 0.11]
GuoLi 2019[206−60]	61	220	0.28	[0.22; 0.34]
GuoLi 2019[206−61]	28	281	0.10	[0.07; 0.14]
GuoLi 2019[206−62]	47	339	0.14	[0.10; 0.18]
GuoLi 2019[206−63]	30	440	0.07	[0.05; 0.10]
GuoLi 2019[206−64]	25	498	0.05	[0.03; 0.07]
GuoLi 2019[206−65]	28	698	0.04	[0.03; 0.06]
HeXuXin 2017[222]	156	466	0.33	[0.29; 0.38]
HuGuangYi 2017[232]	20	210	0.10	[0.06; 0.14]
HuaWeiYu 2018[241]	63	1951	0.03	[0.02; 0.04]
HuaWeiYu 2018[242−1]	15	156	0.10	[0.05; 0.15]
HuaWeiYu 2018[242−2]	17	267	0.06	[0.04; 0.10]
HuaWeiYu 2018[242−3]	9	290	0.03	[0.01; 0.06]
HuaWeiYu 2018[242−4]	14	320	0.04	[0.02; 0.07]
HuaWeiYu 2018[242−5]	13	325	0.04	[0.02; 0.07]
HuaWeiYu 2018[242−6]	5	833	0.01	[0.00; 0.01]
HuaWeiYu 2018[242−7]	13	1806	0.01	[0.00; 0.01]
HuangYanHong 2019[259−2]	37	79	0.47	[0.36; 0.58]
HuangYanHong 2019[259−3]	27	115	0.23	[0.16; 0.32]
HuangYanHong 2019[259−4]	23	120	0.19	[0.13; 0.27]
HuangYanHong 2019[259−5]	24	120	0.20	[0.13; 0.28]
HuangYanHong 2019[259−6]	21	140	0.15	[0.10; 0.22]
HuangYanHong 2019[259−7]	31	146	0.21	[0.15; 0.29]
HuangYanHong 2019[259−8]	27	172	0.16	[0.11; 0.22]
HuangYanHong 2019[259−9]	30	176	0.17	[0.12; 0.23]
HuangYanHong 2019[259−10]	26	199	0.13	[0.09; 0.19]
HuangYanHong 2019[259−11]	32	456	0.07	[0.05; 0.10]
HuangYanHong 2019[259−12]	58	498	0.12	[0.09; 0.15]

HuangYanHong 2019[260]	84	760	0.11 [0.09; 0.14]
HuangYanHong 2019[261]	86	1190	0.07 [0.06; 0.09]
JiangXiHong 2019[293]	40	304	0.13 [0.10; 0.17]
JinXiaoFang 2015[302]	38	281	0.14 [0.10; 0.18]
KangQian 2020[303−1]	35	736	0.05 [0.03; 0.07]
KangQian 2020[303−2]	50	1622	0.03 [0.02; 0.04]
LiBing 2019[315]	67	2354	0.03 [0.02; 0.04]
LiCaiYun 2012[318]	147	940	0.16 [0.13; 0.18]
LiChunLing 2020[319]	15	196	0.08 [0.04; 0.12]
LiJiShan 2018[324−2]	7	40	0.17 [0.07; 0.33]
LiJiShan 2018[324−3]	7	78	0.09 [0.04; 0.18]
LiJiShan 2016[326−1]	16	35	0.46 [0.29; 0.63]
LiJiShan 2016[326−2]	18	41	0.44 [0.28; 0.60]
LiJiShan 2016[326−3]	8	46	0.17 [0.08; 0.31]
LiJun 2012[334]	1252	2877	0.44 [0.42; 0.45]
LiShiE 2018[347−1]	6	36	0.17 [0.06; 0.33]
LiShiE 2018[347−2]	15	38	0.39 [0.24; 0.57]
LiShiE 2018[347−3]	16	48	0.33 [0.20; 0.48]
LiShiE 2018[347−4]	22	66	0.33 [0.22; 0.46]
LiShiE 2018[347−5]	37	89	0.42 [0.31; 0.53]
LiShiE 2018[347−6]	14	94	0.15 [0.08; 0.24]
LiShiE 2018[347−7]	24	126	0.19 [0.13; 0.27]
LiShiE 2018[347−8]	34	173	0.20 [0.14; 0.26]
LiShiE 2018[347−9]	10	221	0.05 [0.02; 0.08]
LiShiE 2018[348]	92	1419	0.06 [0.05; 0.08]
LiShiE 2018[349]	77	8153	0.01 [0.01; 0.01]
LiXiTai 2015[352]	51	1336	0.04 [0.03; 0.05]
LiYan 2017[361]	93	2355	0.04 [0.03; 0.05]
LiangPing 2020[374]	25	196	0.13 [0.08; 0.18]
LiuBaiWei 2017[395]	24	471	0.05 [0.03; 0.07]
LiuBaiWei 2017[396−1]	43	222	0.19 [0.14; 0.25]
LiuBaiWei 2017[396−2]	172	3546	0.05 [0.04; 0.06]
LiuBaiWei 2017[396−3]	58	4500	0.01 [0.01; 0.02]
LiuCaiXia 2019[399−3]	20	208	0.10 [0.06; 0.14]
LiuXiaoXiao 2014[430]	18	70	0.26 [0.16; 0.38]
LiuXiuMei 2017[433]	65	1134	0.06 [0.04; 0.07]
LiuYuan 2016[438−1]	15	142	0.11 [0.06; 0.17]
LiuYuan 2016[438−2]	121	378	0.32 [0.27; 0.37]
LiuYuan 2016[438−3]	55	611	0.09 [0.07; 0.12]
LuXiuZhi 2018[463]	47	3600	0.01 [0.01; 0.02]
LuoQiong 2019[474]	22	2256	0.01 [0.01; 0.01]
NiChunYan 2020[512]	55	491	0.11 [0.09; 0.14]
PanYueFei 2020[522]	45	215	0.21 [0.16; 0.27]
QiYing 2018[535−1]	173	1621	0.11 [0.09; 0.12]
QiYing 2018[535−2]	71	1621	0.04 [0.03; 0.05]
QiYing 2019[535−3]	16	38	0.42 [0.26; 0.59]
QiYing 2019[535−4]	17	39	0.44 [0.28; 0.60]
QiYing 2019[535−5]	13	40	0.32 [0.19; 0.49]
QiYing 2019[535−6]	8	83	0.10 [0.04; 0.18]
QiYing 2019[535−7]	8	129	0.06 [0.03; 0.12]
QiYing 2019[535−8]	10	148	0.07 [0.03; 0.12]
QiYing 2019[535−9]	34	1015	0.03 [0.02; 0.05]
QiYing 2019[535−10]	72	1192	0.06 [0.05; 0.08]
QiYing 2019[535−11]	159	1448	0.11 [0.09; 0.13]
QinDi 2016[544]	16	255	0.06 [0.04; 0.10]
QinLianYang 2021[545]	38	1697	0.02 [0.02; 0.03]
QinMeng 2015[547−1]	35	217	0.16 [0.11; 0.22]
QinMeng 2015[547−2]	25	228	0.11 [0.07; 0.16]
RenLiJun 2020[556]	9	253	0.04 [0.02; 0.07]
RenQiZhi 2018[557−1]	6	100	0.06 [0.02; 0.13]
RenQiZhi 2018[557−2]	12	150	0.08 [0.04; 0.14]
RenQiZhi 2018[557−3]	8	151	0.05 [0.02; 0.10]

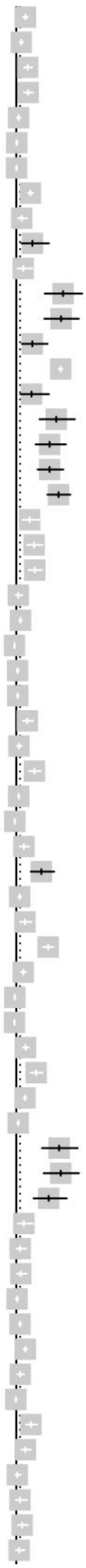

RenQiZhi 2018[557−4]	15	200	0.07 [0.04; 0.12]
RenQiZhi 2018[557−5]	5	2000	0.00 [0.00; 0.01]
RenQiZhi 2018[557−6]	13	2500	0.01 [0.00; 0.01]
RenQiZhi 2018[557−7]	15	10000	0.00 [0.00; 0.00]
SongHuiRong 2017[597]	34	568	0.06 [0.04; 0.08]
SongJie 2014[599]	17	350	0.05 [0.03; 0.08]
SuTong 2020[604]	55	3432	0.02 [0.01; 0.02]
SunHaiBo 2012[608]	66	100177	0.00 [0.00; 0.00]
SunWenLong 2018[618]	8	142	0.06 [0.02; 0.11]
TianJing 2017[641]	39	392	0.10 [0.07; 0.13]
TianYaLin 2021[642]	38	546	0.07 [0.05; 0.09]
WangBing 2017[649]	58	20000	0.00 [0.00; 0.00]
WangMingLiang 2020[671]	107	550	0.19 [0.16; 0.23]
WangXiaoDong 2017[688]	69	2708	0.03 [0.02; 0.03]
WeiYiYun 2015[705]	22	155	0.14 [0.09; 0.21]
WeiXia 2019[708]	22	1024	0.02 [0.01; 0.03]
XieBin 2018[757]	95	5000	0.02 [0.02; 0.02]
XieBin 2020[758]	65	2036	0.03 [0.02; 0.04]
XingYan 2017[759]	119	3500	0.03 [0.03; 0.04]
XuYan 2018[772]	68	969	0.07 [0.05; 0.09]
XuJun 2018[779]	75	2679	0.03 [0.02; 0.03]
YanGeBin 2017[786−1]	14	35	0.40 [0.24; 0.58]
YanGeBin 2017[786−2]	25	62	0.40 [0.28; 0.54]
YangAiQing 2018[795]	40	355	0.11 [0.08; 0.15]
YangShiYong 2015[811]	28	144	0.19 [0.13; 0.27]
YangTongTong 2018[813]	36	450	0.08 [0.06; 0.11]
YuXiaoYun 2018[845]	78	1007	0.08 [0.06; 0.10]
YuHong 2016[848]	80	1184	0.07 [0.05; 0.08]
ZhangAiHua 2018[861]	19	214	0.09 [0.05; 0.14]
ZhangChong 2015[862]	13	2050	0.01 [0.00; 0.01]
ZhangDianXiang 2012[863]	5	40	0.12 [0.04; 0.27]
ZhangHaiYan 2019[867]	145	738	0.20 [0.17; 0.23]
ZhangHaiYan 2016[868]	78	508	0.15 [0.12; 0.19]
ZhangQin 2020[885]	57	4279	0.01 [0.01; 0.02]
ZhangShuang 2017[888]	28	45	0.62 [0.47; 0.76]
ZhangYan 2017[894−1]	8	37	0.22 [0.10; 0.38]
ZhangYan 2017[894−2]	12	2857	0.00 [0.00; 0.01]
ZhangYanMing 2019[898]	15	236	0.06 [0.04; 0.10]
ZhaoQi 2021[920]	53	4760	0.01 [0.01; 0.01]
ZhaoWeiQin 2020[923]	128	4927	0.03 [0.02; 0.03]
ZhaoWenNa 2021[924]	70	3000	0.02 [0.02; 0.03]
ZhenGuoXin 2020[936]	23	204	0.11 [0.07; 0.16]
ZhenGuoXin 2020[937]	11	204	0.05 [0.03; 0.09]
ZhouGuoYing 2016[952]	12	75	0.16 [0.09; 0.26]
ZhuHaiYang 2016[971]	140	1319	0.11 [0.09; 0.12]
Chen D 2019[987]	209	5043	0.04 [0.04; 0.05]
Xiao GD 2019[995]	12	302	0.04 [0.02; 0.07]
Guo XH 2018[1000]	61	1606	0.04 [0.03; 0.05]
Huang XY 2017[1007]	753	22861	0.03 [0.03; 0.04]
Zhang L 2018[1071]	631	15118	0.04 [0.04; 0.05]
**Common effect model**		**388443**	**0.03 [0.03; 0.03]**
**Random effects model**			**0.10 [0.09; 0.12]**
Heterogeneity: $I^2 = 99\%$, $\tau^2 = 1.8718$, $p = 0$			
**area = South**			
BiHua 2014[10]	95	18930	0.01 [0.00; 0.01]
BiHua 2015[11]	55	1262	0.04 [0.03; 0.06]
CaiJian 2013[14−1]	53	2219	0.02 [0.02; 0.03]
CaiJian 2013[14−2]	257	16056	0.02 [0.01; 0.02]
Ming−Wei Cai 2021[17−1]	25	628	0.04 [0.03; 0.06]
Ming−Wei Cai 2021[17−2]	50	693	0.07 [0.05; 0.09]
Ming−Wei Cai 2021[17−3]	27	816	0.03 [0.02; 0.05]

Ming−Wei Cai 2021[17−4]	56	1202	0.05	[0.04; 0.06]
Cai MW 2018[18]	34	223	0.15	[0.11; 0.21]
Cai SJ 2018[20]	69	1781	0.04	[0.03; 0.05]
Cai SX 2017[21]	17	1276	0.01	[0.01; 0.02]
CaiWenFeng 2013[25]	141	16600	0.01	[0.01; 0.01]
CaiWenFeng 2014[26]	27	816	0.03	[0.02; 0.05]
Cao RR 2020[30−1]	41	2853	0.01	[0.01; 0.02]
CaoShen 2019[32]	62	6966	0.01	[0.01; 0.01]
CaoXiaoPing 2018[33]	113	1037	0.11	[0.09; 0.13]
CenYongZhuang 2014[39]	32	106	0.30	[0.22; 0.40]
ZengFengMei 2020[41]	25	255	0.10	[0.06; 0.14]
ZengLei 2018[44]	28	388	0.07	[0.05; 0.10]
ChaRiSheng 2016[46]	22	453	0.05	[0.03; 0.07]
ChaRiSheng 2014[47−1]	36	170	0.21	[0.15; 0.28]
ChaRiSheng 2014[47−2]	29	498	0.06	[0.04; 0.08]
ChaRiSheng 2014[47−3]	43	594	0.07	[0.05; 0.10]
ChaRiSheng 2014[47−4]	18	1111	0.02	[0.01; 0.03]
ChaRiSheng 2014[47−5]	16	1168	0.01	[0.01; 0.02]
ChaRiSheng 2014[47−6]	31	1422	0.02	[0.01; 0.03]
ChaRiSheng 2014[47−7]	74	1749	0.04	[0.03; 0.05]
ChaRiSheng 2014[47−8]	47	1858	0.03	[0.02; 0.03]
ChaRiSheng 2014[47−9]	17	1910	0.01	[0.01; 0.01]
ChaRiSheng 2014[47−10]	168	2002	0.08	[0.07; 0.10]
ChaRiSheng 2014[47−11]	61	2202	0.03	[0.02; 0.04]
ChaRiSheng 2014[47−12]	139	2786	0.05	[0.04; 0.06]
ChaRiSheng 2014[47−13]	39	3095	0.01	[0.01; 0.02]
ShenJiChuan 2011[50]	312	1736	0.18	[0.16; 0.20]
ShenYiPing 2018[52]	26	612	0.04	[0.03; 0.06]
ShenYiPing 2013[53]	65	1900	0.03	[0.03; 0.04]
ShenYuGang 2016[54]	43	480	0.09	[0.07; 0.12]
ShenYuGang 2016[55]	233	1702	0.14	[0.12; 0.15]
ChenAQun 2015[58]	64	19153	0.00	[0.00; 0.00]
ChenAQun 2016[59]	64	18924	0.00	[0.00; 0.00]
ChenBinBin 2017[60−1]	54	392	0.14	[0.11; 0.18]
ChenBinBin 2017[60−2]	161	2122	0.08	[0.06; 0.09]
ChenCaiRong 2020[62]	16	275	0.06	[0.03; 0.09]
ChenCan 2018[63]	24	61	0.39	[0.27; 0.53]
ChenChun 2014[64]	107	1242	0.09	[0.07; 0.10]
ChenGuoCui 2011[69]	27	34	0.79	[0.62; 0.91]
ChenHeJuan 2018[73]	14	49	0.29	[0.17; 0.43]
ChenJian 2016[78]	20	1551	0.01	[0.01; 0.02]
ChenJianMei 2017[79]	110	9736	0.01	[0.01; 0.01]
ChenJian 2017[80]	69	6323	0.01	[0.01; 0.01]
ChenJingFang 2018[84−1]	18	316	0.06	[0.03; 0.09]
ChenJingFang 2018[84−2]	14	360	0.04	[0.02; 0.06]
ChenMinHong 2017[92−1]	21	236	0.09	[0.06; 0.13]
ChenMinHong 2017[92−2]	186	657	0.28	[0.25; 0.32]
ChenMinHong 2017[92−3]	60	4286	0.01	[0.01; 0.02]
ChenQuan 2021[96]	84	821	0.10	[0.08; 0.13]
ChenXiaoFeng 2015[100]	7	648	0.01	[0.00; 0.02]
ChenXin 2020[101]	3	169	0.02	[0.00; 0.05]
ChenXingHong 2012[102]	478	7113	0.07	[0.06; 0.07]
ChenXingFu 2018[103]	43	1240	0.03	[0.03; 0.05]
ChenYan 2019[104]	59	536	0.11	[0.08; 0.14]
ChenYiXiong 2018[108]	21	1861	0.01	[0.01; 0.02]
ChenYiYi 2015[109]	282	38001	0.01	[0.01; 0.01]
ChenZhiQiong 2017[115]	255	1843	0.14	[0.12; 0.15]
ChuXiuJuan 2015[120]	17	147	0.12	[0.07; 0.18]
DaiBenNa 2020[127]	117	3119	0.04	[0.03; 0.04]
DaiYingXue 2018[129−1]	14	378	0.04	[0.02; 0.06]
DaiYingXue 2018[129−2]	12	547	0.02	[0.01; 0.04]
DaiYingXue 2018[129−3]	18	1008	0.02	[0.01; 0.03]

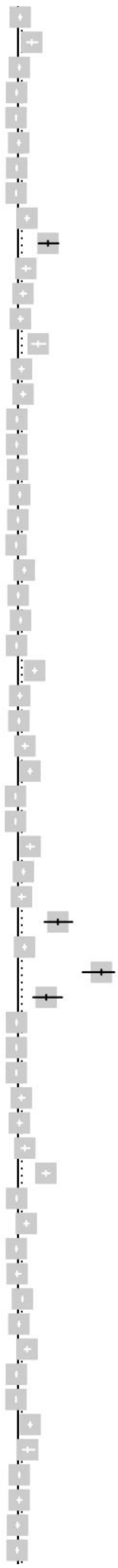

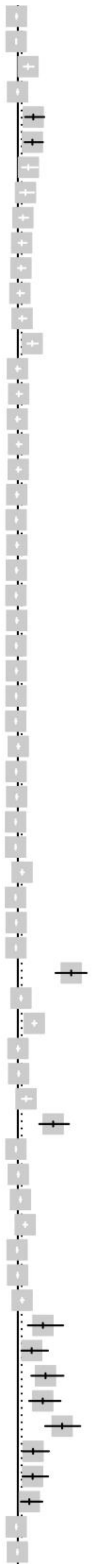

DaiYingXue 2018[129−4]	14	1053	0.01	[0.01; 0.02]
DengXingChao 2019[133]	439	42403	0.01	[0.01; 0.01]
DongShengCao 2017[146]	24	200	0.12	[0.08; 0.17]
DuYueHe 2019[148]	29	1250	0.02	[0.02; 0.03]
DuanRong 2017[152−1]	13	77	0.17	[0.09; 0.27]
DuanRong 2017[152−2]	13	81	0.16	[0.09; 0.26]
DuanRong 2017[152−3]	11	89	0.12	[0.06; 0.21]
DuanRong 2017[152−4]	9	93	0.10	[0.05; 0.18]
DuanRong 2017[152−5]	12	165	0.07	[0.04; 0.12]
DuanRong 2017[152−6]	11	179	0.06	[0.03; 0.11]
DuanRong 2017[152−7]	10	182	0.05	[0.03; 0.10]
DuanRong 2017[152−8]	9	199	0.05	[0.02; 0.08]
DuanRong 2017[152−9]	14	215	0.07	[0.04; 0.11]
DuanRong 2017[152−10]	36	227	0.16	[0.11; 0.21]
DuanRong 2017[152−11]	5	229	0.02	[0.01; 0.05]
DuanRong 2017[152−12]	8	238	0.03	[0.01; 0.07]
DuanRong 2017[152−13]	5	245	0.02	[0.01; 0.05]
DuanRong 2017[152−14]	8	249	0.03	[0.01; 0.06]
DuanRong 2017[152−15]	8	271	0.03	[0.01; 0.06]
DuanRong 2017[152−16]	7	441	0.02	[0.01; 0.03]
DuanRong 2017[152−17]	5	495	0.01	[0.00; 0.02]
DuanRong 2017[152−18]	9	538	0.02	[0.01; 0.03]
DuanRong 2017[152−19]	7	565	0.01	[0.00; 0.03]
DuanRong 2017[152−20]	5	590	0.01	[0.00; 0.02]
DuanRong 2017[152−21]	11	699	0.02	[0.01; 0.03]
DuanRong 2017[152−22]	7	701	0.01	[0.00; 0.02]
DuanRong 2017[152−23]	8	706	0.01	[0.00; 0.02]
DuanRong 2017[152−24]	7	733	0.01	[0.00; 0.02]
DuanRong 2017[152−25]	5	753	0.01	[0.00; 0.02]
DuanRong 2017[152−26]	24	802	0.03	[0.02; 0.04]
DuanRong 2017[152−27]	9	918	0.01	[0.00; 0.02]
DuanRong 2017[152−28]	14	927	0.02	[0.01; 0.03]
DuanRong 2017[152−29]	5	976	0.01	[0.00; 0.01]
DuanRong 2017[152−30]	5	976	0.01	[0.00; 0.01]
DuanRong 2017[152−31]	68	1036	0.07	[0.05; 0.08]
DuanRong 2017[152−32]	5	1145	0.00	[0.00; 0.01]
DuanRong 2017[152−33]	13	1216	0.01	[0.01; 0.02]
DuanRong 2017[152−34]	11	1420	0.01	[0.00; 0.01]
FengZhi 2020[161]	26	50	0.52	[0.37; 0.66]
FengZhi 2018[162]	111	2020	0.05	[0.05; 0.07]
FuJianGuang 2013[163]	276	1546	0.18	[0.16; 0.20]
FuXiaoFei 2012[171]	20	728	0.03	[0.02; 0.04]
GanXiangYang 2014[172]	74	2200	0.03	[0.03; 0.04]
GaoHaiMing 2014[174]	19	187	0.10	[0.06; 0.15]
GaoHuiJuan 2015[176]	18	51	0.35	[0.22; 0.50]
GaoJunYing 2017[177]	12	1501	0.01	[0.00; 0.01]
GaoShuPing 2019[183]	51	1698	0.03	[0.02; 0.04]
GongLiQiang 2013[192]	139	2787	0.05	[0.04; 0.06]
GongShuiYing 2017[194]	93	990	0.09	[0.08; 0.11]
GuShiPing 2009[196]	42	2199	0.02	[0.01; 0.03]
GuYiFu 2020[197]	45	1903	0.02	[0.02; 0.03]
GuiGuoPing 2018[200]	79	1204	0.07	[0.05; 0.08]
GuoMinJian 2017[208−1]	8	31	0.26	[0.12; 0.45]
GuoMinJian 2017[208−2]	6	39	0.15	[0.06; 0.31]
GuoMinJian 2017[208−3]	11	39	0.28	[0.15; 0.45]
GuoMinJian 2017[208−4]	10	39	0.26	[0.13; 0.42]
GuoMinJian 2017[208−5]	17	39	0.44	[0.28; 0.60]
GuoMinJian 2017[208−6]	7	42	0.17	[0.07; 0.31]
GuoMinJian 2017[208−7]	7	43	0.16	[0.07; 0.31]
GuoMinJian 2017[208−8]	7	53	0.13	[0.05; 0.25]
GuoMinJian 2017[208−9]	16	1488	0.01	[0.01; 0.02]
GuoMinJian 2017[208−10]	48	2200	0.02	[0.02; 0.03]

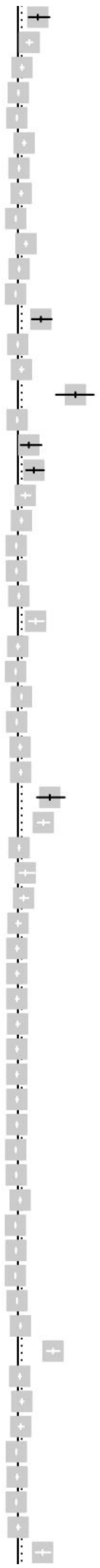

GuoShuiLian 2014[209]	16	76	0.21	[0.13; 0.32]
HeHanZhen 2014[224]	76	578	0.13	[0.11; 0.16]
HouYuYuan 2014[231]	63	984	0.06	[0.05; 0.08]
HuHongAn 2015[233]	105	3453	0.03	[0.02; 0.04]
HuangBinBin 2020[245]	41	2530	0.02	[0.01; 0.02]
HuangChunLi 2015[246]	108	1283	0.08	[0.07; 0.10]
HuangGe 2013[251]	92	2527	0.04	[0.03; 0.04]
HuangGuo 2015[252]	69	1220	0.06	[0.04; 0.07]
HuangGuo 2015[253]	87	13856	0.01	[0.01; 0.01]
HuangLiQing 2020[254]	184	1823	0.10	[0.09; 0.12]
HuangShiTeng 2021[256]	61	1644	0.04	[0.03; 0.05]
HuangSiYue 2020[257]	90	19800	0.00	[0.00; 0.01]
HuangZhongXue 2013[263]	22	92	0.24	[0.16; 0.34]
JiHong 2015[264]	78	3194	0.02	[0.02; 0.03]
JiJinHua 2018[265]	41	694	0.06	[0.04; 0.08]
JiLei 2020[270]	19	34	0.56	[0.38; 0.73]
Lei Ji 2011[271]	42	2210	0.02	[0.01; 0.03]
JiLei 2018[272−1]	7	55	0.13	[0.05; 0.24]
JiLei 2018[272−2]	16	92	0.17	[0.10; 0.27]
JiLei 2018[272−3]	18	191	0.09	[0.06; 0.14]
JiRuPing 2020[274]	119	2030	0.06	[0.05; 0.07]
JiangXianChen 2014[283−1]	16	1485	0.01	[0.01; 0.02]
JiangXianChen 2014[283−2]	23	1877	0.01	[0.01; 0.02]
JiangYingCi 2015[284]	66	1904	0.03	[0.03; 0.04]
JiangChen 2019[285]	26	137	0.19	[0.13; 0.27]
JiangLie 2014[291]	15	590	0.03	[0.01; 0.04]
JiangYiMei 2017[294]	15	3050	0.00	[0.00; 0.01]
KuangHaoCheng 2016[307]	667	11300	0.06	[0.05; 0.06]
LaiShiMing 2014[308]	105	7113	0.01	[0.01; 0.02]
LeiYongLiang 2016[311]	46	984	0.05	[0.03; 0.06]
LiBo 2013[316]	120	2303	0.05	[0.04; 0.06]
LiDaiBo 2018[320]	18	56	0.32	[0.20; 0.46]
LiJianSen 2015[328]	70	268	0.26	[0.21; 0.32]
LiJie 2019[329]	106	2868	0.04	[0.03; 0.04]
LiMeng 2020[338−1]	7	74	0.09	[0.04; 0.19]
LiMeng 2020[338−2]	16	204	0.08	[0.05; 0.12]
LiMeng 2020[338−3]	7	260	0.03	[0.01; 0.05]
LiMeng 2020[338−4]	6	358	0.02	[0.01; 0.04]
LiMeng 2020[338−5]	6	369	0.02	[0.01; 0.04]
LiMeng 2020[338−6]	7	416	0.02	[0.01; 0.03]
LiMeng 2020[338−7]	11	476	0.02	[0.01; 0.04]
LiMeng 2020[338−8]	11	659	0.02	[0.01; 0.03]
LiMeng 2020[338−9]	12	749	0.02	[0.01; 0.03]
LiMeng 2020[338−10]	13	754	0.02	[0.01; 0.03]
LiMeng 2020[338−11]	19	1168	0.02	[0.01; 0.03]
LiMeng 2020[338−12]	9	1462	0.01	[0.00; 0.01]
LiMeng 2020[338−13]	17	1580	0.01	[0.01; 0.02]
LiMeng 2020[338−14]	85	1814	0.05	[0.04; 0.06]
LiMeng 2020[338−15]	6	1863	0.00	[0.00; 0.01]
LiMeng 2020[338−16]	17	2026	0.01	[0.00; 0.01]
LiMeng 2020[338−17]	5	2026	0.00	[0.00; 0.01]
LiQun 2010[340]	258	14269	0.02	[0.02; 0.02]
LiShiCong 2020[344]	181	3622	0.05	[0.04; 0.06]
LiShiCong 2019[345]	91	259	0.35	[0.29; 0.41]
LiShiCong 2018[346]	64	1487	0.04	[0.03; 0.05]
LiShouJun 2016[350]	64	1023	0.06	[0.05; 0.08]
LiXiuFang 2018[359]	20	378	0.05	[0.03; 0.08]
LiYiLan 2014[365]	228	18930	0.01	[0.01; 0.01]
LiYueRong 2015[366]	18	952	0.02	[0.01; 0.03]
LiangRiCheng 2017[376]	27	2518	0.01	[0.01; 0.02]
LiaoChan 2021[380]	19	675	0.03	[0.02; 0.04]
LiaoKeChang 2018[381]	41	161	0.25	[0.19; 0.33]

LinJian 2019[384]	77	2476	0.03	[0.02; 0.04]
LinQiFeng 2018[388]	96	1494	0.06	[0.05; 0.08]
LinQin 2015[390]	34	713	0.05	[0.03; 0.07]
LinQingShuang 2016[391]	29	603	0.05	[0.03; 0.07]
LinYanYan 2018[393]	76	3288	0.02	[0.02; 0.03]
LiuBo 2015[398]	104	1883	0.06	[0.05; 0.07]
LiuDan 2019[403]	90	951	0.09	[0.08; 0.12]
LiuDongSheng 2019[404]	80	3720	0.02	[0.02; 0.03]
LiuGuoHong 2014[406]	109	636	0.17	[0.14; 0.20]
LiuHaoHui 2020[410−1]	6	35	0.17	[0.07; 0.34]
LiuHaoHui 2020[410−2]	28	53	0.53	[0.39; 0.67]
LiuHaoHui 2020[410−3]	10	82	0.12	[0.06; 0.21]
LiuHaoHui 2020[410−4]	14	166	0.08	[0.05; 0.14]
LiuHaoHui 2020[410−5]	9	202	0.04	[0.02; 0.08]
LiuHaoHui 2020[410−6]	16	300	0.05	[0.03; 0.09]
LiuHaoHui 2020[410−7]	12	561	0.02	[0.01; 0.04]
LiuHaoHui 2020[410−8]	145	618	0.23	[0.20; 0.27]
LiuHaoHui 2020[410−9]	17	2191	0.01	[0.00; 0.01]
LiuHongLian 2018[411]	19	1119	0.02	[0.01; 0.03]
LiuJingJing 2018[413]	33	111	0.30	[0.21; 0.39]
LiuKaiQian 2011[415]	634	14439	0.04	[0.04; 0.05]
LiuQingLian 2019[420]	37	817	0.05	[0.03; 0.06]
LiuShiKe 2012[421]	306	1539	0.20	[0.18; 0.22]
LiuShiKe 2016[422]	46	948	0.05	[0.04; 0.06]
LiuTian 2019[423]	10	89	0.11	[0.06; 0.20]
LiuTian 2017[424−1]	16	39	0.41	[0.26; 0.58]
LiuTian 2017[424−2]	11	42	0.26	[0.14; 0.42]
LiuTian 2017[424−3]	32	44	0.73	[0.57; 0.85]
LiuTian 2017[424−4]	9	48	0.19	[0.09; 0.33]
LiuTian 2017[424−5]	14	84	0.17	[0.09; 0.26]
LiuTian 2017[424−6]	70	213	0.33	[0.27; 0.40]
LiuTian 2017[424−7]	35	387	0.09	[0.06; 0.12]
LiuWenJun 2018[428−1]	18	255	0.07	[0.04; 0.11]
LiuWenJun 2018[428−2]	22	388	0.06	[0.04; 0.08]
LiuWenJun 2018[428−3]	38	528	0.07	[0.05; 0.10]
LiuWenJun 2018[428−4]	42	565	0.07	[0.05; 0.10]
LiuWenJun 2018[428−5]	48	672	0.07	[0.05; 0.09]
LiuWenJun 2018[428−6]	36	700	0.05	[0.04; 0.07]
LiuWenJun 2018[428−7]	18	826	0.02	[0.01; 0.03]
LiuWenJun 2018[428−8]	16	904	0.02	[0.01; 0.03]
LiuWenJun 2018[428−9]	105	907	0.12	[0.10; 0.14]
LiuWenJun 2018[428−10]	31	951	0.03	[0.02; 0.05]
LiuWenJun 2018[428−11]	55	955	0.06	[0.04; 0.07]
LiuWenJun 2018[428−12]	49	1213	0.04	[0.03; 0.05]
LiuWenJun 2018[428−13]	193	1285	0.15	[0.13; 0.17]
LiuWenJun 2018[428−14]	46	1314	0.04	[0.03; 0.05]
LiuWenJun 2018[428−15]	17	1328	0.01	[0.01; 0.02]
LiuWenJun 2018[428−16]	54	1467	0.04	[0.03; 0.05]
LiuWenJun 2018[428−17]	180	1554	0.12	[0.10; 0.13]
LiuWenJun 2018[428−18]	19	1776	0.01	[0.01; 0.02]
LiuWenJun 2018[428−19]	68	1926	0.04	[0.03; 0.04]
LiuWenJun 2018[428−20]	17	1932	0.01	[0.01; 0.01]
LiuWenJun 2018[428−21]	55	2174	0.03	[0.02; 0.03]
LiuWenJun 2018[428−22]	52	2332	0.02	[0.02; 0.03]
LiuWenJun 2018[428−23]	70	3608	0.02	[0.02; 0.02]
LiuWenJun 2018[428−24]	43	3707	0.01	[0.01; 0.02]
LiuYi 2013[436]	9	35	0.26	[0.12; 0.43]
LiuYing 2019[437]	14	335	0.04	[0.02; 0.07]
LongJunBiao 2014[441]	13	444	0.03	[0.02; 0.05]
LongQiZhi 2016[442]	101	1707	0.06	[0.05; 0.07]
LuZhengXiang 2016[451]	83	4624	0.02	[0.01; 0.02]
LuHua 2016[454]	14	213	0.07	[0.04; 0.11]

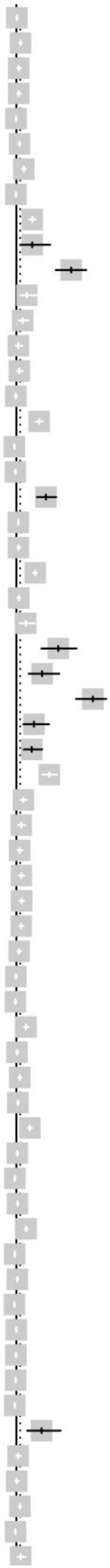

LuJianYong 2015[456]	60	973	0.06	[0.05; 0.08]
LuCaiFang 2020[458]	19	499	0.04	[0.02; 0.06]
LuWeiWei 2016[462]	406	3808	0.11	[0.10; 0.12]
LuoGuiHe 2013[467]	10	422	0.02	[0.01; 0.04]
LuoLe 2017[472]	39	1192	0.03	[0.02; 0.04]
LuoTengXian 2018[475]	13	200	0.06	[0.04; 0.11]
MaMengMeng 2018[484]	223	30711	0.01	[0.01; 0.01]
MaTao 2015[486]	84	901	0.09	[0.08; 0.11]
MaTao 2018[487]	46	587	0.08	[0.06; 0.10]
MaoJianYing 2016[493]	51	559	0.09	[0.07; 0.12]
MengJian 2018[495]	37	465	0.08	[0.06; 0.11]
MengXiangJie 2012[499]	16	34	0.47	[0.30; 0.65]
MoGuiQiong 2016[504]	26	843	0.03	[0.02; 0.04]
MoYuJie 2018[505]	19	1117	0.02	[0.01; 0.03]
MiaoGuoZhong 2018[507]	25	350	0.07	[0.05; 0.10]
NiChaoRong 2019[510]	125	3622	0.03	[0.03; 0.04]
NiChaoRong 2019[511]	28	2158	0.01	[0.01; 0.02]
OuSheXiang 2019[515]	18	603	0.03	[0.02; 0.05]
PanYiFeng 2017[521]	82	7396	0.01	[0.01; 0.01]
PangZhiFeng 2017[524]	79	1696	0.05	[0.04; 0.06]
PangZhiFeng 2014[525]	20	548	0.04	[0.02; 0.06]
PangZhiMing 2015[526]	19	381	0.05	[0.03; 0.08]
PengXiaoXue 2015[528]	37	849	0.04	[0.03; 0.06]
PuPeiLong 2017[529]	98	1126	0.09	[0.07; 0.11]
QiYanQiu 2018[532]	27	570	0.05	[0.03; 0.07]
QiXiaoQi 2019[534]	185	2418	0.08	[0.07; 0.09]
QianLiZhen 2020[537]	23	95	0.24	[0.16; 0.34]
QianZiYu 2012[539]	22	111	0.20	[0.13; 0.28]
QinYanMin 2010[549]	97	1031	0.09	[0.08; 0.11]
QiuHaiYan 2013[551]	76	5965	0.01	[0.01; 0.02]
RenFuLin 2013[554]	43	594	0.07	[0.05; 0.10]
RenFuLin 2013[555]	74	1751	0.04	[0.03; 0.05]
RenYuHua 2016[562]	110	1916	0.06	[0.05; 0.07]
RuiFang 2018[566−1]	18	180	0.10	[0.06; 0.15]
RuiFang 2018[566−2]	18	183	0.10	[0.06; 0.15]
RuiFang 2018[566−3]	11	263	0.04	[0.02; 0.07]
RuiFang 2018[566−4]	5	382	0.01	[0.00; 0.03]
RuiFang 2018[566−5]	18	473	0.04	[0.02; 0.06]
RuiFang 2018[566−6]	16	670	0.02	[0.01; 0.04]
RuiFang 2018[566−7]	54	908	0.06	[0.04; 0.08]
RuiFang 2018[566−8]	8	1059	0.01	[0.00; 0.01]
RuiFang 2018[566−9]	78	1184	0.07	[0.05; 0.08]
RuiFang 2018[566−10]	10	2084	0.00	[0.00; 0.01]
RuiFang 2018[566−11]	15	2905	0.01	[0.00; 0.01]
Wu−Yang Shi 2020[577−1]	35	441	0.08	[0.06; 0.11]
ShiChao 2013[579]	462	2024	0.23	[0.21; 0.25]
ShuaiHuiQun 2012[589]	14	569	0.02	[0.01; 0.04]
SongCanLei 2020[590]	44	812	0.05	[0.04; 0.07]
SongCanLei 2012[591]	18	385	0.05	[0.03; 0.07]
SongCanLei 2013[592−2]	8	161	0.05	[0.02; 0.10]
SongCanLei 2017[593]	36	888	0.04	[0.03; 0.06]
SongCanLei 2013[594]	26	162	0.16	[0.11; 0.23]
SongCanLei 2013[595]	11	71	0.15	[0.08; 0.26]
SongJianQiang 2015[598]	259	10942	0.02	[0.02; 0.03]
SongYuFang 2019[600]	63	783	0.08	[0.06; 0.10]
SunJing 2015[611]	92	8929	0.01	[0.01; 0.01]
SunLiMei 2012[612]	108	5854	0.02	[0.02; 0.02]
SunLiYan 2020[613]	11	51	0.22	[0.11; 0.35]
SunMingHua 2017[614]	36	496	0.07	[0.05; 0.10]
SunQin 2019[616]	32	128	0.25	[0.18; 0.33]
SunYunLan 2018[624]	63	2212	0.03	[0.02; 0.04]
SunZhou 2016[625]	230	45075	0.01	[0.00; 0.01]

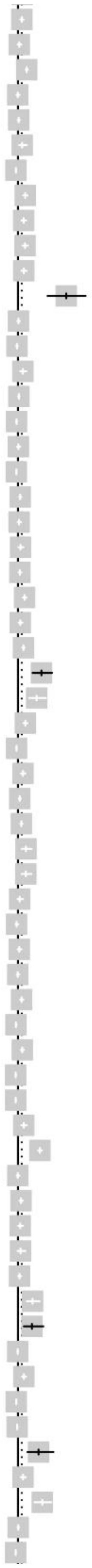

TanDongMei 2012[627]	87	2086	0.04	[0.03; 0.05]
TangYuHuan 2017[630]	44	2494	0.02	[0.01; 0.02]
TangGuoJie 2019[631]	84	2250	0.04	[0.03; 0.05]
TangXuLi 2013[633]	23	116	0.20	[0.13; 0.28]
TangYuXin 2014[634]	97	994	0.10	[0.08; 0.12]
TaoLiYan 2020[636]	47	1857	0.03	[0.02; 0.03]
WangJinSheng 2019[645]	56	1800	0.03	[0.02; 0.04]
WangJinSheng 2016[646]	99	11594	0.01	[0.01; 0.01]
WangDaHu 2020[652]	182	33479	0.01	[0.00; 0.01]
WangHu 2018[656]	72	28270	0.00	[0.00; 0.00]
WangHua 2014[657]	28	497	0.06	[0.04; 0.08]
WangJie 2015[659]	451	11467	0.04	[0.04; 0.04]
WangJun 2012[663]	17	400	0.04	[0.02; 0.07]
WangJun 2021[664]	20	4743	0.00	[0.00; 0.01]
WangKaiLiang 2019[665]	16	446	0.04	[0.02; 0.06]
WangMan 2017[668]	85	540	0.16	[0.13; 0.19]
WangMeiHuan 2020[669]	12	433	0.03	[0.01; 0.05]
WangMin 2018[670−1]	19	180	0.11	[0.06; 0.16]
WangMin 2018[670−2]	11	599	0.02	[0.01; 0.03]
WangMin 2018[670−3]	37	672	0.06	[0.04; 0.08]
WangMin 2018[670−4]	27	861	0.03	[0.02; 0.05]
WangMin 2018[670−5]	32	2843	0.01	[0.01; 0.02]
WangMin 2018[670−6]	47	2869	0.02	[0.01; 0.02]
WangShuangYing 2016[676]	16	119	0.13	[0.08; 0.21]
WangTieJun 2020[678]	15	345	0.04	[0.02; 0.07]
WangXiaoQin 2015[689]	11	142	0.08	[0.04; 0.13]
WeiGuiYing 2019[706]	29	257	0.11	[0.08; 0.16]
WenYa 2018[710]	16	668	0.02	[0.01; 0.04]
WenYingMing 2019[711]	15	35	0.43	[0.26; 0.61]
WuGuoFu 2018[717]	17	506	0.03	[0.02; 0.05]
WuHongXing 2016[718]	63	726	0.09	[0.07; 0.11]
WuJingWen 2020[719]	34	1153	0.03	[0.02; 0.04]
WuMingXiong 2018[723]	10	1292	0.01	[0.00; 0.01]
WuQinDi 2018[725]	24	1578	0.02	[0.01; 0.02]
WuShuiXin 2012[727]	25	1376	0.02	[0.01; 0.03]
WuWenQian 2018[729]	36	2399	0.02	[0.01; 0.02]
WuXiaYan 2015[730]	92	2046	0.04	[0.04; 0.05]
WuXiaoMin 2021[732]	159	1378	0.12	[0.10; 0.13]
WuYang 2018[733]	85	265	0.32	[0.26; 0.38]
WuYang 2018[734]	26	2179	0.01	[0.01; 0.02]
WuYang 2018[735]	91	516	0.18	[0.14; 0.21]
WuYiLing 2017[736]	31	1311	0.02	[0.02; 0.03]
WuZhaoChun 2015[737]	17	130	0.13	[0.08; 0.20]
WuZhenYu 2012[738]	19	57	0.33	[0.21; 0.47]
WuZhiSheng 2014[741]	176	14612	0.01	[0.01; 0.01]
XiaGuangHui 2019[742]	106	2300	0.05	[0.04; 0.06]
XiaYingPin 2018[743]	177	1777	0.10	[0.09; 0.11]
XiaZhongFa 2012[744]	209	1616	0.13	[0.11; 0.15]
XiaoSongJian 2017[745]	51	416	0.12	[0.09; 0.16]
XiaoDaYong 2018[746]	47	154	0.31	[0.23; 0.38]
XieCaiWen 2017[749]	12	58	0.21	[0.11; 0.33]
XieHuaPing 2010[752]	55	1638	0.03	[0.03; 0.04]
XieYuanQi 2016[756]	34	207	0.16	[0.12; 0.22]
XuJianRong 2018[767]	92	2046	0.04	[0.04; 0.05]
XuShiMin 2013[771]	37	116	0.32	[0.24; 0.41]
XuFeng 2012[774]	22	79	0.28	[0.18; 0.39]
XuHao 2016[775]	28	2385	0.01	[0.01; 0.02]
XuJinFeng 2016[778−1]	10	933	0.01	[0.01; 0.02]
XuJinFeng 2016[778−2]	16	2500	0.01	[0.00; 0.01]
XuanLingFeng 2014[784]	17	9523	0.00	[0.00; 0.00]
YanChaoYang 2017[792]	90	2168	0.04	[0.03; 0.05]
YangCaiBin 2018[796]	42	2159	0.02	[0.01; 0.03]

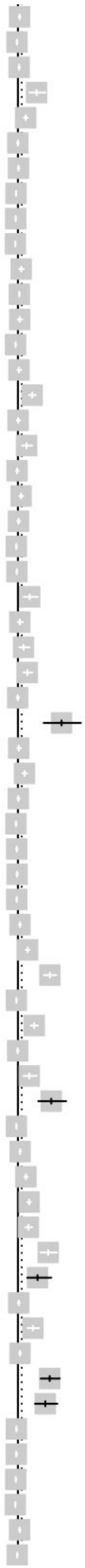

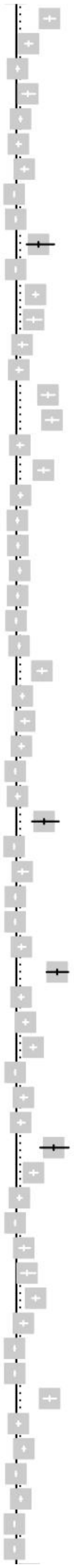

YangCheng 2017[797]	109	330	0.33	[0.28; 0.38]
YangGenMei 2015[798]	54	392	0.14	[0.11; 0.18]
YangJiXing 2019[801]	30	808	0.04	[0.03; 0.05]
YangJiChao 2016[802]	18	137	0.13	[0.08; 0.20]
YangJing 2018[804]	111	1718	0.06	[0.05; 0.08]
YangRongXing 2019[808]	15	334	0.04	[0.03; 0.07]
YangSenPing 2018[810]	30	305	0.10	[0.07; 0.14]
YangYiLong 2014[818]	121	18597	0.01	[0.01; 0.01]
YangYouQing 2016[819]	48	2363	0.02	[0.02; 0.03]
YangZhiWen 2013[820]	11	48	0.23	[0.12; 0.37]
YaoJianXiang 2016[822]	59	2992	0.02	[0.02; 0.03]
YaoJing 2017[824]	179	885	0.20	[0.18; 0.23]
Jin−Bo Ye 2018[830−1]	25	136	0.18	[0.12; 0.26]
Jin−Bo Ye 2018[830−2]	14	178	0.08	[0.04; 0.13]
Jin−Bo Ye 2018[830−3]	11	227	0.05	[0.02; 0.09]
Jin−Bo Ye 2018[830−4]	73	231	0.32	[0.26; 0.38]
Jin−Bo Ye 2018[830−5]	90	255	0.35	[0.29; 0.42]
Jin−Bo Ye 2018[830−6]	15	259	0.06	[0.03; 0.09]
Jin−Bo Ye 2018[830−7]	81	295	0.27	[0.22; 0.33]
Jin−Bo Ye 2018[830−8]	49	760	0.06	[0.05; 0.08]
Jin−Bo Ye 2018[830−9]	40	1125	0.04	[0.03; 0.05]
Jin−Bo Ye 2018[830−10]	55	1377	0.04	[0.03; 0.05]
Jin−Bo Ye 2018[830−11]	101	1784	0.06	[0.05; 0.07]
Jin−Bo Ye 2018[830−12]	77	2034	0.04	[0.03; 0.05]
Jin−Bo Ye 2018[830−13]	60	2572	0.02	[0.02; 0.03]
Jin−Bo Ye 2018[830−14]	132	2584	0.05	[0.04; 0.06]
YeShuJun 2019[831]	104	399	0.26	[0.22; 0.31]
YeXianMing 2017[832]	94	1156	0.08	[0.07; 0.10]
YeXiaoLing 2014[833]	27	264	0.10	[0.07; 0.15]
YinHongMei 2014[839]	37	511	0.07	[0.05; 0.10]
YingLiHong 2016[840]	48	3000	0.02	[0.01; 0.02]
YuZhuXian 2013[849]	13	357	0.04	[0.02; 0.06]
YuKuangMing 2015[851]	16	57	0.28	[0.17; 0.42]
YuanJun 2014[854]	652	115469	0.01	[0.01; 0.01]
YueYong 2017[859]	12	150	0.08	[0.04; 0.14]
ZhanYueWang 2019[860]	38	2311	0.02	[0.01; 0.02]
ZhangDongSheng 2013[864]	97	6614	0.01	[0.01; 0.02]
ZhangHengQiu 2014[869]	18	250	0.07	[0.04; 0.11]
ZhangHuiLing 2018[871]	40	100	0.40	[0.30; 0.50]
ZhangLi 2015[875−1]	33	474	0.07	[0.05; 0.10]
ZhangLi 2015[875−2]	72	656	0.11	[0.09; 0.14]
ZhangLing 2016[879]	132	735	0.18	[0.15; 0.21]
ZhangMeiMei 2013[880]	126	8154	0.02	[0.01; 0.02]
ZhangNing 2018[882]	31	340	0.09	[0.06; 0.13]
ZhangPan 2020[883]	16	244	0.07	[0.04; 0.10]
ZhangXiaoYi 2015[891]	21	57	0.37	[0.24; 0.51]
ZhangZhen 2015[903]	76	414	0.18	[0.15; 0.22]
ZhangZhengDong 2017[904]	25	466	0.05	[0.04; 0.08]
ZhangZhiZhong 2020[906]	263	17752	0.01	[0.01; 0.02]
ZhangGuangMing 2018[907]	14	149	0.09	[0.05; 0.15]
ZhangGuoBao 2020[908]	13	105	0.12	[0.07; 0.20]
ZhangQingHe 2020[909]	109	538	0.20	[0.17; 0.24]
ZhaoJun 2014[916]	26	287	0.09	[0.06; 0.13]
ZhaoMeiLing 2019[918−1]	8	842	0.01	[0.00; 0.02]
ZhaoMeiLing 2019[918−2]	39	3305	0.01	[0.01; 0.02]
ZhaoMengJiao 2018[919]	87	263	0.33	[0.27; 0.39]
ZhaoTengLong 2015[921]	25	564	0.04	[0.03; 0.06]
ZhaoXueCheng 2016[929]	149	1570	0.09	[0.08; 0.11]
ZhaoYiNan 2018[932]	67	3000	0.02	[0.02; 0.03]
ZhaoYuLi 2019[933]	119	1784	0.07	[0.06; 0.08]
HuangJiaCheng 2021[944]	48	5682	0.01	[0.01; 0.01]
ZhongJianMing 2014[946]	11	1208	0.01	[0.00; 0.02]

Study	Events	Total		Proportion	95% CI
ZhongWenLong 2012[948]	10	69		0.14	[0.07; 0.25]
ZhouAiHua 2017[950]	80	650		0.12	[0.10; 0.15]
ZhouJianHong 2013[954]	15	636		0.02	[0.01; 0.04]
ZhouLiHong 2016[956]	13	37		0.35	[0.20; 0.53]
ZhouXiaoHong 2015[958]	107	7113		0.02	[0.01; 0.02]
ZhouXiaoTao 2011[959]	96	1045		0.09	[0.08; 0.11]
ZhouXiaoHong 2010[960]	37	295		0.13	[0.09; 0.17]
ZhouXiaoHong 2017[961]	47	3464		0.01	[0.01; 0.02]
ZhouXiaoMin 2017[962]	47	3917		0.01	[0.01; 0.02]
ZhouYin 2016[964]	12	1061		0.01	[0.01; 0.02]
ZhouZhenQing 2016[966]	28	186		0.15	[0.10; 0.21]
ZhuChengMing 2017[967]	78	4454		0.02	[0.01; 0.02]
ZhuChunXiu 2019[968]	17	401		0.04	[0.02; 0.07]
ZhuWeiWei 2018[976]	91	9702		0.01	[0.01; 0.01]
ZongJun 2017[980]	45	78		0.58	[0.46; 0.69]
HuangKaiXiong 2021[989]	36	660		0.05	[0.04; 0.07]
LiXiaoJing 2021[992]	7	251		0.03	[0.01; 0.06]
ShenJingQiang 2020[993]	15	37		0.41	[0.25; 0.58]
Cui C 2017[994]	34	400		0.08	[0.06; 0.12]
Guo Z 2014[1001]	87	7141		0.01	[0.01; 0.02]
Huang J 2013[1006−3]	6	33		0.18	[0.07; 0.35]
Huang J 2013[1006−4]	6	38		0.16	[0.06; 0.31]
Huang J 2013[1006−5]	14	103		0.14	[0.08; 0.22]
Huang Z 2020[1008]	68	786		0.09	[0.07; 0.11]
Li J 2018[1015]	19	1327		0.01	[0.01; 0.02]
Y Li 2021[1018]	143	346		0.41	[0.36; 0.47]
Y Li 2013[1019]	396	14387		0.03	[0.02; 0.03]
Liu Y 2015[1025]	99	2049		0.05	[0.04; 0.06]
Lu Y 2020[1029]	157	785		0.20	[0.17; 0.23]
Luo LF 2015[1030]	32	45		0.71	[0.56; 0.84]
Qi L 2018[1034]	101	555		0.18	[0.15; 0.22]
WuLin 2021[1035]	17	194		0.09	[0.05; 0.14]
Ruan F 2013[1039]	63	22200		0.00	[0.00; 0.00]
Shang X 2017[1041]	924	23068		0.04	[0.04; 0.04]
Shi C 2016[1046]	57	1966		0.03	[0.02; 0.04]
Tsang TK 2018[1050]	159	643		0.25	[0.21; 0.28]
Wang X 2016[1055]	51	377		0.14	[0.10; 0.17]
Xu H 2013[1059−1]	207	1630		0.13	[0.11; 0.14]
Xu H 2013[1059−2]	65	1912		0.03	[0.03; 0.04]
Xue C 2014[1060]	278	1995		0.14	[0.12; 0.16]
Yang Z 2011[1068]	427	71534		0.01	[0.01; 0.01]
Zhang TL 2017[1075]	20	327		0.06	[0.04; 0.09]
Zhang XF 2020[1076]	27	450		0.06	[0.04; 0.09]
Zhang XF 2015[1077]	276	2442		0.11	[0.10; 0.13]
Zhou X 2019[1080]	75	1612		0.05	[0.04; 0.06]
Zhou X 2012[1081]	108	5769		0.02	[0.02; 0.02]
LiuQin 2011[1088]	257	2497		0.10	[0.09; 0.12]
XuHaoBin 2015[1091]	39	496		0.08	[0.06; 0.11]
LiYing 2018[1097]	53	1288		0.04	[0.03; 0.05]
XiangLunHui 2016[1101]	51	533		0.10	[0.07; 0.12]
ZouYong 2011[1103]	39	2672		0.01	[0.01; 0.02]
**Common effect model**		**1312440**		**0.02**	**[0.02; 0.02]**
**Random effects model**				**0.05**	**[0.04; 0.05]**
Heterogeneity: $I^2 = 99\%$, $\tau^2 = 1.6181$, $p = 0$					
**Common effect model**		**1700883**		**0.03**	**[0.03; 0.03]**
**Random effects model**				**0.06**	**[0.06; 0.07]**

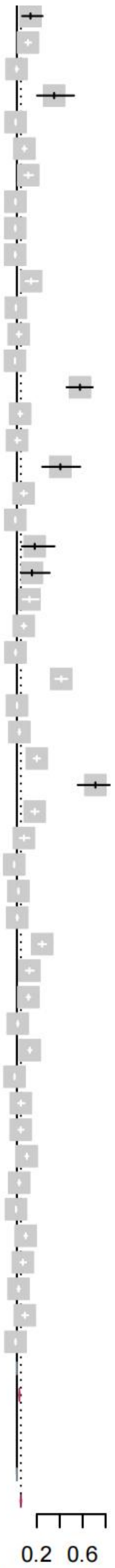

Heterogeneity: $I^2 = 99\%$, $\tau^2 = 1.8337$, $p = 0$

Test for subgroup differences (common effect): $\chi^2_1 = 814.29$, df = 1 ($p < 0.01$)

Test for subgroup differences (random effects): $\chi^2_1 = 54.23$, df = 1 ($p < 0.01$)

(B)

(b1)

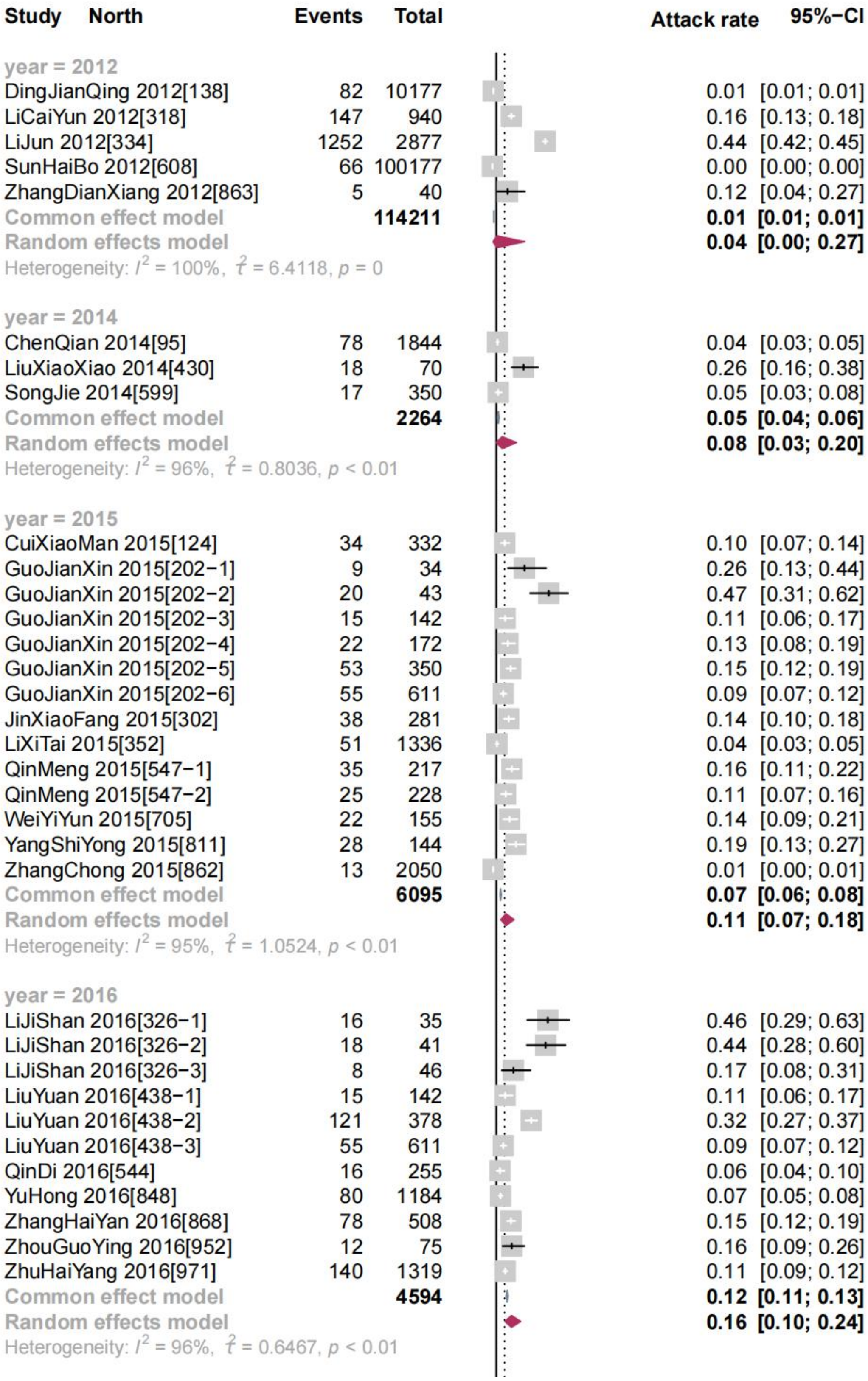
Study North Events Total Attack rate 95%−CI
year = 2012
DingJianQing 2012[138] 82 10177 0.01 [0.01; 0.01]
LiCaiYun 2012[318] 147 940 0.16 [0.13; 0.18]
LiJun 2012[334] 1252 2877 0.44 [0.42; 0.45]
SunHaiBo 2012[608] 66 100177 0.00 [0.00; 0.00]
ZhangDianXiang 2012[863] 5 40 0.12 [0.04; 0.27]
Common effect model 114211 0.01 [0.01; 0.01]
Random effects model 0.04 [0.00; 0.27]
Heterogeneity: I^2 = 100%, τ^2 = 6.4118, p = 0
year = 2014
ChenQian 2014[95] 78 1844 0.04 [0.03; 0.05]
LiuXiaoXiao 2014[430] 18 70 0.26 [0.16; 0.38]
SongJie 2014[599] 17 350 0.05 [0.03; 0.08]
Common effect model 2264 0.05 [0.04; 0.06]
Random effects model 0.08 [0.03; 0.20]
Heterogeneity: I^2 = 96%, τ^2 = 0.8036, p < 0.01
year = 2015
CuiXiaoMan 2015[124] 34 332 0.10 [0.07; 0.14]
GuoJianXin 2015[202−1] 9 34 0.26 [0.13; 0.44]
GuoJianXin 2015[202−2] 20 43 0.47 [0.31; 0.62]
GuoJianXin 2015[202−3] 15 142 0.11 [0.06; 0.17]
GuoJianXin 2015[202−4] 22 172 0.13 [0.08; 0.19]
GuoJianXin 2015[202−5] 53 350 0.15 [0.12; 0.19]
GuoJianXin 2015[202−6] 55 611 0.09 [0.07; 0.12]
JinXiaoFang 2015[302] 38 281 0.14 [0.10; 0.18]
LiXiTai 2015[352] 51 1336 0.04 [0.03; 0.05]
QinMeng 2015[547−1] 35 217 0.16 [0.11; 0.22]
QinMeng 2015[547−2] 25 228 0.11 [0.07; 0.16]
WeiYiYun 2015[705] 22 155 0.14 [0.09; 0.21]
YangShiYong 2015[811] 28 144 0.19 [0.13; 0.27]
ZhangChong 2015[862] 13 2050 0.01 [0.00; 0.01]
Common effect model 6095 0.07 [0.06; 0.08]
Random effects model 0.11 [0.07; 0.18]
Heterogeneity: I^2 = 95%, τ^2 = 1.0524, p < 0.01
year = 2016
LiJiShan 2016[326−1] 16 35 0.46 [0.29; 0.63]
LiJiShan 2016[326−2] 18 41 0.44 [0.28; 0.60]
LiJiShan 2016[326−3] 8 46 0.17 [0.08; 0.31]
LiuYuan 2016[438−1] 15 142 0.11 [0.06; 0.17]
LiuYuan 2016[438−2] 121 378 0.32 [0.27; 0.37]
LiuYuan 2016[438−3] 55 611 0.09 [0.07; 0.12]
QinDi 2016[544] 16 255 0.06 [0.04; 0.10]
YuHong 2016[848] 80 1184 0.07 [0.05; 0.08]
ZhangHaiYan 2016[868] 78 508 0.15 [0.12; 0.19]
ZhouGuoYing 2016[952] 12 75 0.16 [0.09; 0.26]
ZhuHaiYang 2016[971] 140 1319 0.11 [0.09; 0.12]
Common effect model 4594 0.12 [0.11; 0.13]
Random effects model 0.16 [0.10; 0.24]
Heterogeneity: I^2 = 96%, τ^2 = 0.6467, p < 0.01

**year = 2017**			
Cai W 2017[24−4]	12	32	0.38 [0.21; 0.56]
Cai W 2017[24−5]	10	32	0.31 [0.16; 0.50]
Cai W 2017[24−6]	24	36	0.67 [0.49; 0.81]
Cai W 2017[24−7]	8	39	0.21 [0.09; 0.36]
Cai W 2017[24−8]	13	40	0.32 [0.19; 0.49]
Cai W 2017[24−9]	12	43	0.28 [0.15; 0.44]
Cai W 2017[24−10]	17	49	0.35 [0.22; 0.50]
Cai W 2017[24−11]	10	67	0.15 [0.07; 0.26]
Cai W 2017[24−12]	16	78	0.21 [0.12; 0.31]
CuiLiangLiang 2017[123−1]	5	90	0.06 [0.02; 0.12]
CuiLiangLiang 2017[123−2]	14	4600	0.00 [0.00; 0.01]
CuiLiangLiang 2017[123−3]	55	10000	0.01 [0.00; 0.01]
CuiLiangLiang 2017[123−4]	1434	11000	0.13 [0.12; 0.14]
CuiLiangLiang 2017[123−5]	385	15000	0.03 [0.02; 0.03]
CuiLiangLiang 2017[123−6]	235	20000	0.01 [0.01; 0.01]
GaoZhiYong 2017[187−1]	12	32	0.38 [0.21; 0.56]
GaoZhiYong 2017[187−2]	10	46	0.22 [0.11; 0.36]
GaoZhiYong 2017[187−3]	15	57	0.26 [0.16; 0.40]
GaoZhiYong 2017[187−4]	16	63	0.25 [0.15; 0.38]
GaoZhiYong 2017[187−5]	7	78	0.09 [0.04; 0.18]
GaoZhiYong 2017[187−6]	25	953	0.03 [0.02; 0.04]
GaoZhiYong 2017[187−7]	21	980	0.02 [0.01; 0.03]
GaoZhiYong 2017[187−8]	22	1047	0.02 [0.01; 0.03]
HeXuXin 2017[222]	156	466	0.33 [0.29; 0.38]
HuGuangYi 2017[232]	20	210	0.10 [0.06; 0.14]
LiYan 2017[361]	93	2355	0.04 [0.03; 0.05]
LiuBaiWei 2017[395]	24	471	0.05 [0.03; 0.07]
LiuBaiWei 2017[396−1]	43	222	0.19 [0.14; 0.25]
LiuBaiWei 2017[396−2]	172	3546	0.05 [0.04; 0.06]
LiuBaiWei 2017[396−3]	58	4500	0.01 [0.01; 0.02]
LiuXiuMei 2017[433]	65	1134	0.06 [0.04; 0.07]
SongHuiRong 2017[597]	34	568	0.06 [0.04; 0.08]
TianJing 2017[641]	39	392	0.10 [0.07; 0.13]
WangBing 2017[649]	58	20000	0.00 [0.00; 0.00]
WangXiaoDong 2017[688]	69	2708	0.03 [0.02; 0.03]
XingYan 2017[759]	119	3500	0.03 [0.03; 0.04]
YanGeBin 2017[786−1]	14	35	0.40 [0.24; 0.58]
YanGeBin 2017[786−2]	25	62	0.40 [0.28; 0.54]
ZhangShuang 2017[888]	28	45	0.62 [0.47; 0.76]
ZhangYan 2017[894−1]	8	37	0.22 [0.10; 0.38]
ZhangYan 2017[894−2]	12	2857	0.00 [0.00; 0.01]
Huang XY 2017[1007]	753	22861	0.03 [0.03; 0.04]
**Common effect model**		**130331**	**0.03 [0.03; 0.03]**
**Random effects model**			**0.09 [0.05; 0.14]**
Heterogeneity: $I^2 = 99\%$, $\tau^2 = 2.7274$, $p = 0$			
**year = 2018**			
BaiYun 2018[8]	61	648	0.09 [0.07; 0.12]
Cai W 2018[23−2]	38	117	0.32 [0.24; 0.42]
Cai W 2018[23−3]	217	2504	0.09 [0.08; 0.10]
CaiXiuZhi 2018[27−1]	34	150	0.23 [0.16; 0.30]
CaiXiuZhi 2018[27−2]	34	400	0.08 [0.06; 0.12]
CaiXiuZhi 2018[27−3]	40	1045	0.04 [0.03; 0.05]
CaiXiuZhi 2018[27−4]	24	1279	0.02 [0.01; 0.03]
CuiXiaoMan 2018[125]	17	829	0.02 [0.01; 0.03]
FangYunXia 2018[157]	27	357	0.08 [0.05; 0.11]
GaoZhiPeng 2018[186]	130	397	0.33 [0.28; 0.38]
HuaWeiYu 2018[241]	63	1951	0.03 [0.02; 0.04]

HuaWeiYu 2018[242−1]	15	156	0.10 [0.05; 0.15]
HuaWeiYu 2018[242−2]	17	267	0.06 [0.04; 0.10]
HuaWeiYu 2018[242−3]	9	290	0.03 [0.01; 0.06]
HuaWeiYu 2018[242−4]	14	320	0.04 [0.02; 0.07]
HuaWeiYu 2018[242−5]	13	325	0.04 [0.02; 0.07]
HuaWeiYu 2018[242−6]	5	833	0.01 [0.00; 0.01]
HuaWeiYu 2018[242−7]	13	1806	0.01 [0.00; 0.01]
LiJiShan 2018[324−2]	7	40	0.17 [0.07; 0.33]
LiJiShan 2018[324−3]	7	78	0.09 [0.04; 0.18]
LiShiE 2018[347−1]	6	36	0.17 [0.06; 0.33]
LiShiE 2018[347−2]	15	38	0.39 [0.24; 0.57]
LiShiE 2018[347−3]	16	48	0.33 [0.20; 0.48]
LiShiE 2018[347−4]	22	66	0.33 [0.22; 0.46]
LiShiE 2018[347−5]	37	89	0.42 [0.31; 0.53]
LiShiE 2018[347−6]	14	94	0.15 [0.08; 0.24]
LiShiE 2018[347−7]	24	126	0.19 [0.13; 0.27]
LiShiE 2018[347−8]	34	173	0.20 [0.14; 0.26]
LiShiE 2018[347−9]	10	221	0.05 [0.02; 0.08]
LiShiE 2018[348]	92	1419	0.06 [0.05; 0.08]
LiShiE 2018[349]	77	8153	0.01 [0.01; 0.01]
LuXiuZhi 2018[463]	47	3600	0.01 [0.01; 0.02]
QiYing 2018[535−1]	173	1621	0.11 [0.09; 0.12]
QiYing 2018[535−2]	71	1621	0.04 [0.03; 0.05]
RenQiZhi 2018[557−1]	6	100	0.06 [0.02; 0.13]
RenQiZhi 2018[557−2]	12	150	0.08 [0.04; 0.14]
RenQiZhi 2018[557−3]	8	151	0.05 [0.02; 0.10]
RenQiZhi 2018[557−4]	15	200	0.07 [0.04; 0.12]
RenQiZhi 2018[557−5]	5	2000	0.00 [0.00; 0.01]
RenQiZhi 2018[557−6]	13	2500	0.01 [0.00; 0.01]
RenQiZhi 2018[557−7]	15	10000	0.00 [0.00; 0.00]
SunWenLong 2018[618]	8	142	0.06 [0.02; 0.11]
XieBin 2018[757]	95	5000	0.02 [0.02; 0.02]
XuYan 2018[772]	68	969	0.07 [0.05; 0.09]
XuJun 2018[779]	75	2679	0.03 [0.02; 0.03]
YangAiQing 2018[795]	40	355	0.11 [0.08; 0.15]
YangTongTong 2018[813]	36	450	0.08 [0.06; 0.11]
YuXiaoYun 2018[845]	78	1007	0.08 [0.06; 0.10]
ZhangAiHua 2018[861]	19	214	0.09 [0.05; 0.14]
Guo XH 2018[1000]	61	1606	0.04 [0.03; 0.05]
Zhang L 2018[1071]	631	15118	0.04 [0.04; 0.05]
**Common effect model**		**73738**	**0.04 [0.03; 0.04]**
**Random effects model**			**0.06 [0.04; 0.08]**
Heterogeneity: $I^2 = 98\%$, $\tau^2 = 1.7640$, $p = 0$			
**year = 2019**			
GaoRiHong 2019[182]	30	72	0.42 [0.30; 0.54]
GuoLi 2019[205]	75	456	0.16 [0.13; 0.20]
GuoLi 2019[206−15]	9	31	0.29 [0.14; 0.48]
GuoLi 2019[206−16]	8	31	0.26 [0.12; 0.45]
GuoLi 2019[206−17]	10	31	0.32 [0.17; 0.51]
GuoLi 2019[206−18]	14	32	0.44 [0.26; 0.62]
GuoLi 2019[206−19]	9	33	0.27 [0.13; 0.46]
GuoLi 2019[206−20]	18	33	0.55 [0.36; 0.72]
GuoLi 2019[206−21]	8	34	0.24 [0.11; 0.41]
GuoLi 2019[206−22]	10	34	0.29 [0.15; 0.47]
GuoLi 2019[206−23]	14	34	0.41 [0.25; 0.59]
GuoLi 2019[206−24]	18	35	0.51 [0.34; 0.69]

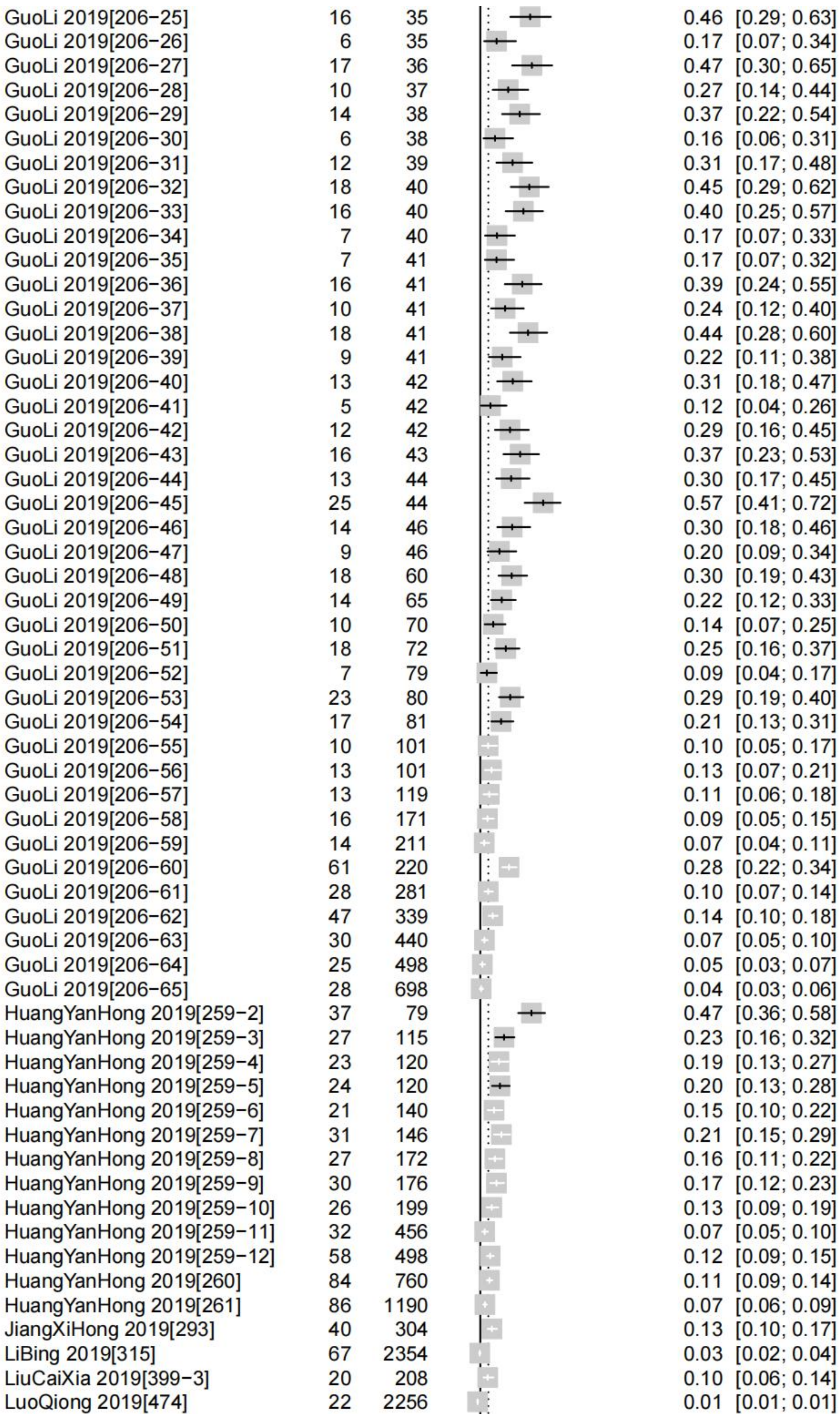
GuoLi 2019[206−25] 16 35 0.46 [0.29; 0.63]
GuoLi 2019[206−26] 6 35 0.17 [0.07; 0.34]
GuoLi 2019[206−27] 17 36 0.47 [0.30; 0.65]
GuoLi 2019[206−28] 10 37 0.27 [0.14; 0.44]
GuoLi 2019[206−29] 14 38 0.37 [0.22; 0.54]
GuoLi 2019[206−30] 6 38 0.16 [0.06; 0.31]
GuoLi 2019[206−31] 12 39 0.31 [0.17; 0.48]
GuoLi 2019[206−32] 18 40 0.45 [0.29; 0.62]
GuoLi 2019[206−33] 16 40 0.40 [0.25; 0.57]
GuoLi 2019[206−34] 7 40 0.17 [0.07; 0.33]
GuoLi 2019[206−35] 7 41 0.17 [0.07; 0.32]
GuoLi 2019[206−36] 16 41 0.39 [0.24; 0.55]
GuoLi 2019[206−37] 10 41 0.24 [0.12; 0.40]
GuoLi 2019[206−38] 18 41 0.44 [0.28; 0.60]
GuoLi 2019[206−39] 9 41 0.22 [0.11; 0.38]
GuoLi 2019[206−40] 13 42 0.31 [0.18; 0.47]
GuoLi 2019[206−41] 5 42 0.12 [0.04; 0.26]
GuoLi 2019[206−42] 12 42 0.29 [0.16; 0.45]
GuoLi 2019[206−43] 16 43 0.37 [0.23; 0.53]
GuoLi 2019[206−44] 13 44 0.30 [0.17; 0.45]
GuoLi 2019[206−45] 25 44 0.57 [0.41; 0.72]
GuoLi 2019[206−46] 14 46 0.30 [0.18; 0.46]
GuoLi 2019[206−47] 9 46 0.20 [0.09; 0.34]
GuoLi 2019[206−48] 18 60 0.30 [0.19; 0.43]
GuoLi 2019[206−49] 14 65 0.22 [0.12; 0.33]
GuoLi 2019[206−50] 10 70 0.14 [0.07; 0.25]
GuoLi 2019[206−51] 18 72 0.25 [0.16; 0.37]
GuoLi 2019[206−52] 7 79 0.09 [0.04; 0.17]
GuoLi 2019[206−53] 23 80 0.29 [0.19; 0.40]
GuoLi 2019[206−54] 17 81 0.21 [0.13; 0.31]
GuoLi 2019[206−55] 10 101 0.10 [0.05; 0.17]
GuoLi 2019[206−56] 13 101 0.13 [0.07; 0.21]
GuoLi 2019[206−57] 13 119 0.11 [0.06; 0.18]
GuoLi 2019[206−58] 16 171 0.09 [0.05; 0.15]
GuoLi 2019[206−59] 14 211 0.07 [0.04; 0.11]
GuoLi 2019[206−60] 61 220 0.28 [0.22; 0.34]
GuoLi 2019[206−61] 28 281 0.10 [0.07; 0.14]
GuoLi 2019[206−62] 47 339 0.14 [0.10; 0.18]
GuoLi 2019[206−63] 30 440 0.07 [0.05; 0.10]
GuoLi 2019[206−64] 25 498 0.05 [0.03; 0.07]
GuoLi 2019[206−65] 28 698 0.04 [0.03; 0.06]
HuangYanHong 2019[259−2] 37 79 0.47 [0.36; 0.58]
HuangYanHong 2019[259−3] 27 115 0.23 [0.16; 0.32]
HuangYanHong 2019[259−4] 23 120 0.19 [0.13; 0.27]
HuangYanHong 2019[259−5] 24 120 0.20 [0.13; 0.28]
HuangYanHong 2019[259−6] 21 140 0.15 [0.10; 0.22]
HuangYanHong 2019[259−7] 31 146 0.21 [0.15; 0.29]
HuangYanHong 2019[259−8] 27 172 0.16 [0.11; 0.22]
HuangYanHong 2019[259−9] 30 176 0.17 [0.12; 0.23]
HuangYanHong 2019[259−10] 26 199 0.13 [0.09; 0.19]
HuangYanHong 2019[259−11] 32 456 0.07 [0.05; 0.10]
HuangYanHong 2019[259−12] 58 498 0.12 [0.09; 0.15]
HuangYanHong 2019[260] 84 760 0.11 [0.09; 0.14]
HuangYanHong 2019[261] 86 1190 0.07 [0.06; 0.09]
JiangXiHong 2019[293] 40 304 0.13 [0.10; 0.17]
LiBing 2019[315] 67 2354 0.03 [0.02; 0.04]
LiuCaiXia 2019[399−3] 20 208 0.10 [0.06; 0.14]
LuoQiong 2019[474] 22 2256 0.01 [0.01; 0.01]

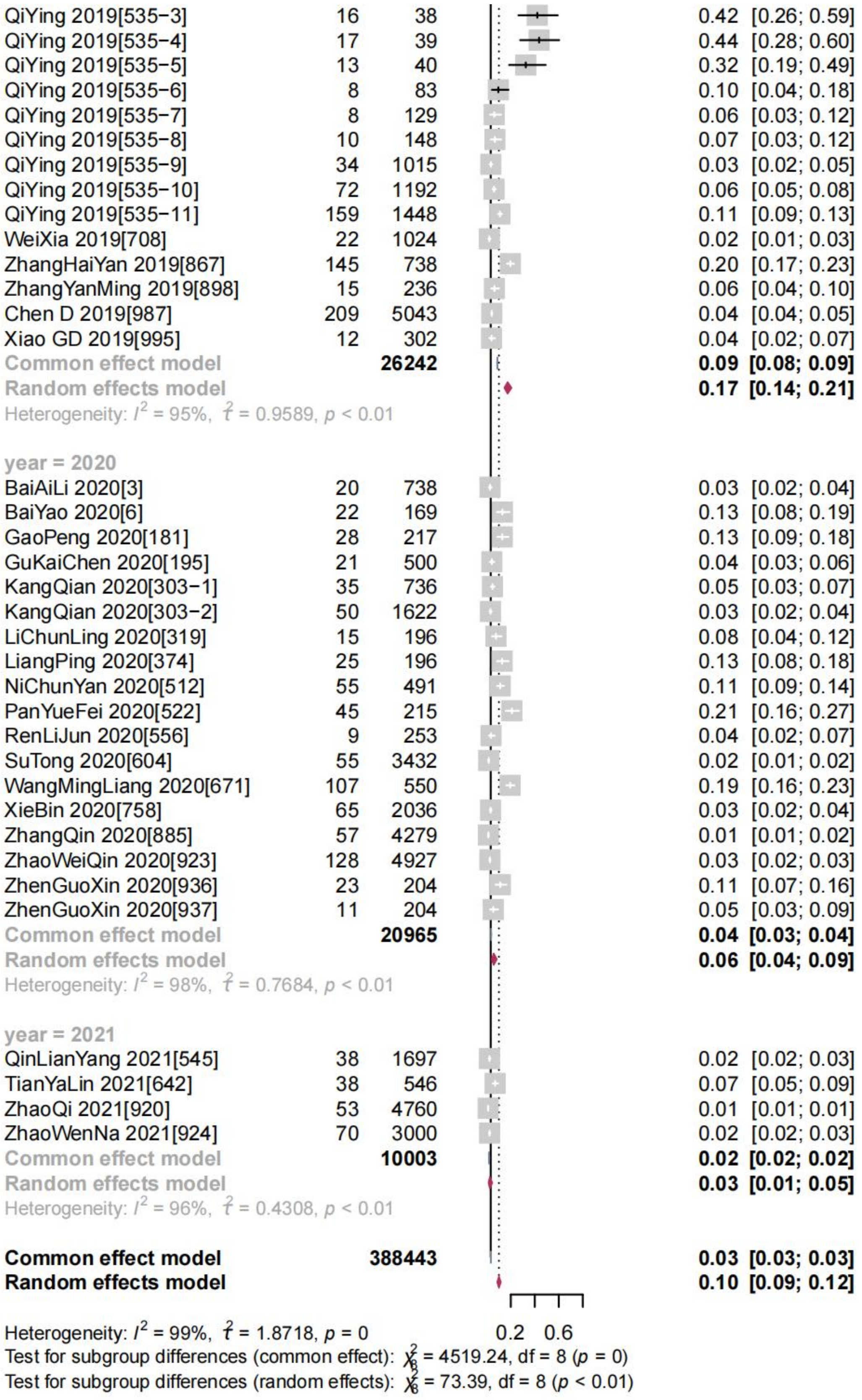
QiYing 2019[535−3] 16 38 0.42 [0.26; 0.59]
QiYing 2019[535−4] 17 39 0.44 [0.28; 0.60]
QiYing 2019[535−5] 13 40 0.32 [0.19; 0.49]
QiYing 2019[535−6] 8 83 0.10 [0.04; 0.18]
QiYing 2019[535−7] 8 129 0.06 [0.03; 0.12]
QiYing 2019[535−8] 10 148 0.07 [0.03; 0.12]
QiYing 2019[535−9] 34 1015 0.03 [0.02; 0.05]
QiYing 2019[535−10] 72 1192 0.06 [0.05; 0.08]
QiYing 2019[535−11] 159 1448 0.11 [0.09; 0.13]
WeiXia 2019[708] 22 1024 0.02 [0.01; 0.03]
ZhangHaiYan 2019[867] 145 738 0.20 [0.17; 0.23]
ZhangYanMing 2019[898] 15 236 0.06 [0.04; 0.10]
Chen D 2019[987] 209 5043 0.04 [0.04; 0.05]
Xiao GD 2019[995] 12 302 0.04 [0.02; 0.07]
Common effect model 26242 0.09 [0.08; 0.09]
Random effects model 0.17 [0.14; 0.21]
Heterogeneity: I^2 = 95%, τ^2 = 0.9589, $p < 0.01$
year = 2020
BaiAiLi 2020[3] 20 738 0.03 [0.02; 0.04]
BaiYao 2020[6] 22 169 0.13 [0.08; 0.19]
GaoPeng 2020[181] 28 217 0.13 [0.09; 0.18]
GuKaiChen 2020[195] 21 500 0.04 [0.03; 0.06]
KangQian 2020[303−1] 35 736 0.05 [0.03; 0.07]
KangQian 2020[303−2] 50 1622 0.03 [0.02; 0.04]
LiChunLing 2020[319] 15 196 0.08 [0.04; 0.12]
LiangPing 2020[374] 25 196 0.13 [0.08; 0.18]
NiChunYan 2020[512] 55 491 0.11 [0.09; 0.14]
PanYueFei 2020[522] 45 215 0.21 [0.16; 0.27]
RenLiJun 2020[556] 9 253 0.04 [0.02; 0.07]
SuTong 2020[604] 55 3432 0.02 [0.01; 0.02]
WangMingLiang 2020[671] 107 550 0.19 [0.16; 0.23]
XieBin 2020[758] 65 2036 0.03 [0.02; 0.04]
ZhangQin 2020[885] 57 4279 0.01 [0.01; 0.02]
ZhaoWeiQin 2020[923] 128 4927 0.03 [0.02; 0.03]
ZhenGuoXin 2020[936] 23 204 0.11 [0.07; 0.16]
ZhenGuoXin 2020[937] 11 204 0.05 [0.03; 0.09]
Common effect model 20965 0.04 [0.03; 0.04]
Random effects model 0.06 [0.04; 0.09]
Heterogeneity: I^2 = 98%, τ^2 = 0.7684, $p < 0.01$
year = 2021
QinLianYang 2021[545] 38 1697 0.02 [0.02; 0.03]
TianYaLin 2021[642] 38 546 0.07 [0.05; 0.09]
ZhaoQi 2021[920] 53 4760 0.01 [0.01; 0.01]
ZhaoWenNa 2021[924] 70 3000 0.02 [0.02; 0.03]
Common effect model 10003 0.02 [0.02; 0.02]
Random effects model 0.03 [0.01; 0.05]
Heterogeneity: I^2 = 96%, τ^2 = 0.4308, $p < 0.01$
Common effect model 388443 0.03 [0.03; 0.03]
Random effects model 0.10 [0.09; 0.12]
0.2 0.6
Heterogeneity: I^2 = 99%, τ^2 = 1.8718, $p = 0$
Test for subgroup differences (common effect): χ^2_8 = 4519.24, df = 8 ($p = 0$)
Test for subgroup differences (random effects): χ^2_8 = 73.39, df = 8 ($p < 0.01$)

(b2)

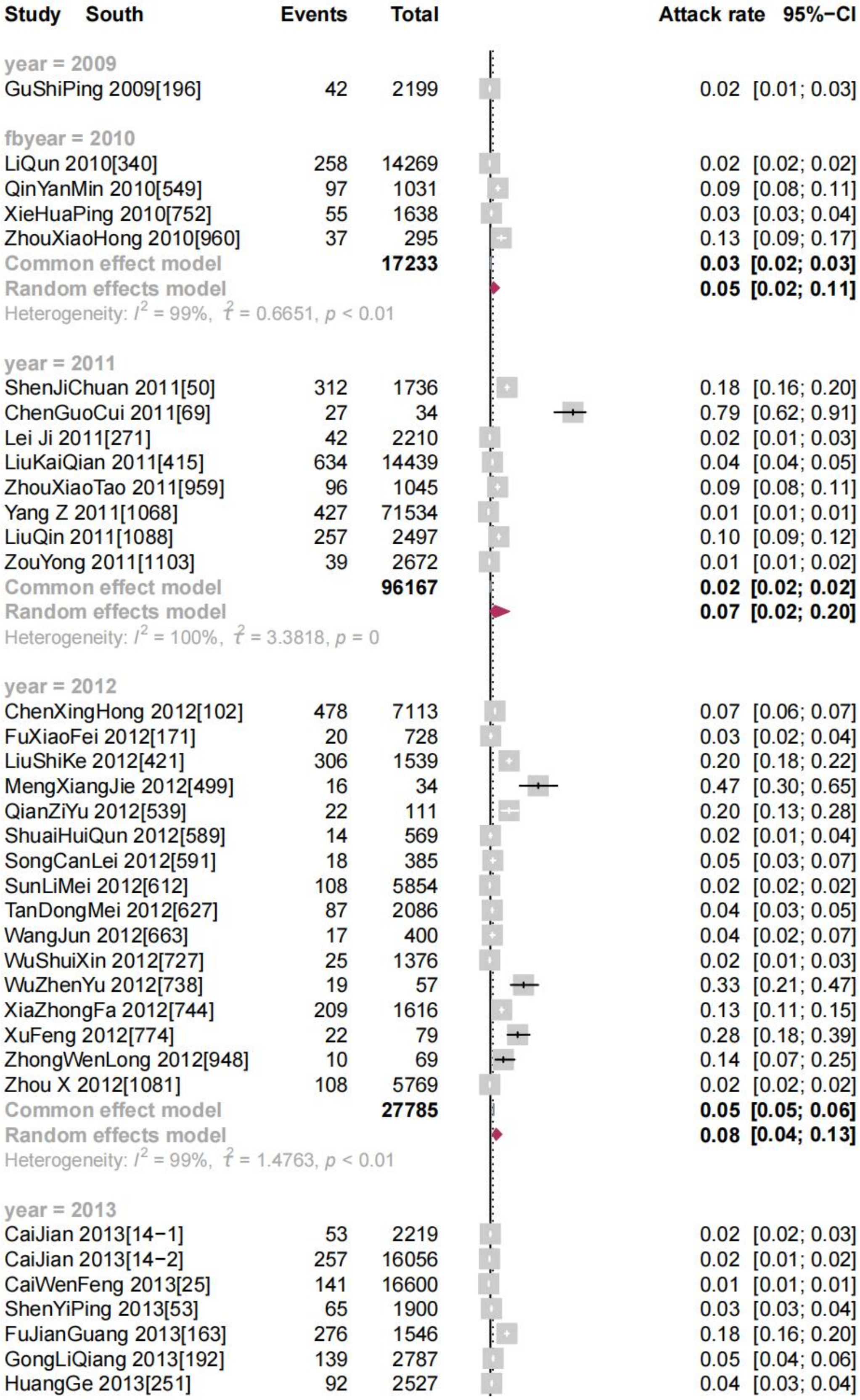
Study South Events Total Attack rate 95%−CI
year = 2009
GuShiPing 2009[196] 42 2199 0.02 [0.01; 0.03]
fbyear = 2010
LiQun 2010[340] 258 14269 0.02 [0.02; 0.02]
QinYanMin 2010[549] 97 1031 0.09 [0.08; 0.11]
XieHuaPing 2010[752] 55 1638 0.03 [0.03; 0.04]
ZhouXiaoHong 2010[960] 37 295 0.13 [0.09; 0.17]
Common effect model 17233 0.03 [0.02; 0.03]
Random effects model 0.05 [0.02; 0.11]
Heterogeneity: I^2 = 99%, τ^2 = 0.6651, p < 0.01
year = 2011
ShenJiChuan 2011[50] 312 1736 0.18 [0.16; 0.20]
ChenGuoCui 2011[69] 27 34 0.79 [0.62; 0.91]
Lei Ji 2011[271] 42 2210 0.02 [0.01; 0.03]
LiuKaiQian 2011[415] 634 14439 0.04 [0.04; 0.05]
ZhouXiaoTao 2011[959] 96 1045 0.09 [0.08; 0.11]
Yang Z 2011[1068] 427 71534 0.01 [0.01; 0.01]
LiuQin 2011[1088] 257 2497 0.10 [0.09; 0.12]
ZouYong 2011[1103] 39 2672 0.01 [0.01; 0.02]
Common effect model 96167 0.02 [0.02; 0.02]
Random effects model 0.07 [0.02; 0.20]
Heterogeneity: I^2 = 100%, τ^2 = 3.3818, p = 0
year = 2012
ChenXingHong 2012[102] 478 7113 0.07 [0.06; 0.07]
FuXiaoFei 2012[171] 20 728 0.03 [0.02; 0.04]
LiuShiKe 2012[421] 306 1539 0.20 [0.18; 0.22]
MengXiangJie 2012[499] 16 34 0.47 [0.30; 0.65]
QianZiYu 2012[539] 22 111 0.20 [0.13; 0.28]
ShuaiHuiQun 2012[589] 14 569 0.02 [0.01; 0.04]
SongCanLei 2012[591] 18 385 0.05 [0.03; 0.07]
SunLiMei 2012[612] 108 5854 0.02 [0.02; 0.02]
TanDongMei 2012[627] 87 2086 0.04 [0.03; 0.05]
WangJun 2012[663] 17 400 0.04 [0.02; 0.07]
WuShuiXin 2012[727] 25 1376 0.02 [0.01; 0.03]
WuZhenYu 2012[738] 19 57 0.33 [0.21; 0.47]
XiaZhongFa 2012[744] 209 1616 0.13 [0.11; 0.15]
XuFeng 2012[774] 22 79 0.28 [0.18; 0.39]
ZhongWenLong 2012[948] 10 69 0.14 [0.07; 0.25]
Zhou X 2012[1081] 108 5769 0.02 [0.02; 0.02]
Common effect model 27785 0.05 [0.05; 0.06]
Random effects model 0.08 [0.04; 0.13]
Heterogeneity: I^2 = 99%, τ^2 = 1.4763, p < 0.01
year = 2013
CaiJian 2013[14−1] 53 2219 0.02 [0.02; 0.03]
CaiJian 2013[14−2] 257 16056 0.02 [0.01; 0.02]
CaiWenFeng 2013[25] 141 16600 0.01 [0.01; 0.01]
ShenYiPing 2013[53] 65 1900 0.03 [0.03; 0.04]
FuJianGuang 2013[163] 276 1546 0.18 [0.16; 0.20]
GongLiQiang 2013[192] 139 2787 0.05 [0.04; 0.06]
HuangGe 2013[251] 92 2527 0.04 [0.03; 0.04]

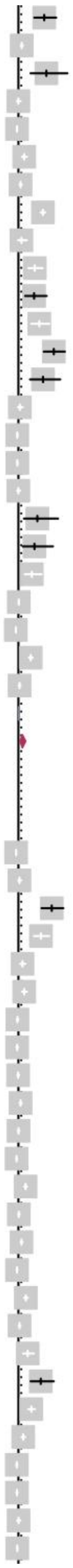

HuangZhongXue 2013[263]	22	92	0.24 [0.16; 0.34]
LiBo 2013[316]	120	2303	0.05 [0.04; 0.06]
LiuYi 2013[436]	9	35	0.26 [0.12; 0.43]
LuoGuiHe 2013[467]	10	422	0.02 [0.01; 0.04]
QiuHaiYan 2013[551]	76	5965	0.01 [0.01; 0.02]
RenFuLin 2013[554]	43	594	0.07 [0.05; 0.10]
RenFuLin 2013[555]	74	1751	0.04 [0.03; 0.05]
ShiChao 2013[579]	462	2024	0.23 [0.21; 0.25]
SongCanLei 2013[592−2]	8	161	0.05 [0.02; 0.10]
SongCanLei 2013[594]	26	162	0.16 [0.11; 0.23]
SongCanLei 2013[595]	11	71	0.15 [0.08; 0.26]
TangXuLi 2013[633]	23	116	0.20 [0.13; 0.28]
XuShiMin 2013[771]	37	116	0.32 [0.24; 0.41]
YangZhiWen 2013[820]	11	48	0.23 [0.12; 0.37]
YuZhuXian 2013[849]	13	357	0.04 [0.02; 0.06]
ZhangDongSheng 2013[864]	97	6614	0.01 [0.01; 0.02]
ZhangMeiMei 2013[880]	126	8154	0.02 [0.01; 0.02]
ZhouJianHong 2013[954]	15	636	0.02 [0.01; 0.04]
Huang J 2013[1006−3]	6	33	0.18 [0.07; 0.35]
Huang J 2013[1006−4]	6	38	0.16 [0.06; 0.31]
Huang J 2013[1006−5]	14	103	0.14 [0.08; 0.22]
Y Li 2013[1019]	396	14387	0.03 [0.02; 0.03]
Ruan F 2013[1039]	63	22200	0.00 [0.00; 0.00]
Xu H 2013[1059−1]	207	1630	0.13 [0.11; 0.14]
Xu H 2013[1059−2]	65	1912	0.03 [0.03; 0.04]
**Common effect model**		**113559**	**0.03 [0.03; 0.03]**
**Random effects model**			**0.06 [0.04; 0.09]**
Heterogeneity: I^2 = 99%, τ^2 = 1.5436, p = 0			
**year = 2014**			
BiHua 2014[10]	95	18930	0.01 [0.00; 0.01]
CaiWenFeng 2014[26]	27	816	0.03 [0.02; 0.05]
CenYongZhuang 2014[39]	32	106	0.30 [0.22; 0.40]
ChaRiSheng 2014[47−1]	36	170	0.21 [0.15; 0.28]
ChaRiSheng 2014[47−2]	29	498	0.06 [0.04; 0.08]
ChaRiSheng 2014[47−3]	43	594	0.07 [0.05; 0.10]
ChaRiSheng 2014[47−4]	18	1111	0.02 [0.01; 0.03]
ChaRiSheng 2014[47−5]	16	1168	0.01 [0.01; 0.02]
ChaRiSheng 2014[47−6]	31	1422	0.02 [0.01; 0.03]
ChaRiSheng 2014[47−7]	74	1749	0.04 [0.03; 0.05]
ChaRiSheng 2014[47−8]	47	1858	0.03 [0.02; 0.03]
ChaRiSheng 2014[47−9]	17	1910	0.01 [0.01; 0.01]
ChaRiSheng 2014[47−10]	168	2002	0.08 [0.07; 0.10]
ChaRiSheng 2014[47−11]	61	2202	0.03 [0.02; 0.04]
ChaRiSheng 2014[47−12]	139	2786	0.05 [0.04; 0.06]
ChaRiSheng 2014[47−13]	39	3095	0.01 [0.01; 0.02]
ChenChun 2014[64]	107	1242	0.09 [0.07; 0.10]
GanXiangYang 2014[172]	74	2200	0.03 [0.03; 0.04]
GaoHaiMing 2014[174]	19	187	0.10 [0.06; 0.15]
GuoShuiLian 2014[209]	16	76	0.21 [0.13; 0.32]
HeHanZhen 2014[224]	76	578	0.13 [0.11; 0.16]
HouYuYuan 2014[231]	63	984	0.06 [0.05; 0.08]
JiangXianChen 2014[283−1]	16	1485	0.01 [0.01; 0.02]
JiangXianChen 2014[283−2]	23	1877	0.01 [0.01; 0.02]
JiangLie 2014[291]	15	590	0.03 [0.01; 0.04]
LaiShiMing 2014[308]	105	7113	0.01 [0.01; 0.02]

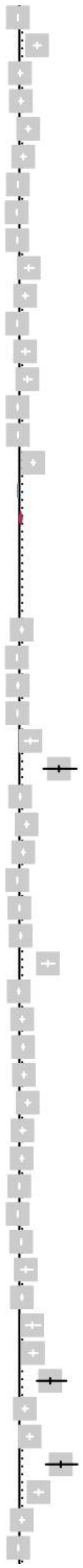

LiYiLan 2014[365]	228	18930	0.01	[0.01; 0.01]
LiuGuoHong 2014[406]	109	636	0.17	[0.14; 0.20]
LongJunBiao 2014[441]	13	444	0.03	[0.02; 0.05]
PangZhiFeng 2014[525]	20	548	0.04	[0.02; 0.06]
TangYuXin 2014[634]	97	994	0.10	[0.08; 0.12]
WangHua 2014[657]	28	497	0.06	[0.04; 0.08]
WuZhiSheng 2014[741]	176	14612	0.01	[0.01; 0.01]
XuanLingFeng 2014[784]	17	9523	0.00	[0.00; 0.00]
YangYiLong 2014[818]	121	18597	0.01	[0.01; 0.01]
YeXiaoLing 2014[833]	27	264	0.10	[0.07; 0.15]
YinHongMei 2014[839]	37	511	0.07	[0.05; 0.10]
YuanJun 2014[854]	652	115469	0.01	[0.01; 0.01]
ZhangHengQiu 2014[869]	18	250	0.07	[0.04; 0.11]
ZhaoJun 2014[916]	26	287	0.09	[0.06; 0.13]
ZhongJianMing 2014[946]	11	1208	0.01	[0.00; 0.02]
Guo Z 2014[1001]	87	7141	0.01	[0.01; 0.02]
Xue C 2014[1060]	278	1995	0.14	[0.12; 0.16]
**Common effect model**		**248655**	**0.01**	**[0.01; 0.01]**
**Random effects model**			**0.03**	**[0.02; 0.05]**
Heterogeneity: $I^2 = 99\%$, $\tau^2 = 1.4597$, $p = 0$				
**year = 2015**				
BiHua 2015[11]	55	1262	0.04	[0.03; 0.06]
ChenAQun 2015[58]	64	19153	0.00	[0.00; 0.00]
ChenXiaoFeng 2015[100]	7	648	0.01	[0.00; 0.02]
ChenYiYi 2015[109]	282	38001	0.01	[0.01; 0.01]
ChuXiuJuan 2015[120]	17	147	0.12	[0.07; 0.18]
GaoHuiJuan 2015[176]	18	51	0.35	[0.22; 0.50]
HuHongAn 2015[233]	105	3453	0.03	[0.02; 0.04]
HuangChunLi 2015[246]	108	1283	0.08	[0.07; 0.10]
HuangGuo 2015[252]	69	1220	0.06	[0.04; 0.07]
HuangGuo 2015[253]	87	13856	0.01	[0.01; 0.01]
JiHong 2015[264]	78	3194	0.02	[0.02; 0.03]
JiangYingCi 2015[284]	66	1904	0.03	[0.03; 0.04]
LiJianSen 2015[328]	70	268	0.26	[0.21; 0.32]
LiYueRong 2015[366]	18	952	0.02	[0.01; 0.03]
LinQin 2015[390]	34	713	0.05	[0.03; 0.07]
LiuBo 2015[398]	104	1883	0.06	[0.05; 0.07]
LuJianYong 2015[456]	60	973	0.06	[0.05; 0.08]
MaTao 2015[486]	84	901	0.09	[0.08; 0.11]
PangZhiMing 2015[526]	19	381	0.05	[0.03; 0.08]
PengXiaoXue 2015[528]	37	849	0.04	[0.03; 0.06]
SongJianQiang 2015[598]	259	10942	0.02	[0.02; 0.03]
SunJing 2015[611]	92	8929	0.01	[0.01; 0.01]
WangJie 2015[659]	451	11467	0.04	[0.04; 0.04]
WangXiaoQin 2015[689]	11	142	0.08	[0.04; 0.13]
WuXiaYan 2015[730]	92	2046	0.04	[0.04; 0.05]
WuZhaoChun 2015[737]	17	130	0.13	[0.08; 0.20]
YangGenMei 2015[798]	54	392	0.14	[0.11; 0.18]
YuKuangMing 2015[851]	16	57	0.28	[0.17; 0.42]
ZhangLi 2015[875−1]	33	474	0.07	[0.05; 0.10]
ZhangLi 2015[875−2]	72	656	0.11	[0.09; 0.14]
ZhangXiaoYi 2015[891]	21	57	0.37	[0.24; 0.51]
ZhangZhen 2015[903]	76	414	0.18	[0.15; 0.22]
ZhaoTengLong 2015[921]	25	564	0.04	[0.03; 0.06]
ZhouXiaoHong 2015[958]	107	7113	0.02	[0.01; 0.02]

Liu Y 2015[1025]	99	2049	0.05 [0.04; 0.06]
Luo LF 2015[1030]	32	45	0.71 [0.56; 0.84]
Zhang XF 2015[1077]	276	2442	0.11 [0.10; 0.13]
XuHaoBin 2015[1091]	39	496	0.08 [0.06; 0.11]
**Common effect model**		**139507**	**0.02 [0.02; 0.02]**
**Random effects model**			**0.06 [0.04; 0.08]**
Heterogeneity: $I^2 = 99\%$, $\tau^2 = 1.6736$, $p = 0$			
**year = 2016**			
ChaRiSheng 2016[46]	22	453	0.05 [0.03; 0.07]
ShenYuGang 2016[54]	43	480	0.09 [0.07; 0.12]
ShenYuGang 2016[55]	233	1702	0.14 [0.12; 0.15]
ChenAQun 2016[59]	64	18924	0.00 [0.00; 0.00]
ChenJian 2016[78]	20	1551	0.01 [0.01; 0.02]
KuangHaoCheng 2016[307]	667	11300	0.06 [0.05; 0.06]
LeiYongLiang 2016[311]	46	984	0.05 [0.03; 0.06]
LiShouJun 2016[350]	64	1023	0.06 [0.05; 0.08]
LinQingShuang 2016[391]	29	603	0.05 [0.03; 0.07]
LiuShiKe 2016[422]	46	948	0.05 [0.04; 0.06]
LongQiZhi 2016[442]	101	1707	0.06 [0.05; 0.07]
LuZhengXiang 2016[451]	83	4624	0.02 [0.01; 0.02]
LuHua 2016[454]	14	213	0.07 [0.04; 0.11]
LuWeiWei 2016[462]	406	3808	0.11 [0.10; 0.12]
MaoJianYing 2016[493]	51	559	0.09 [0.07; 0.12]
MoGuiQiong 2016[504]	26	843	0.03 [0.02; 0.04]
RenYuHua 2016[562]	110	1916	0.06 [0.05; 0.07]
SunZhou 2016[625]	230	45075	0.01 [0.00; 0.01]
WangJinSheng 2016[646]	99	11594	0.01 [0.01; 0.01]
WangShuangYing 2016[676]	16	119	0.13 [0.08; 0.21]
WuHongXing 2016[718]	63	726	0.09 [0.07; 0.11]
XieYuanQi 2016[756]	34	207	0.16 [0.12; 0.22]
XuHao 2016[775]	28	2385	0.01 [0.01; 0.02]
XuJinFeng 2016[778−1]	10	933	0.01 [0.01; 0.02]
XuJinFeng 2016[778−2]	16	2500	0.01 [0.00; 0.01]
YangJiChao 2016[802]	18	137	0.13 [0.08; 0.20]
YangYouQing 2016[819]	48	2363	0.02 [0.02; 0.03]
YaoJianXiang 2016[822]	59	2992	0.02 [0.02; 0.03]
YingLiHong 2016[840]	48	3000	0.02 [0.01; 0.02]
ZhangLing 2016[879]	132	735	0.18 [0.15; 0.21]
ZhaoXueCheng 2016[929]	149	1570	0.09 [0.08; 0.11]
ZhouLiHong 2016[956]	13	37	0.35 [0.20; 0.53]
ZhouYin 2016[964]	12	1061	0.01 [0.01; 0.02]
ZhouZhenQing 2016[966]	28	186	0.15 [0.10; 0.21]
Shi C 2016[1046]	57	1966	0.03 [0.02; 0.04]
Wang X 2016[1055]	51	377	0.14 [0.10; 0.17]
XiangLunHui 2016[1101]	51	533	0.10 [0.07; 0.12]
**Common effect model**		**130134**	**0.02 [0.02; 0.03]**
**Random effects model**			**0.04 [0.03; 0.06]**
Heterogeneity: $I^2 = 99\%$, $\tau^2 = 1.3683$, $p = 0$			
**year = 2017**			
Cai SX 2017[21]	17	1276	0.01 [0.01; 0.02]
ChenBinBin 2017[60−1]	54	392	0.14 [0.11; 0.18]
ChenBinBin 2017[60−2]	161	2122	0.08 [0.06; 0.09]
ChenJianMei 2017[79]	110	9736	0.01 [0.01; 0.01]
ChenJian 2017[80]	69	6323	0.01 [0.01; 0.01]
ChenMinHong 2017[92−1]	21	236	0.09 [0.06; 0.13]

ChenMinHong 2017[92-2]	186	657	0.28 [0.25; 0.32]
ChenMinHong 2017[92-3]	60	4286	0.01 [0.01; 0.02]
ChenZhiQiong 2017[115]	255	1843	0.14 [0.12; 0.15]
DongShengCao 2017[146]	24	200	0.12 [0.08; 0.17]
DuanRong 2017[152-1]	13	77	0.17 [0.09; 0.27]
DuanRong 2017[152-2]	13	81	0.16 [0.09; 0.26]
DuanRong 2017[152-3]	11	89	0.12 [0.06; 0.21]
DuanRong 2017[152-4]	9	93	0.10 [0.05; 0.18]
DuanRong 2017[152-5]	12	165	0.07 [0.04; 0.12]
DuanRong 2017[152-6]	11	179	0.06 [0.03; 0.11]
DuanRong 2017[152-7]	10	182	0.05 [0.03; 0.10]
DuanRong 2017[152-8]	9	199	0.05 [0.02; 0.08]
DuanRong 2017[152-9]	14	215	0.07 [0.04; 0.11]
DuanRong 2017[152-10]	36	227	0.16 [0.11; 0.21]
DuanRong 2017[152-11]	5	229	0.02 [0.01; 0.05]
DuanRong 2017[152-12]	8	238	0.03 [0.01; 0.07]
DuanRong 2017[152-13]	5	245	0.02 [0.01; 0.05]
DuanRong 2017[152-14]	8	249	0.03 [0.01; 0.06]
DuanRong 2017[152-15]	8	271	0.03 [0.01; 0.06]
DuanRong 2017[152-16]	7	441	0.02 [0.01; 0.03]
DuanRong 2017[152-17]	5	495	0.01 [0.00; 0.02]
DuanRong 2017[152-18]	9	538	0.02 [0.01; 0.03]
DuanRong 2017[152-19]	7	565	0.01 [0.00; 0.03]
DuanRong 2017[152-20]	5	590	0.01 [0.00; 0.02]
DuanRong 2017[152-21]	11	699	0.02 [0.01; 0.03]
DuanRong 2017[152-22]	7	701	0.01 [0.00; 0.02]
DuanRong 2017[152-23]	8	706	0.01 [0.00; 0.02]
DuanRong 2017[152-24]	7	733	0.01 [0.00; 0.02]
DuanRong 2017[152-25]	5	753	0.01 [0.00; 0.02]
DuanRong 2017[152-26]	24	802	0.03 [0.02; 0.04]
DuanRong 2017[152-27]	9	918	0.01 [0.00; 0.02]
DuanRong 2017[152-28]	14	927	0.02 [0.01; 0.03]
DuanRong 2017[152-29]	5	976	0.01 [0.00; 0.01]
DuanRong 2017[152-30]	5	976	0.01 [0.00; 0.01]
DuanRong 2017[152-31]	68	1036	0.07 [0.05; 0.08]
DuanRong 2017[152-32]	5	1145	0.00 [0.00; 0.01]
DuanRong 2017[152-33]	13	1216	0.01 [0.01; 0.02]
DuanRong 2017[152-34]	11	1420	0.01 [0.00; 0.01]
GaoJunYing 2017[177]	12	1501	0.01 [0.00; 0.01]
GongShuiYing 2017[194]	93	990	0.09 [0.08; 0.11]
GuoMinJian 2017[208-1]	8	31	0.26 [0.12; 0.45]
GuoMinJian 2017[208-2]	6	39	0.15 [0.06; 0.31]
GuoMinJian 2017[208-3]	11	39	0.28 [0.15; 0.45]
GuoMinJian 2017[208-4]	10	39	0.26 [0.13; 0.42]
GuoMinJian 2017[208-5]	17	39	0.44 [0.28; 0.60]
GuoMinJian 2017[208-6]	7	42	0.17 [0.07; 0.31]
GuoMinJian 2017[208-7]	7	43	0.16 [0.07; 0.31]
GuoMinJian 2017[208-8]	7	53	0.13 [0.05; 0.25]
GuoMinJian 2017[208-9]	16	1488	0.01 [0.01; 0.02]
GuoMinJian 2017[208-10]	48	2200	0.02 [0.02; 0.03]
JiangYiMei 2017[294]	15	3050	0.00 [0.00; 0.01]
LiangRiCheng 2017[376]	27	2518	0.01 [0.01; 0.02]
LiuTian 2017[424-1]	16	39	0.41 [0.26; 0.58]
LiuTian 2017[424-2]	11	42	0.26 [0.14; 0.42]
LiuTian 2017[424-3]	32	44	0.73 [0.57; 0.85]
LiuTian 2017[424-4]	9	48	0.19 [0.09; 0.33]

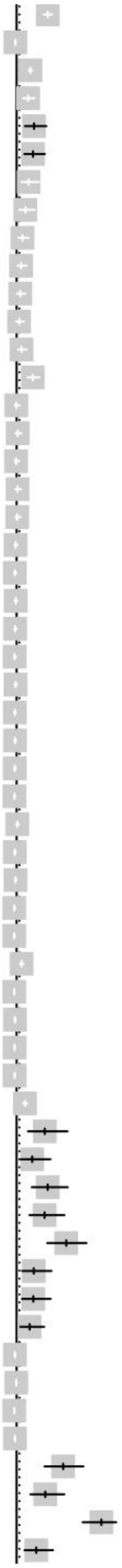

LiuTian 2017[424−5]	14	84	0.17	[0.09; 0.26]
LiuTian 2017[424−6]	70	213	0.33	[0.27; 0.40]
LiuTian 2017[424−7]	35	387	0.09	[0.06; 0.12]
LuoLe 2017[472]	39	1192	0.03	[0.02; 0.04]
PanYiFeng 2017[521]	82	7396	0.01	[0.01; 0.01]
PangZhiFeng 2017[524]	79	1696	0.05	[0.04; 0.06]
PuPeiLong 2017[529]	98	1126	0.09	[0.07; 0.11]
SongCanLei 2017[593]	36	888	0.04	[0.03; 0.06]
SunMingHua 2017[614]	36	496	0.07	[0.05; 0.10]
TangYuHuan 2017[630]	44	2494	0.02	[0.01; 0.02]
WangMan 2017[668]	85	540	0.16	[0.13; 0.19]
WuYiLing 2017[736]	31	1311	0.02	[0.02; 0.03]
XiaoSongJian 2017[745]	51	416	0.12	[0.09; 0.16]
XieCaiWen 2017[749]	12	58	0.21	[0.11; 0.33]
YanChaoYang 2017[792]	90	2168	0.04	[0.03; 0.05]
YangCheng 2017[797]	109	330	0.33	[0.28; 0.38]
YaoJing 2017[824]	179	885	0.20	[0.18; 0.23]
YeXianMing 2017[832]	94	1156	0.08	[0.07; 0.10]
YueYong 2017[859]	12	150	0.08	[0.04; 0.14]
ZhangZhengDong 2017[904]	25	466	0.05	[0.04; 0.08]
ZhouAiHua 2017[950]	80	650	0.12	[0.10; 0.15]
ZhouXiaoHong 2017[961]	47	3464	0.01	[0.01; 0.02]
ZhouXiaoMin 2017[962]	47	3917	0.01	[0.01; 0.02]
ZhuChengMing 2017[967]	78	4454	0.02	[0.01; 0.02]
ZongJun 2017[980]	45	78	0.58	[0.46; 0.69]
Cui C 2017[994]	34	400	0.08	[0.06; 0.12]
Shang X 2017[1041]	924	23068	0.04	[0.04; 0.04]
Zhang TL 2017[1075]	20	327	0.06	[0.04; 0.09]
**Common effect model**		**117502**	**0.04**	**[0.03; 0.04]**
**Random effects model**			**0.05**	**[0.04; 0.07]**
Heterogeneity: $I^2 = 98\%$, $\tau^2 = 2.0084$, $p = 0$				
**year = 2018**				
Cai MW 2018[18]	34	223	0.15	[0.11; 0.21]
Cai SJ 2018[20]	69	1781	0.04	[0.03; 0.05]
CaoXiaoPing 2018[33]	113	1037	0.11	[0.09; 0.13]
ZengLei 2018[44]	28	388	0.07	[0.05; 0.10]
ShenYiPing 2018[52]	26	612	0.04	[0.03; 0.06]
ChenCan 2018[63]	24	61	0.39	[0.27; 0.53]
ChenHeJuan 2018[73]	14	49	0.29	[0.17; 0.43]
ChenJingFang 2018[84−1]	18	316	0.06	[0.03; 0.09]
ChenJingFang 2018[84−2]	14	360	0.04	[0.02; 0.06]
ChenXingFu 2018[103]	43	1240	0.03	[0.03; 0.05]
ChenYiXiong 2018[108]	21	1861	0.01	[0.01; 0.02]
DaiYingXue 2018[129−1]	14	378	0.04	[0.02; 0.06]
DaiYingXue 2018[129−2]	12	547	0.02	[0.01; 0.04]
DaiYingXue 2018[129−3]	18	1008	0.02	[0.01; 0.03]
DaiYingXue 2018[129−4]	14	1053	0.01	[0.01; 0.02]
FengZhi 2018[162]	111	2020	0.05	[0.05; 0.07]
GuiGuoPing 2018[200]	79	1204	0.07	[0.05; 0.08]
JiJinHua 2018[265]	41	694	0.06	[0.04; 0.08]
JiLei 2018[272−1]	7	55	0.13	[0.05; 0.24]
JiLei 2018[272−2]	16	92	0.17	[0.10; 0.27]
JiLei 2018[272−3]	18	191	0.09	[0.06; 0.14]
LiDaiBo 2018[320]	18	56	0.32	[0.20; 0.46]
LiShiCong 2018[346]	64	1487	0.04	[0.03; 0.05]

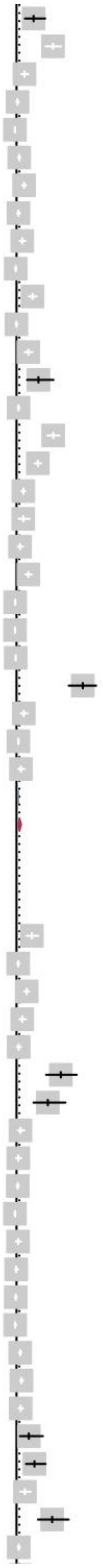

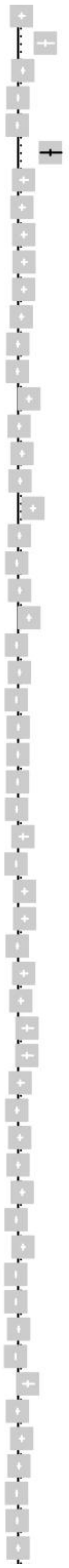

LiXiuFang 2018[359]	20	378	0.05 [0.03; 0.08]
LiaoKeChang 2018[381]	41	161	0.25 [0.19; 0.33]
LinQiFeng 2018[388]	96	1494	0.06 [0.05; 0.08]
LinYanYan 2018[393]	76	3288	0.02 [0.02; 0.03]
LiuHongLian 2018[411]	19	1119	0.02 [0.01; 0.03]
LiuJingJing 2018[413]	33	111	0.30 [0.21; 0.39]
LiuWenJun 2018[428−1]	18	255	0.07 [0.04; 0.11]
LiuWenJun 2018[428−2]	22	388	0.06 [0.04; 0.08]
LiuWenJun 2018[428−3]	38	528	0.07 [0.05; 0.10]
LiuWenJun 2018[428−4]	42	565	0.07 [0.05; 0.10]
LiuWenJun 2018[428−5]	48	672	0.07 [0.05; 0.09]
LiuWenJun 2018[428−6]	36	700	0.05 [0.04; 0.07]
LiuWenJun 2018[428−7]	18	826	0.02 [0.01; 0.03]
LiuWenJun 2018[428−8]	16	904	0.02 [0.01; 0.03]
LiuWenJun 2018[428−9]	105	907	0.12 [0.10; 0.14]
LiuWenJun 2018[428−10]	31	951	0.03 [0.02; 0.05]
LiuWenJun 2018[428−11]	55	955	0.06 [0.04; 0.07]
LiuWenJun 2018[428−12]	49	1213	0.04 [0.03; 0.05]
LiuWenJun 2018[428−13]	193	1285	0.15 [0.13; 0.17]
LiuWenJun 2018[428−14]	46	1314	0.04 [0.03; 0.05]
LiuWenJun 2018[428−15]	17	1328	0.01 [0.01; 0.02]
LiuWenJun 2018[428−16]	54	1467	0.04 [0.03; 0.05]
LiuWenJun 2018[428−17]	180	1554	0.12 [0.10; 0.13]
LiuWenJun 2018[428−18]	19	1776	0.01 [0.01; 0.02]
LiuWenJun 2018[428−19]	68	1926	0.04 [0.03; 0.04]
LiuWenJun 2018[428−20]	17	1932	0.01 [0.01; 0.01]
LiuWenJun 2018[428−21]	55	2174	0.03 [0.02; 0.03]
LiuWenJun 2018[428−22]	52	2332	0.02 [0.02; 0.03]
LiuWenJun 2018[428−23]	70	3608	0.02 [0.02; 0.02]
LiuWenJun 2018[428−24]	43	3707	0.01 [0.01; 0.02]
LuoTengXian 2018[475]	13	200	0.06 [0.04; 0.11]
MaMengMeng 2018[484]	223	30711	0.01 [0.01; 0.01]
MaTao 2018[487]	46	587	0.08 [0.06; 0.10]
MengJian 2018[495]	37	465	0.08 [0.06; 0.11]
MoYuJie 2018[505]	19	1117	0.02 [0.01; 0.03]
MiaoGuoZhong 2018[507]	25	350	0.07 [0.05; 0.10]
QiYanQiu 2018[532]	27	570	0.05 [0.03; 0.07]
RuiFang 2018[566−1]	18	180	0.10 [0.06; 0.15]
RuiFang 2018[566−2]	18	183	0.10 [0.06; 0.15]
RuiFang 2018[566−3]	11	263	0.04 [0.02; 0.07]
RuiFang 2018[566−4]	5	382	0.01 [0.00; 0.03]
RuiFang 2018[566−5]	18	473	0.04 [0.02; 0.06]
RuiFang 2018[566−6]	16	670	0.02 [0.01; 0.04]
RuiFang 2018[566−7]	54	908	0.06 [0.04; 0.08]
RuiFang 2018[566−8]	8	1059	0.01 [0.00; 0.01]
RuiFang 2018[566−9]	78	1184	0.07 [0.05; 0.08]
RuiFang 2018[566−10]	10	2084	0.00 [0.00; 0.01]
RuiFang 2018[566−11]	15	2905	0.01 [0.00; 0.01]
SunYunLan 2018[624]	63	2212	0.03 [0.02; 0.04]
WangHu 2018[656]	72	28270	0.00 [0.00; 0.00]
WangMin 2018[670−1]	19	180	0.11 [0.06; 0.16]
WangMin 2018[670−2]	11	599	0.02 [0.01; 0.03]
WangMin 2018[670−3]	37	672	0.06 [0.04; 0.08]
WangMin 2018[670−4]	27	861	0.03 [0.02; 0.05]
WangMin 2018[670−5]	32	2843	0.01 [0.01; 0.02]
WangMin 2018[670−6]	47	2869	0.02 [0.01; 0.02]
WenYa 2018[710]	16	668	0.02 [0.01; 0.04]

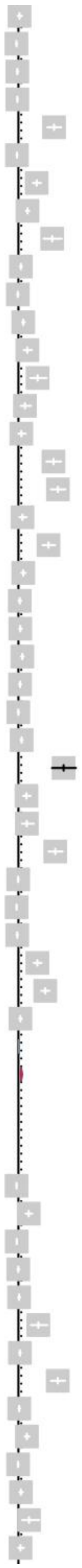

WuGuoFu 2018[717]	17	506	0.03 [0.02; 0.05]
WuMingXiong 2018[723]	10	1292	0.01 [0.00; 0.01]
WuQinDi 2018[725]	24	1578	0.02 [0.01; 0.02]
WuWenQian 2018[729]	36	2399	0.02 [0.01; 0.02]
WuYang 2018[733]	85	265	0.32 [0.26; 0.38]
WuYang 2018[734]	26	2179	0.01 [0.01; 0.02]
WuYang 2018[735]	91	516	0.18 [0.14; 0.21]
XiaYingPin 2018[743]	177	1777	0.10 [0.09; 0.11]
XiaoDaYong 2018[746]	47	154	0.31 [0.23; 0.38]
XuJianRong 2018[767]	92	2046	0.04 [0.04; 0.05]
YangCaiBin 2018[796]	42	2159	0.02 [0.01; 0.03]
YangJing 2018[804]	111	1718	0.06 [0.05; 0.08]
YangSenPing 2018[810]	30	305	0.10 [0.07; 0.14]
Jin−Bo Ye 2018[830−1]	25	136	0.18 [0.12; 0.26]
Jin−Bo Ye 2018[830−2]	14	178	0.08 [0.04; 0.13]
Jin−Bo Ye 2018[830−3]	11	227	0.05 [0.02; 0.09]
Jin−Bo Ye 2018[830−4]	73	231	0.32 [0.26; 0.38]
Jin−Bo Ye 2018[830−5]	90	255	0.35 [0.29; 0.42]
Jin−Bo Ye 2018[830−6]	15	259	0.06 [0.03; 0.09]
Jin−Bo Ye 2018[830−7]	81	295	0.27 [0.22; 0.33]
Jin−Bo Ye 2018[830−8]	49	760	0.06 [0.05; 0.08]
Jin−Bo Ye 2018[830−9]	40	1125	0.04 [0.03; 0.05]
Jin−Bo Ye 2018[830−10]	55	1377	0.04 [0.03; 0.05]
Jin−Bo Ye 2018[830−11]	101	1784	0.06 [0.05; 0.07]
Jin−Bo Ye 2018[830−12]	77	2034	0.04 [0.03; 0.05]
Jin−Bo Ye 2018[830−13]	60	2572	0.02 [0.02; 0.03]
Jin−Bo Ye 2018[830−14]	132	2584	0.05 [0.04; 0.06]
ZhangHuiLing 2018[871]	40	100	0.40 [0.30; 0.50]
ZhangNing 2018[882]	31	340	0.09 [0.06; 0.13]
ZhangGuangMing 2018[907]	14	149	0.09 [0.05; 0.15]
ZhaoMengJiao 2018[919]	87	263	0.33 [0.27; 0.39]
ZhaoYiNan 2018[932]	67	3000	0.02 [0.02; 0.03]
ZhuWeiWei 2018[976]	91	9702	0.01 [0.01; 0.01]
Li J 2018[1015]	19	1327	0.01 [0.01; 0.02]
Qi L 2018[1034]	101	555	0.18 [0.15; 0.22]
Tsang TK 2018[1050]	159	643	0.25 [0.21; 0.28]
LiYing 2018[1097]	53	1288	0.04 [0.03; 0.05]
**Common effect model**		**188074**	**0.03 [0.03; 0.03]**
**Random effects model**			**0.05 [0.04; 0.06]**
Heterogeneity: $I^2 = 98\%$, $\tau^2 = 1.3208$, $p = 0$			
**year = 2019**			
CaoShen 2019[32]	62	6966	0.01 [0.01; 0.01]
ChenYan 2019[104]	59	536	0.11 [0.08; 0.14]
DengXingChao 2019[133]	439	42403	0.01 [0.01; 0.01]
DuYueHe 2019[148]	29	1250	0.02 [0.02; 0.03]
GaoShuPing 2019[183]	51	1698	0.03 [0.02; 0.04]
JiangChen 2019[285]	26	137	0.19 [0.13; 0.27]
LiJie 2019[329]	106	2868	0.04 [0.03; 0.04]
LiShiCong 2019[345]	91	259	0.35 [0.29; 0.41]
LinJian 2019[384]	77	2476	0.03 [0.02; 0.04]
LiuDan 2019[403]	90	951	0.09 [0.08; 0.12]
LiuDongSheng 2019[404]	80	3720	0.02 [0.02; 0.03]
LiuQingLian 2019[420]	37	817	0.05 [0.03; 0.06]
LiuTian 2019[423]	10	89	0.11 [0.06; 0.20]
LiuYing 2019[437]	14	335	0.04 [0.02; 0.07]

NiChaoRong 2019[510]	125	3622	0.03	[0.03; 0.04]
NiChaoRong 2019[511]	28	2158	0.01	[0.01; 0.02]
OuSheXiang 2019[515]	18	603	0.03	[0.02; 0.05]
QiXiaoQi 2019[534]	185	2418	0.08	[0.07; 0.09]
SongYuFang 2019[600]	63	783	0.08	[0.06; 0.10]
SunQin 2019[616]	32	128	0.25	[0.18; 0.33]
TangGuoJie 2019[631]	84	2250	0.04	[0.03; 0.05]
WangJinSheng 2019[645]	56	1800	0.03	[0.02; 0.04]
WangKaiLiang 2019[665]	16	446	0.04	[0.02; 0.06]
WeiGuiYing 2019[706]	29	257	0.11	[0.08; 0.16]
WenYingMing 2019[711]	15	35	0.43	[0.26; 0.61]
XiaGuangHui 2019[742]	106	2300	0.05	[0.04; 0.06]
YangJiXing 2019[801]	30	808	0.04	[0.03; 0.05]
YangRongXing 2019[808]	15	334	0.04	[0.03; 0.07]
YeShuJun 2019[831]	104	399	0.26	[0.22; 0.31]
ZhanYueWang 2019[860]	38	2311	0.02	[0.01; 0.02]
ZhaoMeiLing 2019[918−1]	8	842	0.01	[0.00; 0.02]
ZhaoMeiLing 2019[918−2]	39	3305	0.01	[0.01; 0.02]
ZhaoYuLi 2019[933]	119	1784	0.07	[0.06; 0.08]
ZhuChunXiu 2019[968]	17	401	0.04	[0.02; 0.07]
Zhou X 2019[1080]	75	1612	0.05	[0.04; 0.06]
**Common effect model**		**93101**	**0.03**	**[0.02; 0.03]**
**Random effects model**			**0.05**	**[0.03; 0.07]**
Heterogeneity: $I^2 = 99\%$, $\tau^2 = 1.1539$, $p = 0$				
**year = 2020**				
Cao RR 2020[30−1]	41	2853	0.01	[0.01; 0.02]
ZengFengMei 2020[41]	25	255	0.10	[0.06; 0.14]
ChenCaiRong 2020[62]	16	275	0.06	[0.03; 0.09]
ChenXin 2020[101]	3	169	0.02	[0.00; 0.05]
DaiBenNa 2020[127]	117	3119	0.04	[0.03; 0.04]
FengZhi 2020[161]	26	50	0.52	[0.37; 0.66]
GuYiFu 2020[197]	45	1903	0.02	[0.02; 0.03]
HuangBinBin 2020[245]	41	2530	0.02	[0.01; 0.02]
HuangLiQing 2020[254]	184	1823	0.10	[0.09; 0.12]
HuangSiYue 2020[257]	90	19800	0.00	[0.00; 0.01]
JiLei 2020[270]	19	34	0.56	[0.38; 0.73]
JiRuPing 2020[274]	119	2030	0.06	[0.05; 0.07]
LiMeng 2020[338−1]	7	74	0.09	[0.04; 0.19]
LiMeng 2020[338−2]	16	204	0.08	[0.05; 0.12]
LiMeng 2020[338−3]	7	260	0.03	[0.01; 0.05]
LiMeng 2020[338−4]	6	358	0.02	[0.01; 0.04]
LiMeng 2020[338−5]	6	369	0.02	[0.01; 0.04]
LiMeng 2020[338−6]	7	416	0.02	[0.01; 0.03]
LiMeng 2020[338−7]	11	476	0.02	[0.01; 0.04]
LiMeng 2020[338−8]	11	659	0.02	[0.01; 0.03]
LiMeng 2020[338−9]	12	749	0.02	[0.01; 0.03]
LiMeng 2020[338−10]	13	754	0.02	[0.01; 0.03]
LiMeng 2020[338−11]	19	1168	0.02	[0.01; 0.03]
LiMeng 2020[338−12]	9	1462	0.01	[0.00; 0.01]
LiMeng 2020[338−13]	17	1580	0.01	[0.01; 0.02]
LiMeng 2020[338−14]	85	1814	0.05	[0.04; 0.06]
LiMeng 2020[338−15]	6	1863	0.00	[0.00; 0.01]
LiMeng 2020[338−16]	17	2026	0.01	[0.00; 0.01]
LiMeng 2020[338−17]	5	2026	0.00	[0.00; 0.01]
LiShiCong 2020[344]	181	3622	0.05	[0.04; 0.06]

LiuHaoHui 2020[410−1]	6	35	0.17	[0.07; 0.34]
LiuHaoHui 2020[410−2]	28	53	0.53	[0.39; 0.67]
LiuHaoHui 2020[410−3]	10	82	0.12	[0.06; 0.21]
LiuHaoHui 2020[410−4]	14	166	0.08	[0.05; 0.14]
LiuHaoHui 2020[410−5]	9	202	0.04	[0.02; 0.08]
LiuHaoHui 2020[410−6]	16	300	0.05	[0.03; 0.09]
LiuHaoHui 2020[410−7]	12	561	0.02	[0.01; 0.04]
LiuHaoHui 2020[410−8]	145	618	0.23	[0.20; 0.27]
LiuHaoHui 2020[410−9]	17	2191	0.01	[0.00; 0.01]
LuCaiFang 2020[458]	19	499	0.04	[0.02; 0.06]
QianLiZhen 2020[537]	23	95	0.24	[0.16; 0.34]
Wu−Yang Shi 2020[577−1]	35	441	0.08	[0.06; 0.11]
SongCanLei 2020[590]	44	812	0.05	[0.04; 0.07]
SunLiYan 2020[613]	11	51	0.22	[0.11; 0.35]
TaoLiYan 2020[636]	47	1857	0.03	[0.02; 0.03]
WangDaHu 2020[652]	182	33479	0.01	[0.00; 0.01]
WangMeiHuan 2020[669]	12	433	0.03	[0.01; 0.05]
WangTieJun 2020[678]	15	345	0.04	[0.02; 0.07]
WuJingWen 2020[719]	34	1153	0.03	[0.02; 0.04]
ZhangPan 2020[883]	16	244	0.07	[0.04; 0.10]
ZhangZhiZhong 2020[906]	263	17752	0.01	[0.01; 0.02]
ZhangGuoBao 2020[908]	13	105	0.12	[0.07; 0.20]
ZhangQingHe 2020[909]	109	538	0.20	[0.17; 0.24]
ShenJingQiang 2020[993]	15	37	0.41	[0.25; 0.58]
Huang Z 2020[1008]	68	786	0.09	[0.07; 0.11]
Lu Y 2020[1029]	157	785	0.20	[0.17; 0.23]
Zhang XF 2020[1076]	27	450	0.06	[0.04; 0.09]
**Common effect model**		**118791**	**0.02**	**[0.02; 0.02]**
**Random effects model**			**0.04**	**[0.03; 0.06]**
Heterogeneity: $I^2 = 99\%$, $\tau^2 = 1.9567$, $p = 0$				
**year = 2021**				
Ming−Wei Cai 2021[17−1]	25	628	0.04	[0.03; 0.06]
Ming−Wei Cai 2021[17−2]	50	693	0.07	[0.05; 0.09]
Ming−Wei Cai 2021[17−3]	27	816	0.03	[0.02; 0.05]
Ming−Wei Cai 2021[17−4]	56	1202	0.05	[0.04; 0.06]
ChenQuan 2021[96]	84	821	0.10	[0.08; 0.13]
HuangShiTeng 2021[256]	61	1644	0.04	[0.03; 0.05]
LiaoChan 2021[380]	19	675	0.03	[0.02; 0.04]
WangJun 2021[664]	20	4743	0.00	[0.00; 0.01]
WuXiaoMin 2021[732]	159	1378	0.12	[0.10; 0.13]
HuangJiaCheng 2021[944]	48	5682	0.01	[0.01; 0.01]
HuangKaiXiong 2021[989]	36	660	0.05	[0.04; 0.07]
LiXiaoJing 2021[992]	7	251	0.03	[0.01; 0.06]
Y Li 2021[1018]	143	346	0.41	[0.36; 0.47]
WuLin 2021[1035]	17	194	0.09	[0.05; 0.14]
**Common effect model**		**19733**	**0.04**	**[0.04; 0.04]**
**Random effects model**			**0.05**	**[0.03; 0.08]**
Heterogeneity: $I^2 = 99\%$, $\tau^2 = 1.3731$, $p < 0.01$				
**Common effect model**		**1312440**	**0.02**	**[0.02; 0.02]**
**Random effects model**			**0.05**	**[0.04; 0.05]**

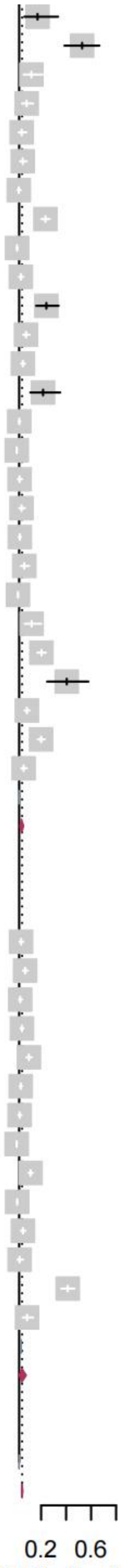

Heterogeneity: $I^2 = 99\%$, $\tau^2 = 1.6181$, $p = 0$
Test for subgroup differences (common effect): $\chi^2_{12} = 3307.78$, df = 12 ($p = 0$)
Test for subgroup differences (random effects): $\chi^2_{12} = 41.64$, df = 12 ($p < 0.01$)

(b3)

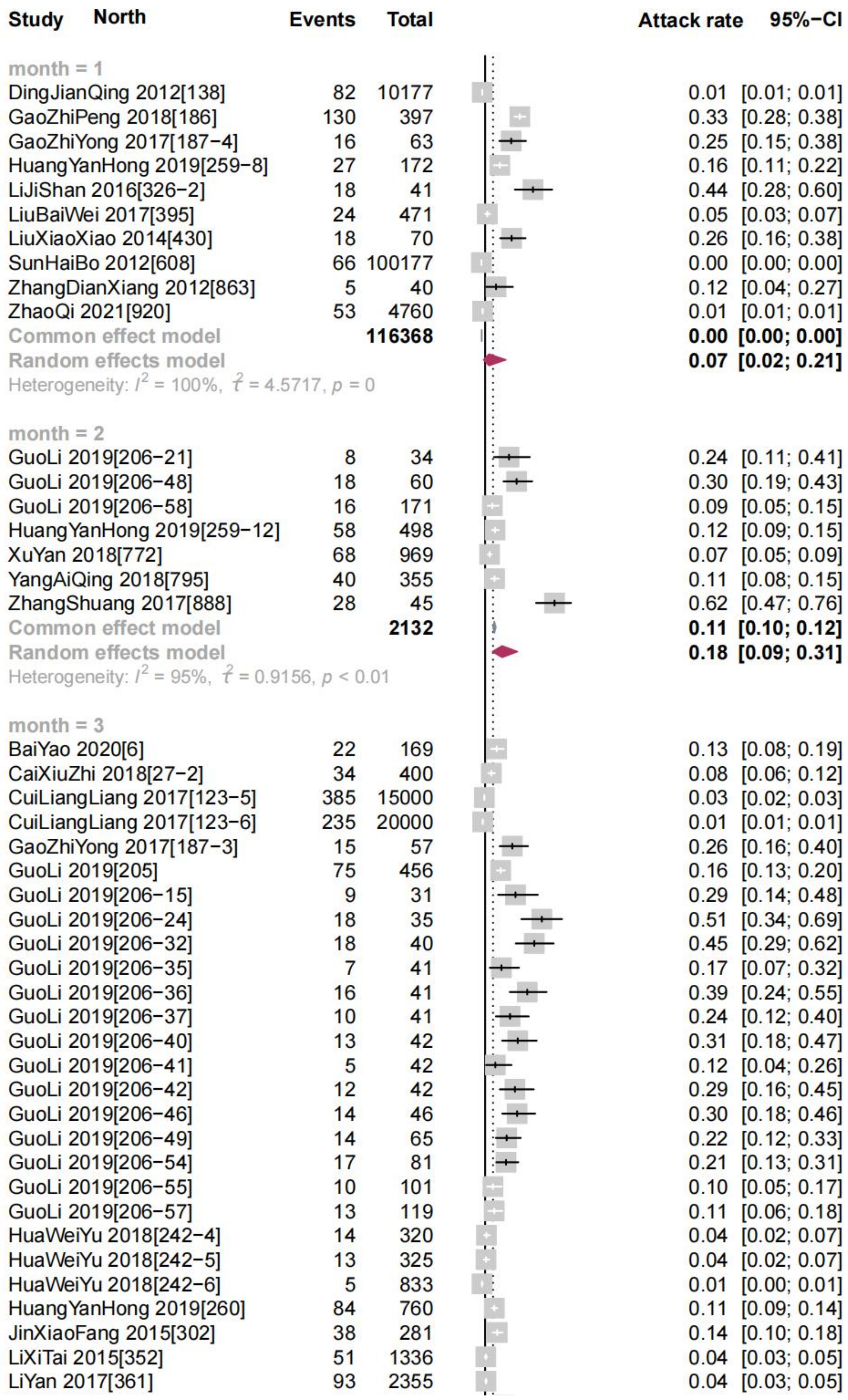
Study North Events Total Attack rate 95%−CI
month = 1
DingJianQing 2012[138] 82 10177 0.01 [0.01; 0.01]
GaoZhiPeng 2018[186] 130 397 0.33 [0.28; 0.38]
GaoZhiYong 2017[187−4] 16 63 0.25 [0.15; 0.38]
HuangYanHong 2019[259−8] 27 172 0.16 [0.11; 0.22]
LiJiShan 2016[326−2] 18 41 0.44 [0.28; 0.60]
LiuBaiWei 2017[395] 24 471 0.05 [0.03; 0.07]
LiuXiaoXiao 2014[430] 18 70 0.26 [0.16; 0.38]
SunHaiBo 2012[608] 66 100177 0.00 [0.00; 0.00]
ZhangDianXiang 2012[863] 5 40 0.12 [0.04; 0.27]
ZhaoQi 2021[920] 53 4760 0.01 [0.01; 0.01]
Common effect model 116368 0.00 [0.00; 0.00]
Random effects model 0.07 [0.02; 0.21]
Heterogeneity: I^2 = 100%, τ^2 = 4.5717, p = 0
month = 2
GuoLi 2019[206−21] 8 34 0.24 [0.11; 0.41]
GuoLi 2019[206−48] 18 60 0.30 [0.19; 0.43]
GuoLi 2019[206−58] 16 171 0.09 [0.05; 0.15]
HuangYanHong 2019[259−12] 58 498 0.12 [0.09; 0.15]
XuYan 2018[772] 68 969 0.07 [0.05; 0.09]
YangAiQing 2018[795] 40 355 0.11 [0.08; 0.15]
ZhangShuang 2017[888] 28 45 0.62 [0.47; 0.76]
Common effect model 2132 0.11 [0.10; 0.12]
Random effects model 0.18 [0.09; 0.31]
Heterogeneity: I^2 = 95%, τ^2 = 0.9156, p < 0.01
month = 3
BaiYao 2020[6] 22 169 0.13 [0.08; 0.19]
CaiXiuZhi 2018[27−2] 34 400 0.08 [0.06; 0.12]
CuiLiangLiang 2017[123−5] 385 15000 0.03 [0.02; 0.03]
CuiLiangLiang 2017[123−6] 235 20000 0.01 [0.01; 0.01]
GaoZhiYong 2017[187−3] 15 57 0.26 [0.16; 0.40]
GuoLi 2019[205] 75 456 0.16 [0.13; 0.20]
GuoLi 2019[206−15] 9 31 0.29 [0.14; 0.48]
GuoLi 2019[206−24] 18 35 0.51 [0.34; 0.69]
GuoLi 2019[206−32] 18 40 0.45 [0.29; 0.62]
GuoLi 2019[206−35] 7 41 0.17 [0.07; 0.32]
GuoLi 2019[206−36] 16 41 0.39 [0.24; 0.55]
GuoLi 2019[206−37] 10 41 0.24 [0.12; 0.40]
GuoLi 2019[206−40] 13 42 0.31 [0.18; 0.47]
GuoLi 2019[206−41] 5 42 0.12 [0.04; 0.26]
GuoLi 2019[206−42] 12 42 0.29 [0.16; 0.45]
GuoLi 2019[206−46] 14 46 0.30 [0.18; 0.46]
GuoLi 2019[206−49] 14 65 0.22 [0.12; 0.33]
GuoLi 2019[206−54] 17 81 0.21 [0.13; 0.31]
GuoLi 2019[206−55] 10 101 0.10 [0.05; 0.17]
GuoLi 2019[206−57] 13 119 0.11 [0.06; 0.18]
HuaWeiYu 2018[242−4] 14 320 0.04 [0.02; 0.07]
HuaWeiYu 2018[242−5] 13 325 0.04 [0.02; 0.07]
HuaWeiYu 2018[242−6] 5 833 0.01 [0.00; 0.01]
HuangYanHong 2019[260] 84 760 0.11 [0.09; 0.14]
JinXiaoFang 2015[302] 38 281 0.14 [0.10; 0.18]
LiXiTai 2015[352] 51 1336 0.04 [0.03; 0.05]
LiYan 2017[361] 93 2355 0.04 [0.03; 0.05]

LiuBaiWei 2017[396−1]	43	222	0.19	[0.14; 0.25]
LiuYuan 2016[438−2]	121	378	0.32	[0.27; 0.37]
LuXiuZhi 2018[463]	47	3600	0.01	[0.01; 0.02]
QiYing 2019[535−6]	8	83	0.10	[0.04; 0.18]
QiYing 2019[535−8]	10	148	0.07	[0.03; 0.12]
QiYing 2019[535−9]	34	1015	0.03	[0.02; 0.05]
RenQiZhi 2018[557−1]	6	100	0.06	[0.02; 0.13]
SongHuiRong 2017[597]	34	568	0.06	[0.04; 0.08]
SuTong 2020[604]	55	3432	0.02	[0.01; 0.02]
TianJing 2017[641]	39	392	0.10	[0.07; 0.13]
WangMingLiang 2020[671]	107	550	0.19	[0.16; 0.23]
WeiXia 2019[708]	22	1024	0.02	[0.01; 0.03]
YuXiaoYun 2018[845]	78	1007	0.08	[0.06; 0.10]
ZhangHaiYan 2016[868]	78	508	0.15	[0.12; 0.19]
ZhuHaiYang 2016[971]	140	1319	0.11	[0.09; 0.12]
Huang XY 2017[1007]	753	22861	0.03	[0.03; 0.04]
Common effect model		**80266**	**0.04**	**[0.03; 0.04]**
Random effects model			**0.10**	**[0.07; 0.13]**
Heterogeneity: $I^2 = 98\%$, $\tau^2 = 1.3595$, $p = 0$				
month = 4				
Cai W 2017[24−7]	8	39	0.21	[0.09; 0.36]
Cai W 2017[24−9]	12	43	0.28	[0.15; 0.44]
Cai W 2017[24−12]	16	78	0.21	[0.12; 0.31]
CuiLiangLiang 2017[123−2]	14	4600	0.00	[0.00; 0.01]
CuiXiaoMan 2018[125]	17	829	0.02	[0.01; 0.03]
FangYunXia 2018[157]	27	357	0.08	[0.05; 0.11]
GaoRiHong 2019[182]	30	72	0.42	[0.30; 0.54]
GuoLi 2019[206−22]	10	34	0.29	[0.15; 0.47]
GuoLi 2019[206−25]	16	35	0.46	[0.29; 0.63]
GuoLi 2019[206−38]	18	41	0.44	[0.28; 0.60]
GuoLi 2019[206−47]	9	46	0.20	[0.09; 0.34]
GuoLi 2019[206−51]	18	72	0.25	[0.16; 0.37]
GuoLi 2019[206−53]	23	80	0.29	[0.19; 0.40]
GuoLi 2019[206−61]	28	281	0.10	[0.07; 0.14]
GuoLi 2019[206−63]	30	440	0.07	[0.05; 0.10]
GuoLi 2019[206−64]	25	498	0.05	[0.03; 0.07]
GuoLi 2019[206−65]	28	698	0.04	[0.03; 0.06]
HuaWeiYu 2018[241]	63	1951	0.03	[0.02; 0.04]
HuaWeiYu 2018[242−7]	13	1806	0.01	[0.00; 0.01]
LiShiE 2018[347−9]	10	221	0.05	[0.02; 0.08]
QiYing 2018[535−1]	173	1621	0.11	[0.09; 0.12]
QiYing 2018[535−2]	71	1621	0.04	[0.03; 0.05]
QiYing 2019[535−7]	8	129	0.06	[0.03; 0.12]
QiYing 2019[535−10]	72	1192	0.06	[0.05; 0.08]
QiYing 2019[535−11]	159	1448	0.11	[0.09; 0.13]
RenQiZhi 2018[557−2]	12	150	0.08	[0.04; 0.14]
SunWenLong 2018[618]	8	142	0.06	[0.02; 0.11]
TianYaLin 2021[642]	38	546	0.07	[0.05; 0.09]
XieBin 2018[757]	95	5000	0.02	[0.02; 0.02]
ZhangQin 2020[885]	57	4279	0.01	[0.01; 0.02]
ZhangYan 2017[894−1]	8	37	0.22	[0.10; 0.38]
ZhangYan 2017[894−2]	12	2857	0.00	[0.00; 0.01]
ZhaoWeiQin 2020[923]	128	4927	0.03	[0.02; 0.03]
ZhouGuoYing 2016[952]	12	75	0.16	[0.09; 0.26]
Common effect model		**36245**	**0.03**	**[0.03; 0.04]**
Random effects model			**0.08**	**[0.05; 0.12]**
Heterogeneity: $I^2 = 97\%$, $\tau^2 = 1.9021$, $p < 0.01$				

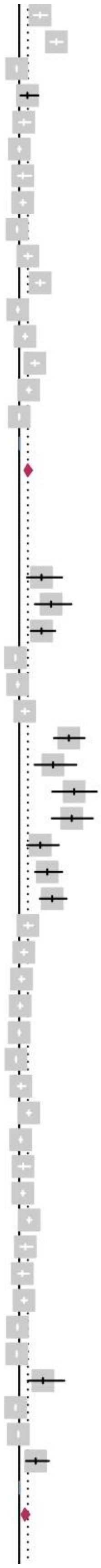

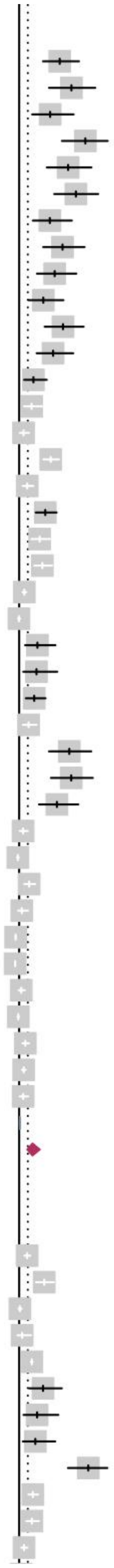

**month = 5**			
Cai W 2017[24−10]	17	49	0.35 [0.22; 0.50]
GuoLi 2019[206−18]	14	32	0.44 [0.26; 0.62]
GuoLi 2019[206−19]	9	33	0.27 [0.13; 0.46]
GuoLi 2019[206−20]	18	33	0.55 [0.36; 0.72]
GuoLi 2019[206−23]	14	34	0.41 [0.25; 0.59]
GuoLi 2019[206−27]	17	36	0.47 [0.30; 0.65]
GuoLi 2019[206−28]	10	37	0.27 [0.14; 0.44]
GuoLi 2019[206−29]	14	38	0.37 [0.22; 0.54]
GuoLi 2019[206−31]	12	39	0.31 [0.17; 0.48]
GuoLi 2019[206−39]	9	41	0.22 [0.11; 0.38]
GuoLi 2019[206−43]	16	43	0.37 [0.23; 0.53]
GuoLi 2019[206−44]	13	44	0.30 [0.17; 0.45]
GuoLi 2019[206−50]	10	70	0.14 [0.07; 0.25]
GuoLi 2019[206−56]	13	101	0.13 [0.07; 0.21]
GuoLi 2019[206−59]	14	211	0.07 [0.04; 0.11]
GuoLi 2019[206−60]	61	220	0.28 [0.22; 0.34]
HuGuangYi 2017[232]	20	210	0.10 [0.06; 0.14]
HuangYanHong 2019[259−3]	27	115	0.23 [0.16; 0.32]
HuangYanHong 2019[259−4]	23	120	0.19 [0.13; 0.27]
HuangYanHong 2019[259−7]	31	146	0.21 [0.15; 0.29]
HuangYanHong 2019[261]	86	1190	0.07 [0.06; 0.09]
KangQian 2020[303−2]	50	1622	0.03 [0.02; 0.04]
LiJiShan 2016[326−3]	8	46	0.17 [0.08; 0.31]
LiShiE 2018[347−1]	6	36	0.17 [0.06; 0.33]
LiShiE 2018[347−6]	14	94	0.15 [0.08; 0.24]
LiuYuan 2016[438−1]	15	142	0.11 [0.06; 0.17]
QiYing 2019[535−3]	16	38	0.42 [0.26; 0.59]
QiYing 2019[535−4]	17	39	0.44 [0.28; 0.60]
QiYing 2019[535−5]	13	40	0.32 [0.19; 0.49]
QinDi 2016[544]	16	255	0.06 [0.04; 0.10]
QinLianYang 2021[545]	38	1697	0.02 [0.02; 0.03]
QinMeng 2015[547−2]	25	228	0.11 [0.07; 0.16]
RenQiZhi 2018[557−3]	8	151	0.05 [0.02; 0.10]
RenQiZhi 2018[557−6]	13	2500	0.01 [0.00; 0.01]
RenQiZhi 2018[557−7]	15	10000	0.00 [0.00; 0.00]
SongJie 2014[599]	17	350	0.05 [0.03; 0.08]
WangXiaoDong 2017[688]	69	2708	0.03 [0.02; 0.03]
YangTongTong 2018[813]	36	450	0.08 [0.06; 0.11]
YuHong 2016[848]	80	1184	0.07 [0.05; 0.08]
ZhangYanMing 2019[898]	15	236	0.06 [0.04; 0.10]
**Common effect model**		**24658**	**0.04 [0.03; 0.04]**
**Random effects model**			**0.14 [0.09; 0.20]**
Heterogeneity: $I^2 = 97\%$, $\tau^2 = 1.8840$, $p < 0.01$			
**month = 6**			
BaiYun 2018[8]	61	648	0.09 [0.07; 0.12]
CaiXiuZhi 2018[27−1]	34	150	0.23 [0.16; 0.30]
CaiXiuZhi 2018[27−3]	40	1045	0.04 [0.03; 0.05]
CuiLiangLiang 2017[123−1]	5	90	0.06 [0.02; 0.12]
CuiLiangLiang 2017[123−4]	1434	11000	0.13 [0.12; 0.14]
GaoZhiYong 2017[187−2]	10	46	0.22 [0.11; 0.36]
GuoLi 2019[206−26]	6	35	0.17 [0.07; 0.34]
GuoLi 2019[206−30]	6	38	0.16 [0.06; 0.31]
GuoLi 2019[206−45]	25	44	0.57 [0.41; 0.72]
GuoLi 2019[206−62]	47	339	0.14 [0.10; 0.18]
HuangYanHong 2019[259−10]	26	199	0.13 [0.09; 0.19]
HuangYanHong 2019[259−11]	32	456	0.07 [0.05; 0.10]

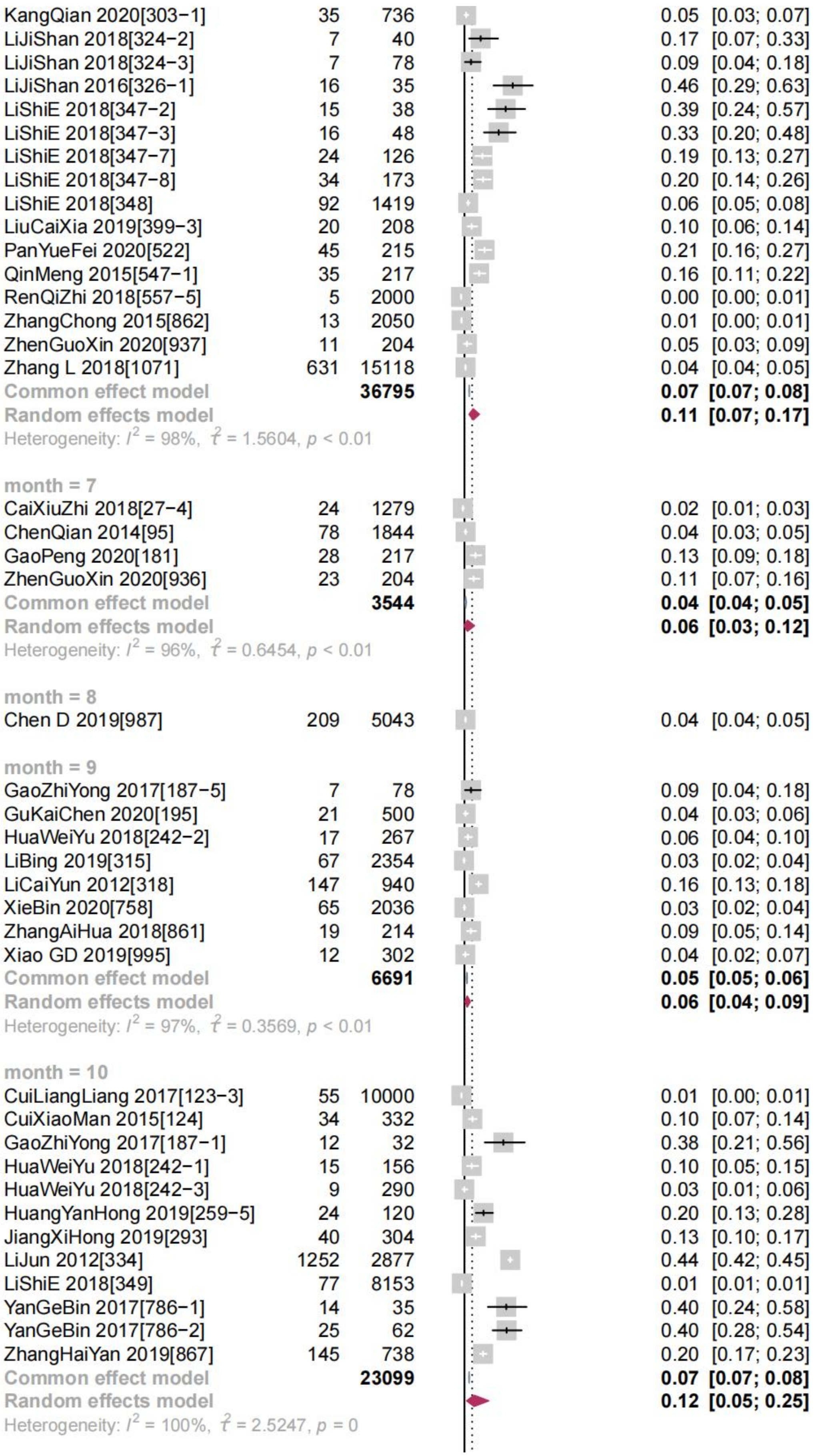

KangQian 2020[303−1] 35 736 0.05 [0.03; 0.07]
LiJiShan 2018[324−2] 7 40 0.17 [0.07; 0.33]
LiJiShan 2018[324−3] 7 78 0.09 [0.04; 0.18]
LiJiShan 2016[326−1] 16 35 0.46 [0.29; 0.63]
LiShiE 2018[347−2] 15 38 0.39 [0.24; 0.57]
LiShiE 2018[347−3] 16 48 0.33 [0.20; 0.48]
LiShiE 2018[347−7] 24 126 0.19 [0.13; 0.27]
LiShiE 2018[347−8] 34 173 0.20 [0.14; 0.26]
LiShiE 2018[348] 92 1419 0.06 [0.05; 0.08]
LiuCaiXia 2019[399−3] 20 208 0.10 [0.06; 0.14]
PanYueFei 2020[522] 45 215 0.21 [0.16; 0.27]
QinMeng 2015[547−1] 35 217 0.16 [0.11; 0.22]
RenQiZhi 2018[557−5] 5 2000 0.00 [0.00; 0.01]
ZhangChong 2015[862] 13 2050 0.01 [0.00; 0.01]
ZhenGuoXin 2020[937] 11 204 0.05 [0.03; 0.09]
Zhang L 2018[1071] 631 15118 0.04 [0.04; 0.05]
Common effect model 36795 0.07 [0.07; 0.08]
Random effects model 0.11 [0.07; 0.17]
Heterogeneity: I^2 = 98%, τ^2 = 1.5604, p < 0.01
month = 7
CaiXiuZhi 2018[27−4] 24 1279 0.02 [0.01; 0.03]
ChenQian 2014[95] 78 1844 0.04 [0.03; 0.05]
GaoPeng 2020[181] 28 217 0.13 [0.09; 0.18]
ZhenGuoXin 2020[936] 23 204 0.11 [0.07; 0.16]
Common effect model 3544 0.04 [0.04; 0.05]
Random effects model 0.06 [0.03; 0.12]
Heterogeneity: I^2 = 96%, τ^2 = 0.6454, p < 0.01
month = 8
Chen D 2019[987] 209 5043 0.04 [0.04; 0.05]
month = 9
GaoZhiYong 2017[187−5] 7 78 0.09 [0.04; 0.18]
GuKaiChen 2020[195] 21 500 0.04 [0.03; 0.06]
HuaWeiYu 2018[242−2] 17 267 0.06 [0.04; 0.10]
LiBing 2019[315] 67 2354 0.03 [0.02; 0.04]
LiCaiYun 2012[318] 147 940 0.16 [0.13; 0.18]
XieBin 2020[758] 65 2036 0.03 [0.02; 0.04]
ZhangAiHua 2018[861] 19 214 0.09 [0.05; 0.14]
Xiao GD 2019[995] 12 302 0.04 [0.02; 0.07]
Common effect model 6691 0.05 [0.05; 0.06]
Random effects model 0.06 [0.04; 0.09]
Heterogeneity: I^2 = 97%, τ^2 = 0.3569, p < 0.01
month = 10
CuiLiangLiang 2017[123−3] 55 10000 0.01 [0.00; 0.01]
CuiXiaoMan 2015[124] 34 332 0.10 [0.07; 0.14]
GaoZhiYong 2017[187−1] 12 32 0.38 [0.21; 0.56]
HuaWeiYu 2018[242−1] 15 156 0.10 [0.05; 0.15]
HuaWeiYu 2018[242−3] 9 290 0.03 [0.01; 0.06]
HuangYanHong 2019[259−5] 24 120 0.20 [0.13; 0.28]
JiangXiHong 2019[293] 40 304 0.13 [0.10; 0.17]
LiJun 2012[334] 1252 2877 0.44 [0.42; 0.45]
LiShiE 2018[349] 77 8153 0.01 [0.01; 0.01]
YanGeBin 2017[786−1] 14 35 0.40 [0.24; 0.58]
YanGeBin 2017[786−2] 25 62 0.40 [0.28; 0.54]
ZhangHaiYan 2019[867] 145 738 0.20 [0.17; 0.23]
Common effect model 23099 0.07 [0.07; 0.08]
Random effects model 0.12 [0.05; 0.25]
Heterogeneity: I^2 = 100%, τ^2 = 2.5247, p = 0

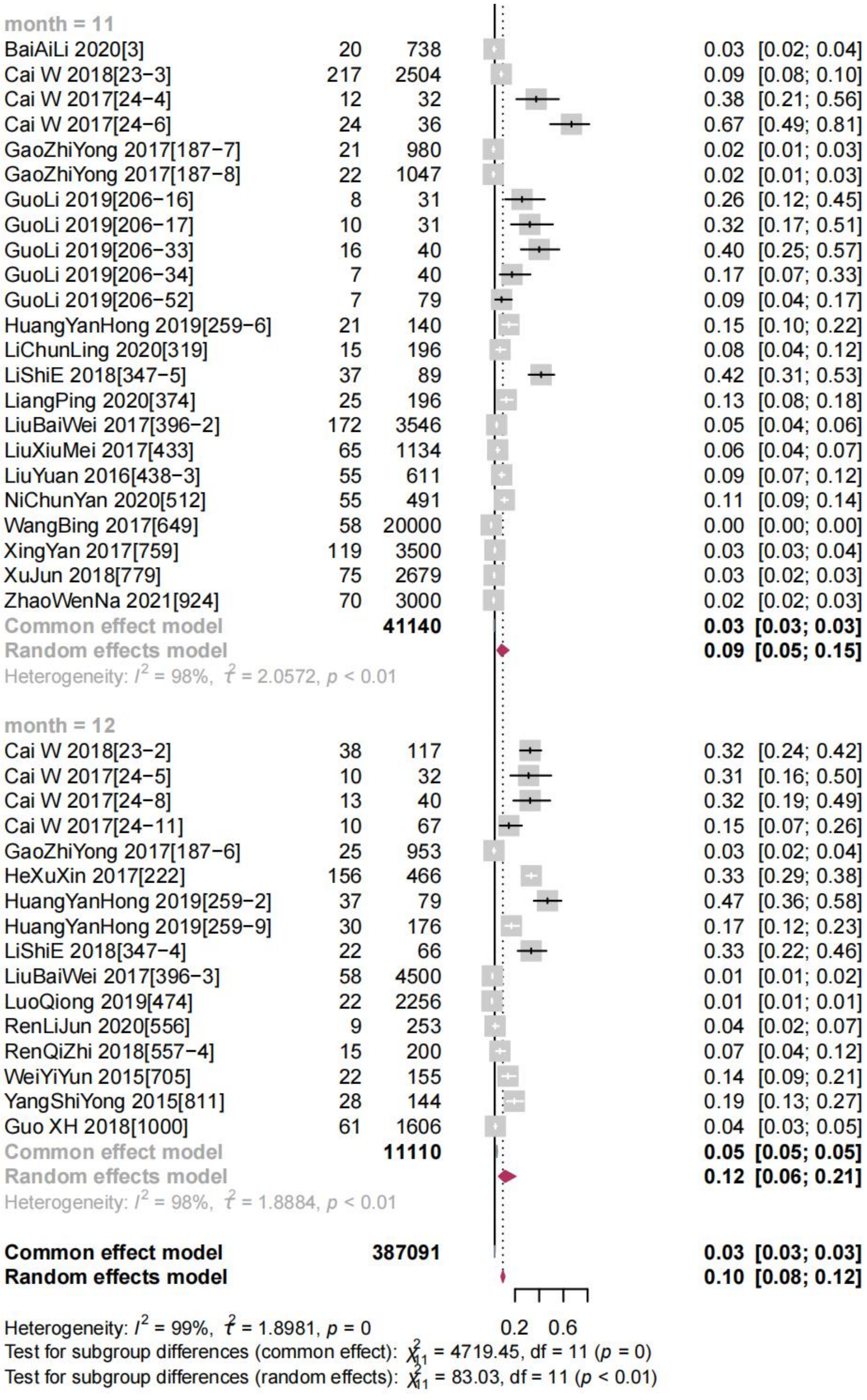
month = 11
BaiAiLi 2020[3] 20 738 0.03 [0.02; 0.04]
Cai W 2018[23−3] 217 2504 0.09 [0.08; 0.10]
Cai W 2017[24−4] 12 32 0.38 [0.21; 0.56]
Cai W 2017[24−6] 24 36 0.67 [0.49; 0.81]
GaoZhiYong 2017[187−7] 21 980 0.02 [0.01; 0.03]
GaoZhiYong 2017[187−8] 22 1047 0.02 [0.01; 0.03]
GuoLi 2019[206−16] 8 31 0.26 [0.12; 0.45]
GuoLi 2019[206−17] 10 31 0.32 [0.17; 0.51]
GuoLi 2019[206−33] 16 40 0.40 [0.25; 0.57]
GuoLi 2019[206−34] 7 40 0.17 [0.07; 0.33]
GuoLi 2019[206−52] 7 79 0.09 [0.04; 0.17]
HuangYanHong 2019[259−6] 21 140 0.15 [0.10; 0.22]
LiChunLing 2020[319] 15 196 0.08 [0.04; 0.12]
LiShiE 2018[347−5] 37 89 0.42 [0.31; 0.53]
LiangPing 2020[374] 25 196 0.13 [0.08; 0.18]
LiuBaiWei 2017[396−2] 172 3546 0.05 [0.04; 0.06]
LiuXiuMei 2017[433] 65 1134 0.06 [0.04; 0.07]
LiuYuan 2016[438−3] 55 611 0.09 [0.07; 0.12]
NiChunYan 2020[512] 55 491 0.11 [0.09; 0.14]
WangBing 2017[649] 58 20000 0.00 [0.00; 0.00]
XingYan 2017[759] 119 3500 0.03 [0.03; 0.04]
XuJun 2018[779] 75 2679 0.03 [0.02; 0.03]
ZhaoWenNa 2021[924] 70 3000 0.02 [0.02; 0.03]
Common effect model 41140 0.03 [0.03; 0.03]
Random effects model 0.09 [0.05; 0.15]
Heterogeneity: I^2 = 98%, τ^2 = 2.0572, p < 0.01
month = 12
Cai W 2018[23−2] 38 117 0.32 [0.24; 0.42]
Cai W 2017[24−5] 10 32 0.31 [0.16; 0.50]
Cai W 2017[24−8] 13 40 0.32 [0.19; 0.49]
Cai W 2017[24−11] 10 67 0.15 [0.07; 0.26]
GaoZhiYong 2017[187−6] 25 953 0.03 [0.02; 0.04]
HeXuXin 2017[222] 156 466 0.33 [0.29; 0.38]
HuangYanHong 2019[259−2] 37 79 0.47 [0.36; 0.58]
HuangYanHong 2019[259−9] 30 176 0.17 [0.12; 0.23]
LiShiE 2018[347−4] 22 66 0.33 [0.22; 0.46]
LiuBaiWei 2017[396−3] 58 4500 0.01 [0.01; 0.02]
LuoQiong 2019[474] 22 2256 0.01 [0.01; 0.01]
RenLiJun 2020[556] 9 253 0.04 [0.02; 0.07]
RenQiZhi 2018[557−4] 15 200 0.07 [0.04; 0.12]
WeiYiYun 2015[705] 22 155 0.14 [0.09; 0.21]
YangShiYong 2015[811] 28 144 0.19 [0.13; 0.27]
Guo XH 2018[1000] 61 1606 0.04 [0.03; 0.05]
Common effect model 11110 0.05 [0.05; 0.05]
Random effects model 0.12 [0.06; 0.21]
Heterogeneity: I^2 = 98%, τ^2 = 1.8884, p < 0.01
Common effect model 387091 0.03 [0.03; 0.03]
Random effects model 0.10 [0.08; 0.12]
0.2 0.6
Heterogeneity: I^2 = 99%, τ^2 = 1.8981, p = 0
Test for subgroup differences (common effect): χ^2_{11} = 4719.45, df = 11 (p = 0)
Test for subgroup differences (random effects): χ^2_{11} = 83.03, df = 11 (p < 0.01)

(b4)

Study South	Events	Total	Attack rate	95%−CI
month = 1				
BiHua 2015[11]	55	1262	0.04	[0.03; 0.06]
CaiWenFeng 2013[25]	141	16600	0.01	[0.01; 0.01]
ChaRiSheng 2014[47−4]	18	1111	0.02	[0.01; 0.03]
ChaRiSheng 2014[47−7]	74	1749	0.04	[0.03; 0.05]
ChaRiSheng 2014[47−12]	139	2786	0.05	[0.04; 0.06]
ChaRiSheng 2014[47−13]	39	3095	0.01	[0.01; 0.02]
ShenYuGang 2016[55]	233	1702	0.14	[0.12; 0.15]
ChenYiYi 2015[109]	282	38001	0.01	[0.01; 0.01]
DaiYingXue 2018[129−1]	14	378	0.04	[0.02; 0.06]
DuanRong 2017[152−13]	5	245	0.02	[0.01; 0.05]
DuanRong 2017[152−17]	5	495	0.01	[0.00; 0.02]
DuanRong 2017[152−20]	5	590	0.01	[0.00; 0.02]
DuanRong 2017[152−29]	5	976	0.01	[0.00; 0.01]
DuanRong 2017[152−30]	5	976	0.01	[0.00; 0.01]
DuanRong 2017[152−32]	5	1145	0.00	[0.00; 0.01]
GaoJunYing 2017[177]	12	1501	0.01	[0.00; 0.01]
GongLiQiang 2013[192]	139	2787	0.05	[0.04; 0.06]
GongShuiYing 2017[194]	93	990	0.09	[0.08; 0.11]
GuoMinJian 2017[208−10]	48	2200	0.02	[0.02; 0.03]
HuangLiQing 2020[254]	184	1823	0.10	[0.09; 0.12]
LeiYongLiang 2016[311]	46	984	0.05	[0.03; 0.06]
LiMeng 2020[338−5]	6	369	0.02	[0.01; 0.04]
LiMeng 2020[338−13]	17	1580	0.01	[0.01; 0.02]
LiShouJun 2016[350]	64	1023	0.06	[0.05; 0.08]
LiYueRong 2015[366]	18	952	0.02	[0.01; 0.03]
LiuShiKe 2016[422]	46	948	0.05	[0.04; 0.06]
LiuWenJun 2018[428−3]	38	528	0.07	[0.05; 0.10]
LuHua 2016[454]	14	213	0.07	[0.04; 0.11]
LuoTengXian 2018[475]	13	200	0.06	[0.04; 0.11]
PangZhiFeng 2017[524]	79	1696	0.05	[0.04; 0.06]
PangZhiFeng 2014[525]	20	548	0.04	[0.02; 0.06]
QianLiZhen 2020[537]	23	95	0.24	[0.16; 0.34]
QianZiYu 2012[539]	22	111	0.20	[0.13; 0.28]
RenFuLin 2013[555]	74	1751	0.04	[0.03; 0.05]
RuiFang 2018[566−11]	15	2905	0.01	[0.00; 0.01]
SongCanLei 2012[591]	18	385	0.05	[0.03; 0.07]
SongCanLei 2013[594]	26	162	0.16	[0.11; 0.23]
SongCanLei 2013[595]	11	71	0.15	[0.08; 0.26]
WangJun 2012[663]	17	400	0.04	[0.02; 0.07]
WangShuangYing 2016[676]	16	119	0.13	[0.08; 0.21]
WuXiaYan 2015[730]	92	2046	0.04	[0.04; 0.05]
XiaoSongJian 2017[745]	51	416	0.12	[0.09; 0.16]
XieYuanQi 2016[756]	34	207	0.16	[0.12; 0.22]
XuJianRong 2018[767]	92	2046	0.04	[0.04; 0.05]
XuHao 2016[775]	28	2385	0.01	[0.01; 0.02]
YanChaoYang 2017[792]	90	2168	0.04	[0.03; 0.05]
YangSenPing 2018[810]	30	305	0.10	[0.07; 0.14]
YangYouQing 2016[819]	48	2363	0.02	[0.02; 0.03]
YingLiHong 2016[840]	48	3000	0.02	[0.01; 0.02]
YuKuangMing 2015[851]	16	57	0.28	[0.17; 0.42]
ZhangLing 2016[879]	132	735	0.18	[0.15; 0.21]
ZhangXiaoYi 2015[891]	21	57	0.37	[0.24; 0.51]

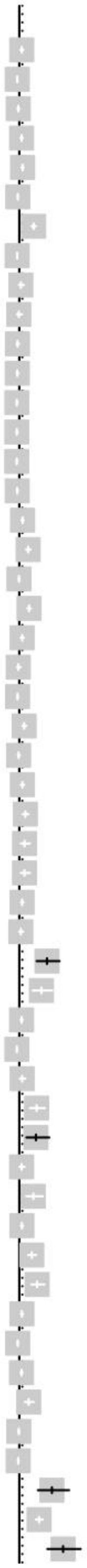

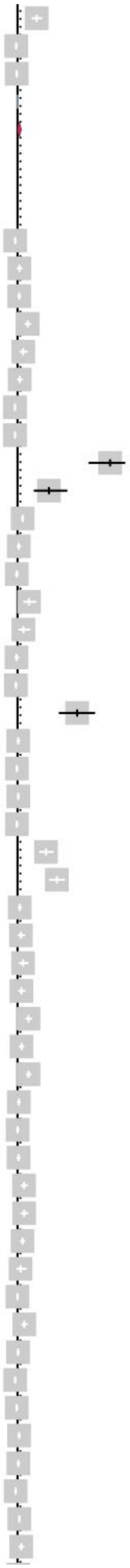

ZhangZhen 2015[903]	76	414	0.18 [0.15; 0.22]
ZhouXiaoHong 2017[961]	47	3464	0.01 [0.01; 0.02]
ZhuChengMing 2017[967]	78	4454	0.02 [0.01; 0.02]
**Common effect model**		**119569**	**0.02 [0.02; 0.03]**
**Random effects model**			**0.04 [0.03; 0.05]**
Heterogeneity: $I^2 = 98\%$, $\tau^2 = 1.4341$, $p = 0$			
**month = 2**			
BiHua 2014[10]	95	18930	0.01 [0.00; 0.01]
Ming−Wei Cai 2021[17−1]	25	628	0.04 [0.03; 0.06]
Cai SJ 2018[20]	69	1781	0.04 [0.03; 0.05]
CaoXiaoPing 2018[33]	113	1037	0.11 [0.09; 0.13]
ZengLei 2018[44]	28	388	0.07 [0.05; 0.10]
ShenYiPing 2018[52]	26	612	0.04 [0.03; 0.06]
ChenAQun 2015[58]	64	19153	0.00 [0.00; 0.00]
ChenAQun 2016[59]	64	18924	0.00 [0.00; 0.00]
ChenGuoCui 2011[69]	27	34	0.79 [0.62; 0.91]
ChenHeJuan 2018[73]	14	49	0.29 [0.17; 0.43]
ChenXingHong 2012[102]	478	7113	0.07 [0.06; 0.07]
ChenXingFu 2018[103]	43	1240	0.03 [0.03; 0.05]
DaiYingXue 2018[129−3]	18	1008	0.02 [0.01; 0.03]
DongShengCao 2017[146]	24	200	0.12 [0.08; 0.17]
DuanRong 2017[152−5]	12	165	0.07 [0.04; 0.12]
DuanRong 2017[152−16]	7	441	0.02 [0.01; 0.03]
DuanRong 2017[152−33]	13	1216	0.01 [0.01; 0.02]
FengZhi 2020[161]	26	50	0.52 [0.37; 0.66]
GaoShuPing 2019[183]	51	1698	0.03 [0.02; 0.04]
GuShiPing 2009[196]	42	2199	0.02 [0.01; 0.03]
HuHongAn 2015[233]	105	3453	0.03 [0.02; 0.04]
Lei Ji 2011[271]	42	2210	0.02 [0.01; 0.03]
LiJianSen 2015[328]	70	268	0.26 [0.21; 0.32]
LiShiCong 2019[345]	91	259	0.35 [0.29; 0.41]
LiShiCong 2018[346]	64	1487	0.04 [0.03; 0.05]
LiXiuFang 2018[359]	20	378	0.05 [0.03; 0.08]
LiuWenJun 2018[428−1]	18	255	0.07 [0.04; 0.11]
LiuWenJun 2018[428−2]	22	388	0.06 [0.04; 0.08]
LiuWenJun 2018[428−9]	105	907	0.12 [0.10; 0.14]
LiuWenJun 2018[428−11]	55	955	0.06 [0.04; 0.07]
LiuWenJun 2018[428−17]	180	1554	0.12 [0.10; 0.13]
LiuWenJun 2018[428−19]	68	1926	0.04 [0.03; 0.04]
LiuWenJun 2018[428−21]	55	2174	0.03 [0.02; 0.03]
LuoLe 2017[472]	39	1192	0.03 [0.02; 0.04]
MaTao 2018[487]	46	587	0.08 [0.06; 0.10]
MengJian 2018[495]	37	465	0.08 [0.06; 0.11]
RuiFang 2018[566−9]	78	1184	0.07 [0.05; 0.08]
SongCanLei 2013[592−2]	8	161	0.05 [0.02; 0.10]
SongJianQiang 2015[598]	259	10942	0.02 [0.02; 0.03]
SongYuFang 2019[600]	63	783	0.08 [0.06; 0.10]
SunYunLan 2018[624]	63	2212	0.03 [0.02; 0.04]
SunZhou 2016[625]	230	45075	0.01 [0.00; 0.01]
TangYuHuan 2017[630]	44	2494	0.02 [0.01; 0.02]
TangGuoJie 2019[631]	84	2250	0.04 [0.03; 0.05]
WangJinSheng 2019[645]	56	1800	0.03 [0.02; 0.04]
WangJinSheng 2016[646]	99	11594	0.01 [0.01; 0.01]
WangJie 2015[659]	451	11467	0.04 [0.04; 0.04]
WangMin 2018[670−3]	37	672	0.06 [0.04; 0.08]

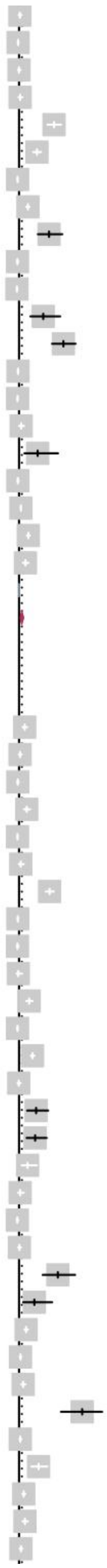

WangMin 2018[670−4]	27	861	0.03 [0.02; 0.05]
WangMin 2018[670−6]	47	2869	0.02 [0.01; 0.02]
WenYa 2018[710]	16	668	0.02 [0.01; 0.04]
WuGuoFu 2018[717]	17	506	0.03 [0.02; 0.05]
WuYang 2018[733]	85	265	0.32 [0.26; 0.38]
WuYang 2018[735]	91	516	0.18 [0.14; 0.21]
WuZhiSheng 2014[741]	176	14612	0.01 [0.01; 0.01]
XiaYingPin 2018[743]	177	1777	0.10 [0.09; 0.11]
XuFeng 2012[774]	22	79	0.28 [0.18; 0.39]
XuJinFeng 2016[778−1]	10	933	0.01 [0.01; 0.02]
XuJinFeng 2016[778−2]	16	2500	0.01 [0.00; 0.01]
YangZhiWen 2013[820]	11	48	0.23 [0.12; 0.37]
ZhangHuiLing 2018[871]	40	100	0.40 [0.30; 0.50]
ZhangMeiMei 2013[880]	126	8154	0.02 [0.01; 0.02]
ZhouXiaoMin 2017[962]	47	3917	0.01 [0.01; 0.02]
ZhuChunXiu 2019[968]	17	401	0.04 [0.02; 0.07]
Huang J 2013[1006−3]	6	33	0.18 [0.07; 0.35]
Li J 2018[1015]	19	1327	0.01 [0.01; 0.02]
Shang X 2017[1041]	924	23068	0.04 [0.04; 0.04]
LiuQin 2011[1088]	257	2497	0.10 [0.09; 0.12]
XuHaoBin 2015[1091]	39	496	0.08 [0.06; 0.11]
**Common effect model**		**251585**	**0.02 [0.02; 0.02]**
**Random effects model**			**0.05 [0.03; 0.06]**
Heterogeneity: $I^2 = 99\%$, $\tau^2 = 1.7595$, $p = 0$			
**month = 3**			
Ming−Wei Cai 2021[17−2]	50	693	0.07 [0.05; 0.09]
CaiWenFeng 2014[26]	27	816	0.03 [0.02; 0.05]
ChaRiSheng 2014[47−5]	16	1168	0.01 [0.01; 0.02]
ShenYuGang 2016[54]	43	480	0.09 [0.07; 0.12]
ChenJianMei 2017[79]	110	9736	0.01 [0.01; 0.01]
ChenJingFang 2018[84−2]	14	360	0.04 [0.02; 0.06]
ChenMinHong 2017[92−2]	186	657	0.28 [0.25; 0.32]
ChenMinHong 2017[92−3]	60	4286	0.01 [0.01; 0.02]
ChenXiaoFeng 2015[100]	7	648	0.01 [0.00; 0.02]
ChenXin 2020[101]	3	169	0.02 [0.00; 0.05]
ChenYan 2019[104]	59	536	0.11 [0.08; 0.14]
ChenYiXiong 2018[108]	21	1861	0.01 [0.01; 0.02]
ChenZhiQiong 2017[115]	255	1843	0.14 [0.12; 0.15]
DaiYingXue 2018[129−2]	12	547	0.02 [0.01; 0.04]
DuanRong 2017[152−1]	13	77	0.17 [0.09; 0.27]
DuanRong 2017[152−2]	13	81	0.16 [0.09; 0.26]
DuanRong 2017[152−4]	9	93	0.10 [0.05; 0.18]
DuanRong 2017[152−12]	8	238	0.03 [0.01; 0.07]
DuanRong 2017[152−27]	9	918	0.01 [0.00; 0.02]
FuXiaoFei 2012[171]	20	728	0.03 [0.02; 0.04]
GaoHuiJuan 2015[176]	18	51	0.35 [0.22; 0.50]
GuoMinJian 2017[208−2]	6	39	0.15 [0.06; 0.31]
HuangChunLi 2015[246]	108	1283	0.08 [0.07; 0.10]
HuangGe 2013[251]	92	2527	0.04 [0.03; 0.04]
JiJinHua 2018[265]	41	694	0.06 [0.04; 0.08]
JiLei 2020[270]	19	34	0.56 [0.38; 0.73]
JiangYingCi 2015[284]	66	1904	0.03 [0.03; 0.04]
JiangChen 2019[285]	26	137	0.19 [0.13; 0.27]
LinQiFeng 2018[388]	96	1494	0.06 [0.05; 0.08]
LiuWenJun 2018[428−4]	42	565	0.07 [0.05; 0.10]
LiuWenJun 2018[428−12]	49	1213	0.04 [0.03; 0.05]

LiuWenJun 2018[428−13]	193	1285	0.15 [0.13; 0.17]
LiuWenJun 2018[428−16]	54	1467	0.04 [0.03; 0.05]
LiuWenJun 2018[428−23]	70	3608	0.02 [0.02; 0.02]
MoYuJie 2018[505]	19	1117	0.02 [0.01; 0.03]
QiYanQiu 2018[532]	27	570	0.05 [0.03; 0.07]
QiXiaoQi 2019[534]	185	2418	0.08 [0.07; 0.09]
RuiFang 2018[566−4]	5	382	0.01 [0.00; 0.03]
RuiFang 2018[566−8]	8	1059	0.01 [0.00; 0.01]
RuiFang 2018[566−10]	10	2084	0.00 [0.00; 0.01]
ShuaiHuiQun 2012[589]	14	569	0.02 [0.01; 0.04]
SunMingHua 2017[614]	36	496	0.07 [0.05; 0.10]
WangMin 2018[670−5]	32	2843	0.01 [0.01; 0.02]
WangXiaoQin 2015[689]	11	142	0.08 [0.04; 0.13]
WuWenQian 2018[729]	36	2399	0.02 [0.01; 0.02]
WuYang 2018[734]	26	2179	0.01 [0.01; 0.02]
WuZhenYu 2012[738]	19	57	0.33 [0.21; 0.47]
XiaGuangHui 2019[742]	106	2300	0.05 [0.04; 0.06]
XieCaiWen 2017[749]	12	58	0.21 [0.11; 0.33]
YangJiXing 2019[801]	30	808	0.04 [0.03; 0.05]
YangJing 2018[804]	111	1718	0.06 [0.05; 0.08]
YaoJing 2017[824]	179	885	0.20 [0.18; 0.23]
YeXianMing 2017[832]	94	1156	0.08 [0.07; 0.10]
YinHongMei 2014[839]	37	511	0.07 [0.05; 0.10]
YuanJun 2014[854]	652	115469	0.01 [0.01; 0.01]
ZhangHengQiu 2014[869]	18	250	0.07 [0.04; 0.11]
ZhangZhengDong 2017[904]	25	466	0.05 [0.04; 0.08]
ZhangZhiZhong 2020[906]	263	17752	0.01 [0.01; 0.02]
ZhaoXueCheng 2016[929]	149	1570	0.09 [0.08; 0.11]
ZhaoYuLi 2019[933]	119	1784	0.07 [0.06; 0.08]
ZhuWeiWei 2018[976]	91	9702	0.01 [0.01; 0.01]
Huang Z 2020[1008]	68	786	0.09 [0.07; 0.11]
Y Li 2021[1018]	143	346	0.41 [0.36; 0.47]
Lu Y 2020[1029]	157	785	0.20 [0.17; 0.23]
LiYing 2018[1097]	53	1288	0.04 [0.03; 0.05]
**Common effect model**		**216185**	**0.02 [0.02; 0.02]**
**Random effects model**			**0.05 [0.04; 0.07]**
Heterogeneity: $I^2 = 99\%$, $\tau^2 = 1.4734$, $p = 0$			
**month = 4**			
Ming−Wei Cai 2021[17−3]	27	816	0.03 [0.02; 0.05]
ChaRiSheng 2016[46]	22	453	0.05 [0.03; 0.07]
ChaRiSheng 2014[47−10]	168	2002	0.08 [0.07; 0.10]
ShenJiChuan 2011[50]	312	1736	0.18 [0.16; 0.20]
ChuXiuJuan 2015[120]	17	147	0.12 [0.07; 0.18]
DuanRong 2017[152−8]	9	199	0.05 [0.02; 0.08]
DuanRong 2017[152−26]	24	802	0.03 [0.02; 0.04]
FengZhi 2018[162]	111	2020	0.05 [0.05; 0.07]
GanXiangYang 2014[172]	74	2200	0.03 [0.03; 0.04]
HuangBinBin 2020[245]	41	2530	0.02 [0.01; 0.02]
HuangShiTeng 2021[256]	61	1644	0.04 [0.03; 0.05]
LiMeng 2020[338−14]	85	1814	0.05 [0.04; 0.06]
LiShiCong 2020[344]	181	3622	0.05 [0.04; 0.06]
LiangRiCheng 2017[376]	27	2518	0.01 [0.01; 0.02]
LiuHaoHui 2020[410−8]	145	618	0.23 [0.20; 0.27]
LiuJingJing 2018[413]	33	111	0.30 [0.21; 0.39]
LiuWenJun 2018[428−22]	52	2332	0.02 [0.02; 0.03]

LiuYi 2013[436]	9	35	0.26	[0.12; 0.43]
LiuYing 2019[437]	14	335	0.04	[0.02; 0.07]
NiChaoRong 2019[510]	125	3622	0.03	[0.03; 0.04]
PuPeiLong 2017[529]	98	1126	0.09	[0.07; 0.11]
Wu−Yang Shi 2020[577−1]	35	441	0.08	[0.06; 0.11]
ShiChao 2013[579]	462	2024	0.23	[0.21; 0.25]
TanDongMei 2012[627]	87	2086	0.04	[0.03; 0.05]
WangMan 2017[668]	85	540	0.16	[0.13; 0.19]
WenYingMing 2019[711]	15	35	0.43	[0.26; 0.61]
ZhangDongSheng 2013[864]	97	6614	0.01	[0.01; 0.02]
ZhangQingHe 2020[909]	109	538	0.20	[0.17; 0.24]
ZhaoMeiLing 2019[918−1]	8	842	0.01	[0.00; 0.02]
ZhouJianHong 2013[954]	15	636	0.02	[0.01; 0.04]
ZongJun 2017[980]	45	78	0.58	[0.46; 0.69]
Huang J 2013[1006−4]	6	38	0.16	[0.06; 0.31]
Huang J 2013[1006−5]	14	103	0.14	[0.08; 0.22]
Qi L 2018[1034]	101	555	0.18	[0.15; 0.22]
Wang X 2016[1055]	51	377	0.14	[0.10; 0.17]
Zhou X 2019[1080]	75	1612	0.05	[0.04; 0.06]
**Common effect model**		**47201**	**0.06**	**[0.06; 0.06]**
**Random effects model**			**0.07**	**[0.05; 0.10]**
Heterogeneity: $I^2 = 99\%$, $\tau^2 = 1.3257$, $p = 0$				
**month = 5**				
Cai SX 2017[21]	17	1276	0.01	[0.01; 0.02]
Cao RR 2020[30−1]	41	2853	0.01	[0.01; 0.02]
ChaRiSheng 2014[47−3]	43	594	0.07	[0.05; 0.10]
ChenQuan 2021[96]	84	821	0.10	[0.08; 0.13]
DuanRong 2017[152−18]	9	538	0.02	[0.01; 0.03]
DuanRong 2017[152−19]	7	565	0.01	[0.00; 0.03]
GuiGuoPing 2018[200]	79	1204	0.07	[0.05; 0.08]
JiHong 2015[264]	78	3194	0.02	[0.02; 0.03]
JiangYiMei 2017[294]	15	3050	0.00	[0.00; 0.01]
LiJie 2019[329]	106	2868	0.04	[0.03; 0.04]
LiuDan 2019[403]	90	951	0.09	[0.08; 0.12]
LiuDongSheng 2019[404]	80	3720	0.02	[0.02; 0.03]
LiuWenJun 2018[428−14]	46	1314	0.04	[0.03; 0.05]
LiuWenJun 2018[428−15]	17	1328	0.01	[0.01; 0.02]
MengXiangJie 2012[499]	16	34	0.47	[0.30; 0.65]
RenFuLin 2013[554]	43	594	0.07	[0.05; 0.10]
RuiFang 2018[566−7]	54	908	0.06	[0.04; 0.08]
SongCanLei 2020[590]	44	812	0.05	[0.04; 0.07]
WangMeiHuan 2020[669]	12	433	0.03	[0.01; 0.05]
YueYong 2017[859]	12	150	0.08	[0.04; 0.14]
ZhaoMengJiao 2018[919]	87	263	0.33	[0.27; 0.39]
HuangJiaCheng 2021[944]	48	5682	0.01	[0.01; 0.01]
ShenJingQiang 2020[993]	15	37	0.41	[0.25; 0.58]
Liu Y 2015[1025]	99	2049	0.05	[0.04; 0.06]
**Common effect model**		**35238**	**0.03**	**[0.03; 0.03]**
**Random effects model**			**0.04**	**[0.03; 0.07]**
Heterogeneity: $I^2 = 98\%$, $\tau^2 = 1.6100$, $p < 0.01$				
**month = 6**				
Cai MW 2018[18]	34	223	0.15	[0.11; 0.21]
DuanRong 2017[152−14]	8	249	0.03	[0.01; 0.06]
GuoMinJian 2017[208−7]	7	43	0.16	[0.07; 0.31]

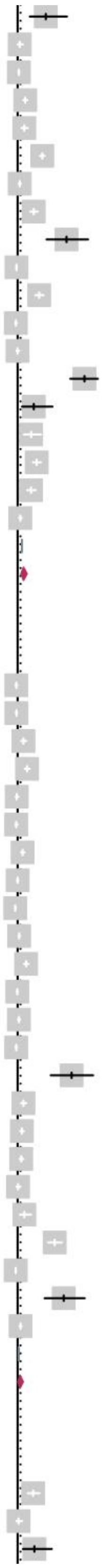

HeHanZhen 2014[224]	76	578	0.13 [0.11; 0.16]
LiuHaoHui 2020[410−9]	17	2191	0.01 [0.00; 0.01]
MaTao 2015[486]	84	901	0.09 [0.08; 0.11]
MoGuiQiong 2016[504]	26	843	0.03 [0.02; 0.04]
RenYuHua 2016[562]	110	1916	0.06 [0.05; 0.07]
ZhongWenLong 2012[948]	10	69	0.14 [0.07; 0.25]
Zhang TL 2017[1075]	20	327	0.06 [0.04; 0.09]
**Common effect model**		**7340**	**0.05 [0.05; 0.06]**
**Random effects model**			**0.06 [0.04; 0.11]**
Heterogeneity: I^2 = 95%, τ^2 = 0.8735, p < 0.01			
**month = 7**			
CaoShen 2019[32]	62	6966	0.01 [0.01; 0.01]
DengXingChao 2019[133]	439	42403	0.01 [0.01; 0.01]
GuoMinJian 2017[208−9]	16	1488	0.01 [0.01; 0.02]
GuoShuiLian 2014[209]	16	76	0.21 [0.13; 0.32]
HuangZhongXue 2013[263]	22	92	0.24 [0.16; 0.34]
LuWeiWei 2016[462]	406	3808	0.11 [0.10; 0.12]
TangXuLi 2013[633]	23	116	0.20 [0.13; 0.28]
LiXiaoJing 2021[992]	7	251	0.03 [0.01; 0.06]
**Common effect model**		**55200**	**0.02 [0.02; 0.02]**
**Random effects model**			**0.05 [0.02; 0.13]**
Heterogeneity: I^2 = 100%, τ^2 = 2.1504, p < 0.01			
**month = 8**			
LongQiZhi 2016[442]	101	1707	0.06 [0.05; 0.07]
MiaoGuoZhong 2018[507]	25	350	0.07 [0.05; 0.10]
TangYuXin 2014[634]	97	994	0.10 [0.08; 0.12]
WuHongXing 2016[718]	63	726	0.09 [0.07; 0.11]
WuShuiXin 2012[727]	25	1376	0.02 [0.01; 0.03]
ZhaoYiNan 2018[932]	67	3000	0.02 [0.02; 0.03]
Zhou X 2012[1081]	108	5769	0.02 [0.02; 0.02]
**Common effect model**		**13922**	**0.03 [0.03; 0.04]**
**Random effects model**			**0.04 [0.03; 0.07]**
Heterogeneity: I^2 = 98%, τ^2 = 0.5114, p < 0.01			
**month = 9**			
ChaRiSheng 2014[47−8]	47	1858	0.03 [0.02; 0.03]
DaiBenNa 2020[127]	117	3119	0.04 [0.03; 0.04]
LaiShiMing 2014[308]	105	7113	0.01 [0.01; 0.02]
LiQun 2010[340]	258	14269	0.02 [0.02; 0.02]
LiaoKeChang 2018[381]	41	161	0.25 [0.19; 0.33]
LiuHaoHui 2020[410−4]	14	166	0.08 [0.05; 0.14]
LiuHaoHui 2020[410−5]	9	202	0.04 [0.02; 0.08]
LiuKaiQian 2011[415]	634	14439	0.04 [0.04; 0.05]
MaMengMeng 2018[484]	223	30711	0.01 [0.01; 0.01]
OuSheXiang 2019[515]	18	603	0.03 [0.02; 0.05]
PengXiaoXue 2015[528]	37	849	0.04 [0.03; 0.06]
QiuHaiYan 2013[551]	76	5965	0.01 [0.01; 0.02]
SunLiMei 2012[612]	108	5854	0.02 [0.02; 0.02]
TaoLiYan 2020[636]	47	1857	0.03 [0.02; 0.03]
WangDaHu 2020[652]	182	33479	0.01 [0.00; 0.01]
WangKaiLiang 2019[665]	16	446	0.04 [0.02; 0.06]
WeiGuiYing 2019[706]	29	257	0.11 [0.08; 0.16]
Jin−Bo Ye 2018[830−11]	101	1784	0.06 [0.05; 0.07]
Jin−Bo Ye 2018[830−13]	60	2572	0.02 [0.02; 0.03]

Jin−Bo Ye 2018[830−14]	132	2584	0.05	[0.04; 0.06]
YeShuJun 2019[831]	104	399	0.26	[0.22; 0.31]
ZhouAiHua 2017[950]	80	650	0.12	[0.10; 0.15]
ZhouLiHong 2016[956]	13	37	0.35	[0.20; 0.53]
ZhouXiaoHong 2015[958]	107	7113	0.02	[0.01; 0.02]
Y Li 2013[1019]	396	14387	0.03	[0.02; 0.03]
**Common effect model**		**150874**	**0.02**	**[0.02; 0.02]**
**Random effects model**			**0.04**	**[0.03; 0.06]**
Heterogeneity: $I^2 = 99\%$, $\tau^2 = 1.2298$, $p = 0$				
**month = 10**				
ZengFengMei 2020[41]	25	255	0.10	[0.06; 0.14]
ChaRiSheng 2014[47−9]	17	1910	0.01	[0.01; 0.01]
ShenYiPing 2013[53]	65	1900	0.03	[0.03; 0.04]
DuYueHe 2019[148]	29	1250	0.02	[0.02; 0.03]
DuanRong 2017[152−6]	11	179	0.06	[0.03; 0.11]
DuanRong 2017[152−7]	10	182	0.05	[0.03; 0.10]
DuanRong 2017[152−10]	36	227	0.16	[0.11; 0.21]
DuanRong 2017[152−11]	5	229	0.02	[0.01; 0.05]
DuanRong 2017[152−23]	8	706	0.01	[0.00; 0.02]
DuanRong 2017[152−31]	68	1036	0.07	[0.05; 0.08]
GaoHaiMing 2014[174]	19	187	0.10	[0.06; 0.15]
GuYiFu 2020[197]	45	1903	0.02	[0.02; 0.03]
GuoMinJian 2017[208−1]	8	31	0.26	[0.12; 0.45]
HouYuYuan 2014[231]	63	984	0.06	[0.05; 0.08]
JiangXianChen 2014[283−1]	16	1485	0.01	[0.01; 0.02]
JiangXianChen 2014[283−2]	23	1877	0.01	[0.01; 0.02]
LiMeng 2020[338−1]	7	74	0.09	[0.04; 0.19]
LiMeng 2020[338−8]	11	659	0.02	[0.01; 0.03]
LiMeng 2020[338−15]	6	1863	0.00	[0.00; 0.01]
LiuHaoHui 2020[410−1]	6	35	0.17	[0.07; 0.34]
LiuHaoHui 2020[410−2]	28	53	0.53	[0.39; 0.67]
LiuHaoHui 2020[410−6]	16	300	0.05	[0.03; 0.09]
LiuHaoHui 2020[410−7]	12	561	0.02	[0.01; 0.04]
LiuWenJun 2018[428−6]	36	700	0.05	[0.04; 0.07]
LiuWenJun 2018[428−8]	16	904	0.02	[0.01; 0.03]
LiuWenJun 2018[428−10]	31	951	0.03	[0.02; 0.05]
QinYanMin 2010[549]	97	1031	0.09	[0.08; 0.11]
RuiFang 2018[566−6]	16	670	0.02	[0.01; 0.04]
SunQin 2019[616]	32	128	0.25	[0.18; 0.33]
WangHua 2014[657]	28	497	0.06	[0.04; 0.08]
XuShiMin 2013[771]	37	116	0.32	[0.24; 0.41]
YangCaiBin 2018[796]	42	2159	0.02	[0.01; 0.03]
YangRongXing 2019[808]	15	334	0.04	[0.03; 0.07]
YangYiLong 2014[818]	121	18597	0.01	[0.01; 0.01]
YaoJianXiang 2016[822]	59	2992	0.02	[0.02; 0.03]
Jin−Bo Ye 2018[830−9]	40	1125	0.04	[0.03; 0.05]
ZhangLi 2015[875−1]	33	474	0.07	[0.05; 0.10]
ZhangLi 2015[875−2]	72	656	0.11	[0.09; 0.14]
ZhangNing 2018[882]	31	340	0.09	[0.06; 0.13]
ZhangGuangMing 2018[907]	14	149	0.09	[0.05; 0.15]
ZhaoJun 2014[916]	26	287	0.09	[0.06; 0.13]
ZhouXiaoTao 2011[959]	96	1045	0.09	[0.08; 0.11]
Xu H 2013[1059−2]	65	1912	0.03	[0.03; 0.04]
Yang Z 2011[1068]	427	71534	0.01	[0.01; 0.01]
ZouYong 2011[1103]	39	2672	0.01	[0.01; 0.02]
**Common effect model**		**127159**	**0.01**	**[0.01; 0.02]**
**Random effects model**			**0.04**	**[0.03; 0.06]**
Heterogeneity: $I^2 = 98\%$, $\tau^2 = 1.3822$, $p = 0$				

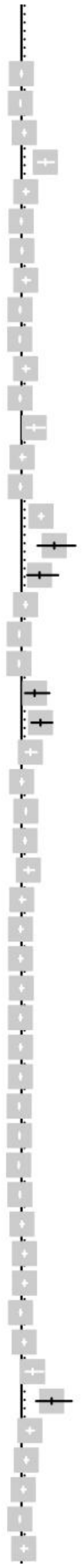

**month = 11**			
CaiJian 2013[14−1]	53	2219	0.02 [0.02; 0.03]
CaiJian 2013[14−2]	257	16056	0.02 [0.01; 0.02]
Ming−Wei Cai 2021[17−4]	56	1202	0.05 [0.04; 0.06]
ChaRiSheng 2014[47−1]	36	170	0.21 [0.15; 0.28]
ChaRiSheng 2014[47−2]	29	498	0.06 [0.04; 0.08]
ChaRiSheng 2014[47−6]	31	1422	0.02 [0.01; 0.03]
ChaRiSheng 2014[47−11]	61	2202	0.03 [0.02; 0.04]
ChenCaiRong 2020[62]	16	275	0.06 [0.03; 0.09]
ChenJian 2016[78]	20	1551	0.01 [0.01; 0.02]
ChenJian 2017[80]	69	6323	0.01 [0.01; 0.01]
ChenJingFang 2018[84−1]	18	316	0.06 [0.03; 0.09]
DaiYingXue 2018[129−4]	14	1053	0.01 [0.01; 0.02]
DuanRong 2017[152−3]	11	89	0.12 [0.06; 0.21]
DuanRong 2017[152−15]	8	271	0.03 [0.01; 0.06]
DuanRong 2017[152−28]	14	927	0.02 [0.01; 0.03]
FuJianGuang 2013[163]	276	1546	0.18 [0.16; 0.20]
GuoMinJian 2017[208−3]	11	39	0.28 [0.15; 0.45]
GuoMinJian 2017[208−6]	7	42	0.17 [0.07; 0.31]
HuangGuo 2015[252]	69	1220	0.06 [0.04; 0.07]
HuangGuo 2015[253]	87	13856	0.01 [0.01; 0.01]
HuangSiYue 2020[257]	90	19800	0.00 [0.00; 0.01]
JiLei 2018[272−1]	7	55	0.13 [0.05; 0.24]
JiLei 2018[272−2]	16	92	0.17 [0.10; 0.27]
JiLei 2018[272−3]	18	191	0.09 [0.06; 0.14]
JiangLie 2014[291]	15	590	0.03 [0.01; 0.04]
KuangHaoCheng 2016[307]	667	11300	0.06 [0.05; 0.06]
LiBo 2013[316]	120	2303	0.05 [0.04; 0.06]
LiMeng 2020[338−2]	16	204	0.08 [0.05; 0.12]
LiMeng 2020[338−3]	7	260	0.03 [0.01; 0.05]
LiMeng 2020[338−4]	6	358	0.02 [0.01; 0.04]
LiMeng 2020[338−6]	7	416	0.02 [0.01; 0.03]
LiMeng 2020[338−7]	11	476	0.02 [0.01; 0.04]
LiMeng 2020[338−9]	12	749	0.02 [0.01; 0.03]
LiMeng 2020[338−10]	13	754	0.02 [0.01; 0.03]
LiMeng 2020[338−11]	19	1168	0.02 [0.01; 0.03]
LiMeng 2020[338−12]	9	1462	0.01 [0.00; 0.01]
LiMeng 2020[338−16]	17	2026	0.01 [0.00; 0.01]
LiMeng 2020[338−17]	5	2026	0.00 [0.00; 0.01]
LiYiLan 2014[365]	228	18930	0.01 [0.01; 0.01]
LiaoChan 2021[380]	19	675	0.03 [0.02; 0.04]
LinQin 2015[390]	34	713	0.05 [0.03; 0.07]
LinQingShuang 2016[391]	29	603	0.05 [0.03; 0.07]
LiuHongLian 2018[411]	19	1119	0.02 [0.01; 0.03]
LiuQingLian 2019[420]	37	817	0.05 [0.03; 0.06]
LiuTian 2019[423]	10	89	0.11 [0.06; 0.20]
LiuTian 2017[424−2]	11	42	0.26 [0.14; 0.42]
LiuTian 2017[424−7]	35	387	0.09 [0.06; 0.12]
LuJianYong 2015[456]	60	973	0.06 [0.05; 0.08]
LuCaiFang 2020[458]	19	499	0.04 [0.02; 0.06]
PanYiFeng 2017[521]	82	7396	0.01 [0.01; 0.01]
RuiFang 2018[566−3]	11	263	0.04 [0.02; 0.07]

SongCanLei 2017[593]	36	888	0.04 [0.03; 0.06]
SunLiYan 2020[613]	11	51	0.22 [0.11; 0.35]
WangJun 2021[664]	20	4743	0.00 [0.00; 0.01]
WangMin 2018[670−1]	19	180	0.11 [0.06; 0.16]
WangMin 2018[670−2]	11	599	0.02 [0.01; 0.03]
WangTieJun 2020[678]	15	345	0.04 [0.02; 0.07]
WuJingWen 2020[719]	34	1153	0.03 [0.02; 0.04]
WuXiaoMin 2021[732]	159	1378	0.12 [0.10; 0.13]
WuYiLing 2017[736]	31	1311	0.02 [0.02; 0.03]
XiaZhongFa 2012[744]	209	1616	0.13 [0.11; 0.15]
XiaoDaYong 2018[746]	47	154	0.31 [0.23; 0.38]
XuanLingFeng 2014[784]	17	9523	0.00 [0.00; 0.00]
Jin−Bo Ye 2018[830−1]	25	136	0.18 [0.12; 0.26]
Jin−Bo Ye 2018[830−5]	90	255	0.35 [0.29; 0.42]
Jin−Bo Ye 2018[830−7]	81	295	0.27 [0.22; 0.33]
YeXiaoLing 2014[833]	27	264	0.10 [0.07; 0.15]
YuZhuXian 2013[849]	13	357	0.04 [0.02; 0.06]
ZhanYueWang 2019[860]	38	2311	0.02 [0.01; 0.02]
ZhangPan 2020[883]	16	244	0.07 [0.04; 0.10]
ZhangGuoBao 2020[908]	13	105	0.12 [0.07; 0.20]
ZhaoMeiLing 2019[918−2]	39	3305	0.01 [0.01; 0.02]
ZhaoTengLong 2015[921]	25	564	0.04 [0.03; 0.06]
ZhouXiaoHong 2010[960]	37	295	0.13 [0.09; 0.17]
HuangKaiXiong 2021[989]	36	660	0.05 [0.04; 0.07]
WuLin 2021[1035]	17	194	0.09 [0.05; 0.14]
Ruan F 2013[1039]	63	22200	0.00 [0.00; 0.00]
Tsang TK 2018[1050]	159	643	0.25 [0.21; 0.28]
Xu H 2013[1059−1]	207	1630	0.13 [0.11; 0.14]
Xue C 2014[1060]	278	1995	0.14 [0.12; 0.16]
Zhang XF 2020[1076]	27	450	0.06 [0.04; 0.09]
**Common effect model**		**185857**	**0.02 [0.02; 0.03]**
**Random effects model**			**0.04 [0.03; 0.05]**
Heterogeneity: $I^2 = 99\%$, $\tau^2 = 1.5899$, $p = 0$			
**month = 12**			
CenYongZhuang 2014[39]	32	106	0.30 [0.22; 0.40]
ChenBinBin 2017[60−1]	54	392	0.14 [0.11; 0.18]
ChenBinBin 2017[60−2]	161	2122	0.08 [0.06; 0.09]
ChenCan 2018[63]	24	61	0.39 [0.27; 0.53]
ChenChun 2014[64]	107	1242	0.09 [0.07; 0.10]
ChenMinHong 2017[92−1]	21	236	0.09 [0.06; 0.13]
DuanRong 2017[152−9]	14	215	0.07 [0.04; 0.11]
DuanRong 2017[152−21]	11	699	0.02 [0.01; 0.03]
DuanRong 2017[152−22]	7	701	0.01 [0.00; 0.02]
DuanRong 2017[152−24]	7	733	0.01 [0.00; 0.02]
DuanRong 2017[152−25]	5	753	0.01 [0.00; 0.02]
DuanRong 2017[152−34]	11	1420	0.01 [0.00; 0.01]
GuoMinJian 2017[208−4]	10	39	0.26 [0.13; 0.42]
GuoMinJian 2017[208−5]	17	39	0.44 [0.28; 0.60]
GuoMinJian 2017[208−8]	7	53	0.13 [0.05; 0.25]
JiRuPing 2020[274]	119	2030	0.06 [0.05; 0.07]
LiDaiBo 2018[320]	18	56	0.32 [0.20; 0.46]
LinJian 2019[384]	77	2476	0.03 [0.02; 0.04]
LinYanYan 2018[393]	76	3288	0.02 [0.02; 0.03]
LiuBo 2015[398]	104	1883	0.06 [0.05; 0.07]
LiuGuoHong 2014[406]	109	636	0.17 [0.14; 0.20]

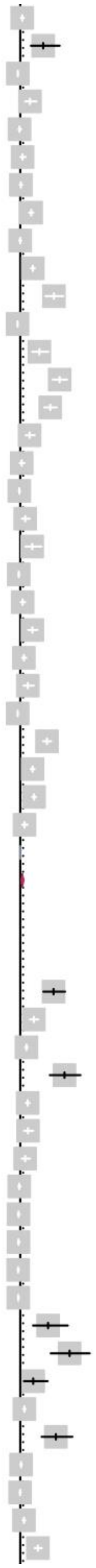

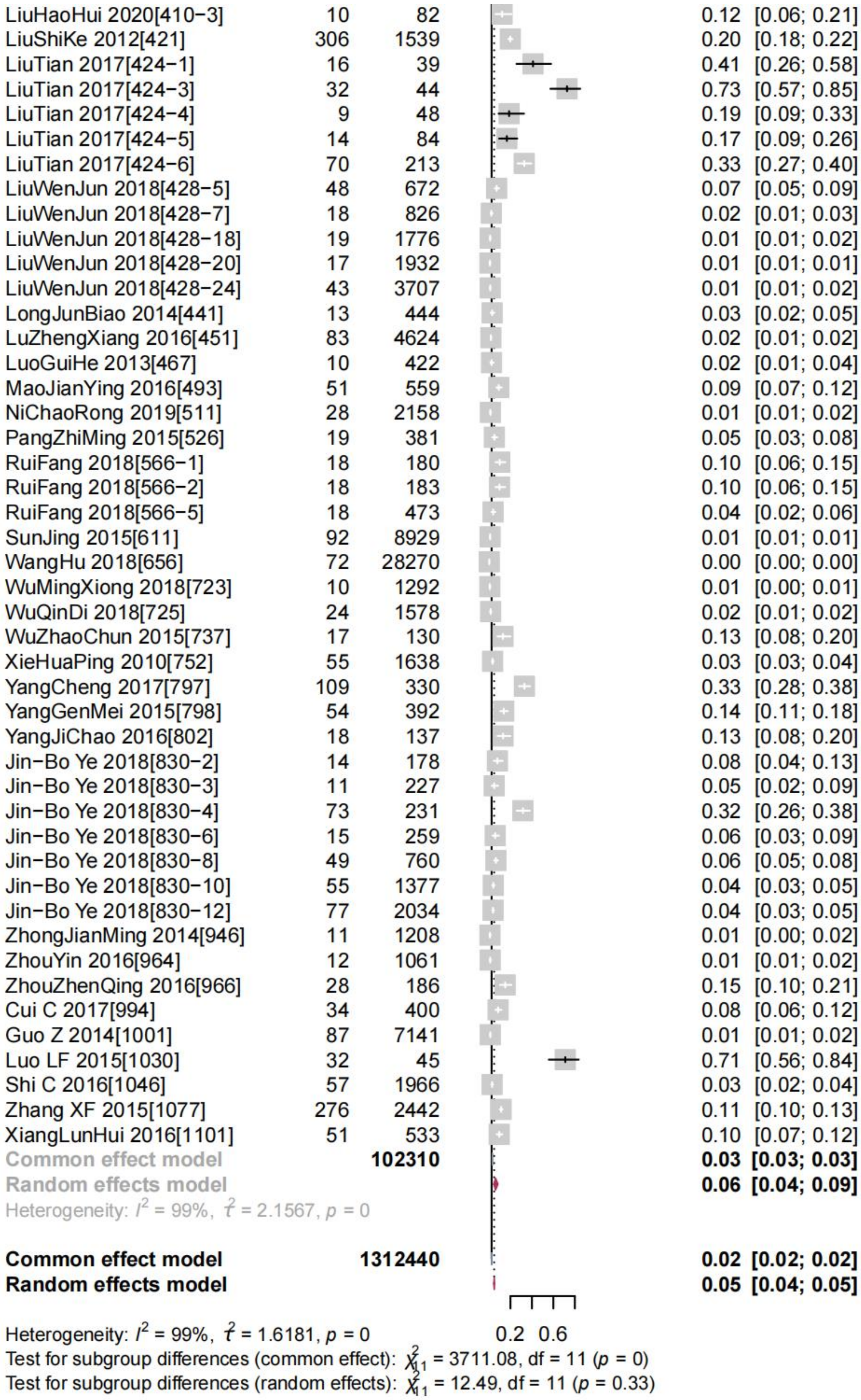
LiuHaoHui 2020[410−3] 10 82 0.12 [0.06; 0.21]
LiuShiKe 2012[421] 306 1539 0.20 [0.18; 0.22]
LiuTian 2017[424−1] 16 39 0.41 [0.26; 0.58]
LiuTian 2017[424−3] 32 44 0.73 [0.57; 0.85]
LiuTian 2017[424−4] 9 48 0.19 [0.09; 0.33]
LiuTian 2017[424−5] 14 84 0.17 [0.09; 0.26]
LiuTian 2017[424−6] 70 213 0.33 [0.27; 0.40]
LiuWenJun 2018[428−5] 48 672 0.07 [0.05; 0.09]
LiuWenJun 2018[428−7] 18 826 0.02 [0.01; 0.03]
LiuWenJun 2018[428−18] 19 1776 0.01 [0.01; 0.02]
LiuWenJun 2018[428−20] 17 1932 0.01 [0.01; 0.01]
LiuWenJun 2018[428−24] 43 3707 0.01 [0.01; 0.02]
LongJunBiao 2014[441] 13 444 0.03 [0.02; 0.05]
LuZhengXiang 2016[451] 83 4624 0.02 [0.01; 0.02]
LuoGuiHe 2013[467] 10 422 0.02 [0.01; 0.04]
MaoJianYing 2016[493] 51 559 0.09 [0.07; 0.12]
NiChaoRong 2019[511] 28 2158 0.01 [0.01; 0.02]
PangZhiMing 2015[526] 19 381 0.05 [0.03; 0.08]
RuiFang 2018[566−1] 18 180 0.10 [0.06; 0.15]
RuiFang 2018[566−2] 18 183 0.10 [0.06; 0.15]
RuiFang 2018[566−5] 18 473 0.04 [0.02; 0.06]
SunJing 2015[611] 92 8929 0.01 [0.01; 0.01]
WangHu 2018[656] 72 28270 0.00 [0.00; 0.00]
WuMingXiong 2018[723] 10 1292 0.01 [0.00; 0.01]
WuQinDi 2018[725] 24 1578 0.02 [0.01; 0.02]
WuZhaoChun 2015[737] 17 130 0.13 [0.08; 0.20]
XieHuaPing 2010[752] 55 1638 0.03 [0.03; 0.04]
YangCheng 2017[797] 109 330 0.33 [0.28; 0.38]
YangGenMei 2015[798] 54 392 0.14 [0.11; 0.18]
YangJiChao 2016[802] 18 137 0.13 [0.08; 0.20]
Jin−Bo Ye 2018[830−2] 14 178 0.08 [0.04; 0.13]
Jin−Bo Ye 2018[830−3] 11 227 0.05 [0.02; 0.09]
Jin−Bo Ye 2018[830−4] 73 231 0.32 [0.26; 0.38]
Jin−Bo Ye 2018[830−6] 15 259 0.06 [0.03; 0.09]
Jin−Bo Ye 2018[830−8] 49 760 0.06 [0.05; 0.08]
Jin−Bo Ye 2018[830−10] 55 1377 0.04 [0.03; 0.05]
Jin−Bo Ye 2018[830−12] 77 2034 0.04 [0.03; 0.05]
ZhongJianMing 2014[946] 11 1208 0.01 [0.00; 0.02]
ZhouYin 2016[964] 12 1061 0.01 [0.01; 0.02]
ZhouZhenQing 2016[966] 28 186 0.15 [0.10; 0.21]
Cui C 2017[994] 34 400 0.08 [0.06; 0.12]
Guo Z 2014[1001] 87 7141 0.01 [0.01; 0.02]
Luo LF 2015[1030] 32 45 0.71 [0.56; 0.84]
Shi C 2016[1046] 57 1966 0.03 [0.02; 0.04]
Zhang XF 2015[1077] 276 2442 0.11 [0.10; 0.13]
XiangLunHui 2016[1101] 51 533 0.10 [0.07; 0.12]
Common effect model 102310 0.03 [0.03; 0.03]
Random effects model 0.06 [0.04; 0.09]
Heterogeneity: I^2 = 99%, τ^2 = 2.1567, p = 0
Common effect model 1312440 0.02 [0.02; 0.02]
Random effects model 0.05 [0.04; 0.05]
0.2 0.6
Heterogeneity: I^2 = 99%, τ^2 = 1.6181, p = 0
Test for subgroup differences (common effect): χ^2_{11} = 3711.08, df = 11 (p = 0)
Test for subgroup differences (random effects): χ^2_{11} = 12.49, df = 11 (p = 0.33)

(b5)

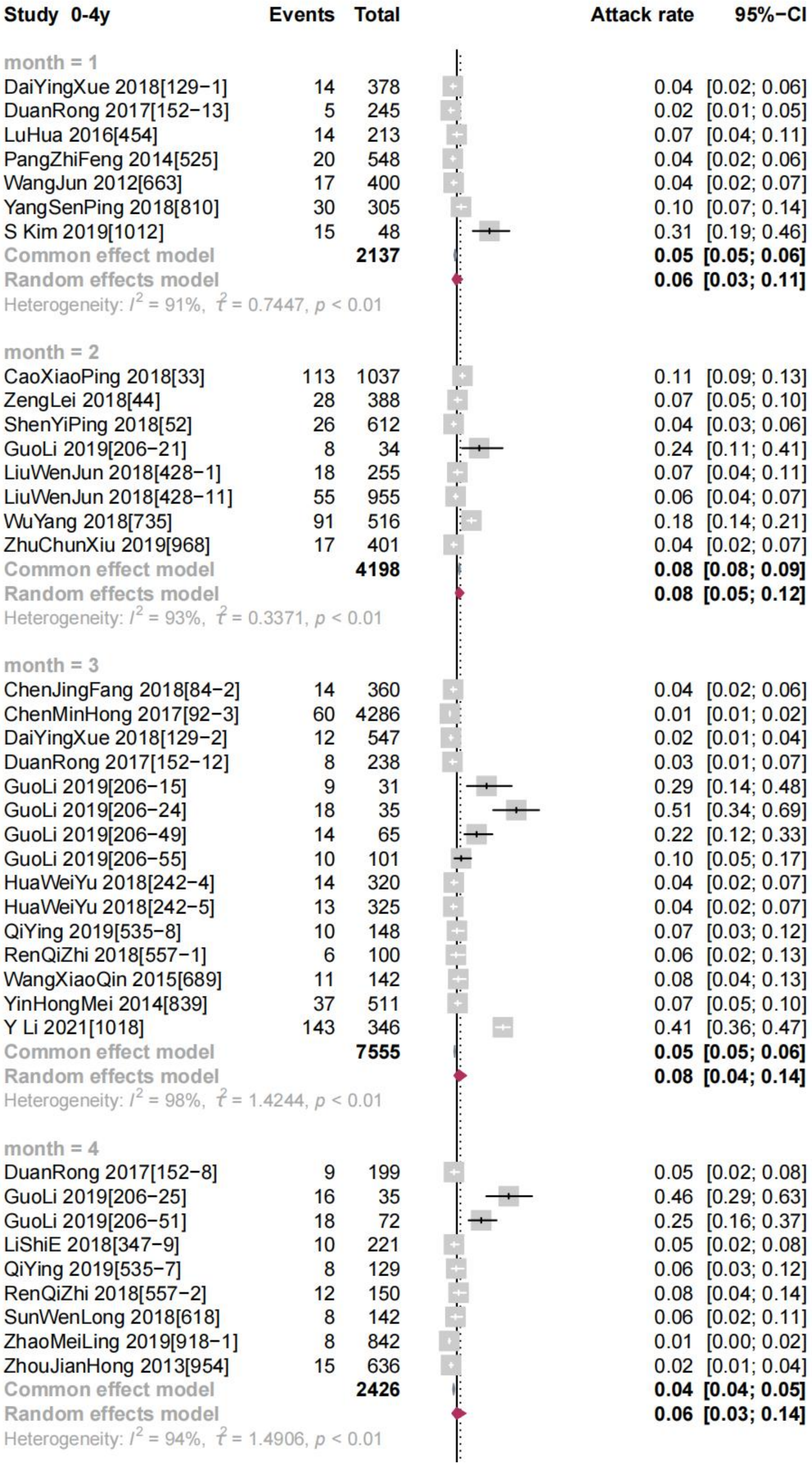
Study 0-4y Events Total Attack rate 95%−CI
month = 1
DaiYingXue 2018[129−1] 14 378 0.04 [0.02; 0.06]
DuanRong 2017[152−13] 5 245 0.02 [0.01; 0.05]
LuHua 2016[454] 14 213 0.07 [0.04; 0.11]
PangZhiFeng 2014[525] 20 548 0.04 [0.02; 0.06]
WangJun 2012[663] 17 400 0.04 [0.02; 0.07]
YangSenPing 2018[810] 30 305 0.10 [0.07; 0.14]
S Kim 2019[1012] 15 48 0.31 [0.19; 0.46]
Common effect model 2137 0.05 [0.05; 0.06]
Random effects model 0.06 [0.03; 0.11]
Heterogeneity: I^2 = 91%, τ^2 = 0.7447, p < 0.01
month = 2
CaoXiaoPing 2018[33] 113 1037 0.11 [0.09; 0.13]
ZengLei 2018[44] 28 388 0.07 [0.05; 0.10]
ShenYiPing 2018[52] 26 612 0.04 [0.03; 0.06]
GuoLi 2019[206−21] 8 34 0.24 [0.11; 0.41]
LiuWenJun 2018[428−1] 18 255 0.07 [0.04; 0.11]
LiuWenJun 2018[428−11] 55 955 0.06 [0.04; 0.07]
WuYang 2018[735] 91 516 0.18 [0.14; 0.21]
ZhuChunXiu 2019[968] 17 401 0.04 [0.02; 0.07]
Common effect model 4198 0.08 [0.08; 0.09]
Random effects model 0.08 [0.05; 0.12]
Heterogeneity: I^2 = 93%, τ^2 = 0.3371, p < 0.01
month = 3
ChenJingFang 2018[84−2] 14 360 0.04 [0.02; 0.06]
ChenMinHong 2017[92−3] 60 4286 0.01 [0.01; 0.02]
DaiYingXue 2018[129−2] 12 547 0.02 [0.01; 0.04]
DuanRong 2017[152−12] 8 238 0.03 [0.01; 0.07]
GuoLi 2019[206−15] 9 31 0.29 [0.14; 0.48]
GuoLi 2019[206−24] 18 35 0.51 [0.34; 0.69]
GuoLi 2019[206−49] 14 65 0.22 [0.12; 0.33]
GuoLi 2019[206−55] 10 101 0.10 [0.05; 0.17]
HuaWeiYu 2018[242−4] 14 320 0.04 [0.02; 0.07]
HuaWeiYu 2018[242−5] 13 325 0.04 [0.02; 0.07]
QiYing 2019[535−8] 10 148 0.07 [0.03; 0.12]
RenQiZhi 2018[557−1] 6 100 0.06 [0.02; 0.13]
WangXiaoQin 2015[689] 11 142 0.08 [0.04; 0.13]
YinHongMei 2014[839] 37 511 0.07 [0.05; 0.10]
Y Li 2021[1018] 143 346 0.41 [0.36; 0.47]
Common effect model 7555 0.05 [0.05; 0.06]
Random effects model 0.08 [0.04; 0.14]
Heterogeneity: I^2 = 98%, τ^2 = 1.4244, p < 0.01
month = 4
DuanRong 2017[152−8] 9 199 0.05 [0.02; 0.08]
GuoLi 2019[206−25] 16 35 0.46 [0.29; 0.63]
GuoLi 2019[206−51] 18 72 0.25 [0.16; 0.37]
LiShiE 2018[347−9] 10 221 0.05 [0.02; 0.08]
QiYing 2019[535−7] 8 129 0.06 [0.03; 0.12]
RenQiZhi 2018[557−2] 12 150 0.08 [0.04; 0.14]
SunWenLong 2018[618] 8 142 0.06 [0.02; 0.11]
ZhaoMeiLing 2019[918−1] 8 842 0.01 [0.00; 0.02]
ZhouJianHong 2013[954] 15 636 0.02 [0.01; 0.04]
Common effect model 2426 0.04 [0.04; 0.05]
Random effects model 0.06 [0.03; 0.14]
Heterogeneity: I^2 = 94%, τ^2 = 1.4906, p < 0.01

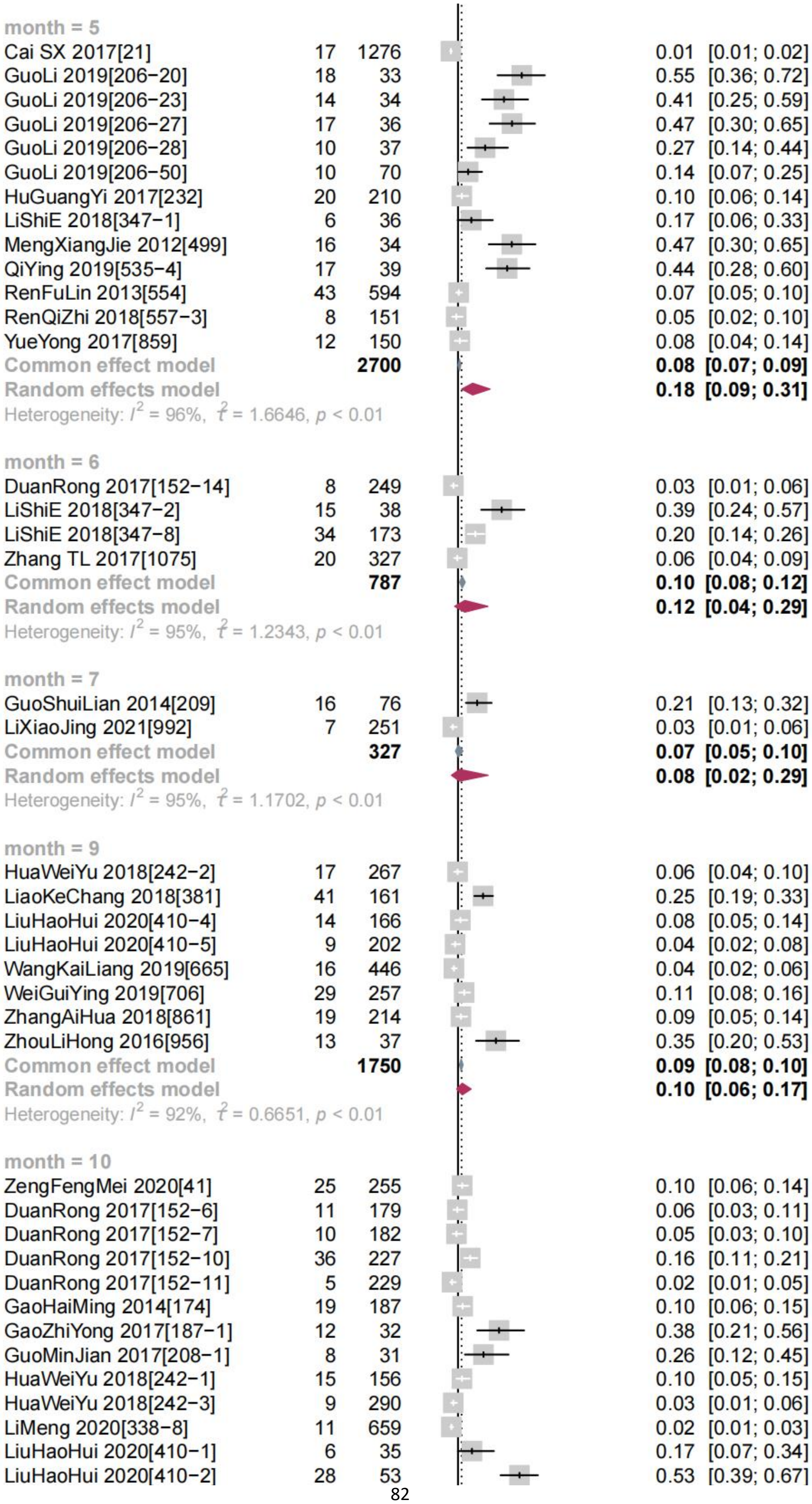
month = 5
Cai SX 2017[21] 17 1276 0.01 [0.01; 0.02]
GuoLi 2019[206−20] 18 33 0.55 [0.36; 0.72]
GuoLi 2019[206−23] 14 34 0.41 [0.25; 0.59]
GuoLi 2019[206−27] 17 36 0.47 [0.30; 0.65]
GuoLi 2019[206−28] 10 37 0.27 [0.14; 0.44]
GuoLi 2019[206−50] 10 70 0.14 [0.07; 0.25]
HuGuangYi 2017[232] 20 210 0.10 [0.06; 0.14]
LiShiE 2018[347−1] 6 36 0.17 [0.06; 0.33]
MengXiangJie 2012[499] 16 34 0.47 [0.30; 0.65]
QiYing 2019[535−4] 17 39 0.44 [0.28; 0.60]
RenFuLin 2013[554] 43 594 0.07 [0.05; 0.10]
RenQiZhi 2018[557−3] 8 151 0.05 [0.02; 0.10]
YueYong 2017[859] 12 150 0.08 [0.04; 0.14]
Common effect model 2700 0.08 [0.07; 0.09]
Random effects model 0.18 [0.09; 0.31]
Heterogeneity: I^2 = 96%, τ^2 = 1.6646, p < 0.01
month = 6
DuanRong 2017[152−14] 8 249 0.03 [0.01; 0.06]
LiShiE 2018[347−2] 15 38 0.39 [0.24; 0.57]
LiShiE 2018[347−8] 34 173 0.20 [0.14; 0.26]
Zhang TL 2017[1075] 20 327 0.06 [0.04; 0.09]
Common effect model 787 0.10 [0.08; 0.12]
Random effects model 0.12 [0.04; 0.29]
Heterogeneity: I^2 = 95%, τ^2 = 1.2343, p < 0.01
month = 7
GuoShuiLian 2014[209] 16 76 0.21 [0.13; 0.32]
LiXiaoJing 2021[992] 7 251 0.03 [0.01; 0.06]
Common effect model 327 0.07 [0.05; 0.10]
Random effects model 0.08 [0.02; 0.29]
Heterogeneity: I^2 = 95%, τ^2 = 1.1702, p < 0.01
month = 9
HuaWeiYu 2018[242−2] 17 267 0.06 [0.04; 0.10]
LiaoKeChang 2018[381] 41 161 0.25 [0.19; 0.33]
LiuHaoHui 2020[410−4] 14 166 0.08 [0.05; 0.14]
LiuHaoHui 2020[410−5] 9 202 0.04 [0.02; 0.08]
WangKaiLiang 2019[665] 16 446 0.04 [0.02; 0.06]
WeiGuiYing 2019[706] 29 257 0.11 [0.08; 0.16]
ZhangAiHua 2018[861] 19 214 0.09 [0.05; 0.14]
ZhouLiHong 2016[956] 13 37 0.35 [0.20; 0.53]
Common effect model 1750 0.09 [0.08; 0.10]
Random effects model 0.10 [0.06; 0.17]
Heterogeneity: I^2 = 92%, τ^2 = 0.6651, p < 0.01
month = 10
ZengFengMei 2020[41] 25 255 0.10 [0.06; 0.14]
DuanRong 2017[152−6] 11 179 0.06 [0.03; 0.11]
DuanRong 2017[152−7] 10 182 0.05 [0.03; 0.10]
DuanRong 2017[152−10] 36 227 0.16 [0.11; 0.21]
DuanRong 2017[152−11] 5 229 0.02 [0.01; 0.05]
GaoHaiMing 2014[174] 19 187 0.10 [0.06; 0.15]
GaoZhiYong 2017[187−1] 12 32 0.38 [0.21; 0.56]
GuoMinJian 2017[208−1] 8 31 0.26 [0.12; 0.45]
HuaWeiYu 2018[242−1] 15 156 0.10 [0.05; 0.15]
HuaWeiYu 2018[242−3] 9 290 0.03 [0.01; 0.06]
LiMeng 2020[338−8] 11 659 0.02 [0.01; 0.03]
LiuHaoHui 2020[410−1] 6 35 0.17 [0.07; 0.34]
LiuHaoHui 2020[410−2] 28 53 0.53 [0.39; 0.67]

LiuHaoHui 2020[410−6]	16	300	0.05 [0.03; 0.09]
LiuHaoHui 2020[410−7]	12	561	0.02 [0.01; 0.04]
LiuWenJun 2018[428−6]	36	700	0.05 [0.04; 0.07]
LiuWenJun 2018[428−8]	16	904	0.02 [0.01; 0.03]
WangHua 2014[657]	28	497	0.06 [0.04; 0.08]
YanGeBin 2017[786−1]	14	35	0.40 [0.24; 0.58]
YanGeBin 2017[786−2]	25	62	0.40 [0.28; 0.54]
YangRongXing 2019[808]	15	334	0.04 [0.03; 0.07]
ZhangGuangMing 2018[907]	14	149	0.09 [0.05; 0.15]
**Common effect model**		**6057**	**0.06 [0.06; 0.07]**
**Random effects model**			**0.09 [0.05; 0.14]**
Heterogeneity: I^2 = 94%, τ^2 = 1.4038, p < 0.01			
**month = 11**			
Cai W 2017[24−4]	12	32	0.38 [0.21; 0.56]
ChenJingFang 2018[84−1]	18	316	0.06 [0.03; 0.09]
DuanRong 2017[152−3]	11	89	0.12 [0.06; 0.21]
DuanRong 2017[152−15]	8	271	0.03 [0.01; 0.06]
GuoLi 2019[206−16]	8	31	0.26 [0.12; 0.45]
GuoLi 2019[206−17]	10	31	0.32 [0.17; 0.51]
GuoLi 2019[206−33]	16	40	0.40 [0.25; 0.57]
JiLei 2018[272−2]	16	92	0.17 [0.10; 0.27]
LiChunLing 2020[319]	15	196	0.08 [0.04; 0.12]
LiMeng 2020[338−2]	16	204	0.08 [0.05; 0.12]
LiMeng 2020[338−3]	7	260	0.03 [0.01; 0.05]
LiMeng 2020[338−4]	6	358	0.02 [0.01; 0.04]
LiMeng 2020[338−6]	7	416	0.02 [0.01; 0.03]
LiMeng 2020[338−7]	11	476	0.02 [0.01; 0.04]
LiShiE 2018[347−5]	37	89	0.42 [0.31; 0.53]
LiangPing 2020[374]	25	196	0.13 [0.08; 0.18]
LinQin 2015[390]	34	713	0.05 [0.03; 0.07]
LiuTian 2017[424−2]	11	42	0.26 [0.14; 0.42]
LuCaiFang 2020[458]	19	499	0.04 [0.02; 0.06]
WangMin 2018[670−1]	19	180	0.11 [0.06; 0.16]
WangMin 2018[670−2]	11	599	0.02 [0.01; 0.03]
ZhangGuoBao 2020[908]	13	105	0.12 [0.07; 0.20]
**Common effect model**		**5235**	**0.06 [0.06; 0.07]**
**Random effects model**			**0.09 [0.06; 0.14]**
Heterogeneity: I^2 = 94%, τ^2 = 1.4018, p < 0.01			
**month = 12**			
Cai W 2017[24−5]	10	32	0.31 [0.16; 0.50]
DuanRong 2017[152−9]	14	215	0.07 [0.04; 0.11]
DuanRong 2017[152−22]	7	701	0.01 [0.00; 0.02]
GuoMinJian 2017[208−5]	17	39	0.44 [0.28; 0.60]
LiShiE 2018[347−4]	22	66	0.33 [0.22; 0.46]
LiuWenJun 2018[428−5]	48	672	0.07 [0.05; 0.09]
RenLiJun 2020[556]	9	253	0.04 [0.02; 0.07]
RenQiZhi 2018[557−4]	15	200	0.07 [0.04; 0.12]
YangCheng 2017[797]	109	330	0.33 [0.28; 0.38]
YangJiChao 2016[802]	18	137	0.13 [0.08; 0.20]
**Common effect model**		**2645**	**0.10 [0.09; 0.11]**
**Random effects model**			**0.12 [0.06; 0.23]**
Heterogeneity: I^2 = 96%, τ^2 = 1.6453, p < 0.01			
**Common effect model**		**35817**	**0.07 [0.06; 0.07]**
**Random effects model**			**0.09 [0.08; 0.11]**

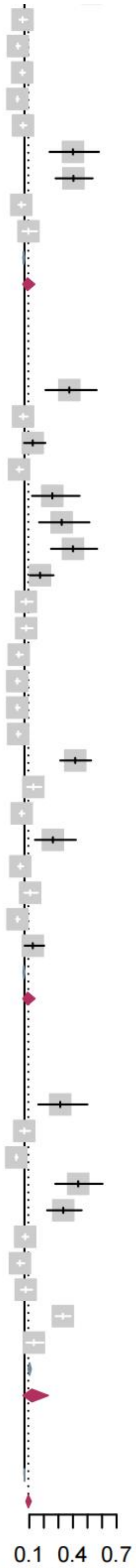

Heterogeneity: I^2 = 95%, τ^2 = 1.3867, p = 0

Test for subgroup differences (common effect): χ^2_{10} = 167.39, df = 10 (p < 0.01)

Test for subgroup differences (random effects): χ^2_{10} = 8.13, df = 10 (p = 0.62)

(b6)

Study 5-17y	Events	Total	Attack rate	95%−CI
**month = 1**				
DuanRong 2017[152−17]	5	495	0.01	[0.00; 0.02]
DuanRong 2017[152−20]	5	590	0.01	[0.00; 0.02]
DuanRong 2017[152−29]	5	976	0.01	[0.00; 0.01]
DuanRong 2017[152−30]	5	976	0.01	[0.00; 0.01]
DuanRong 2017[152−32]	5	1145	0.00	[0.00; 0.01]
GaoJunYing 2017[177]	12	1501	0.01	[0.00; 0.01]
GaoZhiYong 2017[187−4]	16	63	0.25	[0.15; 0.38]
GongLiQiang 2013[192]	139	2787	0.05	[0.04; 0.06]
GongShuiYing 2017[194]	93	990	0.09	[0.08; 0.11]
GuoMinJian 2017[208−10]	48	2200	0.02	[0.02; 0.03]
LeiYongLiang 2016[311]	46	984	0.05	[0.03; 0.06]
LiJiShan 2016[326−2]	18	41	0.44	[0.28; 0.60]
LiMeng 2020[338−13]	17	1580	0.01	[0.01; 0.02]
LiShouJun 2016[350]	64	1023	0.06	[0.05; 0.08]
LiYueRong 2015[366]	18	952	0.02	[0.01; 0.03]
LiuBaiWei 2017[395]	24	471	0.05	[0.03; 0.07]
LiuShiKe 2016[422]	46	948	0.05	[0.04; 0.06]
LiuWenJun 2018[428−3]	38	528	0.07	[0.05; 0.10]
PangZhiFeng 2017[524]	79	1696	0.05	[0.04; 0.06]
RenFuLin 2013[555]	74	1751	0.04	[0.03; 0.05]
WuXiaYan 2015[730]	92	2046	0.04	[0.04; 0.05]
XiaoSongJian 2017[745]	51	416	0.12	[0.09; 0.16]
XuJianRong 2018[767]	92	2046	0.04	[0.04; 0.05]
XuHao 2016[775]	28	2385	0.01	[0.01; 0.02]
YangYouQing 2016[819]	48	2363	0.02	[0.02; 0.03]
ZhangXiaoYi 2015[891]	21	57	0.37	[0.24; 0.51]
ZhouXiaoHong 2017[961]	47	3464	0.01	[0.01; 0.02]
ZhuChengMing 2017[967]	78	4454	0.02	[0.01; 0.02]
**Common effect model**		**38928**	**0.03**	**[0.03; 0.03]**
**Random effects model**			**0.03**	**[0.02; 0.05]**
Heterogeneity: $I^2 = 96\%$, $\tau^2 = 1.6757$, $p < 0.01$				
**month = 2**				
Ming−Wei Cai 2021[17−1]	25	628	0.04	[0.03; 0.06]
Cai SJ 2018[20]	69	1781	0.04	[0.03; 0.05]
ChenXingHong 2012[102]	478	7113	0.07	[0.06; 0.07]
ChenXingFu 2018[103]	43	1240	0.03	[0.03; 0.05]
DaiYingXue 2018[129−3]	18	1008	0.02	[0.01; 0.03]
DuanRong 2017[152−16]	7	441	0.02	[0.01; 0.03]
DuanRong 2017[152−33]	13	1216	0.01	[0.01; 0.02]
GuoLi 2019[206−58]	16	171	0.09	[0.05; 0.15]
HuHongAn 2015[233]	105	3453	0.03	[0.02; 0.04]
HuangYanHong 2019[259−12]	58	498	0.12	[0.09; 0.15]
LiShiCong 2018[346]	64	1487	0.04	[0.03; 0.05]
LiuWenJun 2018[428−2]	22	388	0.06	[0.04; 0.08]
LiuWenJun 2018[428−9]	105	907	0.12	[0.10; 0.14]
LiuWenJun 2018[428−17]	180	1554	0.12	[0.10; 0.13]
LiuWenJun 2018[428−19]	68	1926	0.04	[0.03; 0.04]
LiuWenJun 2018[428−21]	55	2174	0.03	[0.02; 0.03]
LuoLe 2017[472]	39	1192	0.03	[0.02; 0.04]
MaTao 2018[487]	46	587	0.08	[0.06; 0.10]

SongYuFang 2019[600]	63	783	0.08 [0.06; 0.10]
SunYunLan 2018[624]	63	2212	0.03 [0.02; 0.04]
TangYuHuan 2017[630]	44	2494	0.02 [0.01; 0.02]
TangGuoJie 2019[631]	84	2250	0.04 [0.03; 0.05]
WangJinSheng 2019[645]	56	1800	0.03 [0.02; 0.04]
WangJie 2015[659]	451	11467	0.04 [0.04; 0.04]
WangMin 2018[670−3]	37	672	0.06 [0.04; 0.08]
WangMin 2018[670−4]	27	861	0.03 [0.02; 0.05]
WuGuoFu 2018[717]	17	506	0.03 [0.02; 0.05]
XiaYingPin 2018[743]	177	1777	0.10 [0.09; 0.11]
XuYan 2018[772]	68	969	0.07 [0.05; 0.09]
XuJinFeng 2016[778−1]	10	933	0.01 [0.01; 0.02]
XuJinFeng 2016[778−2]	16	2500	0.01 [0.00; 0.01]
Li J 2018[1015]	19	1327	0.01 [0.01; 0.02]
LiuQin 2011[1088]	257	2497	0.10 [0.09; 0.12]
**Common effect model**		**60812**	**0.05 [0.04; 0.05]**
**Random effects model**			**0.04 [0.03; 0.05]**
Heterogeneity: I^2 = 97%, τ^2 = 0.5593, p < 0.01			
**month = 3**			
Ming−Wei Cai 2021[17−2]	50	693	0.07 [0.05; 0.09]
CaiWenFeng 2014[26]	27	816	0.03 [0.02; 0.05]
ShenYuGang 2016[54]	43	480	0.09 [0.07; 0.12]
ChenXiaoFeng 2015[100]	7	648	0.01 [0.00; 0.02]
ChenYan 2019[104]	59	536	0.11 [0.08; 0.14]
ChenYiXiong 2018[108]	21	1861	0.01 [0.01; 0.02]
DuanRong 2017[152−27]	9	918	0.01 [0.00; 0.02]
FuXiaoFei 2012[171]	20	728	0.03 [0.02; 0.04]
GaoHuiJuan 2015[176]	18	51	0.35 [0.22; 0.50]
GuoLi 2019[206−32]	18	40	0.45 [0.29; 0.62]
GuoLi 2019[206−35]	7	41	0.17 [0.07; 0.32]
GuoLi 2019[206−36]	16	41	0.39 [0.24; 0.55]
GuoLi 2019[206−37]	10	41	0.24 [0.12; 0.40]
GuoLi 2019[206−40]	13	42	0.31 [0.18; 0.47]
GuoLi 2019[206−41]	5	42	0.12 [0.04; 0.26]
GuoLi 2019[206−42]	12	42	0.29 [0.16; 0.45]
GuoLi 2019[206−54]	17	81	0.21 [0.13; 0.31]
GuoLi 2019[206−57]	13	119	0.11 [0.06; 0.18]
GuoMinJian 2017[208−2]	6	39	0.15 [0.06; 0.31]
HuaWeiYu 2018[242−6]	5	833	0.01 [0.00; 0.01]
HuangYanHong 2019[260]	84	760	0.11 [0.09; 0.14]
JiangYingCi 2015[284]	66	1904	0.03 [0.03; 0.04]
LiXiTai 2015[352]	51	1336	0.04 [0.03; 0.05]
LinQiFeng 2018[388]	96	1494	0.06 [0.05; 0.08]
LiuBaiWei 2017[396−1]	43	222	0.19 [0.14; 0.25]
LiuWenJun 2018[428−4]	42	565	0.07 [0.05; 0.10]
LiuWenJun 2018[428−12]	49	1213	0.04 [0.03; 0.05]
LiuWenJun 2018[428−13]	193	1285	0.15 [0.13; 0.17]
LiuWenJun 2018[428−23]	70	3608	0.02 [0.02; 0.02]
LiuYuan 2016[438−2]	121	378	0.32 [0.27; 0.37]
MoYuJie 2018[505]	19	1117	0.02 [0.01; 0.03]
QiYanQiu 2018[532]	27	570	0.05 [0.03; 0.07]
QiYing 2019[535−6]	8	83	0.10 [0.04; 0.18]
QiYing 2019[535−9]	34	1015	0.03 [0.02; 0.05]
ShuaiHuiQun 2012[589]	14	569	0.02 [0.01; 0.04]

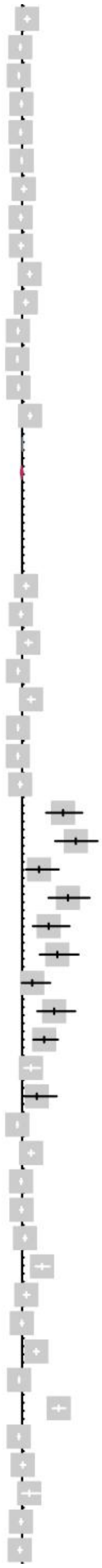

SongHuiRong 2017[597]	34	568	0.06 [0.04; 0.08]
SuTong 2020[604]	55	3432	0.02 [0.01; 0.02]
SunMingHua 2017[614]	36	496	0.07 [0.05; 0.10]
WangMin 2018[670−5]	32	2843	0.01 [0.01; 0.02]
WeiXia 2019[708]	22	1024	0.02 [0.01; 0.03]
WuWenQian 2018[729]	36	2399	0.02 [0.01; 0.02]
WuYang 2018[734]	26	2179	0.01 [0.01; 0.02]
WuZhenYu 2012[738]	19	57	0.33 [0.21; 0.47]
YangJiXing 2019[801]	30	808	0.04 [0.03; 0.05]
YangJing 2018[804]	111	1718	0.06 [0.05; 0.08]
YaoJing 2017[824]	179	885	0.20 [0.18; 0.23]
YeXianMing 2017[832]	94	1156	0.08 [0.07; 0.10]
YuXiaoYun 2018[845]	78	1007	0.08 [0.06; 0.10]
ZhangZhengDong 2017[904]	25	466	0.05 [0.04; 0.08]
ZhaoXueCheng 2016[929]	149	1570	0.09 [0.08; 0.11]
ZhaoYuLi 2019[933]	119	1784	0.07 [0.06; 0.08]
LiYing 2018[1097]	53	1288	0.04 [0.03; 0.05]
**Common effect model**		**47891**	**0.05 [0.05; 0.05]**
**Random effects model**			**0.07 [0.05; 0.09]**
Heterogeneity: $I^2 = 97\%$, $\tau^2 = 1.4103$, $p = 0$			
**month = 4**			
Ming−Wei Cai 2021[17−3]	27	816	0.03 [0.02; 0.05]
Cai W 2017[24−7]	8	39	0.21 [0.09; 0.36]
Cai W 2017[24−9]	12	43	0.28 [0.15; 0.44]
ShenJiChuan 2011[50]	312	1736	0.18 [0.16; 0.20]
CuiLiangLiang 2017[123−2]	14	4600	0.00 [0.00; 0.01]
CuiXiaoMan 2018[125]	17	829	0.02 [0.01; 0.03]
DuanRong 2017[152−26]	24	802	0.03 [0.02; 0.04]
FengZhi 2018[162]	111	2020	0.05 [0.05; 0.07]
GanXiangYang 2014[172]	74	2200	0.03 [0.03; 0.04]
GuoLi 2019[206−22]	10	34	0.29 [0.15; 0.47]
GuoLi 2019[206−38]	18	41	0.44 [0.28; 0.60]
GuoLi 2019[206−47]	9	46	0.20 [0.09; 0.34]
GuoLi 2019[206−53]	23	80	0.29 [0.19; 0.40]
GuoLi 2019[206−61]	28	281	0.10 [0.07; 0.14]
GuoLi 2019[206−63]	30	440	0.07 [0.05; 0.10]
GuoLi 2019[206−64]	25	498	0.05 [0.03; 0.07]
GuoLi 2019[206−65]	28	698	0.04 [0.03; 0.06]
HuaWeiYu 2018[241]	63	1951	0.03 [0.02; 0.04]
HuaWeiYu 2018[242−7]	13	1806	0.01 [0.00; 0.01]
HuangBinBin 2020[245]	41	2530	0.02 [0.01; 0.02]
LiMeng 2020[338−14]	85	1814	0.05 [0.04; 0.06]
LiShiCong 2020[344]	181	3622	0.05 [0.04; 0.06]
LiangRiCheng 2017[376]	27	2518	0.01 [0.01; 0.02]
LiuWenJun 2018[428−22]	52	2332	0.02 [0.02; 0.03]
LiuYing 2019[437]	14	335	0.04 [0.02; 0.07]
NiChaoRong 2019[510]	125	3622	0.03 [0.03; 0.04]
QiYing 2019[535−10]	72	1192	0.06 [0.05; 0.08]
QiYing 2019[535−11]	159	1448	0.11 [0.09; 0.13]
ShiChao 2013[579]	462	2024	0.23 [0.21; 0.25]
TianYaLin 2021[642]	38	546	0.07 [0.05; 0.09]
ZhangQin 2020[885]	57	4279	0.01 [0.01; 0.02]
ZhangQingHe 2020[909]	109	538	0.20 [0.17; 0.24]
**Common effect model**		**45760**	**0.05 [0.05; 0.05]**
**Random effects model**			**0.06 [0.04; 0.09]**
Heterogeneity: $I^2 = 99\%$, $\tau^2 = 1.5647$, $p = 0$			

**month = 5**			
Cai W 2017[24−10]	17	49	0.35 [0.22; 0.50]
ChenQuan 2021[96]	84	821	0.10 [0.08; 0.13]
DuanRong 2017[152−18]	9	538	0.02 [0.01; 0.03]
DuanRong 2017[152−19]	7	565	0.01 [0.00; 0.03]
GuoLi 2019[206−18]	14	32	0.44 [0.26; 0.62]
GuoLi 2019[206−19]	9	33	0.27 [0.13; 0.46]
GuoLi 2019[206−29]	14	38	0.37 [0.22; 0.54]
GuoLi 2019[206−31]	12	39	0.31 [0.17; 0.48]
GuoLi 2019[206−39]	9	41	0.22 [0.11; 0.38]
GuoLi 2019[206−43]	16	43	0.37 [0.23; 0.53]
GuoLi 2019[206−44]	13	44	0.30 [0.17; 0.45]
GuoLi 2019[206−56]	13	101	0.13 [0.07; 0.21]
GuoLi 2019[206−59]	14	211	0.07 [0.04; 0.11]
GuoLi 2019[206−60]	61	220	0.28 [0.22; 0.34]
HuangYanHong 2019[259−3]	27	115	0.23 [0.16; 0.32]
HuangYanHong 2019[259−4]	23	120	0.19 [0.13; 0.27]
HuangYanHong 2019[259−7]	31	146	0.21 [0.15; 0.29]
HuangYanHong 2019[261]	86	1190	0.07 [0.06; 0.09]
JiangYiMei 2017[294]	15	3050	0.00 [0.00; 0.01]
KangQian 2020[303−2]	50	1622	0.03 [0.02; 0.04]
LiJiShan 2016[326−3]	8	46	0.17 [0.08; 0.31]
LiShiE 2018[347−6]	14	94	0.15 [0.08; 0.24]
LiuDan 2019[403]	90	951	0.09 [0.08; 0.12]
LiuDongSheng 2019[404]	80	3720	0.02 [0.02; 0.03]
LiuWenJun 2018[428−14]	46	1314	0.04 [0.03; 0.05]
LiuWenJun 2018[428−15]	17	1328	0.01 [0.01; 0.02]
LiuYuan 2016[438−1]	15	142	0.11 [0.06; 0.17]
QiYing 2019[535−3]	16	38	0.42 [0.26; 0.59]
QiYing 2019[535−5]	13	40	0.32 [0.19; 0.49]
RenQiZhi 2018[557−6]	13	2500	0.01 [0.00; 0.01]
SongCanLei 2020[590]	44	812	0.05 [0.04; 0.07]
WangXiaoDong 2017[688]	69	2708	0.03 [0.02; 0.03]
YuHong 2016[848]	80	1184	0.07 [0.05; 0.08]
ZhangYanMing 2019[898]	15	236	0.06 [0.04; 0.10]
ZhaoMengJiao 2018[919]	87	263	0.33 [0.27; 0.39]
HuangJiaCheng 2021[944]	48	5682	0.01 [0.01; 0.01]
**Common effect model**		**30076**	**0.04 [0.04; 0.04]**
**Random effects model**			**0.09 [0.06; 0.14]**
Heterogeneity: $I^2 = 98\%$, $\tau^2 = 2.0839$, $p < 0.01$			
**month = 6**			
CaiXiuZhi 2018[27−3]	40	1045	0.04 [0.03; 0.05]
GaoZhiYong 2017[187−2]	10	46	0.22 [0.11; 0.36]
GuoLi 2019[206−26]	6	35	0.17 [0.07; 0.34]
GuoLi 2019[206−30]	6	38	0.16 [0.06; 0.31]
GuoLi 2019[206−45]	25	44	0.57 [0.41; 0.72]
GuoLi 2019[206−62]	47	339	0.14 [0.10; 0.18]
GuoMinJian 2017[208−7]	7	43	0.16 [0.07; 0.31]
HeHanZhen 2014[224]	76	578	0.13 [0.11; 0.16]
HuangYanHong 2019[259−10]	26	199	0.13 [0.09; 0.19]
HuangYanHong 2019[259−11]	32	456	0.07 [0.05; 0.10]

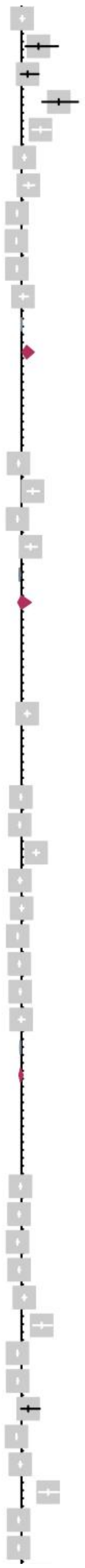

KangQian 2020[303−1]	35	736	0.05	[0.03; 0.07]
LiJiShan 2018[324−2]	7	40	0.17	[0.07; 0.33]
LiJiShan 2018[324−3]	7	78	0.09	[0.04; 0.18]
LiShiE 2018[347−3]	16	48	0.33	[0.20; 0.48]
LiShiE 2018[347−7]	24	126	0.19	[0.13; 0.27]
LiShiE 2018[348]	92	1419	0.06	[0.05; 0.08]
LiuCaiXia 2019[399−3]	20	208	0.10	[0.06; 0.14]
LiuHaoHui 2020[410−9]	17	2191	0.01	[0.00; 0.01]
RenQiZhi 2018[557−5]	5	2000	0.00	[0.00; 0.01]
ZhangChong 2015[862]	13	2050	0.01	[0.00; 0.01]
ZhenGuoXin 2020[937]	11	204	0.05	[0.03; 0.09]
**Common effect model**		**11923**	**0.04**	**[0.04; 0.05]**
**Random effects model**			**0.08**	**[0.05; 0.14]**
Heterogeneity: I^2 = 96%, τ^2 = 1.9333, p < 0.01				
**month = 7**				
CaiXiuZhi 2018[27−4]	24	1279	0.02	[0.01; 0.03]
GaoPeng 2020[181]	28	217	0.13	[0.09; 0.18]
GuoMinJian 2017[208−9]	16	1488	0.01	[0.01; 0.02]
ZhenGuoXin 2020[936]	23	204	0.11	[0.07; 0.16]
**Common effect model**		**3188**	**0.03**	**[0.02; 0.03]**
**Random effects model**			**0.04**	**[0.01; 0.12]**
Heterogeneity: I^2 = 97%, τ^2 = 1.2682, p < 0.01				
**month = 8**				
WuHongXing 2016[718]	63	726	0.09	[0.07; 0.11]
**month = 9**				
DaiBenNa 2020[127]	117	3119	0.04	[0.03; 0.04]
LiBing 2019[315]	67	2354	0.03	[0.02; 0.04]
LiCaiYun 2012[318]	147	940	0.16	[0.13; 0.18]
OuSheXiang 2019[515]	18	603	0.03	[0.02; 0.05]
PengXiaoXue 2015[528]	37	849	0.04	[0.03; 0.06]
QiuHaiYan 2013[551]	76	5965	0.01	[0.01; 0.02]
TaoLiYan 2020[636]	47	1857	0.03	[0.02; 0.03]
XieBin 2020[758]	65	2036	0.03	[0.02; 0.04]
Xiao GD 2019[995]	12	302	0.04	[0.02; 0.07]
**Common effect model**		**18025**	**0.03**	**[0.03; 0.04]**
**Random effects model**			**0.04**	**[0.02; 0.05]**
Heterogeneity: I^2 = 98%, τ^2 = 0.4503, p < 0.01				
**month = 10**				
ShenYiPing 2013[53]	65	1900	0.03	[0.03; 0.04]
DuYueHe 2019[148]	29	1250	0.02	[0.02; 0.03]
DuanRong 2017[152−23]	8	706	0.01	[0.00; 0.02]
GuYiFu 2020[197]	45	1903	0.02	[0.02; 0.03]
HouYuYuan 2014[231]	63	984	0.06	[0.05; 0.08]
HuangYanHong 2019[259−5]	24	120	0.20	[0.13; 0.28]
JiangXianChen 2014[283−1]	16	1485	0.01	[0.01; 0.02]
JiangXianChen 2014[283−2]	23	1877	0.01	[0.01; 0.02]
LiMeng 2020[338−1]	7	74	0.09	[0.04; 0.19]
LiMeng 2020[338−15]	6	1863	0.00	[0.00; 0.01]
LiuWenJun 2018[428−10]	31	951	0.03	[0.02; 0.05]
SunQin 2019[616]	32	128	0.25	[0.18; 0.33]
YangCaiBin 2018[796]	42	2159	0.02	[0.01; 0.03]
YaoJianXiang 2016[822]	59	2992	0.02	[0.02; 0.03]

ZhangHaiYan 2019[867]	145	738	0.20 [0.17; 0.23]
ZhangLi 2015[875−1]	33	474	0.07 [0.05; 0.10]
ZhangLi 2015[875−2]	72	656	0.11 [0.09; 0.14]
Xu H 2013[1059−2]	65	1912	0.03 [0.03; 0.04]
ZouYong 2011[1103]	39	2672	0.01 [0.01; 0.02]
**Common effect model**		**24844**	**0.03 [0.03; 0.03]**
**Random effects model**			**0.04 [0.02; 0.06]**
Heterogeneity: $I^2 = 98\%$, $\tau^2 = 1.3738$, $p < 0.01$			
**month = 11**			
BaiAiLi 2020[3]	20	738	0.03 [0.02; 0.04]
CaiJian 2013[14−1]	53	2219	0.02 [0.02; 0.03]
Ming−Wei Cai 2021[17−4]	56	1202	0.05 [0.04; 0.06]
Cai W 2018[23−3]	217	2504	0.09 [0.08; 0.10]
Cai W 2017[24−6]	24	36	0.67 [0.49; 0.81]
ChenCaiRong 2020[62]	16	275	0.06 [0.03; 0.09]
ChenJian 2016[78]	20	1551	0.01 [0.01; 0.02]
ChenJian 2017[80]	69	6323	0.01 [0.01; 0.01]
DaiYingXue 2018[129−4]	14	1053	0.01 [0.01; 0.02]
DuanRong 2017[152−28]	14	927	0.02 [0.01; 0.03]
GuoLi 2019[206−34]	7	40	0.17 [0.07; 0.33]
GuoLi 2019[206−52]	7	79	0.09 [0.04; 0.17]
GuoMinJian 2017[208−3]	11	39	0.28 [0.15; 0.45]
GuoMinJian 2017[208−6]	7	42	0.17 [0.07; 0.31]
HuangGuo 2015[252]	69	1220	0.06 [0.04; 0.07]
HuangYanHong 2019[259−6]	21	140	0.15 [0.10; 0.22]
JiLei 2018[272−1]	7	55	0.13 [0.05; 0.24]
JiLei 2018[272−3]	18	191	0.09 [0.06; 0.14]
LiBo 2013[316]	120	2303	0.05 [0.04; 0.06]
LiMeng 2020[338−9]	12	749	0.02 [0.01; 0.03]
LiMeng 2020[338−10]	13	754	0.02 [0.01; 0.03]
LiMeng 2020[338−11]	19	1168	0.02 [0.01; 0.03]
LiMeng 2020[338−12]	9	1462	0.01 [0.00; 0.01]
LiMeng 2020[338−16]	17	2026	0.01 [0.00; 0.01]
LiMeng 2020[338−17]	5	2026	0.00 [0.00; 0.01]
LiaoChan 2021[380]	19	675	0.03 [0.02; 0.04]
LinQingShuang 2016[391]	29	603	0.05 [0.03; 0.07]
LiuBaiWei 2017[396−2]	172	3546	0.05 [0.04; 0.06]
LiuHongLian 2018[411]	19	1119	0.02 [0.01; 0.03]
LiuQingLian 2019[420]	37	817	0.05 [0.03; 0.06]
LiuTian 2017[424−7]	35	387	0.09 [0.06; 0.12]
LiuXiuMei 2017[433]	65	1134	0.06 [0.04; 0.07]
LiuYuan 2016[438−3]	55	611	0.09 [0.07; 0.12]
NiChunYan 2020[512]	55	491	0.11 [0.09; 0.14]
SongCanLei 2017[593]	36	888	0.04 [0.03; 0.06]
WangTieJun 2020[678]	15	345	0.04 [0.02; 0.07]
WuJingWen 2020[719]	34	1153	0.03 [0.02; 0.04]
WuXiaoMin 2021[732]	159	1378	0.12 [0.10; 0.13]
WuYiLing 2017[736]	31	1311	0.02 [0.02; 0.03]
XiaZhongFa 2012[744]	209	1616	0.13 [0.11; 0.15]
XuJun 2018[779]	75	2679	0.03 [0.02; 0.03]
ZhanYueWang 2019[860]	38	2311	0.02 [0.01; 0.02]
ZhaoMeiLing 2019[918−2]	39	3305	0.01 [0.01; 0.02]
HuangKaiXiong 2021[989]	36	660	0.05 [0.04; 0.07]
WuLin 2021[1035]	17	194	0.09 [0.05; 0.14]
Xu H 2013[1059−1]	207	1630	0.13 [0.11; 0.14]
**Common effect model**		**55975**	**0.04 [0.04; 0.04]**
**Random effects model**			**0.04 [0.03; 0.06]**
Heterogeneity: $I^2 = 97\%$, $\tau^2 = 1.2964$, $p < 0.01$			

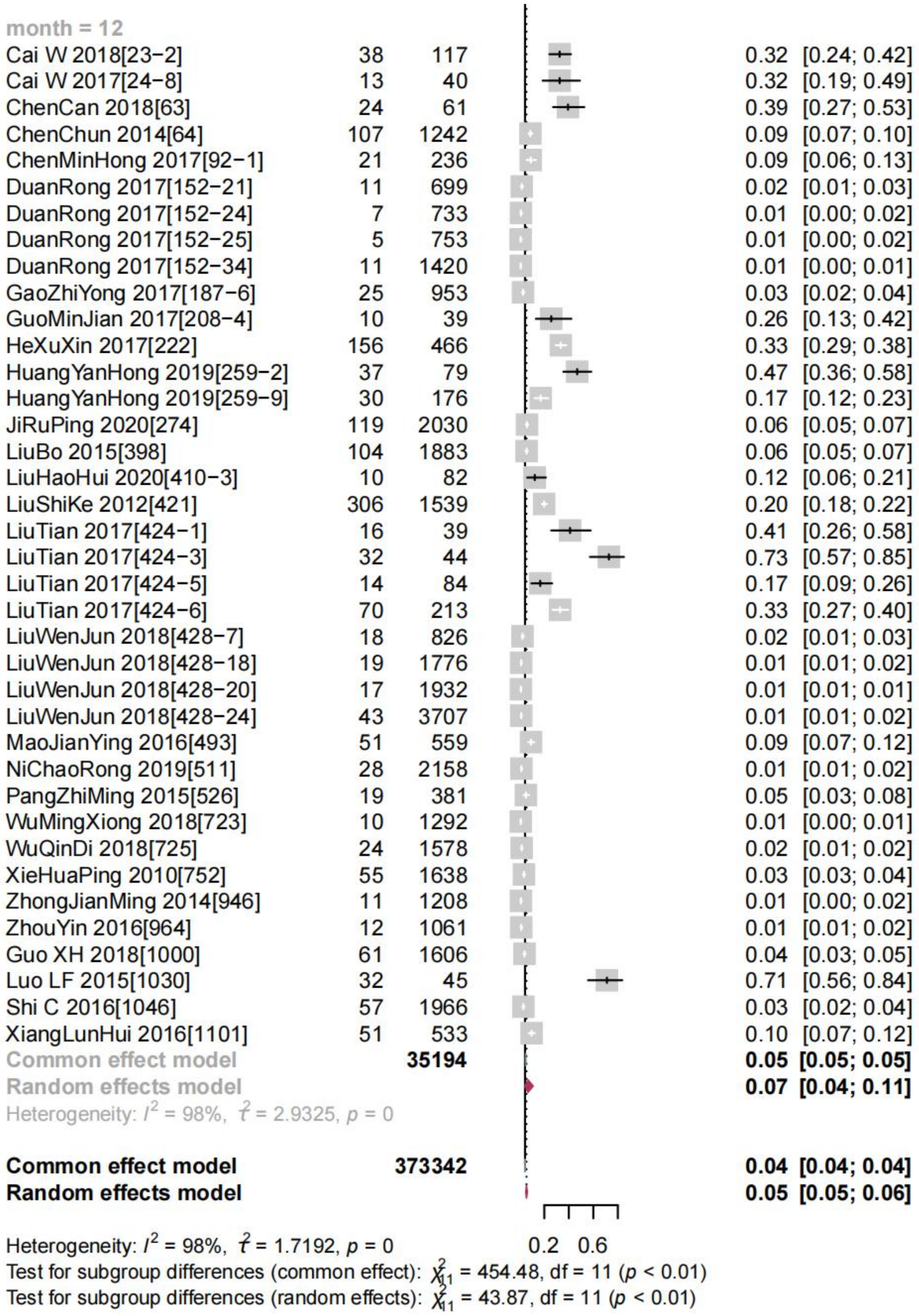
month = 12
Cai W 2018[23−2] 38 117 0.32 [0.24; 0.42]
Cai W 2017[24−8] 13 40 0.32 [0.19; 0.49]
ChenCan 2018[63] 24 61 0.39 [0.27; 0.53]
ChenChun 2014[64] 107 1242 0.09 [0.07; 0.10]
ChenMinHong 2017[92−1] 21 236 0.09 [0.06; 0.13]
DuanRong 2017[152−21] 11 699 0.02 [0.01; 0.03]
DuanRong 2017[152−24] 7 733 0.01 [0.00; 0.02]
DuanRong 2017[152−25] 5 753 0.01 [0.00; 0.02]
DuanRong 2017[152−34] 11 1420 0.01 [0.00; 0.01]
GaoZhiYong 2017[187−6] 25 953 0.03 [0.02; 0.04]
GuoMinJian 2017[208−4] 10 39 0.26 [0.13; 0.42]
HeXuXin 2017[222] 156 466 0.33 [0.29; 0.38]
HuangYanHong 2019[259−2] 37 79 0.47 [0.36; 0.58]
HuangYanHong 2019[259−9] 30 176 0.17 [0.12; 0.23]
JiRuPing 2020[274] 119 2030 0.06 [0.05; 0.07]
LiuBo 2015[398] 104 1883 0.06 [0.05; 0.07]
LiuHaoHui 2020[410−3] 10 82 0.12 [0.06; 0.21]
LiuShiKe 2012[421] 306 1539 0.20 [0.18; 0.22]
LiuTian 2017[424−1] 16 39 0.41 [0.26; 0.58]
LiuTian 2017[424−3] 32 44 0.73 [0.57; 0.85]
LiuTian 2017[424−5] 14 84 0.17 [0.09; 0.26]
LiuTian 2017[424−6] 70 213 0.33 [0.27; 0.40]
LiuWenJun 2018[428−7] 18 826 0.02 [0.01; 0.03]
LiuWenJun 2018[428−18] 19 1776 0.01 [0.01; 0.02]
LiuWenJun 2018[428−20] 17 1932 0.01 [0.01; 0.01]
LiuWenJun 2018[428−24] 43 3707 0.01 [0.01; 0.02]
MaoJianYing 2016[493] 51 559 0.09 [0.07; 0.12]
NiChaoRong 2019[511] 28 2158 0.01 [0.01; 0.02]
PangZhiMing 2015[526] 19 381 0.05 [0.03; 0.08]
WuMingXiong 2018[723] 10 1292 0.01 [0.00; 0.01]
WuQinDi 2018[725] 24 1578 0.02 [0.01; 0.02]
XieHuaPing 2010[752] 55 1638 0.03 [0.03; 0.04]
ZhongJianMing 2014[946] 11 1208 0.01 [0.00; 0.02]
ZhouYin 2016[964] 12 1061 0.01 [0.01; 0.02]
Guo XH 2018[1000] 61 1606 0.04 [0.03; 0.05]
Luo LF 2015[1030] 32 45 0.71 [0.56; 0.84]
Shi C 2016[1046] 57 1966 0.03 [0.02; 0.04]
XiangLunHui 2016[1101] 51 533 0.10 [0.07; 0.12]
Common effect model 35194 0.05 [0.05; 0.05]
Random effects model 0.07 [0.04; 0.11]
Heterogeneity: I^2 = 98%, τ^2 = 2.9325, p = 0
Common effect model 373342 0.04 [0.04; 0.04]
Random effects model 0.05 [0.05; 0.06]
0.2 0.6
Heterogeneity: I^2 = 98%, τ^2 = 1.7192, p = 0
Test for subgroup differences (common effect): χ^2_{11} = 454.48, df = 11 (p < 0.01)
Test for subgroup differences (random effects): χ^2_{11} = 43.87, df = 11 (p < 0.01)

(b7)

Study 18-60y	Events	Total	Attack rate	95%-CI
**month = 1**				
BiHua 2015[11]	55	1262	0.04	[0.03; 0.06]
CaiWenFeng 2013[25]	141	16600	0.01	[0.01; 0.01]
ShenYuGang 2016[55]	233	1702	0.14	[0.12; 0.15]
ChenYiYi 2015[109]	282	38001	0.01	[0.01; 0.01]
GaoZhiPeng 2018[186]	130	397	0.33	[0.28; 0.38]
HuangLiQing 2020[254]	184	1823	0.10	[0.09; 0.12]
LiuXiaoXiao 2014[430]	18	70	0.26	[0.16; 0.38]
QianLiZhen 2020[537]	23	95	0.24	[0.16; 0.34]
XieYuanQi 2016[756]	34	207	0.16	[0.12; 0.22]
ZhangZhen 2015[903]	76	414	0.18	[0.15; 0.22]
**Common effect model**		**60571**	**0.02**	**[0.02; 0.02]**
**Random effects model**			**0.09**	**[0.04; 0.20]**
Heterogeneity: $I^2 = 100\%$, $\tau^2 = 1.9967$, $p = 0$				
**month = 2**				
BiHua 2014[10]	95	18930	0.01	[0.00; 0.01]
ChenAQun 2015[58]	64	19153	0.00	[0.00; 0.00]
ChenAQun 2016[59]	64	18924	0.00	[0.00; 0.00]
ChenGuoCui 2011[69]	27	34	0.79	[0.62; 0.91]
ChenHeJuan 2018[73]	14	49	0.29	[0.17; 0.43]
FengZhi 2020[161]	26	50	0.52	[0.37; 0.66]
GaoShuPing 2019[183]	51	1698	0.03	[0.02; 0.04]
GuShiPing 2009[196]	42	2199	0.02	[0.01; 0.03]
LiXiuFang 2018[359]	20	378	0.05	[0.03; 0.08]
MengJian 2018[495]	37	465	0.08	[0.06; 0.11]
SunZhou 2016[625]	230	45075	0.01	[0.00; 0.01]
WuZhiSheng 2014[741]	176	14612	0.01	[0.01; 0.01]
YangAiQing 2018[795]	40	355	0.11	[0.08; 0.15]
YangZhiWen 2013[820]	11	48	0.23	[0.12; 0.37]
ZhangHuiLing 2018[871]	40	100	0.40	[0.30; 0.50]
**Common effect model**		**122070**	**0.01**	**[0.01; 0.01]**
**Random effects model**			**0.06**	**[0.02; 0.15]**
Heterogeneity: $I^2 = 99\%$, $\tau^2 = 4.6870$, $p = 0$				
**month = 3**				
ChenJianMei 2017[79]	110	9736	0.01	[0.01; 0.01]
ChenMinHong 2017[92−2]	186	657	0.28	[0.25; 0.32]
CuiLiangLiang 2017[123−5]	385	15000	0.03	[0.02; 0.03]
CuiLiangLiang 2017[123−6]	235	20000	0.01	[0.01; 0.01]
GaoZhiYong 2017[187−3]	15	57	0.26	[0.16; 0.40]
GuoLi 2019[205]	75	456	0.16	[0.13; 0.20]
JiJinHua 2018[265]	41	694	0.06	[0.04; 0.08]
JiLei 2020[270]	19	34	0.56	[0.38; 0.73]
JiangChen 2019[285]	26	137	0.19	[0.13; 0.27]
QiXiaoQi 2019[534]	185	2418	0.08	[0.07; 0.09]
WangMingLiang 2020[671]	107	550	0.19	[0.16; 0.23]
XiaGuangHui 2019[742]	106	2300	0.05	[0.04; 0.06]
ZhangHaiYan 2016[868]	78	508	0.15	[0.12; 0.19]
ZhangHengQiu 2014[869]	18	250	0.07	[0.04; 0.11]
ZhangZhiZhong 2020[906]	263	17752	0.01	[0.01; 0.02]
ZhuWeiWei 2018[976]	91	9702	0.01	[0.01; 0.01]
Huang XY 2017[1007]	753	22861	0.03	[0.03; 0.04]
Lu Y 2020[1029]	157	785	0.20	[0.17; 0.23]
**Common effect model**		**103897**	**0.03**	**[0.03; 0.03]**
**Random effects model**			**0.07**	**[0.04; 0.13]**
Heterogeneity: $I^2 = 99\%$, $\tau^2 = 1.8709$, $p = 0$				

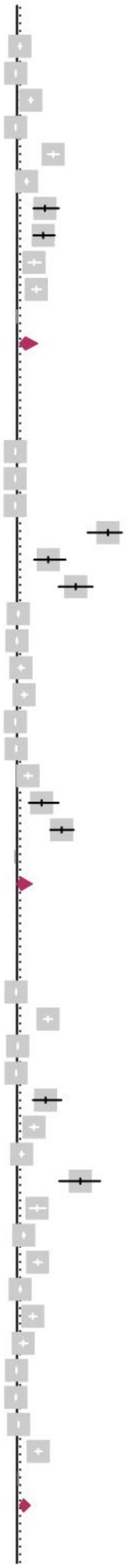

**month = 4**			
GaoRiHong 2019[182]	30	72	0.42 [0.30; 0.54]
HaoYongJian 2020[216]	25	137	0.18 [0.12; 0.26]
LiuJingJing 2018[413]	33	111	0.30 [0.21; 0.39]
LiuYi 2013[436]	9	35	0.26 [0.12; 0.43]
QiYing 2018[535−1]	173	1621	0.11 [0.09; 0.12]
QiYing 2018[535−2]	71	1621	0.04 [0.03; 0.05]
Wu−Yang Shi 2020[577−1]	35	441	0.08 [0.06; 0.11]
TanDongMei 2012[627]	87	2086	0.04 [0.03; 0.05]
WangMan 2017[668]	85	540	0.16 [0.13; 0.19]
ZhangDongSheng 2013[864]	97	6614	0.01 [0.01; 0.02]
ZhouGuoYing 2016[952]	12	75	0.16 [0.09; 0.26]
Wang X 2016[1055]	51	377	0.14 [0.10; 0.17]
Zhou X 2019[1080]	75	1612	0.05 [0.04; 0.06]
**Common effect model**		**15342**	**0.05 [0.05; 0.05]**
**Random effects model**			**0.11 [0.06; 0.18]**
Heterogeneity: I^2 = 98%, τ^2 = 1.0346, p < 0.01			
**month = 5**			
LiJie 2019[329]	106	2868	0.04 [0.03; 0.04]
RenQiZhi 2018[557−7]	15	10000	0.00 [0.00; 0.00]
Liu Y 2015[1025]	99	2049	0.05 [0.04; 0.06]
**Common effect model**		**14917**	**0.01 [0.01; 0.02]**
**Random effects model**			**0.01 [0.00; 0.08]**
Heterogeneity: I^2 = 99%, τ^2 = 2.5272, p < 0.01			
**month = 6**			
Cai MW 2018[18]	34	223	0.15 [0.11; 0.21]
CuiLiangLiang 2017[123−1]	5	90	0.06 [0.02; 0.12]
CuiLiangLiang 2017[123−4]	1434	11000	0.13 [0.12; 0.14]
PanYueFei 2020[522]	45	215	0.21 [0.16; 0.27]
RenYuHua 2016[562]	110	1916	0.06 [0.05; 0.07]
Zhang L 2018[1071]	631	15118	0.04 [0.04; 0.05]
**Common effect model**		**28562**	**0.08 [0.08; 0.08]**
**Random effects model**			**0.09 [0.06; 0.15]**
Heterogeneity: I^2 = 99%, τ^2 = 0.4246, p < 0.01			
**month = 7**			
CaoShen 2019[32]	62	6966	0.01 [0.01; 0.01]
LuWeiWei 2016[462]	406	3808	0.11 [0.10; 0.12]
**Common effect model**		**10774**	**0.04 [0.04; 0.05]**
**Random effects model**			**0.03 [0.01; 0.17]**
Heterogeneity: I^2 = 100%, τ^2 = 1.6724, p < 0.01			
**month = 8**			
MiaoGuoZhong 2018[507]	25	350	0.07 [0.05; 0.10]
WuShuiXin 2012[727]	25	1376	0.02 [0.01; 0.03]
Chen D 2019[987]	209	5043	0.04 [0.04; 0.05]
**Common effect model**		**6769**	**0.04 [0.03; 0.04]**
**Random effects model**			**0.04 [0.02; 0.07]**
Heterogeneity: I^2 = 92%, τ^2 = 0.2892, p < 0.01			

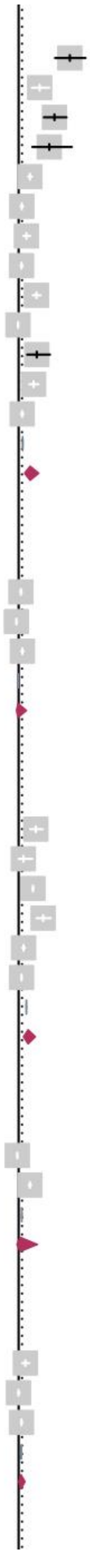

Study	Events	Total	Proportion	95% CI
**month = 9**				
GuKaiChen 2020[195]	21	500	0.04	[0.03; 0.06]
LaiShiMing 2014[308]	105	7113	0.01	[0.01; 0.02]
LiQun 2010[340]	258	14269	0.02	[0.02; 0.02]
LiuKaiQian 2011[415]	634	14439	0.04	[0.04; 0.05]
MaMengMeng 2018[484]	223	30711	0.01	[0.01; 0.01]
WangDaHu 2020[652]	182	33479	0.01	[0.00; 0.01]
ZhouAiHua 2017[950]	80	650	0.12	[0.10; 0.15]
ZhouXiaoHong 2015[958]	107	7113	0.02	[0.01; 0.02]
Y Li 2013[1019]	396	14387	0.03	[0.02; 0.03]
**Common effect model**		**122661**	**0.02**	**[0.02; 0.02]**
**Random effects model**			**0.02**	**[0.01; 0.04]**
Heterogeneity: $I^2 = 99\%$, $\tau^2 = 0.8716$, $p < 0.01$				
**month = 10**				
CuiLiangLiang 2017[123−3]	55	10000	0.01	[0.00; 0.01]
LiShiE 2018[349]	77	8153	0.01	[0.01; 0.01]
**Common effect model**		**18153**	**0.01**	**[0.01; 0.01]**
**Random effects model**			**0.01**	**[0.00; 0.01]**
Heterogeneity: $I^2 = 89\%$, $\tau^2 = 0.0588$, $p < 0.01$				
**month = 11**				
CaiJian 2013[14−2]	257	16056	0.02	[0.01; 0.02]
GaoZhiYong 2017[187−8]	22	1047	0.02	[0.01; 0.03]
HuangGuo 2015[253]	87	13856	0.01	[0.01; 0.01]
HuangSiYue 2020[257]	90	19800	0.00	[0.00; 0.01]
JiangLie 2014[291]	15	590	0.03	[0.01; 0.04]
KuangHaoCheng 2016[307]	667	11300	0.06	[0.05; 0.06]
LiYiLan 2014[365]	228	18930	0.01	[0.01; 0.01]
LuJianYong 2015[456]	60	973	0.06	[0.05; 0.08]
PanYiFeng 2017[521]	82	7396	0.01	[0.01; 0.01]
WangBing 2017[649]	58	20000	0.00	[0.00; 0.00]
XuanLingFeng 2014[784]	17	9523	0.00	[0.00; 0.00]
ZhangPan 2020[883]	16	244	0.07	[0.04; 0.10]
Ruan F 2013[1039]	63	22200	0.00	[0.00; 0.00]
Zhang XF 2020[1076]	27	450	0.06	[0.04; 0.09]
**Common effect model**		**142365**	**0.01**	**[0.01; 0.01]**
**Random effects model**			**0.01**	**[0.01; 0.03]**
Heterogeneity: $I^2 = 99\%$, $\tau^2 = 1.4827$, $p = 0$				
**month = 12**				
LiDaiBo 2018[320]	18	56	0.32	[0.20; 0.46]
LinYanYan 2018[393]	76	3288	0.02	[0.02; 0.03]
LiuBaiWei 2017[396−3]	58	4500	0.01	[0.01; 0.02]
LiuTian 2017[424−4]	9	48	0.19	[0.09; 0.33]
LongJunBiao 2014[441]	13	444	0.03	[0.02; 0.05]
LuZhengXiang 2016[451]	83	4624	0.02	[0.01; 0.02]
SunJing 2015[611]	92	8929	0.01	[0.01; 0.01]
WangHu 2018[656]	72	28270	0.00	[0.00; 0.00]
WeiYiYun 2015[705]	22	155	0.14	[0.09; 0.21]
WuZhaoChun 2015[737]	17	130	0.13	[0.08; 0.20]
Guo Z 2014[1001]	87	7141	0.01	[0.01; 0.02]
**Common effect model**		**57585**	**0.01**	**[0.01; 0.01]**
**Random effects model**			**0.03**	**[0.01; 0.08]**
Heterogeneity: $I^2 = 98\%$, $\tau^2 = 2.2610$, $p < 0.01$				
**Common effect model**		**703666**	**0.02**	**[0.02; 0.02]**
**Random effects model**			**0.05**	**[0.03; 0.06]**

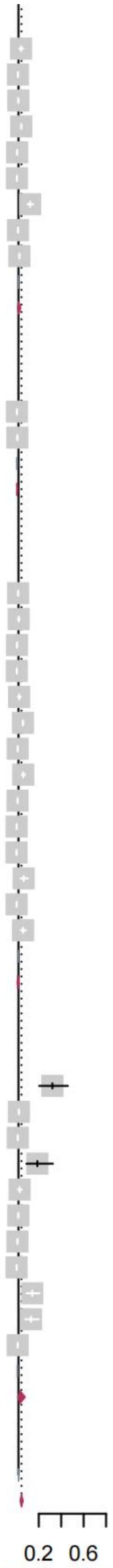

Heterogeneity: $I^2 = 99\%$, $\tau^2 = 2.5492$, $p = 0$
Test for subgroup differences (common effect): $\chi^2_{11} = 7170.25$, df = 11 ($p = 0$)
Test for subgroup differences (random effects): $\chi^2_{11} = 123.01$, df = 11 ($p < 0.01$)

(b8)

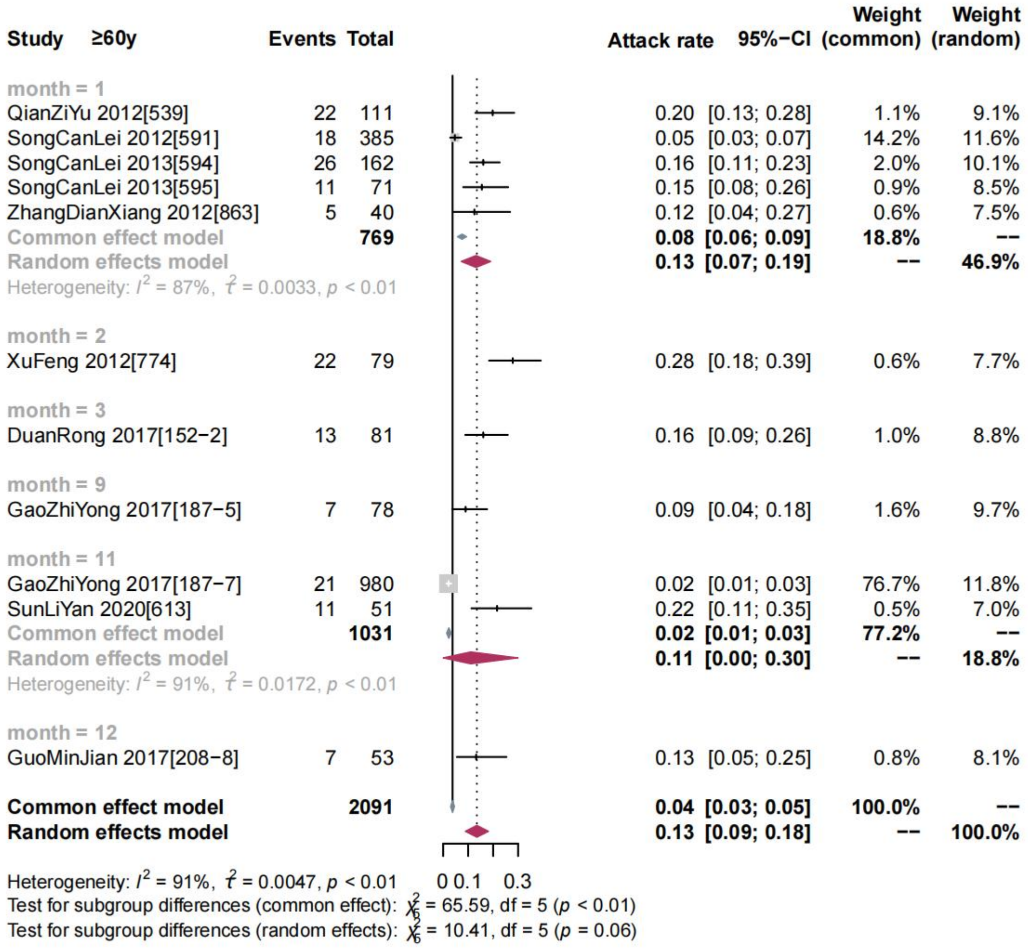

Study ≥60y Events Total Attack rate 95%−CI Weight (common) Weight (random)
month = 1
QianZiYu 2012[539] 22 111 0.20 [0.13; 0.28] 1.1% 9.1%
SongCanLei 2012[591] 18 385 0.05 [0.03; 0.07] 14.2% 11.6%
SongCanLei 2013[594] 26 162 0.16 [0.11; 0.23] 2.0% 10.1%
SongCanLei 2013[595] 11 71 0.15 [0.08; 0.26] 0.9% 8.5%
ZhangDianXiang 2012[863] 5 40 0.12 [0.04; 0.27] 0.6% 7.5%
Common effect model 769 0.08 [0.06; 0.09] 18.8% --
Random effects model 0.13 [0.07; 0.19] -- 46.9%
Heterogeneity: I^2 = 87%, τ^2 = 0.0033, p < 0.01
month = 2
XuFeng 2012[774] 22 79 0.28 [0.18; 0.39] 0.6% 7.7%
month = 3
DuanRong 2017[152−2] 13 81 0.16 [0.09; 0.26] 1.0% 8.8%
month = 9
GaoZhiYong 2017[187−5] 7 78 0.09 [0.04; 0.18] 1.6% 9.7%
month = 11
GaoZhiYong 2017[187−7] 21 980 0.02 [0.01; 0.03] 76.7% 11.8%
SunLiYan 2020[613] 11 51 0.22 [0.11; 0.35] 0.5% 7.0%
Common effect model 1031 0.02 [0.01; 0.03] 77.2% --
Random effects model 0.11 [0.00; 0.30] -- 18.8%
Heterogeneity: I^2 = 91%, τ^2 = 0.0172, p < 0.01
month = 12
GuoMinJian 2017[208−8] 7 53 0.13 [0.05; 0.25] 0.8% 8.1%
Common effect model 2091 0.04 [0.03; 0.05] 100.0% --
Random effects model 0.13 [0.09; 0.18] -- 100.0%
0 0.1 0.3
Heterogeneity: I^2 = 91%, τ^2 = 0.0047, p < 0.01
Test for subgroup differences (common effect): χ^2_5 = 65.59, df = 5 (p < 0.01)
Test for subgroup differences (random effects): χ^2_5 = 10.41, df = 5 (p = 0.06)

(b9)

Study Nursery	Events	Total	Attack rate	95%−CI
**month = 1**				
DaiYingXue 2018[129−1]	14	378	0.04	[0.02; 0.06]
DuanRong 2017[152−13]	5	245	0.02	[0.01; 0.05]
LuHua 2016[454]	14	213	0.07	[0.04; 0.11]
PangZhiFeng 2014[525]	20	548	0.04	[0.02; 0.06]
WangJun 2012[663]	17	400	0.04	[0.02; 0.07]
YangSenPing 2018[810]	30	305	0.10	[0.07; 0.14]
S Kim 2019[1012]	15	48	0.31	[0.19; 0.46]
**Common effect model**		**2137**	**0.05**	**[0.05; 0.06]**
**Random effects model**			**0.06**	**[0.03; 0.11]**
Heterogeneity: $I^2 = 91\%$, $\tau^2 = 0.7447$, $p < 0.01$				
**month = 2**				
CaoXiaoPing 2018[33]	113	1037	0.11	[0.09; 0.13]
ZengLei 2018[44]	28	388	0.07	[0.05; 0.10]
ShenYiPing 2018[52]	26	612	0.04	[0.03; 0.06]
GuoLi 2019[206−21]	8	34	0.24	[0.11; 0.41]
GuoLi 2019[206−48]	18	60	0.30	[0.19; 0.43]
LiuWenJun 2018[428−1]	18	255	0.07	[0.04; 0.11]
LiuWenJun 2018[428−11]	55	955	0.06	[0.04; 0.07]
WuYang 2018[735]	91	516	0.18	[0.14; 0.21]
ZhuChunXiu 2019[968]	17	401	0.04	[0.02; 0.07]
**Common effect model**		**4258**	**0.09**	**[0.08; 0.10]**
**Random effects model**			**0.10**	**[0.06; 0.15]**
Heterogeneity: $I^2 = 93\%$, $\tau^2 = 0.5276$, $p < 0.01$				
**month = 3**				
ChenJingFang 2018[84−2]	14	360	0.04	[0.02; 0.06]
ChenMinHong 2017[92−3]	60	4286	0.01	[0.01; 0.02]
ChenXiaoFeng 2015[100]	7	648	0.01	[0.00; 0.02]
DaiYingXue 2018[129−2]	12	547	0.02	[0.01; 0.04]
DuanRong 2017[152−12]	8	238	0.03	[0.01; 0.07]
GuoLi 2019[206−15]	9	31	0.29	[0.14; 0.48]
GuoLi 2019[206−24]	18	35	0.51	[0.34; 0.69]
GuoLi 2019[206−49]	14	65	0.22	[0.12; 0.33]
GuoLi 2019[206−55]	10	101	0.10	[0.05; 0.17]
HuaWeiYu 2018[242−4]	14	320	0.04	[0.02; 0.07]
HuaWeiYu 2018[242−5]	13	325	0.04	[0.02; 0.07]
JinXiaoFang 2015[302]	38	281	0.14	[0.10; 0.18]
QiYanQiu 2018[532]	27	570	0.05	[0.03; 0.07]
QiYing 2019[535−8]	10	148	0.07	[0.03; 0.12]
RenQiZhi 2018[557−1]	6	100	0.06	[0.02; 0.13]
RuiFang 2018[566−4]	5	382	0.01	[0.00; 0.03]
WangXiaoQin 2015[689]	11	142	0.08	[0.04; 0.13]
YinHongMei 2014[839]	37	511	0.07	[0.05; 0.10]
ZhangZhengDong 2017[904]	25	466	0.05	[0.04; 0.08]
Y Li 2021[1018]	143	346	0.41	[0.36; 0.47]
**Common effect model**		**9902**	**0.05**	**[0.04; 0.05]**
**Random effects model**			**0.07**	**[0.04; 0.11]**
Heterogeneity: $I^2 = 98\%$, $\tau^2 = 1.4308$, $p < 0.01$				

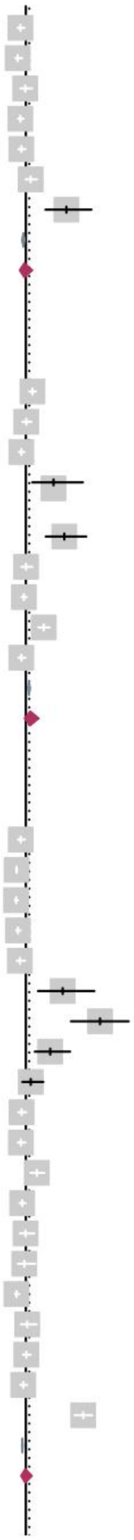

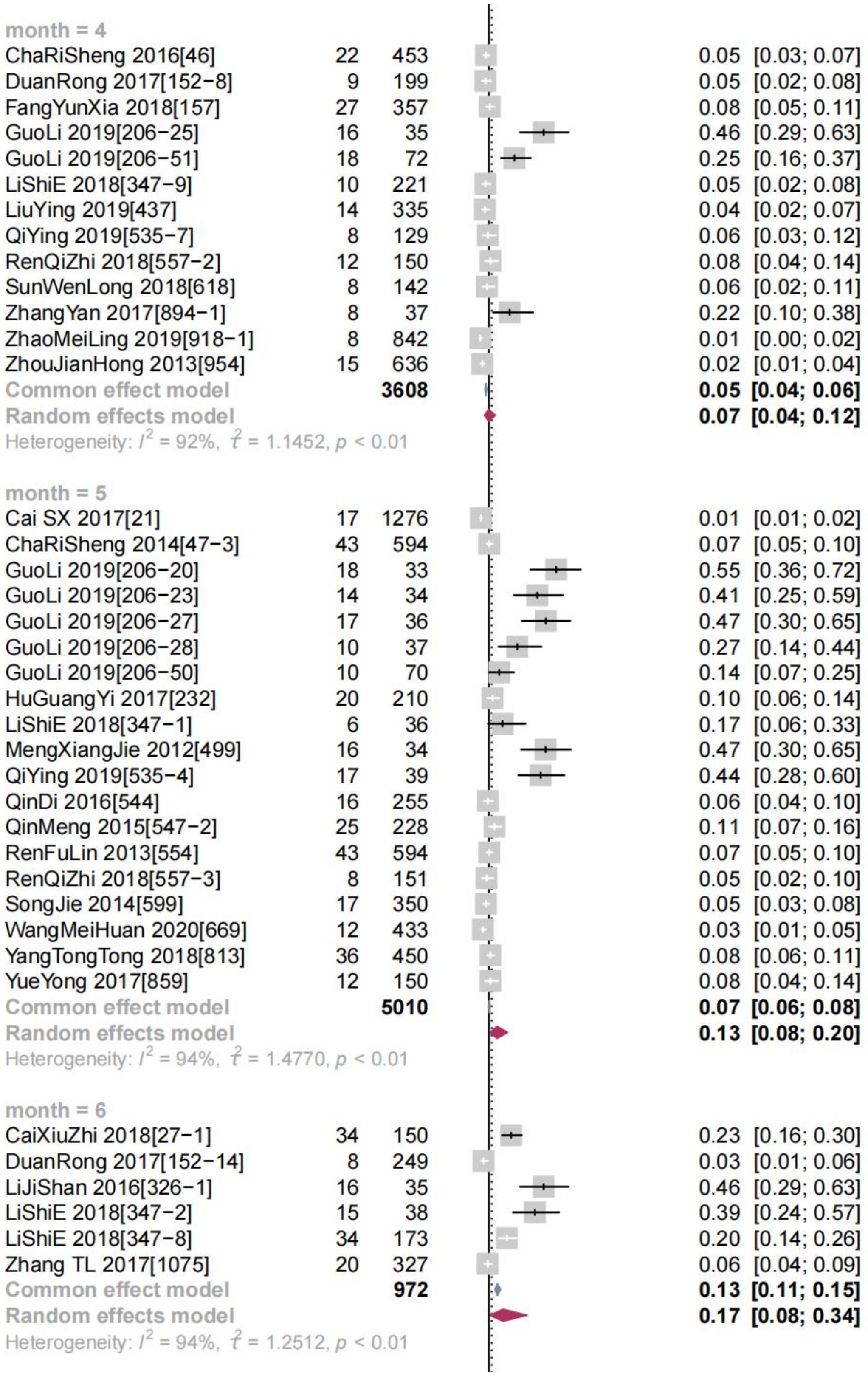
month = 4
ChaRiSheng 2016[46] 22 453 0.05 [0.03; 0.07]
DuanRong 2017[152−8] 9 199 0.05 [0.02; 0.08]
FangYunXia 2018[157] 27 357 0.08 [0.05; 0.11]
GuoLi 2019[206−25] 16 35 0.46 [0.29; 0.63]
GuoLi 2019[206−51] 18 72 0.25 [0.16; 0.37]
LiShiE 2018[347−9] 10 221 0.05 [0.02; 0.08]
LiuYing 2019[437] 14 335 0.04 [0.02; 0.07]
QiYing 2019[535−7] 8 129 0.06 [0.03; 0.12]
RenQiZhi 2018[557−2] 12 150 0.08 [0.04; 0.14]
SunWenLong 2018[618] 8 142 0.06 [0.02; 0.11]
ZhangYan 2017[894−1] 8 37 0.22 [0.10; 0.38]
ZhaoMeiLing 2019[918−1] 8 842 0.01 [0.00; 0.02]
ZhouJianHong 2013[954] 15 636 0.02 [0.01; 0.04]
Common effect model 3608 0.05 [0.04; 0.06]
Random effects model 0.07 [0.04; 0.12]
Heterogeneity: I^2 = 92%, τ^2 = 1.1452, p < 0.01
month = 5
Cai SX 2017[21] 17 1276 0.01 [0.01; 0.02]
ChaRiSheng 2014[47−3] 43 594 0.07 [0.05; 0.10]
GuoLi 2019[206−20] 18 33 0.55 [0.36; 0.72]
GuoLi 2019[206−23] 14 34 0.41 [0.25; 0.59]
GuoLi 2019[206−27] 17 36 0.47 [0.30; 0.65]
GuoLi 2019[206−28] 10 37 0.27 [0.14; 0.44]
GuoLi 2019[206−50] 10 70 0.14 [0.07; 0.25]
HuGuangYi 2017[232] 20 210 0.10 [0.06; 0.14]
LiShiE 2018[347−1] 6 36 0.17 [0.06; 0.33]
MengXiangJie 2012[499] 16 34 0.47 [0.30; 0.65]
QiYing 2019[535−4] 17 39 0.44 [0.28; 0.60]
QinDi 2016[544] 16 255 0.06 [0.04; 0.10]
QinMeng 2015[547−2] 25 228 0.11 [0.07; 0.16]
RenFuLin 2013[554] 43 594 0.07 [0.05; 0.10]
RenQiZhi 2018[557−3] 8 151 0.05 [0.02; 0.10]
SongJie 2014[599] 17 350 0.05 [0.03; 0.08]
WangMeiHuan 2020[669] 12 433 0.03 [0.01; 0.05]
YangTongTong 2018[813] 36 450 0.08 [0.06; 0.11]
YueYong 2017[859] 12 150 0.08 [0.04; 0.14]
Common effect model 5010 0.07 [0.06; 0.08]
Random effects model 0.13 [0.08; 0.20]
Heterogeneity: I^2 = 94%, τ^2 = 1.4770, p < 0.01
month = 6
CaiXiuZhi 2018[27−1] 34 150 0.23 [0.16; 0.30]
DuanRong 2017[152−14] 8 249 0.03 [0.01; 0.06]
LiJiShan 2016[326−1] 16 35 0.46 [0.29; 0.63]
LiShiE 2018[347−2] 15 38 0.39 [0.24; 0.57]
LiShiE 2018[347−8] 34 173 0.20 [0.14; 0.26]
Zhang TL 2017[1075] 20 327 0.06 [0.04; 0.09]
Common effect model 972 0.13 [0.11; 0.15]
Random effects model 0.17 [0.08; 0.34]
Heterogeneity: I^2 = 94%, τ^2 = 1.2512, p < 0.01

**month = 7**				
LiXiaoJing 2021[992]	7	251	0.03	[0.01; 0.06]
**month = 9**				
HuaWeiYu 2018[242−2]	17	267	0.06	[0.04; 0.10]
LiaoKeChang 2018[381]	41	161	0.25	[0.19; 0.33]
LiuHaoHui 2020[410−4]	14	166	0.08	[0.05; 0.14]
LiuHaoHui 2020[410−5]	9	202	0.04	[0.02; 0.08]
OuSheXiang 2019[515]	18	603	0.03	[0.02; 0.05]
WangKaiLiang 2019[665]	16	446	0.04	[0.02; 0.06]
WeiGuiYing 2019[706]	29	257	0.11	[0.08; 0.16]
ZhangAiHua 2018[861]	19	214	0.09	[0.05; 0.14]
ZhouLiHong 2016[956]	13	37	0.35	[0.20; 0.53]
**Common effect model**		**2353**	**0.07**	**[0.06; 0.09]**
**Random effects model**			**0.09**	**[0.05; 0.15]**
Heterogeneity: $I^2 = 93\%$, $\tau^2 = 0.7548$, $p < 0.01$				
**month = 10**				
ZengFengMei 2020[41]	25	255	0.10	[0.06; 0.14]
CuiXiaoMan 2015[124]	34	332	0.10	[0.07; 0.14]
DuanRong 2017[152−6]	11	179	0.06	[0.03; 0.11]
DuanRong 2017[152−7]	10	182	0.05	[0.03; 0.10]
DuanRong 2017[152−10]	36	227	0.16	[0.11; 0.21]
DuanRong 2017[152−11]	5	229	0.02	[0.01; 0.05]
GaoHaiMing 2014[174]	19	187	0.10	[0.06; 0.15]
GaoZhiYong 2017[187−1]	12	32	0.38	[0.21; 0.56]
GuoMinJian 2017[208−1]	8	31	0.26	[0.12; 0.45]
HuaWeiYu 2018[242−1]	15	156	0.10	[0.05; 0.15]
HuaWeiYu 2018[242−3]	9	290	0.03	[0.01; 0.06]
JiangXiHong 2019[293]	40	304	0.13	[0.10; 0.17]
LiMeng 2020[338−8]	11	659	0.02	[0.01; 0.03]
LiuHaoHui 2020[410−1]	6	35	0.17	[0.07; 0.34]
LiuHaoHui 2020[410−2]	28	53	0.53	[0.39; 0.67]
LiuHaoHui 2020[410−6]	16	300	0.05	[0.03; 0.09]
LiuHaoHui 2020[410−7]	12	561	0.02	[0.01; 0.04]
LiuWenJun 2018[428−6]	36	700	0.05	[0.04; 0.07]
LiuWenJun 2018[428−8]	16	904	0.02	[0.01; 0.03]
RuiFang 2018[566−6]	16	670	0.02	[0.01; 0.04]
SunQin 2019[616]	32	128	0.25	[0.18; 0.33]
WangHua 2014[657]	28	497	0.06	[0.04; 0.08]
YanGeBin 2017[786−1]	14	35	0.40	[0.24; 0.58]
YanGeBin 2017[786−2]	25	62	0.40	[0.28; 0.54]
YangRongXing 2019[808]	15	334	0.04	[0.03; 0.07]
ZhangNing 2018[882]	31	340	0.09	[0.06; 0.13]
ZhangGuangMing 2018[907]	14	149	0.09	[0.05; 0.15]
**Common effect model**		**7831**	**0.07**	**[0.06; 0.07]**
**Random effects model**			**0.09**	**[0.06; 0.13]**
Heterogeneity: $I^2 = 94\%$, $\tau^2 = 1.2603$, $p < 0.01$				
**month = 11**				
Cai W 2017[24−4]	12	32	0.38	[0.21; 0.56]
ChenJingFang 2018[84−1]	18	316	0.06	[0.03; 0.09]
DuanRong 2017[152−3]	11	89	0.12	[0.06; 0.21]
DuanRong 2017[152−15]	8	271	0.03	[0.01; 0.06]

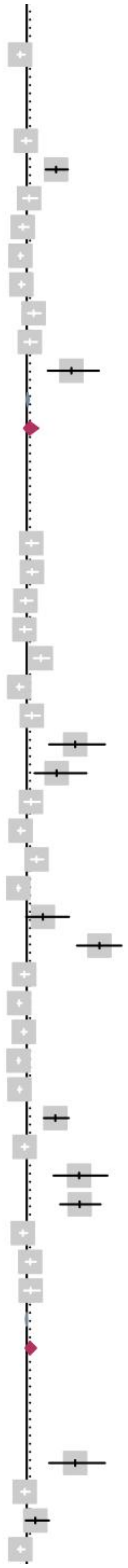

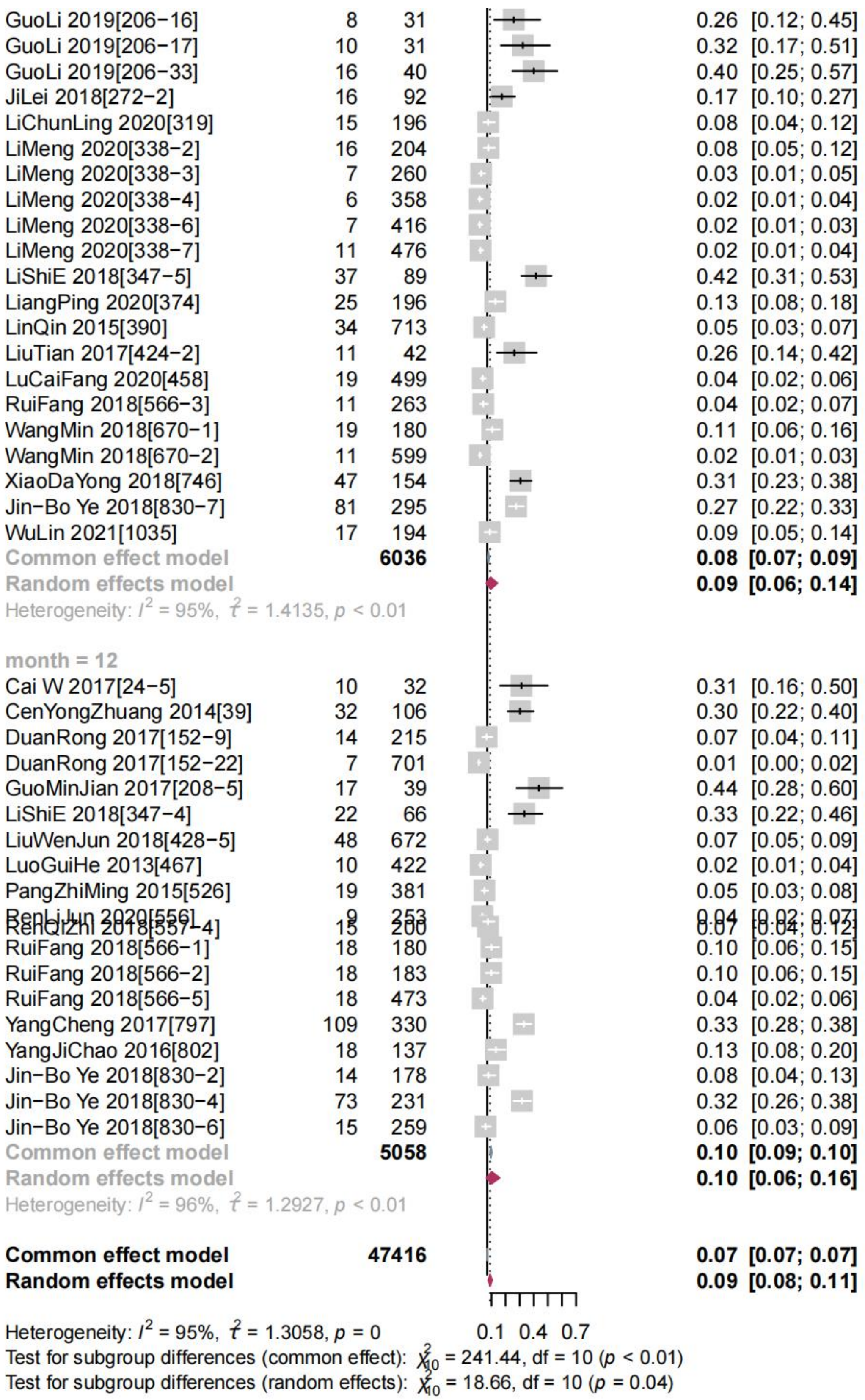
GuoLi 2019[206−16] 8 31 0.26 [0.12; 0.45]
GuoLi 2019[206−17] 10 31 0.32 [0.17; 0.51]
GuoLi 2019[206−33] 16 40 0.40 [0.25; 0.57]
JiLei 2018[272−2] 16 92 0.17 [0.10; 0.27]
LiChunLing 2020[319] 15 196 0.08 [0.04; 0.12]
LiMeng 2020[338−2] 16 204 0.08 [0.05; 0.12]
LiMeng 2020[338−3] 7 260 0.03 [0.01; 0.05]
LiMeng 2020[338−4] 6 358 0.02 [0.01; 0.04]
LiMeng 2020[338−6] 7 416 0.02 [0.01; 0.03]
LiMeng 2020[338−7] 11 476 0.02 [0.01; 0.04]
LiShiE 2018[347−5] 37 89 0.42 [0.31; 0.53]
LiangPing 2020[374] 25 196 0.13 [0.08; 0.18]
LinQin 2015[390] 34 713 0.05 [0.03; 0.07]
LiuTian 2017[424−2] 11 42 0.26 [0.14; 0.42]
LuCaiFang 2020[458] 19 499 0.04 [0.02; 0.06]
RuiFang 2018[566−3] 11 263 0.04 [0.02; 0.07]
WangMin 2018[670−1] 19 180 0.11 [0.06; 0.16]
WangMin 2018[670−2] 11 599 0.02 [0.01; 0.03]
XiaoDaYong 2018[746] 47 154 0.31 [0.23; 0.38]
Jin−Bo Ye 2018[830−7] 81 295 0.27 [0.22; 0.33]
WuLin 2021[1035] 17 194 0.09 [0.05; 0.14]
Common effect model 6036 0.08 [0.07; 0.09]
Random effects model 0.09 [0.06; 0.14]
Heterogeneity: I^2 = 95%, τ^2 = 1.4135, p < 0.01
month = 12
Cai W 2017[24−5] 10 32 0.31 [0.16; 0.50]
CenYongZhuang 2014[39] 32 106 0.30 [0.22; 0.40]
DuanRong 2017[152−9] 14 215 0.07 [0.04; 0.11]
DuanRong 2017[152−22] 7 701 0.01 [0.00; 0.02]
GuoMinJian 2017[208−5] 17 39 0.44 [0.28; 0.60]
LiShiE 2018[347−4] 22 66 0.33 [0.22; 0.46]
LiuWenJun 2018[428−5] 48 672 0.07 [0.05; 0.09]
LuoGuiHe 2013[467] 10 422 0.02 [0.01; 0.04]
PangZhiMing 2015[526] 19 381 0.05 [0.03; 0.08]
RenLiJun 2020[556] 9 253 0.04 [0.02; 0.07]
RenQiZhi 2018[557−4] 15 200 0.07 [0.04; 0.12]
RuiFang 2018[566−1] 18 180 0.10 [0.06; 0.15]
RuiFang 2018[566−2] 18 183 0.10 [0.06; 0.15]
RuiFang 2018[566−5] 18 473 0.04 [0.02; 0.06]
YangCheng 2017[797] 109 330 0.33 [0.28; 0.38]
YangJiChao 2016[802] 18 137 0.13 [0.08; 0.20]
Jin−Bo Ye 2018[830−2] 14 178 0.08 [0.04; 0.13]
Jin−Bo Ye 2018[830−4] 73 231 0.32 [0.26; 0.38]
Jin−Bo Ye 2018[830−6] 15 259 0.06 [0.03; 0.09]
Common effect model 5058 0.10 [0.09; 0.10]
Random effects model 0.10 [0.06; 0.16]
Heterogeneity: I^2 = 96%, τ^2 = 1.2927, p < 0.01
Common effect model 47416 0.07 [0.07; 0.07]
Random effects model 0.09 [0.08; 0.11]
0.1 0.4 0.7
Heterogeneity: I^2 = 95%, τ^2 = 1.3058, p = 0
Test for subgroup differences (common effect): χ^2_{10} = 241.44, df = 10 (p < 0.01)
Test for subgroup differences (random effects): χ^2_{10} = 18.66, df = 10 (p = 0.04)

(b10)

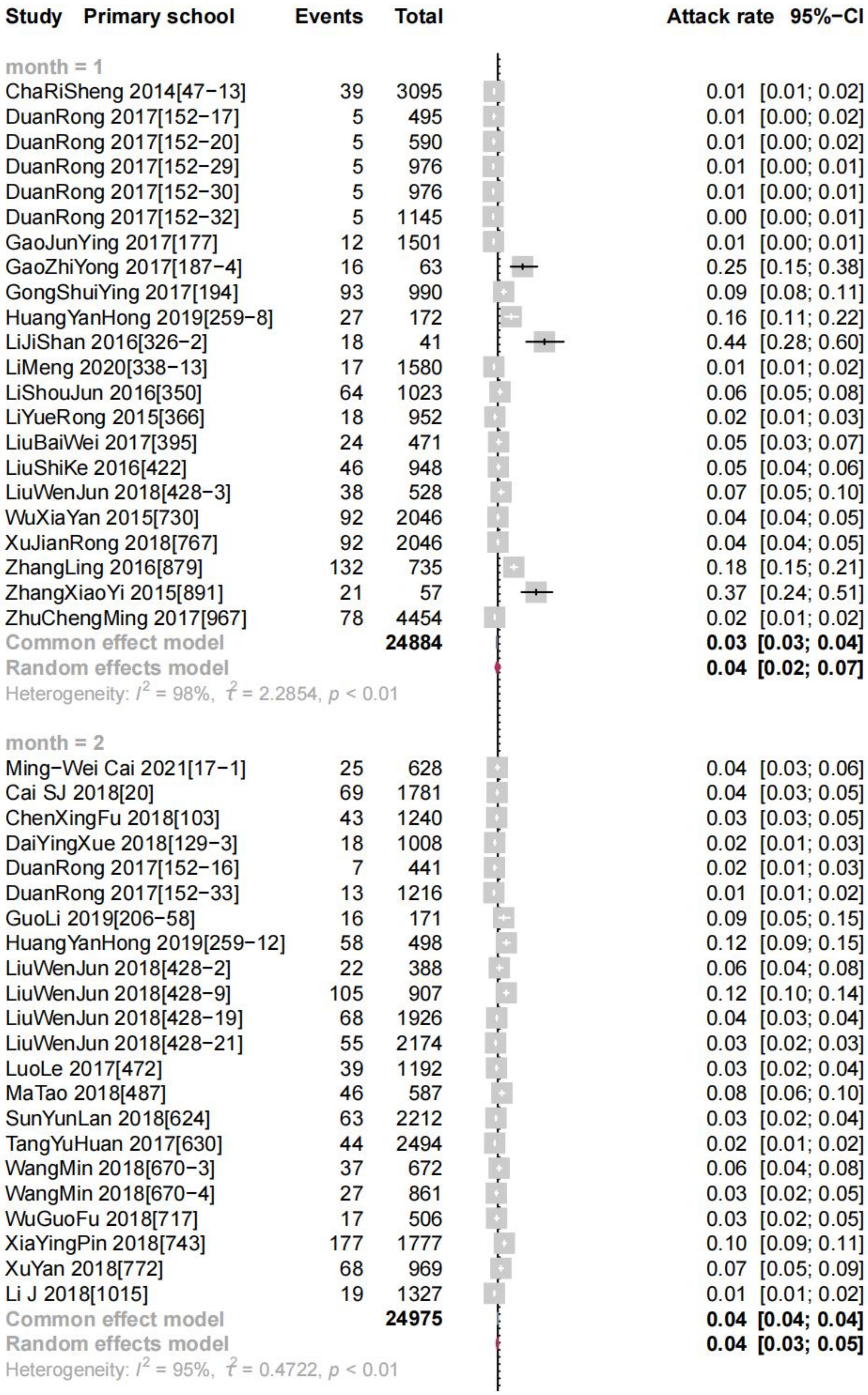
Study Primary school Events Total Attack rate 95%−CI
month = 1
ChaRiSheng 2014[47−13] 39 3095 0.01 [0.01; 0.02]
DuanRong 2017[152−17] 5 495 0.01 [0.00; 0.02]
DuanRong 2017[152−20] 5 590 0.01 [0.00; 0.02]
DuanRong 2017[152−29] 5 976 0.01 [0.00; 0.01]
DuanRong 2017[152−30] 5 976 0.01 [0.00; 0.01]
DuanRong 2017[152−32] 5 1145 0.00 [0.00; 0.01]
GaoJunYing 2017[177] 12 1501 0.01 [0.00; 0.01]
GaoZhiYong 2017[187−4] 16 63 0.25 [0.15; 0.38]
GongShuiYing 2017[194] 93 990 0.09 [0.08; 0.11]
HuangYanHong 2019[259−8] 27 172 0.16 [0.11; 0.22]
LiJiShan 2016[326−2] 18 41 0.44 [0.28; 0.60]
LiMeng 2020[338−13] 17 1580 0.01 [0.01; 0.02]
LiShouJun 2016[350] 64 1023 0.06 [0.05; 0.08]
LiYueRong 2015[366] 18 952 0.02 [0.01; 0.03]
LiuBaiWei 2017[395] 24 471 0.05 [0.03; 0.07]
LiuShiKe 2016[422] 46 948 0.05 [0.04; 0.06]
LiuWenJun 2018[428−3] 38 528 0.07 [0.05; 0.10]
WuXiaYan 2015[730] 92 2046 0.04 [0.04; 0.05]
XuJianRong 2018[767] 92 2046 0.04 [0.04; 0.05]
ZhangLing 2016[879] 132 735 0.18 [0.15; 0.21]
ZhangXiaoYi 2015[891] 21 57 0.37 [0.24; 0.51]
ZhuChengMing 2017[967] 78 4454 0.02 [0.01; 0.02]
Common effect model 24884 0.03 [0.03; 0.04]
Random effects model 0.04 [0.02; 0.07]
Heterogeneity: I^2 = 98%, τ^2 = 2.2854, p < 0.01
month = 2
Ming−Wei Cai 2021[17−1] 25 628 0.04 [0.03; 0.06]
Cai SJ 2018[20] 69 1781 0.04 [0.03; 0.05]
ChenXingFu 2018[103] 43 1240 0.03 [0.03; 0.05]
DaiYingXue 2018[129−3] 18 1008 0.02 [0.01; 0.03]
DuanRong 2017[152−16] 7 441 0.02 [0.01; 0.03]
DuanRong 2017[152−33] 13 1216 0.01 [0.01; 0.02]
GuoLi 2019[206−58] 16 171 0.09 [0.05; 0.15]
HuangYanHong 2019[259−12] 58 498 0.12 [0.09; 0.15]
LiuWenJun 2018[428−2] 22 388 0.06 [0.04; 0.08]
LiuWenJun 2018[428−9] 105 907 0.12 [0.10; 0.14]
LiuWenJun 2018[428−19] 68 1926 0.04 [0.03; 0.04]
LiuWenJun 2018[428−21] 55 2174 0.03 [0.02; 0.03]
LuoLe 2017[472] 39 1192 0.03 [0.02; 0.04]
MaTao 2018[487] 46 587 0.08 [0.06; 0.10]
SunYunLan 2018[624] 63 2212 0.03 [0.02; 0.04]
TangYuHuan 2017[630] 44 2494 0.02 [0.01; 0.02]
WangMin 2018[670−3] 37 672 0.06 [0.04; 0.08]
WangMin 2018[670−4] 27 861 0.03 [0.02; 0.05]
WuGuoFu 2018[717] 17 506 0.03 [0.02; 0.05]
XiaYingPin 2018[743] 177 1777 0.10 [0.09; 0.11]
XuYan 2018[772] 68 969 0.07 [0.05; 0.09]
Li J 2018[1015] 19 1327 0.01 [0.01; 0.02]
Common effect model 24975 0.04 [0.04; 0.04]
Random effects model 0.04 [0.03; 0.05]
Heterogeneity: I^2 = 95%, τ^2 = 0.4722, p < 0.01

**month = 3**			
Ming−Wei Cai 2021[17−2]	50	693	0.07 [0.05; 0.09]
CaiWenFeng 2014[26]	27	816	0.03 [0.02; 0.05]
ChaRiSheng 2014[47−5]	16	1168	0.01 [0.01; 0.02]
ChenYiXiong 2018[108]	21	1861	0.01 [0.01; 0.02]
ChenZhiQiong 2017[115]	255	1843	0.14 [0.12; 0.15]
DuanRong 2017[152−27]	9	918	0.01 [0.00; 0.02]
FuXiaoFei 2012[171]	20	728	0.03 [0.02; 0.04]
GaoHuiJuan 2015[176]	18	51	0.35 [0.22; 0.50]
GuoLi 2019[206−32]	18	40	0.45 [0.29; 0.62]
GuoLi 2019[206−35]	7	41	0.17 [0.07; 0.32]
GuoLi 2019[206−36]	16	41	0.39 [0.24; 0.55]
GuoLi 2019[206−37]	10	41	0.24 [0.12; 0.40]
GuoLi 2019[206−40]	13	42	0.31 [0.18; 0.47]
GuoLi 2019[206−41]	5	42	0.12 [0.04; 0.26]
GuoLi 2019[206−42]	12	42	0.29 [0.16; 0.45]
GuoLi 2019[206−54]	17	81	0.21 [0.13; 0.31]
HuaWeiYu 2018[242−6]	5	833	0.01 [0.00; 0.01]
HuangYanHong 2019[260]	84	760	0.11 [0.09; 0.14]
LiXiTai 2015[352]	51	1336	0.04 [0.03; 0.05]
LiuWenJun 2018[428−4]	42	565	0.07 [0.05; 0.10]
LiuWenJun 2018[428−13]	193	1285	0.15 [0.13; 0.17]
LiuWenJun 2018[428−16]	54	1467	0.04 [0.03; 0.05]
LiuWenJun 2018[428−23]	70	3608	0.02 [0.02; 0.02]
LiuYuan 2016[438−2]	121	378	0.32 [0.27; 0.37]
MoYuJie 2018[505]	19	1117	0.02 [0.01; 0.03]
RuiFang 2018[566−8]	8	1059	0.01 [0.00; 0.01]
RuiFang 2018[566−10]	10	2084	0.00 [0.00; 0.01]
ShuaiHuiQun 2012[589]	14	569	0.02 [0.01; 0.04]
SuTong 2020[604]	55	3432	0.02 [0.01; 0.02]
SunMingHua 2017[614]	36	496	0.07 [0.05; 0.10]
WeiXia 2019[708]	22	1024	0.02 [0.01; 0.03]
WuWenQian 2018[729]	36	2399	0.02 [0.01; 0.02]
WuYang 2018[734]	26	2179	0.01 [0.01; 0.02]
WuZhenYu 2012[738]	19	57	0.33 [0.21; 0.47]
YaoJing 2017[824]	179	885	0.20 [0.18; 0.23]
ZhaoXueCheng 2016[929]	149	1570	0.09 [0.08; 0.11]
ZhaoYuLi 2019[933]	119	1784	0.07 [0.06; 0.08]
Huang Z 2020[1008]	68	786	0.09 [0.07; 0.11]
**Common effect model**		**38121**	**0.05 [0.05; 0.05]**
**Random effects model**			**0.06 [0.04; 0.09]**
Heterogeneity: $I^2 = 98\%$, $\tau^2 = 2.0695$, $p = 0$			
**month = 4**			
Ming−Wei Cai 2021[17−3]	27	816	0.03 [0.02; 0.05]
Cai W 2017[24−7]	8	39	0.21 [0.09; 0.36]
Cai W 2017[24−9]	12	43	0.28 [0.15; 0.44]
Cai W 2017[24−12]	16	78	0.21 [0.12; 0.31]
CuiXiaoMan 2018[125]	17	829	0.02 [0.01; 0.03]
DuanRong 2017[152−26]	24	802	0.03 [0.02; 0.04]
FengZhi 2018[162]	111	2020	0.05 [0.05; 0.07]
GuoLi 2019[206−22]	10	34	0.29 [0.15; 0.47]
GuoLi 2019[206−38]	18	41	0.44 [0.28; 0.60]
GuoLi 2019[206−53]	23	80	0.29 [0.19; 0.40]
GuoLi 2019[206−63]	30	440	0.07 [0.05; 0.10]
HuaWeiYu 2018[241]	63	1951	0.03 [0.02; 0.04]
HuaWeiYu 2018[242−7]	13	1806	0.01 [0.00; 0.01]
LiuHaoHui 2020[410−8]	145	618	0.23 [0.20; 0.27]
NiChaoRong 2019[510]	125	3622	0.03 [0.03; 0.04]
QiYing 2019[535−10]	72	1192	0.06 [0.05; 0.08]
ZhangQin 2020[885]	57	4279	0.01 [0.01; 0.02]
ZhangYan 2017[894−2]	12	2857	0.00 [0.00; 0.01]
ZhangQingHe 2020[909]	109	538	0.20 [0.17; 0.24]
**Common effect model**		**22085**	**0.04 [0.04; 0.04]**
**Random effects model**			**0.07 [0.04; 0.13]**
Heterogeneity: $I^2 = 98\%$, $\tau^2 = 2.0978$, $p < 0.01$			

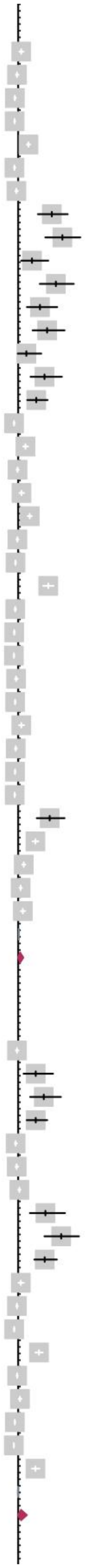

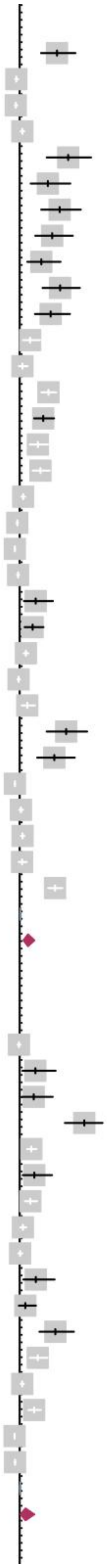

**month = 5**				
Cai W 2017[24−10]	17	49	0.35	[0.22; 0.50]
DuanRong 2017[152−18]	9	538	0.02	[0.01; 0.03]
DuanRong 2017[152−19]	7	565	0.01	[0.00; 0.03]
GuiGuoPing 2018[200]	79	1204	0.07	[0.05; 0.08]
GuoLi 2019[206−18]	14	32	0.44	[0.26; 0.62]
GuoLi 2019[206−19]	9	33	0.27	[0.13; 0.46]
GuoLi 2019[206−29]	14	38	0.37	[0.22; 0.54]
GuoLi 2019[206−31]	12	39	0.31	[0.17; 0.48]
GuoLi 2019[206−39]	9	41	0.22	[0.11; 0.38]
GuoLi 2019[206−43]	16	43	0.37	[0.23; 0.53]
GuoLi 2019[206−44]	13	44	0.30	[0.17; 0.45]
GuoLi 2019[206−56]	13	101	0.13	[0.07; 0.21]
GuoLi 2019[206−59]	14	211	0.07	[0.04; 0.11]
GuoLi 2019[206−60]	61	220	0.28	[0.22; 0.34]
HuangYanHong 2019[259−3]	27	115	0.23	[0.16; 0.32]
HuangYanHong 2019[259−4]	23	120	0.19	[0.13; 0.27]
HuangYanHong 2019[259−7]	31	146	0.21	[0.15; 0.29]
HuangYanHong 2019[261]	86	1190	0.07	[0.06; 0.09]
JiHong 2015[264]	78	3194	0.02	[0.02; 0.03]
JiangYiMei 2017[294]	15	3050	0.00	[0.00; 0.01]
KangQian 2020[303−2]	50	1622	0.03	[0.02; 0.04]
LiJiShan 2016[326−3]	8	46	0.17	[0.08; 0.31]
LiShiE 2018[347−6]	14	94	0.15	[0.08; 0.24]
LiuDan 2019[403]	90	951	0.09	[0.08; 0.12]
LiuWenJun 2018[428−14]	46	1314	0.04	[0.03; 0.05]
LiuYuan 2016[438−1]	15	142	0.11	[0.06; 0.17]
QiYing 2019[535−3]	16	38	0.42	[0.26; 0.59]
QiYing 2019[535−5]	13	40	0.32	[0.19; 0.49]
RenQiZhi 2018[557−6]	13	2500	0.01	[0.00; 0.01]
SongCanLei 2020[590]	44	812	0.05	[0.04; 0.07]
YuHong 2016[848]	80	1184	0.07	[0.05; 0.08]
ZhangYanMing 2019[898]	15	236	0.06	[0.04; 0.10]
ZhaoMengJiao 2018[919]	87	263	0.33	[0.27; 0.39]
**Common effect model**		**20215**	**0.05**	**[0.05; 0.05]**
**Random effects model**			**0.11**	**[0.07; 0.17]**
Heterogeneity: $I^2 = 97\%$, $\tau^2 = 1.8444$, $p < 0.01$				
**month = 6**				
CaiXiuZhi 2018[27−3]	40	1045	0.04	[0.03; 0.05]
GuoLi 2019[206−26]	6	35	0.17	[0.07; 0.34]
GuoLi 2019[206−30]	6	38	0.16	[0.06; 0.31]
GuoLi 2019[206−45]	25	44	0.57	[0.41; 0.72]
GuoLi 2019[206−62]	47	339	0.14	[0.10; 0.18]
GuoMinJian 2017[208−7]	7	43	0.16	[0.07; 0.31]
HuangYanHong 2019[259−10]	26	199	0.13	[0.09; 0.19]
HuangYanHong 2019[259−11]	32	456	0.07	[0.05; 0.10]
KangQian 2020[303−1]	35	736	0.05	[0.03; 0.07]
LiJiShan 2018[324−2]	7	40	0.17	[0.07; 0.33]
LiJiShan 2018[324−3]	7	78	0.09	[0.04; 0.18]
LiShiE 2018[347−3]	16	48	0.33	[0.20; 0.48]
LiShiE 2018[347−7]	24	126	0.19	[0.13; 0.27]
LiShiE 2018[348]	92	1419	0.06	[0.05; 0.08]
QinMeng 2015[547−1]	35	217	0.16	[0.11; 0.22]
RenQiZhi 2018[557−5]	5	2000	0.00	[0.00; 0.01]
ZhangChong 2015[862]	13	2050	0.01	[0.00; 0.01]
**Common effect model**		**8913**	**0.05**	**[0.04; 0.05]**
**Random effects model**			**0.09**	**[0.05; 0.17]**
Heterogeneity: $I^2 = 96\%$, $\tau^2 = 1.9644$, $p < 0.01$				

Study	Events	Total	Proportion [95% CI]
**month = 7**			
CaiXiuZhi 2018[27−4]	24	1279	0.02 [0.01; 0.03]
**month = 8**			
WuHongXing 2016[718]	63	726	0.09 [0.07; 0.11]
**month = 9**			
DaiBenNa 2020[127]	117	3119	0.04 [0.03; 0.04]
PengXiaoXue 2015[528]	37	849	0.04 [0.03; 0.06]
TaoLiYan 2020[636]	47	1857	0.03 [0.02; 0.03]
Jin−Bo Ye 2018[830−13]	60	2572	0.02 [0.02; 0.03]
Xiao GD 2019[995]	12	302	0.04 [0.02; 0.07]
**Common effect model**		**8699**	**0.03 [0.03; 0.04]**
**Random effects model**			**0.03 [0.03; 0.04]**
Heterogeneity: $I^2 = 75\%$, $\tau^2 = 0.0462$, $p < 0.01$			
**month = 10**			
ChaRiSheng 2014[47−9]	17	1910	0.01 [0.01; 0.01]
ShenYiPing 2013[53]	65	1900	0.03 [0.03; 0.04]
DuYueHe 2019[148]	29	1250	0.02 [0.02; 0.03]
DuanRong 2017[152−23]	8	706	0.01 [0.00; 0.02]
HouYuYuan 2014[231]	63	984	0.06 [0.05; 0.08]
JiangXianChen 2014[283−1]	16	1485	0.01 [0.01; 0.02]
JiangXianChen 2014[283−2]	23	1877	0.01 [0.01; 0.02]
LiMeng 2020[338−1]	7	74	0.09 [0.04; 0.19]
LiMeng 2020[338−15]	6	1863	0.00 [0.00; 0.01]
YaoJianXiang 2016[822]	59	2992	0.02 [0.02; 0.03]
ZhangHaiYan 2019[867]	145	738	0.20 [0.17; 0.23]
ZhangLi 2015[875−2]	72	656	0.11 [0.09; 0.14]
Xu H 2013[1059−2]	65	1912	0.03 [0.03; 0.04]
**Common effect model**		**18347**	**0.03 [0.03; 0.03]**
**Random effects model**			**0.03 [0.01; 0.05]**
Heterogeneity: $I^2 = 98\%$, $\tau^2 = 1.3210$, $p < 0.01$			
**month = 11**			
Ming−Wei Cai 2021[17−4]	56	1202	0.05 [0.04; 0.06]
Cai W 2017[24−6]	24	36	0.67 [0.49; 0.81]
ChaRiSheng 2014[47−6]	31	1422	0.02 [0.01; 0.03]
ChaRiSheng 2014[47−11]	61	2202	0.03 [0.02; 0.04]
ChenCaiRong 2020[62]	16	275	0.06 [0.03; 0.09]
ChenJian 2016[78]	20	1551	0.01 [0.01; 0.02]
DaiYingXue 2018[129−4]	14	1053	0.01 [0.01; 0.02]
FuJianGuang 2013[163]	276	1546	0.18 [0.16; 0.20]
GuoLi 2019[206−34]	7	40	0.17 [0.07; 0.33]
GuoLi 2019[206−52]	7	79	0.09 [0.04; 0.17]
GuoMinJian 2017[208−3]	11	39	0.28 [0.15; 0.45]
GuoMinJian 2017[208−6]	7	42	0.17 [0.07; 0.31]
HuangYanHong 2019[259−6]	21	140	0.15 [0.10; 0.22]
JiLei 2018[272−1]	7	55	0.13 [0.05; 0.24]
LiMeng 2020[338−9]	12	749	0.02 [0.01; 0.03]
LiMeng 2020[338−10]	13	754	0.02 [0.01; 0.03]
LiMeng 2020[338−12]	9	1462	0.01 [0.00; 0.01]
LiMeng 2020[338−16]	17	2026	0.01 [0.00; 0.01]
LiMeng 2020[338−17]	5	2026	0.00 [0.00; 0.01]

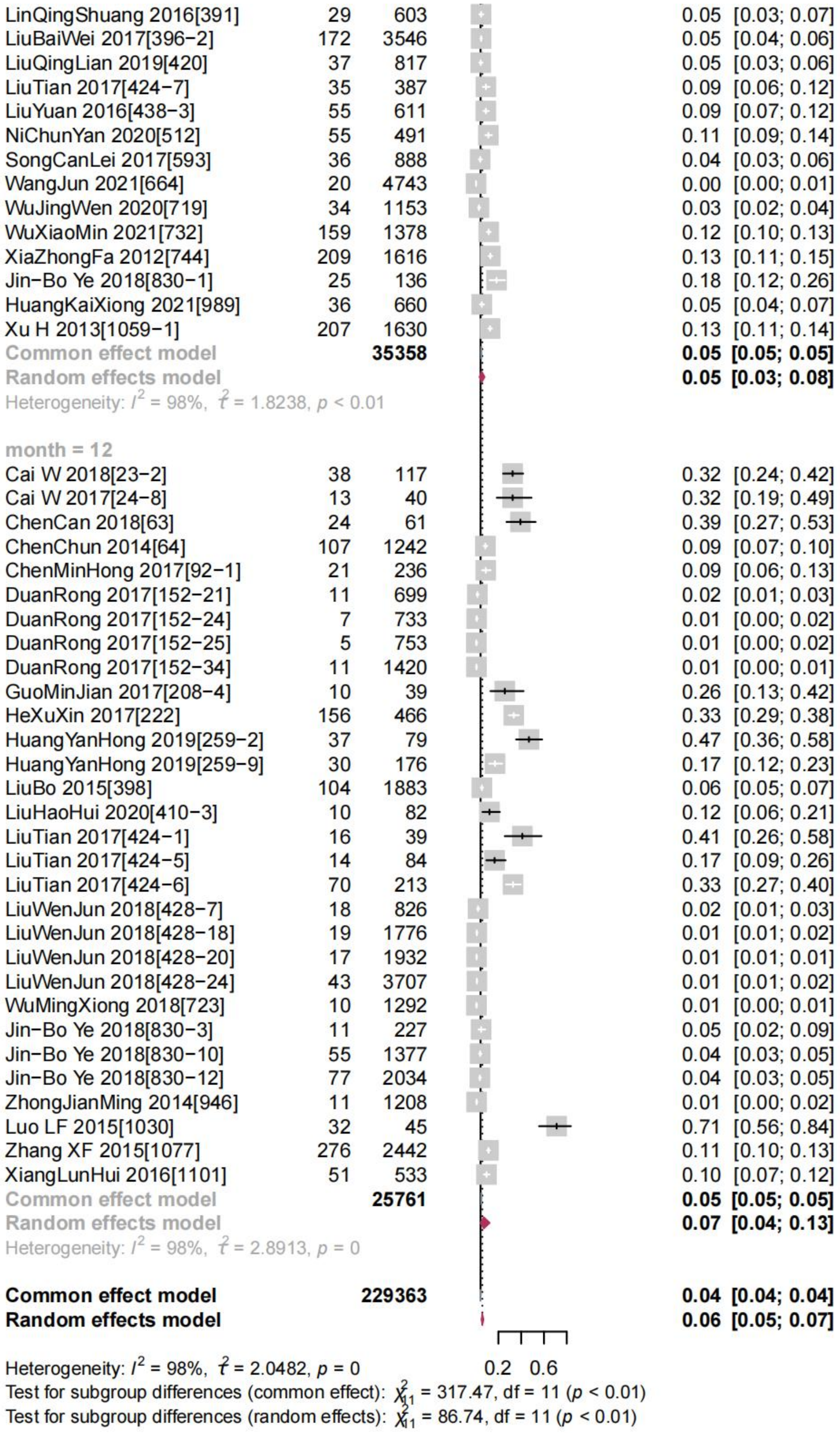
LinQingShuang 2016[391] 29 603 0.05 [0.03; 0.07]
LiuBaiWei 2017[396−2] 172 3546 0.05 [0.04; 0.06]
LiuQingLian 2019[420] 37 817 0.05 [0.03; 0.06]
LiuTian 2017[424−7] 35 387 0.09 [0.06; 0.12]
LiuYuan 2016[438−3] 55 611 0.09 [0.07; 0.12]
NiChunYan 2020[512] 55 491 0.11 [0.09; 0.14]
SongCanLei 2017[593] 36 888 0.04 [0.03; 0.06]
WangJun 2021[664] 20 4743 0.00 [0.00; 0.01]
WuJingWen 2020[719] 34 1153 0.03 [0.02; 0.04]
WuXiaoMin 2021[732] 159 1378 0.12 [0.10; 0.13]
XiaZhongFa 2012[744] 209 1616 0.13 [0.11; 0.15]
Jin−Bo Ye 2018[830−1] 25 136 0.18 [0.12; 0.26]
HuangKaiXiong 2021[989] 36 660 0.05 [0.04; 0.07]
Xu H 2013[1059−1] 207 1630 0.13 [0.11; 0.14]
Common effect model 35358 0.05 [0.05; 0.05]
Random effects model 0.05 [0.03; 0.08]
Heterogeneity: I^2 = 98%, τ^2 = 1.8238, p < 0.01
month = 12
Cai W 2018[23−2] 38 117 0.32 [0.24; 0.42]
Cai W 2017[24−8] 13 40 0.32 [0.19; 0.49]
ChenCan 2018[63] 24 61 0.39 [0.27; 0.53]
ChenChun 2014[64] 107 1242 0.09 [0.07; 0.10]
ChenMinHong 2017[92−1] 21 236 0.09 [0.06; 0.13]
DuanRong 2017[152−21] 11 699 0.02 [0.01; 0.03]
DuanRong 2017[152−24] 7 733 0.01 [0.00; 0.02]
DuanRong 2017[152−25] 5 753 0.01 [0.00; 0.02]
DuanRong 2017[152−34] 11 1420 0.01 [0.00; 0.01]
GuoMinJian 2017[208−4] 10 39 0.26 [0.13; 0.42]
HeXuXin 2017[222] 156 466 0.33 [0.29; 0.38]
HuangYanHong 2019[259−2] 37 79 0.47 [0.36; 0.58]
HuangYanHong 2019[259−9] 30 176 0.17 [0.12; 0.23]
LiuBo 2015[398] 104 1883 0.06 [0.05; 0.07]
LiuHaoHui 2020[410−3] 10 82 0.12 [0.06; 0.21]
LiuTian 2017[424−1] 16 39 0.41 [0.26; 0.58]
LiuTian 2017[424−5] 14 84 0.17 [0.09; 0.26]
LiuTian 2017[424−6] 70 213 0.33 [0.27; 0.40]
LiuWenJun 2018[428−7] 18 826 0.02 [0.01; 0.03]
LiuWenJun 2018[428−18] 19 1776 0.01 [0.01; 0.02]
LiuWenJun 2018[428−20] 17 1932 0.01 [0.01; 0.01]
LiuWenJun 2018[428−24] 43 3707 0.01 [0.01; 0.02]
WuMingXiong 2018[723] 10 1292 0.01 [0.00; 0.01]
Jin−Bo Ye 2018[830−3] 11 227 0.05 [0.02; 0.09]
Jin−Bo Ye 2018[830−10] 55 1377 0.04 [0.03; 0.05]
Jin−Bo Ye 2018[830−12] 77 2034 0.04 [0.03; 0.05]
ZhongJianMing 2014[946] 11 1208 0.01 [0.00; 0.02]
Luo LF 2015[1030] 32 45 0.71 [0.56; 0.84]
Zhang XF 2015[1077] 276 2442 0.11 [0.10; 0.13]
XiangLunHui 2016[1101] 51 533 0.10 [0.07; 0.12]
Common effect model 25761 0.05 [0.05; 0.05]
Random effects model 0.07 [0.04; 0.13]
Heterogeneity: I^2 = 98%, τ^2 = 2.8913, p = 0
Common effect model 229363 0.04 [0.04; 0.04]
Random effects model 0.06 [0.05; 0.07]
0.2 0.6
Heterogeneity: I^2 = 98%, τ^2 = 2.0482, p = 0
Test for subgroup differences (common effect): χ^2_{11} = 317.47, df = 11 (p < 0.01)
Test for subgroup differences (random effects): χ^2_{11} = 86.74, df = 11 (p < 0.01)

(b11)

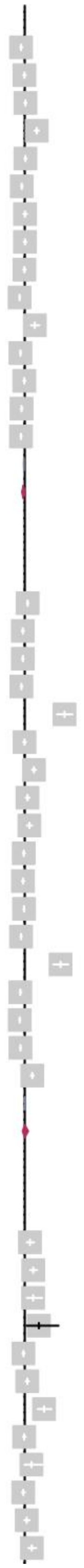

Study Secondary school	Events	Total	Attack rate	95%−CI
month = 1				
ChaRiSheng 2014[47−4]	18	1111	0.02	[0.01; 0.03]
ChaRiSheng 2014[47−7]	74	1749	0.04	[0.03; 0.05]
ChaRiSheng 2014[47−12]	139	2786	0.05	[0.04; 0.06]
ShenYuGang 2016[55]	233	1702	0.14	[0.12; 0.15]
GongLiQiang 2013[192]	139	2787	0.05	[0.04; 0.06]
GuoMinJian 2017[208−10]	48	2200	0.02	[0.02; 0.03]
LeiYongLiang 2016[311]	46	984	0.05	[0.03; 0.06]
PangZhiFeng 2017[524]	79	1696	0.05	[0.04; 0.06]
RenFuLin 2013[555]	74	1751	0.04	[0.03; 0.05]
RuiFang 2018[566−11]	15	2905	0.01	[0.00; 0.01]
XiaoSongJian 2017[745]	51	416	0.12	[0.09; 0.16]
XuHao 2016[775]	28	2385	0.01	[0.01; 0.02]
YanChaoYang 2017[792]	90	2168	0.04	[0.03; 0.05]
YangYouQing 2016[819]	48	2363	0.02	[0.02; 0.03]
YingLiHong 2016[840]	48	3000	0.02	[0.01; 0.02]
**Common effect model**		**30003**	**0.04**	**[0.04; 0.04]**
**Random effects model**			**0.03**	**[0.02; 0.05]**
Heterogeneity: $I^2 = 98\%$, $\tau^2 = 0.7223$, $p < 0.01$				
month = 2				
ChenXingHong 2012[102]	478	7113	0.07	[0.06; 0.07]
GaoShuPing 2019[183]	51	1698	0.03	[0.02; 0.04]
HuHongAn 2015[233]	105	3453	0.03	[0.02; 0.04]
Lei Ji 2011[271]	42	2210	0.02	[0.01; 0.03]
LiShiCong 2019[345]	91	259	0.35	[0.29; 0.41]
LiShiCong 2018[346]	64	1487	0.04	[0.03; 0.05]
LiuWenJun 2018[428−17]	180	1554	0.12	[0.10; 0.13]
RuiFang 2018[566−9]	78	1184	0.07	[0.05; 0.08]
SongYuFang 2019[600]	63	783	0.08	[0.06; 0.10]
TangGuoJie 2019[631]	84	2250	0.04	[0.03; 0.05]
WangJinSheng 2019[645]	56	1800	0.03	[0.02; 0.04]
WangJie 2015[659]	451	11467	0.04	[0.04; 0.04]
WangMin 2018[670−6]	47	2869	0.02	[0.01; 0.02]
WuYang 2018[733]	85	265	0.32	[0.26; 0.38]
XuJinFeng 2016[778−1]	10	933	0.01	[0.01; 0.02]
XuJinFeng 2016[778−2]	16	2500	0.01	[0.00; 0.01]
ZhouXiaoMin 2017[962]	47	3917	0.01	[0.01; 0.02]
LiuQin 2011[1088]	257	2497	0.10	[0.09; 0.12]
**Common effect model**		**48239**	**0.05**	**[0.04; 0.05]**
**Random effects model**			**0.04**	**[0.03; 0.07]**
Heterogeneity: $I^2 = 99\%$, $\tau^2 = 1.3203$, $p < 0.01$				
month = 3				
CaiXiuZhi 2018[27−2]	34	400	0.08	[0.06; 0.12]
ChenYan 2019[104]	59	536	0.11	[0.08; 0.14]
GuoLi 2019[206−57]	13	119	0.11	[0.06; 0.18]
GuoMinJian 2017[208−2]	6	39	0.15	[0.06; 0.31]
JiangYingCi 2015[284]	66	1904	0.03	[0.03; 0.04]
LinQiFeng 2018[388]	96	1494	0.06	[0.05; 0.08]
LiuBaiWei 2017[396−1]	43	222	0.19	[0.14; 0.25]
LiuWenJun 2018[428−12]	49	1213	0.04	[0.03; 0.05]
QiYing 2019[535−6]	8	83	0.10	[0.04; 0.18]
QiYing 2019[535−9]	34	1015	0.03	[0.02; 0.05]
SongHuiRong 2017[597]	34	568	0.06	[0.04; 0.08]
TianJing 2017[641]	39	392	0.10	[0.07; 0.13]

WangMin 2018[670−5]	32	2843	0.01	[0.01; 0.02]
YeXianMing 2017[832]	94	1156	0.08	[0.07; 0.10]
ZhuHaiYang 2016[971]	140	1319	0.11	[0.09; 0.12]
LiYing 2018[1097]	53	1288	0.04	[0.03; 0.05]
Common effect model		**14591**	**0.05**	**[0.05; 0.06]**
Random effects model			**0.07**	**[0.05; 0.09]**
Heterogeneity: $I^2 = 95\%$, $\tau^2 = 0.4896$, $p < 0.01$				
month = 4				
ChaRiSheng 2014[47−10]	168	2002	0.08	[0.07; 0.10]
CuiLiangLiang 2017[123−2]	14	4600	0.00	[0.00; 0.01]
GanXiangYang 2014[172]	74	2200	0.03	[0.03; 0.04]
GuoLi 2019[206−47]	9	46	0.20	[0.09; 0.34]
GuoLi 2019[206−61]	28	281	0.10	[0.07; 0.14]
GuoLi 2019[206−64]	25	498	0.05	[0.03; 0.07]
HuangBinBin 2020[245]	41	2530	0.02	[0.01; 0.02]
HuangShiTeng 2021[256]	61	1644	0.04	[0.03; 0.05]
LiMeng 2020[338−14]	85	1814	0.05	[0.04; 0.06]
LiangRiCheng 2017[376]	27	2518	0.01	[0.01; 0.02]
LiuWenJun 2018[428−22]	52	2332	0.02	[0.02; 0.03]
QiYing 2018[535−1]	173	1621	0.11	[0.09; 0.12]
QiYing 2018[535−2]	71	1621	0.04	[0.03; 0.05]
QiYing 2019[535−11]	159	1448	0.11	[0.09; 0.13]
ShiChao 2013[579]	462	2024	0.23	[0.21; 0.25]
TanDongMei 2012[627]	87	2086	0.04	[0.03; 0.05]
TianYaLin 2021[642]	38	546	0.07	[0.05; 0.09]
ZhaoWeiQin 2020[923]	128	4927	0.03	[0.02; 0.03]
Common effect model		**34738**	**0.05**	**[0.05; 0.05]**
Random effects model			**0.05**	**[0.03; 0.07]**
Heterogeneity: $I^2 = 99\%$, $\tau^2 = 1.1011$, $p < 0.01$				
month = 5				
LiuWenJun 2018[428−15]	17	1328	0.01	[0.01; 0.02]
QinLianYang 2021[545]	38	1697	0.02	[0.02; 0.03]
RuiFang 2018[566−7]	54	908	0.06	[0.04; 0.08]
WangXiaoDong 2017[688]	69	2708	0.03	[0.02; 0.03]
HuangJiaCheng 2021[944]	48	5682	0.01	[0.01; 0.01]
Common effect model		**12323**	**0.02**	**[0.02; 0.02]**
Random effects model			**0.02**	**[0.01; 0.04]**
Heterogeneity: $I^2 = 96\%$, $\tau^2 = 0.4467$, $p < 0.01$				
month = 6				
GaoZhiYong 2017[187−2]	10	46	0.22	[0.11; 0.36]
HeHanZhen 2014[224]	76	578	0.13	[0.11; 0.16]
LiuCaiXia 2019[399−3]	20	208	0.10	[0.06; 0.14]
LiuHaoHui 2020[410−9]	17	2191	0.01	[0.00; 0.01]
MoGuiQiong 2016[504]	26	843	0.03	[0.02; 0.04]
RenYuHua 2016[562]	110	1916	0.06	[0.05; 0.07]
Common effect model		**5782**	**0.04**	**[0.04; 0.05]**
Random effects model			**0.06**	**[0.02; 0.13]**
Heterogeneity: $I^2 = 97\%$, $\tau^2 = 1.2539$, $p < 0.01$				
month = 7				
GuoMinJian 2017[208−9]	16	1488	0.01	[0.01; 0.02]
month = 9				
ChaRiSheng 2014[47−8]	47	1858	0.03	[0.02; 0.03]
LiBing 2019[315]	67	2354	0.03	[0.02; 0.04]
LiCaiYun 2012[318]	147	940	0.16	[0.13; 0.18]

Study	Events	Total	Proportion	95% CI
QiuHaiYan 2013[551]	76	5965	0.01	[0.01; 0.02]
SunLiMei 2012[612]	108	5854	0.02	[0.02; 0.02]
XieBin 2020[758]	65	2036	0.03	[0.02; 0.04]
**Common effect model**		**19007**	**0.03**	**[0.02; 0.03]**
**Random effects model**			**0.03**	**[0.02; 0.06]**
Heterogeneity: $I^2 = 99\%$, $\tau^2 = 0.7048$, $p < 0.01$				
**month = 10**				
DuanRong 2017[152−31]	68	1036	0.07	[0.05; 0.08]
HuangYanHong 2019[259−5]	24	120	0.20	[0.13; 0.28]
LiJun 2012[334]	1252	2877	0.44	[0.42; 0.45]
LiuWenJun 2018[428−10]	31	951	0.03	[0.02; 0.05]
YangCaiBin 2018[796]	42	2159	0.02	[0.01; 0.03]
ZhangLi 2015[875−1]	33	474	0.07	[0.05; 0.10]
ZouYong 2011[1103]	39	2672	0.01	[0.01; 0.02]
**Common effect model**		**10289**	**0.14**	**[0.14; 0.15]**
**Random effects model**			**0.07**	**[0.03; 0.16]**
Heterogeneity: $I^2 = 100\%$, $\tau^2 = 1.6999$, $p = 0$				
**month = 11**				
BaiAiLi 2020[3]	20	738	0.03	[0.02; 0.04]
CaiJian 2013[14−1]	53	2219	0.02	[0.02; 0.03]
ChenJian 2017[80]	69	6323	0.01	[0.01; 0.01]
DuanRong 2017[152−28]	14	927	0.02	[0.01; 0.03]
LiBo 2013[316]	120	2303	0.05	[0.04; 0.06]
LiMeng 2020[338−11]	19	1168	0.02	[0.01; 0.03]
LiuHongLian 2018[411]	19	1119	0.02	[0.01; 0.03]
LiuXiuMei 2017[433]	65	1134	0.06	[0.04; 0.07]
XuJun 2018[779]	75	2679	0.03	[0.02; 0.03]
ZhanYueWang 2019[860]	38	2311	0.02	[0.01; 0.02]
ZhaoMeiLing 2019[918−2]	39	3305	0.01	[0.01; 0.02]
**Common effect model**		**24226**	**0.02**	**[0.02; 0.02]**
**Random effects model**			**0.02**	**[0.02; 0.03]**
Heterogeneity: $I^2 = 95\%$, $\tau^2 = 0.2863$, $p < 0.01$				
**month = 12**				
ChenBinBin 2017[60−2]	161	2122	0.08	[0.06; 0.09]
GaoZhiYong 2017[187−6]	25	953	0.03	[0.02; 0.04]
JiRuPing 2020[274]	119	2030	0.06	[0.05; 0.07]
LiuShiKe 2012[421]	306	1539	0.20	[0.18; 0.22]
LiuTian 2017[424−3]	32	44	0.73	[0.57; 0.85]
NiChaoRong 2019[511]	28	2158	0.01	[0.01; 0.02]
WuQinDi 2018[725]	24	1578	0.02	[0.01; 0.02]
XieHuaPing 2010[752]	55	1638	0.03	[0.03; 0.04]
Jin−Bo Ye 2018[830−8]	49	760	0.06	[0.05; 0.08]
ZhouYin 2016[964]	12	1061	0.01	[0.01; 0.02]
Guo XH 2018[1000]	61	1606	0.04	[0.03; 0.05]
Shi C 2016[1046]	57	1966	0.03	[0.02; 0.04]
**Common effect model**		**17455**	**0.05**	**[0.05; 0.06]**
**Random effects model**			**0.05**	**[0.02; 0.11]**
Heterogeneity: $I^2 = 99\%$, $\tau^2 = 1.9770$, $p < 0.01$				
**Common effect model**		**218141**	**0.04**	**[0.04; 0.05]**
**Random effects model**			**0.04**	**[0.03; 0.05]**

Axis: 0.2 0.6

Heterogeneity: $I^2 = 99\%$, $\tau^2 = 1.1505$, $p = 0$
Test for subgroup differences (common effect): $\chi^2_{10} = 2729.31$, df = 10 ($p = 0$)
Test for subgroup differences (random effects): $\chi^2_{10} = 54.14$, df = 10 ($p < 0.01$)

(b12)

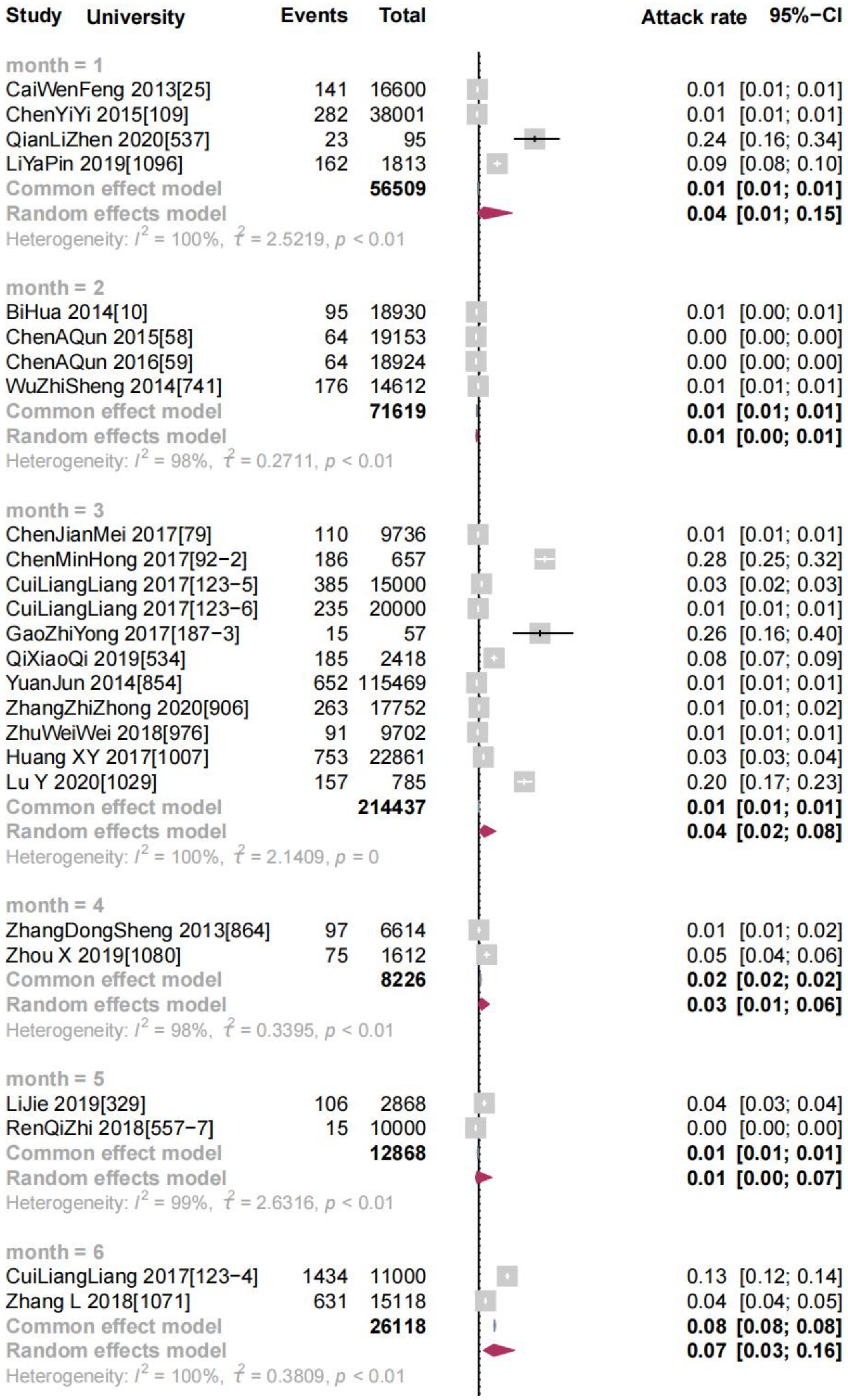
Study University Events Total Attack rate 95%−CI
month = 1
CaiWenFeng 2013[25] 141 16600 0.01 [0.01; 0.01]
ChenYiYi 2015[109] 282 38001 0.01 [0.01; 0.01]
QianLiZhen 2020[537] 23 95 0.24 [0.16; 0.34]
LiYaPin 2019[1096] 162 1813 0.09 [0.08; 0.10]
Common effect model 56509 0.01 [0.01; 0.01]
Random effects model 0.04 [0.01; 0.15]
Heterogeneity: I^2 = 100%, τ^2 = 2.5219, p < 0.01
month = 2
BiHua 2014[10] 95 18930 0.01 [0.00; 0.01]
ChenAQun 2015[58] 64 19153 0.00 [0.00; 0.00]
ChenAQun 2016[59] 64 18924 0.00 [0.00; 0.00]
WuZhiSheng 2014[741] 176 14612 0.01 [0.01; 0.01]
Common effect model 71619 0.01 [0.01; 0.01]
Random effects model 0.01 [0.00; 0.01]
Heterogeneity: I^2 = 98%, τ^2 = 0.2711, p < 0.01
month = 3
ChenJianMei 2017[79] 110 9736 0.01 [0.01; 0.01]
ChenMinHong 2017[92−2] 186 657 0.28 [0.25; 0.32]
CuiLiangLiang 2017[123−5] 385 15000 0.03 [0.02; 0.03]
CuiLiangLiang 2017[123−6] 235 20000 0.01 [0.01; 0.01]
GaoZhiYong 2017[187−3] 15 57 0.26 [0.16; 0.40]
QiXiaoQi 2019[534] 185 2418 0.08 [0.07; 0.09]
YuanJun 2014[854] 652 115469 0.01 [0.01; 0.01]
ZhangZhiZhong 2020[906] 263 17752 0.01 [0.01; 0.02]
ZhuWeiWei 2018[976] 91 9702 0.01 [0.01; 0.01]
Huang XY 2017[1007] 753 22861 0.03 [0.03; 0.04]
Lu Y 2020[1029] 157 785 0.20 [0.17; 0.23]
Common effect model 214437 0.01 [0.01; 0.01]
Random effects model 0.04 [0.02; 0.08]
Heterogeneity: I^2 = 100%, τ^2 = 2.1409, p = 0
month = 4
ZhangDongSheng 2013[864] 97 6614 0.01 [0.01; 0.02]
Zhou X 2019[1080] 75 1612 0.05 [0.04; 0.06]
Common effect model 8226 0.02 [0.02; 0.02]
Random effects model 0.03 [0.01; 0.06]
Heterogeneity: I^2 = 98%, τ^2 = 0.3395, p < 0.01
month = 5
LiJie 2019[329] 106 2868 0.04 [0.03; 0.04]
RenQiZhi 2018[557−7] 15 10000 0.00 [0.00; 0.00]
Common effect model 12868 0.01 [0.01; 0.01]
Random effects model 0.01 [0.00; 0.07]
Heterogeneity: I^2 = 99%, τ^2 = 2.6316, p < 0.01
month = 6
CuiLiangLiang 2017[123−4] 1434 11000 0.13 [0.12; 0.14]
Zhang L 2018[1071] 631 15118 0.04 [0.04; 0.05]
Common effect model 26118 0.08 [0.08; 0.08]
Random effects model 0.07 [0.03; 0.16]
Heterogeneity: I^2 = 100%, τ^2 = 0.3809, p < 0.01

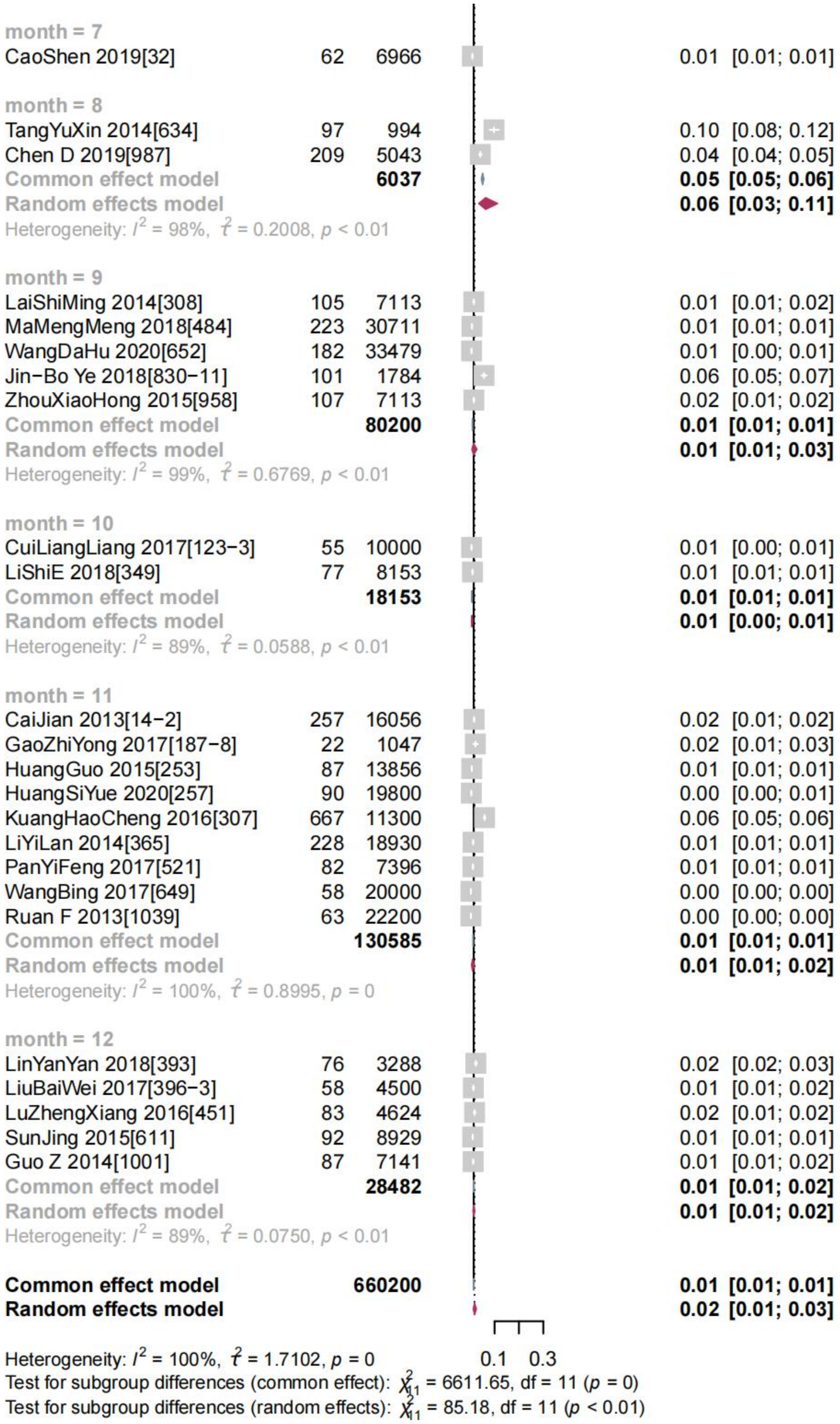
month = 7
CaoShen 2019[32] 62 6966 0.01 [0.01; 0.01]
month = 8
TangYuXin 2014[634] 97 994 0.10 [0.08; 0.12]
Chen D 2019[987] 209 5043 0.04 [0.04; 0.05]
Common effect model 6037 0.05 [0.05; 0.06]
Random effects model 0.06 [0.03; 0.11]
Heterogeneity: I^2 = 98%, τ^2 = 0.2008, p < 0.01
month = 9
LaiShiMing 2014[308] 105 7113 0.01 [0.01; 0.02]
MaMengMeng 2018[484] 223 30711 0.01 [0.01; 0.01]
WangDaHu 2020[652] 182 33479 0.01 [0.00; 0.01]
Jin−Bo Ye 2018[830−11] 101 1784 0.06 [0.05; 0.07]
ZhouXiaoHong 2015[958] 107 7113 0.02 [0.01; 0.02]
Common effect model 80200 0.01 [0.01; 0.01]
Random effects model 0.01 [0.01; 0.03]
Heterogeneity: I^2 = 99%, τ^2 = 0.6769, p < 0.01
month = 10
CuiLiangLiang 2017[123−3] 55 10000 0.01 [0.00; 0.01]
LiShiE 2018[349] 77 8153 0.01 [0.01; 0.01]
Common effect model 18153 0.01 [0.01; 0.01]
Random effects model 0.01 [0.00; 0.01]
Heterogeneity: I^2 = 89%, τ^2 = 0.0588, p < 0.01
month = 11
CaiJian 2013[14−2] 257 16056 0.02 [0.01; 0.02]
GaoZhiYong 2017[187−8] 22 1047 0.02 [0.01; 0.03]
HuangGuo 2015[253] 87 13856 0.01 [0.01; 0.01]
HuangSiYue 2020[257] 90 19800 0.00 [0.00; 0.01]
KuangHaoCheng 2016[307] 667 11300 0.06 [0.05; 0.06]
LiYiLan 2014[365] 228 18930 0.01 [0.01; 0.01]
PanYiFeng 2017[521] 82 7396 0.01 [0.01; 0.01]
WangBing 2017[649] 58 20000 0.00 [0.00; 0.00]
Ruan F 2013[1039] 63 22200 0.00 [0.00; 0.00]
Common effect model 130585 0.01 [0.01; 0.01]
Random effects model 0.01 [0.01; 0.02]
Heterogeneity: I^2 = 100%, τ^2 = 0.8995, p = 0
month = 12
LinYanYan 2018[393] 76 3288 0.02 [0.02; 0.03]
LiuBaiWei 2017[396−3] 58 4500 0.01 [0.01; 0.02]
LuZhengXiang 2016[451] 83 4624 0.02 [0.01; 0.02]
SunJing 2015[611] 92 8929 0.01 [0.01; 0.01]
Guo Z 2014[1001] 87 7141 0.01 [0.01; 0.02]
Common effect model 28482 0.01 [0.01; 0.02]
Random effects model 0.01 [0.01; 0.02]
Heterogeneity: I^2 = 89%, τ^2 = 0.0750, p < 0.01
Common effect model 660200 0.01 [0.01; 0.01]
Random effects model 0.02 [0.01; 0.03]
0.1 0.3
Heterogeneity: I^2 = 100%, τ^2 = 1.7102, p = 0
Test for subgroup differences (common effect): χ^2_{11} = 6611.65, df = 11 (p = 0)
Test for subgroup differences (random effects): χ^2_{11} = 85.18, df = 11 (p < 0.01)

(b13)

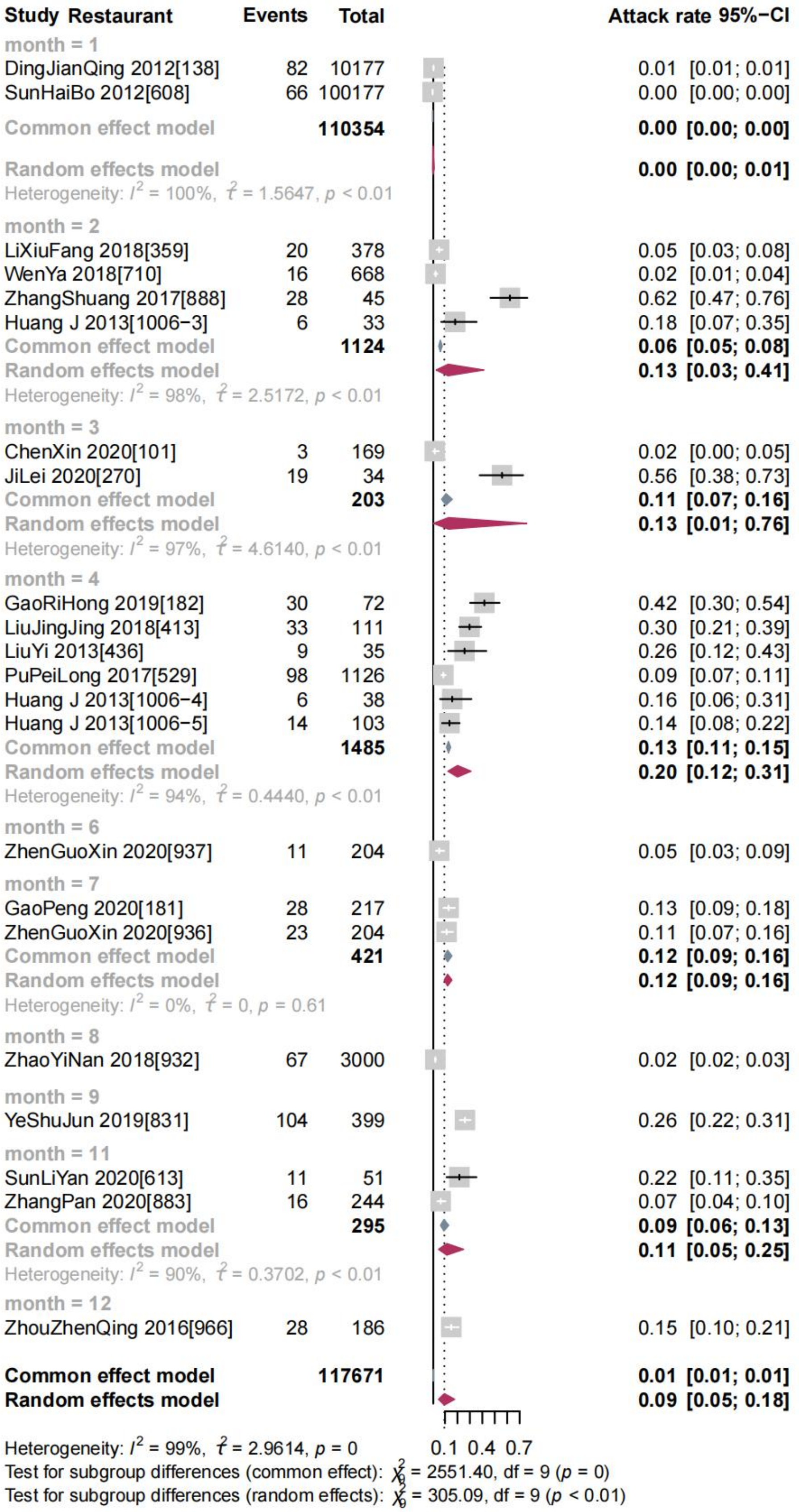
Study Restaurant Events Total Attack rate 95%−CI
month = 1
DingJianQing 2012[138] 82 10177 0.01 [0.01; 0.01]
SunHaiBo 2012[608] 66 100177 0.00 [0.00; 0.00]
Common effect model 110354 0.00 [0.00; 0.00]
Random effects model 0.00 [0.00; 0.01]
Heterogeneity: I^2 = 100%, τ^2 = 1.5647, p < 0.01
month = 2
LiXiuFang 2018[359] 20 378 0.05 [0.03; 0.08]
WenYa 2018[710] 16 668 0.02 [0.01; 0.04]
ZhangShuang 2017[888] 28 45 0.62 [0.47; 0.76]
Huang J 2013[1006−3] 6 33 0.18 [0.07; 0.35]
Common effect model 1124 0.06 [0.05; 0.08]
Random effects model 0.13 [0.03; 0.41]
Heterogeneity: I^2 = 98%, τ^2 = 2.5172, p < 0.01
month = 3
ChenXin 2020[101] 3 169 0.02 [0.00; 0.05]
JiLei 2020[270] 19 34 0.56 [0.38; 0.73]
Common effect model 203 0.11 [0.07; 0.16]
Random effects model 0.13 [0.01; 0.76]
Heterogeneity: I^2 = 97%, τ^2 = 4.6140, p < 0.01
month = 4
GaoRiHong 2019[182] 30 72 0.42 [0.30; 0.54]
LiuJingJing 2018[413] 33 111 0.30 [0.21; 0.39]
LiuYi 2013[436] 9 35 0.26 [0.12; 0.43]
PuPeiLong 2017[529] 98 1126 0.09 [0.07; 0.11]
Huang J 2013[1006−4] 6 38 0.16 [0.06; 0.31]
Huang J 2013[1006−5] 14 103 0.14 [0.08; 0.22]
Common effect model 1485 0.13 [0.11; 0.15]
Random effects model 0.20 [0.12; 0.31]
Heterogeneity: I^2 = 94%, τ^2 = 0.4440, p < 0.01
month = 6
ZhenGuoXin 2020[937] 11 204 0.05 [0.03; 0.09]
month = 7
GaoPeng 2020[181] 28 217 0.13 [0.09; 0.18]
ZhenGuoXin 2020[936] 23 204 0.11 [0.07; 0.16]
Common effect model 421 0.12 [0.09; 0.16]
Random effects model 0.12 [0.09; 0.16]
Heterogeneity: I^2 = 0%, τ^2 = 0, p = 0.61
month = 8
ZhaoYiNan 2018[932] 67 3000 0.02 [0.02; 0.03]
month = 9
YeShuJun 2019[831] 104 399 0.26 [0.22; 0.31]
month = 11
SunLiYan 2020[613] 11 51 0.22 [0.11; 0.35]
ZhangPan 2020[883] 16 244 0.07 [0.04; 0.10]
Common effect model 295 0.09 [0.06; 0.13]
Random effects model 0.11 [0.05; 0.25]
Heterogeneity: I^2 = 90%, τ^2 = 0.3702, p < 0.01
month = 12
ZhouZhenQing 2016[966] 28 186 0.15 [0.10; 0.21]
Common effect model 117671 0.01 [0.01; 0.01]
Random effects model 0.09 [0.05; 0.18]
0.1 0.4 0.7
Heterogeneity: I^2 = 99%, τ^2 = 2.9614, p = 0
Test for subgroup differences (common effect): χ^2_9 = 2551.40, df = 9 (p = 0)
Test for subgroup differences (random effects): χ^2_9 = 305.09, df = 9 (p < 0.01)

(b14)

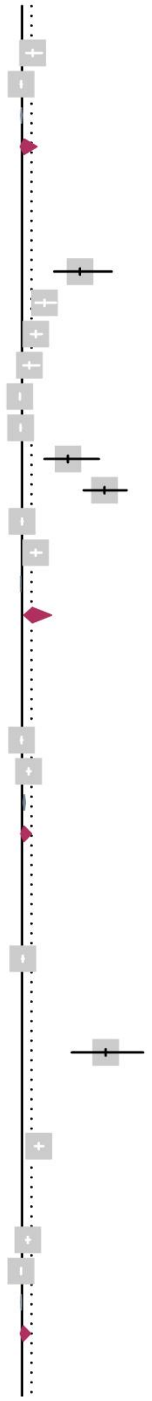

Study Community	Events	Total	Attack rate	95%−CI
month = 1				
LuoTengXian 2018[475]	13	200	0.06	[0.04; 0.11]
ZhaoQi 2021[920]	53	4760	0.01	[0.01; 0.01]
Common effect model		**4960**	**0.01**	**[0.01; 0.02]**
Random effects model			**0.03**	**[0.01; 0.08]**
Heterogeneity: $I^2 = 97\%$, $\tau^2 = 0.7537$, $p < 0.01$				
month = 2				
ChenHeJuan 2018[73]	14	49	0.29	[0.17; 0.43]
DongShengCao 2017[146]	24	200	0.12	[0.08; 0.17]
MengJian 2018[495]	37	465	0.08	[0.06; 0.11]
SongCanLei 2013[592−2]	8	161	0.05	[0.02; 0.10]
SunZhou 2016[625]	230	45075	0.01	[0.00; 0.01]
WangJinSheng 2016[646]	99	11594	0.01	[0.01; 0.01]
YangZhiWen 2013[820]	11	48	0.23	[0.12; 0.37]
ZhangHuiLing 2018[871]	40	100	0.40	[0.30; 0.50]
ZhangMeiMei 2013[880]	126	8154	0.02	[0.01; 0.02]
XuHaoBin 2015[1091]	39	496	0.08	[0.06; 0.11]
Common effect model		**66342**	**0.01**	**[0.01; 0.01]**
Random effects model			**0.06**	**[0.03; 0.15]**
Heterogeneity: $I^2 = 99\%$, $\tau^2 = 2.3930$, $p < 0.01$				
month = 3				
LuXiuZhi 2018[463]	47	3600	0.01	[0.01; 0.02]
XiaGuangHui 2019[742]	106	2300	0.05	[0.04; 0.06]
Common effect model		**5900**	**0.03**	**[0.02; 0.03]**
Random effects model			**0.02**	**[0.01; 0.06]**
Heterogeneity: $I^2 = 98\%$, $\tau^2 = 0.4072$, $p < 0.01$				
month = 4				
XieBin 2018[757]	95	5000	0.02	[0.02; 0.02]
month = 5				
ShenJingQiang 2020[993]	15	37	0.41	[0.25; 0.58]
month = 6				
MaTao 2015[486]	84	901	0.09	[0.08; 0.11]
month = 7				
ChenQian 2014[95]	78	1844	0.04	[0.03; 0.05]
DengXingChao 2019[133]	439	42403	0.01	[0.01; 0.01]
Common effect model		**44247**	**0.01**	**[0.01; 0.01]**
Random effects model			**0.02**	**[0.01; 0.05]**
Heterogeneity: $I^2 = 99\%$, $\tau^2 = 0.5072$, $p < 0.01$				

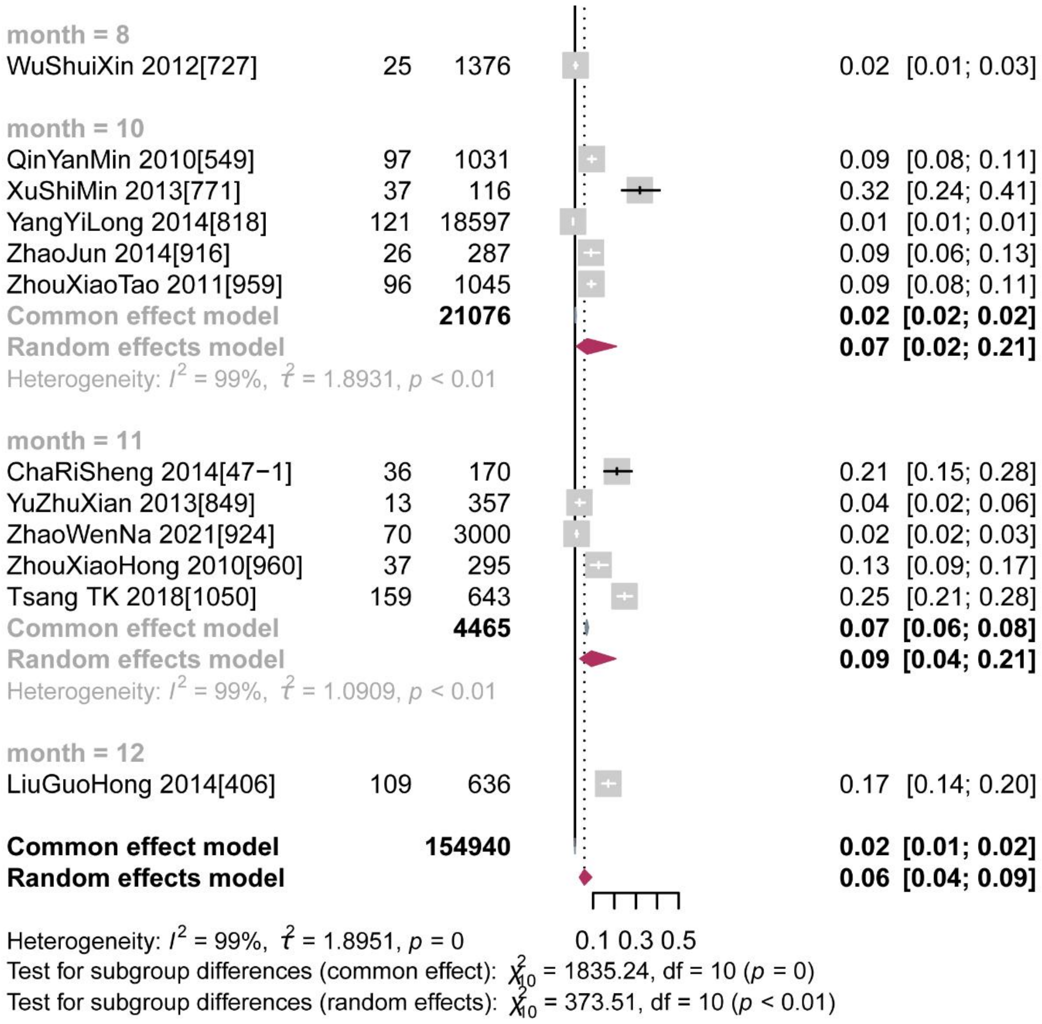
month = 8
WuShuiXin 2012[727] 25 1376 0.02 [0.01; 0.03]
month = 10
QinYanMin 2010[549] 97 1031 0.09 [0.08; 0.11]
XuShiMin 2013[771] 37 116 0.32 [0.24; 0.41]
YangYiLong 2014[818] 121 18597 0.01 [0.01; 0.01]
ZhaoJun 2014[916] 26 287 0.09 [0.06; 0.13]
ZhouXiaoTao 2011[959] 96 1045 0.09 [0.08; 0.11]
Common effect model 21076 0.02 [0.02; 0.02]
Random effects model 0.07 [0.02; 0.21]
Heterogeneity: I^2 = 99%, τ^2 = 1.8931, p < 0.01
month = 11
ChaRiSheng 2014[47−1] 36 170 0.21 [0.15; 0.28]
YuZhuXian 2013[849] 13 357 0.04 [0.02; 0.06]
ZhaoWenNa 2021[924] 70 3000 0.02 [0.02; 0.03]
ZhouXiaoHong 2010[960] 37 295 0.13 [0.09; 0.17]
Tsang TK 2018[1050] 159 643 0.25 [0.21; 0.28]
Common effect model 4465 0.07 [0.06; 0.08]
Random effects model 0.09 [0.04; 0.21]
Heterogeneity: I^2 = 99%, τ^2 = 1.0909, p < 0.01
month = 12
LiuGuoHong 2014[406] 109 636 0.17 [0.14; 0.20]
Common effect model 154940 0.02 [0.01; 0.02]
Random effects model 0.06 [0.04; 0.09]
0.1 0.3 0.5
Heterogeneity: I^2 = 99%, τ^2 = 1.8951, p = 0
Test for subgroup differences (common effect): χ^2_{10} = 1835.24, df = 10 (p = 0)
Test for subgroup differences (random effects): χ^2_{10} = 373.51, df = 10 (p < 0.01)

(b15)

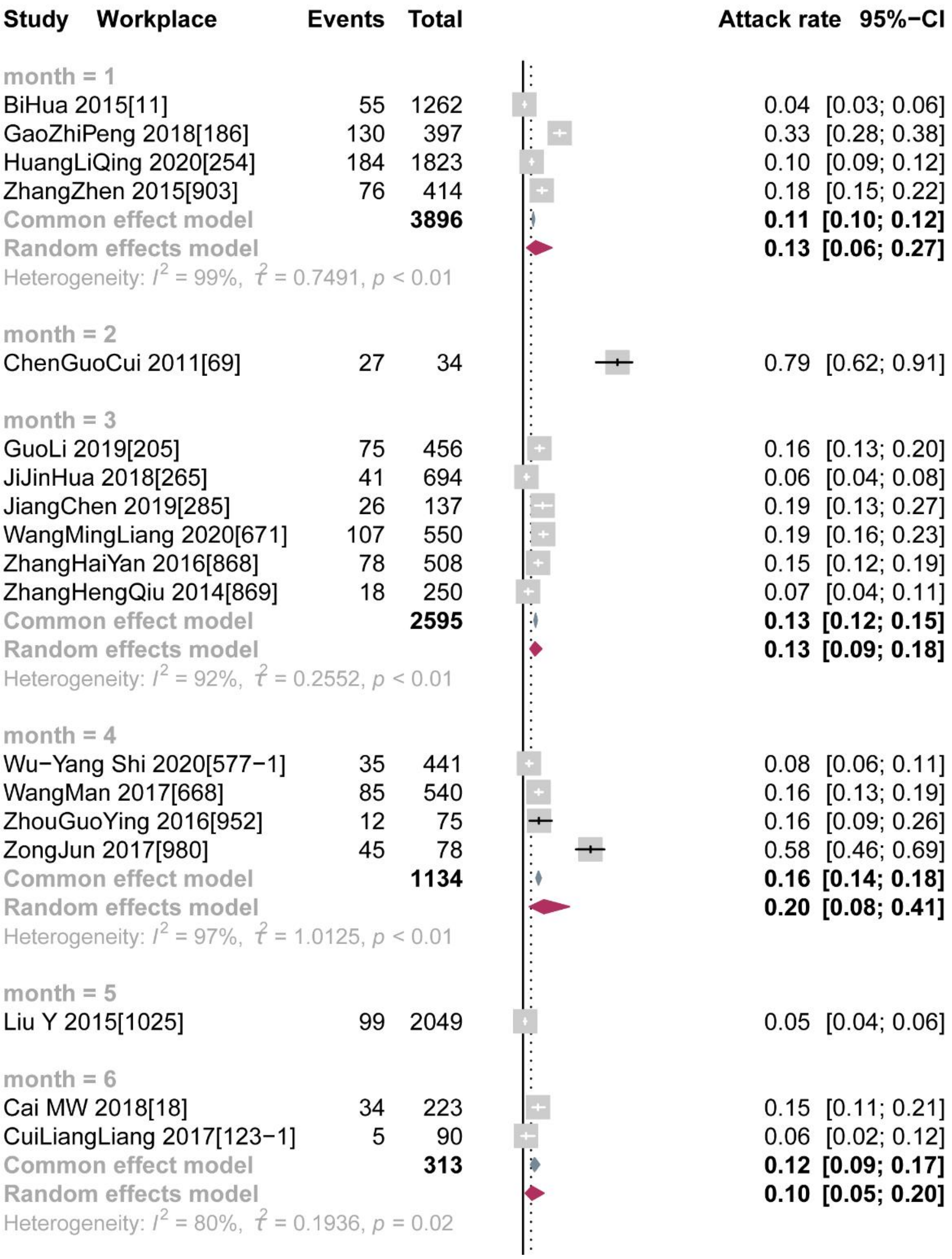

Study Workplace
Events
Total
Attack rate 95%−CI
month = 1
BiHua 2015[11] 55 1262 0.04 [0.03; 0.06]
GaoZhiPeng 2018[186] 130 397 0.33 [0.28; 0.38]
HuangLiQing 2020[254] 184 1823 0.10 [0.09; 0.12]
ZhangZhen 2015[903] 76 414 0.18 [0.15; 0.22]
Common effect model 3896 0.11 [0.10; 0.12]
Random effects model 0.13 [0.06; 0.27]
Heterogeneity: I^2 = 99%, τ^2 = 0.7491, p < 0.01
month = 2
ChenGuoCui 2011[69] 27 34 0.79 [0.62; 0.91]
month = 3
GuoLi 2019[205] 75 456 0.16 [0.13; 0.20]
JiJinHua 2018[265] 41 694 0.06 [0.04; 0.08]
JiangChen 2019[285] 26 137 0.19 [0.13; 0.27]
WangMingLiang 2020[671] 107 550 0.19 [0.16; 0.23]
ZhangHaiYan 2016[868] 78 508 0.15 [0.12; 0.19]
ZhangHengQiu 2014[869] 18 250 0.07 [0.04; 0.11]
Common effect model 2595 0.13 [0.12; 0.15]
Random effects model 0.13 [0.09; 0.18]
Heterogeneity: I^2 = 92%, τ^2 = 0.2552, p < 0.01
month = 4
Wu−Yang Shi 2020[577−1] 35 441 0.08 [0.06; 0.11]
WangMan 2017[668] 85 540 0.16 [0.13; 0.19]
ZhouGuoYing 2016[952] 12 75 0.16 [0.09; 0.26]
ZongJun 2017[980] 45 78 0.58 [0.46; 0.69]
Common effect model 1134 0.16 [0.14; 0.18]
Random effects model 0.20 [0.08; 0.41]
Heterogeneity: I^2 = 97%, τ^2 = 1.0125, p < 0.01
month = 5
Liu Y 2015[1025] 99 2049 0.05 [0.04; 0.06]
month = 6
Cai MW 2018[18] 34 223 0.15 [0.11; 0.21]
CuiLiangLiang 2017[123−1] 5 90 0.06 [0.02; 0.12]
Common effect model 313 0.12 [0.09; 0.17]
Random effects model 0.10 [0.05; 0.20]
Heterogeneity: I^2 = 80%, τ^2 = 0.1936, p = 0.02

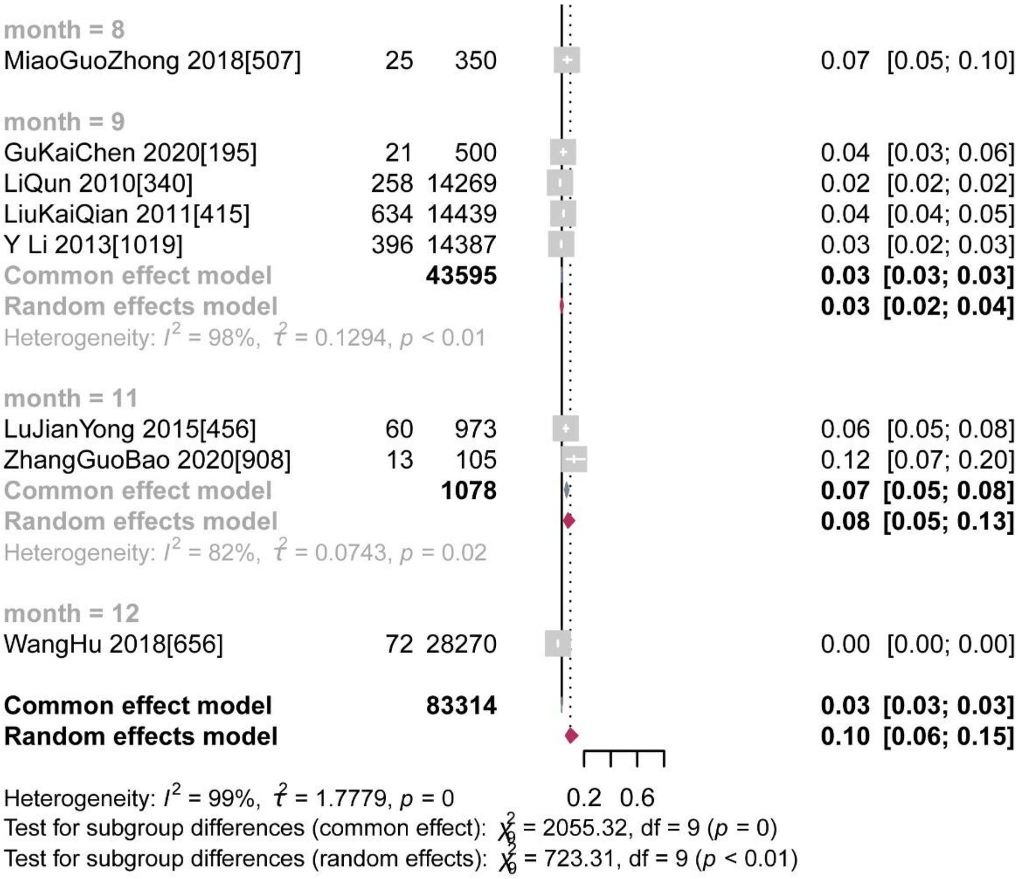
month = 8
MiaoGuoZhong 2018[507] 25 350 0.07 [0.05; 0.10]
month = 9
GuKaiChen 2020[195] 21 500 0.04 [0.03; 0.06]
LiQun 2010[340] 258 14269 0.02 [0.02; 0.02]
LiuKaiQian 2011[415] 634 14439 0.04 [0.04; 0.05]
Y Li 2013[1019] 396 14387 0.03 [0.02; 0.03]
Common effect model 43595 0.03 [0.03; 0.03]
Random effects model 0.03 [0.02; 0.04]
Heterogeneity: I^2 = 98%, τ^2 = 0.1294, p < 0.01
month = 11
LuJianYong 2015[456] 60 973 0.06 [0.05; 0.08]
ZhangGuoBao 2020[908] 13 105 0.12 [0.07; 0.20]
Common effect model 1078 0.07 [0.05; 0.08]
Random effects model 0.08 [0.05; 0.13]
Heterogeneity: I^2 = 82%, τ^2 = 0.0743, p = 0.02
month = 12
WangHu 2018[656] 72 28270 0.00 [0.00; 0.00]
Common effect model 83314 0.03 [0.03; 0.03]
Random effects model 0.10 [0.06; 0.15]
0.2 0.6
Heterogeneity: I^2 = 99%, τ^2 = 1.7779, p = 0
Test for subgroup differences (common effect): χ^2_9 = 2055.32, df = 9 (p = 0)
Test for subgroup differences (random effects): χ^2_9 = 723.31, df = 9 (p < 0.01)

(b16)

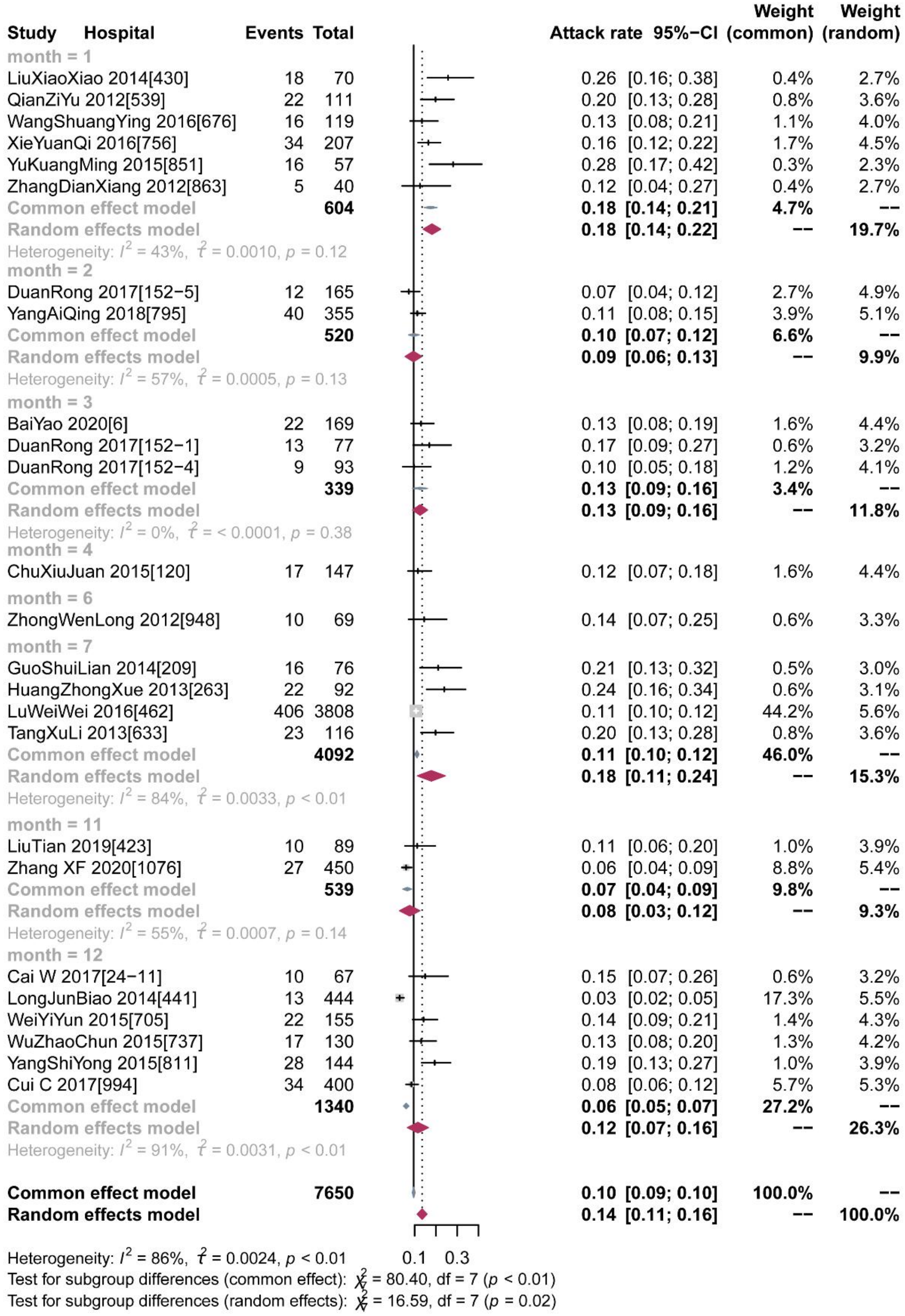

Study Hospital Events Total Attack rate 95%−CI Weight (common) Weight (random)
month = 1
LiuXiaoXiao 2014[430] 18 70 0.26 [0.16; 0.38] 0.4% 2.7%
QianZiYu 2012[539] 22 111 0.20 [0.13; 0.28] 0.8% 3.6%
WangShuangYing 2016[676] 16 119 0.13 [0.08; 0.21] 1.1% 4.0%
XieYuanQi 2016[756] 34 207 0.16 [0.12; 0.22] 1.7% 4.5%
YuKuangMing 2015[851] 16 57 0.28 [0.17; 0.42] 0.3% 2.3%
ZhangDianXiang 2012[863] 5 40 0.12 [0.04; 0.27] 0.4% 2.7%
Common effect model 604 0.18 [0.14; 0.21] 4.7% --
Random effects model 0.18 [0.14; 0.22] -- 19.7%
Heterogeneity: I^2 = 43%, τ^2 = 0.0010, p = 0.12
month = 2
DuanRong 2017[152−5] 12 165 0.07 [0.04; 0.12] 2.7% 4.9%
YangAiQing 2018[795] 40 355 0.11 [0.08; 0.15] 3.9% 5.1%
Common effect model 520 0.10 [0.07; 0.12] 6.6% --
Random effects model 0.09 [0.06; 0.13] -- 9.9%
Heterogeneity: I^2 = 57%, τ^2 = 0.0005, p = 0.13
month = 3
BaiYao 2020[6] 22 169 0.13 [0.08; 0.19] 1.6% 4.4%
DuanRong 2017[152−1] 13 77 0.17 [0.09; 0.27] 0.6% 3.2%
DuanRong 2017[152−4] 9 93 0.10 [0.05; 0.18] 1.2% 4.1%
Common effect model 339 0.13 [0.09; 0.16] 3.4% --
Random effects model 0.13 [0.09; 0.16] -- 11.8%
Heterogeneity: I^2 = 0%, τ^2 = < 0.0001, p = 0.38
month = 4
ChuXiuJuan 2015[120] 17 147 0.12 [0.07; 0.18] 1.6% 4.4%
month = 6
ZhongWenLong 2012[948] 10 69 0.14 [0.07; 0.25] 0.6% 3.3%
month = 7
GuoShuiLian 2014[209] 16 76 0.21 [0.13; 0.32] 0.5% 3.0%
HuangZhongXue 2013[263] 22 92 0.24 [0.16; 0.34] 0.6% 3.1%
LuWeiWei 2016[462] 406 3808 0.11 [0.10; 0.12] 44.2% 5.6%
TangXuLi 2013[633] 23 116 0.20 [0.13; 0.28] 0.8% 3.6%
Common effect model 4092 0.11 [0.10; 0.12] 46.0% --
Random effects model 0.18 [0.11; 0.24] -- 15.3%
Heterogeneity: I^2 = 84%, τ^2 = 0.0033, p < 0.01
month = 11
LiuTian 2019[423] 10 89 0.11 [0.06; 0.20] 1.0% 3.9%
Zhang XF 2020[1076] 27 450 0.06 [0.04; 0.09] 8.8% 5.4%
Common effect model 539 0.07 [0.04; 0.09] 9.8% --
Random effects model 0.08 [0.03; 0.12] -- 9.3%
Heterogeneity: I^2 = 55%, τ^2 = 0.0007, p = 0.14
month = 12
Cai W 2017[24−11] 10 67 0.15 [0.07; 0.26] 0.6% 3.2%
LongJunBiao 2014[441] 13 444 0.03 [0.02; 0.05] 17.3% 5.5%
WeiYiYun 2015[705] 22 155 0.14 [0.09; 0.21] 1.4% 4.3%
WuZhaoChun 2015[737] 17 130 0.13 [0.08; 0.20] 1.3% 4.2%
YangShiYong 2015[811] 28 144 0.19 [0.13; 0.27] 1.0% 3.9%
Cui C 2017[994] 34 400 0.08 [0.06; 0.12] 5.7% 5.3%
Common effect model 1340 0.06 [0.05; 0.07] 27.2% --
Random effects model 0.12 [0.07; 0.16] -- 26.3%
Heterogeneity: I^2 = 91%, τ^2 = 0.0031, p < 0.01
Common effect model 7650 0.10 [0.09; 0.10] 100.0% --
Random effects model 0.14 [0.11; 0.16] -- 100.0%
0.1 0.3
Heterogeneity: I^2 = 86%, τ^2 = 0.0024, p < 0.01
Test for subgroup differences (common effect): χ^2_7 = 80.40, df = 7 (p < 0.01)
Test for subgroup differences (random effects): χ^2_7 = 16.59, df = 7 (p = 0.02)

(b17)

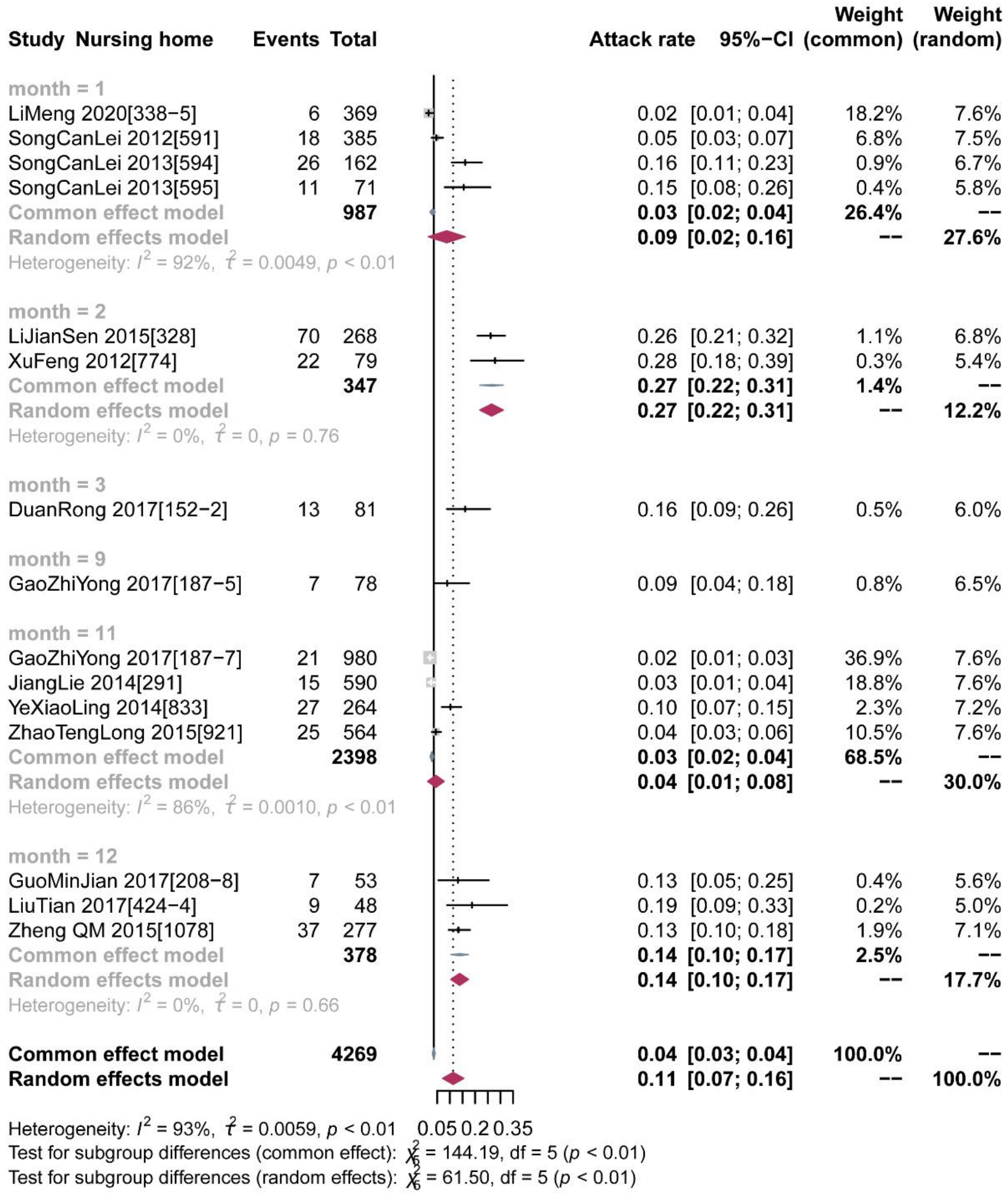
Study Nursing home Events Total
Attack rate 95%−CI Weight (common) Weight (random)
month = 1
LiMeng 2020[338−5] 6 369 0.02 [0.01; 0.04] 18.2% 7.6%
SongCanLei 2012[591] 18 385 0.05 [0.03; 0.07] 6.8% 7.5%
SongCanLei 2013[594] 26 162 0.16 [0.11; 0.23] 0.9% 6.7%
SongCanLei 2013[595] 11 71 0.15 [0.08; 0.26] 0.4% 5.8%
Common effect model 987 0.03 [0.02; 0.04] 26.4% --
Random effects model 0.09 [0.02; 0.16] -- 27.6%
Heterogeneity: I^2 = 92%, τ^2 = 0.0049, p < 0.01
month = 2
LiJianSen 2015[328] 70 268 0.26 [0.21; 0.32] 1.1% 6.8%
XuFeng 2012[774] 22 79 0.28 [0.18; 0.39] 0.3% 5.4%
Common effect model 347 0.27 [0.22; 0.31] 1.4% --
Random effects model 0.27 [0.22; 0.31] -- 12.2%
Heterogeneity: I^2 = 0%, τ^2 = 0, p = 0.76
month = 3
DuanRong 2017[152−2] 13 81 0.16 [0.09; 0.26] 0.5% 6.0%
month = 9
GaoZhiYong 2017[187−5] 7 78 0.09 [0.04; 0.18] 0.8% 6.5%
month = 11
GaoZhiYong 2017[187−7] 21 980 0.02 [0.01; 0.03] 36.9% 7.6%
JiangLie 2014[291] 15 590 0.03 [0.01; 0.04] 18.8% 7.6%
YeXiaoLing 2014[833] 27 264 0.10 [0.07; 0.15] 2.3% 7.2%
ZhaoTengLong 2015[921] 25 564 0.04 [0.03; 0.06] 10.5% 7.6%
Common effect model 2398 0.03 [0.02; 0.04] 68.5% --
Random effects model 0.04 [0.01; 0.08] -- 30.0%
Heterogeneity: I^2 = 86%, τ^2 = 0.0010, p < 0.01
month = 12
GuoMinJian 2017[208−8] 7 53 0.13 [0.05; 0.25] 0.4% 5.6%
LiuTian 2017[424−4] 9 48 0.19 [0.09; 0.33] 0.2% 5.0%
Zheng QM 2015[1078] 37 277 0.13 [0.10; 0.18] 1.9% 7.1%
Common effect model 378 0.14 [0.10; 0.17] 2.5% --
Random effects model 0.14 [0.10; 0.17] -- 17.7%
Heterogeneity: I^2 = 0%, τ^2 = 0, p = 0.66
Common effect model 4269 0.04 [0.03; 0.04] 100.0% --
Random effects model 0.11 [0.07; 0.16] -- 100.0%
0.05 0.2 0.35
Heterogeneity: I^2 = 93%, τ^2 = 0.0059, p < 0.01
Test for subgroup differences (common effect): χ^2_5 = 144.19, df = 5 (p < 0.01)
Test for subgroup differences (random effects): χ^2_5 = 61.50, df = 5 (p < 0.01)

(b18)

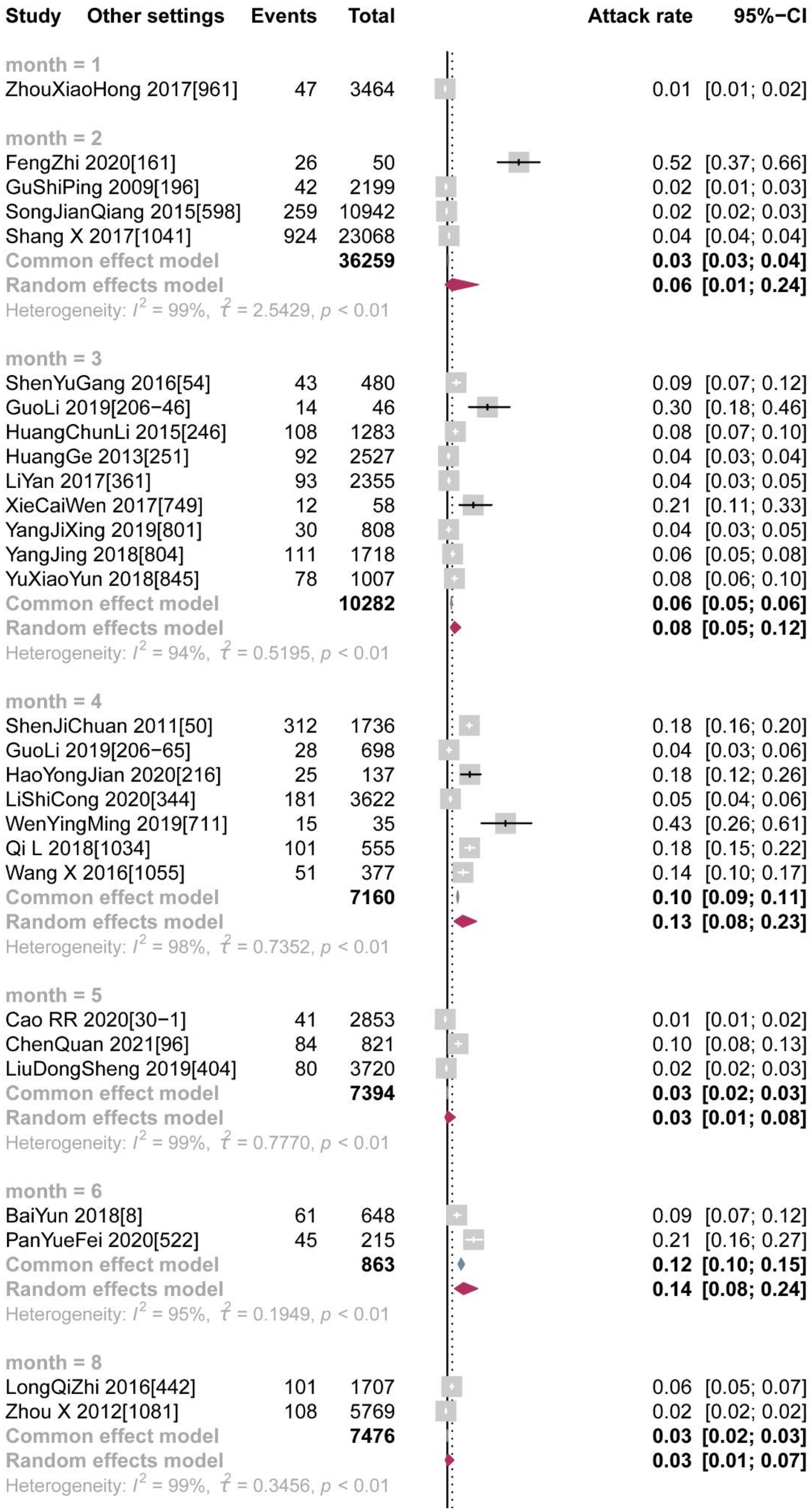

Study Other settings Events Total Attack rate 95%−CI
month = 1
ZhouXiaoHong 2017[961] 47 3464 0.01 [0.01; 0.02]
month = 2
FengZhi 2020[161] 26 50 0.52 [0.37; 0.66]
GuShiPing 2009[196] 42 2199 0.02 [0.01; 0.03]
SongJianQiang 2015[598] 259 10942 0.02 [0.02; 0.03]
Shang X 2017[1041] 924 23068 0.04 [0.04; 0.04]
Common effect model 36259 0.03 [0.03; 0.04]
Random effects model 0.06 [0.01; 0.24]
Heterogeneity: I^2 = 99%, τ^2 = 2.5429, p < 0.01
month = 3
ShenYuGang 2016[54] 43 480 0.09 [0.07; 0.12]
GuoLi 2019[206−46] 14 46 0.30 [0.18; 0.46]
HuangChunLi 2015[246] 108 1283 0.08 [0.07; 0.10]
HuangGe 2013[251] 92 2527 0.04 [0.03; 0.04]
LiYan 2017[361] 93 2355 0.04 [0.03; 0.05]
XieCaiWen 2017[749] 12 58 0.21 [0.11; 0.33]
YangJiXing 2019[801] 30 808 0.04 [0.03; 0.05]
YangJing 2018[804] 111 1718 0.06 [0.05; 0.08]
YuXiaoYun 2018[845] 78 1007 0.08 [0.06; 0.10]
Common effect model 10282 0.06 [0.05; 0.06]
Random effects model 0.08 [0.05; 0.12]
Heterogeneity: I^2 = 94%, τ^2 = 0.5195, p < 0.01
month = 4
ShenJiChuan 2011[50] 312 1736 0.18 [0.16; 0.20]
GuoLi 2019[206−65] 28 698 0.04 [0.03; 0.06]
HaoYongJian 2020[216] 25 137 0.18 [0.12; 0.26]
LiShiCong 2020[344] 181 3622 0.05 [0.04; 0.06]
WenYingMing 2019[711] 15 35 0.43 [0.26; 0.61]
Qi L 2018[1034] 101 555 0.18 [0.15; 0.22]
Wang X 2016[1055] 51 377 0.14 [0.10; 0.17]
Common effect model 7160 0.10 [0.09; 0.11]
Random effects model 0.13 [0.08; 0.23]
Heterogeneity: I^2 = 98%, τ^2 = 0.7352, p < 0.01
month = 5
Cao RR 2020[30−1] 41 2853 0.01 [0.01; 0.02]
ChenQuan 2021[96] 84 821 0.10 [0.08; 0.13]
LiuDongSheng 2019[404] 80 3720 0.02 [0.02; 0.03]
Common effect model 7394 0.03 [0.02; 0.03]
Random effects model 0.03 [0.01; 0.08]
Heterogeneity: I^2 = 99%, τ^2 = 0.7770, p < 0.01
month = 6
BaiYun 2018[8] 61 648 0.09 [0.07; 0.12]
PanYueFei 2020[522] 45 215 0.21 [0.16; 0.27]
Common effect model 863 0.12 [0.10; 0.15]
Random effects model 0.14 [0.08; 0.24]
Heterogeneity: I^2 = 95%, τ^2 = 0.1949, p < 0.01
month = 8
LongQiZhi 2016[442] 101 1707 0.06 [0.05; 0.07]
Zhou X 2012[1081] 108 5769 0.02 [0.02; 0.02]
Common effect model 7476 0.03 [0.02; 0.03]
Random effects model 0.03 [0.01; 0.07]
Heterogeneity: I^2 = 99%, τ^2 = 0.3456, p < 0.01

**month = 9**				
Jin−Bo Ye 2018[830−14]	132	2584	0.05	[0.04; 0.06]
ZhouAiHua 2017[950]	80	650	0.12	[0.10; 0.15]
**Common effect model**		**3234**	**0.07**	**[0.06; 0.07]**
**Random effects model**			**0.08**	**[0.04; 0.14]**
Heterogeneity: $I^2 = 98\%$, $\tau^2 = 0.2176$, $p < 0.01$				
**month = 10**				
GuYiFu 2020[197]	45	1903	0.02	[0.02; 0.03]
Jin−Bo Ye 2018[830−9]	40	1125	0.04	[0.03; 0.05]
Yang Z 2011[1068]	427	71534	0.01	[0.01; 0.01]
**Common effect model**		**74562**	**0.01**	**[0.01; 0.01]**
**Random effects model**			**0.02**	**[0.01; 0.04]**
Heterogeneity: $I^2 = 99\%$, $\tau^2 = 0.5897$, $p < 0.01$				
**month = 11**				
Cai W 2018[23−3]	217	2504	0.09	[0.08; 0.10]
ChaRiSheng 2014[47−2]	29	498	0.06	[0.04; 0.08]
HuangGuo 2015[252]	69	1220	0.06	[0.04; 0.07]
JiLei 2018[272−3]	18	191	0.09	[0.06; 0.14]
LiaoChan 2021[380]	19	675	0.03	[0.02; 0.04]
WangTieJun 2020[678]	15	345	0.04	[0.02; 0.07]
WuYiLing 2017[736]	31	1311	0.02	[0.02; 0.03]
XingYan 2017[759]	119	3500	0.03	[0.03; 0.04]
XuanLingFeng 2014[784]	17	9523	0.00	[0.00; 0.00]
Jin−Bo Ye 2018[830−5]	90	255	0.35	[0.29; 0.42]
Xue C 2014[1060]	278	1995	0.14	[0.12; 0.16]
**Common effect model**		**22017**	**0.04**	**[0.04; 0.04]**
**Random effects model**			**0.05**	**[0.02; 0.10]**
Heterogeneity: $I^2 = 99\%$, $\tau^2 = 1.8022$, $p < 0.01$				
**month = 12**				
ChenBinBin 2017[60−1]	54	392	0.14	[0.11; 0.18]
LiDaiBo 2018[320]	18	56	0.32	[0.20; 0.46]
LinJian 2019[384]	77	2476	0.03	[0.02; 0.04]
LuoQiong 2019[474]	22	2256	0.01	[0.01; 0.01]
MaoJianYing 2016[493]	51	559	0.09	[0.07; 0.12]
YangGenMei 2015[798]	54	392	0.14	[0.11; 0.18]
**Common effect model**		**6131**	**0.05**	**[0.04; 0.05]**
**Random effects model**			**0.08**	**[0.03; 0.18]**
Heterogeneity: $I^2 = 98\%$, $\tau^2 = 1.4980$, $p < 0.01$				
**Common effect model**		**178842**	**0.03**	**[0.03; 0.03]**
**Random effects model**			**0.06**	**[0.05; 0.09]**

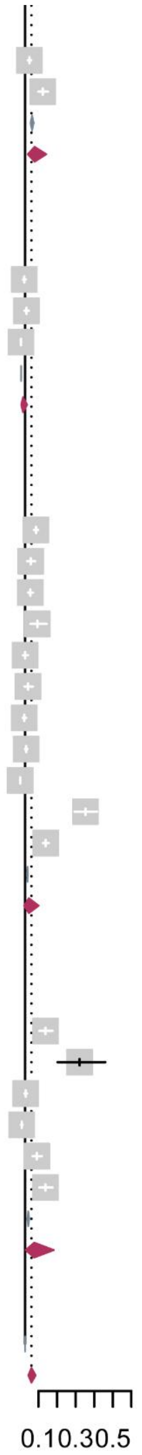

Heterogeneity: $I^2 = 99\%$, $\tau^2 = 1.4698$, $p = 0$

Test for subgroup differences (common effect): $\chi^2_{10} = 2652.46$, df = 10 ($p = 0$)

Test for subgroup differences (random effects): $\chi^2_{10} = 108.06$, df = 10 ($p < 0.01$)

(C)

(c1)

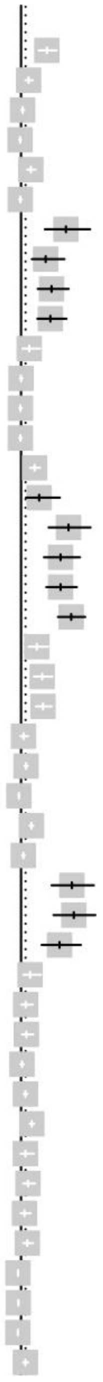

Study	Events	Total	Attack rate	95%−CI
Ecological_regions = I				
CaiXiuZhi 2018[27−1]	34	150	0.23	[0.16; 0.30]
CaiXiuZhi 2018[27−2]	34	400	0.08	[0.06; 0.12]
CaiXiuZhi 2018[27−3]	40	1045	0.04	[0.03; 0.05]
CaiXiuZhi 2018[27−4]	24	1279	0.02	[0.01; 0.03]
CuiXiaoMan 2015[124]	34	332	0.10	[0.07; 0.14]
CuiXiaoMan 2018[125]	17	829	0.02	[0.01; 0.03]
GaoZhiYong 2017[187−1]	12	32	0.38	[0.21; 0.56]
GaoZhiYong 2017[187−2]	10	46	0.22	[0.11; 0.36]
GaoZhiYong 2017[187−3]	15	57	0.26	[0.16; 0.40]
GaoZhiYong 2017[187−4]	16	63	0.25	[0.15; 0.38]
GaoZhiYong 2017[187−5]	7	78	0.09	[0.04; 0.18]
GaoZhiYong 2017[187−6]	25	953	0.03	[0.02; 0.04]
GaoZhiYong 2017[187−7]	21	980	0.02	[0.01; 0.03]
GaoZhiYong 2017[187−8]	22	1047	0.02	[0.01; 0.03]
JiangXiHong 2019[293]	40	304	0.13	[0.10; 0.17]
LiShiE 2018[347−1]	6	36	0.17	[0.06; 0.33]
LiShiE 2018[347−2]	15	38	0.39	[0.24; 0.57]
LiShiE 2018[347−3]	16	48	0.33	[0.20; 0.48]
LiShiE 2018[347−4]	22	66	0.33	[0.22; 0.46]
LiShiE 2018[347−5]	37	89	0.42	[0.31; 0.53]
LiShiE 2018[347−6]	14	94	0.15	[0.08; 0.24]
LiShiE 2018[347−7]	24	126	0.19	[0.13; 0.27]
LiShiE 2018[347−8]	34	173	0.20	[0.14; 0.26]
LiShiE 2018[347−9]	10	221	0.05	[0.02; 0.08]
LiShiE 2018[348]	92	1419	0.06	[0.05; 0.08]
LiShiE 2018[349]	77	8153	0.01	[0.01; 0.01]
QiYing 2018[535−1]	173	1621	0.11	[0.09; 0.12]
QiYing 2018[535−2]	71	1621	0.04	[0.03; 0.05]
QiYing 2019[535−3]	16	38	0.42	[0.26; 0.59]
QiYing 2019[535−4]	17	39	0.44	[0.28; 0.60]
QiYing 2019[535−5]	13	40	0.32	[0.19; 0.49]
QiYing 2019[535−6]	8	83	0.10	[0.04; 0.18]
QiYing 2019[535−7]	8	129	0.06	[0.03; 0.12]
QiYing 2019[535−8]	10	148	0.07	[0.03; 0.12]
QiYing 2019[535−9]	34	1015	0.03	[0.02; 0.05]
QiYing 2019[535−10]	72	1192	0.06	[0.05; 0.08]
QiYing 2019[535−11]	159	1448	0.11	[0.09; 0.13]
RenQiZhi 2018[557−1]	6	100	0.06	[0.02; 0.13]
RenQiZhi 2018[557−2]	12	150	0.08	[0.04; 0.14]
RenQiZhi 2018[557−3]	8	151	0.05	[0.02; 0.10]
RenQiZhi 2018[557−4]	15	200	0.07	[0.04; 0.12]
RenQiZhi 2018[557−5]	5	2000	0.00	[0.00; 0.01]
RenQiZhi 2018[557−6]	13	2500	0.01	[0.00; 0.01]
RenQiZhi 2018[557−7]	15	10000	0.00	[0.00; 0.00]
SongHuiRong 2017[597]	34	568	0.06	[0.04; 0.08]

WangBing 2017[649]	58	20000	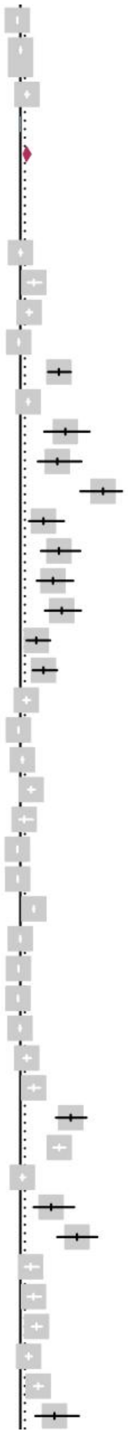	0.00 [0.00; 0.00]
XuJun 2018[779]	75	2679		0.03 [0.02; 0.03]
YuXiaoYun 2018[845]	78	1007		0.08 [0.06; 0.10]
**Common effect model**		**64787**		**0.02 [0.02; 0.03]**
**Random effects model**				**0.07 [0.05; 0.11]**
Heterogeneity: I^2 = 98%, τ^2 = 2.1770, p = 0				
**Ecological_regions = II**				
BaiAiLi 2020[3]	20	738		0.03 [0.02; 0.04]
BaiYao 2020[6]	22	169		0.13 [0.08; 0.19]
BaiYun 2018[8]	61	648		0.09 [0.07; 0.12]
Cai SX 2017[21]	17	1276		0.01 [0.01; 0.02]
Cai W 2018[23−2]	38	117		0.32 [0.24; 0.42]
Cai W 2018[23−3]	217	2504		0.09 [0.08; 0.10]
Cai W 2017[24−4]	12	32		0.38 [0.21; 0.56]
Cai W 2017[24−5]	10	32		0.31 [0.16; 0.50]
Cai W 2017[24−6]	24	36		0.67 [0.49; 0.81]
Cai W 2017[24−7]	8	39		0.21 [0.09; 0.36]
Cai W 2017[24−8]	13	40		0.32 [0.19; 0.49]
Cai W 2017[24−9]	12	43		0.28 [0.15; 0.44]
Cai W 2017[24−10]	17	49		0.35 [0.22; 0.50]
Cai W 2017[24−11]	10	67		0.15 [0.07; 0.26]
Cai W 2017[24−12]	16	78		0.21 [0.12; 0.31]
ZengLei 2018[44]	28	388		0.07 [0.05; 0.10]
ChenJianMei 2017[79]	110	9736		0.01 [0.01; 0.01]
ChenQian 2014[95]	78	1844		0.04 [0.03; 0.05]
ChenYan 2019[104]	59	536		0.11 [0.08; 0.14]
CuiLiangLiang 2017[123−1]	5	90		0.06 [0.02; 0.12]
CuiLiangLiang 2017[123−2]	14	4600		0.00 [0.00; 0.01]
CuiLiangLiang 2017[123−3]	55	10000		0.01 [0.00; 0.01]
CuiLiangLiang 2017[123−4]	1434	11000		0.13 [0.12; 0.14]
CuiLiangLiang 2017[123−5]	385	15000		0.03 [0.02; 0.03]
CuiLiangLiang 2017[123−6]	235	20000		0.01 [0.01; 0.01]
DingJianQing 2012[138]	82	10177		0.01 [0.01; 0.01]
DuYueHe 2019[148]	29	1250		0.02 [0.02; 0.03]
FangYunXia 2018[157]	27	357		0.08 [0.05; 0.11]
GaoPeng 2020[181]	28	217		0.13 [0.09; 0.18]
GaoRiHong 2019[182]	30	72		0.42 [0.30; 0.54]
GaoZhiPeng 2018[186]	130	397		0.33 [0.28; 0.38]
GuKaiChen 2020[195]	21	500		0.04 [0.03; 0.06]
GuoJianXin 2015[202−1]	9	34		0.26 [0.13; 0.44]
GuoJianXin 2015[202−2]	20	43		0.47 [0.31; 0.62]
GuoJianXin 2015[202−3]	15	142		0.11 [0.06; 0.17]
GuoJianXin 2015[202−4]	22	172		0.13 [0.08; 0.19]
GuoJianXin 2015[202−5]	53	350		0.15 [0.12; 0.19]
GuoJianXin 2015[202−6]	55	611		0.09 [0.07; 0.12]
GuoLi 2019[205]	75	456		0.16 [0.13; 0.20]
GuoLi 2019[206−15]	9	31		0.29 [0.14; 0.48]

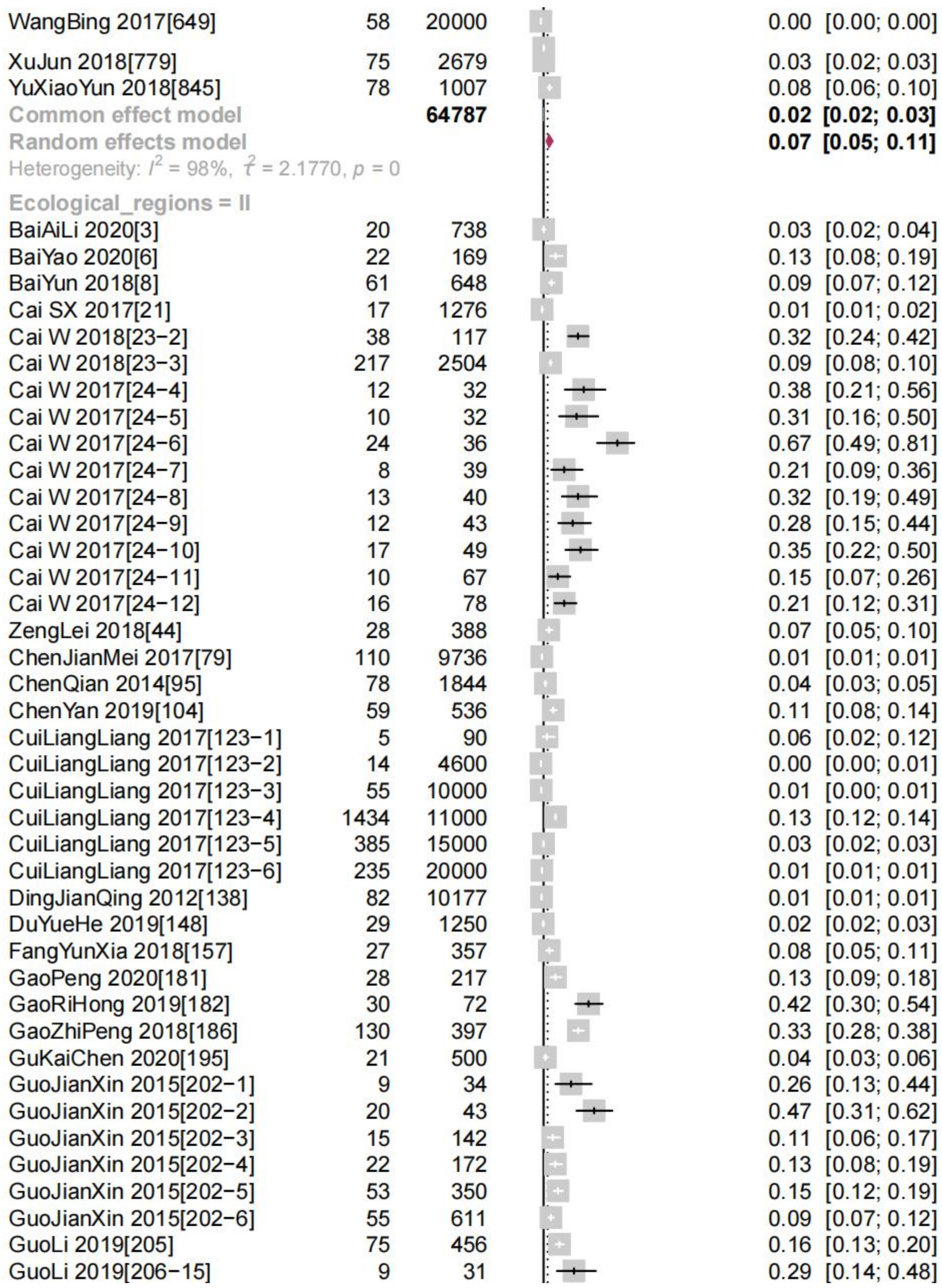

WangBing 2017[649] 58 20000 0.00 [0.00; 0.00]
XuJun 2018[779] 75 2679 0.03 [0.02; 0.03]
YuXiaoYun 2018[845] 78 1007 0.08 [0.06; 0.10]
Common effect model 64787 0.02 [0.02; 0.03]
Random effects model 0.07 [0.05; 0.11]
Heterogeneity: I^2 = 98%, τ^2 = 2.1770, p = 0
Ecological_regions = II
BaiAiLi 2020[3] 20 738 0.03 [0.02; 0.04]
BaiYao 2020[6] 22 169 0.13 [0.08; 0.19]
BaiYun 2018[8] 61 648 0.09 [0.07; 0.12]
Cai SX 2017[21] 17 1276 0.01 [0.01; 0.02]
Cai W 2018[23−2] 38 117 0.32 [0.24; 0.42]
Cai W 2018[23−3] 217 2504 0.09 [0.08; 0.10]
Cai W 2017[24−4] 12 32 0.38 [0.21; 0.56]
Cai W 2017[24−5] 10 32 0.31 [0.16; 0.50]
Cai W 2017[24−6] 24 36 0.67 [0.49; 0.81]
Cai W 2017[24−7] 8 39 0.21 [0.09; 0.36]
Cai W 2017[24−8] 13 40 0.32 [0.19; 0.49]
Cai W 2017[24−9] 12 43 0.28 [0.15; 0.44]
Cai W 2017[24−10] 17 49 0.35 [0.22; 0.50]
Cai W 2017[24−11] 10 67 0.15 [0.07; 0.26]
Cai W 2017[24−12] 16 78 0.21 [0.12; 0.31]
ZengLei 2018[44] 28 388 0.07 [0.05; 0.10]
ChenJianMei 2017[79] 110 9736 0.01 [0.01; 0.01]
ChenQian 2014[95] 78 1844 0.04 [0.03; 0.05]
ChenYan 2019[104] 59 536 0.11 [0.08; 0.14]
CuiLiangLiang 2017[123−1] 5 90 0.06 [0.02; 0.12]
CuiLiangLiang 2017[123−2] 14 4600 0.00 [0.00; 0.01]
CuiLiangLiang 2017[123−3] 55 10000 0.01 [0.00; 0.01]
CuiLiangLiang 2017[123−4] 1434 11000 0.13 [0.12; 0.14]
CuiLiangLiang 2017[123−5] 385 15000 0.03 [0.02; 0.03]
CuiLiangLiang 2017[123−6] 235 20000 0.01 [0.01; 0.01]
DingJianQing 2012[138] 82 10177 0.01 [0.01; 0.01]
DuYueHe 2019[148] 29 1250 0.02 [0.02; 0.03]
FangYunXia 2018[157] 27 357 0.08 [0.05; 0.11]
GaoPeng 2020[181] 28 217 0.13 [0.09; 0.18]
GaoRiHong 2019[182] 30 72 0.42 [0.30; 0.54]
GaoZhiPeng 2018[186] 130 397 0.33 [0.28; 0.38]
GuKaiChen 2020[195] 21 500 0.04 [0.03; 0.06]
GuoJianXin 2015[202−1] 9 34 0.26 [0.13; 0.44]
GuoJianXin 2015[202−2] 20 43 0.47 [0.31; 0.62]
GuoJianXin 2015[202−3] 15 142 0.11 [0.06; 0.17]
GuoJianXin 2015[202−4] 22 172 0.13 [0.08; 0.19]
GuoJianXin 2015[202−5] 53 350 0.15 [0.12; 0.19]
GuoJianXin 2015[202−6] 55 611 0.09 [0.07; 0.12]
GuoLi 2019[205] 75 456 0.16 [0.13; 0.20]
GuoLi 2019[206−15] 9 31 0.29 [0.14; 0.48]

GuoLi 2019[206−16]	8	31	0.26 [0.12; 0.45]
GuoLi 2019[206−17]	10	31	0.32 [0.17; 0.51]
GuoLi 2019[206−18]	14	32	0.44 [0.26; 0.62]
GuoLi 2019[206−19]	9	33	0.27 [0.13; 0.46]
GuoLi 2019[206−20]	18	33	0.55 [0.36; 0.72]
GuoLi 2019[206−21]	8	34	0.24 [0.11; 0.41]
GuoLi 2019[206−22]	10	34	0.29 [0.15; 0.47]
GuoLi 2019[206−23]	14	34	0.41 [0.25; 0.59]
GuoLi 2019[206−24]	18	35	0.51 [0.34; 0.69]
GuoLi 2019[206−25]	16	35	0.46 [0.29; 0.63]
GuoLi 2019[206−26]	6	35	0.17 [0.07; 0.34]
GuoLi 2019[206−27]	17	36	0.47 [0.30; 0.65]
GuoLi 2019[206−28]	10	37	0.27 [0.14; 0.44]
GuoLi 2019[206−29]	14	38	0.37 [0.22; 0.54]
GuoLi 2019[206−30]	6	38	0.16 [0.06; 0.31]
GuoLi 2019[206−31]	12	39	0.31 [0.17; 0.48]
GuoLi 2019[206−32]	18	40	0.45 [0.29; 0.62]
GuoLi 2019[206−33]	16	40	0.40 [0.25; 0.57]
GuoLi 2019[206−34]	7	40	0.17 [0.07; 0.33]
GuoLi 2019[206−35]	7	41	0.17 [0.07; 0.32]
GuoLi 2019[206−36]	16	41	0.39 [0.24; 0.55]
GuoLi 2019[206−37]	10	41	0.24 [0.12; 0.40]
GuoLi 2019[206−38]	18	41	0.44 [0.28; 0.60]
GuoLi 2019[206−39]	9	41	0.22 [0.11; 0.38]
GuoLi 2019[206−40]	13	42	0.31 [0.18; 0.47]
GuoLi 2019[206−41]	5	42	0.12 [0.04; 0.26]
GuoLi 2019[206−42]	12	42	0.29 [0.16; 0.45]
GuoLi 2019[206−43]	16	43	0.37 [0.23; 0.53]
GuoLi 2019[206−44]	13	44	0.30 [0.17; 0.45]
GuoLi 2019[206−45]	25	44	0.57 [0.41; 0.72]
GuoLi 2019[206−46]	14	46	0.30 [0.18; 0.46]
GuoLi 2019[206−47]	9	46	0.20 [0.09; 0.34]
GuoLi 2019[206−48]	18	60	0.30 [0.19; 0.43]
GuoLi 2019[206−49]	14	65	0.22 [0.12; 0.33]
GuoLi 2019[206−50]	10	70	0.14 [0.07; 0.25]
GuoLi 2019[206−51]	18	72	0.25 [0.16; 0.37]
GuoLi 2019[206−52]	7	79	0.09 [0.04; 0.17]
GuoLi 2019[206−53]	23	80	0.29 [0.19; 0.40]
GuoLi 2019[206−54]	17	81	0.21 [0.13; 0.31]
GuoLi 2019[206−55]	10	101	0.10 [0.05; 0.17]
GuoLi 2019[206−56]	13	101	0.13 [0.07; 0.21]
GuoLi 2019[206−57]	13	119	0.11 [0.06; 0.18]
GuoLi 2019[206−58]	16	171	0.09 [0.05; 0.15]
GuoLi 2019[206−59]	14	211	0.07 [0.04; 0.11]
GuoLi 2019[206−60]	61	220	0.28 [0.22; 0.34]
GuoLi 2019[206−61]	28	281	0.10 [0.07; 0.14]
GuoLi 2019[206−62]	47	339	0.14 [0.10; 0.18]
GuoLi 2019[206−63]	30	440	0.07 [0.05; 0.10]

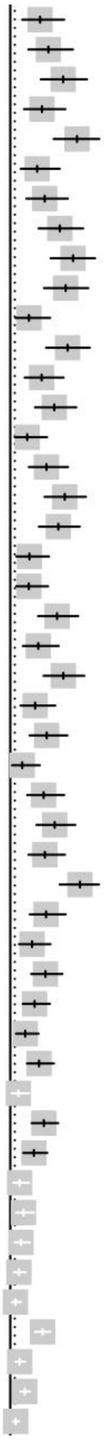

GuoLi 2019[206−64]	25	498	0.05 [0.03; 0.07]
GuoLi 2019[206−65]	28	698	0.04 [0.03; 0.06]
HeXuXin 2017[222]	156	466	0.33 [0.29; 0.38]
HuGuangYi 2017[232]	20	210	0.10 [0.06; 0.14]
HuaWeiYu 2018[241]	63	1951	0.03 [0.02; 0.04]
HuaWeiYu 2018[242−1]	15	156	0.10 [0.05; 0.15]
HuaWeiYu 2018[242−2]	17	267	0.06 [0.04; 0.10]
HuaWeiYu 2018[242−3]	9	290	0.03 [0.01; 0.06]
HuaWeiYu 2018[242−4]	14	320	0.04 [0.02; 0.07]
HuaWeiYu 2018[242−5]	13	325	0.04 [0.02; 0.07]
HuaWeiYu 2018[242−6]	5	833	0.01 [0.00; 0.01]
HuaWeiYu 2018[242−7]	13	1806	0.01 [0.00; 0.01]
HuangYanHong 2019[259−2]	37	79	0.47 [0.36; 0.58]
HuangYanHong 2019[259−3]	27	115	0.23 [0.16; 0.32]
HuangYanHong 2019[259−4]	23	120	0.19 [0.13; 0.27]
HuangYanHong 2019[259−5]	24	120	0.20 [0.13; 0.28]
HuangYanHong 2019[259−6]	21	140	0.15 [0.10; 0.22]
HuangYanHong 2019[259−7]	31	146	0.21 [0.15; 0.29]
HuangYanHong 2019[259−8]	27	172	0.16 [0.11; 0.22]
HuangYanHong 2019[259−9]	30	176	0.17 [0.12; 0.23]
HuangYanHong 2019[259−10]	26	199	0.13 [0.09; 0.19]
HuangYanHong 2019[259−11]	32	456	0.07 [0.05; 0.10]
HuangYanHong 2019[259−12]	58	498	0.12 [0.09; 0.15]
HuangYanHong 2019[260]	84	760	0.11 [0.09; 0.14]
HuangYanHong 2019[261]	86	1190	0.07 [0.06; 0.09]
JiHong 2015[264]	78	3194	0.02 [0.02; 0.03]
JinXiaoFang 2015[302]	38	281	0.14 [0.10; 0.18]
LiCaiYun 2012[318]	147	940	0.16 [0.13; 0.18]
LiJiShan 2018[324−2]	7	40	0.17 [0.07; 0.33]
LiJiShan 2018[324−3]	7	78	0.09 [0.04; 0.18]
LiJiShan 2016[326−1]	16	35	0.46 [0.29; 0.63]
LiJiShan 2016[326−2]	18	41	0.44 [0.28; 0.60]
LiJiShan 2016[326−3]	8	46	0.17 [0.08; 0.31]
LiJun 2012[334]	1252	2877	0.44 [0.42; 0.45]
LiXiTai 2015[352]	51	1336	0.04 [0.03; 0.05]
LiYan 2017[361]	93	2355	0.04 [0.03; 0.05]
LiangPing 2020[374]	25	196	0.13 [0.08; 0.18]
LiuBaiWei 2017[395]	24	471	0.05 [0.03; 0.07]
LiuBaiWei 2017[396−1]	43	222	0.19 [0.14; 0.25]
LiuBaiWei 2017[396−2]	172	3546	0.05 [0.04; 0.06]
LiuBaiWei 2017[396−3]	58	4500	0.01 [0.01; 0.02]
LiuCaiXia 2019[399−3]	20	208	0.10 [0.06; 0.14]
LiuDongSheng 2019[404]	80	3720	0.02 [0.02; 0.03]
LiuWenJun 2018[428−1]	18	255	0.07 [0.04; 0.11]
LiuWenJun 2018[428−2]	22	388	0.06 [0.04; 0.08]
LiuWenJun 2018[428−3]	38	528	0.07 [0.05; 0.10]
LiuWenJun 2018[428−4]	42	565	0.07 [0.05; 0.10]
LiuWenJun 2018[428−5]	48	672	0.07 [0.05; 0.09]

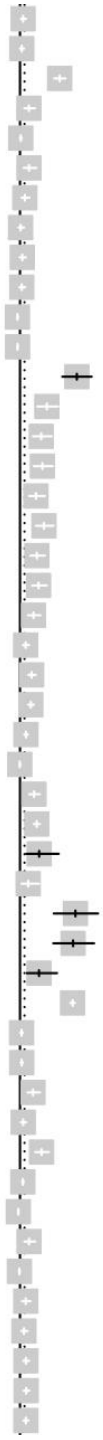

LiuWenJun 2018[428−6]	36	700	0.05 [0.04; 0.07]
LiuWenJun 2018[428−7]	18	826	0.02 [0.01; 0.03]
LiuWenJun 2018[428−8]	16	904	0.02 [0.01; 0.03]
LiuWenJun 2018[428−9]	105	907	0.12 [0.10; 0.14]
LiuWenJun 2018[428−10]	31	951	0.03 [0.02; 0.05]
LiuWenJun 2018[428−11]	55	955	0.06 [0.04; 0.07]
LiuWenJun 2018[428−12]	49	1213	0.04 [0.03; 0.05]
LiuWenJun 2018[428−13]	193	1285	0.15 [0.13; 0.17]
LiuWenJun 2018[428−14]	46	1314	0.04 [0.03; 0.05]
LiuWenJun 2018[428−15]	17	1328	0.01 [0.01; 0.02]
LiuWenJun 2018[428−16]	54	1467	0.04 [0.03; 0.05]
LiuWenJun 2018[428−17]	180	1554	0.12 [0.10; 0.13]
LiuWenJun 2018[428−18]	19	1776	0.01 [0.01; 0.02]
LiuWenJun 2018[428−19]	68	1926	0.04 [0.03; 0.04]
LiuWenJun 2018[428−20]	17	1932	0.01 [0.01; 0.01]
LiuWenJun 2018[428−21]	55	2174	0.03 [0.02; 0.03]
LiuWenJun 2018[428−22]	52	2332	0.02 [0.02; 0.03]
LiuWenJun 2018[428−23]	70	3608	0.02 [0.02; 0.02]
LiuWenJun 2018[428−24]	43	3707	0.01 [0.01; 0.02]
LiuXiaoXiao 2014[430]	18	70	0.26 [0.16; 0.38]
LiuXiuMei 2017[433]	65	1134	0.06 [0.04; 0.07]
LiuYuan 2016[438−1]	15	142	0.11 [0.06; 0.17]
LiuYuan 2016[438−2]	121	378	0.32 [0.27; 0.37]
LiuYuan 2016[438−3]	55	611	0.09 [0.07; 0.12]
LuZhengXiang 2016[451]	83	4624	0.02 [0.01; 0.02]
LuXiuZhi 2018[463]	47	3600	0.01 [0.01; 0.02]
LuoQiong 2019[474]	22	2256	0.01 [0.01; 0.01]
NiChunYan 2020[512]	55	491	0.11 [0.09; 0.14]
PanYueFei 2020[522]	45	215	0.21 [0.16; 0.27]
QiYanQiu 2018[532]	27	570	0.05 [0.03; 0.07]
QinDi 2016[544]	16	255	0.06 [0.04; 0.10]
QinLianYang 2021[545]	38	1697	0.02 [0.02; 0.03]
QinMeng 2015[547−1]	35	217	0.16 [0.11; 0.22]
QinMeng 2015[547−2]	25	228	0.11 [0.07; 0.16]
RenLiJun 2020[556]	9	253	0.04 [0.02; 0.07]
SongJie 2014[599]	17	350	0.05 [0.03; 0.08]
SuTong 2020[604]	55	3432	0.02 [0.01; 0.02]
SunHaiBo 2012[608]	66	100177	0.00 [0.00; 0.00]
SunWenLong 2018[618]	8	142	0.06 [0.02; 0.11]
TianJing 2017[641]	39	392	0.10 [0.07; 0.13]
TianYaLin 2021[642]	38	546	0.07 [0.05; 0.09]
WangMingLiang 2020[671]	107	550	0.19 [0.16; 0.23]
WangXiaoDong 2017[688]	69	2708	0.03 [0.02; 0.03]
WeiYiYun 2015[705]	22	155	0.14 [0.09; 0.21]
XieBin 2018[757]	95	5000	0.02 [0.02; 0.02]
XieBin 2020[758]	65	2036	0.03 [0.02; 0.04]
XingYan 2017[759]	119	3500	0.03 [0.03; 0.04]

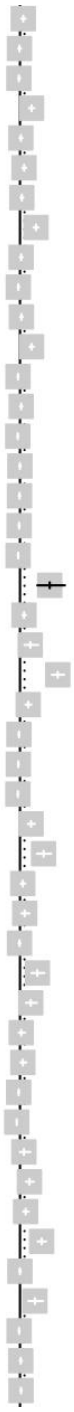

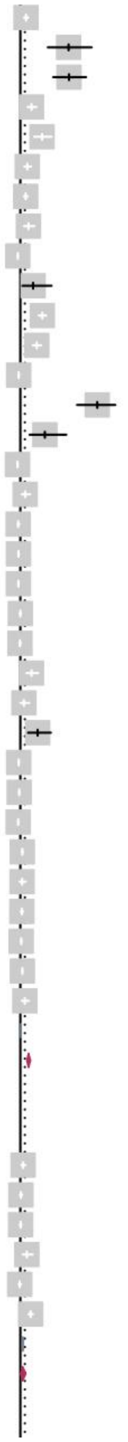

XuYan 2018[772]	68	969	0.07 [0.05; 0.09]
YanGeBin 2017[786−1]	14	35	0.40 [0.24; 0.58]
YanGeBin 2017[786−2]	25	62	0.40 [0.28; 0.54]
YangAiQing 2018[795]	40	355	0.11 [0.08; 0.15]
YangShiYong 2015[811]	28	144	0.19 [0.13; 0.27]
YangTongTong 2018[813]	36	450	0.08 [0.06; 0.11]
YuHong 2016[848]	80	1184	0.07 [0.05; 0.08]
ZhangAiHua 2018[861]	19	214	0.09 [0.05; 0.14]
ZhangChong 2015[862]	13	2050	0.01 [0.00; 0.01]
ZhangDianXiang 2012[863]	5	40	0.12 [0.04; 0.27]
ZhangHaiYan 2019[867]	145	738	0.20 [0.17; 0.23]
ZhangHaiYan 2016[868]	78	508	0.15 [0.12; 0.19]
ZhangQin 2020[885]	57	4279	0.01 [0.01; 0.02]
ZhangShuang 2017[888]	28	45	0.62 [0.47; 0.76]
ZhangYan 2017[894−1]	8	37	0.22 [0.10; 0.38]
ZhangYan 2017[894−2]	12	2857	0.00 [0.00; 0.01]
ZhangYanMing 2019[898]	15	236	0.06 [0.04; 0.10]
ZhaoMeiLing 2019[918−1]	8	842	0.01 [0.00; 0.02]
ZhaoMeiLing 2019[918−2]	39	3305	0.01 [0.01; 0.02]
ZhaoQi 2021[920]	53	4760	0.01 [0.01; 0.01]
ZhaoWeiQin 2020[923]	128	4927	0.03 [0.02; 0.03]
ZhaoWenNa 2021[924]	70	3000	0.02 [0.02; 0.03]
ZhenGuoXin 2020[936]	23	204	0.11 [0.07; 0.16]
ZhenGuoXin 2020[937]	11	204	0.05 [0.03; 0.09]
ZhouGuoYing 2016[952]	12	75	0.16 [0.09; 0.26]
ZhouXiaoHong 2017[961]	47	3464	0.01 [0.01; 0.02]
ZhuChengMing 2017[967]	78	4454	0.02 [0.01; 0.02]
ZhuWeiWei 2018[976]	91	9702	0.01 [0.01; 0.01]
Chen D 2019[987]	209	5043	0.04 [0.04; 0.05]
Xiao GD 2019[995]	12	302	0.04 [0.02; 0.07]
Guo XH 2018[1000]	61	1606	0.04 [0.03; 0.05]
Huang XY 2017[1007]	753	22861	0.03 [0.03; 0.04]
Zhang L 2018[1071]	631	15118	0.04 [0.04; 0.05]
Zhang TL 2017[1075]	20	327	0.06 [0.04; 0.09]
**Common effect model**		**397060**	**0.03 [0.03; 0.03]**
**Random effects model**			**0.09 [0.08; 0.11]**
Heterogeneity: I^2 = 99%, τ^2 = 1.8573, p = 0			
**Ecological_regions = III**			
KangQian 2020[303−1]	35	736	0.05 [0.03; 0.07]
KangQian 2020[303−2]	50	1622	0.03 [0.02; 0.04]
LiBing 2019[315]	67	2354	0.03 [0.02; 0.04]
LiChunLing 2020[319]	15	196	0.08 [0.04; 0.12]
WeiXia 2019[708]	22	1024	0.02 [0.01; 0.03]
ZhuHaiYang 2016[971]	140	1319	0.11 [0.09; 0.12]
**Common effect model**		**7251**	**0.05 [0.04; 0.05]**
**Random effects model**			**0.04 [0.03; 0.07]**
Heterogeneity: I^2 = 96%, τ^2 = 0.3340, p < 0.01			

(D)

(d1)

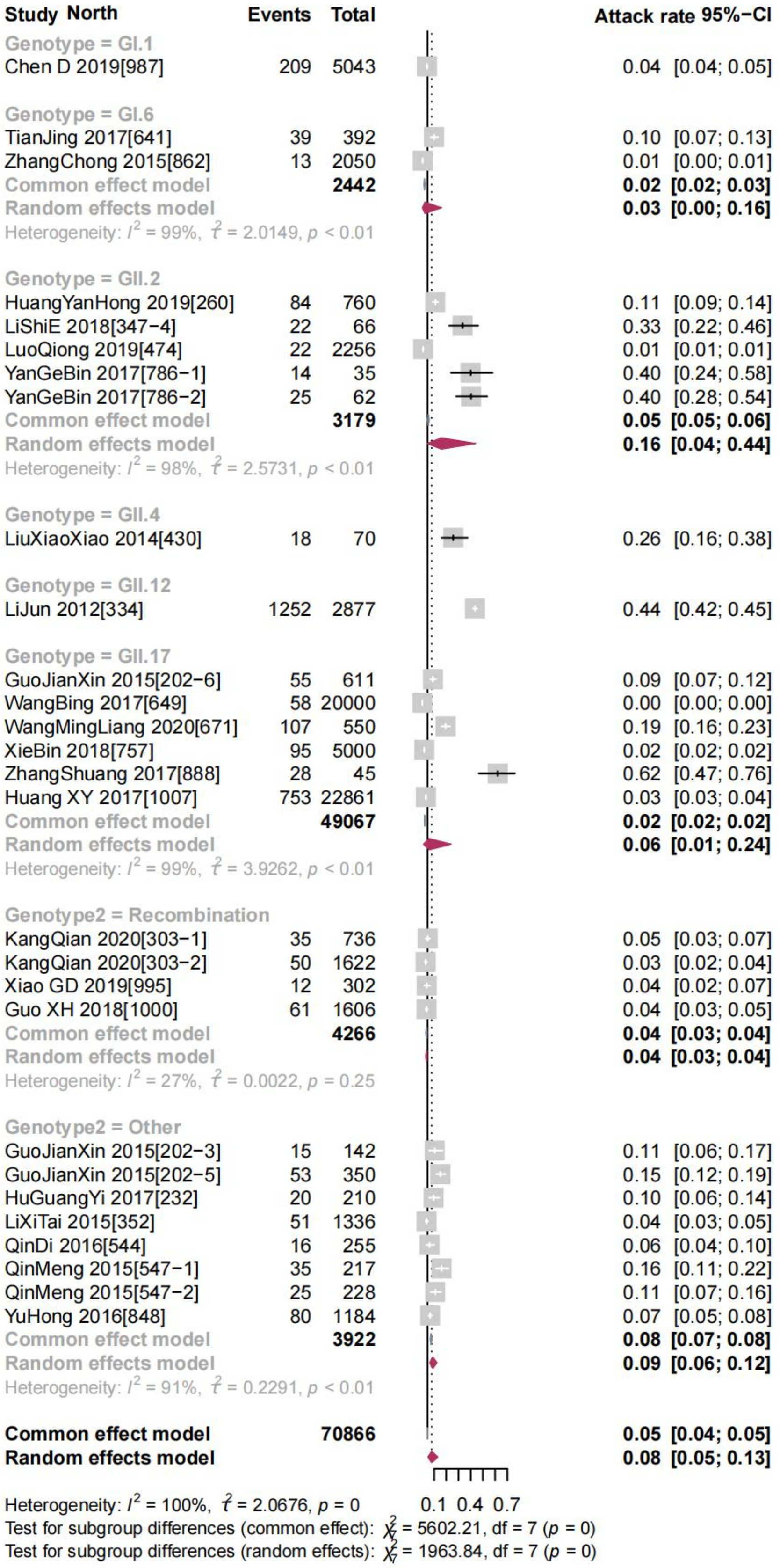
Study North
Events
Total
Attack rate 95%−CI
Genotype = GI.1
Chen D 2019[987] 209 5043 0.04 [0.04; 0.05]
Genotype = GI.6
TianJing 2017[641] 39 392 0.10 [0.07; 0.13]
ZhangChong 2015[862] 13 2050 0.01 [0.00; 0.01]
Common effect model 2442 0.02 [0.02; 0.03]
Random effects model 0.03 [0.00; 0.16]
Heterogeneity: I^2 = 99%, τ^2 = 2.0149, p < 0.01
Genotype = GII.2
HuangYanHong 2019[260] 84 760 0.11 [0.09; 0.14]
LiShiE 2018[347−4] 22 66 0.33 [0.22; 0.46]
LuoQiong 2019[474] 22 2256 0.01 [0.01; 0.01]
YanGeBin 2017[786−1] 14 35 0.40 [0.24; 0.58]
YanGeBin 2017[786−2] 25 62 0.40 [0.28; 0.54]
Common effect model 3179 0.05 [0.05; 0.06]
Random effects model 0.16 [0.04; 0.44]
Heterogeneity: I^2 = 98%, τ^2 = 2.5731, p < 0.01
Genotype = GII.4
LiuXiaoXiao 2014[430] 18 70 0.26 [0.16; 0.38]
Genotype = GII.12
LiJun 2012[334] 1252 2877 0.44 [0.42; 0.45]
Genotype = GII.17
GuoJianXin 2015[202−6] 55 611 0.09 [0.07; 0.12]
WangBing 2017[649] 58 20000 0.00 [0.00; 0.00]
WangMingLiang 2020[671] 107 550 0.19 [0.16; 0.23]
XieBin 2018[757] 95 5000 0.02 [0.02; 0.02]
ZhangShuang 2017[888] 28 45 0.62 [0.47; 0.76]
Huang XY 2017[1007] 753 22861 0.03 [0.03; 0.04]
Common effect model 49067 0.02 [0.02; 0.02]
Random effects model 0.06 [0.01; 0.24]
Heterogeneity: I^2 = 99%, τ^2 = 3.9262, p < 0.01
Genotype2 = Recombination
KangQian 2020[303−1] 35 736 0.05 [0.03; 0.07]
KangQian 2020[303−2] 50 1622 0.03 [0.02; 0.04]
Xiao GD 2019[995] 12 302 0.04 [0.02; 0.07]
Guo XH 2018[1000] 61 1606 0.04 [0.03; 0.05]
Common effect model 4266 0.04 [0.03; 0.04]
Random effects model 0.04 [0.03; 0.04]
Heterogeneity: I^2 = 27%, τ^2 = 0.0022, p = 0.25
Genotype2 = Other
GuoJianXin 2015[202−3] 15 142 0.11 [0.06; 0.17]
GuoJianXin 2015[202−5] 53 350 0.15 [0.12; 0.19]
HuGuangYi 2017[232] 20 210 0.10 [0.06; 0.14]
LiXiTai 2015[352] 51 1336 0.04 [0.03; 0.05]
QinDi 2016[544] 16 255 0.06 [0.04; 0.10]
QinMeng 2015[547−1] 35 217 0.16 [0.11; 0.22]
QinMeng 2015[547−2] 25 228 0.11 [0.07; 0.16]
YuHong 2016[848] 80 1184 0.07 [0.05; 0.08]
Common effect model 3922 0.08 [0.07; 0.08]
Random effects model 0.09 [0.06; 0.12]
Heterogeneity: I^2 = 91%, τ^2 = 0.2291, p < 0.01
Common effect model 70866 0.05 [0.04; 0.05]
Random effects model 0.08 [0.05; 0.13]
0.1 0.4 0.7
Heterogeneity: I^2 = 100%, τ^2 = 2.0676, p = 0
Test for subgroup differences (common effect): χ^2_7 = 5602.21, df = 7 (p = 0)
Test for subgroup differences (random effects): χ^2_7 = 1963.84, df = 7 (p = 0)

(d2)

Study South	Events	Total	Attack rate	95%−CI
**Genotype = GI.1**				
TanDongMei 2012[627]	87	2086	0.04	[0.03; 0.05]
**genotype2 = GI.6**				
ChenMinHong 2017[92−2]	186	657	0.28	[0.25; 0.32]
QiXiaoQi 2019[534]	185	2418	0.08	[0.07; 0.09]
**Common effect model**		**3075**	**0.12**	**[0.11; 0.13]**
**Random effects model**			**0.15**	**[0.06; 0.35]**
Heterogeneity: $I^2 = 99\%$, $\tau^2 = 0.6036$, $p < 0.01$				
**Genotype = GII.2**				
ShenYiPing 2018[52]	26	612	0.04	[0.03; 0.06]
ChenMinHong 2017[92−1]	21	236	0.09	[0.06; 0.13]
ChenMinHong 2017[92−3]	60	4286	0.01	[0.01; 0.02]
GaoJunYing 2017[177]	12	1501	0.01	[0.00; 0.01]
LinYanYan 2018[393]	76	3288	0.02	[0.02; 0.03]
WangJun 2021[664]	20	4743	0.00	[0.00; 0.01]
WuXiaoMin 2021[732]	159	1378	0.12	[0.10; 0.13]
XiaoDaYong 2018[746]	47	154	0.31	[0.23; 0.38]
XieCaiWen 2017[749]	12	58	0.21	[0.11; 0.33]
Zhang TL 2017[1075]	20	327	0.06	[0.04; 0.09]
**Common effect model**		**16583**	**0.03**	**[0.02; 0.03]**
**Random effects model**			**0.04**	**[0.02; 0.10]**
Heterogeneity: $I^2 = 99\%$, $\tau^2 = 1.9922$, $p < 0.01$				
**Genotype = GII.3**				
DaiBenNa 2020[127]	117	3119	0.04	[0.03; 0.04]
YangRongXing 2019[808]	15	334	0.04	[0.03; 0.07]
Y Li 2021[1018]	143	346	0.41	[0.36; 0.47]
**Common effect model**		**3799**	**0.07**	**[0.06; 0.08]**
**Random effects model**			**0.10**	**[0.02; 0.33]**
Heterogeneity: $I^2 = 100\%$, $\tau^2 = 1.7590$, $p < 0.01$				
**Genotype = GII.4**				
Cai MW 2018[18]	34	223	0.15	[0.11; 0.21]
CaiWenFeng 2013[25]	141	16600	0.01	[0.01; 0.01]
CaiWenFeng 2014[26]	27	816	0.03	[0.02; 0.05]
ZengFengMei 2020[41]	25	255	0.10	[0.06; 0.14]
ChenAQun 2015[58]	64	19153	0.00	[0.00; 0.00]
ChenAQun 2016[59]	64	18924	0.00	[0.00; 0.00]
ChenJian 2016[78]	20	1551	0.01	[0.01; 0.02]
JiHong 2015[264]	78	3194	0.02	[0.02; 0.03]
LeiYongLiang 2016[311]	46	984	0.05	[0.03; 0.06]
LiQun 2010[340]	258	14269	0.02	[0.02; 0.02]
Wu−Yang Shi 2020[577−1]	35	441	0.08	[0.06; 0.11]
SongCanLei 2012[591]	18	385	0.05	[0.03; 0.07]
SongCanLei 2013[592−2]	8	161	0.05	[0.02; 0.10]
SunJing 2015[611]	92	8929	0.01	[0.01; 0.01]
SunLiMei 2012[612]	108	5854	0.02	[0.02; 0.02]
WuZhiSheng 2014[741]	176	14612	0.01	[0.01; 0.01]
XieHuaPing 2010[752]	55	1638	0.03	[0.03; 0.04]
YeXiaoLing 2014[833]	27	264	0.10	[0.07; 0.15]
YuanJun 2014[854]	652	115469	0.01	[0.01; 0.01]
ZhangZhen 2015[903]	76	414	0.18	[0.15; 0.22]
ZhouXiaoHong 2010[960]	37	295	0.13	[0.09; 0.17]
Guo Z 2014[1001]	87	7141	0.01	[0.01; 0.02]
Zhou X 2012[1081]	108	5769	0.02	[0.02; 0.02]
**Common effect model**		**237341**	**0.01**	**[0.01; 0.01]**
**Random effects model**			**0.03**	**[0.02; 0.04]**
Heterogeneity: $I^2 = 99\%$, $\tau^2 = 1.4058$, $p = 0$				

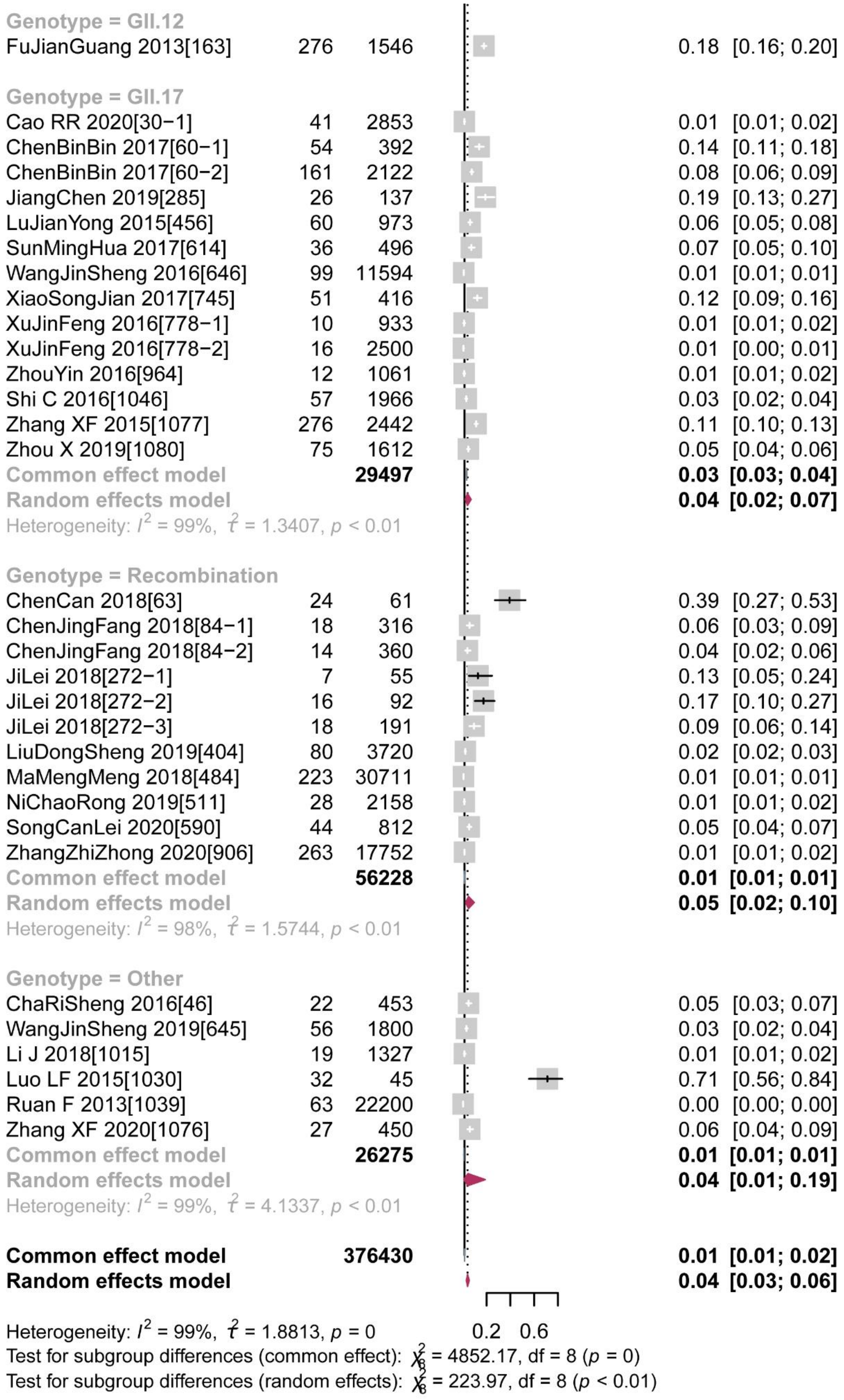
Genotype = GII.12
FuJianGuang 2013[163] 276 1546 0.18 [0.16; 0.20]
Genotype = GII.17
Cao RR 2020[30−1] 41 2853 0.01 [0.01; 0.02]
ChenBinBin 2017[60−1] 54 392 0.14 [0.11; 0.18]
ChenBinBin 2017[60−2] 161 2122 0.08 [0.06; 0.09]
JiangChen 2019[285] 26 137 0.19 [0.13; 0.27]
LuJianYong 2015[456] 60 973 0.06 [0.05; 0.08]
SunMingHua 2017[614] 36 496 0.07 [0.05; 0.10]
WangJinSheng 2016[646] 99 11594 0.01 [0.01; 0.01]
XiaoSongJian 2017[745] 51 416 0.12 [0.09; 0.16]
XuJinFeng 2016[778−1] 10 933 0.01 [0.01; 0.02]
XuJinFeng 2016[778−2] 16 2500 0.01 [0.00; 0.01]
ZhouYin 2016[964] 12 1061 0.01 [0.01; 0.02]
Shi C 2016[1046] 57 1966 0.03 [0.02; 0.04]
Zhang XF 2015[1077] 276 2442 0.11 [0.10; 0.13]
Zhou X 2019[1080] 75 1612 0.05 [0.04; 0.06]
Common effect model 29497 0.03 [0.03; 0.04]
Random effects model 0.04 [0.02; 0.07]
Heterogeneity: I^2 = 99%, τ^2 = 1.3407, p < 0.01
Genotype = Recombination
ChenCan 2018[63] 24 61 0.39 [0.27; 0.53]
ChenJingFang 2018[84−1] 18 316 0.06 [0.03; 0.09]
ChenJingFang 2018[84−2] 14 360 0.04 [0.02; 0.06]
JiLei 2018[272−1] 7 55 0.13 [0.05; 0.24]
JiLei 2018[272−2] 16 92 0.17 [0.10; 0.27]
JiLei 2018[272−3] 18 191 0.09 [0.06; 0.14]
LiuDongSheng 2019[404] 80 3720 0.02 [0.02; 0.03]
MaMengMeng 2018[484] 223 30711 0.01 [0.01; 0.01]
NiChaoRong 2019[511] 28 2158 0.01 [0.01; 0.02]
SongCanLei 2020[590] 44 812 0.05 [0.04; 0.07]
ZhangZhiZhong 2020[906] 263 17752 0.01 [0.01; 0.02]
Common effect model 56228 0.01 [0.01; 0.01]
Random effects model 0.05 [0.02; 0.10]
Heterogeneity: I^2 = 98%, τ^2 = 1.5744, p < 0.01
Genotype = Other
ChaRiSheng 2016[46] 22 453 0.05 [0.03; 0.07]
WangJinSheng 2019[645] 56 1800 0.03 [0.02; 0.04]
Li J 2018[1015] 19 1327 0.01 [0.01; 0.02]
Luo LF 2015[1030] 32 45 0.71 [0.56; 0.84]
Ruan F 2013[1039] 63 22200 0.00 [0.00; 0.00]
Zhang XF 2020[1076] 27 450 0.06 [0.04; 0.09]
Common effect model 26275 0.01 [0.01; 0.01]
Random effects model 0.04 [0.01; 0.19]
Heterogeneity: I^2 = 99%, τ^2 = 4.1337, p < 0.01
Common effect model 376430 0.01 [0.01; 0.02]
Random effects model 0.04 [0.03; 0.06]
0.2 0.6
Heterogeneity: I^2 = 99%, τ^2 = 1.8813, p = 0
Test for subgroup differences (common effect): χ^2_8 = 4852.17, df = 8 (p = 0)
Test for subgroup differences (random effects): χ^2_8 = 223.97, df = 8 (p < 0.01)

(E)

(e1)

Study Vomiting	Events	Total	Proportion	95%−CI	Weight (common)	Weight (random)
age = 0−4y						
Cai SX 2017[21]	17	17	1.00	[0.80; 1.00]	0.1%	0.3%
CaoXiaoPing 2018[33]	107	113	0.95	[0.89; 0.98]	0.4%	0.3%
ZengFengMei 2020[41]	24	25	0.96	[0.80; 1.00]	0.1%	0.3%
ZengLei 2018[44]	26	28	0.93	[0.76; 0.99]	0.1%	0.3%
ShenYiPing 2018[52]	18	26	0.69	[0.48; 0.86]	0.1%	0.3%
ChenJingFang 2018[84−1]	17	18	0.94	[0.73; 1.00]	0.1%	0.3%
ChenJingFang 2018[84−2]	12	14	0.86	[0.57; 0.98]	0.1%	0.3%
CuiXiaoShuang 2016[126]	85	137	0.62	[0.53; 0.70]	0.5%	0.3%
DaiYingXue 2018[129−1]	14	14	1.00	[0.77; 1.00]	0.1%	0.3%
DaiYingXue 2018[129−2]	12	12	1.00	[0.74; 1.00]	0.0%	0.3%
GaoHaiMing 2014[174]	19	19	1.00	[0.82; 1.00]	0.1%	0.3%
GaoZhiYong 2017[187−1]	10	12	0.83	[0.52; 0.98]	0.0%	0.3%
GuoMinJian 2017[208−1]	8	8	1.00	[0.63; 1.00]	0.0%	0.3%
GuoMinJian 2017[208−5]	17	17	1.00	[0.80; 1.00]	0.1%	0.3%
HuGuangYi 2017[232]	18	20	0.90	[0.68; 0.99]	0.1%	0.3%
JiLei 2018[272−2]	16	16	1.00	[0.79; 1.00]	0.1%	0.3%
LiChunLing 2020[319]	5	15	0.33	[0.12; 0.62]	0.1%	0.3%
LiangPing 2020[374]	21	25	0.84	[0.64; 0.95]	0.1%	0.3%
LiaoKeChang 2018[381]	40	41	0.98	[0.87; 1.00]	0.2%	0.3%
LinQin 2015[390]	29	34	0.85	[0.69; 0.95]	0.1%	0.3%
LuCaiFang 2020[458]	16	19	0.84	[0.60; 0.97]	0.1%	0.3%
MengXiangJie 2012[499]	14	16	0.88	[0.62; 0.98]	0.1%	0.3%
PangZhiFeng 2014[525]	20	20	1.00	[0.83; 1.00]	0.1%	0.3%
RenFuLin 2013[554]	41	43	0.95	[0.84; 0.99]	0.2%	0.3%
RenLiJun 2020[556]	9	9	1.00	[0.66; 1.00]	0.0%	0.3%
SunWenLong 2018[618]	8	8	1.00	[0.63; 1.00]	0.0%	0.3%
WangHua 2014[657]	28	28	1.00	[0.88; 1.00]	0.1%	0.3%
WangJun 2012[663]	16	17	0.94	[0.71; 1.00]	0.1%	0.3%
WangKaiLiang 2019[665]	16	16	1.00	[0.79; 1.00]	0.1%	0.3%
WangXiaoQin 2015[689]	11	11	1.00	[0.72; 1.00]	0.0%	0.3%
Ya−Xin Wang 2020[693]	13	14	0.93	[0.66; 1.00]	0.1%	0.3%
WeiGuiYing 2019[706]	27	29	0.93	[0.77; 0.99]	0.1%	0.3%
WuYang 2018[735]	91	91	1.00	[0.96; 1.00]	0.3%	0.3%
YangCheng 2017[797]	94	109	0.86	[0.78; 0.92]	0.4%	0.3%
YangJiChao 2016[802]	18	18	1.00	[0.81; 1.00]	0.1%	0.3%
YangRongXing 2019[808]	14	15	0.93	[0.68; 1.00]	0.1%	0.3%
YangSenPing 2018[810]	29	30	0.97	[0.83; 1.00]	0.1%	0.3%
YinHongMei 2014[839]	37	37	1.00	[0.91; 1.00]	0.1%	0.3%
YueYong 2017[859]	12	12	1.00	[0.74; 1.00]	0.0%	0.3%
ZhangGuangMing 2018[907]	12	14	0.86	[0.57; 0.98]	0.1%	0.3%
ZhangGuoBao 2020[908]	6	13	0.46	[0.19; 0.75]	0.0%	0.3%
ZhaoMeiLing 2019[918−1]	7	8	0.88	[0.47; 1.00]	0.0%	0.3%
ZhouJianHong 2013[954]	15	15	1.00	[0.78; 1.00]	0.1%	0.3%
ZhouLiHong 2016[956]	13	13	1.00	[0.75; 1.00]	0.0%	0.3%
ZhuChunXiu 2019[968]	17	17	1.00	[0.80; 1.00]	0.1%	0.3%
LiXiaoJing 2021[992]	7	7	1.00	[0.59; 1.00]	0.0%	0.2%
S Kim 2019[1012]	15	15	1.00	[0.78; 1.00]	0.1%	0.3%
Y Li 2021[1018]	133	143	0.93	[0.88; 0.97]	0.5%	0.3%
Zhang TL 2017[1075]	18	20	0.90	[0.68; 0.99]	0.1%	0.3%
Zou W 2015[1085]	270	383	0.70	[0.66; 0.75]	1.4%	0.3%
MaYan 2014[1118]	32	52	0.62	[0.47; 0.75]	0.2%	0.3%
SaiLinTao 2014[1120]	54	122	0.44	[0.35; 0.54]	0.5%	0.3%
**Common effect model**		**1975**	**0.87**	**[0.85; 0.89]**	**7.4%**	**--**
**Random effects model**			**0.93**	**[0.89; 0.96]**	**--**	**15.6%**

Heterogeneity: $I^2 = 88\%$, $\tau^2 = 0.0363$, $p < 0.01$

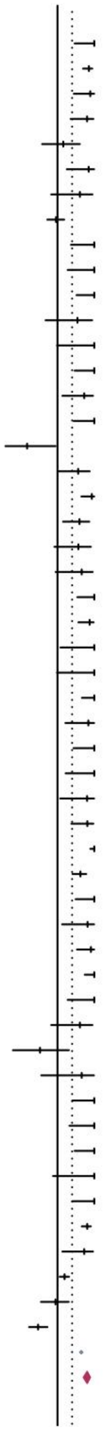

age = 5−17y						
BaiAiLi 2020[3]	11	20	0.55	[0.32; 0.77]	0.1%	0.3%
CaiWenFeng 2014[26]	27	27	1.00	[0.87; 1.00]	0.1%	0.3%
ShenJiChuan 2011[50]	204	312	0.65	[0.60; 0.71]	1.2%	0.3%
ShenYiPing 2013[53]	57	65	0.88	[0.77; 0.95]	0.2%	0.3%
ShenYuGang 2016[54]	29	43	0.67	[0.51; 0.81]	0.2%	0.3%
ChenCaiRong 2020[62]	16	16	1.00	[0.79; 1.00]	0.1%	0.3%
ChenChun 2014[64]	102	107	0.95	[0.89; 0.98]	0.4%	0.3%
ChenJian 2016[78]	6	20	0.30	[0.12; 0.54]	0.1%	0.3%
ChenJian 2017[80]	56	69	0.81	[0.70; 0.90]	0.3%	0.3%
ChenQuan 2021[96]	46	84	0.55	[0.44; 0.66]	0.3%	0.3%
ChenXiaoFeng 2015[100]	7	7	1.00	[0.59; 1.00]	0.0%	0.2%
ChenXingHong 2012[102]	328	478	0.69	[0.64; 0.73]	1.8%	0.3%
ChenXingFu 2018[103]	41	43	0.95	[0.84; 0.99]	0.2%	0.3%
ChenYiXiong 2018[108]	21	21	1.00	[0.84; 1.00]	0.1%	0.3%
CuiXiaoMan 2018[125]	16	17	0.94	[0.71; 1.00]	0.1%	0.3%
DaiBenNa 2020[127]	115	117	0.98	[0.94; 1.00]	0.4%	0.3%
DaiYingXue 2018[129−3]	18	18	1.00	[0.81; 1.00]	0.1%	0.3%
DaiYingXue 2018[129−4]	13	14	0.93	[0.66; 1.00]	0.1%	0.3%
Ying−Hui Deng 2014[135]	71	99	0.72	[0.62; 0.80]	0.4%	0.3%
DuYueHe 2019[148]	26	29	0.90	[0.73; 0.98]	0.1%	0.3%
FangYuLian 2019[155]	126	241	0.52	[0.46; 0.59]	0.9%	0.3%
FangYuLian 2021[156]	524	809	0.65	[0.61; 0.68]	3.0%	0.3%
FengZhi 2018[162]	96	111	0.86	[0.79; 0.92]	0.4%	0.3%
FuXiaoFei 2012[171]	17	20	0.85	[0.62; 0.97]	0.1%	0.3%
GanXiangYang 2014[172]	47	74	0.64	[0.52; 0.74]	0.3%	0.3%
GaoHuiJuan 2015[176]	18	18	1.00	[0.81; 1.00]	0.1%	0.3%
GaoJunYing 2017[177]	12	12	1.00	[0.74; 1.00]	0.0%	0.3%
GaoPeng 2020[181]	25	28	0.89	[0.72; 0.98]	0.1%	0.3%
GaoZhiYong 2017[187−2]	8	10	0.80	[0.44; 0.97]	0.0%	0.3%
GaoZhiYong 2017[187−4]	13	16	0.81	[0.54; 0.96]	0.1%	0.3%
GaoZhiYong 2017[187−6]	22	25	0.88	[0.69; 0.97]	0.1%	0.3%
GongLiQiang 2013[192]	121	139	0.87	[0.80; 0.92]	0.5%	0.3%
GongShuiYing 2017[194]	88	93	0.95	[0.88; 0.98]	0.3%	0.3%
GuYiFu 2020[197]	44	45	0.98	[0.88; 1.00]	0.2%	0.3%
GuoJing 2019[203]	121	193	0.63	[0.55; 0.70]	0.7%	0.3%
GuoMinJian 2017[208−2]	5	6	0.83	[0.36; 1.00]	0.0%	0.2%
GuoMinJian 2017[208−3]	11	11	1.00	[0.72; 1.00]	0.0%	0.3%
GuoMinJian 2017[208−4]	8	10	0.80	[0.44; 0.97]	0.0%	0.3%
GuoMinJian 2017[208−6]	7	7	1.00	[0.59; 1.00]	0.0%	0.2%
GuoMinJian 2017[208−7]	7	7	1.00	[0.59; 1.00]	0.0%	0.2%
GuoMinJian 2017[208−9]	10	16	0.62	[0.35; 0.85]	0.1%	0.3%
GuoMinJian 2017[208−10]	44	48	0.92	[0.80; 0.98]	0.2%	0.3%
HeXuXin 2017[222]	93	156	0.60	[0.51; 0.67]	0.6%	0.3%
HeHanZhen 2014[224]	9	76	0.12	[0.06; 0.21]	0.3%	0.3%
HouYuYuan 2014[231]	39	63	0.62	[0.49; 0.74]	0.2%	0.3%
HuHongAn 2015[233]	37	105	0.35	[0.26; 0.45]	0.4%	0.3%
Ying Hu 2019[239]	42	120	0.35	[0.27; 0.44]	0.4%	0.3%
HuaWeiYu 2018[241]	63	63	1.00	[0.94; 1.00]	0.2%	0.3%
HuangBinBin 2020[245]	20	41	0.49	[0.33; 0.65]	0.2%	0.3%
HuangGuo 2015[252]	68	69	0.99	[0.92; 1.00]	0.3%	0.3%
HuangYanHong 2019[260]	65	84	0.77	[0.67; 0.86]	0.3%	0.3%
HuangYanHong 2019[261]	83	86	0.97	[0.90; 0.99]	0.3%	0.3%
Lei Ji 2021[269]	26	26	1.00	[0.87; 1.00]	0.1%	0.3%
JiLei 2018[272−1]	7	7	1.00	[0.59; 1.00]	0.0%	0.2%

JiLei 2018[272−3]	13	18	0.72	[0.47; 0.90]	0.1%	0.3%
JiRuPing 2020[274]	112	119	0.94	[0.88; 0.98]	0.4%	0.3%
JiangXianChen 2014[283−1]	5	16	0.31	[0.11; 0.59]	0.1%	0.3%
JiangXianChen 2014[283−2]	23	23	1.00	[0.85; 1.00]	0.1%	0.3%
JiangYingCi 2015[284]	22	66	0.33	[0.22; 0.46]	0.2%	0.3%
JiangYiMei 2017[294]	15	15	1.00	[0.78; 1.00]	0.1%	0.3%
KangQian 2020[303−1]	31	35	0.89	[0.73; 0.97]	0.1%	0.3%
KangQian 2020[303−2]	40	50	0.80	[0.66; 0.90]	0.2%	0.3%
LiBo 2013[316]	6	120	0.05	[0.02; 0.11]	0.4%	0.3%
LiCaiYun 2012[318]	15	147	0.10	[0.06; 0.16]	0.5%	0.3%
LiShiCong 2020[344]	134	181	0.74	[0.67; 0.80]	0.7%	0.3%
LiShiCong 2018[346]	43	64	0.67	[0.54; 0.78]	0.2%	0.3%
LiShiE 2018[348]	55	92	0.60	[0.49; 0.70]	0.3%	0.3%
LiShouJun 2016[350]	56	64	0.88	[0.77; 0.94]	0.2%	0.3%
LiXiTai 2015[352]	51	51	1.00	[0.93; 1.00]	0.2%	0.3%
LiYueRong 2015[366]	18	18	1.00	[0.81; 1.00]	0.1%	0.3%
LiangRiCheng 2017[376]	20	27	0.74	[0.54; 0.89]	0.1%	0.3%
LiaoChan 2021[380]	10	19	0.53	[0.29; 0.76]	0.1%	0.3%
LinQiFeng 2018[388]	85	96	0.89	[0.80; 0.94]	0.4%	0.3%
LinQingShuang 2016[391]	28	29	0.97	[0.82; 1.00]	0.1%	0.3%
LiuBaiWei 2017[395]	24	24	1.00	[0.86; 1.00]	0.1%	0.3%
LiuBo 2015[398]	93	104	0.89	[0.82; 0.95]	0.4%	0.3%
LiuDan 2019[403]	87	90	0.97	[0.91; 0.99]	0.3%	0.3%
LiuDongSheng 2019[404]	68	80	0.85	[0.75; 0.92]	0.3%	0.3%
LiuHongLian 2018[411]	16	19	0.84	[0.60; 0.97]	0.1%	0.3%
LiuQingLian 2019[420]	37	37	1.00	[0.91; 1.00]	0.1%	0.3%
LiuShiKe 2012[421]	127	306	0.42	[0.36; 0.47]	1.1%	0.3%
LiuShiKe 2016[422]	39	46	0.85	[0.71; 0.94]	0.2%	0.3%
LiuYing 2019[437]	14	14	1.00	[0.77; 1.00]	0.1%	0.3%
LiuYuan 2016[438−2]	121	121	1.00	[0.97; 1.00]	0.4%	0.3%
LuoLe 2017[472]	31	39	0.79	[0.64; 0.91]	0.1%	0.3%
MaTao 2018[487]	40	46	0.87	[0.74; 0.95]	0.2%	0.3%
MaoJianYing 2016[493]	51	51	1.00	[0.93; 1.00]	0.2%	0.3%
MoYuJie 2018[505]	17	19	0.89	[0.67; 0.99]	0.1%	0.3%
NiChaoRong 2019[510]	114	125	0.91	[0.85; 0.96]	0.5%	0.3%
NiChaoRong 2019[511]	25	28	0.89	[0.72; 0.98]	0.1%	0.3%
NiChunYan 2020[512]	40	55	0.73	[0.59; 0.84]	0.2%	0.3%
OuSheXiang 2019[515]	18	18	1.00	[0.81; 1.00]	0.1%	0.3%
PangZhiFeng 2017[524]	60	79	0.76	[0.65; 0.85]	0.3%	0.3%
PangZhiMing 2015[526]	19	19	1.00	[0.82; 1.00]	0.1%	0.3%
PengXiaoXue 2015[528]	32	37	0.86	[0.71; 0.95]	0.1%	0.3%
QiYanQiu 2018[532]	27	27	1.00	[0.87; 1.00]	0.1%	0.3%
QiaoYingQin 2016[543]	36	67	0.54	[0.41; 0.66]	0.2%	0.3%
QiuHaiYan 2013[551]	31	76	0.41	[0.30; 0.53]	0.3%	0.3%
RenFuLin 2013[555]	59	74	0.80	[0.69; 0.88]	0.3%	0.3%
ShiChao 2013[579]	234	462	0.51	[0.46; 0.55]	1.7%	0.3%
ShiYongLin 2015[587]	11	19	0.58	[0.33; 0.80]	0.1%	0.3%
ShuaiHuiQun 2012[589]	11	14	0.79	[0.49; 0.95]	0.1%	0.3%
SongCanLei 2020[590]	31	44	0.70	[0.55; 0.83]	0.2%	0.3%
SongCanLei 2017[593]	35	36	0.97	[0.85; 1.00]	0.1%	0.3%
SongHuiRong 2017[597]	29	34	0.85	[0.69; 0.95]	0.1%	0.3%
SongYuFang 2019[600]	55	63	0.87	[0.77; 0.94]	0.2%	0.3%
SuTong 2020[604]	51	55	0.93	[0.82; 0.98]	0.2%	0.3%
SunQin 2019[616]	32	32	1.00	[0.89; 1.00]	0.1%	0.3%
TangYuHuan 2017[630]	43	44	0.98	[0.88; 1.00]	0.2%	0.3%
TaoLiYan 2020[636]	37	47	0.79	[0.64; 0.89]	0.2%	0.3%
TianYaLin 2021[642]	27	38	0.71	[0.54; 0.85]	0.1%	0.3%
WangJinSheng 2019[645]	51	56	0.91	[0.80; 0.97]	0.2%	0.3%

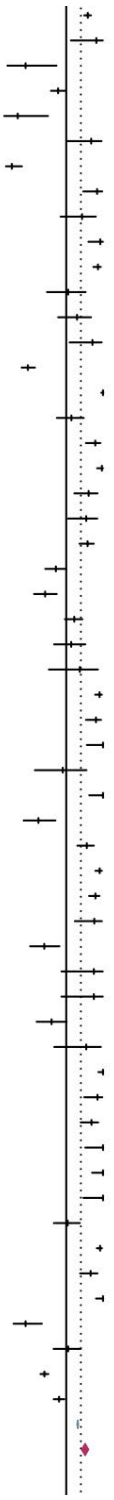

Study	Events	Total	Proportion [95% CI]	Weight (common)	Weight (random)
WangJie 2015[659]	383	451	0.85 [0.81; 0.88]	1.7%	0.3%
WangTieJun 2020[678]	14	15	0.93 [0.68; 1.00]	0.1%	0.3%
WangWeiRu 2013[680]	3	13	0.23 [0.05; 0.54]	0.0%	0.3%
WangWenYing 2011[681]	113	204	0.55 [0.48; 0.62]	0.8%	0.3%
WangXiaoYi 2017[691]	2	13	0.15 [0.02; 0.45]	0.0%	0.3%
WuGuoFu 2018[717]	15	17	0.88 [0.64; 0.99]	0.1%	0.3%
WuHongXing 2016[718]	6	63	0.10 [0.04; 0.20]	0.2%	0.3%
WuJingWen 2020[719]	32	34	0.94 [0.80; 0.99]	0.1%	0.3%
WuQinDi 2018[725]	19	24	0.79 [0.58; 0.93]	0.1%	0.3%
WuWenQian 2018[729]	35	36	0.97 [0.85; 1.00]	0.1%	0.3%
WuXiaoMin 2021[732]	151	159	0.95 [0.90; 0.98]	0.6%	0.3%
WuYang 2018[734]	17	26	0.65 [0.44; 0.83]	0.1%	0.3%
WuYiLing 2017[736]	23	31	0.74 [0.55; 0.88]	0.1%	0.3%
WuZhenYu 2012[738]	17	19	0.89 [0.67; 0.99]	0.1%	0.3%
XiaYingPin 2018[743]	45	177	0.25 [0.19; 0.33]	0.7%	0.3%
XiaZhongFa 2012[744]	209	209	1.00 [0.98; 1.00]	0.8%	0.3%
XiaoSongJian 2017[745]	35	51	0.69 [0.54; 0.81]	0.2%	0.3%
XieBin 2020[758]	60	65	0.92 [0.83; 0.97]	0.2%	0.3%
XuJianRong 2018[767]	91	92	0.99 [0.94; 1.00]	0.3%	0.3%
YangCaiBin 2018[796]	36	42	0.86 [0.71; 0.95]	0.2%	0.3%
YangJiXing 2019[801]	25	30	0.83 [0.65; 0.94]	0.1%	0.3%
YangJing 2018[804]	94	111	0.85 [0.77; 0.91]	0.4%	0.3%
YeXianMing 2017[832]	50	94	0.53 [0.43; 0.64]	0.3%	0.3%
YuHong 2016[848]	34	80	0.42 [0.32; 0.54]	0.3%	0.3%
Yan Yu 2017[852]	80	112	0.71 [0.62; 0.80]	0.4%	0.3%
ZhanYueWang 2019[860]	26	38	0.68 [0.51; 0.82]	0.1%	0.3%
ZhangChong 2015[862]	10	13	0.77 [0.46; 0.95]	0.0%	0.3%
ZhangHaiYan 2019[867]	140	145	0.97 [0.92; 0.99]	0.5%	0.3%
ZhangQin 2020[885]	53	57	0.93 [0.83; 0.98]	0.2%	0.3%
ZhangXiaoYi 2015[891]	21	21	1.00 [0.84; 1.00]	0.1%	0.3%
ZhangYanMing 2019[898]	9	15	0.60 [0.32; 0.84]	0.1%	0.3%
ZhangZhengDong 2017[904]	25	25	1.00 [0.86; 1.00]	0.1%	0.3%
ZhaoMeiLing 2019[918−2]	14	39	0.36 [0.21; 0.53]	0.1%	0.3%
ZhaoMengJiao 2018[919]	73	87	0.84 [0.74; 0.91]	0.3%	0.3%
ZhaoXueCheng 2016[929]	144	149	0.97 [0.92; 0.99]	0.6%	0.3%
ZhaoYuLi 2019[933]	110	119	0.92 [0.86; 0.96]	0.4%	0.3%
ZhenGuoXin 2020[936]	21	23	0.91 [0.72; 0.99]	0.1%	0.3%
HuangJiaCheng 2021[944]	20	48	0.42 [0.28; 0.57]	0.2%	0.3%
ZhongJianMing 2014[946]	10	11	0.91 [0.59; 1.00]	0.0%	0.3%
Jun−Hua Zhou 2018[955]	10	11	0.91 [0.59; 1.00]	0.0%	0.3%
ZhouXiaoHong 2017[961]	23	47	0.49 [0.34; 0.64]	0.2%	0.3%
ZhouYin 2016[964]	10	12	0.83 [0.52; 0.98]	0.0%	0.3%
ZhuChengMing 2017[967]	78	78	1.00 [0.95; 1.00]	0.3%	0.3%
HuangKaiXiong 2021[989]	34	36	0.94 [0.81; 0.99]	0.1%	0.3%
Guo XH 2018[1000]	54	61	0.89 [0.78; 0.95]	0.2%	0.3%
Li J 2018[1015]	19	19	1.00 [0.82; 1.00]	0.1%	0.3%
Luo LF 2015[1030]	32	32	1.00 [0.89; 1.00]	0.1%	0.3%
WuLin 2021[1035]	17	17	1.00 [0.80; 1.00]	0.1%	0.3%
Shi C 2016[1046]	37	57	0.65 [0.51; 0.77]	0.2%	0.3%
Xu H 2013[1059−1]	201	207	0.97 [0.94; 0.99]	0.8%	0.3%
Xu H 2013[1059−2]	57	65	0.88 [0.77; 0.95]	0.2%	0.3%
XiangLunHui 2016[1101]	51	51	1.00 [0.93; 1.00]	0.2%	0.3%
ZouYong 2011[1103]	9	39	0.23 [0.11; 0.39]	0.1%	0.3%
ChenCen 2016[1105]	34	52	0.65 [0.51; 0.78]	0.2%	0.3%
HeFei 2020[1108]	255	610	0.42 [0.38; 0.46]	2.2%	0.3%
LiuYun 2019[1116]	199	353	0.56 [0.51; 0.62]	1.3%	0.3%
**Common effect model**		**12941**	**0.75 [0.74; 0.76]**	**48.0%**	**--**
**Random effects model**			**0.82 [0.79; 0.86]**	**--**	**53.3%**

Heterogeneity: I^2 = 96%, τ^2 = 0.0789, p = 0

age = 18−60y						
Cai MW 2018[18]	22	34	0.65	[0.46; 0.80]	0.1%	0.3%
CaiWenFeng 2013[25]	80	141	0.57	[0.48; 0.65]	0.5%	0.3%
CaoShen 2019[32]	41	62	0.66	[0.53; 0.78]	0.2%	0.3%
ShenYuGang 2016[55]	161	233	0.69	[0.63; 0.75]	0.9%	0.3%
ChenAQun 2016[59]	28	64	0.44	[0.31; 0.57]	0.2%	0.3%
ChenGuoCui 2011[69]	20	27	0.74	[0.54; 0.89]	0.1%	0.3%
ChenHeJuan 2018[73]	12	14	0.86	[0.57; 0.98]	0.1%	0.3%
ChenJianMei 2017[79]	52	110	0.47	[0.38; 0.57]	0.4%	0.3%
ChenYiYi 2015[109]	98	282	0.35	[0.29; 0.41]	1.0%	0.3%
DuYao 2013[150]	31	157	0.20	[0.14; 0.27]	0.6%	0.3%
FengZhi 2020[161]	14	26	0.54	[0.33; 0.73]	0.1%	0.3%
GaoRiHong 2019[182]	17	30	0.57	[0.37; 0.75]	0.1%	0.3%
GaoShuPing 2019[183]	34	51	0.67	[0.52; 0.79]	0.2%	0.3%
GaoZhiYong 2017[187−3]	14	15	0.93	[0.68; 1.00]	0.1%	0.3%
GaoZhiYong 2017[187−8]	22	22	1.00	[0.85; 1.00]	0.1%	0.3%
GuKaiChen 2020[195]	13	21	0.62	[0.38; 0.82]	0.1%	0.3%
GuoLi 2019[205]	59	75	0.79	[0.68; 0.87]	0.3%	0.3%
HaoYongJian 2020[216]	7	25	0.28	[0.12; 0.49]	0.1%	0.3%
HuangGuo 2015[253]	69	87	0.79	[0.69; 0.87]	0.3%	0.3%
HuangLiQing 2020[254]	93	184	0.51	[0.43; 0.58]	0.7%	0.3%
HuangSiYue 2020[257]	42	90	0.47	[0.36; 0.57]	0.3%	0.3%
JiJinHua 2018[265]	11	41	0.27	[0.14; 0.43]	0.2%	0.3%
JiLei 2020[270]	12	19	0.63	[0.38; 0.84]	0.1%	0.3%
JiangChen 2019[285]	20	26	0.77	[0.56; 0.91]	0.1%	0.3%
JiangLie 2014[291]	7	15	0.47	[0.21; 0.73]	0.1%	0.3%
KuangHaoCheng 2016[307]	379	667	0.57	[0.53; 0.61]	2.5%	0.3%
LaiShiMing 2014[308]	53	105	0.50	[0.41; 0.60]	0.4%	0.3%
LiDaiBo 2018[320]	11	18	0.61	[0.36; 0.83]	0.1%	0.3%
LiJie 2019[329]	78	106	0.74	[0.64; 0.82]	0.4%	0.3%
LiQun 2010[340]	50	258	0.19	[0.15; 0.25]	1.0%	0.3%
LiShiE 2018[349]	68	77	0.88	[0.79; 0.95]	0.3%	0.3%
LiXiuFang 2018[359]	9	20	0.45	[0.23; 0.68]	0.1%	0.3%
LiYiLan 2014[365]	148	228	0.65	[0.58; 0.71]	0.8%	0.3%
LiuJingJing 2018[413]	12	33	0.36	[0.20; 0.55]	0.1%	0.3%
LiuKaiQian 2011[415]	126	634	0.20	[0.17; 0.23]	2.3%	0.3%
LiuXiaoXiao 2014[430]	11	18	0.61	[0.36; 0.83]	0.1%	0.3%
LiuYi 2013[436]	7	9	0.78	[0.40; 0.97]	0.0%	0.3%
LuZhengXiang 2016[451]	56	83	0.67	[0.56; 0.77]	0.3%	0.3%
LuJianYong 2015[456]	27	60	0.45	[0.32; 0.58]	0.2%	0.3%
LuWeiWei 2016[462]	118	406	0.29	[0.25; 0.34]	1.5%	0.3%
MaMengMeng 2018[484]	124	223	0.56	[0.49; 0.62]	0.8%	0.3%
PanYiFeng 2017[521]	61	82	0.74	[0.64; 0.83]	0.3%	0.3%
QiXiaoQi 2019[534]	143	185	0.77	[0.71; 0.83]	0.7%	0.3%
QiYing 2018[535−1]	105	173	0.61	[0.53; 0.68]	0.6%	0.3%
QiYing 2018[535−2]	29	71	0.41	[0.29; 0.53]	0.3%	0.3%
QianLiZhen 2020[537]	18	23	0.78	[0.56; 0.93]	0.1%	0.3%
RenYuHua 2016[562]	43	110	0.39	[0.30; 0.49]	0.4%	0.3%
Hao−Yu Shi 2019[582]	3	9	0.33	[0.07; 0.70]	0.0%	0.3%
SunJing 2015[611]	55	92	0.60	[0.49; 0.70]	0.3%	0.3%
SunZhou 2016[625]	104	230	0.45	[0.39; 0.52]	0.8%	0.3%
WangBing 2017[649]	44	58	0.76	[0.63; 0.86]	0.2%	0.3%
WangDaHu 2020[652]	15	182	0.08	[0.05; 0.13]	0.7%	0.3%
WangHu 2018[656]	33	72	0.46	[0.34; 0.58]	0.3%	0.3%
WangMan 2017[668]	25	85	0.29	[0.20; 0.40]	0.3%	0.3%

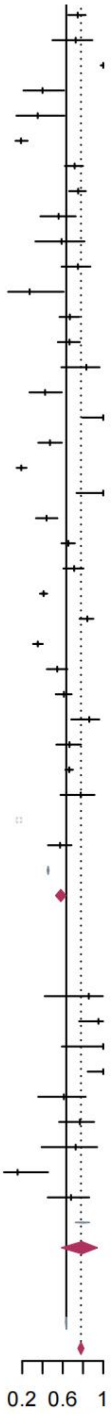

WangMingLiang 2020[671]	80	107	0.75 [0.65; 0.83]	0.4%	0.3%
WeiYiYun 2015[705]	16	22	0.73 [0.50; 0.89]	0.1%	0.3%
WuPei 2011[724]	146	146	1.00 [0.98; 1.00]	0.5%	0.3%
WuShuiXin 2012[727]	10	25	0.40 [0.21; 0.61]	0.1%	0.3%
WuZhaoChun 2015[737]	6	17	0.35 [0.14; 0.62]	0.1%	0.3%
WuZhiSheng 2014[741]	33	176	0.19 [0.13; 0.25]	0.6%	0.3%
XiaGuangHui 2019[742]	76	106	0.72 [0.62; 0.80]	0.4%	0.3%
Xiao−Bo Xie 2020[754]	85	113	0.75 [0.66; 0.83]	0.4%	0.3%
XieYuanQi 2016[756]	19	34	0.56 [0.38; 0.73]	0.1%	0.3%
XuanLingFeng 2014[784]	10	17	0.59 [0.33; 0.82]	0.1%	0.3%
YangAiQing 2018[795]	30	40	0.75 [0.59; 0.87]	0.1%	0.3%
YangZhiWen 2013[820]	3	11	0.27 [0.06; 0.61]	0.0%	0.3%
ZhangDongSheng 2013[864]	65	97	0.67 [0.57; 0.76]	0.4%	0.3%
ZhangHaiYan 2016[868]	52	78	0.67 [0.55; 0.77]	0.3%	0.3%
ZhangHengQiu 2014[869]	15	18	0.83 [0.59; 0.96]	0.1%	0.3%
ZhangHuiLing 2018[871]	17	40	0.42 [0.27; 0.59]	0.1%	0.3%
ZhangPan 2020[883]	16	16	1.00 [0.79; 1.00]	0.1%	0.3%
ZhangZhen 2015[903]	36	76	0.47 [0.36; 0.59]	0.3%	0.3%
ZhangZhiZhong 2020[906]	50	263	0.19 [0.14; 0.24]	1.0%	0.3%
ZhouGuoYing 2016[952]	12	12	1.00 [0.74; 1.00]	0.0%	0.3%
ZhuWeiWei 2018[976]	40	91	0.44 [0.34; 0.55]	0.3%	0.3%
Chen D 2019[987]	137	209	0.66 [0.59; 0.72]	0.8%	0.3%
Guo Z 2014[1001]	62	87	0.71 [0.61; 0.80]	0.3%	0.3%
Huang XY 2017[1007]	309	753	0.41 [0.37; 0.45]	2.8%	0.3%
Li JS 2016[1017−2]	102	121	0.84 [0.77; 0.90]	0.4%	0.3%
Y Li 2013[1019]	140	396	0.35 [0.31; 0.40]	1.5%	0.3%
Liu Y 2015[1025]	54	99	0.55 [0.44; 0.65]	0.4%	0.3%
Lu Y 2020[1029]	96	157	0.61 [0.53; 0.69]	0.6%	0.3%
Qin M 2016[1037]	25	29	0.86 [0.68; 0.96]	0.1%	0.3%
Ruan F 2013[1039]	42	63	0.67 [0.54; 0.78]	0.2%	0.3%
Zhang L 2018[1071]	419	631	0.66 [0.63; 0.70]	2.3%	0.3%
Zhang XF 2020[1076]	21	27	0.78 [0.58; 0.91]	0.1%	0.3%
Zhou N 2016[1079]	270	1614	0.17 [0.15; 0.19]	5.9%	0.3%
Zhou X 2019[1080]	43	75	0.57 [0.45; 0.69]	0.3%	0.3%
**Common effect model**		**11934**	**0.46 [0.45; 0.46]**	**44.1%**	**--**
**Random effects model**			**0.58 [0.53; 0.63]**	**--**	**28.5%**
Heterogeneity: $I^2 = 97\%$, $\tau^2 = 0.0525$, $p = 0$					
age≥60y					
GaoZhiYong 2017[187−5]	6	7	0.86 [0.42; 1.00]	0.0%	0.2%
GaoZhiYong 2017[187−7]	20	21	0.95 [0.76; 1.00]	0.1%	0.3%
GuoMinJian 2017[208−8]	7	7	1.00 [0.59; 1.00]	0.0%	0.2%
QianZiYu 2012[539]	22	22	1.00 [0.85; 1.00]	0.1%	0.3%
SongCanLei 2012[591]	11	18	0.61 [0.36; 0.83]	0.1%	0.3%
SongCanLei 2013[594]	20	26	0.77 [0.56; 0.91]	0.1%	0.3%
SunLiYan 2020[613]	8	11	0.73 [0.39; 0.94]	0.0%	0.3%
WangXiaoYi 2017[690]	2	13	0.15 [0.02; 0.45]	0.0%	0.3%
XuFeng 2012[774]	15	22	0.68 [0.45; 0.86]	0.1%	0.3%
**Common effect model**		**147**	**0.80 [0.72; 0.86]**	**0.6%**	**--**
**Random effects model**			**0.79 [0.59; 0.94]**	**--**	**2.6%**
Heterogeneity: $I^2 = 83\%$, $\tau^2 = 0.0789$, $p < 0.01$					
**Common effect model**		**26997**	**0.63 [0.63; 0.64]**	**100.0%**	**--**
**Random effects model**			**0.78 [0.75; 0.81]**	**--**	**100.0%**

Heterogeneity: $I^2 = 97\%$, $\tau^2 = 0.0832$, $p = 0$

Test for subgroup differences (common effect): $\chi^2_3 = 2779.23$, df = 3 ($p = 0$)

Test for subgroup differences (random effects): $\chi^2_3 = 109.55$, df = 3 ($p < 0.01$)

(e2)

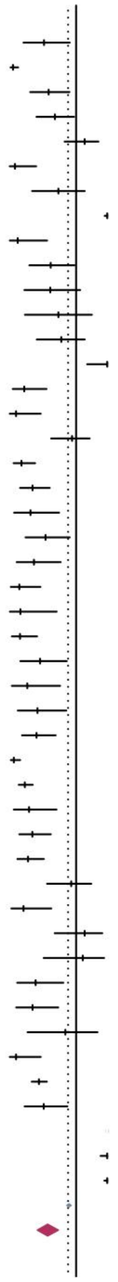

Study Diarrhea	Events	Total	Proportion	95%−CI	Weight (common)	Weight (random)
age = 0−4y						
Cai SX 2017[21]	6	17	0.35	[0.14; 0.62]	0.1%	0.3%
CaoXiaoPing 2018[33]	4	113	0.04	[0.01; 0.09]	0.4%	0.3%
ZengFengMei 2020[41]	10	25	0.40	[0.21; 0.61]	0.1%	0.3%
ZengLei 2018[44]	13	28	0.46	[0.28; 0.66]	0.1%	0.3%
ShenYiPing 2018[52]	20	26	0.77	[0.56; 0.91]	0.1%	0.3%
ChenJingFang 2018[84−1]	1	18	0.06	[0.00; 0.27]	0.1%	0.3%
ChenJingFang 2018[84−2]	7	14	0.50	[0.23; 0.77]	0.1%	0.3%
CuiXiaoShuang 2016[126]	137	137	1.00	[0.97; 1.00]	0.5%	0.3%
DaiYingXue 2018[129−2]	1	12	0.08	[0.00; 0.38]	0.0%	0.3%
GaoHaiMing 2014[174]	8	19	0.42	[0.20; 0.67]	0.1%	0.3%
GaoZhiYong 2017[187−1]	5	12	0.42	[0.15; 0.72]	0.0%	0.3%
GuoMinJian 2017[208−1]	4	8	0.50	[0.16; 0.84]	0.0%	0.3%
GuoMinJian 2017[208−5]	9	17	0.53	[0.28; 0.77]	0.1%	0.3%
GuoShuiLian 2014[209]	16	16	1.00	[0.79; 1.00]	0.1%	0.3%
HuGuangYi 2017[232]	3	20	0.15	[0.03; 0.38]	0.1%	0.3%
LiChunLing 2020[319]	1	15	0.07	[0.00; 0.32]	0.1%	0.3%
LiangPing 2020[374]	16	25	0.64	[0.43; 0.82]	0.1%	0.3%
LiaoKeChang 2018[381]	5	41	0.12	[0.04; 0.26]	0.2%	0.3%
LinQin 2015[390]	8	34	0.24	[0.11; 0.41]	0.1%	0.3%
LuHua 2016[454]	3	14	0.21	[0.05; 0.51]	0.1%	0.3%
LuCaiFang 2020[458]	7	19	0.37	[0.16; 0.62]	0.1%	0.3%
MengXiangJie 2012[499]	4	16	0.25	[0.07; 0.52]	0.1%	0.3%
PangZhiFeng 2014[525]	2	20	0.10	[0.01; 0.32]	0.1%	0.3%
RenLiJun 2020[556]	1	9	0.11	[0.00; 0.48]	0.0%	0.3%
WangHua 2014[657]	3	28	0.11	[0.02; 0.28]	0.1%	0.3%
WangKaiLiang 2019[665]	5	16	0.31	[0.11; 0.59]	0.1%	0.3%
WangXiaoQin 2015[689]	2	11	0.18	[0.02; 0.52]	0.0%	0.3%
Ya−Xin Wang 2020[693]	4	14	0.29	[0.08; 0.58]	0.1%	0.3%
WeiGuiYing 2019[706]	8	29	0.28	[0.13; 0.47]	0.1%	0.3%
WuYang 2018[735]	4	91	0.04	[0.01; 0.11]	0.3%	0.3%
YangCheng 2017[797]	17	109	0.16	[0.09; 0.24]	0.4%	0.3%
YangRongXing 2019[808]	3	15	0.20	[0.04; 0.48]	0.1%	0.3%
YangSenPing 2018[810]	7	30	0.23	[0.10; 0.42]	0.1%	0.3%
YinHongMei 2014[839]	7	37	0.19	[0.08; 0.35]	0.1%	0.3%
ZhangAiHua 2018[861]	12	19	0.63	[0.38; 0.84]	0.1%	0.3%
ZhangGuangMing 2018[907]	2	14	0.14	[0.02; 0.43]	0.1%	0.3%
ZhangGuoBao 2020[908]	10	13	0.77	[0.46; 0.95]	0.1%	0.3%
ZhaoMeiLing 2019[918−1]	6	8	0.75	[0.35; 0.97]	0.0%	0.3%
ZhouJianHong 2013[954]	4	15	0.27	[0.08; 0.55]	0.1%	0.3%
ZhuChunXiu 2019[968]	4	17	0.24	[0.07; 0.50]	0.1%	0.3%
LiXiaoJing 2021[992]	4	7	0.57	[0.18; 0.90]	0.0%	0.3%
S Kim 2019[1012]	1	15	0.07	[0.00; 0.32]	0.1%	0.3%
Y Li 2021[1018]	43	143	0.30	[0.23; 0.38]	0.5%	0.3%
Zhang TL 2017[1075]	7	20	0.35	[0.15; 0.59]	0.1%	0.3%
Zou W 2015[1085]	383	383	1.00	[0.99; 1.00]	1.4%	0.3%
MaYan 2014[1118]	52	52	1.00	[0.93; 1.00]	0.2%	0.3%
SaiLinTao 2014[1120]	122	122	1.00	[0.97; 1.00]	0.5%	0.3%
Common effect model		1883	0.61	[0.59; 0.63]	7.1%	--
Random effects model			0.39	[0.28; 0.50]	--	14.9%

Heterogeneity: $I^2 = 98\%$, $\tau^2 = 0.1269$, $p = 0$

age = 5−17y

BaiAiLi 2020[3]	13	20	0.65	[0.41; 0.85]	0.1%	0.3%
CaiWenFeng 2014[26]	6	27	0.22	[0.09; 0.42]	0.1%	0.3%
ShenJiChuan 2011[50]	258	312	0.83	[0.78; 0.87]	1.2%	0.3%
ShenYiPing 2013[53]	15	65	0.23	[0.14; 0.35]	0.2%	0.3%
ShenYuGang 2016[54]	30	43	0.70	[0.54; 0.83]	0.2%	0.3%
ChenCaiRong 2020[62]	2	16	0.12	[0.02; 0.38]	0.1%	0.3%
ChenChun 2014[64]	29	107	0.27	[0.19; 0.37]	0.4%	0.3%
ChenJian 2016[78]	4	20	0.20	[0.06; 0.44]	0.1%	0.3%
ChenJian 2017[80]	55	69	0.80	[0.68; 0.88]	0.3%	0.3%
ChenQuan 2021[96]	59	84	0.70	[0.59; 0.80]	0.3%	0.3%
ChenXiaoFeng 2015[100]	1	7	0.14	[0.00; 0.58]	0.0%	0.3%
ChenXingHong 2012[102]	398	478	0.83	[0.80; 0.87]	1.8%	0.3%
ChenXingFu 2018[103]	12	43	0.28	[0.15; 0.44]	0.2%	0.3%
CuiXiaoMan 2018[125]	7	17	0.41	[0.18; 0.67]	0.1%	0.3%
DaiBenNa 2020[127]	17	117	0.15	[0.09; 0.22]	0.4%	0.3%
DaiYingXue 2018[129−3]	2	18	0.11	[0.01; 0.35]	0.1%	0.3%
DaiYingXue 2018[129−4]	6	14	0.43	[0.18; 0.71]	0.1%	0.3%
Ying−Hui Deng 2014[135]	21	99	0.21	[0.14; 0.31]	0.4%	0.3%
DuYueHe 2019[148]	1	29	0.03	[0.00; 0.18]	0.1%	0.3%
FangYuLian 2019[155]	241	241	1.00	[0.98; 1.00]	0.9%	0.3%
FangYuLian 2021[156]	809	809	1.00	[1.00; 1.00]	3.0%	0.3%
FengZhi 2018[162]	40	111	0.36	[0.27; 0.46]	0.4%	0.3%
FuXiaoFei 2012[171]	17	20	0.85	[0.62; 0.97]	0.1%	0.3%
GanXiangYang 2014[172]	58	74	0.78	[0.67; 0.87]	0.3%	0.3%
GaoHuiJuan 2015[176]	3	18	0.17	[0.04; 0.41]	0.1%	0.3%
GaoJunYing 2017[177]	5	12	0.42	[0.15; 0.72]	0.0%	0.3%
GaoPeng 2020[181]	17	28	0.61	[0.41; 0.78]	0.1%	0.3%
GaoZhiYong 2017[187−2]	4	10	0.40	[0.12; 0.74]	0.0%	0.3%
GaoZhiYong 2017[187−4]	10	16	0.62	[0.35; 0.85]	0.1%	0.3%
GaoZhiYong 2017[187−6]	16	25	0.64	[0.43; 0.82]	0.1%	0.3%
GongLiQiang 2013[192]	52	139	0.37	[0.29; 0.46]	0.5%	0.3%
GongShuiYing 2017[194]	18	93	0.19	[0.12; 0.29]	0.3%	0.3%
GuYiFu 2020[197]	20	45	0.44	[0.30; 0.60]	0.2%	0.3%
GuoJing 2019[203]	193	193	1.00	[0.98; 1.00]	0.7%	0.3%
GuoMinJian 2017[208−2]	2	6	0.33	[0.04; 0.78]	0.0%	0.3%
GuoMinJian 2017[208−3]	8	11	0.73	[0.39; 0.94]	0.0%	0.3%
GuoMinJian 2017[208−4]	7	10	0.70	[0.35; 0.93]	0.0%	0.3%
GuoMinJian 2017[208−6]	4	7	0.57	[0.18; 0.90]	0.0%	0.3%
GuoMinJian 2017[208−7]	2	7	0.29	[0.04; 0.71]	0.0%	0.3%
GuoMinJian 2017[208−9]	7	16	0.44	[0.20; 0.70]	0.1%	0.3%
GuoMinJian 2017[208−10]	22	48	0.46	[0.31; 0.61]	0.2%	0.3%
HeXuXin 2017[222]	85	156	0.54	[0.46; 0.62]	0.6%	0.3%
HeHanZhen 2014[224]	65	76	0.86	[0.76; 0.93]	0.3%	0.3%
HouYuYuan 2014[231]	63	63	1.00	[0.94; 1.00]	0.2%	0.3%
HuHongAn 2015[233]	105	105	1.00	[0.97; 1.00]	0.4%	0.3%
Ying Hu 2019[239]	21	120	0.17	[0.11; 0.25]	0.4%	0.3%
HuaWeiYu 2018[241]	3	63	0.05	[0.01; 0.13]	0.2%	0.3%
HuangBinBin 2020[245]	26	41	0.63	[0.47; 0.78]	0.2%	0.3%
HuangGuo 2015[252]	19	69	0.28	[0.17; 0.40]	0.3%	0.3%
HuangYanHong 2019[260]	39	84	0.46	[0.35; 0.58]	0.3%	0.3%
HuangYanHong 2019[261]	14	86	0.16	[0.09; 0.26]	0.3%	0.3%
Lei Ji 2021[269]	6	26	0.23	[0.09; 0.44]	0.1%	0.3%
JiLei 2018[272−1]	1	7	0.14	[0.00; 0.58]	0.0%	0.3%
JiLei 2018[272−3]	18	18	1.00	[0.81; 1.00]	0.1%	0.3%
JiRuPing 2020[274]	82	119	0.69	[0.60; 0.77]	0.4%	0.3%

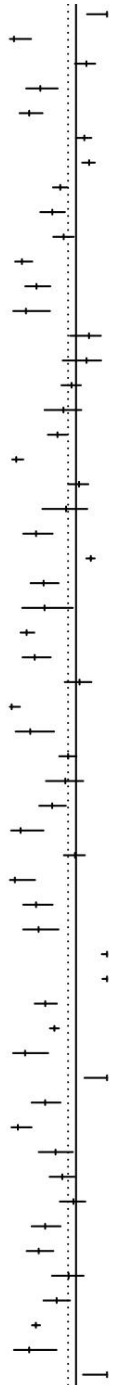

JiangXianChen 2014[283−1]	16	16	1.00	[0.79; 1.00]	0.1%	0.3%
JiangXianChen 2014[283−2]	1	23	0.04	[0.00; 0.22]	0.1%	0.3%
JiangYingCi 2015[284]	52	66	0.79	[0.67; 0.88]	0.2%	0.3%
KangQian 2020[303−1]	11	35	0.31	[0.17; 0.49]	0.1%	0.3%
KangQian 2020[303−2]	10	50	0.20	[0.10; 0.34]	0.2%	0.3%
LiBo 2013[316]	92	120	0.77	[0.68; 0.84]	0.4%	0.3%
LiCaiYun 2012[318]	120	147	0.82	[0.74; 0.88]	0.6%	0.3%
LiShiCong 2020[344]	94	181	0.52	[0.44; 0.59]	0.7%	0.3%
LiShiCong 2018[346]	28	64	0.44	[0.31; 0.57]	0.2%	0.3%
LiShiE 2018[348]	51	92	0.55	[0.45; 0.66]	0.3%	0.3%
LiShouJun 2016[350]	8	64	0.12	[0.06; 0.23]	0.2%	0.3%
LiXiTai 2015[352]	14	51	0.27	[0.16; 0.42]	0.2%	0.3%
LiYueRong 2015[366]	3	18	0.17	[0.04; 0.41]	0.1%	0.3%
LiangRiCheng 2017[376]	22	27	0.81	[0.62; 0.94]	0.1%	0.3%
LiaoChan 2021[380]	15	19	0.79	[0.54; 0.94]	0.1%	0.3%
LinQiFeng 2018[388]	61	96	0.64	[0.53; 0.73]	0.4%	0.3%
LinQingShuang 2016[391]	16	29	0.55	[0.36; 0.74]	0.1%	0.3%
LiuBo 2015[398]	51	104	0.49	[0.39; 0.59]	0.4%	0.3%
LiuDan 2019[403]	6	90	0.07	[0.02; 0.14]	0.3%	0.3%
LiuDongSheng 2019[404]	57	80	0.71	[0.60; 0.81]	0.3%	0.3%
LiuHongLian 2018[411]	11	19	0.58	[0.33; 0.80]	0.1%	0.3%
LiuQingLian 2019[420]	10	37	0.27	[0.14; 0.44]	0.1%	0.3%
LiuShiKe 2012[421]	255	306	0.83	[0.79; 0.87]	1.1%	0.3%
LiuShiKe 2016[422]	16	46	0.35	[0.21; 0.50]	0.2%	0.3%
LiuYing 2019[437]	5	14	0.36	[0.13; 0.65]	0.1%	0.3%
LiuYuan 2016[438−2]	21	121	0.17	[0.11; 0.25]	0.5%	0.3%
LuoLe 2017[472]	10	39	0.26	[0.13; 0.42]	0.1%	0.3%
MaTao 2018[487]	33	46	0.72	[0.57; 0.84]	0.2%	0.3%
MaoJianYing 2016[493]	1	51	0.02	[0.00; 0.10]	0.2%	0.3%
MoYuJie 2018[505]	4	19	0.21	[0.06; 0.46]	0.1%	0.3%
NiChaoRong 2019[510]	75	125	0.60	[0.51; 0.69]	0.5%	0.3%
NiChaoRong 2019[511]	16	28	0.57	[0.37; 0.76]	0.1%	0.3%
NiChunYan 2020[512]	24	55	0.44	[0.30; 0.58]	0.2%	0.3%
OuSheXiang 2019[515]	2	18	0.11	[0.01; 0.35]	0.1%	0.3%
PangZhiFeng 2017[524]	53	79	0.67	[0.56; 0.77]	0.3%	0.3%
PangZhiMing 2015[526]	1	19	0.05	[0.00; 0.26]	0.1%	0.3%
PengXiaoXue 2015[528]	10	37	0.27	[0.14; 0.44]	0.1%	0.3%
QiYanQiu 2018[532]	8	27	0.30	[0.14; 0.50]	0.1%	0.3%
QiaoYingQin 2016[543]	67	67	1.00	[0.95; 1.00]	0.3%	0.3%
QiuHaiYan 2013[551]	76	76	1.00	[0.95; 1.00]	0.3%	0.3%
RenFuLin 2013[555]	27	74	0.36	[0.26; 0.48]	0.3%	0.3%
ShiChao 2013[579]	212	462	0.46	[0.41; 0.51]	1.7%	0.3%
ShiYongLin 2015[587]	3	19	0.16	[0.03; 0.40]	0.1%	0.3%
ShuaiHuiQun 2012[589]	14	14	1.00	[0.77; 1.00]	0.1%	0.3%
SongCanLei 2020[590]	16	44	0.36	[0.22; 0.52]	0.2%	0.3%
SongCanLei 2017[593]	3	36	0.08	[0.02; 0.22]	0.1%	0.3%
SongHuiRong 2017[597]	16	34	0.47	[0.30; 0.65]	0.1%	0.3%
SongYuFang 2019[600]	34	63	0.54	[0.41; 0.67]	0.2%	0.3%
SuTong 2020[604]	36	55	0.65	[0.51; 0.78]	0.2%	0.3%
TangYuHuan 2017[630]	16	44	0.36	[0.22; 0.52]	0.2%	0.3%
TaoLiYan 2020[636]	14	47	0.30	[0.17; 0.45]	0.2%	0.3%
TianYaLin 2021[642]	23	38	0.61	[0.43; 0.76]	0.1%	0.3%
WangJinSheng 2019[645]	27	56	0.48	[0.35; 0.62]	0.2%	0.3%
WangJie 2015[659]	121	451	0.27	[0.23; 0.31]	1.7%	0.3%
WangTieJun 2020[678]	3	15	0.20	[0.04; 0.48]	0.1%	0.3%
WangWeiRu 2013[680]	13	13	1.00	[0.75; 1.00]	0.1%	0.3%

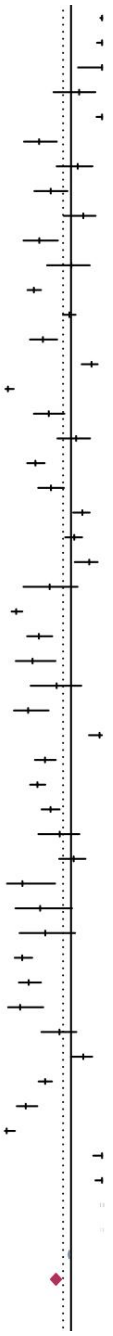

WangWenYing 2011[681]	204	204	1.00	[0.98; 1.00]	0.8%	0.3%
WangXiaoDong 2017[688]	69	69	1.00	[0.95; 1.00]	0.3%	0.3%
WangXiaoYi 2017[691]	13	13	1.00	[0.75; 1.00]	0.1%	0.3%
WuGuoFu 2018[717]	13	17	0.76	[0.50; 0.93]	0.1%	0.3%
WuHongXing 2016[718]	63	63	1.00	[0.94; 1.00]	0.2%	0.3%
WuJingWen 2020[719]	12	34	0.35	[0.20; 0.54]	0.1%	0.3%
WuQinDi 2018[725]	18	24	0.75	[0.53; 0.90]	0.1%	0.3%
WuWenQian 2018[729]	17	36	0.47	[0.30; 0.65]	0.1%	0.3%
WuYang 2018[734]	21	26	0.81	[0.61; 0.93]	0.1%	0.3%
WuYiLing 2017[736]	11	31	0.35	[0.19; 0.55]	0.1%	0.3%
WuZhenYu 2012[738]	13	19	0.68	[0.43; 0.87]	0.1%	0.3%
XiaYingPin 2018[743]	53	177	0.30	[0.23; 0.37]	0.7%	0.3%
XiaZhongFa 2012[744]	139	209	0.67	[0.60; 0.73]	0.8%	0.3%
XiaoSongJian 2017[745]	20	51	0.39	[0.26; 0.54]	0.2%	0.3%
XieBin 2020[758]	58	65	0.89	[0.79; 0.96]	0.2%	0.3%
XuJianRong 2018[767]	3	92	0.03	[0.01; 0.09]	0.3%	0.3%
YangCaiBin 2018[796]	19	42	0.45	[0.30; 0.61]	0.2%	0.3%
YangJiXing 2019[801]	22	30	0.73	[0.54; 0.88]	0.1%	0.3%
YangJing 2018[804]	35	111	0.32	[0.23; 0.41]	0.4%	0.3%
YaoJianXiang 2016[822]	28	59	0.47	[0.34; 0.61]	0.2%	0.3%
YeXianMing 2017[832]	75	94	0.80	[0.70; 0.87]	0.4%	0.3%
Yan Yu 2017[852]	80	112	0.71	[0.62; 0.80]	0.4%	0.3%
ZhanYueWang 2019[860]	33	38	0.87	[0.72; 0.96]	0.1%	0.3%
ZhangChong 2015[862]	6	13	0.46	[0.19; 0.75]	0.1%	0.3%
ZhangHaiYan 2019[867]	17	145	0.12	[0.07; 0.18]	0.5%	0.3%
ZhangQin 2020[885]	20	57	0.35	[0.23; 0.49]	0.2%	0.3%
ZhangXiaoYi 2015[891]	6	21	0.29	[0.11; 0.52]	0.1%	0.3%
ZhangYanMing 2019[898]	8	15	0.53	[0.27; 0.79]	0.1%	0.3%
ZhangZhengDong 2017[904]	6	25	0.24	[0.09; 0.45]	0.1%	0.3%
ZhaoMeiLing 2019[918−2]	38	39	0.97	[0.87; 1.00]	0.1%	0.3%
ZhaoMengJiao 2018[919]	36	87	0.41	[0.31; 0.52]	0.3%	0.3%
ZhaoXueCheng 2016[929]	50	149	0.34	[0.26; 0.42]	0.6%	0.3%
ZhaoYuLi 2019[933]	56	119	0.47	[0.38; 0.56]	0.4%	0.3%
ZhenGuoXin 2020[936]	13	23	0.57	[0.34; 0.77]	0.1%	0.3%
HuangJiaCheng 2021[944]	34	48	0.71	[0.56; 0.83]	0.2%	0.3%
ZhongJianMing 2014[946]	2	11	0.18	[0.02; 0.52]	0.0%	0.3%
Jun−Hua Zhou 2018[955]	4	11	0.36	[0.11; 0.69]	0.0%	0.3%
ZhouYin 2016[964]	5	12	0.42	[0.15; 0.72]	0.0%	0.3%
ZhuChengMing 2017[967]	14	78	0.18	[0.10; 0.28]	0.3%	0.3%
Guo XH 2018[1000]	15	61	0.25	[0.14; 0.37]	0.2%	0.3%
Li J 2018[1015]	3	19	0.16	[0.03; 0.40]	0.1%	0.3%
Luo LF 2015[1030]	18	32	0.56	[0.38; 0.74]	0.1%	0.3%
Shi C 2016[1046]	46	57	0.81	[0.68; 0.90]	0.2%	0.3%
Xu H 2013[1059−1]	86	207	0.42	[0.35; 0.49]	0.8%	0.3%
Xu H 2013[1059−2]	14	65	0.22	[0.12; 0.33]	0.2%	0.3%
XiangLunHui 2016[1101]	1	51	0.02	[0.00; 0.10]	0.2%	0.3%
ZouYong 2011[1103]	39	39	1.00	[0.91; 1.00]	0.1%	0.3%
ChenCen 2016[1105]	52	52	1.00	[0.93; 1.00]	0.2%	0.3%
HeFei 2020[1108]	610	610	1.00	[0.99; 1.00]	2.3%	0.3%
LiuYun 2019[1116]	353	353	1.00	[0.99; 1.00]	1.3%	0.3%
**Common effect model**		**12638**	**0.66**	**[0.65; 0.67]**	**47.5%**	**--**
**Random effects model**			**0.53**	**[0.47; 0.58]**	**--**	**53.0%**

Heterogeneity: $I^2 = 98\%$, $\tau^2 = 0.1204$, $p = 0$

age = 18−60y						
CaiWenFeng 2013[25]	122	141	0.87	[0.80; 0.92]	0.5%	0.3%
CaoShen 2019[32]	57	62	0.92	[0.82; 0.97]	0.2%	0.3%
ShenYuGang 2016[55]	183	233	0.79	[0.73; 0.84]	0.9%	0.3%
ChenAQun 2016[59]	61	64	0.95	[0.87; 0.99]	0.2%	0.3%
ChenGuoCui 2011[69]	19	27	0.70	[0.50; 0.86]	0.1%	0.3%
ChenHeJuan 2018[73]	13	14	0.93	[0.66; 1.00]	0.1%	0.3%
ChenJianMei 2017[79]	95	110	0.86	[0.79; 0.92]	0.4%	0.3%
ChenYiYi 2015[109]	239	282	0.85	[0.80; 0.89]	1.1%	0.3%
DuYao 2013[150]	157	157	1.00	[0.98; 1.00]	0.6%	0.3%
FengZhi 2020[161]	15	26	0.58	[0.37; 0.77]	0.1%	0.3%
GaoRiHong 2019[182]	19	30	0.63	[0.44; 0.80]	0.1%	0.3%
GaoShuPing 2019[183]	31	51	0.61	[0.46; 0.74]	0.2%	0.3%
GaoZhiYong 2017[187−3]	8	15	0.53	[0.27; 0.79]	0.1%	0.3%
GaoZhiYong 2017[187−8]	15	22	0.68	[0.45; 0.86]	0.1%	0.3%
GuKaiChen 2020[195]	16	21	0.76	[0.53; 0.92]	0.1%	0.3%
GuoLi 2019[205]	54	75	0.72	[0.60; 0.82]	0.3%	0.3%
HaoYongJian 2020[216]	17	25	0.68	[0.46; 0.85]	0.1%	0.3%
HuangGuo 2015[253]	36	87	0.41	[0.31; 0.52]	0.3%	0.3%
HuangLiQing 2020[254]	184	184	1.00	[0.98; 1.00]	0.7%	0.3%
HuangSiYue 2020[257]	88	90	0.98	[0.92; 1.00]	0.3%	0.3%
JiJinHua 2018[265]	32	41	0.78	[0.62; 0.89]	0.2%	0.3%
JiLei 2020[270]	17	19	0.89	[0.67; 0.99]	0.1%	0.3%
JiangChen 2019[285]	24	26	0.92	[0.75; 0.99]	0.1%	0.3%
JiangLie 2014[291]	3	15	0.20	[0.04; 0.48]	0.1%	0.3%
KuangHaoCheng 2016[307]	511	667	0.77	[0.73; 0.80]	2.5%	0.3%
LaiShiMing 2014[308]	98	105	0.93	[0.87; 0.97]	0.4%	0.3%
LiDaiBo 2018[320]	13	18	0.72	[0.47; 0.90]	0.1%	0.3%
LiJie 2019[329]	95	106	0.90	[0.82; 0.95]	0.4%	0.3%
LiQun 2010[340]	214	258	0.83	[0.78; 0.87]	1.0%	0.3%
LiShiE 2018[349]	49	77	0.64	[0.52; 0.74]	0.3%	0.3%
LiXiuFang 2018[359]	19	20	0.95	[0.75; 1.00]	0.1%	0.3%
LiYiLan 2014[365]	177	228	0.78	[0.72; 0.83]	0.9%	0.3%
LiuKaiQian 2011[415]	528	634	0.83	[0.80; 0.86]	2.4%	0.3%
LiuXiaoXiao 2014[430]	16	18	0.89	[0.65; 0.99]	0.1%	0.3%
LiuYi 2013[436]	8	9	0.89	[0.52; 1.00]	0.0%	0.3%
LuZhengXiang 2016[451]	47	83	0.57	[0.45; 0.67]	0.3%	0.3%
LuJianYong 2015[456]	60	60	1.00	[0.94; 1.00]	0.2%	0.3%
LuWeiWei 2016[462]	333	406	0.82	[0.78; 0.86]	1.5%	0.3%
MaMengMeng 2018[484]	175	223	0.78	[0.72; 0.84]	0.8%	0.3%
PanYiFeng 2017[521]	44	82	0.54	[0.42; 0.65]	0.3%	0.3%
QiXiaoQi 2019[534]	135	185	0.73	[0.66; 0.79]	0.7%	0.3%
QiYing 2018[535−1]	99	173	0.57	[0.49; 0.65]	0.6%	0.3%
QiYing 2018[535−2]	34	71	0.48	[0.36; 0.60]	0.3%	0.3%
QianLiZhen 2020[537]	16	23	0.70	[0.47; 0.87]	0.1%	0.3%
RenYuHua 2016[562]	73	110	0.66	[0.57; 0.75]	0.4%	0.3%
Hao−Yu Shi 2019[582]	9	9	1.00	[0.66; 1.00]	0.0%	0.3%
SunJing 2015[611]	68	92	0.74	[0.64; 0.83]	0.3%	0.3%
SunZhou 2016[625]	192	230	0.83	[0.78; 0.88]	0.9%	0.3%
TanDongMei 2012[627]	87	87	1.00	[0.96; 1.00]	0.3%	0.3%
WangBing 2017[649]	38	58	0.66	[0.52; 0.78]	0.2%	0.3%
WangDaHu 2020[652]	141	182	0.77	[0.71; 0.83]	0.7%	0.3%
WangHu 2018[656]	66	72	0.92	[0.83; 0.97]	0.3%	0.3%
WangMan 2017[668]	68	85	0.80	[0.70; 0.88]	0.3%	0.3%
WangMingLiang 2020[671]	65	107	0.61	[0.51; 0.70]	0.4%	0.3%
WeiYiYun 2015[705]	20	22	0.91	[0.71; 0.99]	0.1%	0.3%
WuPei 2011[724]	146	146	1.00	[0.98; 1.00]	0.5%	0.3%

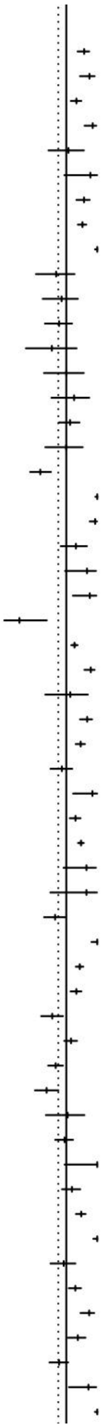

WuShuiXin 2012[727]	25	25	1.00 [0.86; 1.00]	0.1%	0.3%
WuZhaoChun 2015[737]	17	17	1.00 [0.80; 1.00]	0.1%	0.3%
WuZhiSheng 2014[741]	176	176	1.00 [0.98; 1.00]	0.7%	0.3%
XiaGuangHui 2019[742]	82	106	0.77 [0.68; 0.85]	0.4%	0.3%
Xiao−Bo Xie 2020[754]	85	113	0.75 [0.66; 0.83]	0.4%	0.3%
XieYuanQi 2016[756]	34	34	1.00 [0.90; 1.00]	0.1%	0.3%
XuanLingFeng 2014[784]	17	17	1.00 [0.80; 1.00]	0.1%	0.3%
YangAiQing 2018[795]	30	40	0.75 [0.59; 0.87]	0.2%	0.3%
YangZhiWen 2013[820]	11	11	1.00 [0.72; 1.00]	0.0%	0.3%
ZhangDongSheng 2013[864]	97	97	1.00 [0.96; 1.00]	0.4%	0.3%
ZhangHaiYan 2016[868]	66	78	0.85 [0.75; 0.92]	0.3%	0.3%
ZhangHengQiu 2014[869]	7	18	0.39 [0.17; 0.64]	0.1%	0.3%
ZhangHuiLing 2018[871]	33	40	0.82 [0.67; 0.93]	0.2%	0.3%
ZhangPan 2020[883]	15	16	0.94 [0.70; 1.00]	0.1%	0.3%
ZhangZhen 2015[903]	47	76	0.62 [0.50; 0.73]	0.3%	0.3%
ZhangZhiZhong 2020[906]	129	263	0.49 [0.43; 0.55]	1.0%	0.3%
ZhouGuoYing 2016[952]	12	12	1.00 [0.74; 1.00]	0.0%	0.3%
ZhuWeiWei 2018[976]	89	91	0.98 [0.92; 1.00]	0.3%	0.3%
Chen D 2019[987]	146	209	0.70 [0.63; 0.76]	0.8%	0.3%
Guo Z 2014[1001]	52	87	0.60 [0.49; 0.70]	0.3%	0.3%
Huang XY 2017[1007]	401	753	0.53 [0.50; 0.57]	2.8%	0.3%
Li JS 2016[1017−2]	23	121	0.19 [0.12; 0.27]	0.5%	0.3%
Y Li 2013[1019]	308	396	0.78 [0.73; 0.82]	1.5%	0.3%
Liu Y 2015[1025]	99	99	1.00 [0.96; 1.00]	0.4%	0.3%
Lu Y 2020[1029]	60	157	0.38 [0.31; 0.46]	0.6%	0.3%
Qin M 2016[1037]	25	29	0.86 [0.68; 0.96]	0.1%	0.3%
Ruan F 2013[1039]	41	63	0.65 [0.52; 0.77]	0.2%	0.3%
Zhang L 2018[1071]	537	631	0.85 [0.82; 0.88]	2.4%	0.3%
Zhang XF 2020[1076]	12	27	0.44 [0.25; 0.65]	0.1%	0.3%
Zhou N 2016[1079]	229	1614	0.14 [0.13; 0.16]	6.0%	0.3%
Zhou X 2019[1080]	54	75	0.72 [0.60; 0.82]	0.3%	0.3%
**Common effect model**		**11954**	**0.71 [0.70; 0.72]**	**44.8%**	**--**
**Random effects model**			**0.80 [0.75; 0.85]**	**--**	**29.1%**
Heterogeneity: $I^2 = 98\%$, $\tau^2 = 0.0634$, $p = 0$					
**age≥60y**					
GaoZhiYong 2017[187−5]	2	7	0.29 [0.04; 0.71]	0.0%	0.3%
GaoZhiYong 2017[187−7]	8	21	0.38 [0.18; 0.62]	0.1%	0.3%
GuoMinJian 2017[208−8]	3	7	0.43 [0.10; 0.82]	0.0%	0.3%
QianZiYu 2012[539]	17	22	0.77 [0.55; 0.92]	0.1%	0.3%
SongCanLei 2012[591]	16	18	0.89 [0.65; 0.99]	0.1%	0.3%
SongCanLei 2013[594]	15	26	0.58 [0.37; 0.77]	0.1%	0.3%
SunLiYan 2020[613]	7	11	0.64 [0.31; 0.89]	0.0%	0.3%
WangXiaoYi 2017[690]	13	13	1.00 [0.75; 1.00]	0.1%	0.3%
XuFeng 2012[774]	21	22	0.95 [0.77; 1.00]	0.1%	0.3%
ZhangDianXiang 2012[863]	5	5	1.00 [0.48; 1.00]	0.0%	0.3%
**Common effect model**		**152**	**0.74 [0.67; 0.82]**	**0.6%**	**--**
**Random effects model**			**0.74 [0.55; 0.90]**	**--**	**3.0%**
Heterogeneity: $I^2 = 80\%$, $\tau^2 = 0.0681$, $p < 0.01$					
**Common effect model**		**26627**	**0.68 [0.68; 0.69]**	**100.0%**	**--**
**Random effects model**			**0.60 [0.56; 0.64]**	**--**	**100.0%**

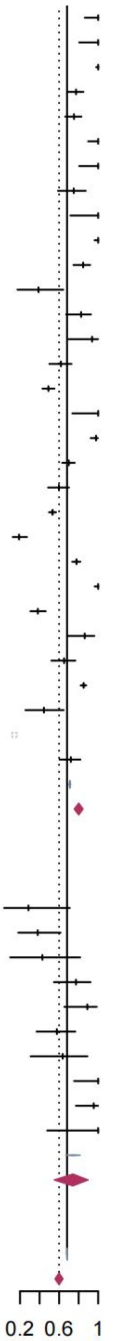

Heterogeneity: $I^2 = 98\%$, $\tau^2 = 0.1248$, $p = 0$
Test for subgroup differences (common effect): $\chi^2_3 = 112.77$, df = 3 ($p < 0.01$)
Test for subgroup differences (random effects): $\chi^2_3 = 76.22$, df = 3 ($p < 0.01$)

(e3)

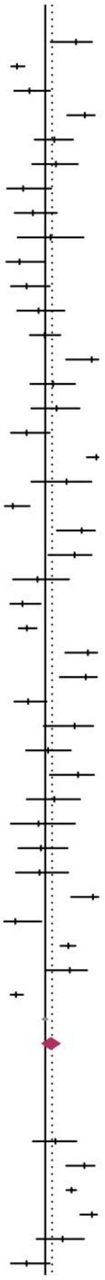

Study	Abdominal pain	Events	Total	Proportion	95%−CI	Weight (common)	Weight (random)
**age = 0−4y**							
Cai SX 2017[21]		13	17	0.76	[0.50; 0.93]	0.1%	0.4%
CaoXiaoPing 2018[33]		17	113	0.15	[0.09; 0.23]	0.5%	0.4%
ZengFengMei 2020[41]		7	25	0.28	[0.12; 0.49]	0.1%	0.4%
ZengLei 2018[44]		24	28	0.86	[0.67; 0.96]	0.1%	0.4%
ShenYiPing 2018[52]		14	26	0.54	[0.33; 0.73]	0.1%	0.4%
ChenJingFang 2018[84−1]		10	18	0.56	[0.31; 0.78]	0.1%	0.4%
ChenJingFang 2018[84−2]		3	14	0.21	[0.05; 0.51]	0.1%	0.4%
GaoHaiMing 2014[174]		6	19	0.32	[0.13; 0.57]	0.1%	0.4%
GuoMinJian 2017[208−1]		4	8	0.50	[0.16; 0.84]	0.0%	0.3%
GuoMinJian 2017[208−5]		3	17	0.18	[0.04; 0.43]	0.1%	0.4%
HuGuangYi 2017[232]		5	20	0.25	[0.09; 0.49]	0.1%	0.4%
JiLei 2018[272−2]		6	16	0.38	[0.15; 0.65]	0.1%	0.4%
LiaoKeChang 2018[381]		18	41	0.44	[0.28; 0.60]	0.2%	0.4%
LuHua 2016[454]		13	14	0.93	[0.66; 1.00]	0.1%	0.4%
LuCaiFang 2020[458]		10	19	0.53	[0.29; 0.76]	0.1%	0.4%
MengXiangJie 2012[499]		9	16	0.56	[0.30; 0.80]	0.1%	0.4%
PangZhiFeng 2014[525]		5	20	0.25	[0.09; 0.49]	0.1%	0.4%
RenFuLin 2013[554]		42	43	0.98	[0.88; 1.00]	0.2%	0.4%
RenLiJun 2020[556]		6	9	0.67	[0.30; 0.93]	0.0%	0.3%
WangHua 2014[657]		3	28	0.11	[0.02; 0.28]	0.1%	0.4%
WangJun 2012[663]		14	17	0.82	[0.57; 0.96]	0.1%	0.4%
WangKaiLiang 2019[665]		12	16	0.75	[0.48; 0.93]	0.1%	0.4%
WangXiaoQin 2015[689]		4	11	0.36	[0.11; 0.69]	0.1%	0.3%
WeiGuiYing 2019[706]		6	29	0.21	[0.08; 0.40]	0.1%	0.4%
WuYang 2018[735]		23	91	0.25	[0.17; 0.35]	0.4%	0.4%
YangJiChao 2016[802]		16	18	0.89	[0.65; 0.99]	0.1%	0.4%
YangRongXing 2019[808]		13	15	0.87	[0.60; 0.98]	0.1%	0.4%
YangSenPing 2018[810]		8	30	0.27	[0.12; 0.46]	0.1%	0.4%
YueYong 2017[859]		9	12	0.75	[0.43; 0.95]	0.1%	0.3%
ZhangAiHua 2018[861]		9	19	0.47	[0.24; 0.71]	0.1%	0.4%
ZhangGuangMing 2018[907]		11	14	0.79	[0.49; 0.95]	0.1%	0.4%
ZhangGuoBao 2020[908]		7	13	0.54	[0.25; 0.81]	0.1%	0.3%
ZhaoMeiLing 2019[918−1]		3	8	0.38	[0.09; 0.76]	0.0%	0.3%
ZhouJianHong 2013[954]		6	15	0.40	[0.16; 0.68]	0.1%	0.4%
ZhouLiHong 2016[956]		5	13	0.38	[0.14; 0.68]	0.1%	0.3%
ZhuChunXiu 2019[968]		16	17	0.94	[0.71; 1.00]	0.1%	0.4%
S Kim 2019[1012]		2	15	0.13	[0.02; 0.40]	0.1%	0.4%
Y Li 2021[1018]		98	143	0.69	[0.60; 0.76]	0.6%	0.4%
Zhang TL 2017[1075]		14	20	0.70	[0.46; 0.88]	0.1%	0.4%
SaiLinTao 2014[1120]		17	122	0.14	[0.08; 0.21]	0.6%	0.4%
**Common effect model**			**1149**	**0.44**	**[0.41; 0.47]**	**5.3%**	**--**
**Random effects model**				**0.50**	**[0.41; 0.60]**	**--**	**14.9%**
Heterogeneity: $I^2 = 91\%$, $\tau^2 = 0.0703$, $p < 0.01$							
**age = 5−17y**							
BaiAiLi 2020[3]		11	20	0.55	[0.32; 0.77]	0.1%	0.4%
CaiWenFeng 2014[26]		23	27	0.85	[0.66; 0.96]	0.1%	0.4%
ShenJiChuan 2011[50]		224	312	0.72	[0.66; 0.77]	1.4%	0.5%
ShenYuGang 2016[54]		40	43	0.93	[0.81; 0.99]	0.2%	0.4%
ChenCaiRong 2020[62]		10	16	0.62	[0.35; 0.85]	0.1%	0.4%
ChenJian 2016[78]		5	20	0.25	[0.09; 0.49]	0.1%	0.4%

MaTao 2018[487]	23	46	0.50	[0.35; 0.65]	0.2%	0.4%
MaoJianYing 2016[493]	12	51	0.24	[0.13; 0.37]	0.2%	0.4%
MoYuJie 2018[505]	11	19	0.58	[0.33; 0.80]	0.1%	0.4%
NiChaoRong 2019[510]	84	125	0.67	[0.58; 0.75]	0.6%	0.4%
NiChaoRong 2019[511]	17	28	0.61	[0.41; 0.78]	0.1%	0.4%
NiChunYan 2020[512]	25	55	0.45	[0.32; 0.59]	0.2%	0.4%
OuSheXiang 2019[515]	7	18	0.39	[0.17; 0.64]	0.1%	0.4%
PangZhiFeng 2017[524]	44	79	0.56	[0.44; 0.67]	0.4%	0.4%
PangZhiMing 2015[526]	11	19	0.58	[0.33; 0.80]	0.1%	0.4%
PengXiaoXue 2015[528]	28	37	0.76	[0.59; 0.88]	0.2%	0.4%
QiYanQiu 2018[532]	5	27	0.19	[0.06; 0.38]	0.1%	0.4%
QiuHaiYan 2013[551]	69	76	0.91	[0.82; 0.96]	0.3%	0.4%
RenFuLin 2013[555]	24	74	0.32	[0.22; 0.44]	0.3%	0.4%
ShiChao 2013[579]	255	462	0.55	[0.51; 0.60]	2.1%	0.5%
ShiYongLin 2015[587]	10	19	0.53	[0.29; 0.76]	0.1%	0.4%
ShuaiHuiQun 2012[589]	6	14	0.43	[0.18; 0.71]	0.1%	0.4%
SongCanLei 2020[590]	6	44	0.14	[0.05; 0.27]	0.2%	0.4%
SongCanLei 2017[593]	13	36	0.36	[0.21; 0.54]	0.2%	0.4%
SongHuiRong 2017[597]	19	34	0.56	[0.38; 0.73]	0.2%	0.4%
SongYuFang 2019[600]	47	63	0.75	[0.62; 0.85]	0.3%	0.4%
SuTong 2020[604]	17	55	0.31	[0.19; 0.45]	0.2%	0.4%
SunQin 2019[616]	32	32	1.00	[0.89; 1.00]	0.1%	0.4%
TangYuHuan 2017[630]	31	44	0.70	[0.55; 0.83]	0.2%	0.4%
TianYaLin 2021[642]	17	38	0.45	[0.29; 0.62]	0.2%	0.4%
WangJinSheng 2019[645]	22	56	0.39	[0.26; 0.53]	0.3%	0.4%
WangJie 2015[659]	225	451	0.50	[0.45; 0.55]	2.0%	0.5%
WangWeiRu 2013[680]	5	13	0.38	[0.14; 0.68]	0.1%	0.3%
WangWenYing 2011[681]	1	204	0.00	[0.00; 0.03]	0.9%	0.4%
WangXiaoYi 2017[691]	7	13	0.54	[0.25; 0.81]	0.1%	0.3%
WeiXia 2019[708]	1	22	0.05	[0.00; 0.23]	0.1%	0.4%
WuGuoFu 2018[717]	5	17	0.29	[0.10; 0.56]	0.1%	0.4%
WuHongXing 2016[718]	41	63	0.65	[0.52; 0.77]	0.3%	0.4%
WuQinDi 2018[725]	14	24	0.58	[0.37; 0.78]	0.1%	0.4%
WuWenQian 2018[729]	18	36	0.50	[0.33; 0.67]	0.2%	0.4%
WuXiaoMin 2021[732]	130	159	0.82	[0.75; 0.87]	0.7%	0.4%
WuYang 2018[734]	21	26	0.81	[0.61; 0.93]	0.1%	0.4%
WuZhenYu 2012[738]	17	19	0.89	[0.67; 0.99]	0.1%	0.4%
XiaYingPin 2018[743]	32	177	0.18	[0.13; 0.25]	0.8%	0.4%
XiaZhongFa 2012[744]	139	209	0.67	[0.60; 0.73]	0.9%	0.4%
XiaoSongJian 2017[745]	38	51	0.75	[0.60; 0.86]	0.2%	0.4%
XieBin 2020[758]	46	65	0.71	[0.58; 0.81]	0.3%	0.4%
XuJianRong 2018[767]	51	92	0.55	[0.45; 0.66]	0.4%	0.4%
YangJiXing 2019[801]	26	30	0.87	[0.69; 0.96]	0.1%	0.4%
YangJing 2018[804]	85	111	0.77	[0.68; 0.84]	0.5%	0.4%
YaoJianXiang 2016[822]	29	59	0.49	[0.36; 0.63]	0.3%	0.4%
YuHong 2016[848]	32	80	0.40	[0.29; 0.52]	0.4%	0.4%
ZhanYueWang 2019[860]	25	38	0.66	[0.49; 0.80]	0.2%	0.4%
ZhangHaiYan 2019[867]	47	145	0.32	[0.25; 0.41]	0.7%	0.4%
ZhangQin 2020[885]	34	57	0.60	[0.46; 0.72]	0.3%	0.4%
ZhangXiaoYi 2015[891]	12	21	0.57	[0.34; 0.78]	0.1%	0.4%
ZhangYanMing 2019[898]	9	15	0.60	[0.32; 0.84]	0.1%	0.4%
ZhangZhengDong 2017[904]	16	25	0.64	[0.43; 0.82]	0.1%	0.4%
ZhaoMeiLing 2019[918−2]	16	39	0.41	[0.26; 0.58]	0.2%	0.4%
ZhaoXueCheng 2016[929]	50	149	0.34	[0.26; 0.42]	0.7%	0.4%
ZhaoYuLi 2019[933]	92	119	0.77	[0.69; 0.84]	0.5%	0.4%

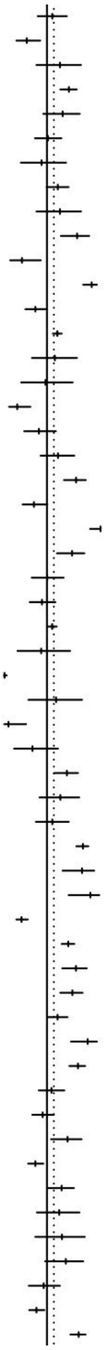

ChenJian 2017[80]	56	69	0.81	[0.70; 0.90]	0.3%	0.4%
ChenXiaoFeng 2015[100]	7	7	1.00	[0.59; 1.00]	0.0%	0.3%
ChenXingHong 2012[102]	435	478	0.91	[0.88; 0.93]	2.2%	0.5%
ChenXingFu 2018[103]	28	43	0.65	[0.49; 0.79]	0.2%	0.4%
ChenYiXiong 2018[108]	12	21	0.57	[0.34; 0.78]	0.1%	0.4%
CuiXiaoMan 2018[125]	6	17	0.35	[0.14; 0.62]	0.1%	0.4%
DaiBenNa 2020[127]	22	117	0.19	[0.12; 0.27]	0.5%	0.4%
Ying-Hui Deng 2014[135]	42	99	0.42	[0.33; 0.53]	0.4%	0.4%
FangYuLian 2019[155]	22	241	0.09	[0.06; 0.13]	1.1%	0.4%
FangYuLian 2021[156]	38	809	0.05	[0.03; 0.06]	3.6%	0.5%
FuXiaoFei 2012[171]	19	20	0.95	[0.75; 1.00]	0.1%	0.4%
GanXiangYang 2014[172]	54	74	0.73	[0.61; 0.83]	0.3%	0.4%
GaoHuiJuan 2015[176]	3	18	0.17	[0.04; 0.41]	0.1%	0.4%
GaoJunYing 2017[177]	8	12	0.67	[0.35; 0.90]	0.1%	0.3%
GongLiQiang 2013[192]	37	139	0.27	[0.19; 0.35]	0.6%	0.4%
GuYiFu 2020[197]	25	45	0.56	[0.40; 0.70]	0.2%	0.4%
GuoJing 2019[203]	55	193	0.28	[0.22; 0.35]	0.9%	0.4%
GuoMinJian 2017[208-9]	8	16	0.50	[0.25; 0.75]	0.1%	0.4%
GuoMinJian 2017[208-10]	18	48	0.38	[0.24; 0.53]	0.2%	0.4%
HeXuXin 2017[222]	119	156	0.76	[0.69; 0.83]	0.7%	0.4%
HeHanZhen 2014[224]	52	76	0.68	[0.57; 0.79]	0.3%	0.4%
HouYuYuan 2014[231]	42	63	0.67	[0.54; 0.78]	0.3%	0.4%
HuHongAn 2015[233]	77	105	0.73	[0.64; 0.81]	0.5%	0.4%
Ying Hu 2019[239]	6	120	0.05	[0.02; 0.11]	0.5%	0.4%
HuaWeiYu 2018[241]	13	63	0.21	[0.11; 0.33]	0.3%	0.4%
HuangBinBin 2020[245]	16	41	0.39	[0.24; 0.55]	0.2%	0.4%
HuangGuo 2015[252]	29	69	0.42	[0.30; 0.55]	0.3%	0.4%
HuangYanHong 2019[260]	15	84	0.18	[0.10; 0.28]	0.4%	0.4%
HuangYanHong 2019[261]	11	86	0.13	[0.07; 0.22]	0.4%	0.4%
JiLei 2018[272-1]	1	7	0.14	[0.00; 0.58]	0.0%	0.3%
JiLei 2018[272-3]	3	18	0.17	[0.04; 0.41]	0.1%	0.4%
JiRuPing 2020[274]	45	119	0.38	[0.29; 0.47]	0.5%	0.4%
JiangXianChen 2014[283-1]	9	16	0.56	[0.30; 0.80]	0.1%	0.4%
JiangXianChen 2014[283-2]	17	23	0.74	[0.52; 0.90]	0.1%	0.4%
JiangYiMei 2017[294]	4	15	0.27	[0.08; 0.55]	0.1%	0.4%
KangQian 2020[303-1]	9	35	0.26	[0.12; 0.43]	0.2%	0.4%
KangQian 2020[303-2]	32	50	0.64	[0.49; 0.77]	0.2%	0.4%
LiBo 2013[316]	60	120	0.50	[0.41; 0.59]	0.5%	0.4%
LiCaiYun 2012[318]	56	147	0.38	[0.30; 0.46]	0.7%	0.4%
LiShiCong 2020[344]	134	181	0.74	[0.67; 0.80]	0.8%	0.4%
LiShiCong 2018[346]	42	64	0.66	[0.53; 0.77]	0.3%	0.4%
LiShiE 2018[348]	20	92	0.22	[0.14; 0.32]	0.4%	0.4%
LiShouJun 2016[350]	40	64	0.62	[0.50; 0.74]	0.3%	0.4%
LiangRiCheng 2017[376]	17	27	0.63	[0.42; 0.81]	0.1%	0.4%
LiaoChan 2021[380]	9	19	0.47	[0.24; 0.71]	0.1%	0.4%
LinQiFeng 2018[388]	49	96	0.51	[0.41; 0.61]	0.4%	0.4%
LinQingShuang 2016[391]	25	29	0.86	[0.68; 0.96]	0.1%	0.4%
LiuBaiWei 2017[395]	8	24	0.33	[0.16; 0.55]	0.1%	0.4%
LiuBo 2015[398]	54	104	0.52	[0.42; 0.62]	0.5%	0.4%
LiuDan 2019[403]	30	90	0.33	[0.24; 0.44]	0.4%	0.4%
LiuDongSheng 2019[404]	58	80	0.72	[0.61; 0.82]	0.4%	0.4%
LiuHongLian 2018[411]	8	19	0.42	[0.20; 0.67]	0.1%	0.4%
LiuShiKe 2012[421]	193	306	0.63	[0.57; 0.68]	1.4%	0.5%
LiuShiKe 2016[422]	22	46	0.48	[0.33; 0.63]	0.2%	0.4%
LiuYing 2019[437]	11	14	0.79	[0.49; 0.95]	0.1%	0.4%
LiuYuan 2016[438-2]	20	121	0.17	[0.10; 0.24]	0.5%	0.4%

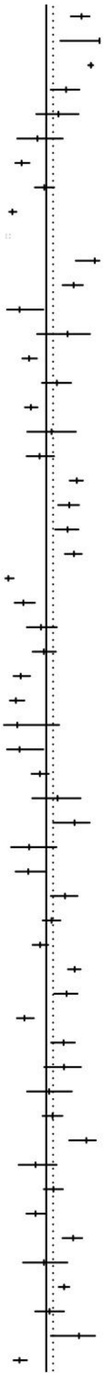

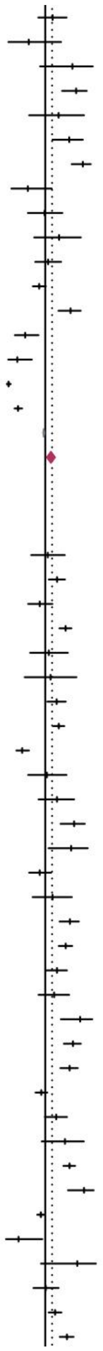

HuangJiaCheng 2021[944]	25	48	0.52 [0.37; 0.67]	0.2%	0.4%
ZhongJianMing 2014[946]	3	11	0.27 [0.06; 0.61]	0.1%	0.3%
Jun−Hua Zhou 2018[955]	8	11	0.73 [0.39; 0.94]	0.1%	0.3%
ZhouXiaoHong 2017[961]	36	47	0.77 [0.62; 0.88]	0.2%	0.4%
ZhouYin 2016[964]	7	12	0.58 [0.28; 0.85]	0.1%	0.3%
HuangKaiXiong 2021[989]	25	36	0.69 [0.52; 0.84]	0.2%	0.4%
Guo XH 2018[1000]	51	61	0.84 [0.72; 0.92]	0.3%	0.4%
Li J 2018[1015]	5	19	0.26 [0.09; 0.51]	0.1%	0.4%
Luo LF 2015[1030]	14	32	0.44 [0.26; 0.62]	0.1%	0.4%
WuLin 2021[1035]	10	17	0.59 [0.33; 0.82]	0.1%	0.4%
Shi C 2016[1046]	27	57	0.47 [0.34; 0.61]	0.3%	0.4%
Xu H 2013[1059−1]	79	207	0.38 [0.32; 0.45]	0.9%	0.4%
Xu H 2013[1059−2]	46	65	0.71 [0.58; 0.81]	0.3%	0.4%
XiangLunHui 2016[1101]	12	51	0.24 [0.13; 0.37]	0.2%	0.4%
ZouYong 2011[1103]	6	39	0.15 [0.06; 0.31]	0.2%	0.4%
HeFei 2020[1108]	38	610	0.06 [0.04; 0.08]	2.7%	0.5%
LiuYun 2019[1116]	57	353	0.16 [0.12; 0.20]	1.6%	0.5%
**Common effect model**		**11449**	**0.43 [0.42; 0.44]**	**51.8%**	**--**
**Random effects model**			**0.50 [0.46; 0.55]**	**--**	**54.9%**
Heterogeneity: $I^2 = 97\%$, $\tau^2 = 0.0628$, $p = 0$					
**age = 18−60y**					
Cai MW 2018[18]	16	34	0.47 [0.30; 0.65]	0.2%	0.4%
CaiWenFeng 2013[25]	80	141	0.57 [0.48; 0.65]	0.6%	0.4%
CaoShen 2019[32]	24	62	0.39 [0.27; 0.52]	0.3%	0.4%
ShenYuGang 2016[55]	153	233	0.66 [0.59; 0.72]	1.0%	0.4%
ChenGuoCui 2011[69]	13	27	0.48 [0.29; 0.68]	0.1%	0.4%
ChenHeJuan 2018[73]	7	14	0.50 [0.23; 0.77]	0.1%	0.4%
ChenJianMei 2017[79]	62	110	0.56 [0.47; 0.66]	0.5%	0.4%
ChenYiYi 2015[109]	165	282	0.59 [0.53; 0.64]	1.3%	0.5%
DuYao 2013[150]	32	157	0.20 [0.14; 0.28]	0.7%	0.4%
FengZhi 2020[161]	12	26	0.46 [0.27; 0.67]	0.1%	0.4%
GaoRiHong 2019[182]	17	30	0.57 [0.37; 0.75]	0.1%	0.4%
GaoShuPing 2019[183]	38	51	0.75 [0.60; 0.86]	0.2%	0.4%
GuKaiChen 2020[195]	15	21	0.71 [0.48; 0.89]	0.1%	0.4%
GuoLi 2019[205]	29	75	0.39 [0.28; 0.51]	0.3%	0.4%
HaoYongJian 2020[216]	13	25	0.52 [0.31; 0.72]	0.1%	0.4%
HuangGuo 2015[253]	61	87	0.70 [0.59; 0.79]	0.4%	0.4%
HuangLiQing 2020[254]	121	184	0.66 [0.58; 0.73]	0.8%	0.4%
HuangSiYue 2020[257]	51	90	0.57 [0.46; 0.67]	0.4%	0.4%
JiJinHua 2018[265]	22	41	0.54 [0.37; 0.69]	0.2%	0.4%
JiangChen 2019[285]	21	26	0.81 [0.61; 0.93]	0.1%	0.4%
LaiShiMing 2014[308]	77	105	0.73 [0.64; 0.81]	0.5%	0.4%
LiJie 2019[329]	74	106	0.70 [0.60; 0.78]	0.5%	0.4%
LiQun 2010[340]	104	258	0.40 [0.34; 0.47]	1.2%	0.4%
LiShiE 2018[349]	43	77	0.56 [0.44; 0.67]	0.3%	0.4%
LiXiuFang 2018[359]	13	20	0.65 [0.41; 0.85]	0.1%	0.4%
LiYiLan 2014[365]	159	228	0.70 [0.63; 0.76]	1.0%	0.4%
LiuJingJing 2018[413]	28	33	0.85 [0.68; 0.95]	0.2%	0.4%
LiuKaiQian 2011[415]	253	634	0.40 [0.36; 0.44]	2.9%	0.5%
LiuXiaoXiao 2014[430]	3	18	0.17 [0.04; 0.41]	0.1%	0.4%
LiuYi 2013[436]	7	9	0.78 [0.40; 0.97]	0.0%	0.3%
LuJianYong 2015[456]	27	60	0.45 [0.32; 0.58]	0.3%	0.4%
MaMengMeng 2018[484]	122	223	0.55 [0.48; 0.61]	1.0%	0.4%
QiYing 2018[535−1]	116	173	0.67 [0.60; 0.74]	0.8%	0.4%

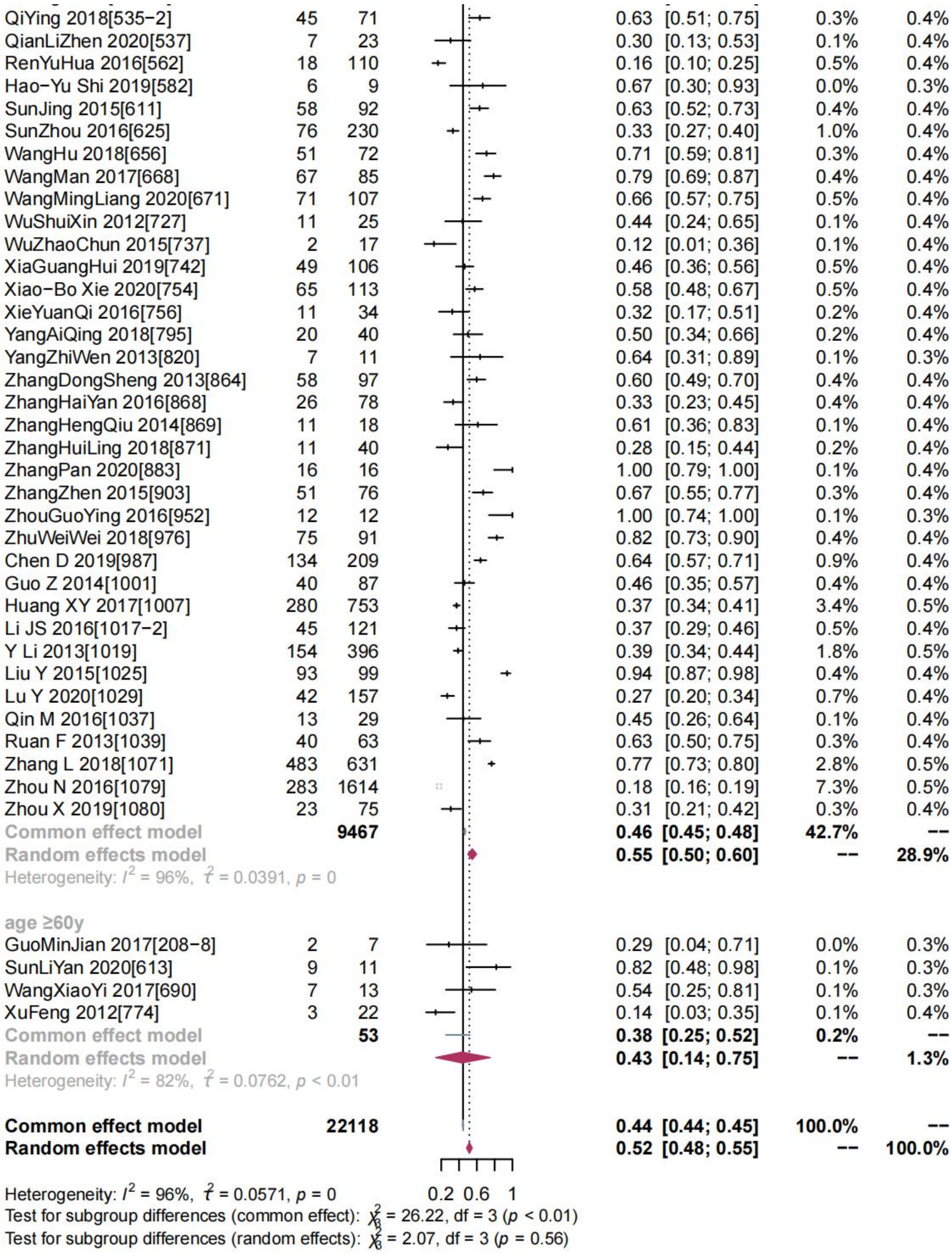
QiYing 2018[535−2] 45 71 0.63 [0.51; 0.75] 0.3% 0.4%
QianLiZhen 2020[537] 7 23 0.30 [0.13; 0.53] 0.1% 0.4%
RenYuHua 2016[562] 18 110 0.16 [0.10; 0.25] 0.5% 0.4%
Hao−Yu Shi 2019[582] 6 9 0.67 [0.30; 0.93] 0.0% 0.3%
SunJing 2015[611] 58 92 0.63 [0.52; 0.73] 0.4% 0.4%
SunZhou 2016[625] 76 230 0.33 [0.27; 0.40] 1.0% 0.4%
WangHu 2018[656] 51 72 0.71 [0.59; 0.81] 0.3% 0.4%
WangMan 2017[668] 67 85 0.79 [0.69; 0.87] 0.4% 0.4%
WangMingLiang 2020[671] 71 107 0.66 [0.57; 0.75] 0.5% 0.4%
WuShuiXin 2012[727] 11 25 0.44 [0.24; 0.65] 0.1% 0.4%
WuZhaoChun 2015[737] 2 17 0.12 [0.01; 0.36] 0.1% 0.4%
XiaGuangHui 2019[742] 49 106 0.46 [0.36; 0.56] 0.5% 0.4%
Xiao−Bo Xie 2020[754] 65 113 0.58 [0.48; 0.67] 0.5% 0.4%
XieYuanQi 2016[756] 11 34 0.32 [0.17; 0.51] 0.2% 0.4%
YangAiQing 2018[795] 20 40 0.50 [0.34; 0.66] 0.2% 0.4%
YangZhiWen 2013[820] 7 11 0.64 [0.31; 0.89] 0.1% 0.3%
ZhangDongSheng 2013[864] 58 97 0.60 [0.49; 0.70] 0.4% 0.4%
ZhangHaiYan 2016[868] 26 78 0.33 [0.23; 0.45] 0.4% 0.4%
ZhangHengQiu 2014[869] 11 18 0.61 [0.36; 0.83] 0.1% 0.4%
ZhangHuiLing 2018[871] 11 40 0.28 [0.15; 0.44] 0.2% 0.4%
ZhangPan 2020[883] 16 16 1.00 [0.79; 1.00] 0.1% 0.4%
ZhangZhen 2015[903] 51 76 0.67 [0.55; 0.77] 0.3% 0.4%
ZhouGuoYing 2016[952] 12 12 1.00 [0.74; 1.00] 0.1% 0.3%
ZhuWeiWei 2018[976] 75 91 0.82 [0.73; 0.90] 0.4% 0.4%
Chen D 2019[987] 134 209 0.64 [0.57; 0.71] 0.9% 0.4%
Guo Z 2014[1001] 40 87 0.46 [0.35; 0.57] 0.4% 0.4%
Huang XY 2017[1007] 280 753 0.37 [0.34; 0.41] 3.4% 0.5%
Li JS 2016[1017−2] 45 121 0.37 [0.29; 0.46] 0.5% 0.4%
Y Li 2013[1019] 154 396 0.39 [0.34; 0.44] 1.8% 0.5%
Liu Y 2015[1025] 93 99 0.94 [0.87; 0.98] 0.4% 0.4%
Lu Y 2020[1029] 42 157 0.27 [0.20; 0.34] 0.7% 0.4%
Qin M 2016[1037] 13 29 0.45 [0.26; 0.64] 0.1% 0.4%
Ruan F 2013[1039] 40 63 0.63 [0.50; 0.75] 0.3% 0.4%
Zhang L 2018[1071] 483 631 0.77 [0.73; 0.80] 2.8% 0.5%
Zhou N 2016[1079] 283 1614 0.18 [0.16; 0.19] 7.3% 0.5%
Zhou X 2019[1080] 23 75 0.31 [0.21; 0.42] 0.3% 0.4%
Common effect model 9467 0.46 [0.45; 0.48] 42.7% --
Random effects model 0.55 [0.50; 0.60] -- 28.9%
Heterogeneity: I^2 = 96%, τ^2 = 0.0391, p = 0
age ≥60y
GuoMinJian 2017[208−8] 2 7 0.29 [0.04; 0.71] 0.0% 0.3%
SunLiYan 2020[613] 9 11 0.82 [0.48; 0.98] 0.1% 0.3%
WangXiaoYi 2017[690] 7 13 0.54 [0.25; 0.81] 0.1% 0.3%
XuFeng 2012[774] 3 22 0.14 [0.03; 0.35] 0.1% 0.4%
Common effect model 53 0.38 [0.25; 0.52] 0.2% --
Random effects model 0.43 [0.14; 0.75] -- 1.3%
Heterogeneity: I^2 = 82%, τ^2 = 0.0762, p < 0.01
Common effect model 22118 0.44 [0.44; 0.45] 100.0% --
Random effects model 0.52 [0.48; 0.55] -- 100.0%
0.2 0.6 1
Heterogeneity: I^2 = 96%, τ^2 = 0.0571, p = 0
Test for subgroup differences (common effect): χ^2_3 = 26.22, df = 3 (p < 0.01)
Test for subgroup differences (random effects): χ^2_3 = 2.07, df = 3 (p = 0.56)

(e4)

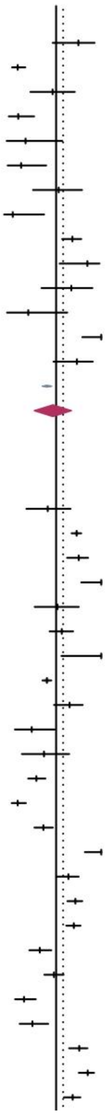

Study Nausea	Events	Total	Proportion	95%-CI	Weight (common)	Weight (random)
**age = 0−4y**						
Cai SX 2017[21]	13	17	0.76	[0.50; 0.93]	0.1%	0.6%
CaoXiaoPing 2018[33]	16	113	0.14	[0.08; 0.22]	0.7%	0.6%
HuGuangYi 2017[232]	10	20	0.50	[0.27; 0.73]	0.1%	0.6%
LinQin 2015[390]	5	34	0.15	[0.05; 0.31]	0.2%	0.6%
RenLiJun 2020[556]	2	9	0.22	[0.03; 0.60]	0.1%	0.5%
WangJun 2012[663]	3	17	0.18	[0.04; 0.43]	0.1%	0.6%
WangKaiLiang 2019[665]	9	16	0.56	[0.30; 0.80]	0.1%	0.5%
WangXiaoQin 2015[689]	1	11	0.09	[0.00; 0.41]	0.1%	0.5%
WuYang 2018[735]	64	91	0.70	[0.60; 0.79]	0.6%	0.6%
ZhangGuangMing 2018[907]	12	14	0.86	[0.57; 0.98]	0.1%	0.5%
ZhangGuoBao 2020[908]	9	13	0.69	[0.39; 0.91]	0.1%	0.5%
ZhaoMeiLing 2019[918−1]	2	8	0.25	[0.03; 0.65]	0.1%	0.5%
ZhuChunXiu 2019[968]	17	17	1.00	[0.80; 1.00]	0.1%	0.6%
Zhang TL 2017[1075]	15	20	0.75	[0.51; 0.91]	0.1%	0.6%
**Common effect model**		**400**	**0.44**	**[0.39; 0.49]**	**2.5%**	**--**
**Random effects model**			**0.50**	**[0.31; 0.69]**	**--**	**7.7%**
Heterogeneity: $I^2 = 93\%$, $\tau^2 = 0.1263$, $p < 0.01$						
**age = 5−17y**						
BaiAiLi 2020[3]	9	20	0.45	[0.23; 0.68]	0.1%	0.6%
ShenJiChuan 2011[50]	233	312	0.75	[0.69; 0.79]	2.0%	0.7%
ShenYiPing 2013[53]	50	65	0.77	[0.65; 0.86]	0.4%	0.6%
ChenCaiRong 2020[62]	16	16	1.00	[0.79; 1.00]	0.1%	0.5%
ChenJian 2016[78]	11	20	0.55	[0.32; 0.77]	0.1%	0.6%
ChenJian 2017[80]	41	69	0.59	[0.47; 0.71]	0.4%	0.6%
ChenXiaoFeng 2015[100]	7	7	1.00	[0.59; 1.00]	0.0%	0.4%
ChenXingHong 2012[102]	211	478	0.44	[0.40; 0.49]	3.0%	0.7%
ChenXingFu 2018[103]	29	43	0.67	[0.51; 0.81]	0.3%	0.6%
ChenYiXiong 2018[108]	6	21	0.29	[0.11; 0.52]	0.1%	0.6%
CuiXiaoMan 2018[125]	7	17	0.41	[0.18; 0.67]	0.1%	0.6%
DaiBenNa 2020[127]	39	117	0.33	[0.25; 0.43]	0.7%	0.6%
Ying−Hui Deng 2014[135]	14	99	0.14	[0.08; 0.23]	0.6%	0.6%
FengZhi 2018[162]	45	111	0.41	[0.31; 0.50]	0.7%	0.6%
FuXiaoFei 2012[171]	20	20	1.00	[0.83; 1.00]	0.1%	0.6%
GanXiangYang 2014[172]	49	74	0.66	[0.54; 0.77]	0.5%	0.6%
GongLiQiang 2013[192]	102	139	0.73	[0.65; 0.81]	0.9%	0.6%
HeXuXin 2017[222]	112	156	0.72	[0.64; 0.79]	1.0%	0.6%
HeHanZhen 2014[224]	28	76	0.37	[0.26; 0.49]	0.5%	0.6%
HuHongAn 2015[233]	54	105	0.51	[0.41; 0.61]	0.7%	0.6%
HuaWeiYu 2018[241]	13	63	0.21	[0.11; 0.33]	0.4%	0.6%
HuangBinBin 2020[245]	12	41	0.29	[0.16; 0.46]	0.3%	0.6%
HuangYanHong 2019[260]	65	84	0.77	[0.67; 0.86]	0.5%	0.6%
HuangYanHong 2019[261]	74	86	0.86	[0.77; 0.93]	0.5%	0.6%
JiRuPing 2020[274]	84	119	0.71	[0.62; 0.79]	0.8%	0.6%

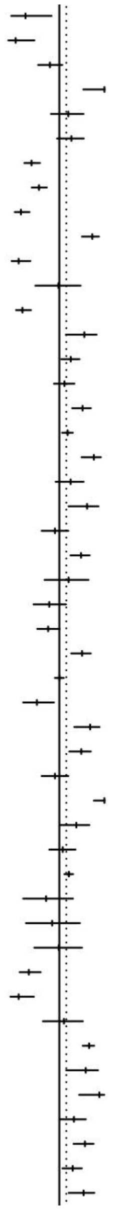

JiangXianChen 2014[283−1]	3	16	0.19	[0.04; 0.46]	0.1%	0.5%
JiangXianChen 2014[283−2]	2	23	0.09	[0.01; 0.28]	0.1%	0.6%
JiangYingCi 2015[284]	29	66	0.44	[0.32; 0.57]	0.4%	0.6%
JiangYiMei 2017[294]	15	15	1.00	[0.78; 1.00]	0.1%	0.5%
KangQian 2020[303−1]	22	35	0.63	[0.45; 0.79]	0.2%	0.6%
KangQian 2020[303−2]	33	50	0.66	[0.51; 0.79]	0.3%	0.6%
LiBo 2013[316]	30	120	0.25	[0.18; 0.34]	0.8%	0.6%
LiCaiYun 2012[318]	48	147	0.33	[0.25; 0.41]	0.9%	0.6%
LiShiE 2018[348]	13	92	0.14	[0.08; 0.23]	0.6%	0.6%
LiShouJun 2016[350]	56	64	0.88	[0.77; 0.94]	0.4%	0.6%
LiXiTai 2015[352]	6	51	0.12	[0.04; 0.24]	0.3%	0.6%
LiaoChan 2021[380]	10	19	0.53	[0.29; 0.76]	0.1%	0.6%
LinQiFeng 2018[388]	15	96	0.16	[0.09; 0.24]	0.6%	0.6%
LinQingShuang 2016[391]	23	29	0.79	[0.60; 0.92]	0.2%	0.6%
LiuBo 2015[398]	68	104	0.65	[0.55; 0.74]	0.7%	0.6%
LiuDan 2019[403]	53	90	0.59	[0.48; 0.69]	0.6%	0.6%
LiuDongSheng 2019[404]	62	80	0.78	[0.67; 0.86]	0.5%	0.6%
LiuShiKe 2012[421]	190	306	0.62	[0.56; 0.68]	1.9%	0.7%
LiuShiKe 2016[422]	41	46	0.89	[0.76; 0.96]	0.3%	0.6%
MaTao 2018[487]	30	46	0.65	[0.50; 0.79]	0.3%	0.6%
NiChaoRong 2019[511]	23	28	0.82	[0.63; 0.94]	0.2%	0.6%
NiChunYan 2020[512]	27	55	0.49	[0.35; 0.63]	0.3%	0.6%
PangZhiFeng 2017[524]	60	79	0.76	[0.65; 0.85]	0.5%	0.6%
PangZhiMing 2015[526]	12	19	0.63	[0.38; 0.84]	0.1%	0.6%
PengXiaoXue 2015[528]	16	37	0.43	[0.27; 0.61]	0.2%	0.6%
QiuHaiYan 2013[551]	32	76	0.42	[0.31; 0.54]	0.5%	0.6%
RenFuLin 2013[555]	57	74	0.77	[0.66; 0.86]	0.5%	0.6%
ShiChao 2013[579]	248	462	0.54	[0.49; 0.58]	2.9%	0.7%
SongCanLei 2017[593]	11	36	0.31	[0.16; 0.48]	0.2%	0.6%
SongHuiRong 2017[597]	29	34	0.85	[0.69; 0.95]	0.2%	0.6%
SongYuFang 2019[600]	48	63	0.76	[0.64; 0.86]	0.4%	0.6%
SuTong 2020[604]	27	55	0.49	[0.35; 0.63]	0.3%	0.6%
SunQin 2019[616]	32	32	1.00	[0.89; 1.00]	0.2%	0.6%
TianYaLin 2021[642]	27	38	0.71	[0.54; 0.85]	0.2%	0.6%
WangJinSheng 2019[645]	32	56	0.57	[0.43; 0.70]	0.4%	0.6%
WangJie 2015[659]	286	451	0.63	[0.59; 0.68]	2.9%	0.7%
WangTieJun 2020[678]	6	15	0.40	[0.16; 0.68]	0.1%	0.5%
WangXiaoYi 2017[691]	6	13	0.46	[0.19; 0.75]	0.1%	0.5%
WuGuoFu 2018[717]	9	17	0.53	[0.28; 0.77]	0.1%	0.6%
WuHongXing 2016[718]	14	63	0.22	[0.13; 0.34]	0.4%	0.6%
WuJingWen 2020[719]	4	34	0.12	[0.03; 0.27]	0.2%	0.6%
WuQinDi 2018[725]	14	24	0.58	[0.37; 0.78]	0.2%	0.6%
WuXiaoMin 2021[732]	134	159	0.84	[0.78; 0.90]	1.0%	0.6%
WuYang 2018[734]	21	26	0.81	[0.61; 0.93]	0.2%	0.6%
WuZhenYu 2012[738]	18	19	0.95	[0.74; 1.00]	0.1%	0.6%
XiaoSongJian 2017[745]	35	51	0.69	[0.54; 0.81]	0.3%	0.6%
XieBin 2020[758]	52	65	0.80	[0.68; 0.89]	0.4%	0.6%
XuJianRong 2018[767]	62	92	0.67	[0.57; 0.77]	0.6%	0.6%
YangCaiBin 2018[796]	33	42	0.79	[0.63; 0.90]	0.3%	0.6%

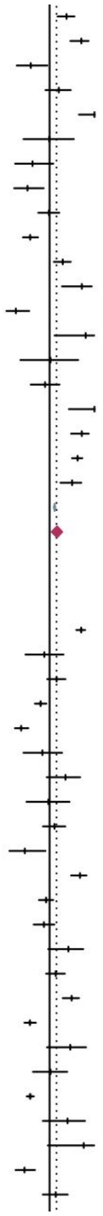

Study	Events	Total	Proportion	95% CI	Weight (common)	Weight (random)
YangJing 2018[804]	79	111	0.71	[0.62; 0.79]	0.7%	0.6%
YaoJianXiang 2016[822]	51	59	0.86	[0.75; 0.94]	0.4%	0.6%
ZhanYueWang 2019[860]	13	38	0.34	[0.20; 0.51]	0.2%	0.6%
ZhangQin 2020[885]	36	57	0.63	[0.49; 0.76]	0.4%	0.6%
ZhangXiaoYi 2015[891]	21	21	1.00	[0.84; 1.00]	0.1%	0.6%
ZhangYanMing 2019[898]	8	15	0.53	[0.27; 0.79]	0.1%	0.5%
ZhangZhengDong 2017[904]	9	25	0.36	[0.18; 0.57]	0.2%	0.6%
ZhaoMeiLing 2019[918−2]	12	39	0.31	[0.17; 0.48]	0.2%	0.6%
ZhaoMengJiao 2018[919]	46	87	0.53	[0.42; 0.64]	0.6%	0.6%
ZhaoXueCheng 2016[929]	50	149	0.34	[0.26; 0.42]	0.9%	0.6%
ZhaoYuLi 2019[933]	80	119	0.67	[0.58; 0.76]	0.8%	0.6%
ZhenGuoXin 2020[936]	20	23	0.87	[0.66; 0.97]	0.1%	0.6%
HuangJiaCheng 2021[944]	9	48	0.19	[0.09; 0.33]	0.3%	0.6%
ZhongJianMing 2014[946]	10	11	0.91	[0.59; 1.00]	0.1%	0.5%
Jun−Hua Zhou 2018[955]	6	11	0.55	[0.23; 0.83]	0.1%	0.5%
ZhouXiaoHong 2017[961]	23	47	0.49	[0.34; 0.64]	0.3%	0.6%
ZhouYin 2016[964]	12	12	1.00	[0.74; 1.00]	0.1%	0.5%
Guo XH 2018[1000]	53	61	0.87	[0.76; 0.94]	0.4%	0.6%
Xu H 2013[1059−1]	171	207	0.83	[0.77; 0.88]	1.3%	0.7%
Xu H 2013[1059−2]	50	65	0.77	[0.65; 0.86]	0.4%	0.6%
**Common effect model**		**7273**	**0.59**	**[0.58; 0.60]**	**46.3%**	**--**
**Random effects model**			**0.61**	**[0.55; 0.67]**	**--**	**57.0%**
Heterogeneity: $I^2 = 94\%$, $\tau^2 = 0.0810$, $p < 0.01$						
**age = 18−60y**						
ShenYuGang 2016[55]	201	233	0.86	[0.81; 0.90]	1.5%	0.7%
ChenGuoCui 2011[69]	13	27	0.48	[0.29; 0.68]	0.2%	0.6%
ChenJianMei 2017[79]	67	110	0.61	[0.51; 0.70]	0.7%	0.6%
ChenYiYi 2015[109]	125	282	0.44	[0.38; 0.50]	1.8%	0.7%
DuYao 2013[150]	38	157	0.24	[0.18; 0.32]	1.0%	0.6%
FengZhi 2020[161]	12	26	0.46	[0.27; 0.67]	0.2%	0.6%
GaoRiHong 2019[182]	21	30	0.70	[0.51; 0.85]	0.2%	0.6%
GuKaiChen 2020[195]	11	21	0.52	[0.30; 0.74]	0.1%	0.6%
GuoLi 2019[205]	44	75	0.59	[0.47; 0.70]	0.5%	0.6%
HaoYongJian 2020[216]	7	25	0.28	[0.12; 0.49]	0.2%	0.6%
HuangGuo 2015[253]	74	87	0.85	[0.76; 0.92]	0.6%	0.6%
HuangLiQing 2020[254]	92	184	0.50	[0.43; 0.57]	1.2%	0.6%
HuangSiYue 2020[257]	43	90	0.48	[0.37; 0.59]	0.6%	0.6%
JiangChen 2019[285]	19	26	0.73	[0.52; 0.88]	0.2%	0.6%
LaiShiMing 2014[308]	63	105	0.60	[0.50; 0.69]	0.7%	0.6%
LiJie 2019[329]	81	106	0.76	[0.67; 0.84]	0.7%	0.6%
LiQun 2010[340]	86	258	0.33	[0.28; 0.39]	1.6%	0.7%
LiXiuFang 2018[359]	15	20	0.75	[0.51; 0.91]	0.1%	0.6%
LiuJingJing 2018[413]	18	33	0.55	[0.36; 0.72]	0.2%	0.6%
LiuKaiQian 2011[415]	213	634	0.34	[0.30; 0.37]	4.0%	0.7%
LiuXiaoXiao 2014[430]	13	18	0.72	[0.47; 0.90]	0.1%	0.6%
LiuYi 2013[436]	8	9	0.89	[0.52; 1.00]	0.1%	0.5%
LuZhengXiang 2016[451]	23	83	0.28	[0.18; 0.39]	0.5%	0.6%
LuJianYong 2015[456]	36	60	0.60	[0.47; 0.72]	0.4%	0.6%

LuWeiWei 2016[462]	175	406	0.43	[0.38; 0.48]	2.6%	0.7%
QiYing 2018[535−1]	145	173	0.84	[0.77; 0.89]	1.1%	0.6%
QiYing 2018[535−2]	61	71	0.86	[0.76; 0.93]	0.5%	0.6%
RenYuHua 2016[562]	52	110	0.47	[0.38; 0.57]	0.7%	0.6%
Hao−Yu Shi 2019[582]	5	9	0.56	[0.21; 0.86]	0.1%	0.5%
SunJing 2015[611]	66	92	0.72	[0.61; 0.81]	0.6%	0.6%
WangBing 2017[649]	52	58	0.90	[0.79; 0.96]	0.4%	0.6%
WangHu 2018[656]	53	72	0.74	[0.62; 0.83]	0.5%	0.6%
WangMingLiang 2020[671]	61	107	0.57	[0.47; 0.67]	0.7%	0.6%
WeiYiYun 2015[705]	16	22	0.73	[0.50; 0.89]	0.1%	0.6%
WuPei 2011[724]	146	146	1.00	[0.98; 1.00]	0.9%	0.6%
WuShuiXin 2012[727]	21	25	0.84	[0.64; 0.95]	0.2%	0.6%
WuZhaoChun 2015[737]	8	17	0.47	[0.23; 0.72]	0.1%	0.6%
Xiao−Bo Xie 2020[754]	95	113	0.84	[0.76; 0.90]	0.7%	0.6%
XuanLingFeng 2014[784]	10	17	0.59	[0.33; 0.82]	0.1%	0.6%
YangAiQing 2018[795]	35	40	0.88	[0.73; 0.96]	0.3%	0.6%
YangZhiWen 2013[820]	4	11	0.36	[0.11; 0.69]	0.1%	0.5%
ZhangHaiYan 2016[868]	17	78	0.22	[0.13; 0.33]	0.5%	0.6%
ZhangHengQiu 2014[869]	13	18	0.72	[0.47; 0.90]	0.1%	0.6%
ZhangHuiLing 2018[871]	17	40	0.42	[0.27; 0.59]	0.3%	0.6%
ZhangPan 2020[883]	8	16	0.50	[0.25; 0.75]	0.1%	0.5%
ZhangZhen 2015[903]	46	76	0.61	[0.49; 0.72]	0.5%	0.6%
ZhouGuoYing 2016[952]	12	12	1.00	[0.74; 1.00]	0.1%	0.5%
Chen D 2019[987]	147	209	0.70	[0.64; 0.76]	1.3%	0.7%
Guo Z 2014[1001]	57	87	0.66	[0.55; 0.75]	0.6%	0.6%
Huang XY 2017[1007]	328	753	0.44	[0.40; 0.47]	4.8%	0.7%
Li JS 2016[1017−2]	51	121	0.42	[0.33; 0.51]	0.8%	0.6%
Qin M 2016[1037]	17	29	0.59	[0.39; 0.76]	0.2%	0.6%
Ruan F 2013[1039]	50	63	0.79	[0.67; 0.89]	0.4%	0.6%
Zhang L 2018[1071]	446	631	0.71	[0.67; 0.74]	4.0%	0.7%
Zhou N 2016[1079]	325	1614	0.20	[0.18; 0.22]	10.3%	0.7%
Zhou X 2019[1080]	40	75	0.53	[0.41; 0.65]	0.5%	0.6%
Common effect model		**8010**	**0.49**	**[0.48; 0.50]**	**51.0%**	**--**
Random effects model			**0.62**	**[0.56; 0.68]**	**--**	**34.2%**
Heterogeneity: $I^2 = 97\%$, $\tau^2 = 0.0519$, $p = 0$						
age = =60y						
SunLiYan 2020[613]	10	11	0.91	[0.59; 1.00]	0.1%	0.5%
XuFeng 2012[774]	13	22	0.59	[0.36; 0.79]	0.1%	0.6%
Common effect model		**33**	**0.71**	**[0.55; 0.85]**	**0.2%**	**--**
Random effects model			**0.76**	**[0.39; 0.98]**	**--**	**1.1%**
Heterogeneity: $I^2 = 77\%$, $\tau^2 = 0.0581$, $p = 0.04$						
**Common effect model**		**15716**	**0.54**	**[0.53; 0.54]**	**100.0%**	**--**
**Random effects model**			**0.61**	**[0.57; 0.65]**	**--**	**100.0%**

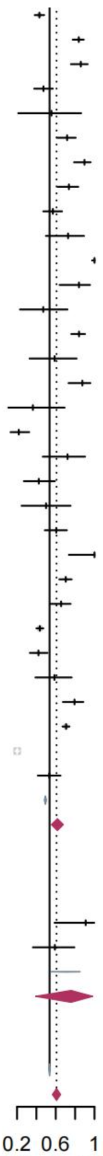

Heterogeneity: $I^2 = 96\%$, $\tau^2 = 0.0730$, $p = 0$

Test for subgroup differences (common effect): $\chi^2_3 = 157.00$, df = 3 ($p < 0.01$)

Test for subgroup differences (random effects): $\chi^2_3 = 1.90$, df = 3 ($p = 0.59$)

(e5)

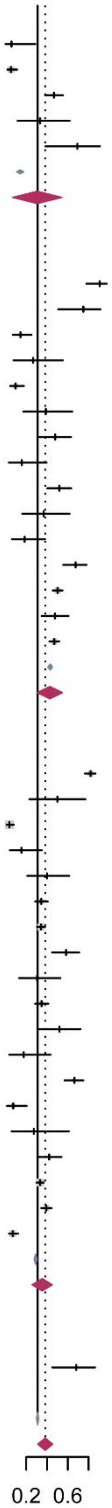

Study	Abdominal distension	Events	Total	Proportion	95%−CI	Weight (common)	Weight (random)
**age = 0−4y**							
Cai SX 2017[21]		1	17	0.06	[0.00; 0.29]	1.0%	2.6%
CaoXiaoPing 2018[33]		6	113	0.05	[0.02; 0.11]	7.1%	2.7%
CuiXiaoShuang 2016[126]		64	137	0.47	[0.38; 0.55]	1.7%	2.7%
LiChunLing 2020[319]		5	15	0.33	[0.12; 0.62]	0.2%	2.1%
ZhangGuoBao 2020[908]		9	13	0.69	[0.39; 0.91]	0.2%	2.1%
**Common effect model**			**295**	**0.14**	**[0.11; 0.18]**	**10.3%**	**--**
**Random effects model**				**0.31**	**[0.07; 0.54]**	**--**	**12.2%**
Heterogeneity: $I^2 = 96\%$, $\tau^2 = 0.0661$, $p < 0.01$							
**age = 5−17y**							
ShenYuGang 2016[54]		39	43	0.91	[0.78; 0.97]	1.6%	2.7%
FuXiaoFei 2012[171]		15	20	0.75	[0.51; 0.91]	0.3%	2.3%
HuangGuo 2015[252]		10	69	0.14	[0.07; 0.25]	1.8%	2.7%
JiangYiMei 2017[294]		4	15	0.27	[0.08; 0.55]	0.2%	2.2%
LiShiE 2018[348]		9	92	0.10	[0.05; 0.18]	3.3%	2.7%
LiYueRong 2015[366]		7	18	0.39	[0.17; 0.64]	0.2%	2.2%
MaTao 2018[487]		22	46	0.48	[0.33; 0.63]	0.6%	2.5%
MoYuJie 2018[505]		3	19	0.16	[0.03; 0.40]	0.5%	2.4%
PangZhiFeng 2017[524]		41	79	0.52	[0.40; 0.63]	1.0%	2.6%
PangZhiMing 2015[526]		7	19	0.37	[0.16; 0.62]	0.3%	2.2%
QiYanQiu 2018[532]		5	27	0.19	[0.06; 0.38]	0.6%	2.5%
RenFuLin 2013[555]		50	74	0.68	[0.56; 0.78]	1.1%	2.6%
ShiChao 2013[579]		232	462	0.50	[0.46; 0.55]	5.9%	2.7%
SongYuFang 2019[600]		30	63	0.48	[0.35; 0.61]	0.8%	2.6%
WangJie 2015[659]		212	451	0.47	[0.42; 0.52]	5.7%	2.7%
**Common effect model**			**1497**	**0.43**	**[0.41; 0.45]**	**23.9%**	**--**
**Random effects model**				**0.43**	**[0.31; 0.55]**	**--**	**37.5%**
Heterogeneity: $I^2 = 96\%$, $\tau^2 = 0.0523$, $p < 0.01$							
**age = 18−60y**							
ShenYuGang 2016[55]		191	233	0.82	[0.76; 0.87]	5.0%	2.7%
ChenHeJuan 2018[73]		7	14	0.50	[0.23; 0.77]	0.2%	2.0%
DuYao 2013[150]		6	157	0.04	[0.01; 0.08]	13.6%	2.7%
FengZhi 2020[161]		4	26	0.15	[0.04; 0.35]	0.6%	2.5%
HaoYongJian 2020[216]		10	25	0.40	[0.21; 0.61]	0.3%	2.3%
LiQun 2010[340]		89	258	0.34	[0.29; 0.41]	3.6%	2.7%
LiuKaiQian 2011[415]		217	634	0.34	[0.31; 0.38]	8.9%	2.7%
LuJianYong 2015[456]		35	60	0.58	[0.45; 0.71]	0.8%	2.5%
QianLiZhen 2020[537]		7	23	0.30	[0.13; 0.53]	0.3%	2.3%
SunZhou 2016[625]		80	230	0.35	[0.29; 0.41]	3.2%	2.7%
WuShuiXin 2012[727]		13	25	0.52	[0.31; 0.72]	0.3%	2.3%
WuZhaoChun 2015[737]		3	17	0.18	[0.04; 0.43]	0.4%	2.3%
Xiao−Bo Xie 2020[754]		75	113	0.66	[0.57; 0.75]	1.6%	2.7%
YangAiQing 2018[795]		3	40	0.07	[0.02; 0.20]	1.8%	2.7%
YangZhiWen 2013[820]		3	11	0.27	[0.06; 0.61]	0.2%	2.0%
ZhangZhen 2015[903]		32	76	0.42	[0.31; 0.54]	1.0%	2.6%
Huang XY 2017[1007]		251	753	0.33	[0.30; 0.37]	10.8%	2.7%
Y Li 2013[1019]		156	396	0.39	[0.35; 0.44]	5.3%	2.7%
Lu Y 2020[1029]		11	157	0.07	[0.04; 0.12]	7.6%	2.7%
**Common effect model**			**3248**	**0.29**	**[0.28; 0.30]**	**65.6%**	**--**
**Random effects model**				**0.35**	**[0.26; 0.45]**	**--**	**48.1%**
Heterogeneity: $I^2 = 98\%$, $\tau^2 = 0.0421$, $p < 0.01$							
**age≥60y**							
XuFeng 2012[774]		15	22	0.68	[0.45; 0.86]	0.3%	2.3%
**Common effect model**			**5062**	**0.31**	**[0.30; 0.32]**	**100.0%**	**--**
**Random effects model**				**0.38**	**[0.31; 0.45]**	**--**	**100.0%**

Heterogeneity: $I^2 = 98\%$, $\tau^2 = 0.0486$, $p = 0$

Test for subgroup differences (common effect): $\chi^2_3 = 223.27$, df = 3 ($p < 0.01$)

Test for subgroup differences (random effects): $\chi^2_3 = 9.57$, df = 3 ($p = 0.02$)

(e6)

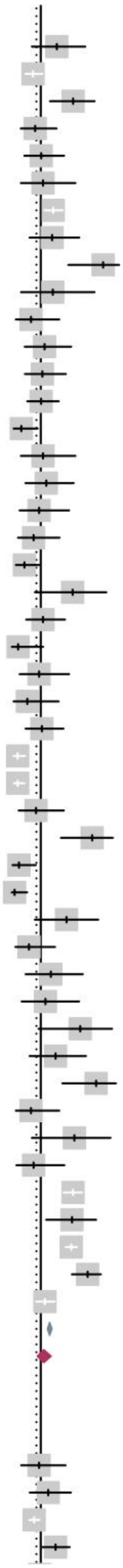

Study Fever	Events	Total	Proportion	95%−CI
age = 0−4y				
Cai SX 2017[21]	7	17	0.41	[0.18; 0.67]
CaoXiaoPing 2018[33]	22	113	0.19	[0.13; 0.28]
ZengFengMei 2020[41]	14	25	0.56	[0.35; 0.76]
ZengLei 2018[44]	6	28	0.21	[0.08; 0.41]
ShenYiPing 2018[52]	7	26	0.27	[0.12; 0.48]
ChenJingFang 2018[84−2]	4	14	0.29	[0.08; 0.58]
CuiXiaoShuang 2016[126]	52	137	0.38	[0.30; 0.47]
GaoHaiMing 2014[174]	7	19	0.37	[0.16; 0.62]
GaoZhiYong 2017[187−1]	10	12	0.83	[0.52; 0.98]
GuoMinJian 2017[208−1]	3	8	0.38	[0.09; 0.76]
GuoMinJian 2017[208−5]	3	17	0.18	[0.04; 0.43]
HuGuangYi 2017[232]	6	20	0.30	[0.12; 0.54]
LiangPing 2020[374]	7	25	0.28	[0.12; 0.49]
LiaoKeChang 2018[381]	11	41	0.27	[0.14; 0.43]
LinQin 2015[390]	3	34	0.09	[0.02; 0.24]
LuHua 2016[454]	4	14	0.29	[0.08; 0.58]
LuCaiFang 2020[458]	6	19	0.32	[0.13; 0.57]
MengXiangJie 2012[499]	4	16	0.25	[0.07; 0.52]
PangZhiFeng 2014[525]	4	20	0.20	[0.06; 0.44]
RenFuLin 2013[554]	5	43	0.12	[0.04; 0.25]
RenLiJun 2020[556]	5	9	0.56	[0.21; 0.86]
WangHua 2014[657]	8	28	0.29	[0.13; 0.49]
WangJun 2012[663]	1	17	0.06	[0.00; 0.29]
WangKaiLiang 2019[665]	4	16	0.25	[0.07; 0.52]
Ya−Xin Wang 2020[693]	2	14	0.14	[0.02; 0.43]
WeiGuiYing 2019[706]	8	29	0.28	[0.13; 0.47]
WuYang 2018[735]	5	91	0.05	[0.02; 0.12]
YangCheng 2017[797]	6	109	0.06	[0.02; 0.12]
YangJiChao 2016[802]	4	18	0.22	[0.06; 0.48]
YangRongXing 2019[808]	11	15	0.73	[0.45; 0.92]
YangSenPing 2018[810]	2	30	0.07	[0.01; 0.22]
YinHongMei 2014[839]	1	37	0.03	[0.00; 0.14]
YueYong 2017[859]	6	12	0.50	[0.21; 0.79]
ZhangAiHua 2018[861]	3	19	0.16	[0.03; 0.40]
ZhangGuangMing 2018[907]	5	14	0.36	[0.13; 0.65]
ZhangGuoBao 2020[908]	4	13	0.31	[0.09; 0.61]
ZhaoMeiLing 2019[918−1]	5	8	0.62	[0.24; 0.91]
ZhouJianHong 2013[954]	6	15	0.40	[0.16; 0.68]
ZhouLiHong 2016[956]	10	13	0.77	[0.46; 0.95]
ZhuChunXiu 2019[968]	3	17	0.18	[0.04; 0.43]
LiXiaoJing 2021[992]	4	7	0.57	[0.18; 0.90]
S Kim 2019[1012]	3	15	0.20	[0.04; 0.48]
Y Li 2021[1018]	80	143	0.56	[0.47; 0.64]
Zhang TL 2017[1075]	11	20	0.55	[0.32; 0.77]
Zou W 2015[1085]	208	383	0.54	[0.49; 0.59]
MaYan 2014[1118]	36	52	0.69	[0.55; 0.81]
SaiLinTao 2014[1120]	37	122	0.30	[0.22; 0.39]
**Common effect model**		**1914**	**0.35**	**[0.33; 0.37]**
**Random effects model**			**0.29**	**[0.23; 0.36]**
Heterogeneity: $I^2 = 82\%$, $\tau^2 = 0.8941$, $p < 0.01$				
age = 5−17y				
BaiAiLi 2020[3]	5	20	0.25	[0.09; 0.49]
CaiWenFeng 2014[26]	9	27	0.33	[0.17; 0.54]
ShenJiChuan 2011[50]	64	312	0.21	[0.16; 0.25]
ShenYiPing 2013[53]	26	65	0.40	[0.28; 0.53]

ShenYuGang 2016[54]	11	43	0.26 [0.14; 0.41]
ChenCaiRong 2020[62]	4	16	0.25 [0.07; 0.52]
ChenChun 2014[64]	50	107	0.47 [0.37; 0.57]
ChenJian 2016[78]	16	20	0.80 [0.56; 0.94]
ChenJian 2017[80]	22	69	0.32 [0.21; 0.44]
ChenXiaoFeng 2015[100]	3	7	0.43 [0.10; 0.82]
ChenXingHong 2012[102]	45	478	0.09 [0.07; 0.12]
ChenXingFu 2018[103]	5	43	0.12 [0.04; 0.25]
ChenYiXiong 2018[108]	8	21	0.38 [0.18; 0.62]
CuiXiaoMan 2018[125]	5	17	0.29 [0.10; 0.56]
DaiBenNa 2020[127]	25	117	0.21 [0.14; 0.30]
Ying−Hui Deng 2014[135]	28	99	0.28 [0.20; 0.38]
FangYuLian 2019[155]	147	241	0.61 [0.55; 0.67]
FangYuLian 2021[156]	560	809	0.69 [0.66; 0.72]
FengZhi 2018[162]	24	111	0.22 [0.14; 0.30]
FuXiaoFei 2012[171]	4	20	0.20 [0.06; 0.44]
GanXiangYang 2014[172]	27	74	0.36 [0.26; 0.48]
GaoHuiJuan 2015[176]	2	18	0.11 [0.01; 0.35]
GaoPeng 2020[181]	3	28	0.11 [0.02; 0.28]
GaoZhiYong 2017[187−2]	5	10	0.50 [0.19; 0.81]
GaoZhiYong 2017[187−4]	10	16	0.62 [0.35; 0.85]
GaoZhiYong 2017[187−6]	20	25	0.80 [0.59; 0.93]
GongLiQiang 2013[192]	18	139	0.13 [0.08; 0.20]
GuYiFu 2020[197]	20	45	0.44 [0.30; 0.60]
GuoJing 2019[203]	99	193	0.51 [0.44; 0.59]
GuoMinJian 2017[208−3]	3	11	0.27 [0.06; 0.61]
GuoMinJian 2017[208−4]	2	10	0.20 [0.03; 0.56]
GuoMinJian 2017[208−9]	2	16	0.12 [0.02; 0.38]
GuoMinJian 2017[208−10]	5	48	0.10 [0.03; 0.23]
HeXuXin 2017[222]	12	156	0.08 [0.04; 0.13]
HeHanZhen 2014[224]	7	76	0.09 [0.04; 0.18]
HouYuYuan 2014[231]	29	63	0.46 [0.33; 0.59]
HuHongAn 2015[233]	13	105	0.12 [0.07; 0.20]
Ying Hu 2019[239]	14	120	0.12 [0.07; 0.19]
HuaWeiYu 2018[241]	18	63	0.29 [0.18; 0.41]
HuangGuo 2015[252]	26	69	0.38 [0.26; 0.50]
HuangYanHong 2019[260]	21	84	0.25 [0.16; 0.36]
HuangYanHong 2019[261]	10	86	0.12 [0.06; 0.20]
Lei Ji 2021[269]	2	26	0.08 [0.01; 0.25]
JiRuPing 2020[274]	55	119	0.46 [0.37; 0.56]
JiangXianChen 2014[283−1]	2	16	0.12 [0.02; 0.38]
JiangXianChen 2014[283−2]	1	23	0.04 [0.00; 0.22]
JiangYiMei 2017[294]	4	15	0.27 [0.08; 0.55]
KangQian 2020[303−1]	8	35	0.23 [0.10; 0.40]
KangQian 2020[303−2]	15	50	0.30 [0.18; 0.45]
LiBo 2013[316]	4	120	0.03 [0.01; 0.08]
LiCaiYun 2012[318]	8	147	0.05 [0.02; 0.10]
LiShiCong 2020[344]	50	181	0.28 [0.21; 0.35]
LiShiCong 2018[346]	21	64	0.33 [0.22; 0.46]
LiShiE 2018[348]	5	92	0.05 [0.02; 0.12]
LiShouJun 2016[350]	20	64	0.31 [0.20; 0.44]
LiXiTai 2015[352]	20	51	0.39 [0.26; 0.54]
LiYueRong 2015[366]	3	18	0.17 [0.04; 0.41]
LiangRiCheng 2017[376]	9	27	0.33 [0.17; 0.54]
LiaoChan 2021[380]	3	19	0.16 [0.03; 0.40]
LinQiFeng 2018[388]	20	96	0.21 [0.13; 0.30]
LinQingShuang 2016[391]	4	29	0.14 [0.04; 0.32]
LiuBaiWei 2017[395]	5	24	0.21 [0.07; 0.42]
LiuBo 2015[398]	1	104	0.01 [0.00; 0.05]
LiuDan 2019[403]	4	90	0.04 [0.01; 0.11]
LiuDongSheng 2019[404]	27	80	0.34 [0.24; 0.45]

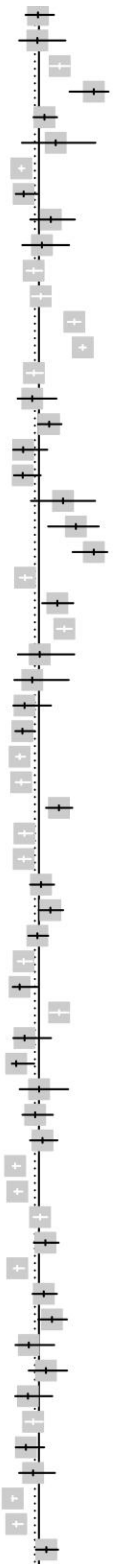

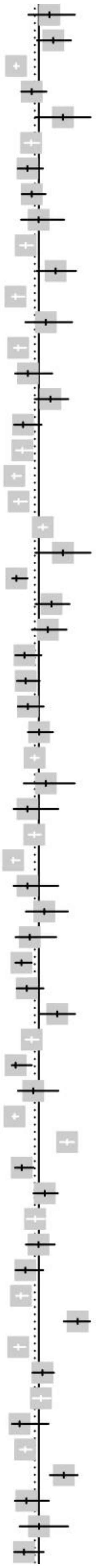

LiuHongLian 2018[411]	7	19	0.37 [0.16; 0.62]
LiuQingLian 2019[420]	15	37	0.41 [0.25; 0.58]
LiuShiKe 2012[421]	13	306	0.04 [0.02; 0.07]
LiuShiKe 2016[422]	9	46	0.20 [0.09; 0.34]
LiuYing 2019[437]	7	14	0.50 [0.23; 0.77]
LiuYuan 2016[438−2]	23	121	0.19 [0.12; 0.27]
LuoLe 2017[472]	6	39	0.15 [0.06; 0.31]
MaoJianYing 2016[493]	10	51	0.20 [0.10; 0.33]
MoYuJie 2018[505]	5	19	0.26 [0.09; 0.51]
NiChaoRong 2019[510]	17	125	0.14 [0.08; 0.21]
NiChaoRong 2019[511]	12	28	0.43 [0.24; 0.63]
NiChunYan 2020[512]	2	55	0.04 [0.00; 0.13]
OuSheXiang 2019[515]	6	18	0.33 [0.13; 0.59]
PangZhiFeng 2017[524]	5	79	0.06 [0.02; 0.14]
PangZhiMing 2015[526]	3	19	0.16 [0.03; 0.40]
PengXiaoXue 2015[528]	14	37	0.38 [0.22; 0.55]
QiYanQiu 2018[532]	3	27	0.11 [0.02; 0.29]
QiaoYingQin 2016[543]	7	67	0.10 [0.04; 0.20]
QiuHaiYan 2013[551]	2	76	0.03 [0.00; 0.09]
RenFuLin 2013[555]	5	74	0.07 [0.02; 0.15]
ShiChao 2013[579]	140	462	0.30 [0.26; 0.35]
ShuaiHuiQun 2012[589]	7	14	0.50 [0.23; 0.77]
SongCanLei 2020[590]	2	44	0.05 [0.01; 0.15]
SongCanLei 2017[593]	14	36	0.39 [0.23; 0.57]
SongHuiRong 2017[597]	12	34	0.35 [0.20; 0.54]
SunQin 2019[616]	4	32	0.12 [0.04; 0.29]
TangYuHuan 2017[630]	6	44	0.14 [0.05; 0.27]
TianYaLin 2021[642]	6	38	0.16 [0.06; 0.31]
WangJinSheng 2019[645]	15	56	0.27 [0.16; 0.40]
WangJie 2015[659]	101	451	0.22 [0.19; 0.27]
WangTieJun 2020[678]	5	15	0.33 [0.12; 0.62]
WangWeiRu 2013[680]	2	13	0.15 [0.02; 0.45]
WangWenYing 2011[681]	45	204	0.22 [0.17; 0.28]
WangXiaoDong 2017[688]	1	69	0.01 [0.00; 0.08]
WangXiaoYi 2017[691]	2	13	0.15 [0.02; 0.45]
WeiXia 2019[708]	7	22	0.32 [0.14; 0.55]
WuGuoFu 2018[717]	3	17	0.18 [0.04; 0.43]
WuHongXing 2016[718]	6	63	0.10 [0.04; 0.20]
WuJingWen 2020[719]	5	34	0.15 [0.05; 0.31]
WuWenQian 2018[729]	16	36	0.44 [0.28; 0.62]
WuXiaoMin 2021[732]	31	159	0.19 [0.14; 0.27]
WuYang 2018[734]	1	26	0.04 [0.00; 0.20]
WuZhenYu 2012[738]	4	19	0.21 [0.06; 0.46]
XiaYingPin 2018[743]	5	177	0.03 [0.01; 0.06]
XiaZhongFa 2012[744]	113	209	0.54 [0.47; 0.61]
XiaoSongJian 2017[745]	5	51	0.10 [0.03; 0.21]
XieBin 2020[758]	21	65	0.32 [0.21; 0.45]
XuJianRong 2018[767]	21	92	0.23 [0.15; 0.33]
YangCaiBin 2018[796]	11	42	0.26 [0.14; 0.42]
YangJiXing 2019[801]	4	30	0.13 [0.04; 0.31]
YangJing 2018[804]	10	111	0.09 [0.04; 0.16]
YaoJianXiang 2016[822]	38	59	0.64 [0.51; 0.76]
YeXianMing 2017[832]	6	94	0.06 [0.02; 0.13]
YuHong 2016[848]	24	80	0.30 [0.20; 0.41]
Yan Yu 2017[852]	32	112	0.29 [0.20; 0.38]
ZhangChong 2015[862]	1	13	0.08 [0.00; 0.36]
ZhangHaiYan 2019[867]	19	145	0.13 [0.08; 0.20]
ZhangQin 2020[885]	29	57	0.51 [0.37; 0.64]
ZhangXiaoYi 2015[891]	3	21	0.14 [0.03; 0.36]
ZhangYanMing 2019[898]	4	15	0.27 [0.08; 0.55]
ZhangZhengDong 2017[904]	3	25	0.12 [0.03; 0.31]

ZhaoMeiLing 2019[918−2]	5	39	0.13	[0.04; 0.27]
ZhaoMengJiao 2018[919]	58	87	0.67	[0.56; 0.76]
ZhaoXueCheng 2016[929]	1	149	0.01	[0.00; 0.04]
ZhaoYuLi 2019[933]	31	119	0.26	[0.18; 0.35]
HuangJiaCheng 2021[944]	33	48	0.69	[0.54; 0.81]
ZhongJianMing 2014[946]	4	11	0.36	[0.11; 0.69]
ZhouYin 2016[964]	4	12	0.33	[0.10; 0.65]
ZhuChengMing 2017[967]	1	78	0.01	[0.00; 0.07]
Guo XH 2018[1000]	24	61	0.39	[0.27; 0.53]
Li J 2018[1015]	1	19	0.05	[0.00; 0.26]
Luo LF 2015[1030]	5	32	0.16	[0.05; 0.33]
WuLin 2021[1035]	5	17	0.29	[0.10; 0.56]
Shi C 2016[1046]	4	57	0.07	[0.02; 0.17]
Xu H 2013[1059−1]	58	207	0.28	[0.22; 0.35]
Xu H 2013[1059−2]	16	65	0.25	[0.15; 0.37]
XiangLunHui 2016[1101]	10	51	0.20	[0.10; 0.33]
ChenCen 2016[1105]	14	52	0.27	[0.16; 0.41]
HeFei 2020[1108]	369	610	0.60	[0.56; 0.64]
LiuYun 2019[1116]	191	353	0.54	[0.49; 0.59]
**Common effect model**		**12210**	**0.29**	**[0.28; 0.30]**
**Random effects model**			**0.21**	**[0.18; 0.24]**
Heterogeneity: I^2 = 93%, τ^2 = 1.0499, p < 0.01				
**age = 18−60y**				
Cai MW 2018[18]	2	34	0.06	[0.01; 0.20]
CaiWenFeng 2013[25]	40	141	0.28	[0.21; 0.37]
CaoShen 2019[32]	1	62	0.02	[0.00; 0.09]
ShenYuGang 2016[55]	31	233	0.13	[0.09; 0.18]
ChenAQun 2016[59]	27	64	0.42	[0.30; 0.55]
ChenGuoCui 2011[69]	5	27	0.19	[0.06; 0.38]
ChenJianMei 2017[79]	38	110	0.35	[0.26; 0.44]
ChenYiYi 2015[109]	19	282	0.07	[0.04; 0.10]
DuYao 2013[150]	7	157	0.04	[0.02; 0.09]
FengZhi 2020[161]	3	26	0.12	[0.02; 0.30]
GaoRiHong 2019[182]	7	30	0.23	[0.10; 0.42]
GaoShuPing 2019[183]	8	51	0.16	[0.07; 0.29]
GaoZhiYong 2017[187−3]	13	15	0.87	[0.60; 0.98]
GaoZhiYong 2017[187−8]	18	22	0.82	[0.60; 0.95]
GuKaiChen 2020[195]	9	21	0.43	[0.22; 0.66]
GuoLi 2019[205]	23	75	0.31	[0.21; 0.42]
HaoYongJian 2020[216]	19	25	0.76	[0.55; 0.91]
HuangGuo 2015[253]	40	87	0.46	[0.35; 0.57]
HuangLiQing 2020[254]	74	184	0.40	[0.33; 0.48]
HuangSiYue 2020[257]	9	90	0.10	[0.05; 0.18]
JiJinHua 2018[265]	16	41	0.39	[0.24; 0.55]
JiLei 2020[270]	11	19	0.58	[0.33; 0.80]
JiangChen 2019[285]	3	26	0.12	[0.02; 0.30]
KuangHaoCheng 2016[307]	117	667	0.18	[0.15; 0.21]
LaiShiMing 2014[308]	10	105	0.10	[0.05; 0.17]
LiJie 2019[329]	18	106	0.17	[0.10; 0.26]
LiQun 2010[340]	7	258	0.03	[0.01; 0.06]
LiShiE 2018[349]	47	77	0.61	[0.49; 0.72]
LiXiuFang 2018[359]	1	20	0.05	[0.00; 0.25]
LiYiLan 2014[365]	110	228	0.48	[0.42; 0.55]
LiuJingJing 2018[413]	3	33	0.09	[0.02; 0.24]
LiuKaiQian 2011[415]	17	634	0.03	[0.02; 0.04]
LiuXiaoXiao 2014[430]	6	18	0.33	[0.13; 0.59]
LuJianYong 2015[456]	11	60	0.18	[0.10; 0.30]
LuWeiWei 2016[462]	49	406	0.12	[0.09; 0.16]
MaMengMeng 2018[484]	97	223	0.43	[0.37; 0.50]

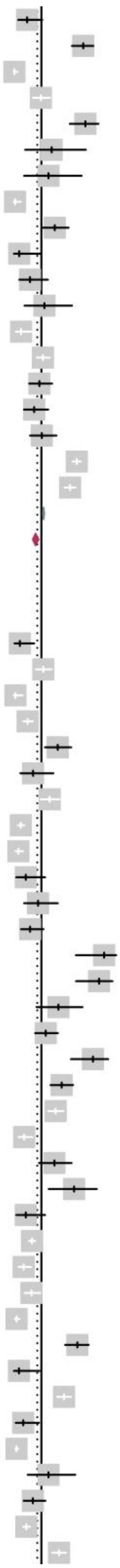

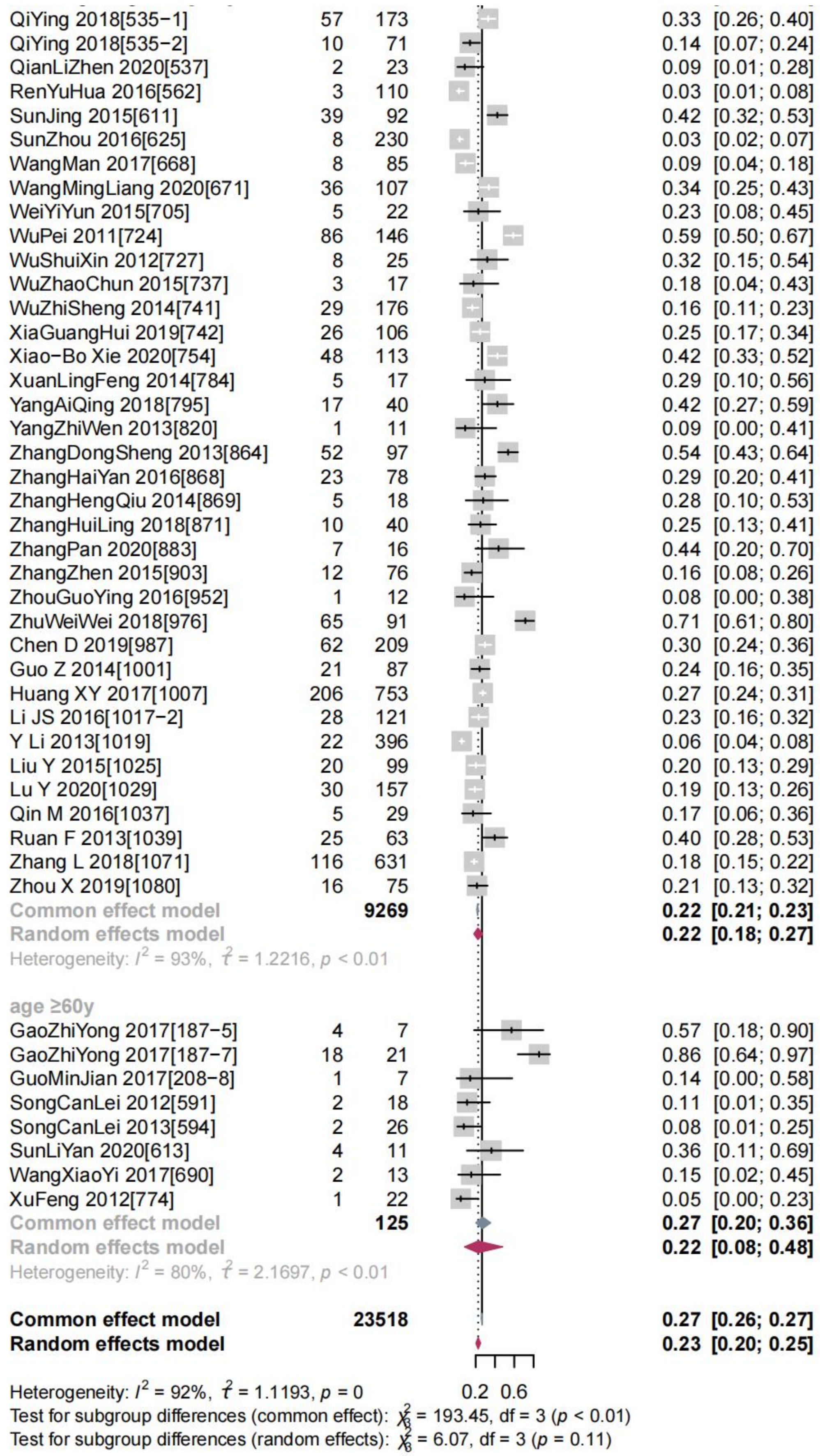
QiYing 2018[535−1] 57 173 0.33 [0.26; 0.40]
QiYing 2018[535−2] 10 71 0.14 [0.07; 0.24]
QianLiZhen 2020[537] 2 23 0.09 [0.01; 0.28]
RenYuHua 2016[562] 3 110 0.03 [0.01; 0.08]
SunJing 2015[611] 39 92 0.42 [0.32; 0.53]
SunZhou 2016[625] 8 230 0.03 [0.02; 0.07]
WangMan 2017[668] 8 85 0.09 [0.04; 0.18]
WangMingLiang 2020[671] 36 107 0.34 [0.25; 0.43]
WeiYiYun 2015[705] 5 22 0.23 [0.08; 0.45]
WuPei 2011[724] 86 146 0.59 [0.50; 0.67]
WuShuiXin 2012[727] 8 25 0.32 [0.15; 0.54]
WuZhaoChun 2015[737] 3 17 0.18 [0.04; 0.43]
WuZhiSheng 2014[741] 29 176 0.16 [0.11; 0.23]
XiaGuangHui 2019[742] 26 106 0.25 [0.17; 0.34]
Xiao−Bo Xie 2020[754] 48 113 0.42 [0.33; 0.52]
XuanLingFeng 2014[784] 5 17 0.29 [0.10; 0.56]
YangAiQing 2018[795] 17 40 0.42 [0.27; 0.59]
YangZhiWen 2013[820] 1 11 0.09 [0.00; 0.41]
ZhangDongSheng 2013[864] 52 97 0.54 [0.43; 0.64]
ZhangHaiYan 2016[868] 23 78 0.29 [0.20; 0.41]
ZhangHengQiu 2014[869] 5 18 0.28 [0.10; 0.53]
ZhangHuiLing 2018[871] 10 40 0.25 [0.13; 0.41]
ZhangPan 2020[883] 7 16 0.44 [0.20; 0.70]
ZhangZhen 2015[903] 12 76 0.16 [0.08; 0.26]
ZhouGuoYing 2016[952] 1 12 0.08 [0.00; 0.38]
ZhuWeiWei 2018[976] 65 91 0.71 [0.61; 0.80]
Chen D 2019[987] 62 209 0.30 [0.24; 0.36]
Guo Z 2014[1001] 21 87 0.24 [0.16; 0.35]
Huang XY 2017[1007] 206 753 0.27 [0.24; 0.31]
Li JS 2016[1017−2] 28 121 0.23 [0.16; 0.32]
Y Li 2013[1019] 22 396 0.06 [0.04; 0.08]
Liu Y 2015[1025] 20 99 0.20 [0.13; 0.29]
Lu Y 2020[1029] 30 157 0.19 [0.13; 0.26]
Qin M 2016[1037] 5 29 0.17 [0.06; 0.36]
Ruan F 2013[1039] 25 63 0.40 [0.28; 0.53]
Zhang L 2018[1071] 116 631 0.18 [0.15; 0.22]
Zhou X 2019[1080] 16 75 0.21 [0.13; 0.32]
Common effect model 9269 0.22 [0.21; 0.23]
Random effects model 0.22 [0.18; 0.27]
Heterogeneity: I^2 = 93%, τ^2 = 1.2216, p < 0.01
age ≥60y
GaoZhiYong 2017[187−5] 4 7 0.57 [0.18; 0.90]
GaoZhiYong 2017[187−7] 18 21 0.86 [0.64; 0.97]
GuoMinJian 2017[208−8] 1 7 0.14 [0.00; 0.58]
SongCanLei 2012[591] 2 18 0.11 [0.01; 0.35]
SongCanLei 2013[594] 2 26 0.08 [0.01; 0.25]
SunLiYan 2020[613] 4 11 0.36 [0.11; 0.69]
WangXiaoYi 2017[690] 2 13 0.15 [0.02; 0.45]
XuFeng 2012[774] 1 22 0.05 [0.00; 0.23]
Common effect model 125 0.27 [0.20; 0.36]
Random effects model 0.22 [0.08; 0.48]
Heterogeneity: I^2 = 80%, τ^2 = 2.1697, p < 0.01
Common effect model 23518 0.27 [0.26; 0.27]
Random effects model 0.23 [0.20; 0.25]
0.2 0.6
Heterogeneity: I^2 = 92%, τ^2 = 1.1193, p = 0
Test for subgroup differences (common effect): χ^2_3 = 193.45, df = 3 (p < 0.01)
Test for subgroup differences (random effects): χ^2_3 = 6.07, df = 3 (p = 0.11)

(e7)

Study Headache	Events	Total	Proportion	95%−CI	Weight (common)	Weight (random)
GII = GII.2						
GaoJunYing 2017[177]	1	12	0.08	[0.00; 0.38]	0.6%	6.6%
XiaoDaYong 2018[746]	3	47	0.06	[0.01; 0.18]	2.2%	7.7%
**Common effect model**		**59**	**0.06**	**[0.01; 0.14]**	**2.8%**	**--**
**Random effects model**			**0.06**	**[0.01; 0.14]**	**--**	**14.3%**
Heterogeneity: $I^2 = 0\%$, $\tau^2 = 0$, $p = 0.66$						
GII = GII.4						
JiHong 2015[264]	41	78	0.53	[0.41; 0.64]	3.7%	7.9%
SongCanLei 2012[591]	1	18	0.06	[0.00; 0.27]	0.9%	7.1%
SunJing 2015[611]	20	92	0.22	[0.14; 0.32]	4.3%	8.0%
ZhangZhen 2015[903]	22	76	0.29	[0.19; 0.40]	3.6%	7.9%
Guo Z 2014[1001]	2	87	0.02	[0.00; 0.08]	4.1%	8.0%
**Common effect model**		**351**	**0.21**	**[0.17; 0.26]**	**16.5%**	**--**
**Random effects model**			**0.20**	**[0.05; 0.40]**	**--**	**38.9%**
Heterogeneity: $I^2 = 95\%$, $\tau^2 = 0.0597$, $p < 0.01$						
GII = GII.12						
LiJun 2012[334]	704	1252	0.56	[0.53; 0.59]	58.4%	8.2%
GII = GII.17						
JiangChen 2019[285]	11	26	0.42	[0.23; 0.63]	1.2%	7.4%
Zhang XF 2015[1077]	17	276	0.06	[0.04; 0.10]	12.9%	8.2%
Zhou X 2019[1080]	4	75	0.05	[0.01; 0.13]	3.5%	7.9%
**Common effect model**		**377**	**0.07**	**[0.05; 0.10]**	**17.6%**	**--**
**Random effects model**			**0.14**	**[0.00; 0.40]**	**--**	**23.5%**
Heterogeneity: $I^2 = 90\%$, $\tau^2 = 0.0613$, $p < 0.01$						
GII = Recombination GII						
LiuDongSheng 2019[404]	60	80	0.75	[0.64; 0.84]	3.8%	7.9%
GII = Other						
HuGuangYi 2017[232]	1	20	0.05	[0.00; 0.25]	1.0%	7.2%
**Common effect model**		**2139**	**0.39**	**[0.37; 0.41]**	**100.0%**	**--**
**Random effects model**			**0.21**	**[0.10; 0.36]**	**--**	**100.0%**

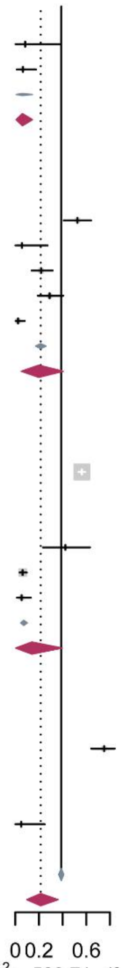

Heterogeneity: $I^2 = 98\%$, $\tau^2 = 0.0789$, $p < 0.01$
Test for subgroup differences (common effect): $\chi^2_5 = 523.71$, df = 5 ($p < 0.01$)
Test for subgroup differences (random effects): $\chi^2_5 = 131.08$, df = 5 ($p < 0.01$)

(e8)

Study Dizziness	Events	Total	Proportion	95%−CI	Weight (common)	Weight (random)
**age = 0−4y**						
ChenJingFang 2018[84−1]	1	18	0.06	[0.00; 0.27]	0.4%	1.6%
ChenJingFang 2018[84−2]	2	14	0.14	[0.02; 0.43]	0.3%	1.5%
**Common effect model**		**32**	**0.09**	**[0.02; 0.21]**	**0.7%**	**--**
**Random effects model**			**0.09**	**[0.02; 0.21]**	**--**	**3.1%**
Heterogeneity: I^2 = 0%, τ^2 = 0, p = 0.40						
**age = 5−17y**						
ShenYuGang 2016[54]	36	43	0.84	[0.69; 0.93]	1.0%	1.7%
ChenJian 2016[78]	3	20	0.15	[0.03; 0.38]	0.4%	1.6%
ChenXiaoFeng 2015[100]	7	7	1.00	[0.59; 1.00]	0.2%	1.3%
ChenYiXiong 2018[108]	10	21	0.48	[0.26; 0.70]	0.5%	1.6%
FuXiaoFei 2012[171]	18	20	0.90	[0.68; 0.99]	0.4%	1.6%
GanXiangYang 2014[172]	41	74	0.55	[0.43; 0.67]	1.7%	1.8%
GaoJunYing 2017[177]	1	12	0.08	[0.00; 0.38]	0.3%	1.5%
HuangYanHong 2019[260]	1	84	0.01	[0.00; 0.06]	1.9%	1.8%
LiShiCong 2020[344]	104	181	0.57	[0.50; 0.65]	4.1%	1.8%
LiShouJun 2016[350]	8	64	0.12	[0.06; 0.23]	1.4%	1.8%
LiYueRong 2015[366]	7	18	0.39	[0.17; 0.64]	0.4%	1.6%
LinQiFeng 2018[388]	4	96	0.04	[0.01; 0.10]	2.2%	1.8%
LiuBo 2015[398]	47	104	0.45	[0.35; 0.55]	2.3%	1.8%
LiuDongSheng 2019[404]	60	80	0.75	[0.64; 0.84]	1.8%	1.8%
LiuYing 2019[437]	3	14	0.21	[0.05; 0.51]	0.3%	1.5%
NiChaoRong 2019[510]	75	125	0.60	[0.51; 0.69]	2.8%	1.8%
OuSheXiang 2019[515]	1	18	0.06	[0.00; 0.27]	0.4%	1.6%
QiuHaiYan 2013[551]	48	76	0.63	[0.51; 0.74]	1.7%	1.8%
SongHuiRong 2017[597]	12	34	0.35	[0.20; 0.54]	0.8%	1.7%
TangYuHuan 2017[630]	19	44	0.43	[0.28; 0.59]	1.0%	1.7%
WuWenQian 2018[729]	14	36	0.39	[0.23; 0.57]	0.8%	1.7%
WuYiLing 2017[736]	6	31	0.19	[0.07; 0.37]	0.7%	1.7%
XuJianRong 2018[767]	44	92	0.48	[0.37; 0.58]	2.1%	1.8%
YangCaiBin 2018[796]	23	42	0.55	[0.39; 0.70]	0.9%	1.7%
ZhanYueWang 2019[860]	8	38	0.21	[0.10; 0.37]	0.9%	1.7%
ZhaoMengJiao 2018[919]	46	87	0.53	[0.42; 0.64]	2.0%	1.8%
ZhenGuoXin 2020[936]	11	23	0.48	[0.27; 0.69]	0.5%	1.6%
ZouYong 2011[1103]	5	39	0.13	[0.04; 0.27]	0.9%	1.7%
**Common effect model**		**1523**	**0.41**	**[0.39; 0.44]**	**34.3%**	**--**
**Random effects model**			**0.40**	**[0.29; 0.52]**	**--**	**47.5%**
Heterogeneity: I^2 = 95%, τ^2 = 0.0931, p < 0.01						

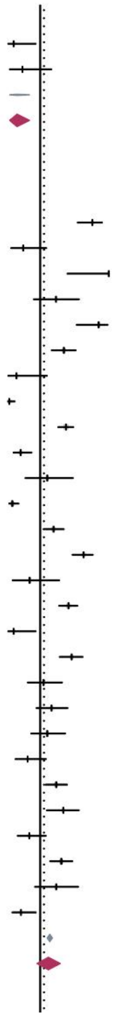

**age = 18−60y**						
ShenYuGang 2016[55]	153	233	0.66	[0.59; 0.72]	5.2%	1.8%
ChenGuoCui 2011[69]	9	27	0.33	[0.17; 0.54]	0.6%	1.7%
ChenHeJuan 2018[73]	1	14	0.07	[0.00; 0.34]	0.3%	1.5%
ChenJianMei 2017[79]	43	110	0.39	[0.30; 0.49]	2.5%	1.8%
FengZhi 2020[161]	5	26	0.19	[0.07; 0.39]	0.6%	1.7%
GaoRiHong 2019[182]	2	30	0.07	[0.01; 0.22]	0.7%	1.7%
HuangGuo 2015[253]	58	87	0.67	[0.56; 0.76]	2.0%	1.8%
JiLei 2020[270]	16	19	0.84	[0.60; 0.97]	0.4%	1.6%
JiangChen 2019[285]	11	26	0.42	[0.23; 0.63]	0.6%	1.7%
LiQun 2010[340]	40	258	0.16	[0.11; 0.21]	5.8%	1.8%
LiXiuFang 2018[359]	7	20	0.35	[0.15; 0.59]	0.4%	1.6%
LiuKaiQian 2011[415]	95	634	0.15	[0.12; 0.18]	14.3%	1.8%
Hao−Yu Shi 2019[582]	2	9	0.22	[0.03; 0.60]	0.2%	1.4%
WangHu 2018[656]	37	72	0.51	[0.39; 0.63]	1.6%	1.8%
WangMan 2017[668]	39	85	0.46	[0.35; 0.57]	1.9%	1.8%
XiaGuangHui 2019[742]	60	106	0.57	[0.47; 0.66]	2.4%	1.8%
XuanLingFeng 2014[784]	4	17	0.24	[0.07; 0.50]	0.4%	1.6%
YangAiQing 2018[795]	8	40	0.20	[0.09; 0.36]	0.9%	1.7%
ZhangHaiYan 2016[868]	19	78	0.24	[0.15; 0.35]	1.8%	1.8%
ZhangPan 2020[883]	5	16	0.31	[0.11; 0.59]	0.4%	1.6%
ZhangZhen 2015[903]	34	76	0.45	[0.33; 0.57]	1.7%	1.8%
ZhouGuoYing 2016[952]	12	12	1.00	[0.74; 1.00]	0.3%	1.5%
Chen D 2019[987]	76	209	0.36	[0.30; 0.43]	4.7%	1.8%
Guo Z 2014[1001]	4	87	0.05	[0.01; 0.11]	2.0%	1.8%
Y Li 2013[1019]	50	396	0.13	[0.10; 0.16]	8.9%	1.8%
Liu Y 2015[1025]	45	99	0.45	[0.35; 0.56]	2.2%	1.8%
Zhou X 2019[1080]	5	75	0.07	[0.02; 0.15]	1.7%	1.8%
**Common effect model**		**2861**	**0.28**	**[0.26; 0.29]**	**64.4%**	**--**
**Random effects model**			**0.34**	**[0.24; 0.45]**	**--**	**46.3%**
Heterogeneity: $I^2 = 96\%$, $\tau^2 = 0.0749$, $p < 0.01$						
**age ≥60y**						
SongCanLei 2012[591]	1	18	0.06	[0.00; 0.27]	0.4%	1.6%
SunLiYan 2020[613]	5	11	0.45	[0.17; 0.77]	0.2%	1.5%
**Common effect model**		**29**	**0.17**	**[0.06; 0.33]**	**0.7%**	**--**
**Random effects model**			**0.21**	**[0.00; 0.68]**	**--**	**3.1%**
Heterogeneity: $I^2 = 85\%$, $\tau^2 = 0.1077$, $p < 0.01$						
**Common effect model**		**4445**	**0.32**	**[0.31; 0.33]**	**100.0%**	**--**
**Random effects model**			**0.36**	**[0.28; 0.43]**	**--**	**100.0%**

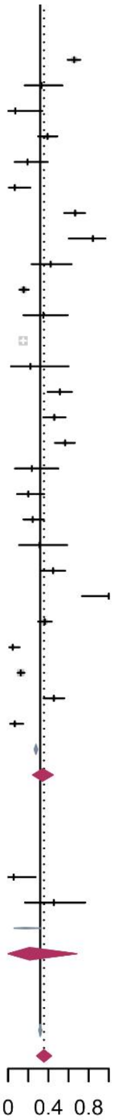

Heterogeneity: $I^2 = 95\%$, $\tau^2 = 0.0836$, $p < 0.01$
Test for subgroup differences (common effect): $\chi^2_3 = 98.76$, df = 3 ($p < 0.01$)
Test for subgroup differences (random effects): $\chi^2_3 = 13.60$, df = 3 ($p < 0.01$)

(e9)

Study Vomiting	Events	Total	Proportion	95%−CI	Weight (common)	Weight (random)
GII = GII.2						
ShenYiPing 2018[52]	18	26	0.69	[0.48; 0.86]	0.4%	1.3%
GaoJunYing 2017[177]	12	12	1.00	[0.74; 1.00]	0.2%	1.2%
HuangYanHong 2019[260]	65	84	0.77	[0.67; 0.86]	1.2%	1.4%
WangJun 2021[664]	20	20	1.00	[0.83; 1.00]	0.3%	1.3%
WangXiaoYi 2017[691]	2	13	0.15	[0.02; 0.45]	0.2%	1.2%
WuXiaoMin 2021[732]	151	159	0.95	[0.90; 0.98]	2.2%	1.5%
XiaoDaYong 2018[746]	43	47	0.91	[0.80; 0.98]	0.7%	1.4%
XieCaiWen 2017[749]	10	12	0.83	[0.52; 0.98]	0.2%	1.2%
Zhang TL 2017[1075]	18	20	0.90	[0.68; 0.99]	0.3%	1.3%
**Common effect model**		**393**	**0.89**	**[0.86; 0.93]**	**5.5%**	**--**
**Random effects model**			**0.85**	**[0.68; 0.97]**	**--**	**11.7%**
Heterogeneity: $I^2 = 87\%$, $\tau^2 = 0.0758$, $p < 0.01$						
GII = GII.3						
DaiBenNa 2020[127]	115	117	0.98	[0.94; 1.00]	1.6%	1.5%
YangRongXing 2019[808]	14	15	0.93	[0.68; 1.00]	0.2%	1.2%
Y Li 2021[1018]	133	143	0.93	[0.88; 0.97]	2.0%	1.5%
**Common effect model**		**275**	**0.96**	**[0.93; 0.99]**	**3.9%**	**--**
**Random effects model**			**0.96**	**[0.91; 1.00]**	**--**	**4.1%**
Heterogeneity: $I^2 = 58\%$, $\tau^2 = 0.0045$, $p = 0.09$						
GII = GII.4						
Cai MW 2018[18]	22	34	0.65	[0.46; 0.80]	0.5%	1.4%
CaiWenFeng 2013[25]	80	141	0.57	[0.48; 0.65]	2.0%	1.5%
CaiWenFeng 2014[26]	27	27	1.00	[0.87; 1.00]	0.4%	1.3%
ZengFengMei 2020[41]	24	25	0.96	[0.80; 1.00]	0.4%	1.3%
ChenAQun 2016[59]	28	64	0.44	[0.31; 0.57]	0.9%	1.4%
ChenJian 2016[78]	6	20	0.30	[0.12; 0.54]	0.3%	1.3%
JiHong 2015[264]	62	78	0.79	[0.69; 0.88]	1.1%	1.4%
LiQun 2010[340]	50	258	0.19	[0.15; 0.25]	3.6%	1.5%
LiuXiaoXiao 2014[430]	11	18	0.61	[0.36; 0.83]	0.3%	1.3%
SongCanLei 2012[591]	11	18	0.61	[0.36; 0.83]	0.3%	1.3%
SunJing 2015[611]	55	92	0.60	[0.49; 0.70]	1.3%	1.4%
SunLiMei 2012[612]	78	108	0.72	[0.63; 0.80]	1.5%	1.5%
WuPei 2011[724]	146	146	1.00	[0.98; 1.00]	2.0%	1.5%
WuZhiSheng 2014[741]	33	176	0.19	[0.13; 0.25]	2.5%	1.5%
YeXiaoLing 2014[833]	16	27	0.59	[0.39; 0.78]	0.4%	1.3%
ZhangZhen 2015[903]	36	76	0.47	[0.36; 0.59]	1.1%	1.4%
ZhouXiaoHong 2010[960]	20	37	0.54	[0.37; 0.71]	0.5%	1.4%
Guo Z 2014[1001]	62	87	0.71	[0.61; 0.80]	1.2%	1.4%
He Y 2016[1005]	17	28	0.61	[0.41; 0.78]	0.4%	1.3%
Zheng QM 2015[1078]	17	37	0.46	[0.29; 0.63]	0.5%	1.4%
Zhou X 2012[1081]	78	108	0.72	[0.63; 0.80]	1.5%	1.5%
**Common effect model**		**1605**	**0.57**	**[0.55; 0.60]**	**22.5%**	**--**
**Random effects model**			**0.63**	**[0.51; 0.75]**	**--**	**29.1%**
Heterogeneity: $I^2 = 97\%$, $\tau^2 = 0.0746$, $p < 0.01$						
GII = GII.12						
FuJianGuang 2013[163]	265	276	0.96	[0.93; 0.98]	3.9%	1.5%
LiJun 2012[334]	637	1252	0.51	[0.48; 0.54]	17.5%	1.5%
**Common effect model**		**1528**	**0.61**	**[0.59; 0.64]**	**21.3%**	**--**
**Random effects model**			**0.78**	**[0.25; 1.00]**	**--**	**3.0%**
Heterogeneity: $I^2 = 100\%$, $\tau^2 = 0.1627$, $p < 0.01$						

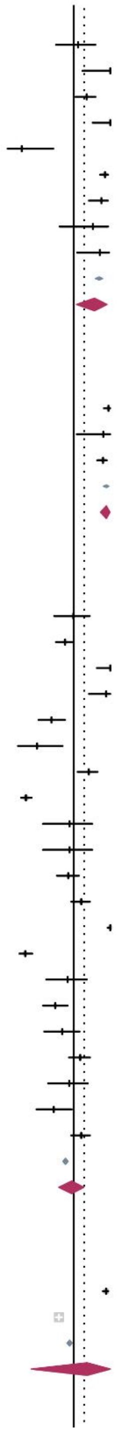

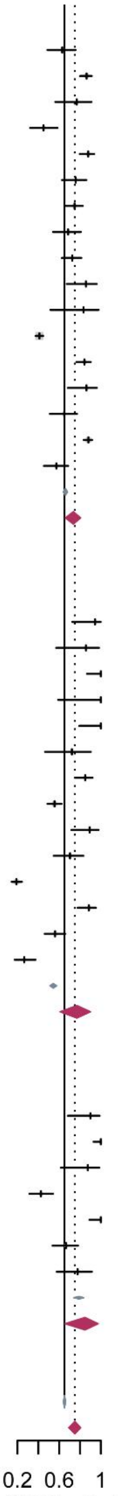

**GII = GII.17**						
ChenBinBin 2017[60−1]	34	54	0.63	[0.49; 0.76]	0.8%	1.4%
ChenBinBin 2017[60−2]	139	161	0.86	[0.80; 0.91]	2.3%	1.5%
JiangChen 2019[285]	20	26	0.77	[0.56; 0.91]	0.4%	1.3%
LuJianYong 2015[456]	27	60	0.45	[0.32; 0.58]	0.8%	1.4%
WangJinSheng 2016[646]	87	99	0.88	[0.80; 0.94]	1.4%	1.4%
WangBing 2017[649]	44	58	0.76	[0.63; 0.86]	0.8%	1.4%
WangMingLiang 2020[671]	80	107	0.75	[0.65; 0.83]	1.5%	1.5%
XiaoSongJian 2017[745]	35	51	0.69	[0.54; 0.81]	0.7%	1.4%
XieBin 2018[757]	69	95	0.73	[0.63; 0.81]	1.3%	1.4%
ZhangShuang 2017[888]	24	28	0.86	[0.67; 0.96]	0.4%	1.3%
ZhouYin 2016[964]	10	12	0.83	[0.52; 0.98]	0.2%	1.2%
Huang XY 2017[1007]	309	753	0.41	[0.37; 0.45]	10.5%	1.5%
Li JS 2016[1017−2]	102	121	0.84	[0.77; 0.90]	1.7%	1.5%
Qin M 2016[1037]	25	29	0.86	[0.68; 0.96]	0.4%	1.3%
Shi C 2016[1046]	37	57	0.65	[0.51; 0.77]	0.8%	1.4%
Zhang XF 2015[1077]	243	276	0.88	[0.84; 0.92]	3.9%	1.5%
Zhou X 2019[1080]	43	75	0.57	[0.45; 0.69]	1.1%	1.4%
**Common effect model**		**2062**	**0.66**	**[0.64; 0.68]**	**28.9%**	**--**
**Random effects model**			**0.74**	**[0.66; 0.81]**	**--**	**23.9%**
Heterogeneity: $I^2 = 96\%$, $\tau^2 = 0.0246$, $p < 0.01$						
**GII = Recombination GII**						
ChenJingFang 2018[84−1]	17	18	0.94	[0.73; 1.00]	0.3%	1.3%
ChenJingFang 2018[84−2]	12	14	0.86	[0.57; 0.98]	0.2%	1.2%
Lei Ji 2021[269]	26	26	1.00	[0.87; 1.00]	0.4%	1.3%
JiLei 2018[272−1]	7	7	1.00	[0.59; 1.00]	0.1%	1.0%
JiLei 2018[272−2]	16	16	1.00	[0.79; 1.00]	0.2%	1.2%
JiLei 2018[272−3]	13	18	0.72	[0.47; 0.90]	0.3%	1.3%
LiuDongSheng 2019[404]	68	80	0.85	[0.75; 0.92]	1.1%	1.4%
MaMengMeng 2018[484]	124	223	0.56	[0.49; 0.62]	3.1%	1.5%
NiChaoRong 2019[511]	25	28	0.89	[0.72; 0.98]	0.4%	1.3%
SongCanLei 2020[590]	31	44	0.70	[0.55; 0.83]	0.6%	1.4%
ZhangZhiZhong 2020[906]	50	263	0.19	[0.14; 0.24]	3.7%	1.5%
Guo XH 2018[1000]	54	61	0.89	[0.78; 0.95]	0.9%	1.4%
Ji L 2019[1020−1]	60	107	0.56	[0.46; 0.66]	1.5%	1.5%
Ji L 2019[1020−2]	22	83	0.27	[0.17; 0.37]	1.2%	1.4%
**Common effect model**		**988**	**0.54**	**[0.51; 0.58]**	**13.9%**	**--**
**Random effects model**			**0.77**	**[0.61; 0.90]**	**--**	**18.7%**
Heterogeneity: $I^2 = 97\%$, $\tau^2 = 0.0943$, $p < 0.01$						
**GII = Other**						
HuGuangYi 2017[232]	18	20	0.90	[0.68; 0.99]	0.3%	1.3%
LiXiTai 2015[352]	51	51	1.00	[0.93; 1.00]	0.7%	1.4%
QinDi 2016[544]	14	16	0.88	[0.62; 0.98]	0.2%	1.2%
YuHong 2016[848]	34	80	0.42	[0.32; 0.54]	1.1%	1.4%
Luo LF 2015[1030]	32	32	1.00	[0.89; 1.00]	0.5%	1.4%
Ruan F 2013[1039]	42	63	0.67	[0.54; 0.78]	0.9%	1.4%
Zhang XF 2020[1076]	21	27	0.78	[0.58; 0.91]	0.4%	1.3%
**Common effect model**		**289**	**0.79**	**[0.74; 0.84]**	**4.1%**	**--**
**Random effects model**			**0.85**	**[0.66; 0.97]**	**--**	**9.4%**
Heterogeneity: $I^2 = 94\%$, $\tau^2 = 0.0779$, $p < 0.01$						
**Common effect model**		**7140**	**0.65**	**[0.64; 0.66]**	**100.0%**	**--**
**Random effects model**			**0.75**	**[0.69; 0.81]**	**--**	**100.0%**

Heterogeneity: $I^2 = 97\%$, $\tau^2 = 0.0703$, $p = 0$

Test for subgroup differences (common effect): $\chi^2_6 = 435.51$, df = 6 ($p < 0.01$)

Test for subgroup differences (random effects): $\chi^2_6 = 34.72$, df = 6 ($p < 0.01$)

(e10)

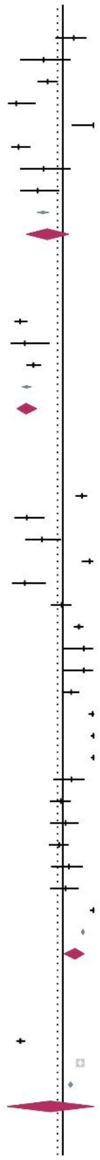

Study Diarrhea	Events	Total	Proportion	95%−CI	Weight (common)	Weight (random)
**GII = GII.2**						
ShenYiPing 2018[52]	20	26	0.77	[0.56; 0.91]	0.4%	1.4%
GaoJunYing 2017[177]	5	12	0.42	[0.15; 0.72]	0.2%	1.3%
HuangYanHong 2019[260]	39	84	0.46	[0.35; 0.58]	1.2%	1.5%
WangJun 2021[664]	2	20	0.10	[0.01; 0.32]	0.3%	1.4%
WangXiaoYi 2017[691]	13	13	1.00	[0.75; 1.00]	0.2%	1.3%
XiaoDaYong 2018[746]	6	47	0.13	[0.05; 0.26]	0.7%	1.5%
XieCaiWen 2017[749]	5	12	0.42	[0.15; 0.72]	0.2%	1.3%
Zhang TL 2017[1075]	7	20	0.35	[0.15; 0.59]	0.3%	1.4%
**Common effect model**		**234**	**0.41**	**[0.35; 0.48]**	**3.5%**	**--**
**Random effects model**			**0.46**	**[0.22; 0.71]**	**--**	**11.1%**
Heterogeneity: $I^2 = 91\%$, $\tau^2 = 0.1101$, $p < 0.01$						
**GII = GII.3**						
DaiBenNa 2020[127]	17	117	0.15	[0.09; 0.22]	1.7%	1.5%
YangRongXing 2019[808]	3	15	0.20	[0.04; 0.48]	0.2%	1.3%
Y Li 2021[1018]	43	143	0.30	[0.23; 0.38]	2.1%	1.5%
**Common effect model**		**275**	**0.22**	**[0.17; 0.27]**	**4.0%**	**--**
**Random effects model**			**0.21**	**[0.11; 0.34]**	**--**	**4.4%**
Heterogeneity: $I^2 = 78\%$, $\tau^2 = 0.0094$, $p = 0.01$						
**GII = GII.4**						
CaiWenFeng 2013[25]	122	141	0.87	[0.80; 0.92]	2.1%	1.5%
CaiWenFeng 2014[26]	6	27	0.22	[0.09; 0.42]	0.4%	1.4%
ZengFengMei 2020[41]	10	25	0.40	[0.21; 0.61]	0.4%	1.4%
ChenAQun 2016[59]	61	64	0.95	[0.87; 0.99]	0.9%	1.5%
ChenJian 2016[78]	4	20	0.20	[0.06; 0.44]	0.3%	1.4%
JiHong 2015[264]	49	78	0.63	[0.51; 0.74]	1.1%	1.5%
LiQun 2010[340]	214	258	0.83	[0.78; 0.87]	3.8%	1.5%
LiuXiaoXiao 2014[430]	16	18	0.89	[0.65; 0.99]	0.3%	1.4%
SongCanLei 2012[591]	16	18	0.89	[0.65; 0.99]	0.3%	1.4%
SunJing 2015[611]	68	92	0.74	[0.64; 0.83]	1.3%	1.5%
SunLiMei 2012[612]	107	108	0.99	[0.95; 1.00]	1.6%	1.5%
WuPei 2011[724]	146	146	1.00	[0.98; 1.00]	2.1%	1.5%
WuZhiSheng 2014[741]	176	176	1.00	[0.98; 1.00]	2.6%	1.5%
YeXiaoLing 2014[833]	20	27	0.74	[0.54; 0.89]	0.4%	1.4%
ZhangZhen 2015[903]	47	76	0.62	[0.50; 0.73]	1.1%	1.5%
ZhouXiaoHong 2010[960]	25	37	0.68	[0.50; 0.82]	0.5%	1.5%
Guo Z 2014[1001]	52	87	0.60	[0.49; 0.70]	1.3%	1.5%
He Y 2016[1005]	20	28	0.71	[0.51; 0.87]	0.4%	1.4%
Zheng QM 2015[1078]	25	37	0.68	[0.50; 0.82]	0.5%	1.5%
Zhou X 2012[1081]	108	108	1.00	[0.97; 1.00]	1.6%	1.5%
**Common effect model**		**1571**	**0.88**	**[0.86; 0.89]**	**23.0%**	**--**
**Random effects model**			**0.78**	**[0.66; 0.89]**	**--**	**29.3%**
Heterogeneity: $I^2 = 96\%$, $\tau^2 = 0.0905$, $p < 0.01$						
**GII = GII.12**						
FuJianGuang 2013[163]	41	276	0.15	[0.11; 0.20]	4.0%	1.5%
LiJun 2012[334]	1057	1252	0.84	[0.82; 0.86]	18.2%	1.5%
**Common effect model**		**1528**	**0.73**	**[0.71; 0.75]**	**22.2%**	**--**
**Random effects model**			**0.50**	**[0.00; 1.00]**	**--**	**3.1%**
Heterogeneity: $I^2 = 100\%$, $\tau^2 = 0.2938$, $p < 0.01$						

**GII = GII.17**					
ChenBinBin 2017[60−1]	28	54	0.52 [0.38; 0.66]	0.8%	1.5%
ChenBinBin 2017[60−2]	71	161	0.44 [0.36; 0.52]	2.3%	1.5%
JiangChen 2019[285]	24	26	0.92 [0.75; 0.99]	0.4%	1.4%
LuJianYong 2015[456]	60	60	1.00 [0.94; 1.00]	0.9%	1.5%
WangJinSheng 2016[646]	32	99	0.32 [0.23; 0.42]	1.4%	1.5%
WangBing 2017[649]	38	58	0.66 [0.52; 0.78]	0.8%	1.5%
WangMingLiang 2020[671]	65	107	0.61 [0.51; 0.70]	1.6%	1.5%
XiaoSongJian 2017[745]	20	51	0.39 [0.26; 0.54]	0.7%	1.5%
XieBin 2018[757]	36	95	0.38 [0.28; 0.48]	1.4%	1.5%
ZhangShuang 2017[888]	8	28	0.29 [0.13; 0.49]	0.4%	1.4%
ZhouYin 2016[964]	5	12	0.42 [0.15; 0.72]	0.2%	1.3%
Huang XY 2017[1007]	401	753	0.53 [0.50; 0.57]	10.9%	1.5%
Li JS 2016[1017−2]	23	121	0.19 [0.12; 0.27]	1.8%	1.5%
Qin M 2016[1037]	25	29	0.86 [0.68; 0.96]	0.4%	1.4%
Shi C 2016[1046]	46	57	0.81 [0.68; 0.90]	0.8%	1.5%
Zhang XF 2015[1077]	27	276	0.10 [0.07; 0.14]	4.0%	1.5%
Zhou X 2019[1080]	54	75	0.72 [0.60; 0.82]	1.1%	1.5%
**Common effect model**		**2062**	**0.47 [0.44; 0.49]**	**30.1%**	**--**
**Random effects model**			**0.55 [0.40; 0.69]**	**--**	**25.1%**
Heterogeneity: I^2 = 97%, τ^2 = 0.0885, p < 0.01					
**GII = Recombination GII**					
ChenJingFang 2018[84−1]	1	18	0.06 [0.00; 0.27]	0.3%	1.4%
ChenJingFang 2018[84−2]	7	14	0.50 [0.23; 0.77]	0.2%	1.3%
Lei Ji 2021[269]	6	26	0.23 [0.09; 0.44]	0.4%	1.4%
JiLei 2018[272−1]	1	7	0.14 [0.00; 0.58]	0.1%	1.2%
JiLei 2018[272−3]	18	18	1.00 [0.81; 1.00]	0.3%	1.4%
LiuDongSheng 2019[404]	57	80	0.71 [0.60; 0.81]	1.2%	1.5%
MaMengMeng 2018[484]	175	223	0.78 [0.72; 0.84]	3.2%	1.5%
NiChaoRong 2019[511]	16	28	0.57 [0.37; 0.76]	0.4%	1.4%
SongCanLei 2020[590]	16	44	0.36 [0.22; 0.52]	0.6%	1.5%
ZhangZhiZhong 2020[906]	129	263	0.49 [0.43; 0.55]	3.8%	1.5%
Guo XH 2018[1000]	15	61	0.25 [0.14; 0.37]	0.9%	1.5%
Ji L 2019[1020−1]	81	107	0.76 [0.66; 0.83]	1.6%	1.5%
Ji L 2019[1020−2]	74	83	0.89 [0.80; 0.95]	1.2%	1.5%
**Common effect model**		**972**	**0.63 [0.60; 0.66]**	**14.2%**	**--**
**Random effects model**			**0.54 [0.36; 0.72]**	**--**	**18.6%**
Heterogeneity: I^2 = 94%, τ^2 = 0.1008, p < 0.01					
**GII = Other**					
HuGuangYi 2017[232]	3	20	0.15 [0.03; 0.38]	0.3%	1.4%
LiXiTai 2015[352]	14	51	0.27 [0.16; 0.42]	0.7%	1.5%
QinDi 2016[544]	2	16	0.12 [0.02; 0.38]	0.2%	1.4%
Luo LF 2015[1030]	18	32	0.56 [0.38; 0.74]	0.5%	1.4%
Ruan F 2013[1039]	41	63	0.65 [0.52; 0.77]	0.9%	1.5%
Zhang XF 2020[1076]	12	27	0.44 [0.25; 0.65]	0.4%	1.4%
**Common effect model**		**209**	**0.42 [0.36; 0.49]**	**3.1%**	**--**
**Random effects model**			**0.37 [0.20; 0.55]**	**--**	**8.6%**
Heterogeneity: I^2 = 85%, τ^2 = 0.0420, p < 0.01					
**Common effect model**		**6851**	**0.64 [0.63; 0.65]**	**100.0%**	**--**
**Random effects model**			**0.58 [0.50; 0.66]**	**--**	**100.0%**

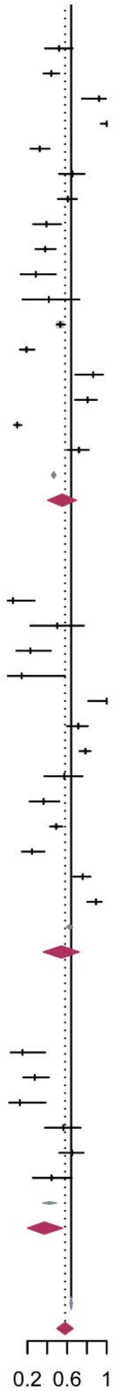

Heterogeneity: I^2 = 98%, τ^2 = 0.1090, p = 0
Test for subgroup differences (common effect): χ^2_6 = 1078.16, df = 6 (p < 0.01)
Test for subgroup differences (random effects): χ^2_6 = 39.64, df = 6 (p < 0.01)

(e11)

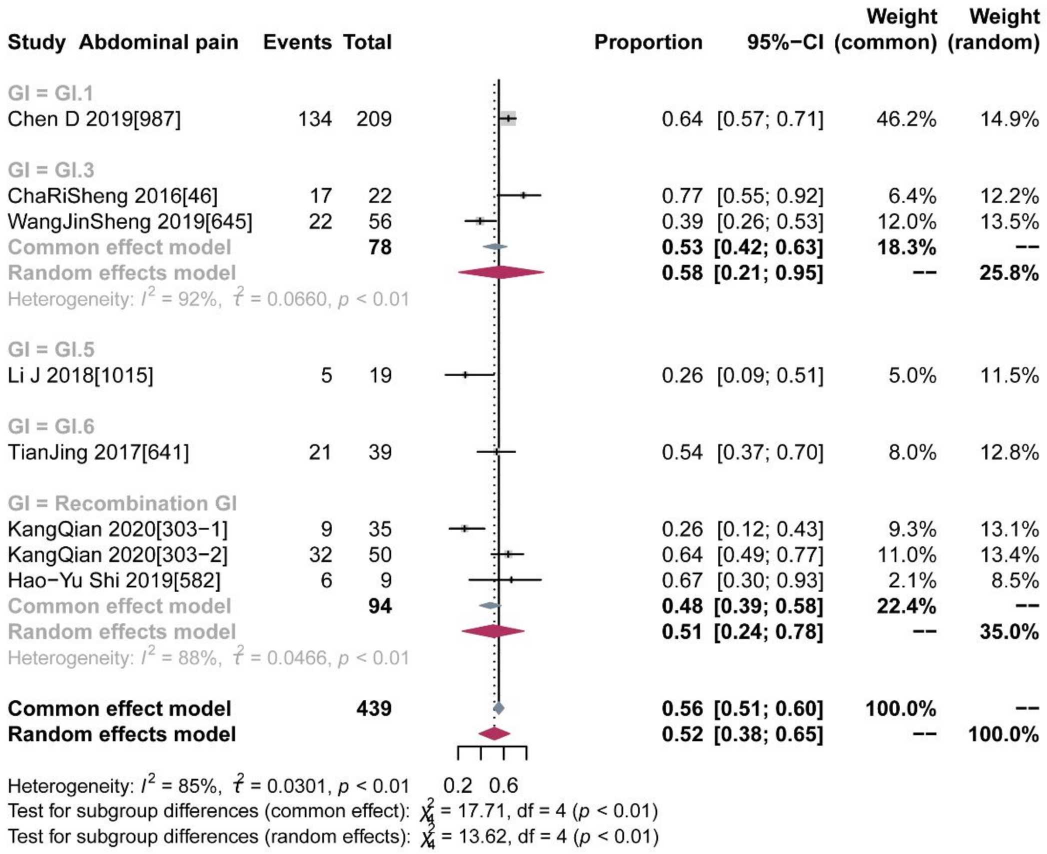
Study Abdominal pain Events Total Proportion 95%−CI Weight (common) Weight (random)
GI = GI.1
Chen D 2019[987] 134 209 0.64 [0.57; 0.71] 46.2% 14.9%
GI = GI.3
ChaRiSheng 2016[46] 17 22 0.77 [0.55; 0.92] 6.4% 12.2%
WangJinSheng 2019[645] 22 56 0.39 [0.26; 0.53] 12.0% 13.5%
Common effect model 78 0.53 [0.42; 0.63] 18.3% --
Random effects model 0.58 [0.21; 0.95] -- 25.8%
Heterogeneity: I^2 = 92%, τ^2 = 0.0660, p < 0.01
GI = GI.5
Li J 2018[1015] 5 19 0.26 [0.09; 0.51] 5.0% 11.5%
GI = GI.6
TianJing 2017[641] 21 39 0.54 [0.37; 0.70] 8.0% 12.8%
GI = Recombination GI
KangQian 2020[303−1] 9 35 0.26 [0.12; 0.43] 9.3% 13.1%
KangQian 2020[303−2] 32 50 0.64 [0.49; 0.77] 11.0% 13.4%
Hao−Yu Shi 2019[582] 6 9 0.67 [0.30; 0.93] 2.1% 8.5%
Common effect model 94 0.48 [0.39; 0.58] 22.4% --
Random effects model 0.51 [0.24; 0.78] -- 35.0%
Heterogeneity: I^2 = 88%, τ^2 = 0.0466, p < 0.01
Common effect model 439 0.56 [0.51; 0.60] 100.0% --
Random effects model 0.52 [0.38; 0.65] -- 100.0%
Heterogeneity: I^2 = 85%, τ^2 = 0.0301, p < 0.01
0.2 0.6
Test for subgroup differences (common effect): χ^2_4 = 17.71, df = 4 (p < 0.01)
Test for subgroup differences (random effects): χ^2_4 = 13.62, df = 4 (p < 0.01)

(e12)

Study Nausea	Events	Total	Proportion	95%−CI	Weight (common)	Weight (random)
GII = GII.2						
HuangYanHong 2019[260]	65	84	0.77	[0.67; 0.86]	0.6%	2.5%
WangJun 2021[664]	14	20	0.70	[0.46; 0.88]	0.1%	2.2%
WangXiaoYi 2017[691]	6	13	0.46	[0.19; 0.75]	0.1%	1.9%
WuXiaoMin 2021[732]	134	159	0.84	[0.78; 0.90]	1.5%	2.5%
XiaoDaYong 2018[746]	19	47	0.40	[0.26; 0.56]	0.2%	2.4%
Zhang TL 2017[1075]	15	20	0.75	[0.51; 0.91]	0.1%	2.2%
**Common effect model**		**343**	**0.77**	**[0.72; 0.81]**	**2.6%**	**--**
**Random effects model**			**0.67**	**[0.52; 0.82]**	**--**	**13.6%**
Heterogeneity: I^2 = 87%, τ^2 = 0.0267, p < 0.01						
GII = GII.3						
DaiBenNa 2020[127]	39	117	0.33	[0.25; 0.43]	0.7%	2.5%
GII = GII.4						
ChenJian 2016[78]	11	20	0.55	[0.32; 0.77]	0.1%	2.1%
JiHong 2015[264]	59	78	0.76	[0.65; 0.85]	0.5%	2.5%
LiQun 2010[340]	86	258	0.33	[0.28; 0.39]	1.4%	2.5%
LiuXiaoXiao 2014[430]	13	18	0.72	[0.47; 0.90]	0.1%	2.1%
SunJing 2015[611]	66	92	0.72	[0.61; 0.81]	0.6%	2.5%
SunLiMei 2012[612]	66	108	0.61	[0.51; 0.70]	0.6%	2.5%
WuPei 2011[724]	146	146	1.00	[0.98; 1.00]	53.7%	2.6%
YeXiaoLing 2014[833]	10	27	0.37	[0.19; 0.58]	0.1%	2.2%
ZhangZhen 2015[903]	46	76	0.61	[0.49; 0.72]	0.4%	2.4%
ZhouXiaoHong 2010[960]	21	37	0.57	[0.39; 0.73]	0.2%	2.3%
Guo Z 2014[1001]	57	87	0.66	[0.55; 0.75]	0.5%	2.5%
He Y 2016[1005]	10	28	0.36	[0.19; 0.56]	0.2%	2.2%
Zheng QM 2015[1078]	1	37	0.03	[0.00; 0.14]	1.7%	2.5%
Zhou X 2012[1081]	66	108	0.61	[0.51; 0.70]	0.6%	2.5%
**Common effect model**		**1120**	**0.93**	**[0.92; 0.94]**	**60.6%**	**--**
**Random effects model**			**0.56**	**[0.44; 0.69]**	**--**	**33.5%**
Heterogeneity: I^2 = 99%, τ^2 = 0.0555, p = 0						

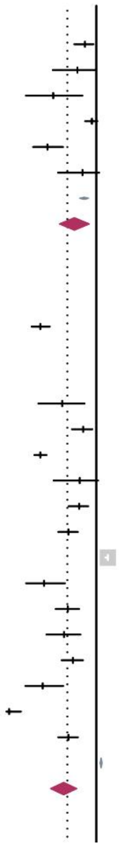

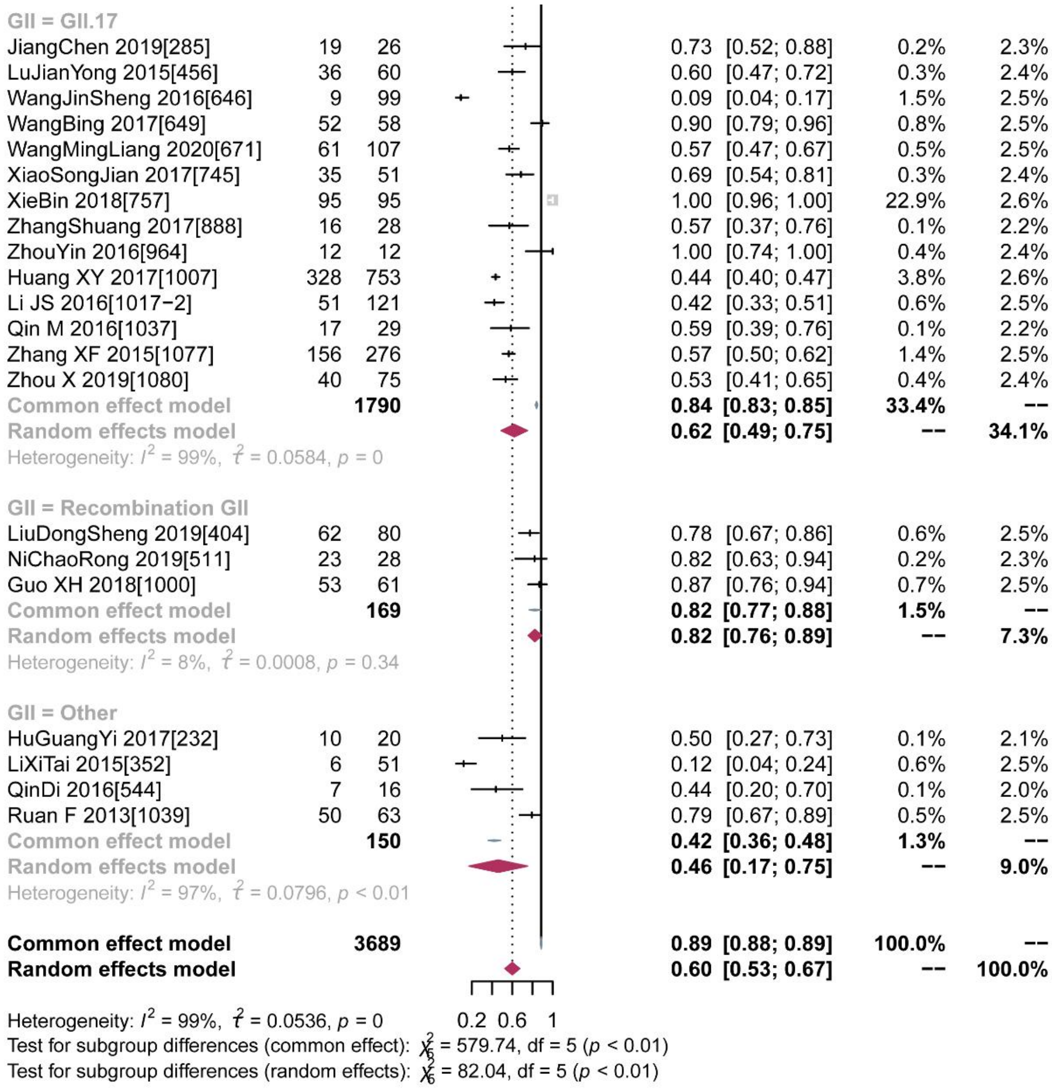
GII = GII.17
JiangChen 2019[285] 19 26 0.73 [0.52; 0.88] 0.2% 2.3%
LuJianYong 2015[456] 36 60 0.60 [0.47; 0.72] 0.3% 2.4%
WangJinSheng 2016[646] 9 99 0.09 [0.04; 0.17] 1.5% 2.5%
WangBing 2017[649] 52 58 0.90 [0.79; 0.96] 0.8% 2.5%
WangMingLiang 2020[671] 61 107 0.57 [0.47; 0.67] 0.5% 2.5%
XiaoSongJian 2017[745] 35 51 0.69 [0.54; 0.81] 0.3% 2.4%
XieBin 2018[757] 95 95 1.00 [0.96; 1.00] 22.9% 2.6%
ZhangShuang 2017[888] 16 28 0.57 [0.37; 0.76] 0.1% 2.2%
ZhouYin 2016[964] 12 12 1.00 [0.74; 1.00] 0.4% 2.4%
Huang XY 2017[1007] 328 753 0.44 [0.40; 0.47] 3.8% 2.6%
Li JS 2016[1017−2] 51 121 0.42 [0.33; 0.51] 0.6% 2.5%
Qin M 2016[1037] 17 29 0.59 [0.39; 0.76] 0.1% 2.2%
Zhang XF 2015[1077] 156 276 0.57 [0.50; 0.62] 1.4% 2.5%
Zhou X 2019[1080] 40 75 0.53 [0.41; 0.65] 0.4% 2.4%
Common effect model 1790 0.84 [0.83; 0.85] 33.4% --
Random effects model 0.62 [0.49; 0.75] -- 34.1%
Heterogeneity: I^2 = 99%, τ^2 = 0.0584, p = 0
GII = Recombination GII
LiuDongSheng 2019[404] 62 80 0.78 [0.67; 0.86] 0.6% 2.5%
NiChaoRong 2019[511] 23 28 0.82 [0.63; 0.94] 0.2% 2.3%
Guo XH 2018[1000] 53 61 0.87 [0.76; 0.94] 0.7% 2.5%
Common effect model 169 0.82 [0.77; 0.88] 1.5% --
Random effects model 0.82 [0.76; 0.89] -- 7.3%
Heterogeneity: I^2 = 8%, τ^2 = 0.0008, p = 0.34
GII = Other
HuGuangYi 2017[232] 10 20 0.50 [0.27; 0.73] 0.1% 2.1%
LiXiTai 2015[352] 6 51 0.12 [0.04; 0.24] 0.6% 2.5%
QinDi 2016[544] 7 16 0.44 [0.20; 0.70] 0.1% 2.0%
Ruan F 2013[1039] 50 63 0.79 [0.67; 0.89] 0.5% 2.5%
Common effect model 150 0.42 [0.36; 0.48] 1.3% --
Random effects model 0.46 [0.17; 0.75] -- 9.0%
Heterogeneity: I^2 = 97%, τ^2 = 0.0796, p < 0.01
Common effect model 3689 0.89 [0.88; 0.89] 100.0% --
Random effects model 0.60 [0.53; 0.67] -- 100.0%
0.2 0.6 1
Heterogeneity: I^2 = 99%, τ^2 = 0.0536, p = 0
Test for subgroup differences (common effect): χ^2_5 = 579.74, df = 5 (p < 0.01)
Test for subgroup differences (random effects): χ^2_5 = 82.04, df = 5 (p < 0.01)

(e13)

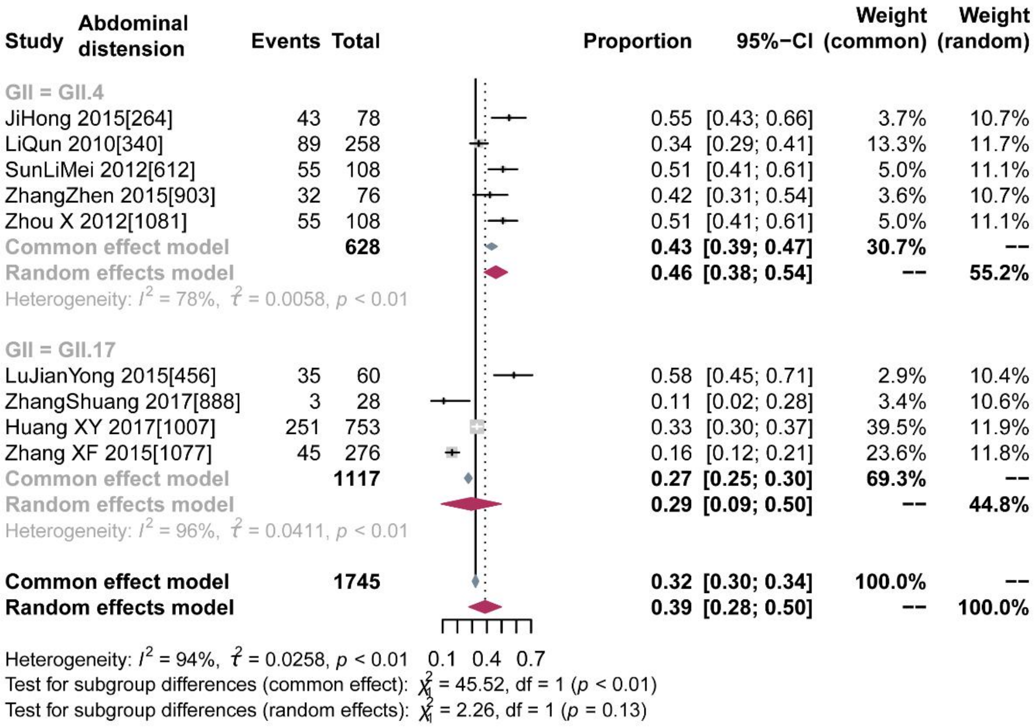

Study
Abdominal distension
Events
Total
Proportion
95%−CI
Weight (common)
Weight (random)
GII = GII.4
JiHong 2015[264] 43 78 0.55 [0.43; 0.66] 3.7% 10.7%
LiQun 2010[340] 89 258 0.34 [0.29; 0.41] 13.3% 11.7%
SunLiMei 2012[612] 55 108 0.51 [0.41; 0.61] 5.0% 11.1%
ZhangZhen 2015[903] 32 76 0.42 [0.31; 0.54] 3.6% 10.7%
Zhou X 2012[1081] 55 108 0.51 [0.41; 0.61] 5.0% 11.1%
Common effect model 628 0.43 [0.39; 0.47] 30.7% --
Random effects model 0.46 [0.38; 0.54] -- 55.2%
Heterogeneity: I^2 = 78%, τ^2 = 0.0058, p < 0.01
GII = GII.17
LuJianYong 2015[456] 35 60 0.58 [0.45; 0.71] 2.9% 10.4%
ZhangShuang 2017[888] 3 28 0.11 [0.02; 0.28] 3.4% 10.6%
Huang XY 2017[1007] 251 753 0.33 [0.30; 0.37] 39.5% 11.9%
Zhang XF 2015[1077] 45 276 0.16 [0.12; 0.21] 23.6% 11.8%
Common effect model 1117 0.27 [0.25; 0.30] 69.3% --
Random effects model 0.29 [0.09; 0.50] -- 44.8%
Heterogeneity: I^2 = 96%, τ^2 = 0.0411, p < 0.01
Common effect model 1745 0.32 [0.30; 0.34] 100.0% --
Random effects model 0.39 [0.28; 0.50] -- 100.0%
Heterogeneity: I^2 = 94%, τ^2 = 0.0258, p < 0.01
0.1 0.4 0.7
Test for subgroup differences (common effect): χ^2_1 = 45.52, df = 1 (p < 0.01)
Test for subgroup differences (random effects): χ^2_1 = 2.26, df = 1 (p = 0.13)

(e14)

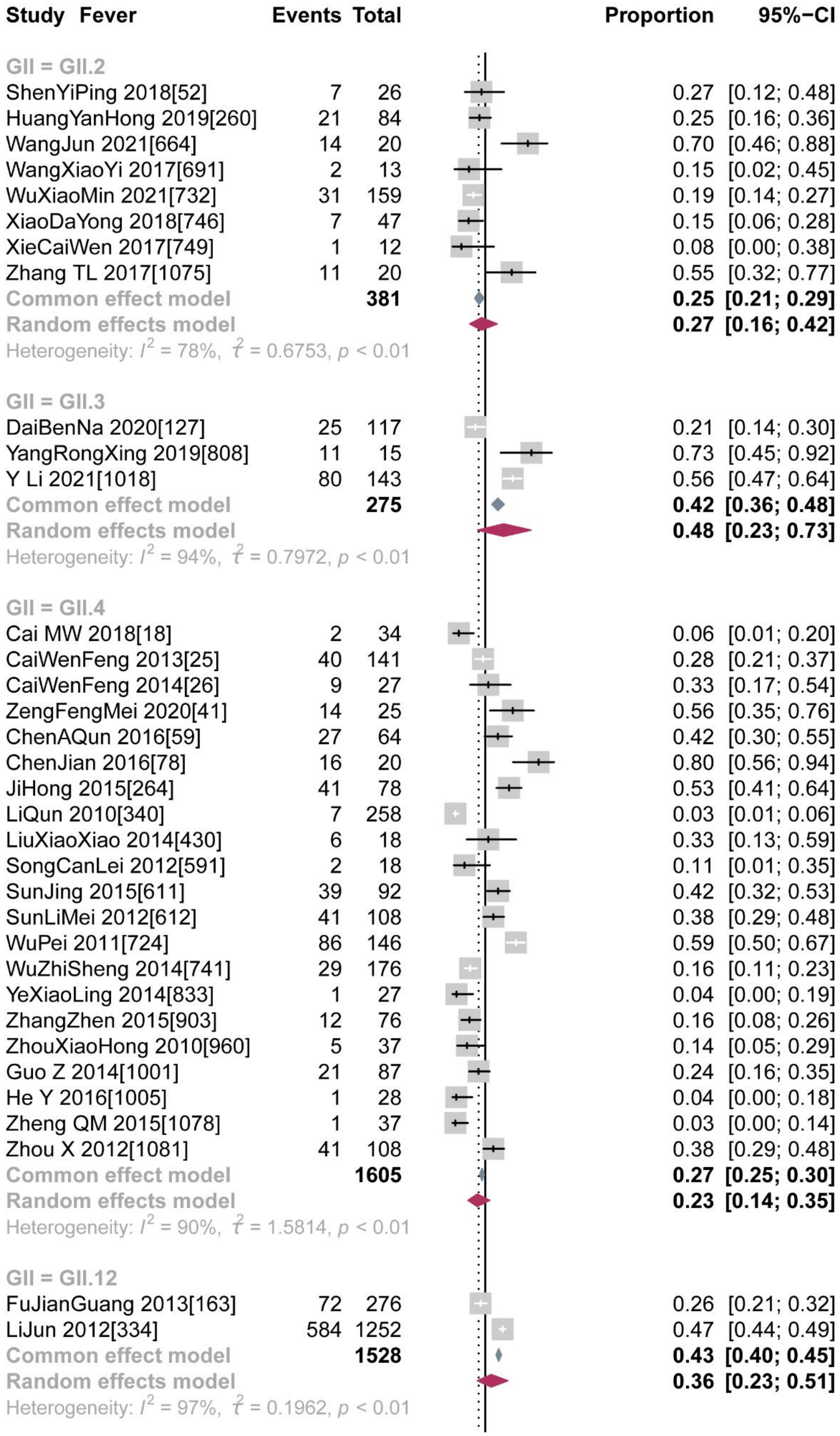
Study Fever Events Total Proportion 95%−CI
GII = GII.2
ShenYiPing 2018[52] 7 26 0.27 [0.12; 0.48]
HuangYanHong 2019[260] 21 84 0.25 [0.16; 0.36]
WangJun 2021[664] 14 20 0.70 [0.46; 0.88]
WangXiaoYi 2017[691] 2 13 0.15 [0.02; 0.45]
WuXiaoMin 2021[732] 31 159 0.19 [0.14; 0.27]
XiaoDaYong 2018[746] 7 47 0.15 [0.06; 0.28]
XieCaiWen 2017[749] 1 12 0.08 [0.00; 0.38]
Zhang TL 2017[1075] 11 20 0.55 [0.32; 0.77]
Common effect model 381 0.25 [0.21; 0.29]
Random effects model 0.27 [0.16; 0.42]
Heterogeneity: I^2 = 78%, τ^2 = 0.6753, p < 0.01
GII = GII.3
DaiBenNa 2020[127] 25 117 0.21 [0.14; 0.30]
YangRongXing 2019[808] 11 15 0.73 [0.45; 0.92]
Y Li 2021[1018] 80 143 0.56 [0.47; 0.64]
Common effect model 275 0.42 [0.36; 0.48]
Random effects model 0.48 [0.23; 0.73]
Heterogeneity: I^2 = 94%, τ^2 = 0.7972, p < 0.01
GII = GII.4
Cai MW 2018[18] 2 34 0.06 [0.01; 0.20]
CaiWenFeng 2013[25] 40 141 0.28 [0.21; 0.37]
CaiWenFeng 2014[26] 9 27 0.33 [0.17; 0.54]
ZengFengMei 2020[41] 14 25 0.56 [0.35; 0.76]
ChenAQun 2016[59] 27 64 0.42 [0.30; 0.55]
ChenJian 2016[78] 16 20 0.80 [0.56; 0.94]
JiHong 2015[264] 41 78 0.53 [0.41; 0.64]
LiQun 2010[340] 7 258 0.03 [0.01; 0.06]
LiuXiaoXiao 2014[430] 6 18 0.33 [0.13; 0.59]
SongCanLei 2012[591] 2 18 0.11 [0.01; 0.35]
SunJing 2015[611] 39 92 0.42 [0.32; 0.53]
SunLiMei 2012[612] 41 108 0.38 [0.29; 0.48]
WuPei 2011[724] 86 146 0.59 [0.50; 0.67]
WuZhiSheng 2014[741] 29 176 0.16 [0.11; 0.23]
YeXiaoLing 2014[833] 1 27 0.04 [0.00; 0.19]
ZhangZhen 2015[903] 12 76 0.16 [0.08; 0.26]
ZhouXiaoHong 2010[960] 5 37 0.14 [0.05; 0.29]
Guo Z 2014[1001] 21 87 0.24 [0.16; 0.35]
He Y 2016[1005] 1 28 0.04 [0.00; 0.18]
Zheng QM 2015[1078] 1 37 0.03 [0.00; 0.14]
Zhou X 2012[1081] 41 108 0.38 [0.29; 0.48]
Common effect model 1605 0.27 [0.25; 0.30]
Random effects model 0.23 [0.14; 0.35]
Heterogeneity: I^2 = 90%, τ^2 = 1.5814, p < 0.01
GII = GII.12
FuJianGuang 2013[163] 72 276 0.26 [0.21; 0.32]
LiJun 2012[334] 584 1252 0.47 [0.44; 0.49]
Common effect model 1528 0.43 [0.40; 0.45]
Random effects model 0.36 [0.23; 0.51]
Heterogeneity: I^2 = 97%, τ^2 = 0.1962, p < 0.01

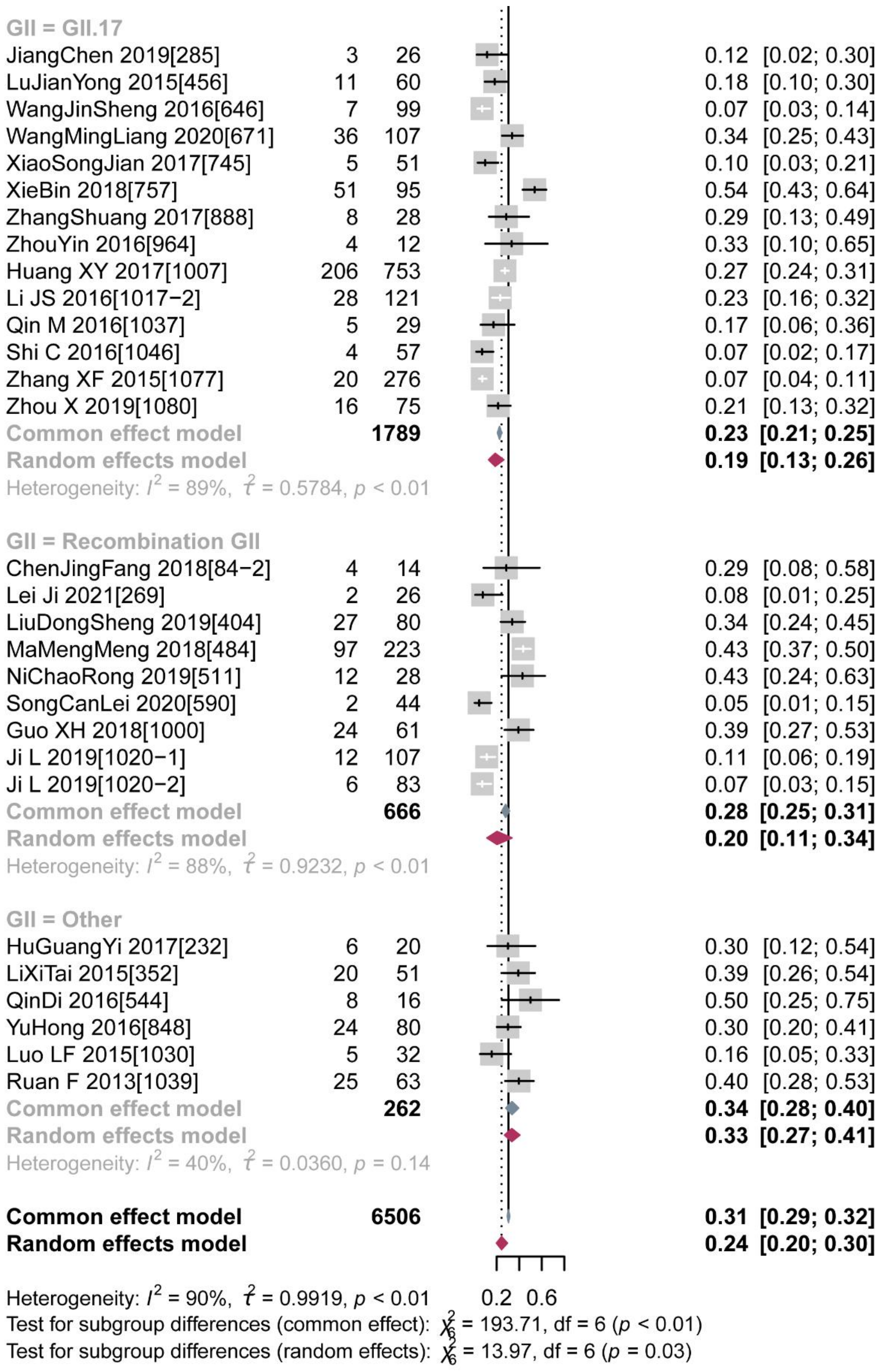
GII = GII.17
JiangChen 2019[285] 3 26 0.12 [0.02; 0.30]
LuJianYong 2015[456] 11 60 0.18 [0.10; 0.30]
WangJinSheng 2016[646] 7 99 0.07 [0.03; 0.14]
WangMingLiang 2020[671] 36 107 0.34 [0.25; 0.43]
XiaoSongJian 2017[745] 5 51 0.10 [0.03; 0.21]
XieBin 2018[757] 51 95 0.54 [0.43; 0.64]
ZhangShuang 2017[888] 8 28 0.29 [0.13; 0.49]
ZhouYin 2016[964] 4 12 0.33 [0.10; 0.65]
Huang XY 2017[1007] 206 753 0.27 [0.24; 0.31]
Li JS 2016[1017−2] 28 121 0.23 [0.16; 0.32]
Qin M 2016[1037] 5 29 0.17 [0.06; 0.36]
Shi C 2016[1046] 4 57 0.07 [0.02; 0.17]
Zhang XF 2015[1077] 20 276 0.07 [0.04; 0.11]
Zhou X 2019[1080] 16 75 0.21 [0.13; 0.32]
Common effect model 1789 0.23 [0.21; 0.25]
Random effects model 0.19 [0.13; 0.26]
Heterogeneity: I^2 = 89%, τ^2 = 0.5784, p < 0.01
GII = Recombination GII
ChenJingFang 2018[84−2] 4 14 0.29 [0.08; 0.58]
Lei Ji 2021[269] 2 26 0.08 [0.01; 0.25]
LiuDongSheng 2019[404] 27 80 0.34 [0.24; 0.45]
MaMengMeng 2018[484] 97 223 0.43 [0.37; 0.50]
NiChaoRong 2019[511] 12 28 0.43 [0.24; 0.63]
SongCanLei 2020[590] 2 44 0.05 [0.01; 0.15]
Guo XH 2018[1000] 24 61 0.39 [0.27; 0.53]
Ji L 2019[1020−1] 12 107 0.11 [0.06; 0.19]
Ji L 2019[1020−2] 6 83 0.07 [0.03; 0.15]
Common effect model 666 0.28 [0.25; 0.31]
Random effects model 0.20 [0.11; 0.34]
Heterogeneity: I^2 = 88%, τ^2 = 0.9232, p < 0.01
GII = Other
HuGuangYi 2017[232] 6 20 0.30 [0.12; 0.54]
LiXiTai 2015[352] 20 51 0.39 [0.26; 0.54]
QinDi 2016[544] 8 16 0.50 [0.25; 0.75]
YuHong 2016[848] 24 80 0.30 [0.20; 0.41]
Luo LF 2015[1030] 5 32 0.16 [0.05; 0.33]
Ruan F 2013[1039] 25 63 0.40 [0.28; 0.53]
Common effect model 262 0.34 [0.28; 0.40]
Random effects model 0.33 [0.27; 0.41]
Heterogeneity: I^2 = 40%, τ^2 = 0.0360, p = 0.14
Common effect model 6506 0.31 [0.29; 0.32]
Random effects model 0.24 [0.20; 0.30]
0.2 0.6
Heterogeneity: I^2 = 90%, τ^2 = 0.9919, p < 0.01
Test for subgroup differences (common effect): χ^2_6 = 193.71, df = 6 (p < 0.01)
Test for subgroup differences (random effects): χ^2_6 = 13.97, df = 6 (p = 0.03)

(e15)

Study Headache	Events	Total	Proportion	95%−CI	Weight (common)	Weight (random)
GII = GII.2						
GaoJunYing 2017[177]	1	12	0.08	[0.00; 0.38]	0.6%	6.6%
XiaoDaYong 2018[746]	3	47	0.06	[0.01; 0.18]	2.2%	7.7%
**Common effect model**		**59**	**0.06**	**[0.01; 0.14]**	**2.8%**	**--**
**Random effects model**			**0.06**	**[0.01; 0.14]**	**--**	**14.3%**
Heterogeneity: $I^2 = 0\%$, $\tau^2 = 0$, $p = 0.66$						
GII = GII.4						
JiHong 2015[264]	41	78	0.53	[0.41; 0.64]	3.7%	7.9%
SongCanLei 2012[591]	1	18	0.06	[0.00; 0.27]	0.9%	7.1%
SunJing 2015[611]	20	92	0.22	[0.14; 0.32]	4.3%	8.0%
ZhangZhen 2015[903]	22	76	0.29	[0.19; 0.40]	3.6%	7.9%
Guo Z 2014[1001]	2	87	0.02	[0.00; 0.08]	4.1%	8.0%
**Common effect model**		**351**	**0.21**	**[0.17; 0.26]**	**16.5%**	**--**
**Random effects model**			**0.20**	**[0.05; 0.40]**	**--**	**38.9%**
Heterogeneity: $I^2 = 95\%$, $\tau^2 = 0.0597$, $p < 0.01$						
GII = GII.12						
LiJun 2012[334]	704	1252	0.56	[0.53; 0.59]	58.4%	8.2%
GII = GII.17						
JiangChen 2019[285]	11	26	0.42	[0.23; 0.63]	1.2%	7.4%
Zhang XF 2015[1077]	17	276	0.06	[0.04; 0.10]	12.9%	8.2%
Zhou X 2019[1080]	4	75	0.05	[0.01; 0.13]	3.5%	7.9%
**Common effect model**		**377**	**0.07**	**[0.05; 0.10]**	**17.6%**	**--**
**Random effects model**			**0.14**	**[0.00; 0.40]**	**--**	**23.5%**
Heterogeneity: $I^2 = 90\%$, $\tau^2 = 0.0613$, $p < 0.01$						
GII = Recombination GII						
LiuDongSheng 2019[404]	60	80	0.75	[0.64; 0.84]	3.8%	7.9%
GII = Other						
HuGuangYi 2017[232]	1	20	0.05	[0.00; 0.25]	1.0%	7.2%
**Common effect model**		**2139**	**0.39**	**[0.37; 0.41]**	**100.0%**	**--**
**Random effects model**			**0.21**	**[0.10; 0.36]**	**--**	**100.0%**

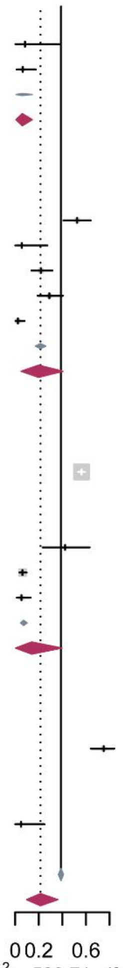

Heterogeneity: $I^2 = 98\%$, $\tau^2 = 0.0789$, $p < 0.01$
Test for subgroup differences (common effect): $\chi^2_6 = 523.71$, df = 5 ($p < 0.01$)
Test for subgroup differences (random effects): $\chi^2_6 = 131.08$, df = 5 ($p < 0.01$)

(e16)

Study Dizziness	Events	Total	Proportion	95%−CI	Weight (common)	Weight (random)
GII = GII.2						
GaoJunYing 2017[177]	1	12	0.08	[0.00; 0.38]	0.3%	4.4%
HuangYanHong 2019[260]	1	84	0.01	[0.00; 0.06]	0.2%	4.3%
Common effect model		**96**	**0.03**	**[0.01; 0.13]**	**0.5%**	**--**
Random effects model			**0.03**	**[0.00; 0.22]**	**--**	**8.7%**
Heterogeneity: $I^2 = 50\%$, $\tau^2 = 0.9409$, $p = 0.16$						
GII = GII.4						
ChenJian 2016[78]	3	20	0.15	[0.03; 0.38]	0.8%	7.0%
LiQun 2010[340]	40	258	0.16	[0.11; 0.21]	10.9%	9.2%
SongCanLei 2012[591]	1	18	0.06	[0.00; 0.27]	0.2%	4.4%
ZhangZhen 2015[903]	34	76	0.45	[0.33; 0.57]	14.1%	9.3%
ZhouXiaoHong 2010[960]	8	37	0.22	[0.10; 0.38]	2.3%	8.4%
Guo Z 2014[1001]	4	87	0.05	[0.01; 0.11]	1.0%	7.3%
Common effect model		**496**	**0.25**	**[0.21; 0.30]**	**29.4%**	**--**
Random effects model			**0.16**	**[0.08; 0.31]**	**--**	**45.6%**
Heterogeneity: $I^2 = 89\%$, $\tau^2 = 0.5343$, $p < 0.01$						
GII = GII.17						
JiangChen 2019[285]	11	26	0.42	[0.23; 0.63]	4.4%	8.9%
Zhang XF 2015[1077]	33	276	0.12	[0.08; 0.16]	8.6%	9.2%
Zhou X 2019[1080]	5	75	0.07	[0.02; 0.15]	1.2%	7.7%
Common effect model		**377**	**0.17**	**[0.13; 0.22]**	**14.2%**	**--**
Random effects model			**0.16**	**[0.05; 0.45]**	**--**	**25.7%**
Heterogeneity: $I^2 = 92\%$, $\tau^2 = 0.7836$, $p < 0.01$						
GII = Recombination GII						
ChenJingFang 2018[84−1]	1	18	0.06	[0.00; 0.27]	0.2%	4.4%
ChenJingFang 2018[84−2]	2	14	0.14	[0.02; 0.43]	0.5%	6.2%
LiuDongSheng 2019[404]	60	80	0.75	[0.64; 0.84]	55.1%	9.4%
Common effect model		**112**	**0.73**	**[0.64; 0.83]**	**55.9%**	**--**
Random effects model			**0.22**	**[0.05; 1.00]**	**--**	**20.0%**
Heterogeneity: $I^2 = 85\%$, $\tau^2 = 1.4740$, $p < 0.01$						
**Common effect model**		**1081**	**0.43**	**[0.39; 0.47]**	**100.0%**	**--**
**Random effects model**			**0.15**	**[0.09; 0.25]**	**--**	**100.0%**

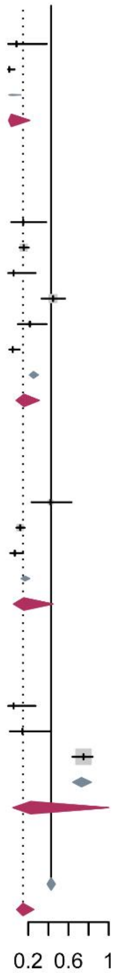

Heterogeneity: $I^2 = 95\%$, $\tau^2 = 0.8126$, $p < 0.01$
Test for subgroup differences (common effect): $\chi^2_3 = 173.23$, df = 3 ($p < 0.01$)
Test for subgroup differences (random effects): $\chi^2_3 = 2.79$, df = 3 ($p = 0.43$)

(F)

(f1)

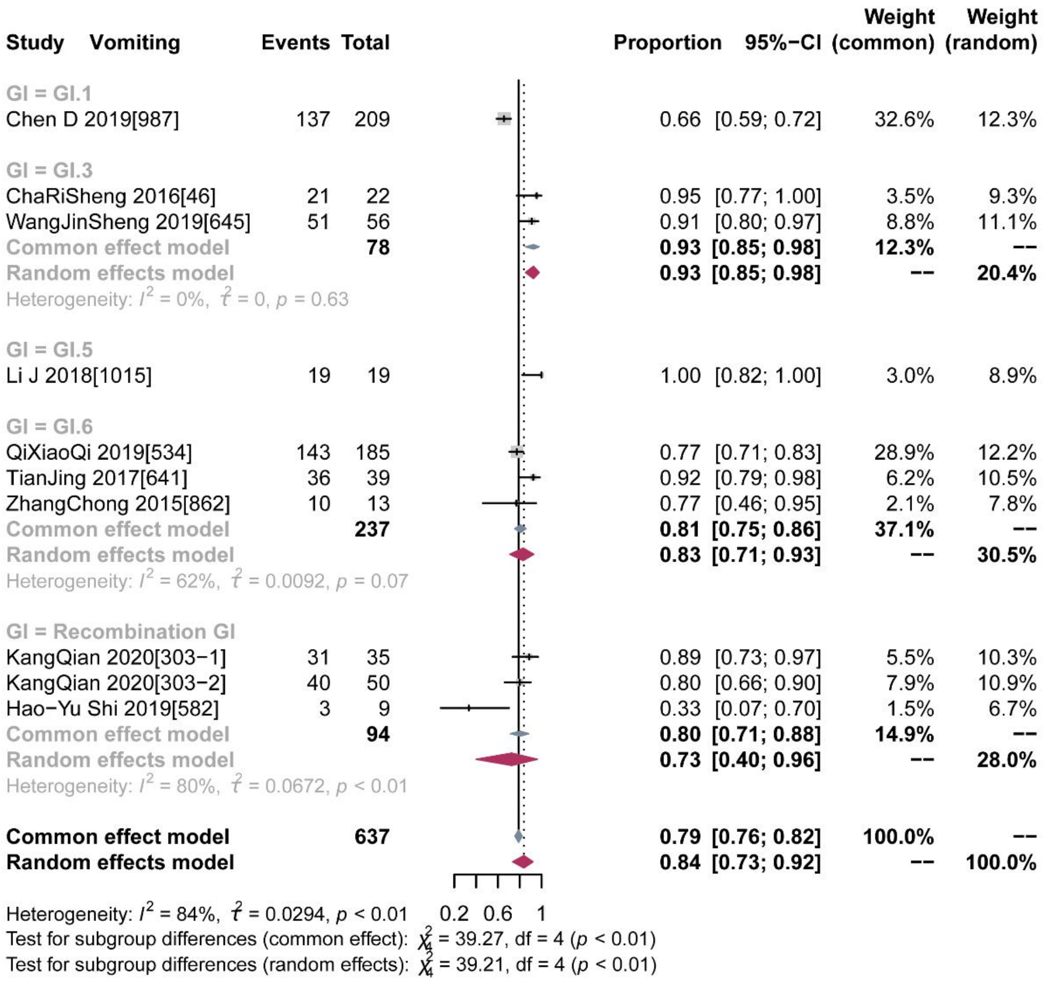

Study Vomiting Events Total Proportion 95%−CI Weight (common) Weight (random)
GI = GI.1
Chen D 2019[987] 137 209 0.66 [0.59; 0.72] 32.6% 12.3%
GI = GI.3
ChaRiSheng 2016[46] 21 22 0.95 [0.77; 1.00] 3.5% 9.3%
WangJinSheng 2019[645] 51 56 0.91 [0.80; 0.97] 8.8% 11.1%
Common effect model 78 0.93 [0.85; 0.98] 12.3% --
Random effects model 0.93 [0.85; 0.98] -- 20.4%
Heterogeneity: I^2 = 0%, τ^2 = 0, p = 0.63
GI = GI.5
Li J 2018[1015] 19 19 1.00 [0.82; 1.00] 3.0% 8.9%
GI = GI.6
QiXiaoQi 2019[534] 143 185 0.77 [0.71; 0.83] 28.9% 12.2%
TianJing 2017[641] 36 39 0.92 [0.79; 0.98] 6.2% 10.5%
ZhangChong 2015[862] 10 13 0.77 [0.46; 0.95] 2.1% 7.8%
Common effect model 237 0.81 [0.75; 0.86] 37.1% --
Random effects model 0.83 [0.71; 0.93] -- 30.5%
Heterogeneity: I^2 = 62%, τ^2 = 0.0092, p = 0.07
GI = Recombination GI
KangQian 2020[303−1] 31 35 0.89 [0.73; 0.97] 5.5% 10.3%
KangQian 2020[303−2] 40 50 0.80 [0.66; 0.90] 7.9% 10.9%
Hao−Yu Shi 2019[582] 3 9 0.33 [0.07; 0.70] 1.5% 6.7%
Common effect model 94 0.80 [0.71; 0.88] 14.9% --
Random effects model 0.73 [0.40; 0.96] -- 28.0%
Heterogeneity: I^2 = 80%, τ^2 = 0.0672, p < 0.01
Common effect model 637 0.79 [0.76; 0.82] 100.0% --
Random effects model 0.84 [0.73; 0.92] -- 100.0%
0.2 0.6 1
Heterogeneity: I^2 = 84%, τ^2 = 0.0294, p < 0.01
Test for subgroup differences (common effect): χ^2_4 = 39.27, df = 4 (p < 0.01)
Test for subgroup differences (random effects): χ^2_4 = 39.21, df = 4 (p < 0.01)

(f2)

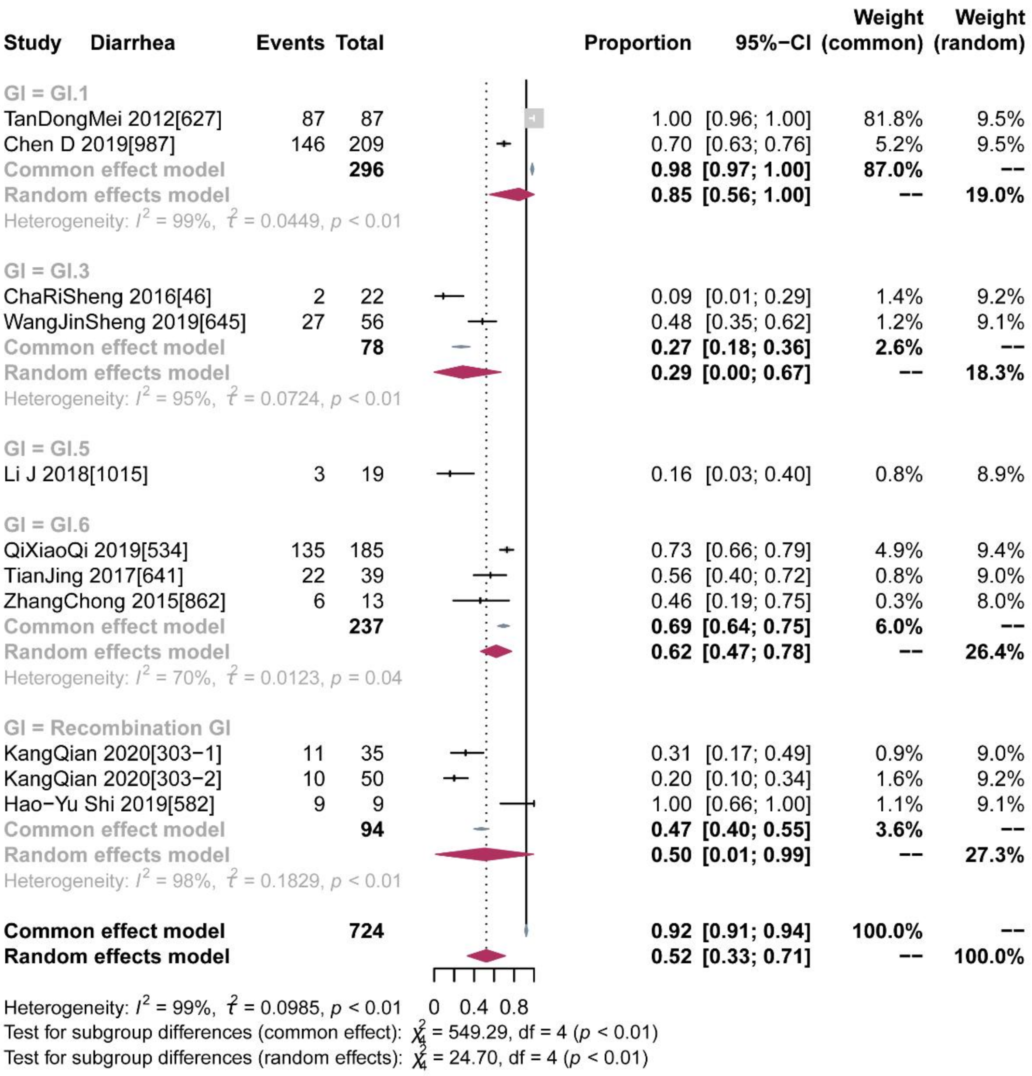
Study Diarrhea Events Total Proportion 95%−CI Weight (common) Weight (random)
GI = GI.1
TanDongMei 2012[627] 87 87 1.00 [0.96; 1.00] 81.8% 9.5%
Chen D 2019[987] 146 209 0.70 [0.63; 0.76] 5.2% 9.5%
Common effect model 296 0.98 [0.97; 1.00] 87.0% --
Random effects model 0.85 [0.56; 1.00] -- 19.0%
Heterogeneity: I^2 = 99%, τ^2 = 0.0449, p < 0.01
GI = GI.3
ChaRiSheng 2016[46] 2 22 0.09 [0.01; 0.29] 1.4% 9.2%
WangJinSheng 2019[645] 27 56 0.48 [0.35; 0.62] 1.2% 9.1%
Common effect model 78 0.27 [0.18; 0.36] 2.6% --
Random effects model 0.29 [0.00; 0.67] -- 18.3%
Heterogeneity: I^2 = 95%, τ^2 = 0.0724, p < 0.01
GI = GI.5
Li J 2018[1015] 3 19 0.16 [0.03; 0.40] 0.8% 8.9%
GI = GI.6
QiXiaoQi 2019[534] 135 185 0.73 [0.66; 0.79] 4.9% 9.4%
TianJing 2017[641] 22 39 0.56 [0.40; 0.72] 0.8% 9.0%
ZhangChong 2015[862] 6 13 0.46 [0.19; 0.75] 0.3% 8.0%
Common effect model 237 0.69 [0.64; 0.75] 6.0% --
Random effects model 0.62 [0.47; 0.78] -- 26.4%
Heterogeneity: I^2 = 70%, τ^2 = 0.0123, p = 0.04
GI = Recombination GI
KangQian 2020[303−1] 11 35 0.31 [0.17; 0.49] 0.9% 9.0%
KangQian 2020[303−2] 10 50 0.20 [0.10; 0.34] 1.6% 9.2%
Hao−Yu Shi 2019[582] 9 9 1.00 [0.66; 1.00] 1.1% 9.1%
Common effect model 94 0.47 [0.40; 0.55] 3.6% --
Random effects model 0.50 [0.01; 0.99] -- 27.3%
Heterogeneity: I^2 = 98%, τ^2 = 0.1829, p < 0.01
Common effect model 724 0.92 [0.91; 0.94] 100.0% --
Random effects model 0.52 [0.33; 0.71] -- 100.0%
0 0.4 0.8
Heterogeneity: I^2 = 99%, τ^2 = 0.0985, p < 0.01
Test for subgroup differences (common effect): χ^2_4 = 549.29, df = 4 (p < 0.01)
Test for subgroup differences (random effects): χ^2_4 = 24.70, df = 4 (p < 0.01)

(f3)

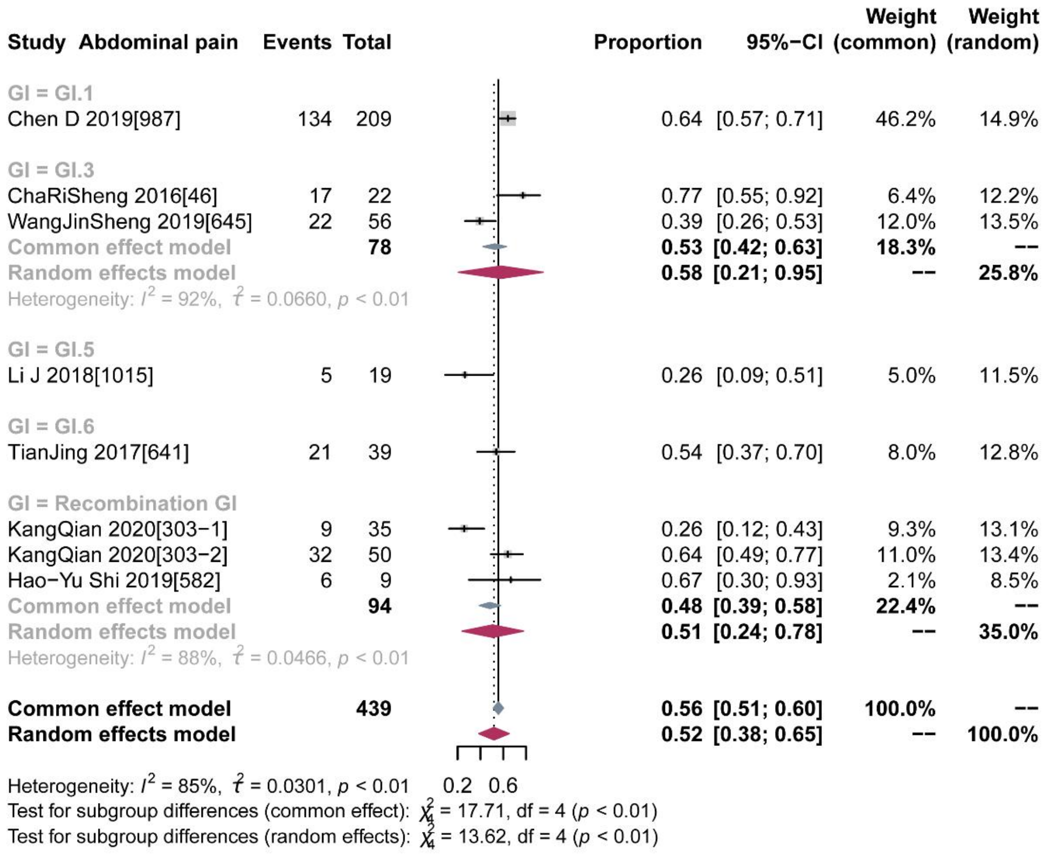
Study Abdominal pain Events Total Proportion 95%−CI Weight (common) Weight (random)
GI = GI.1
Chen D 2019[987] 134 209 0.64 [0.57; 0.71] 46.2% 14.9%
GI = GI.3
ChaRiSheng 2016[46] 17 22 0.77 [0.55; 0.92] 6.4% 12.2%
WangJinSheng 2019[645] 22 56 0.39 [0.26; 0.53] 12.0% 13.5%
Common effect model 78 0.53 [0.42; 0.63] 18.3% --
Random effects model 0.58 [0.21; 0.95] -- 25.8%
Heterogeneity: I^2 = 92%, τ^2 = 0.0660, p < 0.01
GI = GI.5
Li J 2018[1015] 5 19 0.26 [0.09; 0.51] 5.0% 11.5%
GI = GI.6
TianJing 2017[641] 21 39 0.54 [0.37; 0.70] 8.0% 12.8%
GI = Recombination GI
KangQian 2020[303−1] 9 35 0.26 [0.12; 0.43] 9.3% 13.1%
KangQian 2020[303−2] 32 50 0.64 [0.49; 0.77] 11.0% 13.4%
Hao−Yu Shi 2019[582] 6 9 0.67 [0.30; 0.93] 2.1% 8.5%
Common effect model 94 0.48 [0.39; 0.58] 22.4% --
Random effects model 0.51 [0.24; 0.78] -- 35.0%
Heterogeneity: I^2 = 88%, τ^2 = 0.0466, p < 0.01
Common effect model 439 0.56 [0.51; 0.60] 100.0% --
Random effects model 0.52 [0.38; 0.65] -- 100.0%
0.2 0.6
Heterogeneity: I^2 = 85%, τ^2 = 0.0301, p < 0.01
Test for subgroup differences (common effect): χ^2_4 = 17.71, df = 4 (p < 0.01)
Test for subgroup differences (random effects): χ^2_4 = 13.62, df = 4 (p < 0.01)

(f4)

Study	Nausea	Events	Total	Proportion	95%−CI	Weight (common)	Weight (random)
GI = GI.1							
Chen D 2019[987]		147	209	0.70	[0.64; 0.76]	55.7%	37.5%
GI = GI.3							
WangJinSheng 2019[645]		32	56	0.57	[0.43; 0.70]	12.7%	17.0%
GI = GI.6							
TianJing 2017[641]		22	39	0.56	[0.40; 0.72]	8.8%	12.9%
GI = Recombination GI							
KangQian 2020[303−1]		22	35	0.63	[0.45; 0.79]	8.3%	12.4%
KangQian 2020[303−2]		33	50	0.66	[0.51; 0.79]	12.4%	16.7%
Hao−Yu Shi 2019[582]		5	9	0.56	[0.21; 0.86]	2.0%	3.6%
**Common effect model**			**94**	**0.64**	**[0.54; 0.74]**	**22.8%**	**--**
**Random effects model**				**0.64**	**[0.54; 0.74]**	**--**	**32.6%**
Heterogeneity: $I^2 = 0\%$, $\tau^2 = 0$, $p = 0.83$							
**Common effect model**			**398**	**0.66**	**[0.61; 0.71]**	**100.0%**	**--**
**Random effects model**				**0.64**	**[0.58; 0.70]**	**--**	**100.0%**

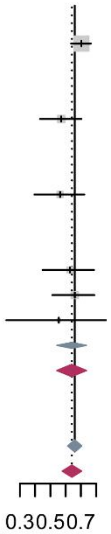

Heterogeneity: $I^2 = 12\%$, $\tau^2 = 0.0018$, $p = 0.34$

Test for subgroup differences (common effect): $\chi^2_3 = 5.31$, df = 3 ($p = 0.15$)

Test for subgroup differences (random effects): $\chi^2_3 = 5.31$, df = 3 ($p = 0.15$)

(f5)

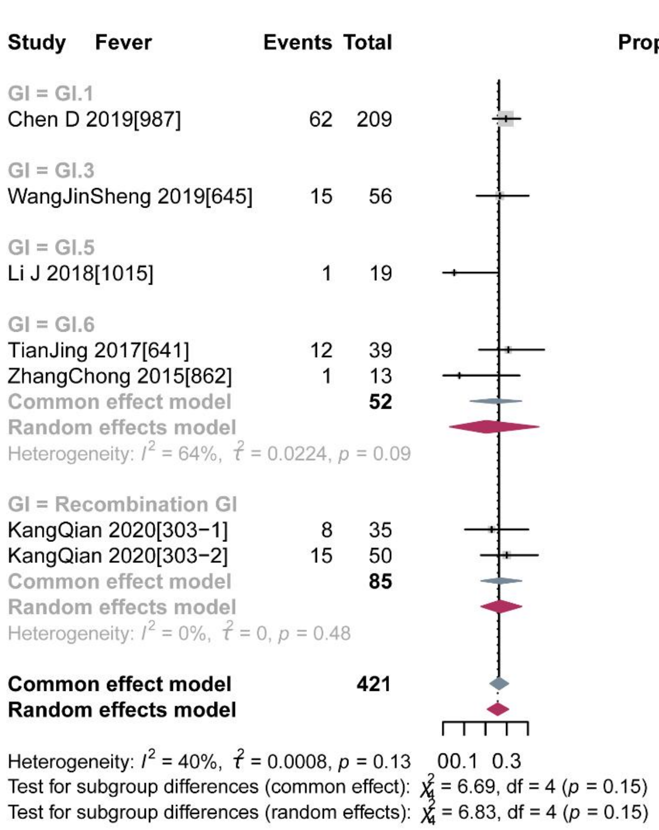
Study Fever Events Total Proportion 95%−CI Weight (common) Weight (random)
GI = GI.1
Chen D 2019[987] 62 209 0.30 [0.24; 0.36] 49.4% 39.8%
GI = GI.3
WangJinSheng 2019[645] 15 56 0.27 [0.16; 0.40] 13.3% 15.2%
GI = GI.5
Li J 2018[1015] 1 19 0.05 [0.00; 0.26] 4.6% 5.8%
GI = GI.6
TianJing 2017[641] 12 39 0.31 [0.17; 0.48] 9.3% 11.1%
ZhangChong 2015[862] 1 13 0.08 [0.00; 0.36] 3.2% 4.1%
Common effect model 52 0.24 [0.13; 0.37] 12.5% --
Random effects model 0.20 [0.03; 0.45] -- 15.2%
Heterogeneity: I^2 = 64%, τ^2 = 0.0224, p = 0.09
GI = Recombination GI
KangQian 2020[303−1] 8 35 0.23 [0.10; 0.40] 8.4% 10.1%
KangQian 2020[303−2] 15 50 0.30 [0.18; 0.45] 11.9% 13.8%
Common effect model 85 0.27 [0.18; 0.37] 20.3% --
Random effects model 0.27 [0.18; 0.37] -- 23.9%
Heterogeneity: I^2 = 0%, τ^2 = 0, p = 0.48
Common effect model 421 0.26 [0.22; 0.31] 100.0% --
Random effects model 0.26 [0.21; 0.31] -- 100.0%
Heterogeneity: I^2 = 40%, τ^2 = 0.0008, p = 0.13
00.1 0.3
Test for subgroup differences (common effect): χ^2_4 = 6.69, df = 4 (p = 0.15)
Test for subgroup differences (random effects): χ^2_4 = 6.83, df = 4 (p = 0.15)

(f6)

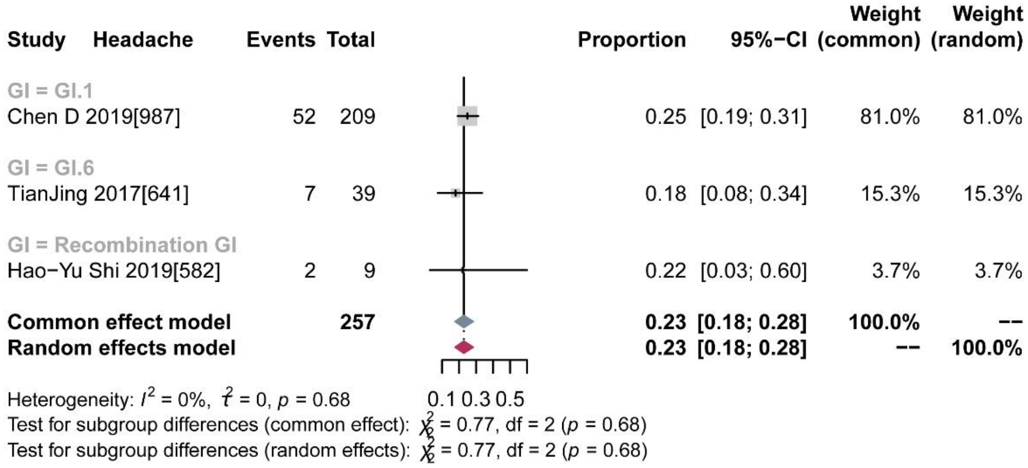
Study Headache Events Total Proportion 95%−CI Weight (common) Weight (random)
GI = GI.1
Chen D 2019[987] 52 209 0.25 [0.19; 0.31] 81.0% 81.0%
GI = GI.6
TianJing 2017[641] 7 39 0.18 [0.08; 0.34] 15.3% 15.3%
GI = Recombination GI
Hao−Yu Shi 2019[582] 2 9 0.22 [0.03; 0.60] 3.7% 3.7%
Common effect model 257 0.23 [0.18; 0.28] 100.0% --
Random effects model 0.23 [0.18; 0.28] -- 100.0%
0.1 0.3 0.5
Heterogeneity: I^2 = 0%, τ^2 = 0, p = 0.68
Test for subgroup differences (common effect): χ^2_2 = 0.77, df = 2 (p = 0.68)
Test for subgroup differences (random effects): χ^2_2 = 0.77, df = 2 (p = 0.68)

(f7)

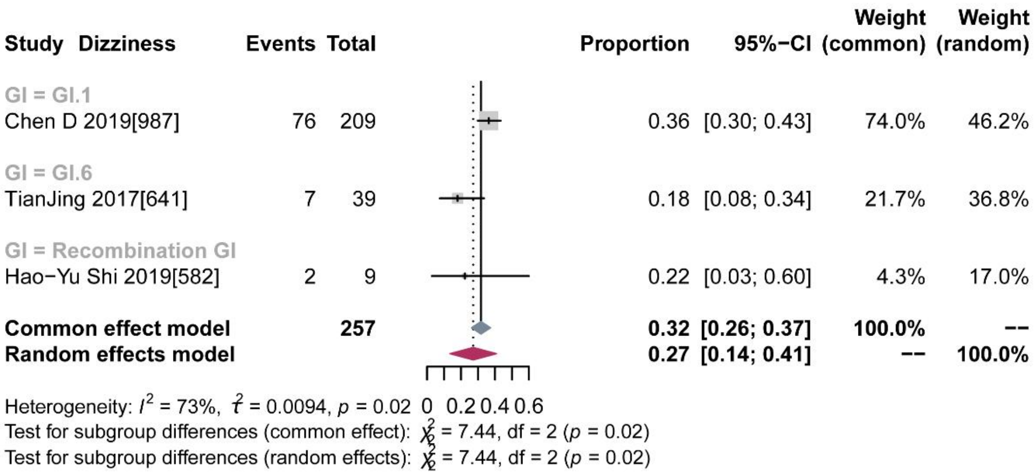
Study Dizziness
Events
Total
Proportion
95%−CI
Weight (common)
Weight (random)
GI = GI.1
Chen D 2019[987] 76 209 0.36 [0.30; 0.43] 74.0% 46.2%
GI = GI.6
TianJing 2017[641] 7 39 0.18 [0.08; 0.34] 21.7% 36.8%
GI = Recombination GI
Hao−Yu Shi 2019[582] 2 9 0.22 [0.03; 0.60] 4.3% 17.0%
Common effect model 257 0.32 [0.26; 0.37] 100.0% --
Random effects model 0.27 [0.14; 0.41] -- 100.0%
0 0.2 0.4 0.6
Heterogeneity: I^2 = 73%, τ^2 = 0.0094, p = 0.02
Test for subgroup differences (common effect): χ^2_2 = 7.44, df = 2 (p = 0.02)
Test for subgroup differences (random effects): χ^2_2 = 7.44, df = 2 (p = 0.02)
