# Supplementary material for: Prevalence and Etiological Characteristics of Norovirus Infection in China: A Systematic Review and Meta-Analysis
Source: Viruses. 2023 Jun 7;15(6):1336. doi: 10.3390/v15061336 (PMC10302178; doi:10.3390/v15061336)
Supplement: Supplementary file 1 [file viruses-15-01336-s001.zip › Appendix S4.pdf]

Appendix S4: Included Reference

1. An SY, Zhao Z, Guo JQ, Han Y, Wang ZS, Ren Y, Zhou BS. Epidemiological study on viral diarrhea during 2009–2011 in Liaoning Province (in Chinese). *Chin J Infect Dis* **2013**, *3*, 166–169.
2. An XW. Analysis of Active Surveillance of Foodborne Diseases in Hongqiao District, Tianjin from 2018 to 2019 (in Chinese). *Continu Med Edu* **2021**, *11*, 113–115.
3. Bai AL. Analysis of norovirus infection in a school in Beichen District, Tianjin (in Chinese). *Henan J Prev Med* **2020**, *10*, 784–785.
4. Bai J, Zhang N, Liu W, Han SY, Ji LL. Active surveillance results of foodborne disease in Haidian District of Beijing from 2014–2017 (in Chinese). *Occup & Health* **2018**, *16*, 2207–2211.
5. Bai X, Zhang SH, Shen ZX. Analysis on gene of norovirus from sporadic diarrhea cases in Hebei province (in Chinese). *Int J Viro* **2020**, *1*, 44–47.
6. Bai Y, Li Y, Liu JF, Chen ZJ. An outbreak of norovirus infection in a hospital in Xi'an, Shaanxi, 2019 (in Chinese). *Dis Surveil* **2020**, *8*, 773–776.
7. Bai YF, Lu J, Yu XL, Chen Y, Chen X, Zhu J. Etiological study on viral pathogens in 654 acute diarrhea cases (in Chinese). *Chin J Health Lab Tech* **2014**, *4*, 556–558.
8. Bai Y, Cai WL, Ren LJ, Bai XX. An outbreak of gastroenteritis caused by norovirus in a school in Shijingshan district, Beijing (in Chinese). *Capital J Public Health* **2018**, *4*, 208–210.
9. Bao L, Liu C, Pang YY, Tan JC, Cui PW, Tan YY, Hang H. Etiological analysis of viral diarrhea in children aged< 5 years in Suzhou, 2014–2015 (in Chinese). *Jiangsu J Prev Med* **2019**, *6*, 668–670.
10. Bi H, Zhong Y, Wang DD, Fu XS, Zhou ZY, Liu L. Field investigation of an outbreak of GII norovirus gastroenteritis (in Chinese). *China Health Industry* **2014**, *15*, 130–131.
11. Bi H, Zhong Y, Wang DD, Zhou ZY, Fu XS, Li ZR, Huang RD, Li XT. A survey on the outbreak of gastroenteritis caused by source water polluted by GII style norovirus (in Chinese). *J Med Pest Control* **2015**, *8*, 918–920.
12. Bi WJ, Xu JJ. Distribution of viral pathogens among child outpatients with acute diarrhea, 2015–2018 (in Chinese). *Chin J Public Health* **2020**, *9*, 1371–1373.
13. Bi WJ, Xu JJ, Yan JT, Liu YB, Wang M. Surveillance and analysis of diarrhea pathogens in a hospital in Shanghai from 2016 to 2018 (in Chinese). *Pract Prev Med* **2019**, *11*, 1369–1372.
14. Cai J, Chen EF, Ding H, Sun Z, Xu XJ, Tong WS, Wang XC, Chen YF. Investigation of an outbreak of acute gastroenteritis with homologous norovirus in two schools (in Chinese). *Chin J School Health* **2013**, *9*, 1148–1149.
15. Cai MS, Xu SJ, Li YH, Lai XL, Cai JH, He XM, Liu AP, Sun HJ, Quan DF, Ren Y. Analysis of infective diarrhea pathogen monitoring during 2014–2015 in Shenzhen Longhua (in Chinese). *J Trop Med* **2016**, *11*, 1455–1457.
16. Cai M, Wang DY, Shen MZ, Chang WB. Pathogenic characteristics and burden of patients with foodborne diseases in Changshu City in 2018 (in Chinese). *Occup & Health* **2019**, *19*, 2633–2637.
17. Cai MW. Epidemiological characteristics of 12 norovirus infection outbreaks in schools at Liwan District, Guangzhou (in Chinese). *Int Med & Health Guid News* **2021**, *15*, 2338–2342.
18. Cai MW, Lu L, Wang H, Yang CH, Wang M. Investigation of the Norovirus diarrhea outbreak cause by the recessive infection kitchen staff in Guangzhou in the summer (in Chinese). *J Med Pest Control* **2018**, *5*, 413–416.
19. Kong FM. Study of epidemic genotypes of norovirus in Linyi, 2015–2017 (in Chinese). *Chin J Health Lab Tech* **2019**, *4*, 421–423.
20. Cai SJ, Wu SG, Zhan MR, Huang ZQ, Ou JM, Chen W. Cost–benefit analysis on a norovirus outbreak in Fujian (in Chinese). *Strait J Prev Med* **2018**, *3*, 17–18.
21. Cai SX, Han W. Investigation and analysis of gastroenteritis infected with norovirus in kindergarten (in Chinese). *China Health Care & Nutr* **2017**, *14*, 343–344.
22. Cai T, Yue LX, Zhang KY. Detection and analysis of norovirus in diarrhea patients in Dalian in 2013 (in Chinese). *Strait J Prev Med* **2015**, *5*, 38–39.
23. Cai W, Liu LX, Ma N, Ying HQ, Hou WY, Zhao Z, Liu F, Han W, Wang J. Epidemiological investigation and analysis of the norovirus infection outbreaks in Beifing in 2016 (in Chinese). *Chin J Viral Dis* **2018**, *4*, 288–292.
24. Cai W, Ying HQ, Xing Y, Hua WY, Liu FY, Wang L, Hou WY, Zhao Z, Liu F, Zhang HF. Epidemiological characteristics of clusters or outbreaks of Norovirus infection in Haidian district, Beijing, 2016 (in Chinese). *Dis Surveil* **2017**, *8*, 656–659.
25. Cai WF, Xie HP, Liu YF, Yuan J, Xiao XC, Ding P, Chen C, Zhang D, Chen JD, Ma XW, et al. An epidemiological investigation on a food–born outbreak of norovirus caused by Sydney_2012 GII.4 strain (in Chinese). *Chin J Epidemiol* **2013**, *8*, 804–807.
26. Cai WF, Yang CH, Ma XW, Yang LL, Fan JW, Liu YF, Chen JD. An epidemiological investigation on a suspected foodborne outbreak of norovirus (in Chinese). *J Trop Med* **2014**, *2*, 254–257.
27. Cai XZ, Xu HN, Pan YH, Zhang GR, Deng MR, Wang SP, Liu JR. Analysis on outbreaks caused by norovirus in Harbin in 2017 (in Chinese). *Chin J Public Health Manage* **2018**, *5*, 712–714.
28. Cai Y, Xie L, Xu JY. Epidemiological features of rotavirus and norovirus diarrhea in Ningbo from 2013 to 2015 (in Chinese). *Chin Prev Med* **2017**, *11*, 836–839.
29. Cao J, Xu H, Xiao JW. Study on prevalence of pathogen for diarrhea in Yangpu District, Shanghai (in Chinese). *Chin Primary Health Care* **2017**, *3*, 58–59.
30. Cao RR, He Y, Wu XY, Ma XZ, Ren M. Molecular etiological characteristics of the gastroenteritis outbreak associated with Norovirus infection in Deyang city, 2018 (in Chinese). *Chin J Exp & Clin Virol* **2020**, *3*, 284–288.

31. Cao RR, Ma XZ, Liao XC, Ren M. Infantile diarrhea with Picobimavirus, Chengdu (in Chinese). *Modern Prev Med* **2015**, *23*, 4250–4251. +4261.
32. Cao S, Wu HY, Han X, Xiao ZP, Zhang ZX, Wang L. Investigation on an outbreak of Norovirus gastroenteritis in a university in Shanghai (in Chinese). *J Trop Med* **2019**, *11*, 1434–1437.
33. Cao XP, Ding ZY, Wei CC, Wang Y. Investigation and analysis of an outbreak of norovirus acute gastroenteritis in Wuxi (in Chinese). *World Latest Med Inform* **2018**, *92*, 284–286.
34. Cao YH. Surveillance and analysis of viral diarrhea pathogens in Yunnan province from 2018 to 2019 (in Chinese). *Lab Med & Clin* **2021**, *12*, 1681–1683.
35. Cao YH. Analysis of pathogen detection of viral diarrhea in Southwest China from 2017 to 2019 (in Chinese). *J Public Health & Prev Med* **2021**, *1*, 10–13.
36. Cao YH, Xiang YB, Jiang LL, Zhou XF, Yin J, Yin JW, Gu WP, Cun JP. Analysis on diarrhea pathogens in Yunnan, Guizhou, Sichuan and Chongqing from 2012 to 2015 (in Chinese). *Int J Epidemiol & Infect Dis* **2017**, *5*, 292–295.
37. Cao YH, Zhou XF, Jiang LL, Cun JP, Xiang YB. Analysis of the surveillance results of viral diarrhea in Yunnan from 2012 to 2015 (in Chinese). *Int J Viro* **2018**, *1*, 35–38.
38. Cao YN, Zhang XJ, Chen N. Epidemiological surveillance of norovirus and rotavirus in children with diarrhea (in Chinese). *J Public Health & Prev Med* **2020**, *5*, 42–44.
39. Cen YZ, Wang T, Chen XQ, Shu B, Wang M. Investigation on outbreak of norovirus infection caused by a kindergarten parent–child gathering in Shan City (in Chinese). *South China J Prev Med* **2014**, *4*, 377–379.
40. Zeng DX, Gao MS, Peng T. Analysis and Significance of Virus Test in Children with Diarrhea (in Chinese). *Chin & Foreign Med Res* **2015**, *1*, 69–70.
41. Zeng FM, Wang H, Zhang JE, Yang F, Li MX, Li YL, Luo L. Outbreak investigation of infectious diarrhea associated with norovirus GII.4 in a kindergarten in Guangzhou (in Chinese). *Dis Surveil* **2020**, *4*, 357–361.
42. Zeng HS, Zhou HT, Hou HB, Chen RL, Lai ZF, Zhang Y. Main pathogen detection and study of child viral diarrhea in Shenzhen in 2013 (in Chinese). *Chin J Health Lab Tech* **2015**, *3*, 351–352. +355.
43. Zeng JR, Wang YL, Bai PS, Li RJ. Molecular epidemiological study of norovirus infection in Conghua, Guangzhou in 2011 (in Chinese). *Guangdong Med J* **2013**, *4*, 549–551.
44. Zeng L, Shen YP. Epidemiological analysis of an outbreak of acute gastroenteritis caused by norovirus in a kindergarten in 2017 (in Chinese). *Electron J Clin Med Literature* **2018**, *64*, 171–172.
45. Cha J, Ma ZL, Ding W, Yang MD, Dai WJ. Pathogen prevalent in the epidemic of acute gastroenteritis, Taizhou (in Chinese). *Modern Prev Med* **2020**, *5*, 908–912.
46. Cha RS, Xia Y, Lu RK, Guo F, Zhang YH, Wu WH. An acute gastroenteritis outbreak caused by norovirus GI in a kindergarten (in Chinese). *J Med Pest Control* **2016**, *10*, 1149–1151.
47. Cha RS, Xia Y, Ya XR, Hang H, Liu C, Li J. Characteristic of 14 norovirus gastroenteritis outbreaks in Suzhou City (in Chinese). *Jiangsu J Prev Med* **2014**, *6*, 14–16.
48. Chang HL, Ceng M, Huang Z, Cai JH, Guo JY, Xu XB, Wang XS, Ge YL, Wang ZL. Surveillance of enteric pathogens in outpatient children with acute diarrhea (in Chinese). *Chin J Infect Dis* **2016**, *1*, 19–22.
49. Chang XH, Chang FY, Zhao JF, Dong JF. Investigation on Norovirus Infection in Foodborne Diarrhea Patients in Qingdao City (in Chinese). *Chin J Clin Rational Drug Use* **2017**, *16*, 133–134.
50. Shen JC, Lin JF, Gao J, Yao WT, Wen D, Liu GT, Han JK, Ma HL, Zhang LJ, Zhu BP. A norovirus–borne outbreak caused by contaminated bottled spring water in a school, Zhejiang province (in Chinese). *Chin J Epidemiol* **2011**, *8*, 800–803.
51. Shen MY, Zhang Q. Analysis of surveillance results of foodborne diseases in Xishan District, Wuxi City from 2014 to 2015 (in Chinese). *Chin J School Doct* **2016**, *10*, 756–757.
52. Shen YP. Epidemiological investigation and analysis of an outbreak of norovirus infection (in Chinese). *China Rural Health* **2018**, *22*, 80. +82.
53. Shen YP, Xu YX. Epidemiological investigation of an outbreak of norovirus–infected diarrhea in a school (in Chinese). *Jiangsu J Prev Med* **2013**, *4*, 48–49.
54. Shen YG, Ying XJ, Zhu XC, Qiu L, Qian XY. Investigation and analysis of an outbreak of norovirus type II acute gastroenteritis in a rural school (in Chinese). *Chin Rural Health Service Adm* **2016**, *1*, 72–75.
55. Shen YG, Ying XJ, Zhu XC, Qiu L, Shi ZW, Xiong MJ. Outbreak of GII Norovirus gastroenteritis in a middle school of Zhejiang Province (in Chinese). *Chin Rural Health Service Adm* **2016**, *9*, 1154–1158.
56. Shen YH, Ji L, Chen LP, Zhu XJ, Cha YF, Xu DS, Wu XF. Infection status and genetic diversity of noroviruses in patients with acute gastroenteritis in Huzhou, Zhejiang, 2014 (in Chinese). *Dis Surveil* **2016**, *11*, 941–944.
57. Chen AQ, Chen MY, Wei ZN, Zhou HY. Epidemiological characteristics and surveillance results analysis on other infectious diarrheal diseases in Jiangmen, 2012–2016 (in Chinese). *Dis Surveil* **2017**, *12*, 936–939.
58. Chen AQ, Gao SP, Sun LM, Zhao SQ, Chang CY, Xiao XC. Investigating an outbreak of norovirus–infected diarrhea using a case–control approach (in Chinese). *World Latest Med Inform* **2015**, *67*, 172.
59. Chen AQ, Gao SP, Sun LM, Zhao SQ, Chang CY, Xiao XC. Investigation and analysis on an outbreak of infectious diarrhea caused by norovirus in a university in Guangzhou in 2014 (in Chinese). *Appl Prev Med* **2016**, *1*, 15–18. +25.
60. Chen BB, Pan F, Sun Z. Molecular epidemiological characteristics of two outbreaks caused by norovirus GII.17 genotype during December 2014 and January 2015 in Hangzhou, China (in Chinese). *Chin J Viral Dis* **2017**, *6*, 444–448.
61. Chen B, Weng ZJ, Chen MJ, Chen BQ, Chen WX, Chen JH. Molecular epidemiological analysis of norovirus in outbreaks of acute gastroenteritis in school (in Chinese). *Chin J Health Lab Tech* **2019**, *2*, 174–175. +182.

62. Chen CR, Zhang HF, Liang HB. An epidemic of mixed infection of norovirus GI and GII (in Chinese). *Dis Surveil* **2020**, *1*, 86–89.
63. Chen C, Wang HL, Yan JB, Li KF, Zhang H, Wu B, Li P. Etiological identification and molecular characteristics of a norovirus outbreak, Zhoushan (in Chinese). *Chin J Zoonoses* **2018**, *11*, 1026–1032. +1048.
64. Chen C, Wang DH, Xie HP, Geng JM, Xiao XC. Epidemiological analysis is of norovirus outbreak in a primary school in Guangzhou in 2013 (in Chinese). *Int J Epidemiol & Infect Dis* **2014**, *4*, 255–257.
65. Chen FQ, Weng SF, Zhuo L. Pathogen analysis on 156 cases with infectious diarrhea in Children's Hospital (in Chinese). *National Med Front China* **2013**, *15*, 6–7.
66. Chen FQ, Zhuo L, Lin WD, Ye LY, Wu BS. Pathogen isolation in 459 infants with acute diarrhea (in Chinese). *Chin J Pract Pediatr* **2012**, *5*, 368–370.
67. Chen GS, Shen TPP, Zhang T. Epidemiological characteristics and etiological analysis of foodborne diseases among primary and secondary school students in Jinhua City from 2017 to 2019 (in Chinese). *Chin J School Health* **2020**, *7*, 1084–1087.
68. Chen GS, Chen MA. Analysis of foodborne disease surveillance results in Jinhua city (in Chinese). *Prev Med* **2018**, *12*, 1234–1237.
69. Chen GC, Xia W. Investigation of a foodborne disease outbreak caused by norovirus contamination (in Chinese). *Chin Primary Health Care* **2011**, *11*, 76–77.
70. Chen GP. Etiological study on viral diarrhea among patients under five years old in sentinel hospital of Anhui Province (2010–2015) (in Chinese). *J Public Health & Prev Med* **2018**, *1*, 56–59.
71. Chen HL, Yang CY, Zhang WJ, Liu Y, Tian D, Wang W, Yi ZG, Zhu ZQ. Prevalence of Norovirus GII in adult acute gastroenteritis outpatients in Shanghai from 2015 to 2016 (in Chinese). *Chin J Exp & Clin Virol* **2020**, *2*, 145–150.
72. Chen HC, He L, Mei SJ, Liao YX, Qing WJ, Ye QK, Zhan DN, Yang M. Etiological analysis of food–borne viral diarrhea in Longhua District, Shenzhen from 2014 to 2017 (in Chinese). *Pract Prev Med* **2019**, *8*, 981–983.
73. Chen HJ, Wang PY. Investigation and analysis of an outbreak of norovirus acute gastroenteritis (in Chinese). *Psychologist* **2018**, *22*, 45–46.
74. Chen H, Yao JZ. Outbreak of norovirus infection in geriatric ward: control measures and nursing care (in Chinese). *J Nurs Sci* **2012**, *1*, 86–87.
75. Chen HF, Hu TT, Yao YX, Huang YH, Xiao N, Liu X, Xiao Y, Chen Q, Yu SY. Etiological and epidemic characterization of viral diarrhea in children under the age of 5 years in Guangzhou City (in Chinese). *Chin J Dis Control & Prev* **2014**, *4*, 336–339.
76. Chen HZ, Liu B, Liu FY, Liu XM. Results of active surveillance of foodborne diseases in Shenyang city, 2016–2018 (in Chinese). *Pract Prev Med* **2019**, *9*, 1040–1042.
77. Chen HZ, Liu B, Liu XM, Liu FY. Surveillance analysis of foodborne diseases in sentinel hospitals in Shenyang from 2015 to 2016 (in Chinese). *Pract Prev Med* **2018**, *12*, 1514–1516.
78. Chen J, He YH, Zhong JM, Deng KJ, Duan LN, Xu YC, Dong C. A norovirus–borne outbreak investigation in a primary school, Shenzhen (in Chinese). *Modern Prev Med* **2016**, *15*, 2848–2851.
79. Chen JM, Dong JY, Zhang SH, Wang M. Investigation and analysis of an outbreak of infectious diarrhea caused by norovirus (in Chinese). *Anhui J Prev Med* **2017**, *5*, 335–338.
80. Chen J, Gao ZW, Lin JG. Investigation and handling of an outbreak of norovirus gastroenteritis in a school (in Chinese). *Strait J Prev Med* **2017**, *2*, 91–93.
81. Chen J, Qi XJ, Zhang Z, Chen LL, Sun L, Liao NB, Zhang RH. Surveillance data analysis of foodborne diseases in Yinzhou District diseases in Zhejiang in 2016 (in Chinese). *Chin Prev Med* **2019**, *1*, 6–10.
82. Chen JH, Zhong XZ, Lin JM. Analysis of Surveillance Results of Viral Diarrhea in Shantou City from 2012 to 2013 (in Chinese). *Pract Prev Med* **2015**, *9*, 1121–1123.
83. Chen J. Epidemiological Analysis of Foodborne Disease Surveillance in A District from 2017 to 2019 (in Chinese). *Guid China Med* **2020**, *14*, 16–18.
84. Chen JF, Yao D, Zhang RS, Ou XH, Zhao J, Li LZ, Sun BC. Recombinant norovirus associated with gastroenteritis outbreaks in Changsha in 2016 (in Chinese). *J Pathog Bio* **2018**, *3*, 295–298. +305.
85. Chen J, Sui Y, Ceng P, Cao J, Liu M, Wu XJ. Surveillance of foodborne diseases in Jurong, 2016–2018 (in Chinese). *Modern Prev Med* **2020**, *1*, 132–135.
86. Chen LL, Chen J, Liao NB, Sun L, Qi XJ, Zhang RH. Epidemiological characteristics of norovirus infectious diarrhea in Zhejiang Province, 2014–2016 (in Chinese). *Chin J Health Lab Tech* **2018**, *7*, 863–866.
87. Chen LP, Ji L, Shen YH, Xu DS. Epidemic characteristics of norovirus in Huzhou during 2015–2017 (in Chinese). *Chin J Health Lab Tech* **2020**, *6*, 666–668. +672.
88. Chen LL, Zhou Y, Lin XC, Du J, Liu Y, Wang Y, Li HJ. Genotypes and epidemiological characteristics of Noroviruses in Shenzhen during the winter of 2017 (in Chinese). *Chin J Microbiol & Immunol* **2018**, *10*, 725–730.
89. Chen LX. Pathogen and epidemiological analysis of viral diarrhea in Huairou District, Beijing from 2013 to 2015 (in Chinese). *Pract Prev Med* **2016**, *5*, 603–605.
90. Chen LH, Chen CH, Yu LG. Epidemiological survey of 459 patients with diarrheal disease (in Chinese). *Henan Med Res* **2015**, *4*, 37–38.
91. Chen M, Qiao J, Sun JP, Zheng JF, Jiang XJ. Shanghai East Hospital (south courtyard) Analysis of Pathogens in Children Acute Infective Gastroenteritis (in Chinese). *Parasitol & Infect Dis* **2016**, *2*, 81–83.
92. Chen MH, Jia HM, Wang JQ, Ji HL. Analysis of three norovirus gastroenteritis outbreaks in Fuzhou in 2016 (in Chinese). *Strait J Prev Med* **2017**, *6*, 79–81.
93. Chen MM, Zhou KJ, Ju Y, Tan Y. The first detection of Norovirus GII.4 Sydney variant in Guangxi, 2012 (in Chinese). *Dis Surveil* **2014**, *1*, 28–31.

94. Chen P, Xu ZL, Cui DW, Xie GL, Yang XZ, Han DS, Zheng SF, Yu F, Chen Y. Determination and analysis of enteric viruses from children with acute diarrhea in Zhejiang province (in Chinese). *China Modern Doct* **2013**, *12*, 109–113.
95. Chen Q, Jie XH, Zhou P, Ding JQ, Hou CC. Investigation of a case of norovirus diarrhea outbreak in Tianjin (in Chinese). *J Environ & Health* **2014**, *3*, 250–252.
96. Chen Q, Li J, Chen C, Zu RQ. Investigation on outbreak of acute gastroenteritis caused by GI norovirus in a junior high school of Changzhou City (in Chinese). *Occup & Health* **2021**, *2*, 258–261. +265.
97. Chen WP, Cui DW, Yang XZ, Zheng SF, Xie GL, Chen Y. Molecular epidemiological analysis of norovirus in patients with acute gastroenteritis in Zhejiang province during 2016 (in Chinese). *Chin J Clin Lab Sci* **2018**, *4*, 304–308.
98. Chen XK, Liao HY, Ye Q, Hu PW, Cao X, Pei XF. Study on norovirus infection in diarrhea of children in Chengdu (in Chinese). *Modern Prev Med* **2012**, *23*, 6123–6125. +6129.
99. Chen XD, Zhong YP, Chen HB, Li MH, Guo ZZ, Liao YH. Active surveillance results and analysis of norovirus in Longyan City in 2019 (in Chinese). *The Med Forum* **2020**, *35*, 5168–5170.
100. Chen XF, Sheng S. Investigation on an Aggregation Epidemic of Gastroenteritis Caused by Norovirus in a Kindergarten (in Chinese). *J Prev Med Inform* **2015**, *9*, 697–698.
101. Chen X, Cao YL, Cao H, Wu B. Investigation of the Infection of Close Contacts and Environmental Contamination in a Norovirus Outbreak (in Chinese). *Sichuan J Anatomy* **2020**, *2*, 14–16.
102. Chen XH, Li WJ, Xu XC, Su S, Ceng GF. Epidemiological Analysis of An Outbreak of Norovirus Diarrhea (in Chinese). *Henan J Prev Med* **2012**, *2*, 120–122.
103. Chen XF, Wei Z, Feng HP, Wang X, Deng HM. Investigation on an outbreak of norovirus diarrhea in a primary school (in Chinese). *Anhui J Prev Med* **2018**, *1*, 51–52. +66.
104. Chen Y, Cai Y, Wang YP. Report on the investigation and handling of the outbreak of norovirus infection in Wuqiao Middle School, Jiangdu District, Yangzhou City (in Chinese). *Electron J Gen Stomatol* **2019**, *29*, 190. +198.
105. Chen Y, Li P, Wang HL, Zhang YL. Analysis of Surveillance Results of Foodborne Diseases in Zhoushan City (in Chinese). *Prev Med* **2020**, *4*, 398–401.
106. Chen Y, Zhu FY. Analysis of the active surveillance results of foodborne diseases in Zhoushan in 2014 (in Chinese). *Chin J Health Lab Tech* **2016**, *7*, 1025–1026.
107. Chen YW, Gao ZY, Yan HQ, Liu BW, Tian Y, Jia L, Li XY, Wang QY, He Y. Epidemiological characteristics of norovirus infection among older adults with diarrhea in Beijing, between 2011 and 2015 (in Chinese). *Int J Viro* **2017**, *1*, 36–39.
108. Chen YX, Ceng JS, Li Y, Xiong TT, Tang GX, Li J. Investigation and analysis on an outbreak of infectious diarrhea caused by norovirus in a university in Guangzhou in 2014 diarrhea caused by norovirus in a school (in Chinese). *Prev Med Tribune* **2018**, *9*, 683–685.
109. Chen YY, Ruan CH, Fan SG. Investigation of a norovirus diarrhea outbreak in a university of Guangdong Province (in Chinese). *Chin J School Doct* **2015**, *7*, 510–512. +514.
110. Chen Y. Infection and genotypes of norovirus among children with acute diarrhea in sentinel hospitals in Hunan Province, 2012–2014 (in Chinese). *Pract Prev Med* **2016**, *7*, 773–776.
111. Chen YF, Pan W, Luan MC, Song XY, Bao ZJ. Analysis on surveillance results of foodborne diseases in Dalian City from 2017–2019 (in Chinese). *Occup & Health* **2021**, *11*, 1473–1477.
112. Chen Y, Zhu JM, Yuan YJ, Ling QF, Chen Y. Analysis of pathogen distribution and epidemic characteristics of viral diarrhea in adults during 2014–2015 in Jiashan, Zhejiang (in Chinese). *Chin J Health Lab Tech* **2017**, *8*, 1161–1163.
113. Chen ZM, Cao XO, Ceng HH, Liang ZL, Zhang YL. Monitoring and analysis on pathogens of viral gastroenteritis in Nanhai of foshan (in Chinese). *Modern Prev Med* **2012**, *4*, 995–996. +998.
114. Chen ZH, Huang GL, Chen XL, She YS, Zhou YL. Detection and genotyping of norovirus in diarrhea infant feces in Xiamen (in Chinese). *Chin J Health Lab Tech* **2012**, *11*, 2654–2656.
115. Chen ZQ, Su LY, Song TX. An Investigation on a Case of Foodborne Clustering Norovirus Gastroenteritis in a Primary School (in Chinese). *J Prev Med Inform* **2017**, *2*, 177–181.
116. Cheng C, Huang X, Xu J, Zhang LL, Zhou H. Viral epidemiological analysis of infectious diarrhea in Taizhou in 2017 (in Chinese). *Electron J Clin Med Literature* **2019**, *87*, 182–183.
117. Cheng P, Shi YL, Yang XS, Zhang ZX. Molecular typing and genetic characterization of norovirus in two outbreaks in Huangshan City in2018 (in Chinese). *Anhui J Prev Med* **2019**, *3*, 174–177.
118. Cheng SS, Dong Y, Liu GL, Sun X, Zhao Q. Etiological analysis of viral diarrhea among preschoolers in Shijiazhuang from 2012 to 2017 (in Chinese). *J Pathog Bio* **2018**, *12*, 1389–1392. +1396.
119. Cheng Y, Wu XX. Etiological detection and bacterial resistance analysis of infectious diarrhea cases in Xiwuyuan County (in Chinese). *Appl Prev Med* **2017**, *4*, 295–297.
120. Chu XJ, Li Y, Shang H. A Nosocomial Norovirus Outbreak in Senior Inpatients (in Chinese). *J Environ & Occup Med* **2015**, *6*, 557–560.
121. Cui DW, Li ZJ, Lin J, Jin M, Yang XZ, Xie GL, Zheng SF, Yu F, Chen Y. Epidemiologic characteristics of noroviruses isolated in outpatients with acute gastroenteritis in Hangzhou area, from 2014 to 2015 (in Chinese). *Chin J Epidemiol* **2016**, *2*, 254–258.
122. Yan YX. Epidemiological Characteristics of Norovirus in Lanzhou from 2015 to 2018 and in Part of China in 2018. Master, Lanzhou University; 2020.
123. Cui LL, Geng XY, Cao RM, Zhou JW, Zhang J. Epidemiological characters of several norovirus outbreaks in Jinan City (in Chinese). *J Public Health & Prev Med* **2017**, *5*, 11–14.

124. Cui XM, Sun YY, Ma WB, Qin SJ, Qi Q, Li YS, Xu YH, Dong B. Investigation and analysis of cluster epidemic situation of norovirus infectious gastroenteritis in kindergartens (in Chinese). *Med J Chin People's Health* **2015**, *18*, 101–103.
125. Cui XM, Sun YY, Qin SJ, Li YW, Yang AY, Liu F, Dou JZ. Investigation and analysis of a cluster of norovirus infection in a primary school in Dalian (in Chinese). *Med J Chin People's Health* **2018**, *18*, 76–77. +91.
126. Cui XS, Feng GS, Jin F, Xu XW. Investigation and clinical analysis of norovirus and rotavirus infection in 942 children with acute diarrhea (in Chinese). *Chin J Pract Pediatr* **2016**, *8*, 603–607.
127. Dai BN, Zhu R, Wu Y, Huang H, Wang F. Investigation on a school outbreak of norovirus infectious diarrhea caused by contaminated distribution cakes in Hubei Province (in Chinese). *Occup & Health* **2020**, *4*, 478–481.
128. Dai L, Wang SQ, Jiang SY, Sun LM, Niu WD, Chen XR. Surveillance results analysis of food–borne diseases in Zhengzhou City in 2017 (in Chinese). *Modern Prev Med* **2019**, *4*, 715–717. +759.
129. Dai YX, Su LY, Liu YS, Song TX, He YF, Ren Q, Chen H, Han DL, Yue Y. Analysis on four norovirus epidemic among primary schools and kindergartens (in Chinese). *Modern Prev Med* **2018**, *6*, 1127–1129.
130. Deng AP, Sun LM, Mo YL, Fang L, Kang M, Tan XH, Li H, Lin JY. Etiological characteristics of viral diarrhea in Guangdong Province, 2012 (in Chinese). *South China J Prev Med* **2014**, *2*, 119–122.
131. Deng JK, Wang RL, Luo X, Guo YH, Yu CH, Lan M, Che XY, Ding XX. Distribution and genotype of diarrhea causing viruses in children and adult patients in Guangzhou (in Chinese). *Modern Prev Med* **2015**, *13*, 2393–2396. +2400.
132. Deng L. Analysis of pathogenic prevalence in children with acute viral diarrhea (in Chinese). *Beijing Med J* **2015**, *6*, 521–523.
133. Deng XC, Tan XL, Xu ZL, He JZ, Tan WF, Yang ZX, Liang ZL, Lin YF, Huang ML. Investigation on a diarrhea outbreak caused by mixed infection of norovirus and enteroaggregative escherichia coli in Gangbei County of Guigang City (in Chinese). *Occup & Health* **2019**, *17*, 2377–2380. +2385.
134. Deng Y, Gao ZY, Yan HQ, Li XT, Li WH, Li J, Jia L, Wang QY. Investigation of human calicivirus infections for diarrhea patients visiting enteric clinics in Beijing (in Chinese). *Int J Viro* **2012**, *5*, 193–197.
135. Deng YH, Ke XL, Jin F. Investigation of norovirus gastroenteritis outbreak in a primary school in Tianhe District, Guangzhou (in Chinese). *Int Med & Health Guid News* **2014**, *22*, 3503–3505.
136. Zhai GF, Feng YQ, Zhao CX. Epidemiological characteristics of foodborne diseases in Maanshan city (in Chinese). *Anhui Med J* **2019**, *10*, 1176–1180.
137. Zhai QQ, Fang CG, Bai GD. Surveillance for viral diarrhea in Jilin, 2013–2015 (in Chinese). *Dis Surveil* **2016**, *10*, 843–846.
138. Ding JQ, Chen Q, Zhou P, Xie XH, Sun ML. Epidemiological investigation on an outbreak of infectious diarrhea caused by Norovirus in the hotel (in Chinese). *Occup & Health* **2012**, *22*, 2794–2795.
139. Ding M, Liu CL, Pan LF, Lu L, Qiu HF, Liu Q, Zhu LY, Ma P, Hao LP, Fu YF. Detection and analysis of viral diarrhea in Shanghai Pudong New District from 2012 to 2013 (in Chinese). *J Navy Med* **2016**, *3*, 233–236.
140. Ding YF. Analysis of norovirus type I and type II infection in children with acute diarrhea (in Chinese). *Prev Med* **2013**, *5*, 74. +79.
141. Dong GL, Chen SM, Yan XM, Yang TF. Investigation of a norovirus infection outbreak in a university (in Chinese). *Chin J School Doct* **2018**, *6*, 441. +443.
142. Dong HG, Gao YH, Yan LQ. Analysis of the surveillance results of foodborne diseases in general hospitals (in Chinese). *J Taishan Med Coll* **2017**, *1*, 44–45.
143. Dong HY, Ma ZL, Yang JG. Aetiological study on viral pathogens of diarrhea patients from Taizhou in 2013 (in Chinese). *Modern Prev Med* **2015**, *1*, 134–136.
144. Dong HY, Ma ZL, Yang MD, Ding W. Etiological study on viral pathogens of diarrhea patients in Taizhou during 2013–2015 (in Chinese). *Chin J Health Lab Tech* **2017**, *5*, 728–730.
145. Dong JH. Correlation between serum CK, CK–MB and acidosis in children with viral diarrhea (in Chinese). *Lab Med & Clin* **2019**, *19*, 2868–2871.
146. Dong SC, Li YM, Wang XG, Huang L. Investigation on an outbreak of acute gastroenteritis of water–borne norovirus in rural areas (in Chinese). *Prev Med* **2017**, *4*, 365–367.
147. Dong ZJ, Gong LF, Xu YC, Sun ZL, Mou XD, Xu JJ. Analysis of pathogenic monitoring of virus diarrhea in Yantai in 2017 (in Chinese). *Chin J Health Lab Tech* **2020**, *2*, 245–246. +251.
148. Du YH, Zhang XJ, Sun LL. Investigation on an outbreak of GII norovirus in Lianyungang City in 2018 (in Chinese). *Jiangsu J Prev Med* **2019**, *4*, 443–444.
149. Du ZY. Analysis of virus infection in children with diarrhea in Chaoyang area (in Chinese). *China Med Eng* **2014**, *1*, 181. +184.
150. Du Y, Wu YF, Chen YT, Cui JL, Xie DS, Huang JF, Huang HB, Liu CX, Ma J. Clinical and etiological analysis of 708 diarrheal cases (in Chinese). *Chin J Infect Dis* **2013**, *12*, 740–743.
151. Duan JJ, Yao Z, Li XH, Han TW, Li XQ, Xu LY, Shen XJ, Zhao JY. Pathogen spectrum study on viral diarrhea in children under the age of 5 in Zhengzhou City, 2011–2016 (in Chinese). *Modern Prev Med* **2018**, *5*, 828–831. +838.
152. Duan R, Liu JY, Shen H, Zhang JJ, Zhang CH. Analysis on epidemiological and aetiological characteristics of aggregation epidemic induced by norovirus infection in Xuhui District of Shanghai from 2013–2015 (in Chinese). *Occup & Health* **2017**, *1*, 63–65. +70.
153. E JW, Wu LL, Zhao LM, Zhu LH. Common Virus Monitoring and the Analysis of Disease Burdenin the Diarrhea Patients (in Chinese). *Labeled Immunoassays & Clin Med* **2014**, *3*, 276–279.
154. Fan CM, Lin SX, Li X, Wang W, Fang YL, Peng L. Detection and Clinical Characteristics of Clostridium Difficile Infection in Children with Acute Diarrhea (in Chinese). *Shandong Med J* **2019**, *16*, 63–66.

155. Fang YL, Wang W, Wang P, Zhao Y, Zhang TQ, Li X, Fan CM, Lin SX. Molecular epidemiology of norovirus in children with acute gastroenteritis in Tianjin, China (in Chinese). *Chin J Contemp Pediatr* **2019**, *2*, 139–143.
156. Fang YL, Zhang TQ, Wang W, Hou MZ, Wang L, Wu JY, Lin SX. Molecular epidemiological characteristics of norovirus in hospitalized children with sporadic acute gastroenteritis in Tianjin in 2019 (in Chinese). *Chin J Microbiol & Immunol* **2021**, *2*, 126–132.
157. Fang YX, Wang JZ. A Norovirus Outbreak Occurred in a Kindergarten of Tianjin (in Chinese). *J Prev Med Inform* **2018**, *7*, 1001–1002.
158. Fei Y. Investigation of two clustered outbreaks of norovirus diarrhea in households (in Chinese). *Shanghai J Prev Med* **2011**, *6*, 269–270.
159. Fei Y, Sun Q, Fu YF, Zhu WP, Xue CY. Surveillance and analysis on diarrheal disease pathogen spectrum among children under 5 years old in Pudong New Area of Shanghai City (in Chinese). *Shanghai J Prev Med* **2013**, *11*, 602–605.
160. Feng WH, Xiao Y. Etiological analysis of a GII norovirus (NoV) coinfection outbreak (in Chinese). *Chin J Health Lab Tech* **2014**, *11*, 1573–1575. +1578.
161. Feng Z, Feng JY, Jing ZM, Xia GJ, Li Y, Wang J. Investigation of an outbreak of gastroenteritis in VIP district of airport (in Chinese). *Chin Community Doctors* **2020**, *36*, 159–160.
162. Feng Z, Qi ML. Investigation on an outbreak of norovirus gastroenteritis in a primary school of Nanjing city (in Chinese). *Chin J Hyg Rescue (Electron E)* **2018**, *6*, 369–374.
163. Fu JG. Investigation of an acute gastroenteritis outbreak in Jiangsu province (in Chinese). *Chin J Zoonoses* **2013**, *5*, 509–512.
164. Fu JG, Shi C, Sha D, Shi P, Bao CJ, Ai J. Genetic characterization of norovirus isolated in an outbreak of gastroenteritis in Jiangsu province (in Chinese). *Chin J Epidemiol* **2018**, *1*, 72–74.
165. Fu JG, Wu B, Ji H, Li L, Qi X, Qin YF, Wang SJ, Deng F, Li ZF, Shang FY, et al. Molecular epidemiology of noroviruses from infantile diarrhea at Suzhou and Nanjing area in JiangSu province in 2010 (in Chinese). *Chin J Microbiol & Immunol* **2011**, *11*, 989–993.
166. Fu Y, Hu JM, Zhang P. Analysis of monitoring results in sentinel hospitals for diarrheal diseases in Huzhou City in 2013 (in Chinese). *Prev Med* **2015**, *9*, 931–932. +934.
167. Fu Y, Yuan R, Song ZP. Epidemiological distribution characteristics of foodborne diseases in Huzhou during 2016–2017 (in Chinese). *Chin J Health Lab Tech* **2019**, *20*, 2537–2540.
168. Fu L. Prevalence and Clinical Characteristics of Norovirus in Patients with Diarrhea (in Chinese). *J Beihua Univ (Nat Sci)* **2015**, *6*, 770–773.
169. Fu YL. Analysis on monitoring results of foodborne diseases in sentinel hospital in northern Nanjing from 2013–2014 (in Chinese). *Chin J Food Hyg* **2017**, *1*, 93–96.
170. Fu YF, Sun Q, Zhu WP, Zhu LY, Fei Y, Ye CC, Xue CY, Wang WQ, Pan LF. Analysis of pathogen spectrum surveillance of diarrheal patients in Pudong New Area, Shanghai, 2011 (in Chinese). *Chin J Dis Control & Prev* **2013**, *3*, 246–250.
171. Fu XF, Lin Y, Zhou WL, Li YF, Gao L. Investigation on a clustered outbreak of norovirus gastroenteritis (in Chinese). *Chin Rural Health Service Adm* **2012**, *1*, 68–70.
172. Gan XY, Huang HK, Zheng H, Huang J, Liang FL, Du HJ. Epidemiological investigation of an outbreak of infectious diarrhea caused by norovirus (1) (in Chinese). *J Front Med* **2014**, *16*, 362–363.
173. Gao GL, Wu YL, Xi C, Zhang QH. Analysis on the surveillance results of infectious diarrhea pathogens in Songjiang District, Shanghai in 2013 (in Chinese). *J Med Inform* **2015**, *20*, 198–199.
174. Gao HM, Huang SW, Dai ZY. Investigation on the cluster of norovirus infection in a kindergarten (in Chinese). *Prev Med* **2014**, *4*, 397–399.
175. Gao HM, Wu LZ, Ma Y. Pathogen monitoring and analysis of foodborne disease in Qingpu District of Shanghai from 2014–2018 (in Chinese). *Occup & Health* **2020**, *5*, 608–612. +617.
176. Gao HJ, He F, Guo MJ, Cao Y, Zhang XY. Investigation of an outbreak of acute gastroenteritis caused by norovirus (in Chinese). *Prev Med* **2015**, *7*, 721–723.
177. Gao JY, Yang X, Wang XY, Song Y, Li LZ, Wang J. Investigation and analysis of clustered epidemic situation of norovirus infectious diarrhea (in Chinese). *Parasitol & Infect Dis* **2017**, *3*, 173–175.
178. Gao L, Wang HH, Gao WJ, Chen LX, Li P. Pathogens of foodborne diseases in Jiaxing (in Chinese). *Prev Med* **2021**, *1*, 75–77. +80.
179. Gao L, Li JM. Pathogenic spectrum and epidemiological characteristics of diarrhea patients in Tianjin during 2015 – 2017 (in Chinese). *Chin J Infect Dis* **2019**, *4*, 209–213.
180. Gao L, Li JM. Pathogenic spectrum and clinical characteristics of 455 children under age of 5 with diarrhea in Tianjin, 2015–2017 (in Chinese). *China Trop Med* **2019**, *12*, 1149–1154.
181. Gao P, Lv JC, Chen DW, Liu XF, He C, Zhen GX. Analysis of an outbreak of gastroenteritis caused by norovirus in a study tour of Beijing (in Chinese). *Capital J Public Health* **2020**, *2*, 94–96.
182. Gao RH, Liu LD, Wang W. survey on a norovirus outbreak (in Chinese). *Prev Med Tribune* **2019**, *4*, 309–311.
183. Gao SP, Long YF, Zhang M, Lin YY, Zhu HC, Wu MJ, Wang H. An outbreak of norovirus infectious diarrhea caused by bottled water contamination in Heyuan (in Chinese). *South China J Prev Med* **2019**, *3*, 249–252. +293.
184. Gao X, Yang CJ, Tian JH, Zhang HC. Analysis of pathogen monitoring results of infectious diarrhea in Beijing Traditional Chinese Medicine Hospital Affiliated to Capital Medical University from 2016 to 2019 (in Chinese). *Chin J Prev Med* **2021**, *7*, 879–883.

185. Gao X, Tu Y, Wang YM, Yang ZL, Zhao LR, Yang HM, Liang XP, Zhang YL, Li RF, Mei L. The significance of etiological analysis and cytokine detection of benign infantile convulsions associated with mild gastroenteritis (in Chinese). *Chin Pract Med* **2020**, *24*, 197–199.
186. Gao ZP, Zhao XJ, Li C. Disinfection of the foci of an outbreak caused by norovirus (in Chinese). *Chin J Disinfect* **2018**, *6*, 469–470.
187. Gao ZY, Liu BW, Hou LY, Yan HQ, Tian Y, Chen YW, Zhang XX, Zhang Y, Jia L, Qian HK, et al. Analysis of infection characteristics of patients in norovirus acute gastroenteritis epidemic (in Chinese). *Chin J Exp & Clin Virol* **2017**, *1*, 38–41.
188. Ge B, Li S. Study on the test value of virus infection in children with diarrhea in Qichun area (in Chinese). *Int J Lab Med* **2018**, *4*, 483–485.
189. Ge HX. Pathogenic Analysis on Viral Diarrhea of the Children in Hospital that Under 5 Years in Suzhou from 2013 to 2017 (in Chinese). *Chin J Hemorheol* **2018**, *2*, 199–202.
190. Geng Q, Shi P, Ji L, Shi C, Chen SH, Qian YH. Monitoring system of viral diarrhea in Wuxi, 2013–2015 (in Chinese). *Modern Prev Med* **2017**, *5*, 903–906.
191. Gong CH, Liu F, Chen M, Sun JP, Ding GD. Surveillance and analysis on pathogen spectrum of diarrhea among children in Sanlin Area, Shanghai, 2015–2016 (in Chinese). *Pract Prev Med* **2018**, *7*, 886–889.
192. Gong LQ, Qian JQ, Ren YH, Zhang MW. Investigation on Outbreak of Norovirus Type II Infectious Diarrhea in Middle Schools (in Chinese). *Chin J School Health* **2013**, *9*, 1140–1141.
193. Gong M, Yu XL, Chen XF, Mao ZM, Shao LZ. Surveillance results analysis of viral diarrhea in children under 5–year–old in Quzhou, 2016 (in Chinese). *Chin J Health Lab Tech* **2018**, *22*, 2779–2781.
194. Gong SY, Zhu ZH, Dong XJ, Weng ZJ. A school norovirus outbreak investigation (in Chinese). *Chin Rural Health Service Adm* **2017**, *9*, 1069–1070.
195. Gu KC, Chang ZR, Yao XY, Wei T. Investigation of an incident of foodborne illness caused by Norovirus type II (in Chinese). *Our Health* **2020**, *3*, 227.
196. Gu SP, Zhang H, Hu XG, Zhang XQ, You JC, Huang Y. Outbreak of drinking water type gastroenteritis caused by mixed infection of norovirus GGI and GGII (in Chinese). *Chin J Epidemiol* **2009**, *10*, 1033.
197. Gu YF, Mao JY, Xu YQ. Epidemiological characteristics of a clustered epidemic caused by norovirus infection in a school (in Chinese). *Renowned Doct* **2020**, *10*, 61–62.
198. Guan HX, Xiao Y, Sha D. Etiological analysis of infectious diarrhea in Wuxi, 2014 (in Chinese). *Lab Med* **2016**, *11*, 953–958.
199. Guan HX, Xiao Y, Sha D, Kan B, Feng WH. Analysis of surveillance results of viral diarrhea in Wuxi (in Chinese). *Chin J Health Lab Tech* **2014**, *9*, 1306–1308.
200. Gui GP, Wei CF, Cha RS. Investigation on an outbreak of norovirus acute gastroenteritis in a primary school (in Chinese). *Jiangsu J Prev Med* **2018**, *3*, 304–305. +307.
201. Guo BF, Jiang Y, Jiang X, Du XF, Ye YH, Wang W, Xie GX. Epidemiological characteristics of foodborne diseases in sentinel hospitals of Nanjing City from 2014–2015 (in Chinese). *Occup & Health* **2016**, *21*, 2939–2942.
202. Guo JX, Zhang HY, Wu W, Huang H, Li XT, Jia L, Gao ZY, Liu BW, Xu WC, Wang J, et al. Characteristics analysis of norovirus outbreak in Beijing Dongcheng District, 2014 (in Chinese). *Modern Prev Med* **2015**, *23*, 4371–4373.
203. Guo J, Liu Y, Sun M, Xu LF. Analysis of clinical characteristics of norovirus infection in children (in Chinese). *Clin J Med ficers* **2019**, *2*, 161–163.
204. Guo J, Yuan S, Zou L, Zhu YQ. Analysis on pathogenic results of active surveillance for foodborne diseases in Yangzhou City in 2019 (in Chinese). *Occup & Health* **2021**, *8*, 1056–1060.
205. Guo L, Hua WY, Liu F, Zhao Z, He B. Investigation into outbreak of norovirus infection caused by meal delivery by catering company (in Chinese). *Prev Med* **2019**, *11*, 1159–1161. +1164.
206. Guo L, Hua WY, Xing Y, Liu F, Shao YP, Chen CZ, Zhao YH, Jin LN, Wei YY, Jiao Y, et al. Epidemiological characteristics of clustered epidemic situation of norovirus infection in Haidian District of Beijing in 2017 (in Chinese). *Occup & Health* **2019**, *2*, 207–212. +217.
207. Guo LM, Guo S, Liu L, Cao XQ, Yuan Y, Mao JN. Monitoring and analysis of pathogenic microorganisms in 463 patients with diarrhea (in Chinese). *J Pathog Bio* **2018**, *5*, 536–538. +542.
208. Guo MJ, Yan ZL, Wang GH. Analysis of the epidemic character of norovirus gastroenteritis in Tongxiang (in Chinese). *Chin J Health Lab Tech* **2017**, *19*, 2870–2871. +2875.
209. Guo SL. Nursing intervention research on norovirus diarrhea in neonatal room (in Chinese). *Modern Diagn & Treat* **2014**, *23*, 5504–5505.
210. Guo XH, Li HJ, Tian J, Dong RQ, Shi L, Li LL. The Intestinal outpatient cases in Fangshan District of Beijing, 2013–2015 (in Chinese). *Modern Prev Med* **2017**, *7*, 1316–1318. +1344.
211. Han DJ, Xiang Y, Ai HW, Liu YY, Liu F, Wu JG, Wu KL. Etiological detection of viral diarrhea in Hubei and Jiangxi provinces from 2009 to 2015 (in Chinese). *J Front Med* **2016**, *30*, 354–356.
212. Han GQ, Cai CA, Yang XY, Chen LY. Clinical characteristics analysis of 69 children infected with norovirus (in Chinese). *Clin Med* **2015**, *10*, 15–16.
213. Han N, Wang DL, Zhang HL, Yao XJ, Shu BH, He YQ. Surveillance and analysis of noroviruses in water environment and human population in Shenzhen (in Chinese). *Microbiol China* **2016**, *10*, 2166–2171.
214. Han YF, Gao J, Wei JJ, Deng T, Zhang JD, Shang WJ, He SJ. Surveillance results of rotavirus and norovirus diarrhea in Hanzhong, Shaanxi (in Chinese). *China Trop Med* **2020**, *8*, 731–734. +742.
215. Hao SX, Xu H, Song YS, Xue Y, Xu JF. Analysis on Norovirus infection and evolutionary recombination in 832 diarrhea samples of Zhenjiang area in 2015 (in Chinese). *Modern Prev Med* **2017**, *22*, 4181–4185.

216. Hao YJ, Gu LQ, Chen C, Shi SG, Liu B, Jiang ZT. Survey and analysis of epidemiology of 1 case of Norovirus outbreak (in Chinese). *Med J National Defending Forces Southwest China* **2020**, *3*, 279–281.
217. He CJ, Hu GF. Etiological characteristics and epidemiological characteristics of infectious diarrhea in Zhaoqing from 2015 to 2017 (in Chinese). *J Trop Med* **2019**, *2*, 240–243.
218. He LX. Status of norovirus infection in infectious diarrhea cases in Yangshan County, 2014–2016 (in Chinese). *South China J Prev Med* **2018**, *2*, 154–156.
219. He LY, Zhou YM, Gu WP, Xu WF, Zhang SX, Ai L, Lu Y, Tian LG. Molecular epidemiological characteristics of Human Calicivirus in Kunming city, 2014–2015 (in Chinese). *Chin J Exp & Clin Virol* **2017**, *6*, 525–529.
220. He MT, Wu X. Analysis and discussion on the results of routine clinical examination of stool in patients with diarrhea (in Chinese). *Gems Health* **2020**, *35*, 2.
221. He QY, Chen ZQ, Zou YW. Epidemiological features of norovirus and rotavirus diarrhea in Meizhou from 2012 to 2017 (in Chinese). *Capital J Public Health* **2019**, *1*, 48–50.
222. He XX, Wei XM, Wang JL, Chen YF. Investigation and analysis of an outbreak of norovirus diarrhea (in Chinese). *J Med Pest Control* **2017**, *6*, 698–699.
223. He FQ, Cao ZA, Luo YH. Analysis of bacterial and viral pathogenic surveillance results of infectious diarrhea in Zhuzhou City from May 2015 to April 2016 (in Chinese). *Henan J Prev Med* **2017**, *9*, 691–692. +694.
224. He HZ, Xu SM. Investigation on an incident of infectious diarrhea caused by norovirus in a middle school in a county (in Chinese). *China Health Care & Nutr* **2014**, *2*, 771.
225. Hong CJ, Li Y, Sun BC, Lin D, Shang GZH. An analysis on the distribution and drug resistance of pathogenic bacteria in infectious diarrhea among infants (in Chinese). *Prev Med* **2016**, *5*, 453–457.
226. Hong WS, Liao XW, Liu MZ, Lin F, Wu JG, Chen JB, Chen ZX, Wu WQ. Surveillance results of pathogens of viral diarrhea in infants and young children in Ruian City (in Chinese). *Prev Med* **2015**, *1*, 65–67.
227. Hong WS, Liao XW, Liu MZ, Lin F, Wu JG, Chen JB, Chen ZX, Wu WQ. Analysis of the surveillance results of viral diarrhea etiology in the city from 2012 to 2013 (in Chinese). *Chin J Rural Medicine & Pharm* **2015**, *1*, 65–66.
228. Hong Y. Molecular epidemiological analysis of Norovirus in patients with foodborne diseases in sentinel hospital, Ma'anshan City, Anhui Province (in Chinese). *Chin J Prev Med* **2019**, *6*, 581–585.
229. Hong ZT, Xu L, Zhong HL, Zhang SF, Luo HJ, Tuo JL, Zhang T, Cao KY. Etiological study on diarrhea virus in Guangzhou area from 2011 to 2013 (in Chinese). *J Trop Med* **2015**, *12*, 1679–1682.
230. Hou W, Du H, Shao B, Xue XN. Disposal of the first imported mass Norovirus infection at Shandong port (in Chinese). *Chin J Front Health & Quarantine* **2018**, *5*, 377–378.
231. Hou YY, Liu SW, Liu ZX, Chen P. Investigation on an outbreak of norovirus infectious diarrhea in You County (in Chinese). *Pract Prev Med* **2014**, *10*, 1212–1214.
232. Hu GY, Zhang WZ, Wu D, Jing HB. Investigation on an outbreak of acute gastroenteritis in a kindergarten (in Chinese). *Prev Med* **2017**, *4*, 390–392.
233. Hu HA, Zhang YB, Luo JH, Jiang CH, Lu Y, Gong Q, Chen Y, Hu SX, Luo KW. An outbreak of infectious diarrhea with noroviruses caused by poor toilet hygiene (in Chinese). *Pract Prev Med* **2015**, *9*, 1061–1063.
234. Hu H, Liu WC, Lu WC. Analysis of detection results of virus antigen from the faeces of 700 adult patients with acute diarrhoea (in Chinese). *J Trop Med* **2018**, *10*, 1355–1357.
235. Hu J. Analysis of the results of active surveillance of foodborne diseases in sentinel hospitals in Yixing City from 2016 to 2018 (in Chinese). *Jiangsu J Prev Med* **2019**, *5*, 555–564.
236. Hu JM, Xue T, Dong X, Shen ZH, Ji L, Zhang Y, Wen XH. Status and characteristics of norovirus infection in foodborne diarrhea cases in Huzhou city (in Chinese). *Prev Med* **2014**, *12*, 1226–1228.
237. Hu TT, Ji XF, Chen HF, Yao YX, Luo MH, Li MQ, Huang YH, Luo C, Chen Q. Epidemic characterization of viral diarrhea among migrant children in Guangzhou during 2010 to 2011 (in Chinese). *Chin J Dis Control & Prev* **2013**, *4*, 359–361.
238. Hu TT, Luo MH, Ji XF, Chen HF, Yao YX, Huang YH, Chen Q, Yu SY. Molecular epidemiological studies of the viral diarrhea in adults of Guangzhou (in Chinese). *J Trop Med* **2013**, *1*, 20–23.
239. Hu Y. Analysis of an outbreak of gastroenteritis caused by norovirus in a primary school in Jiangsu (in Chinese). *Our Health* **2019**, *18*, 130.
240. Hu Z, Shi WS, Liu YL, Fang Y. Analysis of sporadic status of foodborne diseases in Zhuzhou City in 2016 (in Chinese). *Studies Trace Elements & Health* **2017**, *6*, 41–42.
241. Hua WY, Chen CZ, Liang JB, Liu F, Shao YP, Guo L, Song W. Infectious Diarrhea of Norovirus Outbreak in A Primary School in Haidian District of Beijfing in 2017 (in Chinese). *Parasitol & Infect Dis* **2018**, *3*, 143–146.
242. Hua WY, Liu F. Investigation of Clustering Epidemics of Infectious Diarrhea Caused by Norovirus in Haidian District of Beijing from 2014 to 2015 (in Chinese). *Parasitol & Infect Dis* **2018**, *2*, 89–91.
243. Hua WY, Zhao Z, Li Y, Shao YP, Guo L. Surveillance for other infectious diarrheal diseases in Haidian district of Beijing, 2011–2015 (in Chinese). *Dis Surveil* **2017**, *7*, 577–581.
244. Huang AX, Shen YQ, Wu Y, Cao LL. Analysis of laboratory test results and drug resistance monitoring results of foodborne diseases in Wuwei City (in Chinese). *Chin J Health Lab Tech* **2020**, *2*, 235–238.
245. Huang BB, Zan W, Liu MY, Liu XB. Investigation on the Outbreak of Norovirus in A Boarding Middle School in Chengdu (in Chinese). *J Prev Med Inform* **2020**, *7*, 822–826.
246. Huang CL, Li CJ, Tan ZJ. Epidemiological investigation of a norovirus infections diarrhea outbreak (in Chinese). *J Med Pest Control* **2015**, *3*, 303–306.

247. Huang CM, Deng Y, Li DM, Wu NW. Investigation on the outbreak of infectious diarrhea in multiple tourist groups caused by a norovirus in a city (in Chinese). *Int J Lab Med* **2016**, *5*, 719–720.
248. Huang DZ. Investigation report on successful handling of a suspected food poisoning incident (in Chinese). *China Food Safety Magazine* **2017***NA*, 47.
249. Huang EM, Chen XY, Yao ZF. Epidemic characteristics of other infectious diarrhea in Zhongshan city from 2013 to 2017 (in Chinese). *J Trop Med* **2019**, *6*, 793–795. +804.
250. Huang FG, Gao SP, Yue YH, Zhang XL. Infectious diarrhea symptoms and etiological surveillance in Heyuan City from 2015 to 2017 (in Chinese). *Chin J School Doct* **2018**, *7*, 538–540.
251. Huang G, Li JM. Analysis on the outbreaks of norovirus infection diarrhea in Hechi City (in Chinese). *Modern Prev Med* **2013**, *11*, 2151–2153. +2158.
252. Huang G, Chen MY, Zhang YH, Li YP. Investigation of a gastroenteritis outbreak caused by norovirus GII in a school in Jiangmen, Guangdong (in Chinese). *J Trop Med* **2015**, *10*, 1432–1435.
253. Huang G, Zhang YH, Chen MY, Li YP. Investigation of a food–brone outbreak of an infectious diarrhea caused by Norovirus in students of a school (in Chinese). *China Trop Med* **2015**, *7*, 895–897.
254. Huang LQ, He XL, Lu WZ, Wu XF, Liu HL, Liu CS, Huang KY. An outbreak of acute gastroenteritis caused by a Norovirus contaminated water reservoir in a company, Nanning City (in Chinese). *Modern Prev Med* **2020**, *1*, 173–176.
255. Huang PF, Wu ZJ, Wu DK. Analysis of the etiological characteristics of viral diarrhea in Xiangxi from 2015 to 2016 (in Chinese). *J Modern Med & Health* **2018**, *4*, 630–631.
256. Huang ST. Investigation on an epidemic of norovirus infection in a middle school in Quzhou City (in Chinese). *Chin J Health Lab Tech* **2021**, *6*, 742–744.
257. Huang SY, He JF, Yi LP, Zong J, Xu M, Fu JJ. Outbreak investigation of acute gastroenteritis by secondary pollution in water supply, Pingxiang College, Jiangxi (in Chinese). *Modern Prev Med* **2020**, *3*, 542–545.
258. Huang TR, Lu YZ, Lv B, Feng XL, Feng XY. Clinical analysis of 48 cases of severe gastroenteritis with benign convulsions in infants and young children (in Chinese). *Shenzhen J Integr Tradit Chin & West Med* **2020**, *23*, 166–168.
259. Huang YH. Epidemiological characteristics of outbreaks of acute gastroenteritis in Xicheng District of Beijing from 2015–2018 (in Chinese). *Occup & Health* **2019**, *12*, 1669–1671.
260. Huang YH, Chu YH, Liu XX, Kong QZ. Survey on a school public health emergency event caused by Norovirus GII.2 infection in Beijing (in Chinese). *J Med Pest Control* **2019**, *6*, 591–593. +597.
261. Huang YH, Chu YH, Liu XX, Kong QZ. Investigation of acute gastroenteritis outbreak caused by norovirus in a boarding school of Beijing (in Chinese). *Capital J Public Health* **2019**, *2*, 87–90.
262. Huang Y, Chen YD, Li YF, Li JQ. Surveillance of viral diarrhea in Dongguan, 2010–2016 (in Chinese). *China Trop Med* **2018**, *2*, 143–146.
263. Huang ZX, Zhan JY, Wu SH, Cao R. Investigation of an incident of norovirus–infected diarrhea in a hospital (in Chinese). *Internal Med* **2013**, *2*, 181–182.
264. Ji H, Xiang LH, Zhang XF, Fu JG, Ai J, Dan J, Zhu YF, Shang FY, Xu JF, Chen WG, et al. An outbreak of Norovirus gastroenteritis in a primary school of Jiangsu Province (in Chinese). *Chin J School Health* **2015**, *5*, 721–724.
265. Ji JH, Wang ZC, Zhong YL. Investigation of an outbreak of Norovirus infection in a power plant in Changjiang County (in Chinese). *China Trop Med* **2018**, *5*, 512–515.
266. Ji YL, Wang YQ, Cui HY, Jin B, Cao W, Liu XX. Investigation on the epidemiological characteristics of viral diarrhea among children under 5 years in Beijing Xicheng District, 2015–2018 (in Chinese). *J Public Health & Prev Med* **2020**, *3*, 104–107.
267. Ji YL, Wang YQ, Yang QJ, Cui HY, Jin B, Cao W, Liu XX. Investigation on the infection status of the five diarrhea–related viruses among acute infantile diarrheacases in Beijing (in Chinese). *J Public Health & Prev Med* **2017**, *5*, 42–45.
268. Ji L, Chen LP, Shen YH, Yan W, Wu XF, Xu DS, Zhu XJ, Cha YF. Epidemiologic and genetic characteristics of norovirus caused acute gastroenteritis in Huzhou, Zhejiang, 2016 (in Chinese). *Dis Surveil* **2018**, *3*, 235–240.
269. Ji L, Liu GT, Shen YH, Cha YF, Xu DS. Genetic characteristics of norovirus GII.P7–GII.6 isolated from acute gastroenteritis cases in Huzhou of Zhejiang, 2013–2019 (in Chinese). *Dis Surveil* **2021**, *1*, 53–58.
270. Ji L, Liu GT, Liu BH, Wu XF, Xu DS, Chen LP. Molecular characterization of an acute gastroenteritis outbreak caused by mixed infection of GI and GII norovirus (in Chinese). *Chin J Health Lab Tech* **2020**, *13*, 1567–1570. +1573.
271. Ji L, Wu XF, Xu DS, Gong LM. Preliminary study on molecular biological characteristics of norovirus in outbreaks of nonbacterial acute gastroenteritis in Huzhou city (in Chinese). *Chin J Virol* **2011**, *5*, 469–474.
272. Ji L, Xu DS, Liu GT, Chen LP, Wu XF. Etiology and Genotype Features Analysis of Acute Gastroenteritis Outbreak Associated with Norovirus GII.P16–GII.2 (in Chinese). *Chin J Virol* **2018**, *2*, 159–164.
273. Ji L, Zhu YY, Chen LP, Shen YH, Wu XF, Xu DS. Infection status and genotype characteristics of GI norovirus in acute gastroenteritis cases in Huzhou, Zhejiang, 2017–2018 (in Chinese). *Dis Surveil* **2019**, *6*, 536–540.
274. Ji RP, Cai YL, Wu H. Analysis on the disposal of an outbreak of gastroenteritis caused by norovirus (in Chinese). *Jiangsu J Prev Med* **2020**, *6*, 648–649. +659.
275. Ji XF, Luo C, Hu TT, Luo MH, Li MQ, Liu Y, Chen Q, Yu SY. The surveillance of viral diarrhea among migrant children in Guangzhou (in Chinese). *J Trop Med* **2012**, *4*, 418–421.
276. Jia LP, Deng J, Qian Y. A GII.17 Norovirus–associated Acute Gastroenteritis Outbreak in a Tourist Group in Inner Mongolia, China (in Chinese). *Chin J Virol* **2017**, *5*, 767–773.
277. Jia LL, Chen YL, Wang YQ, Yang YS. Analysis on epidemiological characteristics of other infectious diarrhea in Miyun County of Beijing from 2009–2014 (in Chinese). *Occup & Health* **2016**, *11*, 1510–1512.

278. Jia N, Xie LJ, Suo JJ, Xing YB, Gao Y, He L, Liu YX. Molecular epidemiology of norovirus infection in patients with diarrhea (in Chinese). *Chin J Nosocomiol* **2012**, *3*, 536–538.
279. Jiang LL, Chen YQ, Wang XD, Yu SY. Surveillance results of foodborne disease in Jing'an District, Shanghai (2014–2017) (in Chinese). *J Public Health & Prev Med* **2018**, *3*, 101–103.
280. Jiang X, Du XF, Ye YH, Jin P, Wang W, Ding J, Jiang Y. Etiological surveillance of infectious diarrhea among children in Nanjing region, 2015–2016 (in Chinese). *Pract Prev Med* **2018**, *12*, 1508–1510.
281. Jiang ZL, Jiang CM, Li SY. Analysis of characteristics and clinical characteristics of 350 children with diarrhea virus infection (in Chinese). *Clin Focus* **2014**, *8*, 916–918.
282. Jiang HB, Meng TY, Wang CX, Li WM, Li X, Yang XD, Sun LW. Analysis of pathogenic spectrum and clinical characteristics of viral diarrhea in children (in Chinese). *Chin J Pract Pediatr* **2019**, *7*, 583–586.
283. Jiang XC, Zhao RF. Epidemiological analysis of three cases of norovirus infectious diarrhea in schools in Quzhou city (in Chinese). *Chin J School Health* **2014**, *6*, 920–921.
284. Jiang YC, Ma FF, Li C, Cai YB, Chen LL, Zhang J, Liu HH. Field epidemiologic survey of outbreak of norovirus infection in a middle school in Suzhou (in Chinese). *Dis Surveil* **2015**, *12*, 1070–1071.
285. Jiang C, Zhou JG, Zhang HJ, Zou GH, Fu JG, Xiao Y, Peng ZH. Investigation on a suspected foodborne norovirus subtype GII.17 outbreak (in Chinese). *Jiangsu J Prev Med* **2019**, *6*, 650–651. +707.
286. Jiang CM, Sun CW, Yu JX. Surveillance analysis of enterovirus in patients with infectious diarrhea in Xuzhou city from 2012 to 2013 (in Chinese). *Chin J School Doct* **2013**, *12*, 904–905.
287. Jiang CL, Hang H, Ai J, Wu QB, Zhang J, Gu PQ, Fu JG. Genotyping of 67 norovirus strains (in Chinese). *Chin J Infect Dis* **2016**, *5*, 280–284.
288. Jiang CL, Xu Y, Liu Y, Chen YJ. Analysis of rotavirus and norovirus infections among infants with diarrhea in an outpatient in Nanjing (in Chinese). *Int J Viro* **2018**, *6*, 392–396.
289. Jiang CL, Ye LL, Xu Y, Gu PQ, Zhang HY, Jin M, Ai J, Fu JG. Detection and genetic analysis of new GII.17 norovirus variant from infantile diarrhea in Nanjing (in Chinese). *Chin J Nosocomiol* **2016**, *20*, 4717–4720.
290. Jiang HJ, Fan M, Liu K, Xu XY, Yang T, Dian ZQ. Epidemiological characteristics and main pathogenic characteristics of viral diarrhea in infants and young children in Kunming (in Chinese). *Int J Lab Med* **2020**, *19*, 2323–2327.
291. Jiang L, Chang WB, Zhou T. Detection and analysis of an outbreak of gastroenteritis caused by norovirus infection in an elderly apartment (in Chinese). *China Health Industry* **2014**, *33*, 31–32.
292. Jiang WJ, Zhu Z, Zheng YF, Wang L. Surveillance analysis of foodborne diseases in Zhuzhou City in 2018 (in Chinese). *Jiangsu J Prev Med* **2019**, *4*, 452–454.
293. Jiang XH, Yang J. Survey on an outbreak of norovirus GII in a kindergarten (in Chinese). *Prev Med Tribune* **2019**, *2*, 142–144.
294. Jiang YM, Zhao Z, Wang XY. Investigation on a cluster outbreak of acute gastroenteritis caused by norovirus (in Chinese). *Jiangsu J Prev Med* **2017**, *6*, 681–682.
295. Jiang ZJ, Chen JK, Zhao DY. Analysis on common pathogens of viral diarrhea in children under 5 years old in Shaoxing city (in Chinese). *Prev Med* **2020**, *7*, 730–732.
296. Jiao Y, He FR, Gao Y, Qi X, Zhang SY, Gu L, Zhang YS, Zhao JH, Zhang Y, Ge S, et al. Molecular epidemiology of norovirus among infants with diarrhea in Chaoyang district Beijing from 2011 to 2017 (in Chinese). *Chin J Exp & Clin Virol* **2019**, *2*, 125–130.
297. Jin D, Sun CW. Analysis on Etiology of Infectious Diarrhea Cases from Xuzhou from 2013 to 2015 (in Chinese). *Chin Primary Health Care* **2017**, *2*, 56–57. +64.
298. Jin D, Guo BF, Sun GJ. Pathogens isolated form foodborne diseases cases in Nanjing city, 2018: analysis on active surveillance data (in Chinese). *Chin J Public Health* **2021**, *3*, 564–567.
299. Jin SY, Lin J, Xu XJ, Zhong DM. Genotypes and molecular characteristics of Norovirus from children with acute diarrhea in Hangzhou area from 2010 to 2011 (in Chinese). *Chin J Clin Lab Sci* **2012**, *11*, 924–926.
300. Jin SG. Molecular epidemiological investigation of norovirus acute gastroenteritis (in Chinese). *J Med Inform* **2015**, *42*, 249–249.
301. Jin XM, Chen XN, Zheng L, Jin LQ. Analysis of epidemic character and influence factors of foodborne diarrhea in Ouhai District in 2017 (in Chinese). *Chin J Health Lab Tech* **2018**, *24*, 3040–3043.
302. Jin XF, Zhang SS. Epidemiological investigation of norovirus infectious diarrhea in a kindergarten in Fengtai District, Beijing (in Chinese). *Modern Women (Front in Med)* **2015**, *4*, 357–358.
303. Kang Q, Chen JH, Wang P, Zhang HM, Wang XY, Zhang H, Yu DS. Molecular etiologic investigation of gastroenteritis outbreaks caused by Norovirus GI in two schools in Lanzhou, 2018 (in Chinese). *Dis Surveil* **2020**, *3*, 269–273.
304. Kang YJ. Analysis and Significance of Virus Test in Children with Diarrhea (1) (in Chinese). *J Med Theory & Pract* **2017**, *11*, 1676–1677.
305. Kong BL, Yang ZX, He DH, Kong QL, Deng XC, Wei G, Huang ZX. Etiological Analysis of Infectious Diarrhea Surveillance Cases in Guigang City in 2014 (in Chinese). *Appl Prev Med* **2015**, *4*, 253–254.
306. Zhang JM. Molecular epidemiological study on viral diarrhea among pediatric patients under five years old in Inner Mongolia Materal and Child Health Care Hospital from 2015 to 2016. Master, Inner Mongolia Medical University; 2017.
307. Kuang HC, Wei YH, Wang H, Ruan SR, Li FF, Liang GC. An investigation of an outbreak of acute gastroenteritis caused by norovirus (in Chinese). *Prev Med Tribune* **2016**, *4*, 281–283.
308. Lai SM, Zhan BD, Zhao SG. Investigation of an outbreak of foodborne norovirus infectious diarrhea on a university campus (in Chinese). *Chin J Health Lab Tech* **2014**, *19*, 2887–2888.

309. Lan YY, Qiu WH, Zhou LZ, Cao XM, Huang LM, Zhu WP. Pathogenic surveillance results of diarrhea cases in a tertiary hospital in Shanghai from 2015 to 2019 (in Chinese). *Hainan Med J* **2021**, *7*, 940–943.
310. Lei L, Chi J, Yu GQ, Xiao JH, Xiong TT, Zheng XC. Etiological analysis of infectious diarrhea among adults and children in Baoan District, Shenzhen City, 2012–2014 (in Chinese). *Pract Prev Med* **2016**, *8*, 934–937.
311. Lei YL, Wang XG, Ye BF, Qian WM, Chen XY. Analysis of genetic testing of norovirus outbreak in a school of Lishui (in Chinese). *Chin J Health Lab Tech* **2016**, *16*, 2355–2356. +2360.
312. Lei Y, Zhuang ZC, Tian H, Li XY. Molecular characteristics of norovirus in acute gastrointestinal illness, Tianjin, 2019 (in Chinese). *Dis Surveil* **2020**, *10*, 913–919.
313. Li J, Xiao WJ, Hu JY, Wu HY, Xue Y, Pan H, He Y. Bacteriological and virological surveillance for infectious diarrhea in Shanghai, May–December, 2012 (in Chinese). *Dis Surveil* **2014**, *11*, 880–883.
314. Li JQ, Huang Y, Yuan DK, Yang HK, Li YF, Chen YD, Zhou JM. Analysis of viral diarrhea pathogen in Dongguan city (in Chinese). *China Trop Med* **2013**, *1*, 15–18.
315. Li B, Wang XL. An investigation report on an outbreak of norovirus infectious diarrhea (in Chinese). *World Latest Med Inform* **2019**, *88*, 385–386.
316. Li B. An analysis detect by RT–PCR to an infectious diarrhea outbreak caused by Norwalk Virus (in Chinese). *Henan J Prev Med* **2013**, *3*, 207–209.
317. Li B, Chen H, Ju CY, Huang RM, Wang T. Surveillance of viral diarrhea in Nanshan District of Shenzhen City in 2012–2013 (in Chinese). *China Trop Med* **2014**, *7*, 873–875.
318. Li CY, Wang JL, Xie Y, Chu HN, Ceng Q, Zhao GL, Qi SX. An investigation for infectious diarrhea incident caused by norovirus (in Chinese). *Chin J Health Lab Tech* **2012**, *4*, 866–867.
319. Li CL. Epidemiological investigation report on gastrointestinal reactions caused by norovirus in Hezhan Kindergarten in Lanhot City (in Chinese). *World Latest Med Inform* **2020**, *22*, 152–153. +161.
320. Li DB, Yu DX, Yan HC, Zhou ZJ, Yin T, Liu YB. Epidemiological Investigation of an Outbreak of Infectious Diarrhea Caused by Norovirus (in Chinese). *J Prev Med Chin PLA* **2018**, *8*, 963–966.
321. Li HQ, Li R, Hao RE, Yao SX, Xu XB, Yang HX. Surveillance and trace research of foodborne multiple pathogen in Yangquan, Shangxi from 2015 to 2017 (in Chinese). *Chin J Health Lab Tech* **2019**, *10*, 1153–1156. +1160.
322. Li H, Dan FR, An J, Sun XY, Guo SY. Analysis on the Results of Detection of Norovirus in Diarrhea Case in Yingkou City From 2015 to 2017 (in Chinese). *Med Innov China* **2019**, *20*, 150–153.
323. Li JS, Cui HY, Qin D. Analysis of clinical and epidemiological features of Rotavirus and Norovirus infe4ction in adults with acute gastroenteritis in Xicheng District, Beijng (2011–2015) (in Chinese). *J Public Health & Prev Med* **2017**, *2*, 60–63.
324. Li JS, Li CY, Dong YH. Analysis on infectious disease aggregations and outbreaks in schools and kindergartens in Baizhifang Street of Beijing in 2017 (in Chinese). *Occup & Health* **2018**, *17*, 2403–2406.
325. Li JS, Pang JJ, Gao B, Cui HY. Analysis of etiology and epidemiology in 104 sporadic acute diarrhea cases (in Chinese). *Chin J Health Lab Tech* **2012**, *8*, 1970–1972. +1977.
326. Li JS, Qin D, Cui HY. Analysis on aggregations and outbreaks caused by norovirus in Xicheng District of Beijing in 2015 (in Chinese). *Occup & Health* **2016**, *22*, 3137–3139.
327. Li JY, Liu YB, Zhao YQ, Liu HS, Dong X. Monitoring results analysis of norovirus infected diarrhea in Shenyang from 2015 to 2017 (in Chinese). *Chin J Health Lab Tech* **2019**, *15*, 1884–1885. +1889.
328. Li JS, Deng AP, Mo YL, Li H, Sun LM. A Norovirus caused gastroenteritis outbreak in a nursing home in Guangdong (in Chinese). *Dis Surveil* **2015**, *3*, 242–244.
329. Li J, Liu RY, Zhou XY, Lv Q, Wang HR, Zhou LJ, Qing JF. Investigation of An Outbreak of Norovirus Infection in A School in Sichuan Province (in Chinese). *J Prev Med Inform* **2019**, *9*, 1048–1052.
330. Li J, Yang Y, Liang ZC, Gao ZY, Jia L, Liu BW, Chen LJ, Wang QY. Analysis of pathogenic composition and clinical characteristics of viral acute gastroenteritis in children under five years old in Beijing (in Chinese). *Chin J Prev Med* **2020**, *10*, 1104–1110.
331. Li J, Li GM, Zhang T, Xu JQ, Xing XS, Wu Y, Liu GP, Jiang XQ, Jiang JW, Zhan JB. Etiological analysis on viral diarrhea in Xiangyang of Hubei from 2012 to 2013 (in Chinese). *Chin Prev Med* **2015**, *4*, 245–251.
332. Li J, Xing Y, Zou WJ, Zhang T, Xu JQ. Etiological and epidemiological analysis of viral diarrhea surveillance in Hubei Province in 2017–2019 (in Chinese). *J Public Health & Prev Med* **2021**, *2*, 30–33.
333. Li J, Zhang T, Xu JQ, Zhan JB, Guan XH, Xing XS, Ma J, Qiu DZ, Huang J, Guo PG, et al. Epidemic characteristics and genotypes of norovirus in Hubei Province, 2015 (in Chinese). *Chin J Dis Control & Prev* **2017**, *4*, 349–352. +361.
334. Li J, Sun YQ, Qi SX, Chen SL, Yu QL, Wang YH, Xie Y. The etiological study on the outbreak of acute gastroenteritis in Chengde city of Hebei province (in Chinese). *Hebei Med J* **2012**, *6*, 817–818.
335. Li JX, Gu XL. Analysis of norovirus infection status in infectious diarrhea cases in Baiyin area (in Chinese). *Chin J Health Lab Tech* **2014**, *14*, 2011–2013.
336. Li L, He BB, Fang JG, Ma L. Investigation on the first outbreak of Norwalk virus gastroenteritis caused by water contamination (in Chinese). *Chin J Health Insp* **2011**, *3*, 274–279.
337. Li LY, Li JH. Analysis of laboratory test results for active surveillance of foodborne diseases in Yuci District, Jinzhong City in 2017 (in Chinese). *Clin Lab J (Electron E)* **2019**, *2*, 16–18.
338. Li M, Gao GL, Zhang QH, Han N, Guo XQ. Epidemiological characteristics of norovirus clusters and outbreaks in Songjiang district of Shanghai, 2018 (in Chinese). *Pract Prev Med* **2020**, *4*, 491–494.
339. Li P, Rong JR, Lu L, Qu YH, Chen Y. Surveillance data analysis of foodborne diseases in Yinzhou District (in Chinese). *Prev Med* **2020**, *1*, 77–79.

340. Li Q, Li Y, Wang T. Investigation on an Outbreak of Diarrhea Caused by Norovirus Infection in a Large Factory of Shenzhen (in Chinese). *Occup & Health* **2010**, *20*, 2352–2354.
341. Li RH. Epidemiological Characteristics of Food Borne Diseases in Jining City in 2018 (in Chinese). *J Insp & QUARA* **2019**, *3*, 65–66.
342. Li RF, Yu Q, Tu Y, Kang WB. Epidemiological characteristics of 202 pathogenic children with viral diarrhea (in Chinese). *China Health Care & Nutr* **2018**, *9*, 6–8.
343. Li RQ, Hua WY, Sun YM, Sun PY, Liu F, He B, Xu J. Etiology investigation and epidemiological analysis of viral diarrhea among adults in Haidian district, Beijing (in Chinese). *Modern Prev Med* **2014**, *23*, 4235–4237. +4251.
344. Li SC, Lv B, Wu Y, Guan XH. Investigation of a norovirus infection outbreak among students in a spring tour in Hubei province (in Chinese). *Int J Viro* **2020**, *3*, 252–255.
345. Li SC, Wu Y, Guan XH, Xing XS. A Norovirus outbreak of infection diarrhea caused by noodles at a middle school in Hubei Province (in Chinese). *Chin J Food Hyg* **2019**, *2*, 174–178.
346. Li SC, Wu Y, Guan XH, Xing XS, Wu Y. Investigation on an outbreak of norovirus infectious diarrhea in a middle school in northern province (in Chinese). *Appl Prev Med* **2018**, *4*, 318–320.
347. Li SE, Feng Q, Li XY, Xiao FJ, Sun YX, Yang C, Guo SZ, Cai XZ. Epidemiologic characteristics of norovirus outbreaks in Harbin, 2016–2017 (in Chinese). *Int J Viro* **2018**, *4*, 235–238.
348. Li SE, Sun YX, Zhou H, Yang C, Tang DZ, Feng Q, Guo SZ, Xiao FJ, Li XY. Investigation on a Norovirus Infectious Diarrhea Outbreak Through Contaminated Centralized Water Supply System (in Chinese). *China Health Standard Manage* **2018**, *9*, 15–18.
349. Li SE, Zhou H, Liu JR, Wang Y, Jin DL, Wang J, Yang C, Xiao FJ, Feng Q, Guo SZ. Investigation on an outbreak of acute norovirus gastroenteritis in a university (in Chinese). *Chin J Public Health Manage* **2018**, *4*, 567–569. +572.
350. Li SJ. Investigation on an outbreak of norovirus acute gastroenteritis in a primary school (1) (in Chinese). *Shanghai J Prev Med* **2016**, *3*, 185–186.
351. Li W, Qi X, Wu P, Fu JG, Xie GX, Zhang HY. The aged with diarrhea infected with Sapporo virus in Nanjing (in Chinese). *Chin J Gerontology* **2015**, *12*, 3359–3361.
352. Li XT. Epidemiological survey of an outbreak of acute gastroenteritis caused by norovirus GII. 7 in primary school in Beijing (in Chinese). *Dis Surveil* **2015**, *6*, 516–518.
353. Li X, Yang XD, Xu S, Wu DL, Sun LW, Shen B, Li J, Liu HM. Detection of Norovirus Genotype GII.4Sydney2012 Variant from Children under 5 Years of Age in Jilin Province, 2012–2013 (in Chinese). *Chin J Vaccines & Immuniz* **2015**, *3*, 283–288.
354. Li X, Yang XD, Xu S, Wu DL, Sun LW, Lu XR, Shen B, Liu HM, Li J. Epidemiological Characteristics and Genotype of Norovirus in Infants under 5 Years Old in Jilin Province in 2011 (in Chinese). *Chin J Vaccines & Immuniz* **2013**, *5*, 431–434.
355. Li X, Yang XD, Yang Y, Sun LW, Xu S, Wu DL. Genetic analysis of norovirus in infants and children in Jilin Province from 2014 to 2016 (in Chinese). *Chin J Exp & Clin Virol* **2019**, *3*, 297–302.
356. Li X, Yang Y, Xiao X, Diao BB, Lu XR, Wu DL, Fang CG. Epidemiology and genetic characteristics of norovirus among sporadic cases of adult diarrhea in Jilin province, 2015 (in Chinese). *Int J Viro* **2019**, *4*, 237–241.
357. Li XY, Li DD, Cheng WX, Xie GC, Gao XQ, Kong GP, Jin Y, Duan ZJ. Molecular and epidemiological study on among children under 5 years old in Nanjin (in Chinese). *Chin J Exp & Clin Virol* **2012**, *1*, 14–17.
358. Li XY, Bian C, Liu C, Yu HQ, Zhang PY. Epidemiologic features of summer viral diarrhea in 2010 in Qingdao (in Chinese). *Qilu Med J* **2012**, *1*, 43–45.
359. Li XF, Zhao XY, Yang J, Wen YQ, Wu CH. Investigation on An Outbreak of Norovirus Infection in A Restaurant in Chengdu (in Chinese). *J Prev Med Inform* **2018**, *12*, 1517–1520.
360. Li Y, Li X, Gao Q, Liu YM, Xu J. Pathogenic study on viral diarrhea of infants and young children in Shanghai (in Chinese). *Chin J Health Lab Tech* **2015**, *17*, 2838–2842.
361. Li Y, Chen YJ, Li W, Lu CH, Zhao WX, Ai HF. Survey on an outbreak of norovirus gastroenteritis in a school (in Chinese). *Prev Med Tribune* **2017**, *5*, 353–355.
362. Li YF, Li JQ, Yuan DK, Huang Y, Chen YD, Yang HK. Surveillance of viral diarrhea in Dongguan in 2012 (in Chinese). *China Trop Med* **2014**, *1*, 51–53. +68.
363. Li YH, Liu M, Zhao LT, Zheng L, Luan WW, Zhang YZ. Study on pathogen surveillance of adult foodborne diarrhea cases in Jinan in 2013–2019 (in Chinese). *J Public Health & Prev Med* **2021**, *4*, 40–44.
364. Li YY, Xu L, Xiao HC, Chen WW, Yan ZF, Sang F. Analysis of active monitoring results of foodborne diseases in Weihai from 2014 to 2016 (in Chinese). *Chin J Health Lab Tech* **2017**, *18*, 2693–2695. +2698.
365. Li YL, Xie HP, Chen C, Cao L, Huang J, Liu L, Wang DH, Xiao XC, Gu JQ. Investigation on the epidemic situation of type II norovirus infection among college students (in Chinese). *Chin J School Health* **2014**, *7*, 1082–1084.
366. Li YR, Gan XZ, Yang XB. Investigation and handling of an outbreak of acute gastroenteritis caused by norovirus in a primary school (in Chinese). *Appl Prev Med* **2015**, *4*, 251–252.
367. Li Z, Sun CD, Jiang J. Clinical analysis of sporadic norovirus diarrhea in adult community (in Chinese). *Chin J Nosocomiol* **2009**, *9*, 1099.
368. Li ZQ, Lu P, Liu B, Li GT. Investigation and analysis of norovirus outbreak caused by dinner together (in Chinese). *Health Horizon* **2020**, *14*, 259.
369. Liang CQ, Shang ML, Xie ZH. Distribution and seasonal prevalence of pathogens causing diarrhea in a hospital of Shenzhen (in Chinese). *Lab Med* **2020**, *9*, 868–871.
370. Liang JH, Zhu JP, Huang ML, Guan YR, Feng MC. Analysis of Norovirus Surveillance Results in Diarrhea Patients in Jiangmen City in 2013 (in Chinese). *China Med Eng* **2015**, *3*, 148–149.

371. Liang LR, Li YP, Zhao LF, Zhang XH, Guo XF, Wang JT. Pathogen characteristics from an active surveillance program on foodborne diseases in Taiyuan, 2013 to 2019 (in Chinese). *Chin Remedies & Clin* **2021**, *10*, 1654–1657.
372. Liang L, Wei JH, Deng LL, Ma YY, Wei YZ, Qin Y, Zhong G, Liu W. Analysis of norovirus and human astrovirus infection in children with diarrhea under 2 years old in a hospital in Nanning in 2013 (in Chinese). *Appl Prev Med* **2017**, *5*, 371–373.
373. Liang L, Ge RH. Pathogenic analysis of 1607 infants with diarrhea in a hospital during 2012–2013 (in Chinese). *J Modern Med & Health* **2014**, *14*, 2128–2129. +2141.
374. Liang P. Investigation on Norovirus Outbreak in a Kindergarten in Yantai Development Zone (in Chinese). *Med Industry* **2020**, *2*, 121.
375. Liang Q, Xu J, Shi X, Shu C, Cao B, Liu YF. Surveillance and Analysis of Viral Diarrhea in Heilongjiang Province from 2012 to 2015 (in Chinese). *Chin J Public Health Manage* **2017**, *5*, 730–732.
376. Liang RC, Shi XD. Epidemiological investigation of an outbreak of infectious diarrhea caused by Norovirus (in Chinese). *J Med Pest Control* **2017**, *5*, 539–541.
377. Liang XL, Ruan DP, Chen YM. Analysis of the etiological characteristics of viral diarrhea in infants and young children in Xinchang County, Zhejiang Province from 2010 to 2012 (in Chinese). *Chin Prev Med* **2015**, *10*, 811–813.
378. Liang Y, Du XL, Zhang MZ, Wei XP, Meng L, Guo WQ, Wei CY, Li RL. Analysis of surveillance results of foodborne disease etiology in a comprehensive hospital (in Chinese). *Chin J Public Health Manage* **2021**, *1*, 102–105.
379. Liang YJ. Analysis of an outbreak of infectious diarrhea caused by Norovirus in a middle school (in Chinese). *Int Med & Health Guid News* **2019**, *13*, 2118–2120.
380. Liao C, Cao YQ, Gao JM. Investigation and Analysis of a Norovirus Clustering Epidemic in A Vocational and Technical School in Chengdu (in Chinese). *Parasitol & Infect Dis* **2021**, *1*, 33–37.
381. Liao KC, Huang CJ, Tan JM. Investigation of a Norovirus Infection Outbreak in a Kindergarten (in Chinese). *Strait J Prev Med* **2018**, *1*, 84–86.
382. Liao Y, Chen JH, Liu ZY, Peng Z, Xing LL, Zhang AH, Zhu CM. The characterization of norovirus genotypes in children in Chongqing (in Chinese). *J Chongqing Med Univ* **2011**, *2*, 242–245.
383. Lin D, Wang LL, Shan RQ, Gao SH, Cai YY. Analysis on surveillance results of foodborne diseases from sentinel hospitals in Wenzhou in 2014 (in Chinese). *Chin J Food Hyg* **2016**, *3*, 378–381.
384. Lin J, Li WD, Lv HX, Liu FR. Investigation on an outbreak of norovirus in a school, Shenzhen city (in Chinese). *Prev Med Tribune* **2019**, *5*, 365–368.
385. Lin LQ. Analysis of the results of routine clinical examination of stool in patients with diarrhea (in Chinese). *Electron J Clin Med Literature* **2020**, *49*, 138. +140.
386. Lin LJ, Peng YB, Luo X, Chen MJ, Deng JK, Yu N. Distribution of enteropathogens detected by the Luminex xTAG Gastrointestinal Pathogen Panel and clinical characteristics of diarrhea patients in Guangzhou (in Chinese). *J Pract Med* **2016**, *22*, 3724–3727.
387. Lin L. Analysis of surveillance results of viral diarrhea among children under 5 years of age in Shandong province from 2012 to 2017 (in Chinese). *Chin J Exp & Clin Virol* **2019**, *5*, 463–467.
388. Lin QF, Lin XY. Investigation on a food poisoning infected with Staphylococcus aureus and Norovirus (in Chinese). *Shanghai J Prev Med* **2018**, *6*, 480–483.
389. Lin Q, Cheng WX, Jin Y, Li M, Lian M, Zhou JS, Lu F, Duan ZJ. Epidemiological characteristics of human caliciviruses in infants and young children with acute diarrhea in Nanjing in 2009–2010 (in Chinese). *J Clin Pediatr* **2012**, *10*, 924–927.
390. Lin Q, Chen C, Hu FF, Qi R, Qian J. Study on an outbreak of acute gastroenteritis doubtfully associated with enterovirus mixed infection (in Chinese). *Modern Prev Med* **2015**, *3*, 398–399. +422.
391. Lin QS, Wang YL, Wu HX, Liu JY. Investigation on an outbreak of norovirus gastroenteritis in a school in Jinjiang City (in Chinese). *Strait J Prev Med* **2016**, *2*, 84–85.
392. Lin S, Pan H, Xiao WJ, Gong XH, Kuang XZ, Teng Z, Zhang X, Wu HY. Epidemiologic characteristics of Norovirus among adult patients with infectious diarrhea, in Shanghai, 2013–2018 (in Chinese). *Chin J Epidemiol* **2019**, *8*, 883–888.
393. Lin YY, Xing ZW, Tan HQ. Pathogen detection of an acute gastroenteritis outbreak caused by Norovirus in a school (in Chinese). *Chin J School Doct* **2018**, *4*, 272–274.
394. Lin YX, Ruan F, Wei QD, Zhang LR. Molecular epidemiological study of norovirus infection in infants and young children in Zhuhai, 2011–2013 (in Chinese). *South China J Prev Med* **2016**, *4*, 344–347.
395. Liu BW, Chu YH, Qin D, Gao ZY, Jia L, Wang QY. Epidemiological investigation on an outbreak of acute gastroenteritis caused by Norovirus in Beijing (in Chinese). *Capital J Public Health* **2017**, *6*, 281–283.
396. Liu BW, Gao ZY, Jia L, Yan HQ, Wang QY. Epidemiological characteristics of acute gastroenteritis public health emergencies in Beijing, 2016 (in Chinese). *Capital J Public Health* **2017**, *2*, 65–68.
397. Liu BW, Gao ZY, Wang QY, He X, Jia L, Li XT, Qian HK, Huo D, Cao WH. Epidemiological and clinical features of norovirus infection among diarrhea patients from the enteric clinics in Beijing, 2013–2014 (in Chinese). *Chin J Epidemiol* **2015**, *4*, 383–386.
398. Liu B, Zhao XF, Xu M, Huang MP, Zheng ZL, Fu YQ, Ye W. Epidemiological analysis of norovirus gastroenteritis outbreaks (in Chinese). *Prev Med* **2015**, *12*, 1253–1255.
399. Liu CX. Epidemiological characteristics of norovirus infectious diarrhea in Jizhou District of Tianjin from 2017–2018 (in Chinese). *Occup & Health* **2019**, *19*, 2721–2723.
400. Liu C, Feng X, Shao DH, He ML, Liang GW. Epidemic Characteristics of Four Kinds of Viral Diarrhea in Children under 5 Years Old (in Chinese). *J Med Res* **2020**, *6*, 137–141. +147.
401. Liu DJ, Guo XB, Chen SY. Epidemiological analysis and research on pathogen spectrum among children of foodborne acute diarrhea in Qinghai Province during 2013–2016 (in Chinese). *Chin J Food Hyg* **2018**, *3*, 279–282.

402. Liu D. Epidemiological characteristics of norovirus infectious diarrhea in Pudong New Area of Shanghai in 2015–2019 (in Chinese). *J Public Health & Prev Med* **2021**, *3*, 54–57.
403. Liu D, Wang YP, Chen Z, Xu HM, Wu YM, Zhang H, Zhu WP, Hao LP. Influencing factors of a norovirus gastroenteritis outbreak in a primary school (in Chinese). *China Trop Med* **2019**, *5*, 494–497.
404. Liu DS, Song TZ, Zhang SL, Sheng H, Zhang TL. Epidemiological investigation on an outbreak of norovirus acute gastroenteritis in a school (in Chinese). *Chin J School Doct* **2019**, *4*, 255–257. +310.
405. Liu GD, Fan ZX, Xie F. Results of active surveillance for food–borne diseases in Zigong City from 2015–2016 (in Chinese). *Occup & Health* **2017**, *20*, 2792–2794. +2799.
406. Liu GH, He JF, He YQ, Peng CQ, Yu SY, Zhang Z, Zhou GH. Investigation on Infectious Diarrhea Cases Caused by Norovirus in Secondary Water Supply (in Chinese). *Modern Prev Med* **2014**, *6*, 991–993.
407. Liu GR, Sun W, Wang HX, Li HT. Active surveillance results of foodborne diseases in Changping District of Beijing from 2016–2017 (in Chinese). *Occup & Health* **2019**, *14*, 1918–1921.
408. Liu HB, Liu Y, Wang YX, Shi WF, Huang SP, Kan Z. Surveillance results and antibiotic susceptibility analysis of Campylobacter in Fangshan District of Beijing in 2018 (in Chinese). *Occup & Health* **2020**, *9*, 1197–1199. +1204.
409. Liu HZ, Cui C, Zhu WP, Fu HQ, Zhao B, Lv J. Bacterial and viral infectious diarrhea of kindergarten children in Pudong New Area, Shanghai (in Chinese). *Chin J Child Health Care* **2018**, *5*, 555–558.
410. Liu HH, Chen T, Tan JM, Nong H. Analysis of epidemic characteristics of norovirus clusters and outbreaks in Nanning City in 2018 (in Chinese). *Occup & Health* **2020**, *14*, 1987–1990.
411. Liu HL, Shang H, Xia Y, Liang QQ, Fang Y, Zhuang JL. A case–control investigation on Norovirus aggregation in school (in Chinese). *Shanghai J Prev Med* **2018**, *4*, 304–306.
412. Liu JZ, Du MY, Zhu XM, Han TT, Wei Y. Analysis of surveillance data of viral diarrhea in Dezhou City in 2018 (in Chinese). *J Jining Med Univ* **2020**, *3*, 161–164.
413. Liu JJ, Sun ZX, Hu JZ, Li ZP, Yang H. Investigation on the outbreak of norovirus infectious diarrhea from secondary water supply contamination in a hotel in Zhangjiajie city (in Chinese). *J Trop Dis & Parasitol* **2018**, *4*, 191–193.
414. Liu L, Liu CC, Yang PF, Zhang MH, Hou HY, Liu H, Liu Y, Zhu YF. Epidemiological analysis of norovirus infection in Huai'an city from 2013 to 2014 (in Chinese). *Jiangsu J Prev Med* **2015**, *5*, 76–77.
415. Liu KQ, Wu TS, Li Y, Xu ZH, Li Q, Zhang LJ. Field epidemiological research of a water–borne norovirus diarrhea outbreak (in Chinese). *South China J Prev Med* **2011**, *1*, 13–16.
416. Liu KQ, Wu TS, Yuan W, Ma ZC, Li Y. Study on risk factors of norovirus infectious diarrhea in Bao'an District, Shenzhen (in Chinese). *J Community Med* **2013**, *21*, 8–9.
417. Liu LX, Qian HF. Investigation and analysis of an outbreak of norovirus infectious diarrhea in a university (in Chinese). *World Latest Med Inform* **2019**, *18*, 208. +215.
418. Liu L, Bai GD, Xing Y, Sun PH, Zhai QQ, Lv Y. Analysis on the surveillance results of Foodborne diseases in Jilin Province in 2013 (in Chinese). *Pract Prev Med* **2015**, *7*, 886–888. +883.
419. Liu MF, Yuan D, Chen D, Wang HH. Analysis on Viral Pathogens for Diarrhea in Children Under Five Years Old in Yueyang (in Chinese). *Pract Prev Med* **2011**, *10*, 1879–1881.
420. Liu QL, Zhou Y, Guo JX. Epidemiological analysis of an outbreak of foodborne norovirus infectious diarrhea (in Chinese). *Strait J Prev Med* **2019**, *5*, 89–90.
421. Liu SK, Huang ML, Kong ZF, Ge BX, Hong YZ. Investigation of an outbreak of diarrhea caused by norovirus in a school (in Chinese). *Shanghai J Prev Med* **2012**, *8*, 421–423.
422. Liu SK, Zhang HB, Zhang F, Wang F, Hu LL, You LX. Investigation of an outbreak of norovirus diarrhea in a school (in Chinese). *Prev Med* **2016**, *9*, 930–932.
423. Liu T. A report on the improvement of disinfection and isolation measures for a clustered outbreak of vomiting and diarrhea caused by norovirus (in Chinese). *Chin J Disinfect* **2019**, *5*, 399–400.
424. Liu T, Song CL, Zhu JM, Du JR, Wang XK. Epidemiological analysis on the clusters of norovirus gastroenteritis in Jinshan District, 2014 (in Chinese). *Pract Prev Med* **2017**, *9*, 1048–1050.
425. Liu TT, Song ZZ. Analysis of surveillance results of foodborne diseases in Inner Mongolia Autonomous Region in 2016 (in Chinese). *Pract Prev Med* **2019**, *2*, 235–237.
426. Liu W, Bai J, Han XR, Han SY, Shi LF, Ji LL. Analysis on pathogen detection of foodborne diseases in Haidian District in Beijing, 2015–2019 (in Chinese). *Strait J Prev Med* **2021**, *3*, 54–56.
427. Liu WF, Yang WK, Ceng YD, Ruan QY, Cai JW, Wang DH. Analysis of epidemiological characteristics of diarrhea monitoring in Xiaolan Town in 2016 (in Chinese). *J Med Pest Control* **2018**, *8*, 760–763.
428. Liu WJ, Wu Q, Huang Y, Zhang XL, Xu C, Dong YY, Zhou Y, Xu Q. Molecular epidemiology of norovirus gastroenteritis outbreaks in Yangzhou city from 2015 to 2017 (in Chinese). *J Food Safety & Quality* **2018**, *4*, 729–732.
429. Liu WG, Xu CH, Liu AX, Lin D, Huang LL. Monitoring results analysis of foodborne pathogens in Lishui from 2014 to 2016 (in Chinese). *Chin J Health Lab Tech* **2017**, *20*, 3007–3009.
430. Liu XX, Chu YH, Sun XY, Qin D, Ren J. An epidemiological investigation on infectious diarrhea outbreak caused by norovirus (in Chinese). *Capital J Public Health* **2014**, *2*, 75–77.
431. Liu XF, Zhang Y, Zhang LR, Zhen BJ, Li CH, Wu YB, Gao ZY. Etiological analysis of diarrhea patients visiting enteric disease clinics in Beijing (in Chinese). *Chin J Infect Dis* **2015**, *8*, 460–464.
432. Liu XL, Chou GC, Tang QF, Gu CS, Gao MM. Analysis of etiological factors and characteristics of diarrhea in infants and young children in Yancheng City in 2018 (in Chinese). *Jiangsu J Prev Med* **2019**, *5*, 553–554.

433. Liu XM, Liu K, Liu RY. Investigation on an outbreak of infections diarrhea caused by norovirus (in Chinese). *Prev Med Tribune* **2017**, *8*, 638–639.
434. Liu XJ, Zhou WJ, Ma QF, Chen WW. Detection and analysis of norovirus in patients with acute diarrhea in a hospital in Fuzhou (in Chinese). *Strait J Prev Med* **2019**, *5*, 67–69.
435. Liu YW, Wang WS, Mo QH, Yang Z, Du T. Epidemiological features on 3 important viral diarrhea among children in Zhuhai during winter and spring (in Chinese). *Chin J Exp & Clin Virol* **2013**, *2*, 98–101.
436. Liu Y, Xu ZP. Investigation and analysis on a case of infectious diarrhea incident caused by norovirus (in Chinese). *J Med Pest Control* **2013**, *10*, 1130–1131.
437. Liu Y, Shu H. Investigation on cluster epidemics of norovirus transmitted by aerosol in a kindergarten (in Chinese). *Chin Med & Pharm* **2019**, *16*, 189–192.
438. Liu Y. Investigation and analysis on 3 norovirus outbreaks in a primary school in Beijing, 2014 (in Chinese). *Int J Viro* **2016**, *5*, 314–317.
439. Liu LJ, Zhu F, Fan F, Qian HF. Analysis of surveillance results of food borne diseases from sentinel hospitals in Zhangjiagang (in Chinese). *Chin J Health Lab Tech* **2016**, *5*, 733–735.
440. Long HY, Wen L, Long ZG. Surveillance and analysis of pathogens of viral diarrhea in infants and young children in Changsha area in autumn and winter (in Chinese). *Pract Prev Med* **2010**, *8*, 1669–1670.
441. Long JB, Zhou JS, Huang Y, Di B. Investigation and detection analysis of norovirus infectious diarrhea in a nursing home (in Chinese). *Pract Prev Med* **2014**, *3*, 297–298.
442. Long QZ, Chen X, Zhang PF, Peng WF. Investigation of an outbreak of norovirus infectious diarrhea in a school (in Chinese). *Pract Prev Med* **2016**, *12*, 1486–1488.
443. Long QZ, He L, Hu SX, Gao LD, Li JH. Etiological surveillance results of other infectious diarrhea in Zhuzhou, Hunan, 2015–2018 (in Chinese). *Dis Surveil* **2019**, *9*, 788–794.
444. Long QJ, Jie HC, Yao Y, Zheng Y, Zhang P. Etiological surveillance of norovirus infection and epidemiological characteristics of clusters in Jiulongpo District, Chongqing (in Chinese). *Strait J Prev Med* **2020**, *2*, 28–30.
445. Lou Q, Sun B, Zhang ZK, Zhang XY, Wang YY, Huang CW, Chen WX. Etiological study of enteric viruses and bacteria in adult diarrhea in Chongqing area (in Chinese). *Chin J Microecol* **2012**, *9*, 781–785.
446. Lu HP, Luo J, Xiao MS, Chen XJ. Analysis on norovirus infection characteristics and risk factors in infants in Taizhou area, Zhejiang (in Chinese). *Int J Epidemiol & Infect Dis* **2020**, *1*, 46–49.
447. Lu LB, Yan X, Zhao MX, Guo H, Fan Y. Analysis on epidemiological characteristics of foodborne diseases in Huairou District of Beijing from 2017–2018 (in Chinese). *Occup & Health* **2020**, *3*, 324–328.
448. Lu LB, Yan X, Zhao MX, Guo H, Fan Y, Liu D. Active monitoring results of food–borne diseases in Huairou District of Beijing from 2014–2016 (in Chinese). *Occup & Health* **2017**, *18*, 2494–2496. +2501.
449. Lu XD, Wang L, Peng JM. Pathogen analysis and epidemiological characteristics of viral diarrhea in hospitalized children in Zhongshan area (in Chinese). *Lab Med & Clin* **2019**, *12*, 1662–1664.
450. Lu ZP, Ceng YM, Zhang ZW, Zhang QL, Huang ZY, Chen YD, Li YF, Li JQ. Surveillance of infectious diarrhea in Dongguan, 2012 (in Chinese). *J Trop Med* **2014**, *3*, 369–371. +388.
451. Lu ZX, Chen YZ, Tang WQ, Yang ZQ, Jiang RJ. Investigation on an outbreak of Norovirus diarrhea in a university, Yancheng city (in Chinese). *Prev Med Tribune* **2016**, *11*, 832–835. +840.
452. Lu DL, Duan SG, Qi C, Liu H. Epidemiological features and dietary histories of foodborne disease cases in Shanghai, 2014–2018 (in Chinese). *Modern Prev Med* **2020**, *11*, 1970–1974.
453. Lu HM, Su JT, Tan BB, Nian CS. Analysis of surveillance results of foodborne diseases in Guigang City from 2014 to 2017 (in Chinese). *Appl Prev Med* **2018**, *2*, 149–150.
454. Lu H, Wu PC, Deng XC, He DH, Li W. Investigation and analysis of a clustered outbreak of norovirus gastroenteritis (in Chinese). *Appl Prev Med* **2016**, *4*, 346–347.
455. Lu H, Zhang BJ, Wang L, Han JJ. Analysis on pathogenic surveillance results of adult diarrhea cases in Chongming District of Shanghai City, 2015–2019 (in Chinese). *Strait J Prev Med* **2021**, *3*, 65–67.
456. Lu JY, Dai CW, Qiu XY, Lv HZ, Yang RX, Xu YZ, Ye J. Epidemiological investigation on an infectious gastroenteritis outbreak caused by GII.17 Norovirus (in Chinese). *Occup & Health* **2015**, *17*, 2431–2433. +2436.
457. Lu Q. Clinical analysis of 2376 patients in intestinal outpatient clinic (in Chinese). *Diet & Health care* **2016**, *18*, 27–28.
458. Lv CF, Liu S, Chen YH, Zhuo XN, Ou ZN, Pei GH. Investigation on a norovirus cluster outbreak caused by improper handling of vomitus from imported cases (in Chinese). *Chin J School Health* **2020**, *1*, 133–135.
459. Lv GJ, Shen TPP, Zhu JH. Analysis of Surveillance Results of Foodborne Diseases in Jinhua City (in Chinese). *Prev Med* **2017**, *7*, 698–701.
460. Lv LJ. Study on the etiology of diarrhea virus in infants under 5 years old (in Chinese). *Zhejiang Pract Med* **2015**, *2*, 108–109.
461. Lv P, Zhang RH, Qi XJ, Chen J. Analysis on the current situation of foodborne disease surveillance in Zhejiang Province (in Chinese). *Prev Med* **2015**, *8*, 828–829. +837.
462. Lv WW, Mao YX, Zhou H, Zhang J, Cha RS, Gao YJ, Lu BL, Zhang JJ, Yang HB. An outbreak of norovirus infection associated with contamination of secondary water supply in a hospital (in Chinese). *Dis Surveil* **2016**, *1*, 49–53.
463. Lv XZ. Investigation of an outbreak of norovirus gastroenteritis caused by water pollution (in Chinese). *Henan J Prev Med* **2018**, *6*, 468–469. +483.
464. Luan MC. Analysis on Norovirus infection among patients from food–borne disease sentinel surveillance points, Dalian city, 2015 (in Chinese). *Prev Med Tribune* **2017**, *2*, 115–117.

465. Luan MC, Liu YL, Lang XY, Yu L, Bao ZJ. Genetic characteristics of noroviruses from foodborne diarrhea in Dal ian (in Chinese). *Chin J Microecol* **2018**, *11*, 1313–1316.
466. Luan MC, Yu L, Liu YL, Bao ZJ. Norovirus type GI was detected for the first time in samples from an outbreak of acute gastroenteritis in Dalian (in Chinese). *Appl Prev Med* **2016**, *5*, 416–417.
467. Luo GH. Investigation and analysis of an acute gastroenteritis epidemic in a kindergarten in Baiyun District in 2012 (in Chinese). *China Health Care & Nutr* **2013**, *6*, 3283–3284.
468. Luo HL. 2017.7–2018.8 Norovirus in Qiannan Hospital of Traditional Chinese Medicine (in Chinese). *Home Med* **2018**, *8*, 26–27.
469. Luo JZ, Wang JP. Analysis of Foodborne Disease Active Surveillance in the Sentinel Hospital of Xinjiang Corps 2013–2015 (in Chinese). *J Prev Med Inform* **2016**, *11*, 1145–1148.
470. Luo KW, Zhao SL, Hu SX, Deng ZH, Ou YY, Zhang XH, Long QZ, Yao ZC, Gao LD, Li JH. Pathogen spectrum distribution of infectious diarrhea in Hunan province from 2015 to 2017 (in Chinese). *J Trop Dis & Parasitol* **2018**, *2*, 71–74.
471. Luo L, Wu XW, Liu YF, Li QY, Xie HP, Wu YJ, Li L, Jiang LY, Yang X. The molecular epidemiology characteristics of norovirus in environment and clinical samples in Guangzhou from 2009 to 2011 (in Chinese). *Chin J Prev Med* **2013**, *1*, 40–43.
472. Luo Y, Wang M, Zhu YY, Lin YY. Investigation of an outbreak caused by transmission of norovirus in Zhongshan city (in Chinese). *J Trop Med* **2017**, *4*, 540–542.
473. Luo L, Wang XH, Liu CP, Zhang YD, Huang L, Zhu HL. Surveillance of viral diarrhea in Pingshan District, Shenzhen, 2016–2017 (in Chinese). *South China J Prev Med* **2018**, *6*, 552–555.
474. Luo Q. Preliminary study on epidemiological characteristics and prevention and control of norovirus in primary and secondary schools (in Chinese). *China Pharm* **2019**, *Z1*, 117–118.
475. Luo TX. Investigation and result analysis of a food poisoning incident (in Chinese). *Health Prot & Prom* **2018**, *12*, 65–66.
476. Luo XM, Li XJ, Xu MQ, Liao GD. Etiological characteristics of viral diarrhea in Maoming during 2012 to 2015 (in Chinese). *J Trop Med* **2016**, *12*, 1574–1577.
477. Luo C, Dong C. Epidemiological characteristics and influencing factors of norovirus diarrhea cases in Bazhou, Xinjiang (in Chinese). *China Health Care & Nutr* **2020**, *8*, 300–301.
478. Luo LF, Wang XG, Li CZ, Yan HJ, Zhen SS, Liu JQ, He N. Pathogenic surveillance of viral diarrhea in Minhang District of Shanghai from 2014 to 2016 (in Chinese). *Fudan Univ J Med Scis* **2017**, *5*, 617–620. +625.
479. Luo SC, Chen XM, Liu H, Guo XY, Zhen SQ, Zu RQ. Distribution and epidemic characteristics of pathogenic pathogens of foodborne diseases in Huai'an from 2014–2016 (in Chinese). *Occup & Health* **2018**, *1*, 48–51.
480. Ma HP. Epidemiological status and prevention and control measures of norovirus infectious diarrhea in the Geriatrics Branch of Luohu District People's Hospital of Shenzhen City (in Chinese). *Chin Pract Med* **2016**, *8*, 114–115.
481. Ma JH, Ceng TX, Yang JP. Surveillance and Analysis of Diarrhea Syndrome Cases in Baiyin City (in Chinese). *Bull Dis Control & Prev (China)* **2019**, *4*, 36–38. +68.
482. Ma J. Analysis of foodborne disease surveillance results in sentinel hospitals in Zibo City in 2018 (in Chinese). *China Health Care & Nutr* **2020**, *25*, 21–22.
483. Ma LL. Analysis of foodborne disease surveillance situation in Yunlong District of Xuzhou City during 2014–2015 (in Chinese). *J Modern Med & Health* **2018**, *18*, 2828–2830.
484. Ma MM, Wang H, Lu JY, Wang DH, Ceng Q, Geng JM, Li TG, Zhang ZB, Yang ZC. Survey on a norovirus– borne outbreak caused by GII.4 Sydney 2012 variant in a university of Guangzhou, 2017 (in Chinese). *Chin J Epidemiol* **2018**, *12*, 1570–1575.
485. Ma SB, Wang XY, Tang YQ. Epidemiological characteristics of other infectious diarrhea in Changing District of Beijing City in 2013 (in Chinese). *Occup & Health* **2015**, *14*, 1935–1938.
486. Ma T, Hong L, Zhang Z, Xu YT, Feng LJ, Xu Q. An investigation report on a suspected water–borne norovirus outbreak (in Chinese). *Jiangsu J Prev Med* **2015**, *4*, 78–79.
487. Ma T, Zhang M, Hong L, Wang X, Dai WJ, Wu ZW, Wang R, Lin D, Xie GX. Outbreak investigation of acute gastroenteritis associated with GII.P7–GII.6 norovirus in a primary school of Nanjing in 2017 (in Chinese). *Modern Prev Med* **2018**, *22*, 4188–4191.
488. Ma X, Yu F, Liu Y, Su J, Yuan XM, Yang QD. Analysis of norovirus infection status in food–borne disease at 2015 in Xinjiang (in Chinese). *Chin J Health Lab Tech* **2016**, *19*, 2834–2835. +2840.
489. Ma XL. Analysis of norovirus infection status of foodborne diarrhea cases in sentinel hospitals in Fangshan District, Beijing from 2015 to 2018 (in Chinese). *Diet & Health care* **2021**, *7*, 276–277.
490. Ma YP, Chen RF, Zhang HF, Zhang SR, Meng H, Lu YZ. Surveillance results analysis of food–borne diarrhea pathogens in Lishui (in Chinese). *Chin J Health Lab Tech* **2018**, *8*, 934–937.
491. Ma YJ, Chen Z, Hu SL, Zheng YP, Li XB. Epidemiological analysis of viral diarrhea in infants and young children in Jinhua area (in Chinese). *Modern Pract Med* **2016**, *7*, 865–866.
492. Mao JY, Xiang LH, Shen JQ, Jin K, Yang XT, Yuan GP. Surveillance results of diarrhea in Baoshan District, Shanghai City, 2014 (in Chinese). *Pract Prev Med* **2016**, *2*, 215–218.
493. Mao JY, Xiang LH, Yuan GP, Gao Y, Shen JQ, Wang J, Yang XT. Investigation on an outbreak of acute gastroenteritis caused by norovirus infection in a school (in Chinese). *Shanghai J Prev Med* **2016**, *7*, 488–490. +507.
494. Mei GY, Xu Y, Yao XM, Zhang L, Wang SX. Epidemiological Status and Monitoring Results of Foodborne Diseases in Qixia District of Nanjing from 2016 to 2017 (in Chinese). *J Environ Hyg* **2019**, *2*, 113–117.
495. Meng J, Ge LX, Huang LM. An investigation report on an outbreak of norovirus infectious diarrhea (in Chinese). *Health Horizon* **2018**, *24*, 48.
496. Meng LX. Analysis and Significance of Virus Test in Children with Diarrhea (2) (in Chinese). *Oriental Med Diet* **2020**, *23*, 98.

497. Meng QH, Wang SH, Shi MY. Analysis of pathogen detection results of food borne diseases in a district of Tianjin city in 2017 (in Chinese). *Henan J Prev Med* **2018**, *11*, 841–843. +849.
498. Meng QH, Wang SH, Shi MY, Li ZY. Surveillance and analysis of foodborne disease pathogens in a district of Tianjin in 2015 (in Chinese). *Appl Prev Med* **2016**, *3*, 250–252.
499. Meng XJ. Epidemiological study of norovirus type II infection (in Chinese). *Chin J Prev Med* **2012**, *2*, 186.
500. Miao SH, Xu M, Wang YR, Zhang J, Wu L. Analysis of surveillance results of foodborne diseases from sentinel hospitals in Xuzhou city from 2015 to 2017 (in Chinese). *Modern Prev Med* **2018**, *24*, 4500–4503. +4512.
501. Miao SH, Zhang J, Wu L, Xue C. Surveillance results of foodborne diseases from sentinel hospitals in Xuzhou City, 2013–2014 (in Chinese). *Pract Prev Med* **2017**, *3*, 352–354.
502. Min P, Feng HE, Zhou ZK. Analysis of virus infection in children with diarrhea in Xining City (in Chinese). *J Med Forum* **2011**, *19*, 117–118.
503. Min P, Feng HE, Zhou ZK. Analysis on Sapovirus (SV) Detected from Infant and Children Diarrhea Cases in Xining City (in Chinese). *J Modern Lab Med* **2012**, *2*, 90–91.
504. Mo GQ. Epidemiological investigation on an outbreak of infectious diarrhea in a middle school in 2014 (in Chinese). *Henan J Prev Med* **2016**, *10*, 774–776.
505. Mo YJ, Wang XL, Yan RZ. Analysis of epidemiological characteristics of a cluster of infectious diarrhea caused by norovirus type GII in a school in Jiading District, Shanghai (in Chinese). *Shanghai Med & Pharm J* **2018**, *18*, 49–51.
506. Miu GZ, Ma Y, Lu HD, Tang JM, He Z, Jiang JZ, Wang YP, Gao HY. Investigation on infectious diarrhea pathogens and epidemiological characteristics in Jiangyin city (2012) (in Chinese). *J Public Health & Prev Med* **2013**, *5*, 37–40.
507. Miu GZ, Zhang J, He Z, Xu C, Zhang HB. Survey Report on an Outbreak of Norovirus GI Foodborne Illness (in Chinese). *J Med Pest Control* **2018**, *3*, 250–252.
508. Na YD, Cui W, Yi Y. Analysis of viral etiology of diarrhea syndrome in Liaoning during 2010–2014 (in Chinese). *Chin J Health Lab Tech* **2016**, *7*, 1030–1031.
509. Na YD, Yi Y. Detection and identification of Norovirus GII.17 type in Panjin area (in Chinese). *Chin J Health Lab Tech* **2015**, *22*, 3861–3862.
510. Ni CR, Lv B, Liu TC, Zhang XM, Pan QJ, Hu CS, Fu B, Cai J. Investigation on a norovirus gastroenteritis outbreak affected four schools (in Chinese). *Prev Med* **2019**, *3*, 305–308.
511. Ni CR, Wang J, Pan QJ, Xu F, Xiang FL, Ying SY. Outbreak investigation of acute gastroenteritis associated with norovirus in Wenzhou (in Chinese). *Chin J Public Health Manage* **2019**, *6*, 854–857.
512. Ni CY, Zhang LQ. An investigation report on an outbreak of norovirus diarrhea in a school (in Chinese). *China Health Care & Nutr* **2020**, *13*, 327–328.
513. Ni HX, Xie L, Yang TC, Dong HJ, Xu GZ. Analysis on outbreaks of acute gastroenteritis caused by norovirus in Ningbo, 2014 (in Chinese). *Int J Epidemiol & Infect Dis* **2015**, *5*, 293–296.
514. Nong H, Liu HH, Yang C, Yin LJ, Zhan XJ, Qin JQ. Results of norovirus detection of some diarrhea specimens in Nanning City, Guangxi Province (in Chinese). *Modern Prev Med* **2020**, *3*, 488–491.
515. Ou SX, Liu W, Chen WH. Investigation on the Outbreak of Norovirus in Kindergartens in Chenzhou (in Chinese). *China Health Industry* **2019**, *8*, 147–149.
516. Pan F, Shen YC, Li ZD. Investigation on Outbreak of Infectious Diarrhea Caused by Norovirus in a University (in Chinese). *Health Med Res & Pract* **2014**, *1*, 27–28.
517. Pan H, Li J, Wu HY, Gong XH, Xiao WJ, Hu JY, Yuan ZA. Incidence of diarrheal diseases in Shanghai city, 2013–2015: results of integrated surveillance (in Chinese). *Chin J Public Health* **2017**, *10*, 1507–1511.
518. Pan LF. Detection and analysis of the norovirus infection from 2011 to 2014 in Pudong New District of Shanghai, China (in Chinese). *Chin J Viral Dis* **2015**, *3*, 212–216.
519. Pan LF, Liu D, Liu Q, Ma P, Zhu LY, Fu YF, Sun Q. Detection and Analysis of Viral Diarrhea in Pudong New District, Shanghai, 2011 (in Chinese). *Chin Primary Health Care* **2012**, *9*, 50–52.
520. Pan LF, Lu L, Ding M, Qiu HF, Liu Q, Zhu LY, Ma P, Hao LP. Investigation on infection status of viral diarrhea among adults in Pudong New District of Shanghai from 2012–2013 (in Chinese). *Occup & Health* **2015**, *7*, 929–931.
521. Pan YF, Chen ZX, Wang BM, Jin HY, Sun Z, Zheng L, Hu XW. Epidemiological investigation of an outbreak of norovirus G II gastroenteritis on a university campus (in Chinese). *Chin Prev Med* **2017**, *1*, 69–71.
522. Pan YF. An Outbreak of Norovirus Gastroenteritis Caused by Water Pollution in a Troop in Training (in Chinese). *Advances Clin Med* **2020**, *8*, 1817–1821.
523. NASurveillance and analysis of the pathogens of viral gastroenteritis in 2011 and 2013, Wuhan, China (in Chinese). *Chin J Zoonoses* **2016**, *5*, 457–461.
524. Pang ZF, Lou MF, Zhang GM, Zhang ZG, Shen TPP. Investigation of an acute gastroenteritis outbreak caused by norovirus in a middle school in Jinhua of Zhejiang province (in Chinese). *Dis Surveil* **2017**, *5*, 437–438.
525. Pang ZF, Zhang ZG, Wu XH, Fang QL. Survey on an outbreak of norovirus related acute gastroenteritis in a kindergarten in Jinhua City of Zhejiang (in Chinese). *Modern Prev Med* **2014**, *9*, 1697–1698.
526. Pang ZM. Analysis of Norovirus Cluster Epidemic in Baiyun District, Guangzhou City (in Chinese). *Strait J Prev Med* **2015**, *6*, 52–54.
527. Peng P, Xie FS, Liu BX. Epidemiological and etiological characteristics of viral diarrhea in children under 10 years old in Tianjin in 2017 (in Chinese). *Shandong Med J* **2018**, *39*, 74–76.

528. Peng XX, Huang M, Dang RB, Lu JY, Zhang Z, Mei SJ. An epidemiological investigation of a suspected food poisoning event in a primary school in Shenzhen city (in Chinese). *China Trop Med* **2015**, *7*, 845–847. +856.
529. Pu PL, Shao B, Song YF, Wu FM, Xu ZY. Investigation of an outbreak of gastroenteritis caused by norovirus (in Chinese). *Jiangsu J Prev Med* **2017**, *3*, 316–317.
530. Qi HZ, Zhong H. Genetic analysis of norovirus in Changsha (in Chinese). *J Community Med* **2014**, *12*, 12–13.
531. Qi XQ, Wang ZY, Zheng TT. Analysis of surveillance results of foodborne diseases in Sanmen County (in Chinese). *Prev Med* **2021**, *6*, 631–633.
532. Qi YQ. Investigation on an outbreak of GII norovirus acute gastroenteritis in kindergarten (in Chinese). *Chin J School Doct* **2018**, *11*, 851–853.
533. Qi J, Li PG. Analysis of proactive monitoring results of food–borne diseases in Pinggu district of Beijing in 2018 (in Chinese). *J Food Safety & Quality* **2019**, *12*, 3760–3764.
534. Qi XQ, Cai SJ, Xie ZH, Ou JM, Wu BS, Chen MH, Huang WL. Investigation on a diarrhea outbreak caused by norovirus in a university in Fujian Province (in Chinese). *Strait J Prev Med* **2019**, *4*, 17–19.
535. Qi Y, Chen Y, Wang B, Wang P, An XD, Liu Y, Shao ZC, Wen Y. Survey on norovirus outbreaks in a school (in Chinese). *Prev Med Tribune* **2018**, *11*, 821–824. +831.
536. Qi Y, Wang P, Chen Y, Wang B. Epidemiological characteristics of norovirus aggregations and outbreaks in Shenyang City in 2017 (in Chinese). *Occup & Health* **2019**, *1*, 65–68.
537. Qian LZ, Hong WS, Jin RY. Investigation and analysis of an outbreak of norovirus infection in a university (in Chinese). *Appl Prev Med* **2020**, *3*, 211–212. +214.
538. Qian YH, Shao J, Shi C, Zhou WJ, Zhu Y, Zu RQ, Yu RB. Establishment and implication of infectious diarrhea surveillance system in Wuxi City (in Chinese). *Jiangsu J Prev Med* **2011**, *5*, 1–3. +6.
539. Qian ZY, Zhang YW, Zhou Q, Shen XT. An outbreak of nosocomial norovirus gastroenteritis (in Chinese). *Chin J Nosocomiol* **2012**, *12*, 2573–2575.
540. Qiao FJ, Sun BX, Sun Y, Yin Y. Analysis of molecular biological characteristics of an acute gastrointestinal tract incidence caused by norovirus GII in a university campus (in Chinese). *Chin J Health Lab Tech* **2018**, *17*, 2107–2108. +2112.
541. Qiao HY. Analysis of Active Surveillance of Foodborne Diseases in a Hospital from 2018 to 2020 (in Chinese). *Henan Med Res* **2021**, *11*, 1974–1977.
542. Qiao K, Luo LF, Song CP, Liu JQ, Chen GQ, Ou YL, Chen XH, Wang XG. Etiological study on enteric viruses in adult diarrhea in the southwest of Shanghai (in Chinese). *Occup & Health* **2014**, *14*, 2012–2014.
543. Qiao YQ, Yu F, Chen WB, Xu HM. Epidemiological characteristics and molecular subtype identification of norovirus associated diarrhea in 511 children from Chongqing region during the year of 2014 (in Chinese). *J Army Med Univ* **2016**, *1*, 101–106.
544. Qin D, Chu YH, Sun JY, Li JS, Zhang Z. Epidemiological investigation on a cluster of GII.6 norovirus (in Chinese). *Chin J School Health* **2016**, *5*, 794–797.
545. Qin LY. Epidemiological Investigation and analysis of a suspected epidemic situation of norovirus infectious diarrhea in a school (in Chinese). *The Med Forum* **2021**, *10*, 1466–1467.
546. Qin M, Dong XG, Zhang DY, Yang JY, Xiao GY, Shang X, Lu YY, Zhang H, Li J. Laboratory analysis of prevalence of Roxavirus infectious Diarrhea in Fengtai District of Beijing during November 2013 (in Chinese). *Chin J Exp & Clin Virol* **2014**, *6*, 404–406.
547. Qin M, Lu YY, Feng HR, Yang JY, Wu QR, Yu H, Wang ZE, Dong XG, Li J. Etiological identification and genotyping of norovirus detected in gastroenteritis outbreaks in Fengtai, Beijing (in Chinese). *Dis Surveil* **2015**, *2*, 118–121.
548. Qin TX. Investigation on the first norovirus food poisoning incident in Guilin (in Chinese). *China Health Care & Nutr* **2013**, *1*, 397–398.
549. Qin YM, Yang XK, Yang H, He YQ. Epidemiological investigation and laboratory test analysis on an outbreak of acute gastroenteritis caused by norovirus in Shenzhen (in Chinese). *South China J Prev Med* **2010**, *3*, 18–21.
550. Qiu CL. Etiological characteristics of viral diarrhea in Shaoguan city (in Chinese). *J Trop Med* **2015**, *5*, 708–710.
551. Qiu HY, Xu ZG, Lan WQ, Wu XL, Cheng Z. The investigation ofall infectious diarrhea epidemic caused by norovirus in a rural middle school (in Chinese). *Int Med & Health Guid News* **2013**, *14*, 2246–2248.
552. Qiu X, Sheng FS, Sun ZX. Surveillance results of foodborne diseases in Songjiang District of Shanghai from 2016–2018 (in Chinese). *Occup & Health* **2019**, *13*, 1774–1777.
553. Qiu ZY, Zhang M, Wu LL, Chui HX, Zhang XL. Surveillance situation of foodborne norovirus in Henan between 2016 and 2018 (in Chinese). *Modern Prev Med* **2019**, *14*, 2551–2553.
554. Ren FL, Cai YB, Li C. Epidemiological investigation of an outbreak of norovirus gastroenteritis in a kindergarten (in Chinese). *Shanghai J Prev Med* **2013**, *5*, 234–236.
555. Ren FL, Jiang TH, Li C. Epidemiological investigation of a school foodborne norovirus outbreak (in Chinese). *Chin Primary Health Care* **2013**, *11*, 98–99.
556. Ren LJ, Cai WL, Bai XX, Bai Y. Investigation and analysis about an outbreak of gastroenteritis caused by norovirus in a kindergarten (in Chinese). *Henan J Prev Med* **2020**, *7*, 564–566.
557. Ren QZ, Liu HB, Wen DA, Ma ZJ. Epidemiological analysis of Norovirus infection in Nangang district of Harbin, 2016–2017 (in Chinese). *Chin J Public Health Manage* **2018**, *6*, 831–833.
558. Ren SM, Wang QY, Qu M, Liu F, Zhao L, Zhao H. Analysis of infectious diarrheal disease in Yanqing District, Beijing in 2016 (in Chinese). *Chin J Health Lab Tech* **2018**, *7*, 855–858.
559. Ren YP, Shen HP, Qu F, Bai PQ. Results of active surveillance of foodborne diseases in Pudong New Area of Shanghai, 2015–2018 (in Chinese). *Chin J Food Hyg* **2020**, *6*, 676–680.

560. Ren Y, Xu SJ, Chen HB, Lai XL, Huang XY, Man YX, Hao XQ, Li JZ, Liu JX, Quan DF, et al. Analysis of genotypic characteristics of Norovirus in Longhua district of Shenzhen city in 2018 (in Chinese). *J Trop Med* **2020**, *2*, 203–207.
561. Ren Y, Xu SJ, Zhang HL, Quan DF, Lai XL, Man YX, Hao XQ, Liu JX, Wang JM, Lin QH. Laboratory analysis of viral diarrhea pathogens in Longhua district, Shenzhen city from 2015 to 2017 (in Chinese). *J Trop Med* **2018**, *10*, 1346–1350.
562. Ren YH, Gong LQ, Xu LQ. Investigation on an outbreak of acute gastroenteritis with norovirus type G II in a school (in Chinese). *Jiangsu J Prev Med* **2016**, *1*, 91–93.
563. Rong JR, Lu L, Wu FQ, Yuan YR. Etiological surveillance and analysis of infectious diarrhea in Jiangdong, Ningbo from 2011 to 2012 (in Chinese). *Modern Prev Med* **2014**, *7*, 1310–1312.
564. Rong XS, Ma RJ, Jiang QL, Li H, Zhou HL. Surveillance and analysis of foodborne diseases with Norovirus infection in Aksu prefecture (in Chinese). *Bull Dis Control & Prev (China)* **2021**, *2*, 64–66.
565. Ruan Y, Mo LY, Zou AJ. Etiological surveillance results of infectious diarrhea in children in Hunan region, 2016–2018 (in Chinese). *Pract Prev Med* **2019**, *11*, 1372–1374.
566. Rui F, Zhang L. Epidemiological characteristics of clusters epidemic and outbreaks of norovirus in Hefei City in 2016 (in Chinese). *Occup & Health* **2018**, *2*, 241–244.
567. Sang H. Epidemiological characteristics and etiological analysis of adult infectious diarrhea in a hospital in Shanghai (in Chinese). *Shanghai J Prev Med* **2016**, *11*, 810–813.
568. Sang H, Cui Y, Gu WC, Kong LN, Wang R, Jiang XL, Wang QH. Epidemiological characteristics of diarrhea in children and drug resistance of diarrheagenic Escherichia coli in Shanghai, 2015–2018 (in Chinese). *Dis Surveil* **2019**, *6*, 559–564.
569. Sang XL. Supervision and Analysis on Factors associated with Diet of foodborneI Diarrhea among Children (in Chinese). *Chin Primary Health Care* **2016**, *8*, 62–63.
570. Sang XL. The epidemiologic features and influence factors of foodborne norovirus in Gansu Province (in Chinese). *Modern Prev Med* **2018**, *24*, 4423–4426.
571. Sha BRM·THTM, A YGL·YEHL, Liu HB, Wu YW, Li XL. Surveillance of viral diarrhea among hospitalized children in Urumqi, 2010 (in Chinese). *Dis Surveil* **2013**, *6*, 439–442.
572. Sha BRM·THTM, Xiao KLT·NE, Zhou HJ, Jin M, Kong XY, Zhou YK, Li HY, Duan ZJ, Ma HMT. Molecular epidemiology of norovirus infection in hospitalized children with acute gastroenteritis in Urumqi, Xinjiang, 2012–2014 (in Chinese). *Dis Surveil* **2018**, *10*, 814–818.
573. Shang LH, Xiong LJ, Liu LR, Deng XZ, Xie XL. Epidemiological and clinical features of calicivirus–associated diarrhea in hospitalized children in Chengdu, China from 2012 to 2014 (in Chinese). *Chin J Contemp Pediatr* **2016**, *10*, 1005–1008.
574. Shang XC, Shuai HQ, Zhao XQ, Huang QH, Zhou XH. Etiological and epidemiological characteristics of viral diarrhea in infants and young children in Hangzhou from 2012 to 2019 (in Chinese). *Chin J Dis Control & Prev* **2020**, *9*, 1110–1112.
575. Shen HW, Ceng HS, Ou YX, Xu YC, Ke JY, Chen HC. Analysis of viral diarrhea surveillance in Futian District, Shenzhen during 2015–2017 (in Chinese). *Chin J Health Lab Tech* **2019**, *21*, 2561–2563. +2570.
576. Shen TPP, Lv GJ, Zhu JH. Surveillance results analysis of foodborne diseases in Jinhua during 2014–2017 (in Chinese). *Chin J Health Lab Tech* **2018**, *22*, 2803–2806.
577. Shi WY. Molecular epidemiological characteristics of 20 epidemic outbreaks of norovirus in Zhongshan, 2013–2017 (in Chinese). *Pract Prev Med* **2020**, *12*, 1464–1467. +1482.
578. Shi AP, Shi JP, Wu PC, Fan F, Xu L. Analysis of surveillance results of foodborne diseases in sentinel hospitals in Zhangjiagang City from 2016 to 2018 (in Chinese). *J Med Pest Control* **2021**, *1*, 64–67.
579. Shi C, He EQ, Xiao Y, Shao J, Feng WH, Qian YH. An investigation on an outbreak of norovirus gastroenteritis in a school (in Chinese). *Modern Prev Med* **2013**, *6*, 1120–1121. +1127.
580. Shi QF, Pan XD, Shao WF, Wang YP. Analysis on the distribution characteristics of pathogens in 124 children with viral diarrhea (in Chinese). *Matern & Child Health Care China* **2014**, *14*, 2178–2180.
581. Shi QF, Pan XD, Wang YP, Shao WF, Qian JY, Sun AH. Analysis of relationship between viral infection and diarrhea–related clinical parameters in infants (in Chinese). *Chin J Nosocomiol* **2015**, *18*, 4252–4254. +4267.
582. Shi HY, Liu Y, Fan PC, Li X. Genetic characteristics of recombinant norovirus in an acute gastroenteritis epidemic (in Chinese). *Modern Prev Med* **2019**, *12*, 2251–2254.
583. Shi P, Qian YH, Shi C. Analysis on monitoring of diarrhea infectious and evaluation on effect of early warning in Wuxi City from 2010–2013 (in Chinese). *Occup & Health* **2016**, *5*, 641–644.
584. Shi X. Analysis on the detection of norovirus and its genetic characteristics among patients with diarrhea in Heilongjiang Province from 2017 to 2019 (in Chinese). *Chin J Public Health Manage* **2020**, *4*, 457–459. +498.
585. Shi WF, Liu HB. Analysis on Etiological Surveillance of Rotavirus and Norovirus Among Diarrhea Cases Over 14 Years Old from the Enteric Diseases Clinic in Fangshan District of Beijing from 2015 to 2017 (in Chinese). *J Prev Med Inform* **2019**, *4*, 338–341.
586. Shi YL, Cha ZQ, Hu WF, Wu JB, Wang JJ. Molecular epidemiological characters of viral diarrhea in infants and young children less than 5 years old in surveillance hospitals from Anhui Province, 2011–2012 (in Chinese). *Chin J Dis Control & Prev* **2014**, *6*, 508–511.
587. Shi YL, Kong XY, Jin M, Fang JC, Shi XD, Hu WF. Molecular etiological characteristics of the gastroenteritis outbreak associated to norovirus infection in Anhui province (in Chinese). *Chin J Exp & Clin Virol* **2015**, *4*, 310–312.
588. Shu YP. Analysis of foodborne disease surveillance results in Huangyan District, Taizhou City from 2017 to 2018 (in Chinese). *Chin J Rural Medicine & Pharm* **2020**, *8*, 48–49.

589. Shuai HQ, Shou J, Shang XC, Zhao XQ. Investigation of an infectious diarrhea epidemic caused by norovirus in a school in Xiacheng district, Hangzhou (in Chinese). *Dis Surveil* **2012**, *12*, 1013–1014.
590. Song CL, Dong ZP, Zhu L, Wang T, Mo PH, Li L. Confirmation and epidemiological investigation of a GII.P7–GII.6 norovirus outbreak in a primary school of Jinshan District, Shanghai (in Chinese). *China Trop Med* **2020**, *5*, 443–447.
591. Song CL, Li L, Cao ZC, Mo PH, Zhong HM. Analysis on the outbreak of norovirus gastroenteritis in an elderly nursing home (in Chinese). *J Public Health & Prev Med* **2012**, *1*, 93–94.
592. Song CL, Li L, Yuan JC, Cao ZC, Zhong HM. Molecular characteristics of the clustering of norovirus gastroenteritis in Jinshan (in Chinese). *Chin J Health Lab Tech* **2013**, *5*, 1177–1179.
593. Song CL, Liu T, Du JR, Zhu JM. Epidemiological investigation on an outbreak of norovirus: induced infectious diarrhea in a primary school in Jinshan Dstrict (in Chinese). *Pract Prev Med* **2017**, *4*, 429–432.
594. Song CL, Yuan JC, Cao ZC, Li L, Zhong HM, Zhu JM, Gao X. Molecular epidemiology study on an acute gastroenteritis outbreak caused by a new GII.4 norovirus recombinant strain (in Chinese). *Chin J Prev Med* **2013**, *12*, 1114–1117.
595. Song CL, Yuan JC, Li L, Cao ZC, Zhu JM, Gao X. Investigation on norovirus infection of close contacts and environment contamination in nursing home (in Chinese). *Chin J Health Lab Tech* **2013**, *16*, 3290–3292.
596. Song CL, Zhu JM, Yuan JC, Li J, Li L, Jin YQ, Xu F. Epidemiological investigation of norovirus gastroenteritis with Staphylococcus aureus infection (in Chinese). *Shanghai J Prev Med* **2013**, *2*, 76–77.
597. Song HR, Liu XD, Wang X. Investigation of an outbreak caused by norovirus infection (in Chinese). *Chin J Public Health Manage* **2017**, *5*, 727–729.
598. Song JQ, Zhu XW, Lou YW, Jiang WP, Shang Y. Investigation of an outbreak of norovirus acute gastroenteritis caused by unclean bottled water (in Chinese). *Prev Med* **2015**, *10*, 1045–1048.
599. Song J, Chen FG, Zhao W, Fan WW. Survey of a diarrhea outbreak caused by norovirus in a nursery school (in Chinese). *J Med Pest Control* **2014**, *10*, 1121–1123.
600. Song YF, Wu FM, Pu PL, Xu ZY. Investigation on an outbreak of school norovirus infection (in Chinese). *Int J Epidemiol & Infect Dis* **2019**, *3*, 244–246.
601. Su AR, Wang H, Du Y, Li XG, Tan DM, Qu C, Wei CY, Huang Y. Correlation analysis of foodborne diseases caused by norovirus and Vibrio parahaemolyticus (in Chinese). *Occup & Health* **2020**, *12*, 1632–1635.
602. Su J, Xu HY, Xiong HP. Results of virological surveillance on infectious diarrhea in Nantong City from 2012–2014 (in Chinese). *Occup & Health* **2016**, *22*, 3080–3082. +3087.
603. Su T, Liu YY, Yu QL, Zhao WN, Xie Y, Li Q, Qi SX. Infection status and epidemiology of Human calicivirus (HuCV) among children under 5 years of age in sentinel hospitals in Hebei Province, 2010–2015 (in Chinese). *J Pathog Bio* **2017**, *12*, 1188–1191. +1214.
604. Su T, Liu YY, Zhao WN, Yu QL, Xie Y, Qi SX, Li Q. Epidemiological investigation of an outbreak of norovirus–caused gastroenteritis (in Chinese). *Dis Surveil* **2020**, *4*, 362–366.
605. Su WJ. Virus Infection and Preliminary Analysis of Diarrhea Specimens in Children in Xining City (in Chinese). *J Front Med* **2013**, *33*, 246–247.
606. Su WZ, Geng JM, Lai SL, Liu JW, Wang H, Chen C, Wang DH, Di B, Zhang ZB, Xie HP. A molecular epidemiological survey on the outbreak of recombinant Norovirus GII.P16–GII.2 in Guangzhou city (in Chinese). *Chin J Exp & Clin Virol* **2019**, *4*, 362–368.
607. Sun B, Zhou F, Li XQ. Analysis on Pathogen Infection in Children Patients with Viral Diarrhea in He'nan Children's Hospital and Its Intervention Measures (in Chinese). *Anti–infect Pharmacy* **2021**, *1*, 107–110.
608. Sun HB. Survey of food–borne diarrhea outbreaks caused by norovirus in a restaurant (in Chinese). *Occup & Health* **2012**, *15*, 1865–1866.
609. Sun HM, Huang JP, Zhang WB, Tan WW. Epidemiological characteristics of pathogenic pathogens of food–borne diseases in Nantong City from 2015–2017 (in Chinese). *Occup & Health* **2019**, *3*, 367–370.
610. Sun JF, Ji LL, Chen LX, Song SE. Monitoring results of viral diarrhea in Huairou District of Beijing in 2012 (in Chinese). *Occup & Health* **2014**, *15*, 2092–2094.
611. Sun J, Liu M, Wang JQ. Epidemiological investigation on a norovirus outbreak in one university (in Chinese). *Chin J School Health* **2015**, *7*, 1069–1071.
612. Sun LM, Chen SX, Liang JH, Wu XL, Li H, He CY, Zhou XH. Investigation of a gastroenteritis outbreak caused by well water polluted by norovirus GII.4 in rural schools in Guangdong (in Chinese). *Modern Prev Med* **2012**, *7*, 1618–1621.
613. Sun LY. Investigation on a cluster of norovirus infections in Suzhou (in Chinese). *Electron J Clin Med Literature* **2020**, *103*, 178–179.
614. Sun MH, Xu PH, Zhou XH, Zhu XW, Shang Y. An epidemic report of clustered diarrhea caused by GII.17 norovirus (in Chinese). *Prev Med* **2017**, *2*, 185–186. +190.
615. Sun MH, Zhou XH, Xu PH, Xu JY. Monitoring and genotype analysis of the norovirus epidemic strains in Haiyan county in 2013 (in Chinese). *Chin J Health Lab Tech* **2015**, *16*, 2777–2779.
616. Sun Q, Liu DM, Xie NJ. Investigation on an outbreak of norovirus infection in a kindergarten in Huaining County (in Chinese). *Anhui J Prev Med* **2019**, *5*, 366–369.
617. Sun QS. Analysis of etiological surveillance results of diarrheal disease surveillance sites in Jinshan District, Shanghai in recent 2 years (in Chinese). *Shanghai J Prev Med* **2017**, *3*, 209–211.
618. Sun WL. Investigation and disposal report of suspected "norovirus infection" in a Kindergarten in Tianjin Hongqiao district (in Chinese). *Henan J Prev Med* **2018**, *1*, 71–72. +75.

619. Sun YN, Liu JL. Surveillance results of pathogens of foodborne diseases in Jinnan District of Tianjin from 2016–2017 (in Chinese). *Occup & Health* **2020**, *18*, 2486–2489.
620. Sun YM, Lin Y, Luo JY, Wang HH. Analysis on epidemic characteristics of foodborne norovirus sporadic infection in Jiaxing (in Chinese). *Chin J Public Health Manage* **2016**, *5*, 647–648. +660.
621. Sun YM, Luo JY, Wang HH, Lin Y. Surveillance results of foodborne diseases with specific pathogens in Jiaxing City (in Chinese). *Prev Med* **2016**, *4*, 411–412. +426.
622. Sun Y, Qiao FJ, Wang H, Sun BX. Molecular epidemiological characteristics of norovirus in foodborne diseases cases in Changchun City, Jilin Province, China in 2016 (in Chinese). *Chin J Biologicals* **2018**, *12*, 1356–1359.
623. Sun YL, Wang GQ, Xing YF, Gong CB, Dong FG, Wang CX. Analysis on surveillance results of food–borne diseases from sentinel hospital of Yantai in 2016 (in Chinese). *Modern Prev Med* **2017**, *20*, 3798–3800. +3812.
624. Sun YL, Yang LY, Ding ZY. Investigation of an outbreak of infectious diarrhea caused by norovirus in a school (in Chinese). *Jiangsu J Prev Med* **2018**, *1*, 59–60.
625. Sun Z, Fang LH, Xu Z, Kao QJ, Xie L. A gastroenteritis outbreak caused by tap water polluted by norovirus in a town of Zhejiang (in Chinese). *Chin Rural Health Service Adm* **2016**, *4*, 446–448.
626. Tan L. Norovirus infection surveillance in early warning of outbreaks in population in oyster farming areas (in Chinese). *J Hyg Res* **2017**, *2*, 201–206. +212.
627. Tan DM, Liu W, Deng LL, Yu MK, Tang ZZ, Ma YY, Wei YZ, Zhuo JT. Detection of Norovirus Genogroup I Type 1 from a Gastroenteritis Outbreak in Guangxi Zhuang Autonomous Region (in Chinese). *Chin J Vaccines & Immuniz* **2012**, *5*, 451–455.
628. Tan WW, An N. Analysis on monitoring results of foodborne diseases in sentinel hospitals of Nantong urban area from 2013–2014 (in Chinese). *Occup & Health* **2015**, *20*, 2776–2779.
629. Tan WW, Zhang WB, Xu HY, Huang JP. Analysis of surveillance results of viral diarrhea of Nantong City in 2017 (in Chinese). *Modern Prev Med* **2018**, *20*, 3803–3806.
630. Shang YH. An outbreak investigation of norovirus acute gastroenteritis in a school (in Chinese). *Chin Rural Health Service Adm* **2017**, *12*, 1482–1484.
631. Tang GJ. Investigation on a cluster outbreak of acute gastroenteritis caused by norovirus infection (in Chinese). *Diet & Health care* **2019**, *16*, 296.
632. Tang X, Chen RJ, Lai FF, Huang AL, Xu HM. The genotype and recombination of norovirus in children with diarrhea caused by viral infection in Chongqing, 2012 (in Chinese). *Chin J Evidence–Based Pediatr* **2014**, *3*, 167–171.
633. Tang XL. A report of an outbreak caused by delayed diagnosis of norovirus infection (in Chinese). *J Nursing (China)* **2013**, *18*, 65–68.
634. Tang YX, Chen X, Cai XF, Li YY. An outbreak of norovirus gastroenteritis caused by water pollution in school in Zhuzhou City (in Chinese). *Occup & Health* **2014**, *15*, 2174–2176.
635. Tang Z. Molecular characteristics of norovirus (NV) in acute foodborne disease in Jiangsu Province from 2013 to 2014 (in Chinese). *J Nanjing Med Univ (Natural Scis)* **2016**, *10*, 1213–1217. +1236.
636. Tao LY. Epidemiological investigation of a school public health emergency caused by norovirus infection (in Chinese). *Intinfects Dis (Electron E)* **2020**, *2*, 236–237.
637. Tian D, Liu GX, Xu Q, Zhao SC, Zhang HY, Feng YL, Xiao JY. Analysis of the prevalence of norovirus genotypes in Qinghai Province from 2016 to 2017 (in Chinese). *J Pathog Bio* **2018**, *11*, 1258–1260.
638. Tian G, Jin M, Wang J, Wang X, Kang LH. Analysis of clinical features of community–acquired sporadic norovirus gastroenteritis (in Chinese). *J Pract Med* **2015**, *19*, 3222–3224.
639. Tian H, Lei Y, Liu Y, Zhang Y, Li JM. Pathogenic Analysis on Viral Diarrhea in Tianjin (in Chinese). *J Environ & Health* **2011**, *7*, 605–608.
640. Tian JG, Zhang LL, Wang YF, Zhu S, Chen Y. Analysis on Surveillance Results of Foodborne Diseases Caused by Grain and Its Products in Guizhou Province in 2018 (in Chinese). *J Med Inform* **2019**, *21*, 121–124.
641. Tian J, Li LL, Shi L, Dong RQ, Kan Z, Cui LM. Epidemiological survey of an outbreak of acute gastroenteritis caused by norovirus G type I in primary school in Dongcheng district in Beijing (in Chinese). *J Prev Med Inform* **2017**, *10*, 973–976.
642. Tian YL, Sun CJ, Dang YL. Investigation and analysis of clustered epidemic situation of norovirus infectious diarrhea in a middle school in Beijing Olympic Village (in Chinese). *Gems Health* **2021**, *2*, 271+276.
643. Wang HB, Wang Q, Tu CN, Tan H, Yang Z, Mo QH. Epidemiological analysis on eight outbreaks of infectious diarrhea in Guangdong (in Chinese). *Chin J Front Health & Quarantine* **2015**, *5*, 348–351.
644. Wang H, Wang DH, Chen C, Zhang ZB, Li TG, Wang M. Molecular epidemiological characteristics of norovirus in diarrhea cases in Guangzhou (in Chinese). *Dis Surveil* **2016**, *10*, 838–842.
645. Wang JS, Fan LF, Li XX, Gao WL, Wu ZC, Cao MC. Investigation on an outbreak of norovirus infectious diarrheaina, junior middle school in Anqing City (in Chinese). *Anhui J Prev Med* **2019**, *5*, 394–397.
646. Wang JS, Sun Q, Cao MC, Li XX, Shi YL, Jin AW. Epidemiological survey of a waterborne outbreak of acute gastroenteritis caused by norovirus (in Chinese). *Anhui J Prev Med* **2016**, *2*, 87–90.
647. Wang Y. Epidemiological study of norovirus infection among sporadic cases of diarrhea in Guangzhou area from 2012 to 2013 (in Chinese). *J Trop Med* **2014**, *5*, 567–570.
648. Wang AN, Zhong XW, Tan L, Wan Z, Chen RF, Lin H, Huang Q, Zhang YH. Circulation routes of norovirus among population and environment in coastal area (in Chinese). *South China J Prev Med* **2016**, *2*, 101–107.
649. Wang B, Zhang CQ, Wang P, Zhang MM, Lian ZY. The molecularly etiological study on the GII.17 norovirus causing an acute gastroenteritis outbreak in Shenyang (in Chinese). *Chin J Exp & Clin Virol* **2017**, *3*, 208–211.

650. Wang CR, Guan HY, Liu LZ, Li Z, Yang GL. Analysis on rotavirus and norovirus infections among patients with acute diarrhea in autumn and winter in Jinan (in Chinese). *Chin J Clinicians (Electron E)* **2015**, *15*, 2843–2845.
651. Wang DH, Ding P, Chen C, Xiao XC, Luo L, Li MX. Epidemiological characteristics analysis of other infectious diarrhea in Guangzhou in 2011 (in Chinese). *J Med Pest Control* **2012**, *10*, 1123–1124.
652. Wang DH, Li MX, Wang H, Lu Y, Chen C, Pan XH, Li XN. Investigation and analysis on the outbreak of Norovirus infection in a university (in Chinese). *J Med Pest Control* **2020**, *12*, 1202–1206.
653. Wang DY, Wang J, Chang WB, Shen MZ, Cai M. Analysis of active surveillance of foodborne diseases in Changshu City from 2012 to 2015 (in Chinese). *J Public Health & Prev Med* **2016**, *2*, 71–73.
654. Wang G, Shen TPP, Zhu JH. Analysis of the surveillance results of food borne diseases in Jinhua in 2014 (in Chinese). *Chin J Health Lab Tech* **2016**, *1*, 113–114. +131.
655. Wang HY, Tu LX, Ye HY. Infants and young children with acute diarrhea virus test results (in Chinese). *Prev Med* **2014**, *8*, 848–850.
656. Wang H, Li WD, Li JY. Investigation of an outbreak of infectious diarrhea caused by norovirus in an enterprise (in Chinese). *Anhui J Prev Med* **2018**, *6*, 438–440. +406.
657. Wang H, Guan XY, Wang J. Investigation report on a cluster outbreak of norovirus gastroenteritis (in Chinese). *Chin Rural Health Service Adm* **2014**, *11*, 1384–1386.
658. Wang H, Wang YN. Analysis of Surveillance Results of Foodborne Diseases in Hedong District from 2015 to 2016 (in Chinese). *Chin J Urban & Rural Enterp Hyg* **2017**, *1*, 164–165.
659. Wang J, Sun PJ, Li Q, Fu XF. Investigation on an outbreak of multi–school norovirus acute gastroenteritis caused by bottled water (in Chinese). *Chin J School Health* **2015**, *7*, 1107–1109.
660. Wang J, Wang Y, Ding F. Disinfection and effect evaluation of a case of norovirus infectious diarrhea at the multi–campus outbreak site (in Chinese). *Chin J Disinfect* **2015**, *5*, 524–525.
661. Wang J, Wu YD, Chen SW, Zhou RX. Etiological exploration on viral diarrhea among children under 5 years old in Hangzhou during 2014 –2015 (in Chinese). *Chin J Health Lab Tech* **2016**, *6*, 877–879.
662. Wang J, Wang XQ, Liu PB. Comparison of clinical features between rotavirus and norovirus infections in hospitalized infants with acute diarrhea (in Chinese). *J Clin Pediatr* **2012**, *10*, 935–938.
663. Wang J. Investigation of an epidemic about gastrointestinal infection (in Chinese). *Modern Prev Med* **2012**, *1*, 152–153. +155.
664. Wang J. Epidemiological investigation on an outbreak of norovirus infectious diarrhea in a primary school in Jiangning District, Nanjing (in Chinese). *J Front Med* **2021**, *5*, 193–194.
665. Wang KL, Huang ZH, Pan LL, Jin HL, Luo RY, Chen HB. Investigation and analysis of a clustering event of infectious diarrhea by Norovirus type II in a kindergarten (in Chinese). *Anhui J Prev Med* **2019**, *1*, 58–61.
666. Wang KM, Ke MY, Shi H, Lin YH. Pathogenic molecular biological characteristics analysis of a suspected foodborne Norovirus gastroenteritis incident (in Chinese). *Chin J Food Hyg* **2017**, *1*, 42–46.
667. Wang LL, Lin D, Gao SH, Cai YY, Shan RQ, Zhang YY, Wang J. Analysis of Epidemiological Characteristics of Foodborne Diseases in Wenzhou City (in Chinese). *Prev Med* **2021**, *3*, 306–308.
668. Wang M, Mao YX, Chen XW, Li L, Wang T. A foodborne norovirus–born outbreak field investigation in one factory of Zhongshan City (in Chinese). *Chin J Dis Control & Prev* **2017**, *2*, 209–211.
669. Wang MH, Guo LM, Ling SQ. Investigation on An Outbreak of Diarrhea Infected with Norovirus in A Kindergarten (in Chinese). *Parasitol & Infect Dis* **2020**, *1*, 41–44.
670. Wang M. Epidemiological analysis of norovirus outbreaks in Liwan District, Guangzhou, 2016–2017 (in Chinese). *Pract Prev Med* **2018**, *12*, 1511–1513.
671. Wang ML, Wang W, Yun SY, Bai Y, Ren LJ, Bai XX, Liu CW, Bao CX, Su LY, Ma XC. Investigation of an outbreak of foodborne illness caused by norovirus GII.17 (in Chinese). *Dis Surveil* **2020**, *2*, 172–176.
672. Wang P, Li ZM, Xie YQ, Pan SX, Li M, Liu J, Lin X, Ou XC. Effect of Norovirus on Blood Physiological Index in Mental Patients with Infectious Diarrhea (in Chinese). *Lett Biotechnol* **2016**, *2*, 254–256.
673. Wang QM, Zhang JY, Zhang YY, Xing C. Surveillance results of foodborne diseases in Shaoxing (in Chinese). *Prev Med* **2019**, *7*, 712–714. +718.
674. Wang ST, Shi Y, Wang SM, Li XY, Zhang XH. Analysis of epidemiological characteristics of food borne diseases in Shanxi, 2016 (in Chinese). *Chin J Public Health Manage* **2018**, *3*, 328–331.
675. Wang SL, Chen Y. Etiological analysis of infectious diarrhea in summer in the watery township of Zhejiang (in Chinese). *Modern Prev Med* **2011**, *9*, 1707–1708. +1712.
676. Wang SY, Yu KM, Peng J, Chen H, Wang XY, Hu JF, Ye JJ. An outbreak investigation on imported norovirus gastroenteritis in a hospital (in Chinese). *Prev Med* **2016**, *2*, 136–139.
677. Wang TY, Yang H, Wang Y, Wan R. Disposal of the first large scale Norovirus infection at Beijing Capital Airport (in Chinese). *Chin J Front Health & Quarantine* **2018**, *3*, 226–228.
678. Wang TJ, Liu SY, Liao XH, Gan XQ, Zhang WW. Investigation and analysis of a norovirus outbreak (in Chinese). *Anhui J Prev Med* **2020**, *1*, 52–54.
679. Wang TY. Analysis on epidemiological characteristics of Norovirus infection among diarrhea cases in sentinel hospitals in Beijing, 2017 (in Chinese). *Chin J Food Hyg* **2019**, *3*, 250–254.
680. Wang WR, Xu HR, Xu SH, Chang CY. Analysis on Surveillance of Viral Diarrhea, Jinan City, 2011 (in Chinese). *Prev Med Tribune* **2013**, *6*, 441–442. +445.

681. Wang WY, Li XF, Liao Y. Clinical Characteristics of Children with Norovirus Gastroenteritis in Mianyang Region (in Chinese). *Chin J Obstet & Gynecol & Pediatr (Electronic E)* **2011**, *6*, 579–581.
682. Wang WQ, Liu D, Zhao B, Fu HQ, Zhang ZK, Yu JX, Ye CC, Xue CY, Zhu WP, Zhu LY, et al. Epidemiological and etiological surveillance on infectious diarrhea in Pudong New Area, Shanghai, 2013–2017 (in Chinese). *Chin J Epidemiol* **2020**, *3*, 417–422.
683. Wang WL, Tian T, Gu LN·TED, Liang JH, Ai YRT·MMT, Chen XY, Liu ZL. An analysis of active surveillance of food–borne diseases in Xinjiang from 2013 to 2016 (in Chinese). *Bull Dis Control & Prev (China)* **2017**, *5*, 23–26.
684. Wang WX, Xie JH, Zhang Q. Detection and analysis of norovirus infected diarrhea in Lishui area from 2017 to 2019 (in Chinese). *Chin J Health Lab Tech* **2021**, *6*, 745–747.
685. Wang X, Zhang WW, Liu JY, Li LQ, Li L, Zhao JY, Shen XJ. Etiological study on viral diarrhea among infants and young children in Kaifeng: 2012–2014 (in Chinese). *Modern Prev Med* **2016**, *11*, 1939–1943.
686. Wang X, Zhang WW, Zhang J, Wang P. Epidemiological characteristics and influencing factors of norovirus diarrhea in Kaifeng City in 2015 (in Chinese). *Jiangsu J Prev Med* **2017**, *4*, 425–426.
687. Wang X, Zhao YQ, Meng W, Zhang WW. Epidemiological characteristics and etiological analysis of foodborne diseases in Kaifeng City in 2015–2016 (in Chinese). *Henan J Prev Med* **2018**, *5*, 397–399.
688. Wang XD, Zhao YJ, Xu YH. With infectious diarrhea caused by water pollution, Norovirus outbreak investigation (in Chinese). *J Med Pest Control* **2017**, *9*, 971–973.
689. Wang XQ, Wang J. Investigation on a clustered outbreak of norovirus gastroenteritis in a kindergarten (in Chinese). *Prev Med* **2015**, *10*, 1061–1063.
690. Wang XY. Investigation of Virus Diarrhea Incidence in the Elderly (in Chinese). *Geriatrics & Health Care* **2017**, *4*, 333–335.
691. Wang XY. Analysis of clinical characteristics of 13 patients with norovirus infection (in Chinese). *Special Health* **2017**, *15*, 191–192.
692. Wang X, Shi LM, Zhang HY, Guo BF, Xie GX, Ding J. Etiological analysis on the epidemic situation of Norovirus GII.4/Sydney variant infection in Nanjing (in Chinese). *Chin J Health Lab Tech* **2014**, *24*, 3501–3504.
693. Wang YX, Shen XT, Wang CX, Chen YF, Zhang JL, Zhao XT, Liu JY, Wu QS. Analysis of the duration of Norovirus shedding among infected kindergarten children during an outbreak (in Chinese). *Chin J School Health* **2020**, *5*, 762–764.
694. Wang YF, Zhou YJ, Zhu S, Yang H. Analysis on surveillance results of foodborne diseases in Guizhou province from 2015 to 2017 (in Chinese). *Modern Prev Med* **2019**, *4*, 723–727.
695. Wang YB, Zhang XM. Analysis on the results of foodborne disease surveillance in Baiyin City, Gansu Province, 2015 (in Chinese). *Chin J Food Hyg* **2017**, *4*, 488–491.
696. Wang YH. Epidemiological status and clinical characteristics of norovirus infection in patients with diarrhea (in Chinese). *Our Health* **2021**, *15*, 56–57.
697. Wang YY, Zhao J, Xiao GY, Wang JJ, Ma XM, Li J. Monitoring and analysis of food borne diseases during 2016–2017 in Fengtai District, Beijing (in Chinese). *Chin J Health Lab Tech* **2019**, *4*, 490–493.
698. Wang YX, Li DD, Jin Y, Zhang Q, Wang H, Kong XY, Li YN, Duan ZJ. Molecular epidemiological and clinical feature of human calicivirus and adenovirus among children with diarrhea less than 5 years old from 2010 to 2011 in Lanzhou, Gansu province (in Chinese). *Chin J Exp & Clin Virol* **2012**, *1*, 8–10.
699. Wang Y. Molecular epidemiological characteristics of norovirus infectious diarrhea in Harbin from 2014 to 2016 (in Chinese). *Int J Viro* **2019**, *2*, 116–119.
700. Wang YP, Yang SX, Rong JR, Lu L, Zhang LJ. Analysis on Surveillance Results of Foodborne Diseases in Jiangdong District (in Chinese). *Prev Med* **2016**, *10*, 1023–1025. +1028.
701. Wang YY, Zhang S. The pathogenic characteristics of Rotavirus and Norovirus diarrhea surveillance in Shunyi district of Beijing (in Chinese). *Int J Viro* **2015**, *4*, 274–276.
702. Wang Y, Shi C, Yu L. Analysis of active surveillance of foodborne diseases in a sentinel hospital in Qinhuangdao from 2016 to 2019 (in Chinese). *J Food Safety & Quality* **2020**, *9*, 3029–3034.
703. Wang YS, Zhang J, Li XQ, Yu J, Zhou F. Epidemiological characteristics and etiological surveillance of virus diarrheal among children under 5 years old, Henan Province (in Chinese). *Modern Prev Med* **2015**, *23*, 4290–4293.
704. Wang ZY, Zhou HH, Zheng TT. Active surveillance results of foodborne diseases in sentinel hospitals, 2015–2018 (in Chinese). *Chin J Health Lab Tech* **2020**, *20*, 2546–2548. +2551.
705. Wei YY, Sun YM, Sun PY, Gao ZY. An outbreak of norovirus gastroenteritis in a hospital ward (in Chinese). *Modern Prev Med* **2015**, *18*, 3399–3402.
706. Wei GY, Zong J. Investigation on an outbreak of acute gastroenteritis caused by norovirus GII infection in a kindergarten institution (in Chinese). *Appl Prev Med* **2019**, *4*, 302–304.
707. Wei KF, Liu XF, Liu DP, Chang QE, Xiang JP, Wang P. Prevalence of group A rotavirus and norovirus infection among children hospitalized with diarrhea in Wuwei of China (in Chinese). *Chin J Viral Dis* **2014**, *2*, 137–140.
708. Wei X. Investigation and analysis of an outbreak caused by infective diarrhea of Norovirus (in Chinese). *Gansu Med J* **2019**, *3*, 267–268. +275.
709. Wei XH. Analysis of foodborne disease surveillance results in Decheng District from 2018 to 2019 (in Chinese). *The Med Forum* **2020**, *23*, 3374–3375.
710. Wen Y, Wen YQ, Tang X, Peng XJ, Xu L. An Epidemiological Survey on The Outbreak of Viral Gastroenteritis in a Restaurant (in Chinese). *Chin Primary Health Care* **2018**, *8*, 38–40.
711. Wen YM, Liao HD, Lai ZJ. Investigation of clustering epidemic caused by norovirus in a welfare center for children (in Chinese). *Chin J School Doct* **2019**, *8*, 574–576.

712. Weng XQ. Molecular epidemiological characteristics of viral diarrhea in children (in Chinese). *Compr Med* **2017**, *2*, 238.
713. Wu HM. Epidemiological investigation and analysis of an outbreak of norovirus infection in a university (in Chinese). *Contemp Med Sym* **2020**, *19*, 13–14.
714. Wu BS. Detection of novel norovirus in children with diarrhea in Fujian province (in Chinese). *Int J Viro* **2018**, *6*, 389–392.
715. Wu BS, Huang ZM, Ou JM, Qi XQ, Huang YW, Weng YW. New recombinant norovirus strain GII.P16/GII.2 caused an outbreak of viral gastroenteritis in Fujian Province in winter 2016 (in Chinese). *Chin J Zoonoses* **2017**, *9*, 805–808. +813.
716. Wu BS, Weng YW, Huang ZM, He WX. A novel variant of norovirus GII.17 caused an outbreak of viral gastroenteritis in Fujian Province from 2014 to 2015 (in Chinese). *Chin J Virol* **2017**, *3*, 320–324.
717. Wu GF, Zhang AB. Investigation report on a clustered incident situation of norovirus gastroenteritis in a primary school (in Chinese). *Anhui J Prev Med* **2018**, *3*, 210–212.
718. Wu HX, Pu ZW, Yao ZC, Yang CJ, Yang FY, Zhang X, Yao J. Investigation on an outbreak of norovirus infectious diarrhea in schools due to drinking water contamination (in Chinese). *Pract Prev Med* **2016**, *8*, 977–979.
719. Wu JW, Zhou K, Cheng WJ, Wang WK, Tu JL. Epidemiological Investigation on an Outbreak of Norovirus in a Primary School (in Chinese). *Chin Primary Health Care* **2020**, *11*, 87–89.
720. Wu J. Analysis of pathogenic detection results of viral diarrhea patients in Qixia District of Nanjing city in 2014 (in Chinese). *Electron J Clin Med Literature* **2015**, *23*, 4919–4920.
721. Wu J. Research on the infection situation of pathogenic microorganisms of foodborne diseases in the north of Nanjing in 2016 (in Chinese). *Int J Lab Med* **2018**, *1*, 113–116.
722. Wu J, Zhou J, Yang WW, Wang H. Analysis of surveillance results of foodborne diarrheal disease cases in a tertiary hospital in Nanjing from 2017 to 2018 (in Chinese). *Int infect Dis (Electron E)* **2019**, *2*, 58–59.
723. Wu MX. Epidemiology of a clustered outbreak of school infectious diarrhea caused by norovirus GII (in Chinese). *Med Diet & Health* **2018**, *6*, 120.
724. Wu P, Zhang Q, Qin YP, Gao C, Su RX, Zhang HY. Clinical characteristics and prevention and control measures of norovirus diarrhea in adults (in Chinese). *Chin J Prev Med* **2011**, *12*, 1124–1125.
725. Wu QD, Sun G, Zhou L, Kong WR. Investigation on an outbreak of acute gastroenteritis in a middle school in Liyang (in Chinese). *China Rural Health* **2018**, *148*, 37.
726. Wu SH, He JT, Peng CY, Li XY, Xu LH, Wang XJ. Diarrhea pathogens in rotavirus negative infants in Hangzhou (in Chinese). *Chin J Lab Med* **2019**, *8*, 688–693.
727. Wu SX, Guo S, Chen JX. An investigation and analysis on an outbreak of norovirus diarrhea in Longyan City (in Chinese). *Chin J Urban & Rural Enterp Hyg* **2012**, *5*, 84–86.
728. Wu W, Zhang HB, Zhang HL, Yang H, Chen HL, Shu BH, He YQ. The Genotype of Norovirus in Shenzhen, 2010 (in Chinese). *Chin J Virol* **2012**, *3*, 219–223.
729. Wu WQ. An outbreak of Norovirus gastroenteritis in a school of Nanjing (in Chinese). *Modern Med J* **2018**, *12*, 1360–1363.
730. Wu XY, Chen L. An investigation on an outbreak of acute gastroenteritis caused by Norovirus (in Chinese). *Modern Prev Med* **2015**, *23*, 4398–4399. +4405.
731. Wu XF, Chen LP, Xu DS, Ji L, Shen YH, Zhu XJ. Analysis of norovirus detection results in sporadic acute gastroenteritis cases in Huzhou city (in Chinese). *Prev Med* **2017**, *7*, 714–716.
732. Wu XM. A foodborne gastroenteritis outbreak caused by Norovirus GII.2 (in Chinese). *Chin J Food Hyg* **2021**, *2*, 234–237.
733. Wu Y, Li MC, Li D, He CH. An acute gastroenteritis outbreak caused by norovirus in a middle school in Hubei province, China (in Chinese). *Int J Viro* **2018**, *4*, 241–244.
734. Wu Y, Liu K, Li SC, Guan XH, Xing XS. Investigation and analysis of an outbreak of norovirus acute gastroenteritis (1) (in Chinese). *Appl Prev Med* **2018**, *1*, 72–73. +76.
735. Wu Y, Yao ML, Mao AL, Huang JG, Guan XH, Li D. Investigation of an outbreak of Norovirus infection in a kindergarten (in Chinese). *China Trop Med* **2018**, *5*, 509–511.
736. Wu YL, Qiao XF, Zhang QH. Investigation of an outbreak of vomiting and diarrhea in a school cluster (in Chinese). *For all Health* **2017**, *2*, 19.
737. Wu ZC, Wang JS, Li XX, Gao WL, Liu MH. Investigation and analysis of an outbreak of infectious diarrhea in a hospital (in Chinese). *Anhui J Prev Med* **2015**, *4*, 294–296.
738. Wu ZY, Zhang DY, Lei YL. Investigation of an aerosol–transmitted school norovirus outbreak (in Chinese). *Chin J School Health* **2012**, *2*, 244–245.
739. Wu XJ. Epidemiological Investigation and Analysis of the Epidemic Situation of Infectious Disease in Gongan County (in Chinese). *China Health Industry* **2015**, *23*, 156–158.
740. Wu YT, Wang X, Shi MD, Sun YF, Wang F. Pathogenic factors of foodborne diseases in Wuhan, 2013–2017 (in Chinese). *Modern Prev Med* **2019**, *7*, 1199–1202. +1237.
741. Wu ZS, Hong L, Ma T, Hua LH, Zhang HY. Epidemiological investigation and treatment of an infectious diarrhea (in Chinese). *Jiangsu J Prev Med* **2014**, *5*, 62–63.
742. Xia GH, Pan HH, Xie Y. Epidemic analysis of a norovirus infectious diarrhea outbreak via contaminated rural centralized water supply system in Jiangxi province (in Chinese). *Pract Prev Med* **2019**, *10*, 1234–1236.
743. Xia YP, Dong WB, Jiang JL, Chen J. Investigation and reflection on the norovirus infection in primary schools with contact transmission (in Chinese). *Chin Rural Health Service Adm* **2018**, *5*, 606–608.
744. Xia ZF, Yang MH. Investigation of an outbreak of norovirus infection (in Chinese). *Shanghai J Prev Med* **2012**, *10*, 554–555.

745. Xiao SJ, Zhou Y, Lian YF, Ruan F. Investigation on outbreak of GII.17 norovirus caused by food contamination by a kitchen worker (in Chinese). *Strait J Prev Med* **2017**, *1*, 86–88.
746. Xiao DY, Li D, Zhou GH, Tan XL, Du XY, Ran L, Li Q. An investigation on food–borne outbreak of norovirus infection in kindergartens in Chongqing (in Chinese). *J Trop Med* **2018**, *10*, 1364–1367.
747. Xiao L, Guo M, Xia TJ, Chen ZL, Chen ZQ, Qiu ZG, Wang JF, Zhang B, Yang D, Wang XW, et al. Investigation of Norovirus Infection in Infants and Young Children with Viral Diarrhea in Tianjin (in Chinese). *J Environ & Health* **2014**, *9*, 806–808.
748. Xiao Y, Feng WH, Sha D, Guan HX, Wang JM. Analysis of molecular epidemiological characteristics of norovirus in diarrhea group in Wuxi in 2013 (in Chinese). *Chin J Health Lab Tech* **2016**, *7*, 1006–1009.
749. Xie CW, Tang W, Du CS, Tian ZP. An outbreak of Norovirus acute gastroenteritis in a special education school in Guangzhou city (in Chinese). *J Dis Monitor & Control* **2017**, *1*, 30–32.
750. Xie CY, Hu Q, Cui HY, Chen RB. Analysis on pathogens and intestinal viral infection monitoring results of infantile infectious diarrhea (in Chinese). *Lab Med & Clin* **2014**, *2*, 168–170.
751. Xie HY, Ye HY, He L, Zhou Y, Shi LM, Zhang XG. Etiological analysis of infant viral diarrhea in Beilun area, Ningbo (in Chinese). *Chin J Health Lab Tech* **2020**, *1*, 41–42. +57.
752. Xie HP, Li QY, Kang Y, Geng JM, Lu EJ, Wu YJ, Di B. Epidemiological Study of a Diarrhea Outbreak Caused by Norovirus in Guangzhou (in Chinese). *J Prev Med Inform* **2010**, *8*, 669–671.
753. Xie SR, Shen HW, Zhang JJ, Du T, Zhang Y, Yang M. Etiology characteristics and epidemiological analysis of viral diarrhea in Shenzhen, 2014–2015 (in Chinese). *China Trop Med* **2017**, *7*, 686–690.
754. Xie XB, Yu DX, Zhou ZJ, Ren RW, Fei F, Xiong W, Qiu D, Liu WY, Yan HC. Investigation of public health emergencies in troops caused by norovirus and Staphylococcus aureus infection (in Chinese). *J Prev Med Chin PLA* **2020**, *8*, 5–8.
755. Xie YX. Analysis on the status and characteristics of norovirus infection in infants with acute gastroenteritis in a county hospital (in Chinese). *Gansu Med J* **2021**, *6*, 496–498.
756. Xie YQ, Wang P, Yu YM, Lin XT. An outbreak of infectious diarrhea in hospital caused by mental patient infected with norovirus (in Chinese). *J Med Pest Control* **2016**, *9*, 981–983.
757. Xie Y, Liu YY, Yu QL, Zhao WN, Su T, Qi SX. Pathogenic characteristics of infectious diarrhea outbreak in a community in Hebei province (in Chinese). *Hebei Med J* **2018**, *17*, 2688–2690.
758. Xie Y, Yu QL, Liu YY, Zhao WN, Su T, Qi SX. Investigation on a norovirus induced infectious diarrhea outbreak in a school (in Chinese). *J Med Pest Control* **2020**, *6*, 555–557.
759. Xing Y, Jiang C, Hua WY, Liu F, Zhao Z, Ding YJ, Wang L, Li J. Survey on a public health emergency event caused by norovirus (in Chinese). *Chin J Epidemiol* **2017**, *9*, 1174–1178.
760. Xing YF, Sun YL, Wang CX, Gong CB, Dong FG. Active surveillance and results analysis of foodborne diseases in Yantai from 2013 to 2017 (in Chinese). *Chin J Health Lab Tech* **2019**, *6*, 747–750.
761. Xiong FY, Ye SQ, He J. Etiological characteristics and influencing factors of acute gastroenteritis in children in Jiujiang City (in Chinese). *Appl Prev Med* **2016**, *4*, 318–320. +323.
762. Xiong XS, Liu CX, Hu YY, Li XY. Epidemiological condition investigation of diarrhea virus infection among children in Wenzhou area from 2014 to 2016 (in Chinese). *Chin J Health Lab Tech* **2017**, *15*, 2257–2259.
763. Xu J. Analysis of infection status of norovirus and rotavirus diarrhea in 2021 children with diarrhea (in Chinese). *Lab Med & Clin* **2021**, *12*, 1743–1745.
764. Xu D, Gan P, Jiang L, Zhang LF, Li SC. Norovirus screening and genotyping analysis among infants with diarrhea in Nan Shan district of Shen Zhen city (in Chinese). *Int J Lab Med* **2014**, *6*, 663–666.
765. Xu D, Luo L, Tan W, Ban B, Shen HQ, Lu XD. Multiplex PCR to identify infant diarrhea common virus (in Chinese). *J Trop Med* **2012**, *2*, 184–186. +216.
766. Xu FF, Jin XX, Chen MF, Cai Y, Xu JY. Surveillance results of foodborne diseases in Jiangbei District (in Chinese). *Prev Med* **2018**, *11*, 1163–1166.
767. Xu JR, Zheng GY, Gao L, Chen L. Survey on an outbreak of acute gastroenteritis caused by norovirus type II in a school (in Chinese). *Chin J Public Health Manage* **2018**, *1*, 48–51.
768. Xu LX, Lei YL, Yu KD, Ji BL, Lin Q, Wu J. Detection and analysis of norovirus from patients with food borne diarrhea in Lishui (in Chinese). *Chin J Health Lab Tech* **2016**, *23*, 3450–3452.
769. Xu Q, Zhang YJ. Investigation on Norovirus Infection in Acute Diarrhea Cases in Yangzhou City in 2013 (in Chinese). *Jiangsu Health Care* **2014**, *6*, 7–8.
770. Xu RQ. Epidemiological analysis on Norovirus diarrhea in children of Guangzhou from 2016 to 2018 (in Chinese). *Chin J Clin Lab Manage(Electron E)* **2021**, *1*, 29–33.
771. Xu SM, Xie ZC, Xie YH, He WT, Xie ZG, Tan Y, Zhu JG. Study on the high risk factor of an infectious diarrhea outbreak in a Sino–Vietnamese border village (in Chinese). *Appl Prev Med* **2013**, *5*, 272–275.
772. Xu Y, Wang AN, Zhang WZ, Shi W, Ji GQ. Investigation of an Outbreak of Infectious Diarrhea Caused by Norovirus (in Chinese). *J Med Inform* **2018**, *15*, 113–114. +117.
773. Xu ZQ, Chen JJ, Zhu SQ, Zhang WJ, Hu YW, Qian FX. The epidemiology of adults acute viral gastroenteritis in Shanghai Changning district from 2010 to 2013 (in Chinese). *Chin J Postgraduates Med* **2016**, *5*, 419–424.
774. Xu F, Song CL, Li L, Zhong HM, Yuan JC, Zhu JM. Investigation on an outbreak of acute norovirus gastroenteritis in a geriatric nursing hospital (in Chinese). *Occup & Health* **2012**, *6*, 731–733.
775. Xu H, Fu HP, Zhao Z, Guo MJ, Cao Y. Analysis of an epidemic outbreak of gastroenteritis caused by Norovirus (in Chinese). *Chin J Health Lab Tech* **2016**, *10*, 1484–1486.

776. Xu HJ, Jiao JD, Tan WW, Li C. Analysis on surveillance results of foodborne diseases from sentinel hospitals in Yixing City of Jiangsu Province from 2016 to 2017 (in Chinese). *J Med Pest Control* **2019**, *7*, 638–641.
777. Xu JF, Xu H, Song YS, Xue Y, Shao SH, Shen HX. Molecular epidemiological characteristics and genotype analysis of norovirus in Zhenjiang region (in Chinese). *Chin J Clin Lab Sci* **2017**, *11*, 857–861.
778. Xu JF, Xu H, Xue Y, Zhou Y, Shao SH, Shen HX. Molecular epidemiological analysis on 3 outbreaks of GII.17 norovirus (in Chinese). *Chin J Health Lab Tech* **2016**, *23*, 3423–3425. +3428.
779. Xu J, Shi X, Shu C, Leng Y, Cao B, Sun W. Molecular characteristics of norovirus in 4 outbreaks of gastroenteritis in Heilongjiang province in2016 (in Chinese). *Chin J Exp & Clin Virol* **2018**, *1*, 57–61.
780. Xu J, Shu C, Yang M, Hua H, Su Y, Liu YF. Epidemiological study of viral diarrhea in Harbin in 2011 (in Chinese). *Chin J Public Health Manage* **2012**, *3*, 301–302.
781. Xu LQ. Etiological Analysis on Infectious Diarrhea of Children in Shiyan City Area From 2013 to 2014 (in Chinese). *J Hubei Univ Med* **2015**, *5*, 472–475.
782. Xu SJ, Ren Y, Chen HB, Lai XL, Zhang HL, Luo JW, Man YX, Hao XQ, Li JZ, Liu JX, et al. Molecular epidemiology of gastroenteritis caused by norovirus type GI.5 (in Chinese). *J Trop Med* **2019**, *12*, 1575–1577.
783. Xu XC, Chen XL, Huang YH, Mai L. Surveillance analysis of rotavirus and norovirus in infants and young children with diarrhea in Yangjiang City (in Chinese). *Strait J Prev Med* **2015**, *5*, 96–97.
784. Xuan LF. Investigation and handling of an outbreak of enterovirus infectious diarrhea (in Chinese). *J Med Pest Control* **2014**, *1*, 103–104.
785. Xue CY, Zhu WP, Pan LF, Wang YP, Fu HQ, Cui C, Lu L, Sun Q, Xu B. Epidemiological analysis on the norovirus detection based on diarrhea syndromic surveillance in Pudong, Shanghai, 2012–2016 (in Chinese). *Chin J Infect Dis* **2017**, *11*, 688–692.
786. Yan GB, Peng H, Shu GL, Li DX, Wang WJ, Xu DQ, Yan HQ. Etiological analysis of two acute gastroenteritis outbreaks caused by norovirus GII.2 (in Chinese). *Int J Viro* **2017**, *6*, 384–388.
787. Yan X, Wang Y, Dong JS, Liu LJ, Luo YS, Bai X. Norovirus monitoring in fecal samples of some patients with diarrhea in Qinhuangdao (in Chinese). *J Food Safety & Quality* **2018**, *19*, 5064–5068.
788. Yan YX, Zhou YK, Zhang HF, Wang F, Jin M, Zhang Q, Kong XY, Li HY, Li JX, Liu N, et al. Molecular Epidemiology of norovirus infection among hospitalized children under 5 years with diarrhea in Lanzhou, 2015–2018 (in Chinese). *Int J Viro* **2020**, *1*, 53–56.
789. Yan W, Xu DS, Wu XF, Chen LP. Surveillance and analysis of foodborne diseases in Huzhou, 2017 (in Chinese). *Chin J Health Lab Tech* **2019**, *13*, 1613–1615. +1618.
790. Yan Y. Genotype Features of Norovirus Associated with Acute Gastroenteritis through Sentinel Surveillance in Guizhou Province, 2011 (in Chinese). *Chin J Virol* **2013**, *1*, 51–55.
791. Yan Y, Guo J, Jiang WJ, Tian KC, Li SJ, Hu J, Tang GP, Wang DM. A preliminary study on Norovirus variant for GII.4 in Guiyang regions (in Chinese). *Chin J Microbiol & Immunol* **2012**, *7*, 606–609.
792. Yan CY, Luo C, Zheng DK, Lang ZK, Wang HQ. Outbreak investigation of foodborne norovirus gastroenteritis in Wanzhou District, Chongqing (in Chinese). *Strait J Prev Med* **2017**, *3*, 77–79.
793. Yan W, Zhang J, Wu J, Sun Z. Epidemiological characteristics and pathogenic surveillance of viral diarrhea in Binzhou city from 2017 to 2018 (in Chinese). *J Trop Med* **2020**, *2*, 264–266. +278.
794. Yan F, Zhang JB, Zhang XH, Luo KW, Zhao SL, Wu ZJ, Li JH. Pathogenic monitoring of infectious diarrhea in Longshan County, 2015–2016 (in Chinese). *Pract Prev Med* **2018**, *12*, 1462–1465.
795. Yang AQ, Wang Y, Wu YP, Xu YF, Wang ST, Yang HB. Investigation and analysis of an outbreak infectious diarrhea caused by norovirus in meals on wheel in hospitals of Zibo in 2016 (in Chinese). *Modern Prev Med* **2018**, *14*, 2538–2541.
796. Yang CB, He YF, Zhang YC, Chen WW, Ren SH, Zhang XP. An Outbreak of Norovirus Related Acute Gastroenteritis in High School (in Chinese). *J Prev Med Inform* **2018**, *2*, 219–221.
797. Yang C. Investigation on a Norovirus Infectious Diarrhea (in Chinese). *J Dis Monitor & Control* **2017**, *7*, 548–549.
798. Yang GM, Ni ZM, Xu DG, Xi Y, Fang YZ. Analysis on infectious diarrhea outbreak disposal and related factors in a welfare center for handicapped (in Chinese). *Modern Prev Med* **2015**, *22*, 4111–4112. +4121.
799. Yang H, Li XQ, Yu J, Wang XH, Gao RF, Lu YX. Analysis of pathogens of viral diarrhea in children under 3years old in Zhengzhou (in Chinese). *J Community Med* **2020**, *9*, 631–633.
800. Yang H, Chen YJ, Jin YJ, Liu Q, Li JM, Gan LP. Analysis of the surveillance results of infectious diarrhea spectrum in Longgang district of Shenzhen during 2010–2014 (in Chinese). *Chin J Health Lab Tech* **2016**, *24*, 3584–3587.
801. Yang JX. Investigation and analysis of an incident of norovirus gastroenteritis with Staphylococcus aureus contamination (in Chinese). *J Front Med* **2019**, *15*, 224–225.
802. Yang JC, Li JX. Investigation on a cluster of norovirus infection in kindergarten (in Chinese). *Strait J Prev Med* **2016**, *5*, 64–65.
803. Yang JH, Qin HX, Pan YY, Peng ZY, Liang SS. Analysis of epidemiological characteristics of other infectious diarrhea in Nanhai district of Foshan city (in Chinese). *Bull Dis Control & Prev (China)* **2020**, *2*, 41–43.
804. Yang J, Li XF, Geng DL, Liu QH, Mai ZLM. An Outbreak of Norovirus Diarrhea in A Boarding School (in Chinese). *J Prev Med Inform* **2018**, *12*, 1544–1547.
805. Yang L, Duan DS, Sun T, Liu SQ, Sun YB. Analysis on the epidemiological characteristics of Norovirus infection in foodborne diarrhea cases in Jinan City, 2014–2016 (in Chinese). *Chin J Food Hyg* **2018**, *3*, 264–269.
806. Yang M, Du T, Xie SR, Li Y, Chen WX. Analysis of epidemiological characteristics of foodborne diseases among residents in Futian community, Shenzhen (in Chinese). *J Public Health & Prev Med* **2017**, *1*, 118–119.
807. Yang M, Xu J, Shu C, Fan XS, Hua H, Liu YF, Li BX, Su Y. Investigation on pathogens of viral diarrhea in Harbin in 2010 (in Chinese). *Chin J Public Health Manage* **2011**, *6*, 627–628.

808. Yang RX, Zhang SH, Zhang HL, Lin LQ, Dai CW. An epidemiological investigation on an outbreak of noroviral gastroenteritis caused by GII.3 strains in a kindergarten (in Chinese). *J Trop Med* **2019**, *12*, 1561–1563.
809. Yang RS, Zuo SW, Liu HY, Ni ZL, Liu ML. Surveillance of Viral Diarrhea among Children under 5 Years Old in Winter, Hongta Contry of Yuxi (in Chinese). *Chin Primary Health Care* **2016**, *5*, 22–23.
810. Yang SP, Xiu K, Su XY, Luo L, Zhang XH, Liu H, Hao YD. Investigation of norovirus outbreak in a kindergarten (in Chinese). *South China J Prev Med* **2018**, *2*, 170–172.
811. Yang SY, Chang H, Zhao Y, Lei M, Zhi XY. Epidemiological investigation and emergency response of a norovirus nosocomial infection outbreak (in Chinese). *Chin J Disinfect* **2015**, *12*, 1281–1282.
812. Yang TC, Zhao FM, Li QF, Kong ZF, Ma JM, Dong HJ. Analysis of the pathogenic surveillance results of acute diarrhea in Ningbo residents during 2011–2013 (in Chinese). *Chin J Health Lab Tech* **2016**, *4*, 559–561. +564.
813. Yang TT, Lv XZ, Liu M, Yang YP. Investigation and analysis of an outbreak of norovirus diarrhea in Binhai New Area (in Chinese). *J Community Med* **2018**, *1*, 29–30.
814. Yang XD. Analysis of pathogenic and epidemiological characteristics of patients with viral diarrhea in Jilin Province from 2017 to 2018 (in Chinese). *Clin J Diabetes World* **2019**, *12*, 199.
815. Yang XJ, Yuan YH, Huang JF, Huang HB, Du Y, Ma J, Wu YF, Xie DS. Analysis of the pathogenic surveillance results of 1982 patients with acute diarrhea (in Chinese). *China Med Herald* **2016**, *20*, 60–63.
816. Yang YN, Li HJ, Li QH. Etiological and epidemiological characteristics of viral diarrheal disease among children under 5 years old in Tongzhou District, Beijing, 2014–2016 (in Chinese). *Pract Prev Med* **2018**, *7*, 877–879.
817. Yang YL, Liao WD. Surveillance of norovirus infectious diarrhea in Meizhou city, 2013–2014 (in Chinese). *J Trop Med* **2015**, *5*, 711–713.
818. Yang YL, Tan XH, Liao WD. Outbreak of waterborne infectious diarrhea associated with Norovirus in community (in Chinese). *South China J Prev Med* **2014**, *2*, 149–153.
819. Yang YQ, Zhang XY, Fang HQ, Xu DB. Investigation, handling and inspiration of an outbreak of norovirus acute gastroenteritis in a middle school (in Chinese). *Chin J Rural Medicine & Pharm* **2016**, *24*, 66–67.
820. Yang ZW, Chen MY, Yang YF, Cen XL, Li YP, Zhen YY. Investigation of noroviral gastroenteritis outbreak in a rural community of Enping city (in Chinese). *J Trop Med* **2013**, *1*, 101–103.
821. Yao D, Chen JF, Ye W, Ou XH. Etiology and genotype features analysis of an acute Gastroenteritis outbreak associated with Norovirus GII.17 (in Chinese). *Chin J Zoonoses* **2016**, *7*, 641–643. +650.
822. Yao JX, Ma Y, Liang F. An investigation on an outbreak of norovirus diarrhea in a school (in Chinese). *J Med Pest Control* **2016**, *6*, 653–654. +657.
823. Yao JX, Shu FY, Liang F. Analysis of the etiological and epidemiological characteristics of infectious diarrhea in Jiangyin City in 2013 (in Chinese). *J Med Pest Control* **2016**, *3*, 325–327.
824. Yao J, Zhang J, Wu HX, Ma XJ, Li XJ. With the school outbreaks of norovirus infections and related sanitation investigation (in Chinese). *J Anhui Med Coll* **2017**, *6*, 8–10.
825. Yao LL, Shen XB, He P, Yang XT, Shen JQ. Monitoring of food–borne diseases from sentinel hospitals in Baoshan district of Shanghai, 2015–2017 (in Chinese). *Modern Prev Med* **2019**, *3*, 543–546.
826. Yao LL, He P, Shen XB, Yang XT, Shen JQ. Analysis on monitoring results of foodborne disease pathogens in Baoshan District of Shanghai from 2015–2018 (in Chinese). *Occup & Health* **2020**, *1*, 38–41.
827. Yao P, Fu JG, Ge YY, Li JH, Wang FM, Xu XY. Pathology research on an acute gastroenteritis outbreak in Changzhou (in Chinese). *Chin J Health Lab Tech* **2013**, *6*, 1450–1452.
828. Yao XP. Genotyping of norovirus in 614 patients with acute diarrhea (in Chinese). *Chin J Health Lab Tech* **2021**, *10*, 1204–1207.
829. Ye HY, Zhou FM, Cui DW, Wang HY, Tu LX, Xie HY, Ge TT. Distribution and clinical features of gastrointestinal virus infection in infants with acute diarrhea (in Chinese). *Chin J Clin Infect Dis* **2013**, *6*, 335–338.
830. Ye JB, Gao LD, Liu FQ, Xiao JH, Duan HY, Yue WF. Epidemiological analysis of norovirus infection outbreaks in schools in Hunan Province from 2013 to 2016 (in Chinese). *Chin J School Health* **2018**, *3*, 394–396. +400.
831. Ye SJ, Qin QC, Huang Y, Li Z, Lu CH. An investigation of Norovirus gastroenteritis outbreak in a scenic spot caused by well water contamination (in Chinese). *Dis Surveil* **2019**, *8*, 771–774.
832. Ye XM, Wu XF, Fang F, Shen SB, Ye JY, Yang CF, Lin HH. Analysis of an outbreak of norovirus in a school with multi–channel transmission (in Chinese). *Chin J School Health* **2017**, *9*, 1429–1431.
833. Ye XL, Long DL, Chen WS, Zhuo F, Huang M, Yang GQ, Mei SJ, Zhang SX, You J. Investigation on outbreak of gastroenteritis due to norovirus in a welfare center in Shenzhen (in Chinese). *Modern Prev Med* **2014**, *7*, 1317–1320. +1325.
834. Ye YH, Du XF, Jiang X, Guo BF, Jin D. Analysis of active monitoring results of foodborne diseases in sentinel hospitals in Nanjing City (in Chinese). *J Public Health & Prev Med* **2018**, *5*, 100–103.
835. Ye YH, Zhang HL, He YQ, Yang H, Yao XJ, Zhong JC. Analysis of norovirus infection status in Futian district of Shenzhen during 2012–2014 (in Chinese). *Chin J Health Lab Tech* **2015**, *21*, 3656–3658.
836. Yi M, Zhan Y, Cai T. Surveillance results and analysis of norovirus in patients with diarrhea in Dalian city (in Chinese). *China Health Care & Nutr* **2018**, *7*, 210–211.
837. Yi Y, Jiang X, Jin M, Zhang L. Molecular epidemiological analysis of acute gastroenteritis outbreak caused by norovirus (in Chinese). *Chin J Health Lab Tech* **2015**, *3*, 396–398.
838. Yi Y, Wang ZG, Zhang HH. Analysis on epidemiology and genotype of norovirus infection in Qingdao of 2010 (in Chinese). *J Med Pest Control* **2013**, *4*, 376–378.

839. Yin HM. Investigation and handling of an outbreak caused by norovirus infection (in Chinese). *Inner Mongolia J Traditional Chin Med* **2014**, *2*, 56–57.
840. Ying LH, Zhang GB, Xu HJ, Wen L, Zhu WJ, Zhang XY, Lou BL. Investigation on an outbreak of norovirus infection in a middle school (in Chinese). *Shanghai J Prev Med* **2016**, *8*, 539–542.
841. Zhao W. Etiological study of viral diarrhea in infants and young children in Hebei Province. Master, Hebei Medical University; 2011.
842. You XY, Zhou HD, Liu Y, Peng SL, Liu DF, Liu CW, Tong W. Etiologic characteristics of food–borne disease in Jiangxi, 2016–2017 (in Chinese). *Modern Prev Med* **2020**, *9*, 1678–1683.
843. Yu FY, Meng LJ, Pan F, Ma Z, Wang C, Sun Y, Zhou Y, Zhang H. Genetic characteristics of norovirus in children with acute gastroenteritis in a single center in Shanghai (in Chinese). *Chin J Microbiol & Immunol* **2020**, *10*, 792–799.
844. Yu M. Study on pathogenic microorganisms and drug resistance of infectious diarrhea in infants and young children (in Chinese). *Diet & Health care* **2019**, *20*, 17–18.
845. Yu XY, Lian ZY, Wang SC, Tong Y, Shao ZC. Investigation on a school outbreak of Norovirus in Shenyang City in 2017 (in Chinese). *Occup & Health* **2018**, *2*, 260–263.
846. Yu YH, Xia W, Shi HJ, Qiu HY. Analysis on the results of active surveillance of foodborne diseases in Zhangjiagang City, Jiangsu Province from 2017 to 2018 (in Chinese). *J Med Pest Control* **2020**, *5*, 498–501.
847. Yu GQ, Chi J, Huang YM, Lei L, Xiao JH, Zhang ZW. Surveillance of viral diarrhea in Baoan District of Shenzhen City (in Chinese). *Occup & Health* **2013**, *14*, 1764–1765. +1768.
848. Yu H, Qin M, Wei XX, Lu YY, Yang JY, Wu QR, Dong XG, Li J. Etiological identification and genotyping of the first GII.8 norovirus outbreak in Fengtai District, Beijing (in Chinese). *Pract Prev Med* **2016**, *5*, 618–620.
849. Yu ZX. Investigation and Treatment of Infectious Diarrhea Caused by Norovirus (in Chinese). *Gems Health* **2013**, *6*, 494.
850. Yu JC, Xiang YB, Jin M, Zhang Q, Cao YH, Xu W, Wang SY, Mi HY, Li L, Yang JH. Molecular Epidemiological Characteristics of Norovirus in Children under 5 Years Old in Yuxi, China, 2015–2018 (in Chinese). *Chin J Virol* **2020**, *3*, 446–454.
851. Yu KM, Wang SY, Chen H. The investigation and control measures for nosocomial norovirus gastroenteritis (in Chinese). *Chin J Disinfect* **2015**, *12*, 1220–1222. +1225.
852. Yu Y. Clinical analysis of outbreaks of norovirus infectious diarrhea (in Chinese). *Special Health* **2017**, *15*, 88.
853. Yuan JM, Zhang F, Wei Y, Ma P. Analysis of virological results of children with infectious diarrhea under 5 years old in Nantong city (in Chinese). *J Xinjiang Med Univ* **2019**, *6*, 828–832.
854. Yuan J. Investigation on continual outbreaks of norovirus caused by the Sydney2012 GII.4 strain after a school outbreak controlled in higher education mega center of Guangzhou (in Chinese). *Chin J Epidemiol* **2014**, *6*, 755–756.
855. Yuan L, Pan XP, Liu GX, Yang WC. Analysis of viral diarrhea in children under 5 years old in Xining from 2015 to 2016 (in Chinese). *J Med Pest Control* **2018**, *2*, 147–150.
856. Yuan YJ, Chen Y, Li N, Zhu JM, Ling QF. Analysis of pathogen distribution and epidemiological characteristics of viral diarrhea during 2017–2018 in Jiashan, Zhejiang Province (in Chinese). *Chin J Health Lab Tech* **2019**, *14*, 1776–1778. +1787.
857. Yuan Y, Shi YL, Fu GL, Ma WW, Chen F, Liu GZ, Cheng XL, Sun Y. Molecular characteristics of an GII.P16–GII.2 norovirus outbreak in Anhui Province (in Chinese). *Anhui J Prev Med* **2020**, *3*, 173–175. +236.
858. Yuan Y, Shi YL, Sun Y, Hu WF, Li WW, Ge YL. Genetic analysis of norovirus strains detected from sporadic cases in Anhui province, 2016–2017 (in Chinese). *Chin J Exp & Clin Virol* **2019**, *2*, 136–141.
859. Yue Y, Dai YX. Investigation on Clustered Cases of Norovirus Infection in an Early Education Center (in Chinese). *J Prev Med Inform* **2017**, *11*, 1139–1141.
860. Zhan YW, Xu BC, Lin ML. Investigation and analysis of an outbreak of norovirus infection in a middle school in Anxi County (in Chinese). *Strait J Prev Med* **2019**, *3*, 67–69.
861. Zhang AH, Guo HY. Survey on a norovirus infectious diarrhea outbreak (in Chinese). *Prev Med Tribune* **2018**, *11*, 855–856. +863.
862. Zhang C, Zhang LR, Zhang Y, Zou L, Wang RP, Gao ZY. Etiology analysis of an outbreak of acute gastroenteritis caused by norovirus (in Chinese). *Chin J Health Lab Tech* **2015**, *9*, 1394–1396.
863. Zhang DX, Li CL, Tian CF. Investigation and control of norovirus diarrhea suspected nosocomial infection outbreak (in Chinese). *Chin J Med* **2012**, *1*, 57–59.
864. Zhang DS, Lin SM, Shi YM, Lin WB. Investigation on the mixed infection of norovirus GII.4 Sydney2012 variant strain and GII.3 (in Chinese). *Strait J Prev Med* **2013**, *6*, 32–34.
865. Zhang DY. Analysis of the clinical features and etiology monitoring results of 360 children with acute infectious diarrhea (in Chinese). *Chin Pediatr Integr Tradit & West Med* **2020**, *2*, 176–180.
866. Zhang HL, Li Y, Yao XJ, Xian HX, Yang H, Luo M, He YQ. The molecular characteristics of norovirus gastroenteritis in Baoan district, Shenzhen, 2010–2011 (in Chinese). *Int J Viro* **2013**, *3*, 118–122.
867. Zhang HY, Sun BJ, Xu BL, Wang LJ. Epidemiological survey of an outbreak of acute gastroenteritis caused by norovirus G type I in primary school in Dongcheng district in Beijing (in Chinese). *Modern Prev Med* **2019**, *24*, 4502–4505.
868. Zhang HY, Xu WC, Guo JX, Sun BJ, Wang J. An epidemiological investigation on an outbreak of diarrhea caused by norovirus GII at the collective unit (in Chinese). *Capital J Public Health* **2016**, *2*, 89–92.
869. Zhang HQ, Chen B, Zhou BQ, Zhao CM, Jiao L, Luo ZH. Analysis on the risk factors of an outbreak of gastroenteritis caused by norovirus infection (in Chinese). *Pract Prev Med* **2014**, *6*, 683–685.
870. Zhang HZ, Wang S, Li JY, Shu M, Shen FJ. Etiological and epidemiological characteristics of diarrhea cases in Huangpu District of Shanghai (in Chinese). *Occup & Health* **2015**, *18*, 2504–2507.

871. Zhang HL, Zhang D, Ju JJ. Investigation of an outbreak of infectious diarrhea caused by norovirus in Lujiang County (in Chinese). *Anhui J Prev Med* **2018**, *6*, 446–447. +474.
872. Zhang JM, Chen XF, Huang ST, Cao GP, Yang RJ, Zhang FF. Surveillance of viral diarrhea in children under 5 years old in Quzhou between 2011 and 2012 (in Chinese). *Chin J Health Lab Tech* **2014**, *10*, 1468–1470.
873. Zhang J, Yang F. Correlation between infection with rotavirus and norovirus and acute gastroenteritis in children (in Chinese). *Bull Dis Control & Prev (China)* **2018**, *3*, 74–76. +83.
874. Zhang J, Liu J, Zhang BH. Analysis of surveillance results of food borne diseases in Nanning from 2012 to 2015 (in Chinese). *Chin J Health Lab Tech* **2016**, *22*, 3290–3292. +3295.
875. Zhang L, Yu MD, Li Y, Wu ZH, Yang ZY, Gao LD, Hu SX, Deng ZH, Xiao JH, Yang RD. Investigation of an outbreak of infectious diarrhea caused by Norovirus in school (in Chinese). *China Trop Med* **2015**, *11*, 1350–1354.
876. Zhang LS, Liu TT, Wang X. Influencing factors of viral diarrhea in children under 5 years old in Xicheng District, Beijing (in Chinese). *South China J Prev Med* **2019**, *6*, 565–567.
877. Zhang L, Xu LZ. Norovirus–carrying rate investigation among healthy people and patients with diarrhea of linyi in 2014 (in Chinese). *J Shandong Med Coll* **2015**, *4*, 253–256. +321.
878. Zhang LL, Li F, Wang HY. Analysis of the surveillance results of diarrhea cases in Yuhang District in 2013 (in Chinese). *Chin Rural Health Service Adm* **2016**, *1*, 65–67.
879. Zhang L, Liu Q, Zhou XM, Yan Y. Epidemiological investigation and laboratory analysis of the first case of diarrhea caused by the Norovirus in a district (in Chinese). *J Dis Monitor & Control* **2016**, *4*, 273–274.
880. Zhang MM, Ceng LZ, He QY. Investigation and analysis of an outbreak of diarrhea caused by norovirus infection (in Chinese). *Strait J Prev Med* **2013**, *3*, 37–38.
881. Zhang M, Sun CG, Chen X, Yu F, Chen Y. Pathogen spectrum analysis of infectious diarrhea in Hangzhou during 2013 (in Chinese). *Chin J Clin Lab Sci* **2015**, *6*, 469–471.
882. Zhang N. Epidemiological investigation and analysis of an outbreak of norovirus infectious diarrhea in a kindergarten (in Chinese). *China Rural Health* **2018**, *24*, 36.
883. Zhang P, Zhai P, Li Z, Jian ML. Investigation and analysis of a clustered epidemic of norovirus infected diarrhea in an agritainment resort (in Chinese). *Anhui J Prev Med* **2020**, *2*, 116–118. +145.
884. Zhang Q, Jin JH, Lin RQ. Investigation of rotavirus and norovirus infections in diarrhea children under five years old in Gongshu district of hangzhou in 2016 (in Chinese). *Chin J Health Lab Tech* **2017**, *24*, 3634–3635. +3638.
885. Zhang Q, Chi MY, Zhang KM. Investigation and Analysis of Norovirus Epidemic in a Primary School in Tianjin (in Chinese). *J Med Theory & Pract* **2020**, *3*, 484–486.
886. Zhang SH, Bai X, Jiang RP, Chen FY, Shi C, He XX, Dong XF, Shen ZX. Analysis on the epidemiological characteristics of norovirus diarrhea in Hebei province in 2015–2016 (in Chinese). *J Food Safety & Quality* **2019**, *7*, 2073–2077.
887. Zhang S, Jing HB, Ma HM, Jin M, Li Y. Characterization of norovirus in adults with acute gastroenteritis in Shunyi district of Beijing from 2014 to 2015 (in Chinese). *Chin J Exp & Clin Virol* **2016**, *6*, 526–531.
888. Zhang S, Jing HB, Ma HM, Li Y, Jin M. Molecular epidemiology of norovirus in an outbreak of acute gastroenteritis (in Chinese). *Pract Prev Med* **2017**, *1*, 15–19.
889. Zhang WS, Qiu DZ, Shi L, Hu YJ, Li J, Liu GP, Chen M. The etiological characteristics of diarrhea in Xiangyang City, 2013–2018 (in Chinese). *J Public Health & Prev Med* **2020**, *2*, 70–73.
890. Zhang WW, Feng BL, Wang JL, Han YJ, He GX. Epidemiological characteristics of diarrhea pathogens and drug resistance of diarrhea–related bacteria in diarrheal patients in Miyun district, Beijing (in Chinese). *Dis Surveil* **2021**, *4*, 381–386.
891. Zhang XY, Fang HQ, Yang YQ, Xu DB. Epidemiological investigation of an outbreak of school gastroenteritis caused by norovirus (in Chinese). *Chin Rural Health Service Adm* **2015**, *9*, 1160–1161.
892. Zhang YK, Yu DS, He JX, Liu L, Yan J. Surveillance results analysis of viral diarrhea among children under 5 years old in Pingliang, Gansu during 2013–2015 (in Chinese). *Chin J Health Lab Tech* **2017**, *23*, 3472–3474. +3477.
893. Zhang YL, Li J, Li M, Zhou P, Ma J. Composition of pathogens on viral diarrhea in Yichang (2017) (in Chinese). *J Public Health & Prev Med* **2018**, *6*, 90–93.
894. Zhang Y. Investigation on two clusters of norovirus infections in Dongli District, Tianjin (in Chinese). *Chin J Urban & Rural Enterp Hyg* **2017**, *9*, 73–74.
895. Zhang Y, Guo YB, Gao H, Liu YH. Analysis of the epidemic character and influence factors of diarrhea cases caused by nororvirus infection in Ningbo in 2015 (in Chinese). *Chin J Health Lab Tech* **2017**, *2*, 276–278. +282.
896. Zhang YL, Cao XO, Ceng HH, Chen ZM. Surveillance and Analysis of Pathogens of Viral Diarrhea in Children Under 5 Years Old in Nanhai District, Foshan City (in Chinese). *Chin Prev Med* **2013**, *5*, 384–385.
897. Zhang YL, Qian SX, Su Y, Luo L, Li YH. Surveillance results of viral diarrhea in Nanhai District of Foshan City from 2016–2018 (in Chinese). *Occup & Health* **2019**, *15*, 2053–2056.
898. Zhang YM. Investigation and Analysis of Norovirus Infection Cluster in Fangguang Primary School in Pinggu District (in Chinese). *China Health Care & Nutr* **2019**, *16*, 260.
899. Zhang YF, Liu X, Li N, Hao Q, Ma JT, Chen H, Yuan F. Surveillance analysis of foodborne diseases in Ningxia from 2015 to 2017 (in Chinese). *Bull Dis Control & Prev (China)* **2018**, *6*, 71–73.
900. Zhang YJ. Investigation and Analysis of Acute Gastroenteritis Caused by Tainted Food (in Chinese). *Value Eng* **2017**, *6*, 220–221.
901. Zhang YH, Chen Y, Liu ZY, Zhang YP. Analysis of etiology and clinical feature of children's acute diarrhea in Yinchuan (in Chinese). *Chin J Child Health Care* **2015**, *4*, 425–427.

902. Zhang Y, Zhang ZJ, Zhou SM, Xiao L, Zhang XQ, Zhu ZS, Huang YS, Ma ZY, Zou MY, Peng Q, et al. Clinical analysis on coinfection in acute gastroenteritis of children (in Chinese). *IntJ Pediatr* **2017**, *8*, 570–573.
903. Zhang Z, Chen B, Liao YX, Chen YX, Wu TS. Field epidemiological investigation of an outbreak of infectious diarrhea caused by new norovirus (in Chinese). *South China J Prev Med* **2015**, *1*, 11–15.
904. Zhang ZD, Chen X, Zhou SX, Deng JP, Fan ZX, Zhou M. An Outbreak of Norovirus in Kindergarten, Zigong, 2016 (in Chinese). *Occup Health & Damage* **2017**, *2*, 70–73.
905. Zhang ZQ. Epidemiological features of Norovirus in food–borne disease sentinel surveillance points in Hanzhong City from 2017–2018 (in Chinese). *Occup & Health* **2019**, *24*, 3338–3340. +3345.
906. Zhang ZZ, Li J, Cao JH, Li WP, Zhang HY. An analysis of influencing factors of an outbreak caused by a new Norovirus recombinant strain GII. P16–GII. 2 (in Chinese). *J Med Pest Control* **2020**, *5*, 481–483.
907. Zhang GM, Pang ZF, Lv GJ, Zhu JH, Zhang B. An investigation of norovirus outbreak in a kindergarten of Jinhua City (in Chinese). *Chin Rural Health Service Adm* **2018**, *9*, 1181–1183.
908. Zhang GB, Ying LH, Hu XX, Ying LY, Shi QW, Zhang XY. Investigation and laboratory test of a cluster epidemic of norovirus in construction sites (in Chinese). *Chin J Health Lab Tech* **2020**, *2*, 227–228. +238.
909. Zhang QH, Xu SM, Cheng SG, Hu XS, Chen X, Wang HB. Investigation report on the outbreak of norovirus infectious diarrhea in a central primary school in Huangshan City (in Chinese). *Anhui J Prev Med* **2020**, *1*, 30–33.
910. Zhang SS. Etiological monitoring results and analysis of food borne pathogens in diarrhea patients in Panjin in 2016 (in Chinese). *Chin J Health Lab Tech* **2017**, *18*, 2689–2692.
911. Zhao D. Molecular epidemiological characteristics of norovirus outbreaks in Qingdao, 2014–2016 (in Chinese). *Chin J Microbiol & Immunol* **2017**, *8*, 618–623.
912. Zhao D, Su ZL, Zhang F, Shi XY, Wang ZG. Molecular epidemiology of Norovirus infection among sporadic hospitalized adults with diarrhea in Qingdao, 2015 (in Chinese). *Chin J Exp & Clin Virol* **2017**, *2*, 104–107.
913. Zhao D, Yu WS, Zhang XY, Su ZL, Sun R, Wang ZG. Molecular epidemiology of norovirus GII.15 in Qingdao City (in Chinese). *Chin J Infect Dis* **2019**, *12*, 754–759.
914. Zhao HL, Pan F, Han L. Surveillance on foodborne disease in Daxing District of Beijing City from 2012–2015 (in Chinese). *Occup & Health* **2017**, *10*, 1332–1334. +1338.
915. Zhao JY, Shen XJ, Wang ZQ, Zhang BF, Su J, Huang XY. Pathogen spectrum analysis of viral diarrhea in children below 5 years old of Henan, 2010–2015 (in Chinese). *Modern Prev Med* **2016**, *23*, 4240–4244.
916. Zhao J, Meng XJ, Geng H, Song SL, He JL. Investigation on an outbreak of norovirus infectious diarrhea in a community (in Chinese). *Shanghai J Prev Med* **2014**, *1*, 19–20.
917. Zhao LJ, Zhao HL, Kong FM, Jiang HY. Epidemiological and pathogenic characteristics of norovirus infection in viral diarrhea in Linyi city (in Chinese). *Bull Dis Control & Prev (China)* **2019**, *3*, 13–15.
918. Zhao ML, Zhang YX, Zhao XM. Norovirus infection cluster and outbreak characteristics in Hongze District, Huaian City in 2018 (in Chinese). *Jiangsu J Prev Med* **2019**, *4*, 441–442.
919. Zhao MJ, Zhou HJ, Zhang J, Zhang LJ, Feng LZ, Chen LL. Survey on an outbreak of norovirus caused by person to person contact in a primary school (in Chinese). *Prev Med Tribune* **2018**, *2*, 112–114.
920. Zhao Q, Yong MY, Liu HM, Zhao YF. Investigation and analysis on a diarrhea breakout caused by microbial contaminated domestic drinking water (in Chinese). *J Med Pest Control* **2021**, *2*, 170–174.
921. Zhao TL. Isolation, Disinfection and Disposal Measures for Collective Diarrhea Caused by Norovirus (in Chinese). *J Baotou Med Coll* **2015**, *12*, 9–10.
922. Zhao TW, Lu GF, Wu C, Sheng MY. Analysis of the results of foodborne disease surveillance of specific pathogens in Haining City (in Chinese). *Prev Med* **2018**, *5*, 519–521.
923. Zhao WQ. Investigation on an outbreak of infectious diarrhea caused by norovirus in a school (in Chinese). *Prev Med Tribune* **2020**, *3*, 229–230.
924. Zhao WN. Epidemiological characteristics and etiological study of a water–borne outbreak of norovirus in Baoding city, Hebei province (in Chinese). *Int J Viro* **2021**, *3*, 226–230.
925. Zhao WN, Liu YY, Yu QL, Su T, Xie Y, Qi SX. Epidemiological characteristics of viral diarrhea in children under five years old in Lulong county of Hebei, 2013– 2017 (in Chinese). *Dis Surveil* **2019**, *2*, 132–136.
926. Zhao XJ, Chu T, Lv QY, Wang ZY. Results of active surveillance of food–borne diseases in Mentougou District of Beijing in 2017 (in Chinese). *Occup & Health* **2018**, *23*, 3217–3219.
927. Zhao XJ, Wang ZY, Chu T, Lv QY, Liu Y. Monitoring results of viral diarrhea among diarrhea patients in enteric disease clinics in Mentougou District of Beijing from 2015–2016 (in Chinese). *Occup & Health* **2018**, *7*, 954–957.
928. Zhao XL, Zhang KC, Yang J, Lin DY. Monitoring results analysis of viral infections diarrhea in Bao'an Shenzhen 2012 (in Chinese). *Med J Chin People's Health* **2013**, *12*, 1–3. +82.
929. Zhao XC, Zhou YH, Pan YZ, Yuan CY, Wu H. An epidemiological investigation on a suspected foodborne norovirus outbreak (in Chinese). *Modern Prev Med* **2016**, *6*, 1020–1022. +1026.
930. Zhao XQ, Shou J, Zhang R. Analysis of viral diarrhea surveillance results among infants in Hangzhou from 2012 to 2013 (in Chinese). *Chin J Health Lab Tech* **2014**, *22*, 3301–3303.
931. Zhao YL. Surveillance analysis of foodborne diseases in Changzhi City from 2018 to 2019 (in Chinese). *Compr Med* **2020***NA*, 278.
932. Zhao YN, Xiang XZ, Xia Y, Li BS, Wang ZH. An outbreak of infectious diarrhea caused by norovirus in the scenic area (in Chinese). *J Trop Med* **2018**, *5*, 697–699.

933. Zhao YL. Investigation and handling report on the outbreak of norovirus infectious diarrhea in a central primary school in Qiandeng Town (in Chinese). *Chin Community Doctors* **2019**, *16*, 173–174.
934. Zhao YQ, Yin FK. Surveillance results of foodborne diseases in Gulou district of Kaifeng city from 2013 to 2019 (in Chinese). *Henan J Prev Med* **2020**, *8*, 643–645.
935. Zhao Y, Guo YY. Analysis of the epidemiology and clinical features in diarrhea caused by a norovirus infection (in Chinese). *J Pathog Bio* **2018**, *3*, 302–305.
936. Zhen GX, Gao P. Epidemiological investigation on an outbreak of norovirus infection in a study tour in Beijing (in Chinese). *Capital J Public Health* **2020**, *6*, 318–320.
937. Zhen GX, Li Y, Ji GQ, Zhang S, Wang YY, Zhang YC, Wang M, Jing HB, Ma XC, Zhang MJ. Laboratory detection for an acute gastroenteritis outbreak caused by Norovirus and Staphylococcus aureus enterotoxins (in Chinese). *Dis Surveil* **2020**, *3*, 264–268.
938. Zheng L, Yu W, Gu LP. Detection of noroviruses from viral infectious diarrhea patients in Jiading, Shanghai (in Chinese). *Chin J Zoonoses* **2013**, *6*, 636–638.
939. Zheng SF, Yu F, Chen X, Cui DW, Yang XZ, Xie GL, Wang YY, Yu JX, Li ZJ, Chen Y. Monitoring and research on pathogen spectrum in patients with acute diarrhea from sentinel hospital of Zhejiang Province during 2009 to 2014 (in Chinese). *Chin J Prev Med* **2016**, *12*, 1084–1090.
940. Zheng WL. Investigation of norovirus infection status in diarrhea cases in sentinel hospitals in Tianjin, 2015–2016 (in Chinese). *Dis Surveil* **2019**, *1*, 48–52.
941. Zheng XY. Analysis on surveillance results of foodborne diseases of sentinel hospital in Quanzhou City, 2015–2016 (in Chinese). *Strait J Prev Med* **2018**, *3*, 40–42.
942. Zheng YP, Ma YJ, Yuan Q, Chen W, Ying HY. Investigation on diarrhea patients infected with pathogens in gastroenterology clinic (in Chinese). *Chin J Disinfect* **2016**, *6*, 579–581.
943. Zhu GXL. Molecular Epidemiological Study on Human CaliciViruses of Acute Diarrhea Patients in Zhejiang Province. Master, Zhejiang University; 2011.
944. Huang JC. Epidemiological Investigation on Norovirus Diarrhea Outbreak in A School (in Chinese). *Smart & Healthcare* **2021**, *7*, 108–110. +128.
945. Zheng YX, Fang Q, Yang YH, Lin HB. Epidemiological characteristics analysis of other infectious diarrhea in Jieyang City, Guangdong Province, 2017–2019 (in Chinese). *Henan J Prev Med* **2020**, *12*, 961–963.
946. Zhong JM, Deng KJ, Pang F, Zhang JB, Cao L. An investigation of cluster outbreak of gastroenteritis due to norovirus infection in a primary school (in Chinese). *China Trop Med* **2014**, *6*, 755–757.
947. Zhong QC, Liang CL, Li SP, Ye YE, Wen CY, Ye MJ. Detection and analysis of Dongguan town of Liaobu City, in 2013 the hospital outpatient and emergency cases of diarrhea pathogens (in Chinese). *J Dis Monitor & Control* **2014**, *5*, 281–282.
948. Zhong WL, Fu SS, Wang L, Chen XL, Zhu JZ, Chen MY, Yang YF. Investigation on an outbreak of norovirus gastroenteritis in senior psychiatric ward (in Chinese). *Med J Chin People's Health* **2012**, *13*, 1635–1636. +1642.
949. Zhong YX. Analysis of norovirus infection in diarrhea patients in sentinel hospitals for foodborne disease surveillance in Guangxi from 2013 to 2015 (in Chinese). *Appl Prev Med* **2017**, *4*, 328–330.
950. Zhou AH, Luo Y, Li G, Huang LJ. Analysis of the detection results of infectious diarrhea epidemic situation (in Chinese). *Henan J Prev Med* **2017**, *5*, 380–381. +406.
951. Zhou AM, Liu XC. Investigation and analysis on 408 cases of foodborne diseases under active surveillance in Tongchuan city (in Chinese). *Clin Res & Pract* **2020**, *23*, 10–12.
952. Zhou GY, Yang XH, Zhang WW, Zheng DS, Yang YS, Feng BL, Chen YL, Feng YM. Analysis of an outbreak of infectious diarrhea caused by norovirus (in Chinese). *Capital J Public Health* **2016**, *4*, 166–168.
953. Zhou HF, Wang Y, Jiang YQ, Shen LJ. Analysis of pathogen surveillance results of 439 cases of enteroviral diarrhea in Haining City in 2017 (in Chinese). *Chin J Rural Medicine & Pharm* **2018**, *11*, 54–55.
954. Zhou JH, Chu BB. An investigation report on a cluster of norovirus gastroenteritis in a kindergarten (in Chinese). *Prev Med* **2013**, *7*, 52–53.
955. Zhou JH, Wu HL, Yu XL. Investigation and Analysis of a Norovirus Cluster Epidemic in Qingyunpu District, Nanchang City (in Chinese). *Exp & Lab Med* **2018**, *4*, 612–613. +616.
956. Zhou LH, Zhang J, Zhang H, Lu YP, Xia Y, Chen LL. Epidemiological investigation on an aggregation epidemic of gastroenteritis caused by norovirus GII (in Chinese). *Occup & Health* **2016**, *15*, 2135–2137.
957. Zhou ML. Research on nucleotide sequence of a newly emerged pandemic norovirus GII. 4 genotype (in Chinese). *Int J Lab Med* **2017**, *2*, 231–232.
958. Zhou XH, Shu GT. Analysis of a case of responsibility investigation for an outbreak of foodborne norovirus infectious diarrhea in a university (in Chinese). *Chin J Health Insp* **2015**, *1*, 66–68.
959. Zhou XT, Li Y, Xu ZH, Jin DH, Ma ZC, Huang ZY, Ren HY, Wu TS. Epidemiological Investigation on a Waterborne Outbreak of Norovirus Gastroenteritis in a Rural Community of Shenzhen (in Chinese). *Pract Prev Med* **2011**, *2*, 236–238.
960. Zhou XH, Li H, Yang XF, Ke CW, Zhong HJ, Sun LM, Guo RN. An epidemiological study on the norovirus detected in the field environment after an outbreak of norovirus gastroenteritis (in Chinese). *Chin J Epidemiol* **2010**, *1*, 43–46.
961. Zhou XH, Zhu ZZ, Chen XL. Epidemic analysis of an acute gastrointestinal disease outbreak caused by norovirus in a school (in Chinese). *J Med Pest Control* **2017**, *8*, 857–858.
962. Zhou XM, Cai MW, Lu L. Analysis on the outbreak of norovirus infectious diarrhea in a middle school in Guangzhou in 2016 (in Chinese). *Henan J Prev Med* **2017**, *6*, 467–469.

963. Zhou YZ, Zhu CM, Chen Y, Su L. Analysis of Etiological Surveillance Results of Foodborne Diseases in Changsha City in 2015 (in Chinese). *Strait J Prev Med* **2018**, *1*, 77–79.
964. Zhou Y, Yuan ZH, Zhang WJ, Xu JF. Investigation of a norovirus outbreak suspected of aerosol transmission (in Chinese). *Jiangsu J Prev Med* **2016**, *6*, 722. +750.
965. Zhou Y, Qi L, Liang JR, Sun H, Xu BL, Wang X. Etiological study of viral diarrhea among children under 5 years of age in Dongcheng district of Beijing (in Chinese). *Int J Viro* **2017**, *2*, 119–122.
966. Zhou ZQ. Survey Report of Foodborne Disease Caused by Norovirus in Some Hotel (in Chinese). *China Health Industry* **2016**, *21*, 84–86.
967. Zhu CM. Analysis of an outbreak of norovirus infection in Yangzhou City (in Chinese). *Chin Rural Health Service Adm* **2017**, *7*, 801–803.
968. Zhu CX, Chen HB, Huang LW, Li H. Epidemiological analysis of a cluster of norovirus infection in a kindergarten (in Chinese). *Strait J Prev Med* **2019**, *4*, 88–89.
969. Zhu F, Liu LJ, Shi JP. Active surveillance results of foodborne diseases in Zhangjiagang City from 2013–2015 (in Chinese). *Occup & Health* **2017**, *14*, 1921–1924.
970. Zhu GQ, Yuan Y, Liu XF, Jiang XJ, Wang Q, Zhang LJ, Li JS, Meng L. Etiological analysis of surveillance cases of diarrhea syndrome in Gansu Province from 2009 to 2018 (in Chinese). *Chin J Dis Control & Prev* **2020**, *3*, 303–307. +347.
971. Zhu HY, Ma X, Zhang M. An Outbreak of Infectious Diarrhea Caused by Norovirus (in Chinese). *J Ningxia Med Univ* **2016**, *1*, 56–58.
972. Zhu HL. Pathogen detection results of suspected foodborne diarrhea cases in sentinel hospital of Chuzhou City from 2016 to 2018 (in Chinese). *Anhui J Prev Med* **2020**, *1*, 15–18.
973. Zhu JH, Zhang H, Shu GL, Jin WJ, Wang RQ. Analysis of the pathogenic surveillance results of acute infectious diarrhea among adults in Changping District, Beijing from 2014 to 2017 (in Chinese). *Chin J Primary Med & Pharm* **2020**, *14*, 1770–1774.
974. Zhu M, Ding CX, Huang J, Nie M. Epidemiological analysis of norovirus infectious diarrhea in from 2017 to 2018 Jiashan, Zhejiang (in Chinese). *Int J Epidemiol & Infect Dis* **2019**, *6*, 530–533.
975. Zhu TT. Epidemiological characteristics of infectious diarrhea other than cholera, dysentery, typhoid and paratyphoid and paratyphoid in Huairou District of Beijing City from 2010–2013 (in Chinese). *Occup & Health* **2015**, *10*, 1331–1333.
976. Zhu WW, Yuan S, Han XL, Zhu YQ. Epidemiological investigation and analysis of norovirus outbreak in a university (in Chinese). *J Modern Med & Health* **2018**, *10*, 1597–1598. +1600.
977. Zhu XL, Gong FW, Zhuang JY, Zhang XJ, Li L. Epidemic characteristics of Norovirus in diarrheic population in Lianyungang, 2014–2016 (in Chinese). *Modern Prev Med* **2018**, *1*, 16–18. +23.
978. Zhu X, Zhou WJ, Gao MG. Surveillance results of foodborne diseases between 2015 and 2018 in Wuxi (in Chinese). *Modern Prev Med* **2019**, *14*, 2558–2562.
979. Zhu L, Tong JX, Yao XL, Deng XQ, Lu Y. Analysis of norovirus detection of patients with acute gastroenteritis in Fushun from 2017 to 2019 (in Chinese). *Chin J Front Health & Quarantine* **2020**, *5*, 337–339.
980. Zong J, Tao RH, Pan HH. Outbreak investigation of norovirus gastroenteritis in a brick and tile factory in Jiangxi Province in 2015 (in Chinese). *Jiangxi Med J* **2017**, *9*, 931–933.
981. Zou HY, Liu LH, Chen RB, Wu J, Zhong WX, Lv CD. Pathogen spectrum analysis of viral diarrhea in children below 5 years old of Henan, 2010–2015 (in Chinese). *J Trop Med* **2016**, *11*, 1452–1454. +1461.
982. Zou YW. Analysis of Detection Results of Key Infectious Diarrhea Specimen from 2017 to 2019 In Meizhou City (in Chinese). *J Mathematc Med* **2021**, *4*, 511–513.
983. Ao YY, Yu JM, Li LL, Jin M, Duan ZJ. Detection of human norovirus GIV.1 in China: a case report. *J Clin Virol* **2014**, *61*, 298–301.
984. Cao RR, Ma XZ, Li WY, Wang BN, Yang Y, Wang HR, Kuang Y, You JZ, Zhao ZY, Ren M, et al. Epidemiology of norovirus gastroenteritis in hospitalized children under five years old in western China, 2015–2019. *J Microbiol Immunol Infect* **2021**, *54*, 918–925.
985. Chang H, Zhang L, Ge Y, Cai J, Wang X, Huang Z, Guo J, Xu H, Gu Z, Chen H, et al. A Hospital–based Case–control Study of Diarrhea in Children in Shanghai. *Pediatr Infect Dis J* **2017**, *36*, 1057–1063.
986. Chen C, Wu B, Zhang H, Li KF, Liu R, Wang HL, Yan JB. Molecular evolution of GII.P17–GII.17 norovirus associated with sporadic acute gastroenteritis cases during 2013–2018 in Zhoushan Islands, China. *Virus Genes* **2020**, *56*, 279–287.
987. Chen D, Li Y, Lv J, Liu X, Gao P, Zhen G, Zhang W, Wu D, Jing H, Li Y, et al. A foodborne outbreak of gastroenteritis caused by Norovirus and Bacillus cereus at a university in the Shunyi District of Beijing, China 2018: a retrospective cohort study. *BMC Infect Dis* **2019**, *19*, 910.
988. Chen H, Qian F, Xu J, Chan M, Shen Z, Zai S, Shan M, Cai J, Zhang W, He J, et al. A novel norovirus GII.17 lineage contributed to adult gastroenteritis in Shanghai, China, during the winter of 2014–2015. *Emerg Microbes Infect* **2015**, *4*, e67.
989. Chen SY, Feng Y, Chao HC, Lai MW, Huang WL, Lin CY, Tsai CN, Chen CL, Chiu CH. Emergence in Taiwan of novel norovirus GII.4 variants causing acute gastroenteritis and intestinal haemorrhage in children. *J Med Microbiol* **2015**, *64*, 544–550.
990. Chen Y, Li Z, Han D, Cui D, Chen X, Zheng S, Yu F, Liu J, Lai S, Yan Y, et al. Viral agents associated with acute diarrhea among outpatient children in southeastern China. *Pediatr Infect Dis J* **2013**, *32*, e285–290.
991. Cheng VC, Wong LM, Tai JW, Chan JF, To KK, Li IW, Hung IF, Chan KH, Ho PL, Yuen KY. Prevention of nosocomial transmission of norovirus by strategic infection control measures. *Infect Control Hosp Epidemiol* **2011**, *32*, 229–237.
992. Li XJ. Epidemiological Survey and Analysis of a Cluster of Norovirus Gastroenteritis Cases in an Early Childhood Education Institution (in Chinese). *Guid China Med* **2021**, *19*, 98–99. +102.

993. Shen JQ. Epidemiological survey of a clustering infection of norovirus GI infection in a community in Xiangcheng District of Suzhou in 2020 (in Chinese). *Chin J Clin (Electron E)* **2020**, *14*, 992–995.
994. Cui C, Pan L, Wang Y, Xue C, Zhu W, Zhu L, Ye C, Lu X, Song H, Fu Y, et al. An outbreak of acute GII.17 norovirus gastroenteritis in a long–term care facility in China: The role of nursing assistants. *J Infect Public Health* **2017**, *10*, 725–729.
995. Dong X, Qin M, Wang ZE, Yang X, Wu Q, Feng H, Wei X, Yu H, Li J, Li J. Should we pay attention to recombinant norovirus strain GII.P7/GII.6?. *J Infect Public Health* **2019**, *12*, 403–409.
996. Fu JG, Ai J, Zhang J, Wu QB, Qi X, Ji H, Jin M, Liu C, Wang SJ, Shan J, et al. Molecular epidemiology of genogroup II norovirus infection among hospitalized children with acute gastroenteritis in Suzhou (Jiangsu, China) from 2010 to 2013. *J Med Virol* **2016**, *88*, 954–960.
997. Gao Z, Li X, Yan H, Li W, Jia L, Hu L, Hu H, Liu B, Li J, Wang Q. Human calicivirus occurrence among outpatients with diarrhea in Beijing, China, between April 2011 and March 2013. *J Med Virol* **2015**, *87*, 2040–2047.
998. Gao Z, Liu B, Huo D, Yan H, Jia L, Du Y, Qian H, Yang Y, Wang X, Li J, et al. Increased norovirus activity was associated with a novel norovirus GII.17 variant in Beijing, China during winter 2014–2015. *BMC Infect Dis* **2015**, *15*, 574.
999. Gong XH, Wu HY, Li J, Xiao WJ, Zhang X, Chen M, Teng Z, Pan H, Yuan ZA. Epidemiology, aetiology and seasonality of infectious diarrhoea in adult outpatients through active surveillance in Shanghai, China, 2012–2016: a cross–sectional study. *BMJ Open* **2018**, *8*, 9.
1000. Guo XH, Kan Z, Liu BW, Li LL. A foodborne acute gastroenteritis outbreak caused by GII.P16–GII.2 norovirus in a boarding high school, Beijing, China: a case–control study. *BMC Res Notes* **2018**, *11*, 1.
1001. Guo Z, Huang J, Shi G, Su CH, Niu JJ. A food–borne outbreak of gastroenteritis caused by norovirus GII in a university located in Xiamen City, China. *Int J Infect Dis* **2014**, *28*, 101–106.
1002. Han J, Ji L, Shen Y, Wu X, Xu D, Chen L. Emergence and predominance of norovirus GII.17 in Huzhou, China, 2014–2015. *Virol J* **2015**, *12*, 139.
1003. Han J, Wu X, Chen L, Fu Y, Xu D, Zhang P, Ji L. Emergence of norovirus GII.P16–GII.2 strains in patients with acute gastroenteritis in Huzhou, China, 2016–2017. *BMC Infect Dis* **2018**, *18*, 342.
1004. He T, McMillen TA, Qiu Y, Chen LH, Lu X, Pang XL, Kamboj M, Tang YW. Norovirus Loads in Stool Specimens of Cancer Patients with Norovirus Gastroenteritis. *J Mol Diagn* **2017**, *19*, 836–842.
1005. He Y, Jin M, Chen K, Zhang H, Yang H, Zhuo F, Zhao D, Zeng H, Yao X, Zhang Z, et al. Gastroenteritis Outbreaks Associated with the Emergence of the New GII.4 Sydney Norovirus Variant during the Epidemic of 2012/13 in Shenzhen City, China. *PLoS One* **2016**, *11*, e0165880.
1006. Huang J, Xu X, Weng Q, Hong H, Guo Z, He S, Niu J. Serial foodborne norovirus outbreaks associated with multiple genotypes. *PLoS One* **2013**, *8*, e63327.
1007. Huang XY, Su J, Lu QC, Li SZ, Zhao JY, Li ML, Li Y, Shen XJ, Zhang BF, Wang HF, et al. A large outbreak of acute gastroenteritis caused by the human norovirus GII.17 strain at a university in Henan Province, China. *Infect Dis Poverty* **2017**, *6*, 6.
1008. Huang Z, Yao D, Xiao S, Yang D, Ou X. Full–genome sequences of GII.13[P21] recombinant norovirus strains from an outbreak in Changsha, China. *Arch Virol* **2020**, *165*, 1647–1652.
1009. Ji L, Hu G, Xu D, Wu X, Fu Y, Chen L. Molecular epidemiology and changes in genotype diversity of norovirus infections in acute gastroenteritis patients in Huzhou, China, 2018. *J Med Virol* **2020**, *92*, 3173–3178.
1010. Jia L, Zhang Y, Liu L, Dong H, Zhao L, Qian Y. High prevalence of GII norovirus in hospitalized children with acute diarrhea, in Beijing. *PLoS One* **2017**, *12*, e0179839.
1011. Jia LP, Qian Y, Zhang Y, Deng L, Liu LY, Zhu RN, Zhao LQ, Huang H, Zheng CG, Dong HJ. Prevalence and genetic diversity of noroviruses in outpatient pediatric clinics in Beijing, China 2010–2012. *Infect Genet Evol* **2014**, *28*, 71–77.
1012. Kim S, Kim YW, Ryu S, Kim JW. Norovirus Outbreak in a Kindergarten: Human to Human Transmission among Children. *Infect Chemother* **2019**, *51*, 171–176.
1013. Kuang X, Teng Z, Zhang X. Genotypic prevalence of norovirus GII in gastroenteritis outpatients in Shanghai from 2016 to 2018. *Gut Pathog* **2019**, *11*, 40.
1014. Li HY, Zhang YG, Lei X, Song J, Duan ZJ. Prevalence of noroviruses in children hospitalized for acute gastroenteritis in Hohhot, China, 2012–2017. *BMC Infect Dis* **2019**, *19*, 595.
1015. Li J, Gao X, Ye YL, Wan T, Zang H, Mo PH, Song CL. An acute gastroenteritis outbreak associated with person–to–person transmission in a primary school in Shanghai: first report of a GI.5 norovirus outbreak in China. *BMC Infect Dis* **2018**, *18*, 316.
1016. Li J, Zhang T, Cai K, Jiang Y, Guan X, Zhan J, Zou W, Yang Z, Xing X, Wu Y, et al. Temporal evolutionary analysis of re–emerging recombinant GII.P16_GII.2 norovirus with acute gastroenteritis in patients from Hubei Province of China, 2017. *Virus Res* **2018**, *249*, 99–109.
1017. Li JS, Qin M, Dong XG, Yang JY, Yang XX, Wei XX, Wang ZE, Feng HR, Wu QR, Li RX, et al. Norovirus outbreaks in Fengtai District, Beijing, China, 2014. *Arch Virol* **2016**, *161*, 2855–2858.
1018. Li Y, Fan X, Yu G, Wei P, Wang Y, Guo H. An acute gastroenteritis outbreak associated with breakfast contaminated with norovirus by asymptotic food handler at a kindergarten in Shenzhen, China. *BMC Infect Dis* **2021**, *21*, 54.
1019. Li Y, Guo H, Xu Z, Zhou X, Zhang H, Zhang L, Miao J, Pan Y. An outbreak of norovirus gastroenteritis associated with a secondary water supply system in a factory in south China. *BMC Public Health* **2013**, *13*, 283.
1020. Li Y, Zhang P, Wu X, Wen D, Ji L, Chen L, Liu G, Fu X, Zhang J, Zhang C, et al. High prevalence of norovirus GII.P16/GII.2 and chicken anemia virus in two acute gastroenteritis outbreaks in Huzhou, China. *Acta Virol* **2019**, *63*, 328–332.
1021. Liu D, Zhang Z, Li S, Wu Q, Tian P, Zhang Z, Wang D. Fingerprinting of human noroviruses co–infections in a possible foodborne outbreak by metagenomics. *Int J Food Microbiol* **2020**, *333*, 108787.

1022. Liu J, Li S, Wang C, Zheng L, Ma J, Li C, Huo Y, Wang Y. Genomic characterization of GII.3 noroviruses isolated from children in Zhengzhou city, China, 2015/16. *Arch Virol* **2018**, *163*, 2737–2742.
1023. Liu P, Wang X, Lee JC, Teunis P, Hu S, Paradise HT, Moe C. Genetic susceptibility to norovirus GII.3 and GII.4 infections in Chinese pediatric diarrheal disease. *Pediatr Infect Dis J* **2014**, *33*, e305–309.
1024. Liu X, Liu P, Wang J, Moe C, Hu S, Cheng L, Gu W, Wang X. Seroepidemiology of Norovirus GII.3 and GII.4 Infections in Children with Diarrhea in Xi'an, China. *Foodborne Pathog Dis* **2015**, *12*, 500–505.
1025. Liu Y, Tam YH, Yuan J, Chen F, Cai W, Liu J, Ma X, Xie C, Zheng C, Zhuo L, et al. A Foodborne Outbreak of Gastroenteritis Caused by Vibrio parahaemolyticus and Norovirus through Non–Seafood Vehicle. *PLoS One* **2015**, *10*, e0137848.
1026. Lu L, Jia R, Zhong H, Xu M, Su L, Cao L, Dong Z, Dong N, Xu J. Molecular characterization and multiple infections of rotavirus, norovirus, sapovirus, astrovirus and adenovirus in outpatients with sporadic gastroenteritis in Shanghai, China, 2010–2011. *Arch Virol* **2015**, *160*, 1229–1238.
1027. Lu L, Zhong H, Xu M, Su L, Cao L, Jia R, Xu J. Genetic diversity and epidemiology of Genogroup II noroviruses in children with acute sporadic gastroenteritis in Shanghai, China, 2012–2017. *BMC Infect Dis* **2019**, *19*, 736.
1028. Lu QB, Huang DD, Zhao J, Wang HY, Zhang XA, Xu HM, Qu F, Liu W, Cao WC. An increasing prevalence of recombinant GII norovirus in pediatric patients with diarrhea during 2010–2013 in China. *Infect Genet Evol* **2015**, *31*, 48–52.
1029. Lu Y, Ma M, Wang H, Wang D, Chen C, Jing Q, Geng J, Li T, Zhang Z, Yang Z. An outbreak of norovirus–related acute gastroenteritis associated with delivery food in Guangzhou, southern China. *BMC Public Health* **2020**, *20*, 25.
1030. Luo LF, Qiao K, Wang XG, Ding KY, Su HL, Li CZ, Yan HJ. Acute gastroenteritis outbreak caused by a GII.6 norovirus. *World J Gastroenterol* **2015**, *21*, 5295–5302.
1031. Mai H, Gao Y, Cong X, Wang H, Liu N, Huang X, Xu L, Chen Y, Wei L. GII.4 Sydney_2012 norovirus infection in immunocompromised patients in Beijing and its rapid evolution in vivo. *J Med Virol* **2016**, *88*, 224–233.
1032. Mai H, Jin M, Guo X, Liu J, Liu N, Cong X, Gao Y, Wei L. Clinical and epidemiologic characteristics of norovirus GII.4 Sydney during winter 2012–13 in Beijing, China following its global emergence. *PLoS One* **2013**, *8*, e71483.
1033. Pan L, Xue C, Fu H, Liu D, Zhu L, Cui C, Zhu W, Fu Y, Qiao S. The novel norovirus genotype GII.17 is the predominant strain in diarrheal patients in Shanghai, China. *Gut Pathog* **2016**, *8*, 49.
1034. Qi L, Xiang X, Xiong Y, Ling H, Shen H, Deng W, Tang W, Shen T, Li Q. Outbreak of Acute Gastroenteritis Caused by Norovirus Genogroup II Attributed to Contaminated Cold Dishes on a Cruise Ship in Chongqing, China, 2017. *Int J Environ Res Public Health* **2018**, *15*, 12.
1035. Wu L. Investigation and analysis of a cluster epidemic of norovirus infection in kindergarten (in Chinese). *Anhui J Prev Med* **2021**, *27*, 141–143.
1036. Qiao N, Wang SM, Wang JX, Kang B, Zhen SS, Zhang XJ, Hao ZY, Ma JC, Qiu C, Zhao YL, et al. Variation analysis of norovirus among children with diarrhea in rural Hebei Province, north of China. *Infect Genet Evol* **2017**, *53*, 199–205.
1037. Qin M, Dong XG, Jing YY, Wei XX, Wang ZE, Feng HR, Yu H, Li JS, Li J. A Waterborne Gastroenteritis Outbreak Caused by Norovirus GII.17 in a Hotel, Hebei, China, December 2014. *Food Environ Virol* **2016**, *8*, 180–186.
1038. Ren Z, Kong Y, Wang J, Wang Q, Huang A, Xu H. Etiological study of enteric viruses and the genetic diversity of norovirus, sapovirus, adenovirus, and astrovirus in children with diarrhea in Chongqing, China. *BMC Infect Dis* **2013**, *13*, 412.
1039. Ruan F, Tan AJ, Man TF, Li H, Mo YL, Lin YX, Deng XL. Gastroenteritis outbreaks caused by Norovirus genotype II.7 in a college in China (Zhuhai, Guangdong) in 2011. *Foodborne Pathog Dis* **2013**, *10*, 856–860.
1040. Sang S, Zhao Z, Suo J, Xing Y, Jia N, Gao Y, Xie L, Du M, Liu B, Ren S, et al. Report of recombinant norovirus GII.g/GII.12 in Beijing, China. *PLoS One* **2014**, *9*, e88210.
1041. Shang X, Fu X, Zhang P, Sheng M, Song J, He F, Qiu Y, Wu H, Lu Q, Feng Y, et al. An outbreak of norovirus–associated acute gastroenteritis associated with contaminated barrelled water in many schools in Zhejiang, China. *PLoS One* **2017**, *12*, e0171307.
1042. Shen H, Zhang J, Li Y, Xie S, Jiang Y, Wu Y, Ye Y, Yang H, Mo H, Situ C, et al. The 12 Gastrointestinal Pathogens Spectrum of Acute Infectious Diarrhea in a Sentinel Hospital, Shenzhen, China. *Front Microbiol* **2016**, *7*, 1926.
1043. Shen W, Sheng Y, Weng J, Li G, Wang D, Qiu D, Lu Y, Lin H. Molecular epidemiology of norovirus associated with acute gastroenteritis in Taizhou, China: A retrospective study. *J Infect Public Health* **2020**, *13*, 34–39.
1044. Shen XX, Qiu FZ, Li GX, Zhao MC, Wang J, Chen C, Zhao L, Qi JJ, Liu H, Zhang Y, et al. A case control study on the prevalence of enterovirus in children samples and its association with diarrhea. *Arch Virol* **2019**, *164*, 63–68.
1045. Shen Z, Qian F, Li Y, Hu Y, Yuan Z, Zhang J. Novel Norovirus GII.4 Variant, Shanghai, China, 2012. *Emerg Infect Dis* **2013**, *19*, 1337–1339.
1046. Shi C, Feng WH, Shi P, Ai J, Guan HX, Sha D, Geng Q, Mei J, Chen SH, Xiao Y, et al. An acute gastroenteritis outbreak caused by GII.17 norovirus in Jiangsu Province, China. *Int J Infect Dis* **2016**, *49*, 30–32.
1047. Sun C, Zhao Y, Wang G, Huang D, He H, Sai L. Molecular epidemiology of GII noroviruses in outpatients with acute gastroenteritis in Shandong Province, China. *Arch Virol* **2021**, *166*, 375–387.
1048. Sun XM, Yan XF, He ZG, Li JX, Guo NJ, Xu ZQ, Jin M, Li DD, Zhou YK, Xie GC, et al. Norovirus Infection and Histo–blood Group Antigens in Children Hospitalized with Diarrhea in Lulong and Chenzhou in China. *Biomed Environ Sci* **2016**, *29*, 286–289.
1049. Tan D, Deng L, Wang M, Li X, Ma Y, Liu W. High prevalence and genetic diversity of noroviruses among children with sporadic acute gastroenteritis in Nanning City, China, 2010–2011. *J Med Virol* **2015**, *87*, 498–503.
1050. Tim K.Tsang, Tian–Mu Chen, Ira M.Longini Jr., M.Elizabeth Halloran, Ying Wu, and Yang Yang. Transmissibility of Norovirus in Urban Versus Rural Households in a Large Community Outbreak in China. *Epidemiology* **2018**, *29*, 675–683.

1051. Zhang JY, Su M, Xu H, Zhuang Y. Analysis and significance of virus testing in children with diarrhea (in Chinese). *Chin J Med Aesth & Cosmet* **2020**, *29*, 57.
1052. Wang LP, Zhou SX, Wang X, Lu QB, Shi LS, Ren X, Zhang HY, Wang YF, Lin SH, Zhang CH, et al. Etiological, epidemiological, and clinical features of acute diarrhea in China. *Nat Commun* **2021**, *12*, 2464.
1053. Wang X, Du X, Yong W, Qiao M, He M, Shi L, Guo B, Hong L, Jiang Y, Xie G, et al. Genetic characterization of emergent GII.17 norovirus variants from 2013 to 2015 in Nanjing, China. *J Med Microbiol* **2016**, *65*, 1274–1280.
1054. Wang X, Wei Z, Guo J, Cai J, Chang H, Ge Y, Zeng M. Norovirus Activity and Genotypes in Sporadic Acute Diarrhea in Children in Shanghai During 2014–2018. *Pediatr Infect Dis J* **2019**, *38*, 1085–1089.
1055. Wang X, Yong W, Shi L, Qiao M, He M, Zhang H, Guo B, Xie G, Zhang M, Jin M, et al. An outbreak of multiple norovirus strains on a cruise ship in China, 2014. *J Appl Microbiol* **2016**, *120*, 226–233.
1056. Zhang XY. Epidemiological and clinical characteristics of pregnant women infected with norovirus (in Chinese). *J Pract Gynec (Electron E)* **2021**, *8*, 5–7.
1057. Wu W, Yang H, Zhang HL, Xian HX, Yao XJ, Zhao DJ, Chen L, Shu BH, Zhou YK, He YQ. Surveillance of pathogens causing gastroenteritis and characterization of norovirus and sapovirus strains in Shenzhen, China, during 2011. *Arch Virol* **2014**, *159*, 1995–2002.
1058. Wu X, Han J, Chen L, Xu D, Shen Y, Zha Y, Zhu X, Ji L. Prevalence and genetic diversity of noroviruses in adults with acute gastroenteritis in Huzhou, China, 2013–2014. *Arch Virol* **2015**, *160*, 1705–1713.
1059. Xu H, Lin Q, Chen C, Zhang J, Zhang H, Hao C. Epidemiology of norovirus gastroenteritis outbreaks in two primary schools in a city in eastern China. *Am J Infect Control* **2013**, *41*, e107–109.
1060. Xue C, Fu Y, Zhu W, Fei Y, Zhu L, Zhang H, Pan L, Xu H, Wang Y, Wang W, et al. An outbreak of acute norovirus gastroenteritis in a boarding school in Shanghai: a retrospective cohort study. *BMC Public Health* **2014**, *14*, 1092.
1061. Xue C, Pan L, Zhu W, Wang Y, Fu H, Cui C, Lu L, Qiao S, Xu B. Molecular epidemiology of genogroup II norovirus infections in acute gastroenteritis patients during 2014–2016 in Pudong New Area, Shanghai, China. *Gut Pathog* **2018**, *10*, 7.
1062. Xue L, Cai W, Gao J, Zhang L, Dong R, Li Y, Wu H, Chen M, Zhang J, Wang J, et al. The resurgence of the norovirus GII.4 variant associated with sporadic gastroenteritis in the post–GII.17 period in South China, 2015 to 2017. *BMC Infect Dis* **2019**, *19*, 696.
1063. Xue L, Dong R, Wu Q, Li Y, Cai W, Kou X, Zhang J, Guo W. Molecular epidemiology of noroviruses associated with sporadic gastroenteritis in Guangzhou, China, 2013–2015. *Arch Virol* **2016**, *161*, 1377–1384.
1064. Xue L, Wu Q, Dong R, Kou X, Li Y, Zhang J, Guo W. Genetic analysis of noroviruses associated with sporadic gastroenteritis during winter in Guangzhou, China. *Foodborne Pathog Dis* **2013**, *10*, 888–895.
1065. Xue Y, Pan H, Hu J, Wu H, Li J, Xiao W, Zhang X, Yuan Z, Wu F. Epidemiology of norovirus infections among diarrhea outpatients in a diarrhea surveillance system in Shanghai, China: a cross–sectional study. *BMC Infect Dis* **2015**, *15*, 1–9.
1066. Yang F, Jiang Y, Yang L, Qin J, Guo M, Lu Y, Chen H, Zhuang Y, Zhang J, Zhang H, et al. Molecular and Conventional Analysis of Acute Diarrheal Isolates Identifies Epidemiological Trends, Antibiotic Resistance and Virulence Profiles of Common Enteropathogens in Shanghai. *Front Microbiol* **2018**, *9*, 164.
1067. Yang S, Li M, Cheng J, Wan G, Zhou Y, Jia H, Wei H, Song R, Sheng L, Wang H, et al. Diagnostic determination of Norovirus infection as one of the major causes of infectious diarrhea in HIV patients using a multiplex polymerase chain reaction assay. *Int J STD AIDS* **2019**, *30*, 550–556.
1068. Yang Z, Wu X, Li T, Li M, Zhong Y, Liu Y, Deng Z, Di B, Huang C, Liang H, et al. Epidemiological survey and analysis on an outbreak of gastroenteritis due to water contamination. *Biomed Environ Sci* **2011**, *24*, 275–283.
1069. Yu J, Ye C, Lai S, Zhu W, Zhang Z, Geng Q, Xue C, Yang W, Wu S, Hall AJ, et al. Incidence of Norovirus–Associated Diarrhea, Shanghai, China, 2012–2013. *Emerg Infect Dis* **2017**, *23*, 312–315.
1070. Zhang DM, Ma MM, Wen WT, Zhu X, Xu L, He ZJ, He X, Wu JH, Hu YW, Zheng Y, et al. Clinical epidemiology and molecular profiling of human bocavirus in faecal samples from children with diarrhoea in Guangzhou, China. *Epidemiol Infect* **2015**, *143*, 2315–2329.
1071. Zhang L, Li X, Wu R, Chen H, Liu J, Wang Z, Xing Y, Ishaq HM, Wang J, Yu P, et al. A gastroenteritis outbreak associated with drinking water in a college in northwest China. *J Water Health* **2018**, *16*, 508–515.
1072. Zhang P, Chen L, Fu Y, Ji L, Wu X, Xu D, Han J. Clinical and molecular analyses of norovirus–associated sporadic acute gastroenteritis: the emergence of GII.17 over GII.4, Huzhou, China, 2015. *BMC Infect Dis* **2016**, *16*, 717.
1073. Zhang S, Chen TH, Wang J, Dong C, Pan J, Moe C, Chen W, Yang L, Wang X, Tang H, et al. Symptomatic and asymptomatic infections of rotavirus, norovirus, and adenovirus among hospitalized children in Xi'an, China. *J Med Virol* **2011**, *83*, 1476–1484.
1074. Zhang SX, Yang CL, Gu WP, Ai L, Serrano E, Yang P, Zhou X, Li SZ, Lv S, Dang ZS, et al. Case–control study of diarrheal disease etiology in individuals over 5 years in southwest China. *Gut Pathog* **2016**, *8*, 58.
1075. Zhang TL, Lu J, Ying L, Zhu XL, Zhao LH, Zhou MY, Wang JL, Chen GC, Xu L. An acute gastroenteritis outbreak caused by GII.P16–GII.2 norovirus associated with airborne transmission via the air conditioning unit in a kindergarten in Lianyungang, China. *Int J Infect Dis* **2017**, *65*, 81–84.
1076. Zhang XF, Chen JR, Song CL, Xie DJ, Tan M, Wang L, Koroma MM, Hou YZ, Dong ZP, Yu JR, et al. Characterization of a hospital–based gastroenteritis outbreak caused by GII.6 norovirus in Jinshan, China. *Epidemiol Infect* **2020**, *148*, e289.
1077. Zhang XF, Huang Q, Long Y, Jiang X, Zhang T, Tan M, Zhang QL, Huang ZY, Li YH, Ding YQ, et al. An outbreak caused by GII.17 norovirus with a wide spectrum of HBGA–associated susceptibility. *Sci Rep* **2015**, *5*, 17687.
1078. Zheng QM, Zeng HT, Dai CW, Zhang SX, Zhang Z, Mei SJ, He YQ, Ma HW. Epidemiological investigation of a norovirus GII.4 Sydney outbreak in a China elder care facility. *Jpn J Infect Dis* **2015**, *68*, 70–74.

1079. Zhou N, Zhang H, Lin X, Hou P, Wang S, Tao Z, Bi Z, Xu A. A waterborne norovirus gastroenteritis outbreak in a school, eastern China. *Epidemiol Infect* **2016**, *144*, 1212–1219.
1080. Zhou X, Kong DG, Li J, Pang BB, Zhao Y, Zhou JB, Zhang T, Xu JQ, Kobayashi N, Wang YH. An Outbreak of Gastroenteritis Associated with GII.17 Norovirus–Contaminated Secondary Water Supply System in Wuhan, China, 2017. *Food Environ Virol* **2019**, *11*, 126–137.
1081. Zhou X, Li H, Sun L, Mo Y, Chen S, Wu X, Liang J, Zheng H, Ke C, Varma JK, et al. Epidemiological and molecular analysis of a waterborne outbreak of norovirus GII.4. *Epidemiol Infect* **2012**, *140*, 2282–2289.
1082. Zhu XH, Tian L, Cheng ZJ, Liu WY, Li S, Yu WT, Zhang WQ, Xiang X, Sun ZY. Viral and Bacterial Etiology of Acute Diarrhea among Children under 5 Years of Age in Wuhan, China. *Chin Med J (Engl)* **2016**, *129*, 1939–1944.
1083. Zhu YN, Ye YH, Zhang Z, Wu YJ, Chen L, Wang J, Tang YJ, Meng J, Zhang HL, Hu GF. Prevalence and molecular characterization of parechovirus A in children with acute gastroenteritis in Shenzhen, 2016–2018. *Arch Virol* **2020**, *165*, 1377–1384.
1084. Zhuang ZL, Jin Y, Yan KL, Cheng WX. Study of the association between histo–blood group antigens and norovirus infection in Chinese children. *Arch Virol* **2017**, *162*, 3511–3515.
1085. Zou W, Cui D, Wang X, Guo H, Yao X, Jin M, Huang Q, Gao M, Wen X. Clinical characteristics and molecular epidemiology of noroviruses in outpatient children with acute gastroenteritis in Huzhou of China. *PLoS One* **2015**, *10*, e0127596.
1086. Cui XS, Xu XW. A comparative study on the prevalence and clinical characteristics of norovirus and rotavirus in children. The 18th National Pediatric Academic Conference of the Chinese Medical Association; Changsha; 2013.
1087. Hou J, Xue WJ, Tian PX, Sun YQ, Feng XS, Xiang HL, Guo Q, He XL. Causes and Treatment of Diarrhea in Patients After Kidney Transplantation. 2012 China Organ Transplant Congress; Xiamen; 2012.
1088. Liu Q, Gao J, Ma HL. Investigation on an outbreak of gastroenteritis caused by norovirus in Zhejiang Province in 2011. The Second Academic Conference of the Public Health Branch of the Chinese Medical Association; Beijing; 2011.
1089. Pang BB, Wang YH, Zhou X. Surveillance of diarrhea virus in patients with gastroenteritis in Wuhan in 2011. The 3rd China Congress of Clinical Microbiology; Hangzhou; 2012.
1090. Wang GZ. Epidemiological analysis of pathogens of acute diarrhea in infants and young children in Hangzhou and surrounding towns. 2014 Zhejiang Provincial Laboratory Medicine Annual Conference; Hangzhou; 2014.
1091. Xu HB, Ruan HJ, Pan YQ. Investigation and analysis of an outbreak of water-borne norovirus infectious diarrhea. The 13th Fuzhou Association for Science and Technology Annual Conference; Fuzhou; 2015.
1092. Xu SJ, Quan DF, Sun HJ, Bai JT, Li YH, Cai JH, He XM. Surveillance of viral diarrhea in Longhua District Shenzhen in 2014. The 6th Infectious Disease Prevention and Control Technology Research and Application Technology Forum; Yanbian; 2015.
1093. Yao WL, Cai JH, Zhu QR, Ceng M. Surveillance of norovirus-associated diarrhea in children in Shanghai, 2009-2011. The 18th National Pediatric Academic Conference of the Chinese Medical Association; Changsha; 2013.
1094. Ai XM. Pathogen distribution and risk factors analysis in elderly patients with diarrhea. The 12th National Clinical Microbiology Academic Conference of the Chinese Medical Association; Lanzhou; 2015.
1095. Chen FQ, Huang YJ, Zhuo L, Lin WD, Ye LY, Wu BS. Analysis of clinical characteristics of 205 cases of children with norovirus enteritis. The 10th National Academic Conference on Children's Digestive System Diseases; Quanzhou; 2014.
1096. Li YP, Yang HS, Zhang HY, Wang M, Li QF, Wang J, Song WJ, Gao DQ. An Investigation of Acute Gastroenteritis Outbreak Caused by Norovirus GII Infection in a University. 2019 4th International Conference on Public Health and Medical Sciences; Xi'an; 2019.
1097. Li Y, Yao JX, Yan YJ, Gu MH. Outbreak investigation of gastroenteritis caused by norovirus in Xinqiao Middle School of Jiangyin City in 2017. Healthy China - The 4th Asia-Pacific International Conference on Health Emergency and Rescue; Shenzhen; 2018.
1098. Lu XD, Wang Q, Zhang YH. Detection and genotype analysis of norovirus diarrhea in Shenzhen area. 2012 National Symposium on Clinical Microbiology and Infection Immunity; Chongqing; 2012.
1099. Sun LM, Li H, Tan XH. Epidemiology and etiology of diarrheal disease among children under 5 years of age in Guangdong province. Hot Symposium on Prevention and Control of Emerging Infectious Diseases; Guangzhou; 2014.
1100. Wang Q, He Y, Zhang YH, Lu XD. Norovirus detection and genotyping for children with acute diarrhea in autumn and winter in Shenzhen. The 4th China Congress of Clinical Microbiology; Wuxi; 2013.
1101. Xiang LH, Mao JY, Yuan GP, Gao Y, Shen JQ, Wang J, Yang XT. Epidemiological investigation on an outbreak of Norovirus gastroenteritis in a school of Shanghai City. The 2nd Asia-Pacific International Conference on Health Emergency Strategies and Capacity Research; Nanjing; 2016.
1102. Zhu GXL, Cui DW, Zheng SF, Chen LX, Yu F, Chen Y, Wu YP, Jin M, Duan ZJ, Xu SJ. Molecular epidemiological characteristic of Caliciviruses of gastroenteritis diarrhea in Hangzhou from 2009 to 2010. 2011 Zhejiang Provincial Laboratory Medicine Annual Conference; Ningbo; 2011.
1103. Zou Y. Field investigation and analysis of an outbreak of norovirus diarrhea in a middle school. The 19th Rural Health Reform and Development Academic Conference of Zhejiang Province; Shaoxing; 2011.
1104. Ceng H. Study on epidemiology and pathogen composition of other infectious diarrhea in Hubei Province, 2016–2017. Master, Wuhan University of Science and Technology; 2019.
1105. Chen C. Molecular epidemiology study of pediatric norovirus gastroenteritis in chongqing, 2015. Master, Chongqing Medical University; 2016.
1106. Dang W. Study on the pathogenic spectrum of viral diarrhea and the molecular epidemiology of norovirus in Ningxia from 2016 to 2018. Master, Ningxia Medical University; 2019.
1107. Han N. Surveillance and Analysis of Norovirus in MaoZhou river and in its nearby Acute gastroenteritis patients in Shenzhen, China. Master, Huazhong University of Science and Technology; 2016.

1108. He F. Molecular Epidemiological Analysis of Norovirus Infection in Children in Tianjin, 2018. Master, Tianjin Medical University; 2020.
1109. He YQ. Study on Molecular Epidemiology of Norovirus Infection and Its Interaction with Host Receptor in Shenzhen. Ph.D Dissertation, Southern Medical University; 2017.
1110. Hu TT. The epidemiological study of viral diarrhea in sentinel hospitals of Guangzhou from 2011–2012. Master, Southern Medical University; 2013.
1111. Huang DD. Molecular epidemiology study of the viral pathogens and an emerging pathogen in adult with acute diarrhea in winter and spring in Beijing, 2010–2011. Master, Anhui Medical University; 2015.
1112. Huang YW. Genotype analysis of Norovirus infected among children in Fuzhou and preliminary establishment of capture ELISA for genogroup II Norovirus detection. Master, Fujian Medical University; 2018.
1113. Ji XF. The surveillance of viral diarrhea among migrant residents in Baiyun district, Guangzhou city, 2010–2011. Master, Southern Medical University; 2012.
1114. Li WW. Epidemiological characteristics of foodborne diseases based on sentinel hospitals in Jining, 2016–2017. Master, Shan Dong University; 2020.
1115. Liu C. Epidemiological characteristics of norovirus in Suzhou,2011–2013. Master, Suzhou University; 2017.
1116. Liu Y. Study on Norovirus Infection and Genotype of Children in a Hospital of Shenyang City from 2017 to 2018. Master, China Medical University; 2019.
1117. Luo C. The surveillance of diarrhea caused by rotavirus and norovirus among community residents in Baiyun district, Guangzhou. Master, Southern Medical University; 2011.
1118. Ma Y. Etiology and Clinical Characteristics Study on Norovirus Diarrhea in 52 Children. Master, Jilin University; 2014.
1119. Qian MM. Establishment of RT–PCR techniques for the detection of Norovirus and the epidemiological investigation in some areas of Jiangsu. Master, Yangzhou University; 2015.
1120. Sai LT. A study on the feature of norovirus infection in adults and the comparison between rotavirus infection and norovirus infection in children in Ji' nan. Ph.D Dissertation, Shan Dong University; 2014.
1121. Sang SW. Molecular epidemiological study of norovirus in acute gastroenteritis. Master, Shan Dong University; 2012.
1122. Shen Z. Molecular epidemiology of norovirus associated with acute gastroenteritis in Shanghai and the preliminary investigation of evolutionary mechanisms of the GII.4 epidemic strains. Master, Fudan University; 2014.
1123. Su C. Pathogen spectrum and related factors of acute infectious diarrhea in Tianjin during 2017–2019. Master, Tianjin Medical University; 2020.
1124. Tan ZM. A study on norovirus infection of children with diarrhea in a grade three first class hospital. Master, Xinjiang Medical University; 2018.
1125. Tang ZJ. Epidemiological characteristics and genotyping of group A rotavirus in infants with diarrhea in Qinghai Province. Master, Shanxi Medical University; 2013.
1126. Wang HY. Molecular Epidemiology of Noroviruses Based on the Sentinel Hospitals Surveillance of Foodborne Disease in Shandong Province. Master, Shan Dong University; 2017.
1127. Wang P. Study on the Association between Histo–Blood Group Antigen and viral diarrhea of children. Master, Nanjing University; 2015.
1128. Wang YG. Molecular Epidemiology Characteristics of Caliciviruses in Patients with Diarrhea in Shenzhen, 2009. Master, Southern Medical University; 2011.
1129. Wu J. The incidence and clinical characteristics of norovirus infection in 181 children with acute diarrhea. Master, Jilin University; 2017.
1130. Wu W. Study on molecular epidemiology of Norovirus about Shenzhen area from 2010 to 2011. Master, Huazhong University of Science and Technology; 2013.
1131. Xue CC. Clinical Characteristics and Follow–up study in Children with Benign Infantile Convulsions with Mild Gastroenteritis. Master, Wenzhou Medical University; 2017.
1132. Zhong XF. Molecular epidemiology of viral diarrhea of children in Chongqing. Ph.D Dissertation, Chongqing Medical University; 2014.
